# Transcript‐ and annotation‐guided genome assembly of the European starling

**DOI:** 10.1111/1755-0998.13679

**Published:** 2022-07-18

**Authors:** Katarina C. Stuart, Richard J. Edwards, Yuanyuan Cheng, Wesley C. Warren, David W. Burt, William B. Sherwin, Natalie R. Hofmeister, Scott J. Werner, Gregory F. Ball, Melissa Bateson, Matthew C. Brandley, Katherine L. Buchanan, Phillip Cassey, David F. Clayton, Tim De Meyer, Simone L. Meddle, Lee A. Rollins

**Affiliations:** ^1^ Evolution & Ecology Research Centre, School of Biological, Earth and Environmental Sciences UNSW Sydney Sydney New South Wales Australia; ^2^ Evolution & Ecology Research Centre, School of Biotechnology and Biomolecular Sciences UNSW Sydney Sydney New South Wales Australia; ^3^ School of Life and Environmental Sciences The University of Sydney, Sydney New South Wales Australia; ^4^ Department of Animal Sciences, Institute for Data Science and Informatics The University of Missouri Columbia Missouri USA; ^5^ Office of the Deputy Vice‐Chancellor (Research and Innovation) The University of Queensland Brisbane Australia; ^6^ Department of Ecology and Evolutionary Biology Cornell University New York USA; ^7^ Fuller Evolutionary Biology Program Cornell Lab of Ornithology New York USA; ^8^ United States Department of Agriculture, Animal and Plant Health Inspection Service, Wildlife Services National Wildlife Research Center Fort Collins Colorado USA; ^9^ Department of Psychology University of Maryland Maryland USA; ^10^ Institute of Neuroscience Newcastle University Newcastle upon Tyne UK; ^11^ Section of Amphibians and Reptiles Carnegie Museum of Natural History Pittsburgh Pennsylvania USA; ^12^ School of Life and Environmental Sciences Deakin University Waurn Ponds Victoria Australia; ^13^ Invasion Science & Wildlife Ecology Lab University of Adelaide Adelaide Australia; ^14^ Department of Genetics & Biochemistry Clemson University South Carolina USA; ^15^ Department of Data Analysis & Mathematical Modelling, Faculty of Bioscience Engineering Ghent University Ghent Belgium; ^16^ The Roslin Institute, The Royal (Dick) School of Veterinary Studies The University of Edinburgh Midlothian UK

**Keywords:** full‐length transcripts, genome annotation, genome assembly, genome assessment, *Sturnus vulgaris*

## Abstract

The European starling, *Sturnus vulgaris*, is an ecologically significant, globally invasive avian species that is also suffering from a major decline in its native range. Here, we present the genome assembly and long‐read transcriptome of an Australian‐sourced European starling (*S. vulgaris* vAU), and a second, North American, short‐read genome assembly (*S. vulgaris* vNA), as complementary reference genomes for population genetic and evolutionary characterization. *S. vulgaris* vAU combined 10× genomics linked‐reads, low‐coverage Nanopore sequencing, and PacBio Iso‐Seq full‐length transcript scaffolding to generate a 1050 Mb assembly on 6222 scaffolds (7.6 Mb scaffold N50, 94.6% busco completeness). Further scaffolding against the high‐quality zebra finch (*Taeniopygia guttata*) genome assigned 98.6% of the assembly to 32 putative nuclear chromosome scaffolds. Species‐specific transcript mapping and gene annotation revealed good gene‐level assembly and high functional completeness. Using *S. vulgaris* vAU, we demonstrate how the multifunctional use of PacBio Iso‐Seq transcript data and complementary homology‐based annotation of sequential assembly steps (assessed using a new tool, saaga) can be used to assess, inform, and validate assembly workflow decisions. We also highlight some counterintuitive behaviour in traditional busco metrics, and present buscomp, a complementary tool for assembly comparison designed to be robust to differences in assembly size and base‐calling quality. This work expands our knowledge of avian genomes and the available toolkit for assessing and improving genome quality. The new genomic resources presented will facilitate further global genomic and transcriptomic analysis on this ecologically important species.

## 1 INTRODUCTION

The European starling (*Sturnus vulgaris*) is a globally invasive passerine that was deliberately introduced during early European acclimatization efforts into North America, Australia, New Zealand, and South Africa during the mid‐late 19th century (Feare, 1984). More recently, the species was accidentally introduced into South America (Palacio et al., 2016). Since these introductions the invasive ranges of the starling have been expanding, with the species now occupying a range in excess of 38,400,000 km^2^ globally (BirdLife International, 2020), posing threats to the economics and health of the agriculture industry, as well as local biodiversity (Bomford & Sinclair, 2002; Koch et al., 2009; Linz et al., 2017; Palacio et al., 2016). Recent molecular ecology studies of individuals from the invasive ranges of North America, Australia, and South Africa report that these populations are undergoing rapid and independent evolution in response to novel local selection pressures (Bodt et al., 2020; Hofmeister et al., 2021; Phair et al., 2018; Stuart et al., 2021), a common phenomenon in many invasive populations (Prentis et al., 2008). This suggests the starling has a flexible invasion strategy, potentially enabling colonization of ecosystems vastly different from those in their native range.

Despite their invasive range success, European starlings are increasingly of ecological concern within their native range (Rintala et al., 2003; Robinson et al., 2005). High densities of native range starlings have traditionally been supported by cattle farming across Europe, because starlings preferentially feed in open grasslands, and benefit from invertebrates in overturned soil produced by livestock grazing (Coleman, 1977). A shift towards modern indoor cattle rearing processes across Europe may contribute to the decline in starling numbers, which has been a concern since the 1980s (Wretenberg et al., 2006). This decline is reflected globally, with starling and other avifauna numbers decreasing sharply over the last few decades (Rosenberg et al., 2019; Spooner et al., 2018), though this may be further amplified for starling populations subjected to control strategies to reduce their economic impact (Linz et al., 2007). The biological and ecological importance of this species is evident from its prolific use in research, as it is the most studied nondomesticated passerine (Bateson & Feenders, 2010). It is evident that future research on the European starling will focus on identifying patterns of evolutionary diversification, and investigating genes associated with invasion success. Such research provides important information for the improvement of control measures and may also provide insight into recovery and dispersive potential in other species that would benefit global conservation efforts. For this, a high‐quality, annotated reference genome is essential.

Once reliant on large consortia, assembling high‐quality reference genomes for genetic analyses is now commonplace. Nevertheless, de novo assembly of nonmodel organism genomes still poses many challenges, as basic information such as genome size, repeat landscape, and ploidy may be unknown. Whilst not always documented in final publications, the standard practice for nonmodel species genomes is to select from multiple assemblies generated using different assembly methods, none of which is universally best (Montoliu‐Nerin et al., 2020; Rhie et al., 2021; Whibley et al., 2020). This complexity can be magnified further when sequencing occurs across multiple technology platforms that may be combined and utilized in different ways (Jayakumar & Sakakibara, 2019; Kono & Arakawa, 2019). A multitude of tools and approaches are available for genome assembly assessment during this process. Common approaches employed to guide genome assembly decisions focus on contiguity (how continuous the assembled sequences are), such as assembly statistics contig/scaffold counts and L50/N50, and completeness (whether the assembly contains all the genetic information for that species) such as benchmarking universal single copy orthologues (BUSCO) estimates of genome completeness (Simão et al., 2015). Benchmarking approaches must be used in consort as each has benefits and drawbacks: assembly statistics are easy to generate, but hidden assembly errors and artefacts may confound signals of improvement; busco provides a standardized comparison point but is prone to stochastic errors (see Box 1) and genome coverage is limited to “easy” to assemble regions (Peona et al., 2021). In addition, general benchmarking methods do not explicitly test the genome assembly's ability to perform the role for which it was intended (e.g., to serve as a reference genome for specific genomic analysis). Because of this, assembly benchmarking approaches are expanding to cover previously hard to assemble regions (e.g., Peona et al., 2021). While some may not be applicable or feasibly implemented for a particular species/assembly and/or the data available (e.g., Bradnam et al., 2013; Hunt et al., 2013; Ou et al., 2018; Yuan et al., 2017), often these new tools severe the dual purpose of informing assembly decisions and characterizing important biological aspects of the species' genome itself.

Here, we present the first official European starling draft genome, releasing two assemblies: *S. vulgaris* vAU and *S. vulgaris* vNA. This manuscript focuses primarily on the newer genome assembly of *S. vulgaris* vAU, which represents the first synthesis of species‐specific full‐length transcripts, together with linked‐ and long‐read genomic data for this species. In this study, we examine how a diverse range of assembly benchmarking tools, including transcriptome, annotation, and repeat based assessment approaches, help determine genome assembly quality and completeness. In doing so, we also release two new benchmarking tools: (1) BUSCO Compilation and Comparison tool (buscomp) can help avoid overinterpretation or misinterpretation of small differences in busco completeness; (2) Summarize, annotate and assess genome annotations (saaga) utilizes a lightweight homology‐based annotation by gemoma (Keilwagen et al., 2018) to provide genome‐wide feedback on gene prediction quality. Finally, we take to opportunity to contrast our two starling assemblies, enabling reference‐specific biases to be identified in future genomics studies.

## 2 MATERIALS AND METHODS

### 2.1 Transcriptome assembly and analysis

We processed the raw PacBio Iso‐Seq whole transcript reads (Appendix S1: Transcriptome sample collection, RNA extraction, and sequencing) using the protocol outlined in smrt link (version 9.0) (PacBio). Briefly, this involved generating circular consensus sequences (CCS) using ccs (version 4.2.0), which we then processed using Lima (version 1.11.0) for primer removal and demultiplexing. We further processed the reads (PolyA tail minimum length = 8) and clustered them using iso‐seq (version 3.3). We aligned the high‐quality clustered Iso‐Seq reads to the reference genome (see section 2.1 Genome assembly and scaffolding) using minimap2 (version 2.17) (Li, 2018), before we further processed them using tama collapse (Kuo et al., 2020) (settings ‐a 100 ‐z 30 ‐sj sj_priority ‐lde 5). We assessed both of these steps using busco (version 3.0.2b) (Simão et al., 2015) (parameters: aves lineage, transcriptome mode), alongside a short read transcriptome produced from *S. vulgaris* liver RNA (Richardson et al., 2017), as well as other available avian Iso‐Seq transcriptomes (Workman et al., 2018; Yin et al., 2019). Computational steps for this and all further sections were carried out on the UNSW Sydney cluster Katana (PVC Research Infrastructure, 2010).

### 2.2 Genome assembly and scaffolding

**FIGURE 1:**
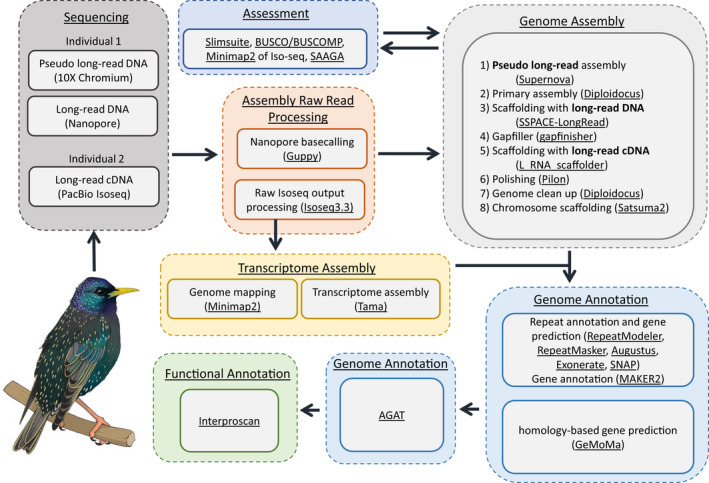
Workflow for genome assembly and annotation. A summary of all the experimental methods used for sequencing, genome assembly, transcriptome assembly, genome annotation, and functional annotation, with programs used underlined

To create the *S. vulgaris* vAU genome assembly we used 10x chromium linked reads and low coverage ONT long reads (Appendix S2: Genomic DNA sample collection, gDNA extraction, and sequencing) via eight assembly steps (Figure 1). We assembled the 10x reads into an initial diploid assembly using supernova (version 2.1.1) (Weisenfeld et al., 2017) with barcode fraction and reads subsample calculated following supernova best practices for a genome size based on k‐mer counts calculation by jellyfish (version 2.2.10) (Marçais & Kingsford, 2011) (parameters: bcfrac = 0.8, maxreads = 550 million, Appendix S3, Validation of supernova genome size prediction using jellyfish, Figure S1). We then split this assembly into nonredundant primary and alternative haploid assemblies using diploidocus (parameters: runmode = diphapnr) (version 0.9.5) ( https://github.com/slimsuite/diploidocus). diploidocus creation of a primary assembly is completed by first combining both supernova pseudohap2 assemblies and removing any sequences that lack definitive base calls (100% Ns). Remaining scaffolds were size‐sorted and gaps reduced in size to a maximum of 10 Ns then subject to an all‐by‐all search with minimap2 (version 2.17) (Li, 2018) (−‐cs ‐p 0.0001 ‐x asm20 ‐N 250). (Note that gap size reduction is used for minimap2 searching only, and the nonredundant pseudodiploid assembly produced has the same gap sizes as generated by supernova.). Any sequences that were 100% contained within another sequence are removed. Where two or more scaffolds had an 100% identical sequence, only one is kept. Scaffolds are then matched into haplotig pairs based on their supernova names. Where a single haplotig is found, it is assigned as diploid, under the assumption that the two original haplotigs were identical with one removed, and added to the primary assembly (note: it is possible that only one parent had this scaffold, e.g., a sex chromosome scaffold or structural variant.). If two haplotigs are identified, the longest is assigned to the primary assembly and the shorter to the alternative assembly. The primary assembly should therefore contain an entire haploid copy of the genome, whilst the alternative assembly contains the subset of scaffolds with heterozygous haplotigs.

We then scaffolded the primary haploid assembly produced by diploidocus using the filtered ONT reads using the program sspace‐longread (version 1–1) (Boetzer & Pirovano, 2014). Following this, we gap‐filled the assembly with the ONT reads and the program gapfinisher (version 1.0) (Kammonen et al., 2019). We processed the assembly through a second round of scaffolding, this time using the clustered high‐quality Iso‐Seq reads (see section 2.1 Transcriptome assembly and analysis) and the program l_rna_scaffolder (Xue et al., 2013). We processed the paired‐end 10x linked reads with 10x Genomics long ranger (version 2.2) and mapped onto this scaffolded assembly using bwa mem before error correction of SNPs and indels using pilon (version 1.23) (Walker et al., 2014) (parameters: ‐‐diploid –fix all settings). We validated the scaffolds by analysing the assembly using the break10x toolkit in scaff10x (version 3.1) (https://github.com/wtsi‐hpag/Scaff10X). We further checked the assembly for assembly artefacts and contamination using diploidocus (parameters: runmode = purgehaplotig & runmode = vecscreen; runmode = DipCycle was tested yet discarded due to over‐pruning, see: Figure S2) (version 0.9.5) (Chen et al., 2022). Avian species are characterized by distinctive and constrained karyotypes, generally comprised of approximately 10 macrochromosomes and approximately 30 indistinguishable microchromosomes (Griffin et al., 2007; O'Connor et al., 2019), a pattern to which the *S. vulgaris* genome conforms (Calafati & Capanna, 1981). In the absence of genome‐wide linkage data (e.g., Hi‐C data) to assist with long‐range scaffolding, we used a synteny based approach, which enables the identification of putative chromosomes, often helpful for downstream analyses (e.g., analysis of sex vs. autosomal genetic variants). We aligned our assembly to the chromosome scale assembly of zebra finch (*Taeniopygia guttata*) (NCBI = GCF_008822105.2) (Rhie et al., 2021) using the satsuma2 Chromosemble function (https://github.com/bioinfologics/satsuma2) to create super‐scaffolds that could be assigned putative chromosome identifiers through assumed orthology. This assembly formed the final updated draft genome we present for the species: *Sturnus vulgaris* vAU.

### 2.3 Genome assembly completeness assessment

In addition to genome statistics, we used several approaches to assess assembly contiguity and completeness for sequential genome assembly steps of the *S. vulgaris* vAU assembly, as well as the *S. vulgaris* vNA genome (assembly accession GCF_001447265.1, Supporting Information Material: Appendix S4, Assembly and annotation of the *S. vulgaris* vNA genome version).

#### 2.3.1 busco and buscomp assembly completeness assessment

We estimated genome completeness using busco (version 3.0.2b, genome mode, Aves lineage). We collated the busco results across all genome assembly stages using buscomp (version 0.10.1) (https://github.com/slimsuite/buscomp) (Box 1: busco versus buscomp performance benchmarking), which compiled a maximal nonredundant set of 4789 complete buscos found at single copy in at least one assembly. Compiled busco predicted gene sequences were mapped onto each assembly to be rated with minimap2 (version 2.17) (Li, 2018) and rescored in terms of completeness, thereby providing a robust and consistent means of assessing comparable completeness across assemblies of the same genome.

We additionally ran busco on four other existing passerine chromosome‐level assemblies available on NCBI to compare to *S. vulgaris* vAU and vNA.

#### 2.3.2 PacBio Iso‐Seq completeness assessment

We mapped the PacBio Iso‐Seq reads on to genome assemblies using minimap2 (parameters: ‐axe splice ‐uf ‐‐secondary = no ‐‐splice‐flank = no ‐C5 ‐O6,24 ‐B4) (Li, 2018) and calculated the number of Iso‐Seq transcripts mapping on to each assembly, and their corresponding mapping quality.

#### 2.3.3 Assessment of predicted protein completeness using saaga

We used our newly released tool saaga (https://github.com/slimsuite/saaga) to assess the annotation quality of the genome assemblies. This involved an initial annotation using gemoma (version 1.7.1) (Keilwagen et al., 2018) following the protocol described in the final assembly annotation (see section 2.4: Genome annotation and functional annotation), followed by assessment using summarize, annotate and assess genome annotations (saaga) with the repeat‐filtered Swiss‐Prot database used as the benchmarking database (see Box 2: Annotation assessment using saaga).

#### 2.3.4 Analysis of assembly completeness using the MHCIIB gene

We assessed the completeness of the harder to assemble regions of the genome by assessing the highly variable exon 2 and conserved exon 3 of the MHCIIB gene complex, following a similar protocol to that established in Peona et al. (2021). Briefly, we used existing avian MHCIIB exon alignments (Goebel et al., 2017), filtered the aligned sequences excluding those that fell underneath a minimum length threshold (minimum size, exon 2: 270 bp, exon 3: 215 bp), and blast searched these against the sequential assembly steps, merging any overlapping intervals. We then blast searched the obtained intervals against GenBank, and retained only matches to the search term “MHC class II beta”. Finally, we aligned the remaining sequences using mafft (version 7.407) (Katoh et al., 2002), and filtered for sequences covering a minimum length of the alignment (exon 2: 220 bp, exon 3: 185 bp). Finally, we manually curated the sequence list to prune out non‐MHCIIB sequences from the alignment file.

#### 2.3.5 Assembly repeat content assessment

We assessed the repeat content of each genome assembly step with repeatmasker version 4.0.7 (Smit et al., 2013), using a joint repeat library of the Aves lineage specific sequence, combined with the custom generated repeat library generated during genome annotation (see section 2.4 Genome annotation and functional annotation).

#### 2.3.6 kat k‐mer completeness assessment

We assessed overall genome assembly completeness by examining the read k‐mer frequency distribution with different assembly copy numbers based on the 10x chromium linked reads using k‐mer analysis toolkit (kat) version 2.4.2 (Mapleson et al., 2017) (30 bp trimmed for R1 reads, and 16 bp trimmed for R2 reads).

BOX 1 **busco versus buscomp performance benchmarking**

busco (Simão et al., 2015) is an extremely useful and widely‐used used assembly assessment tool, providing information on which conserved lineage specific genes are present, fragmented, or absent from a genome assembly. The program, however, can suffer from inconsistent busco gene identification, where a particularly busco may be dropped from a report due to changes elsewhere in the assembly (Edwards, 2019), which can result in under‐reporting of assembly completeness (Edwards et al., 2018, 2021; Field et al., 2020). We therefore present in this manuscript the tool buscomp (https://github.com/slimsuite/buscomp) and showcase it in this manuscript through analysis of sequential assembly steps to gain a more accurate understanding of how assembly decisions affected genome completeness (see Section 2.3.1 busco and buscomp assembly completeness assessment). We also have provided buscomp software performance benchmarking (Supporting Information Material: Appendix S5: busco vs. buscomp performance benchmarking), for which we briefly discuss the results directly below.

We confirmed the stochastic busco behaviour on benchmarking data sets derived from the *S. vulgaris* vAU pseudodiploid 10x linked read assembly (Figures S3, S4). Adding and removing scaffolds can both alter the busco ratings for “Complete” genes within the unchanged scaffolds (Figure S4, Supporting Information File S1, buscomp version 3 results, Supporting Information File S2, buscomp version 5 results). Many of these changes are likely to be the consequence of changes in score thresholds and/or gene prediction models. However, we also demonstrated some unexpected behaviours that are harder to explain, such as changes to busco gene ratings when scaffolds are reverse complemented (Figure S4).

This unpredictable variability in the identification of buscos across genome assembly versions poses some obvious challenges when trying to compare alternate versions of the same assembly. This is particularly true when trying to interpret small changes in busco ratings as assemblies near completion. In addition, an important feature of busco is that it incorporates sequence quality in the context of the gene prediction models it generates. This is desirable for assessing final assembly quality, but can present problems when comparing early assembly stages, prior to error‐correction by “polishing”. buscomp is robust to differences in assembly size, base‐calling quality, and rates the “completeness potential” of an assembly based on the presence of genes first identified for that species by busco. buscomp analysis can then be complemented by other tools, such as kat (Mapleson et al., 2017), merqury (Rhie et al., 2020), saaga (Box 2), and busco itself to get additional assessment of sequence quality.

### 2.4 Genome annotation and functional annotation

We annotated the final *S. vulgaris* vAU genome assembly using gemoma (version 1.7.1) (Keilwagen et al., 2018) and the 26 avian genome annotations available on Ensembl at the time this analysis was conducted (Table S1) and with the high‐quality clustered Iso‐seq, as RNA evidence. We ran the gemoma GeMoMaPipeline function to complete the full pipeline with a maximum intron size of 200 kb (parameters: tblastn = false gemoma.m = 200,000 gemoma.Score = ReAlign AnnotationFinalizer.r = SIMPLE pc = true o = true).

We also annotated the final *S. vulgaris* vAU genome assembly with maker2 (Holt & Yandell, 2011), BLAST+ (version 2.9) (Camacho et al., 2009), augustus (version 3.3.2) (Stanke & Morgenstern, 2005), exonerate (version 2.2.0) (Slater & Birney, 2005), repeatmasker (version 4.0.7) (Smit et al., 2013), repeatmodeler (version 1.0.11) (http://www.repeatmasker.org/RepeatModeler/), and snap (version 0.15.4) (Korf, 2004) using repeat‐filtered Swiss‐Prot protein sequences (downloaded August 2018) (UniProt Consortium, 2019). We created a custom augustus species database by running busco using the Optimization mode Augustus self‐training mode (−‐long), using the aves database for lineage. We ran maker2 using the recommended protocol, including generation of a repeat library (following the maker2 advanced repeat library construction protocol (http://weatherby.genetics.utah.edu/MAKER/wiki/index.php/Repeat_Library_Construction‐Advanced) with MITES identified using mitetracker (Crescente et al., 2018)), and with the tama‐processed Iso‐Seq data included as primary species transcript evidence, and the pre‐existing short read liver transcript data (Richardson et al., 2017) provided as alternate transcript evidence in the first iteration of the maker2 annotation process. We ran maker2 for a total of three training runs, using the hidden Markov models (HMMs) produced from snap training in each subsequent run. Ab initio genes were not retained in the final annotation model to produce high quality and conservative gene predictions. We combined the gemoma and maker2 annotations for the final *S. vulgaris* vAU assembly using the agat agat_sp_merge_annotations function to produce the final annotation. We generated functional annotation of protein‐coding genes using interproscan 5.25–64.0 (parameters: ‐dp ‐goterms ‐iprlookup ‐appl TIGRFAM, SFLD, Phobius, SUPERFAMILY, PANTHER, Gene3D, Hamap, ProSiteProfiles, Coils, SMART, CDD, PRINTS, Pro SitePatterns, SignalP_EUK, Pfam, ProDom, MobiDBLite, PIRSF, TMHMM). We used blast to annotate predicted genes using all Swiss‐Prot proteins (parameters: ‐evalue 0.000001 ‐seg yes ‐soft_masking true ‐lcase_masking ‐max_hsps). We generated annotation summaries using the agat agat_sp_functional_statistics.pl script, used bedtools to calculate gene coverage statistics. We assigned gene ontology terms using wego version 2.0 (Ye et al., 2018). We further assessed the quality of the final *S. vulgaris* vAU annotation through saaga (see Box 2: Annotation assessment using saaga), using the repeat‐filtered Swiss‐Prot database used in annotation, as well as the *Gallus gallus* reference proteome (UP000000539_9031), to assess predicted protein quality and annotated proteome completeness.

BOX 2 **Annotation assessment using saaga**

In this manuscript we present summarize, annotate and assess genome annotations (saaga) (version 0.5.3) (https://github.com/slimsuite/saaga), a tool designed to assess annotation quality and compare predicted proteins to the repeat‐ and transposase‐filter Swiss‐Prot protein sequences used for maker2 annotation (above). saaga performs a reciprocal mmseqs2 (Steinegger & Söding, 2017) search of annotated proteins against a (high‐quality) reference proteome, identifying best hits for protein identification and employing coverage ratios between query and hit proteins as a means of annotation assessment to generate summary statistics, including:

- *Protein length ratio*. The length ratio of the annotated proteins versus its top reference hit
- *F1 score*. An annotation consistency metric calculated using the formula:
$$\left(2\times \text{PROTCOV}\times \text{REFCOV}\right)/\left(\text{PROTCOV}\times \text{REFCOV}\right)$$
  where protcov is the proportion of the annotated protein covered by its best reference protein hit, and refcov is the proportion of the best reference protein hit covered by the annotated protein.
- *Completeness*. The summed percentage coverage of reference proteome.
- *Purity*. The summed percentage reference coverage of the annotated proteome.
- *Homology*. The percentage of annotated genes with any hit in reference.
- *Orthology*. The percentage of annotated genes with reciprocal best hits in reference.
- *Duplicity*. The mean number of annotated genes sharing the same best reference hit.
- *Compression*. The number of unique annotated genes that were the top hit for reference proteins, divided by the total number of reference proteins with a hit.
- *Multiplicity*. The ratio of total number of annotated genes to reference proteins.

For protein length ratio and F1 score, values close to 1 means that the query protein closely matches the length of the hit protein, indicating high fidelity of the gene prediction model and underlying assembly. The remaining metrics will be closer to 1 (or 100%) for complete annotations and assemblies without duplications, akin to busco scores. Although the maximum achievable value for these metrics will generally be unknown, comparative values can be used to assess improvement in assembly and/or annotation.

saaga scores may be used to compare alternate annotations of the same assembly, or to compare alternative assemblies in conjunction with consistent annotation. Low genome contiguity, misassembles, or frameshifting indels will affect the quality of predicted genes, with poorer assemblies resulting in more fragmented or truncated genes. This approach has been facilitated by the rapid homology‐based gene prediction program gemoma, which uses reference genome annotation to predict protein‐coding genes in the target genome. The program can be run from one line of code and may be parallelised to run much faster than other annotation software (e.g., maker2). The ease of this annotation tool opens the way for conducting annotations for the purpose of assessment on sequential or even competing genome annotation steps. Assessing the quality of protein‐coding region predictions will help ensure the final genome assembly can produce a high‐quality annotation.

### 2.5 Exploration of the *S. vulgaris* genome

We calculated the Iso‐Seq and final annotation transcript density, final annotation gene density, and GC‐content in sliding windows of width 1 Mb using bedtools (version 2.27.1) (Quinlan & Hall, 2010), and plotted them across the largest 32 super‐scaffolds in our final genome assembly (representing more than 98% of the total assembly captured on putative chromosomes orthologous to other avian chromosomes) using circlize (version 0.4.9) (Gu et al., 2014). Further, we also plotted the locations of the MHCIIB exon 2 and exon 3 from the genome assembly version vAU (genes identified on unplaced scaffold were not plotted). We also calculated and plotted global SNP variant density in the circular plot, based on a whole genome data set of eight individuals each from the UK, North America, and Australia, total *N* = 24 (Hofmeister et al., 2021).

#### 2.5.1 Synteny analysis

We conducted synteny analysis to investigate how the choice of chromosome‐level reference assembly affected the final synteny‐based assembly scaffolding, and also to briefly examine patterns of synteny between the resulting *S. vulgaris* assemblies and other Aves genomes. To achieve this, we repeated step 8 of the assembly process (Figure 1), the satsuma2 Chromosemble function, using three additional genome assemblies as a reference (*Passer domesticus* assembly accession GCA_001700915.1, *Calypte anna* assembly accession GCA_003957555.2, *Parus major* assembly accession GCA_001522545.3). We used the tool chromsyn (https://github.com/slimsuite/chromsyn) in r, which is a busco guided synteny plotting tool, to visualize synteny patterns across these eight genome assemblies (the four Aves references, and the four resulting synteny‐based scaffolded *S. vulgaris* assembly versions).

#### 2.5.2 Transposable element composition and repeat content analysis across *S. vulgaris* vAU and vNA

For de novo transposable element (TE) detection, we ran the *S. vulgaris* vAU and *S. vulgaris* vNA genome assemblies (with no masking) through repeatmodeler2 (Flynn et al., 2020), retaining the raw consensus sequences. Then, we assessed the repeat content of *S. vulgaris* vAU and *S. vulgaris* vNA using repeatmasker version 4.0.7 (Smit et al., 2013), using a joint repeat library of the Aves lineage specific sequence, combined with the newly generated TE libraries for each genome.

### 2.6 Genome assembly correction

NCBI VecScreen flagged possible bacterial and adapter contamination in the final *S. vulgaris vAU* assembly, which was missed by earlier contamination screening steps. Hence we ran an updated version of diploidocus (runmode vecscreen) to mask shorter adapter sequences and flag additional organism contaminates (screenmode = purge vecmask = 27). We identified four related bacterial strains (Delftia acidovorans SPH‐1, Acidovorax sp. JS42, Alicycliphilus denitrificans K601, Paraburkholderia xenovorans LB400), and so used gablam version 2.30.5 (Davey et al., 2006) to search these four genomes against the final assembly, and purge small contigs (<5000 bp) that contained sequence matches (285 short contigs excluded). For larger scaffolds that contained possible embedded contaminated sequences, we mapped the high‐quality ONT reads using Minimap2 over the regions. For those contaminated sites that had Nanopore reads spanning the contaminated region, the sequences were masked, and for those lacking nanopore support, the scaffold was split and/or trimmed to remove the contaminating sequence (seq 4 trimmed, seq 12 and 31 split into chromosome and unplaced scaffold). Finally, gaps of unknown size were standardized to 100 bp, and we assessed mitochondrial genome insertions into the nuclear genome using NUMTfinder (https://github.com/slimsuite/numtfinder) (Edwards et al., 2021) and the published starling mtDNA (Rollins et al., 2011) (none located). This study primarily analyses *S. vulgaris* vAU1.0 (which we refer to as *S. vulgaris* vAU), while the final NCBI release (accession  GCA_023376015.1) is explicitly referred to as *S. vulgaris* vAU1.1 when relevant.

## 3 RESULTS

### 3.1 *Sturnus vulgaris* vAU whole transcriptome data analysis

**FIGURE 2:**
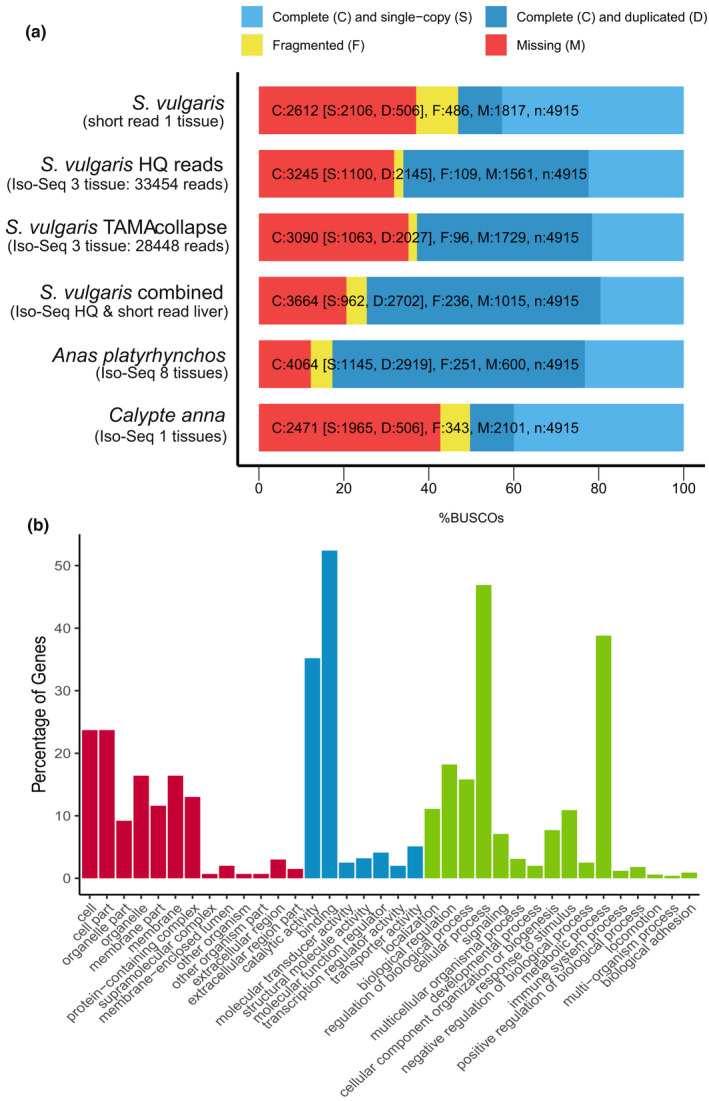
Assessment of 3 tissue Iso‐Seq (brain, gonad, heart) *Sturnus vulgaris* transcriptome. (a) busco (aves) rating summaries for *S. vulgaris* short read liver transcriptome, the high‐quality Iso‐Seq *S. vulgaris* transcript produced though the Iso‐Seq version 3.3 pipeline, the final *S. vulgaris* transcriptome produced by tama collapse pipeline, and combined high‐quality Iso‐Seq and short read liver transcripts, alongside two other avian Iso‐Seq transcriptomes (*Anas platyrhynchos* using pectoralis, heart, uterus, ovary, testis, hypothalamus, pituitary and 13 day‐old embryo tissue (Yin et al., 2019), and *Calypte anna* using liver tissue (Workman et al., 2018)). (b) Breakdown of major gene ontology (GO) terms in the sequenced Iso‐Seq reads, with cellular component (red) molecular function (blue) and biological process (green)

We generated approximately 68 Gb of PacBio Iso‐Seq whole transcript (39,544,054 subreads) (Table 1). This produced a total of 33,454 clustered high‐quality (predicted accuracy ≥0.99) reads, and 157 clustered low‐quality (predicted accuracy <0.99) reads (Table S2). These high‐quality read data were used to improve the scaffold assembly of the genome using l_rna_scaffolder (see section 2.2) and assess genome completeness (using count comparison of unmapped Iso‐Seq reads, see section 2.3.2). After being passed through the tama collapse pipeline, a total of 28,448 nonredundant transcripts were retained to create the final *S. vulgaris* vAU transcriptome, which was used for gene prediction when completing the annotation of the genome assembly. This final three tissue (brain, gonad, heart) Iso‐Seq transcriptome had a moderate level overall busco completeness of around 63% that compares to other avian Iso‐Seq transcriptomes (Figure 2a), with a wide range of gene ontology terms identified in the final Iso‐Seq transcript list (Figure 2b) that resembled other avian Iso‐Seq GO term distributions (Yin et al., 2019).

**TABLE 1:** Summary of sequencing data for *Sturnus vulgaris* vAU genome assembly and annotation

Genetic data	Platform	Library	Library length/mean insert size (kb)	Mean raw read length (bp)	Number of reads	Number of bases (Gb)
gDNA	Hiseq X Ten	Paired‐end 10x chromium	51.7 kb	150	361,950,449	108.58
gDNA	ONT MinION	Ligation	47 kb	6417	1,225,865	7.865
cDNA	PacBio	Iso‐Seq	Full transcripts (brain) (2.6 kb)	12,000	20,558,110	38.650
cDNA	PacBio	Iso‐Seq	Full transcripts (heart + testes) (2.0 kb)	10,000	18,985,944	29.496

### 3.2 *Sturnus vulgaris* vAU genome assembly

To create the genome assembly of *Sturnus vulgaris* vAU, we combined three different sequencing technologies for de novo genome assembly (10× genomics linked reads, ONT long reads, and PacBio Iso‐Seq full length transcripts) (Table 1), before using reference‐based scaffolding to predicted‐chromosome super‐scaffold level using the high‐quality reference assembly of *T. guttata* (NCBI REF: GCF_008822105.2). We created a primary assembly using approximately 109 Gb (97× coverage) of 10× linked read data (subsampled during assembly to 56× based on the estimated genome size of 1.119 Gb, barcode subsampling of 80%) with supernova (version 2.1.1) (Weisenfeld et al., 2017) (Figure S2 step 1) which we then converted to a primary haploid assembly (Figure S2 step2). We generated approximately 8 Gb of raw genomic reads using an ONT minion, which were reduced to 5 Gb after stringent filtering (Table 1). We used these data to scaffold the genome (Figure S2 step 3) and gap‐fill (Figure S2 step 4), reducing the total number of scaffolds from 18,439 to 7856, increasing the scaffold N50 from 1.76 Mb to 7.12 Mb, and decreasing the scaffold L50 from 146 to 39 (Figure S5). We further improved these measures after Iso‐Seq scaffolding (Figure S2 step 5) (7776 scaffolds, N50 7.12 Mb, and L50 38), followed by Pilon polishing using 10x linked reads (Figure S2 step 6). Finally, following haplotig removal (Figure S2 step 7), we used chromosomal alignment against the *T. guttata* reference genome (Figure S2 step 8) to reduce the final number of scaffolds to 1628 (N50 72.5 Mb, and L50 5) (Figure S5), with 98.6% of the assembly assigned to the 32 super‐scaffolds representing predicted chromosomes. While no whole mitochondrial genome insertions were found, 27 smaller mitochondrial pseudogenes (NUMTs) were located in *S. vulgaris* vAU1.1, with super‐scaffold corresponding to the predicted Z chromosome containing the highest amount (Table S3).

#### 3.2.1 Improvements to genome assembly completeness during scaffolding

**FIGURE 3:**
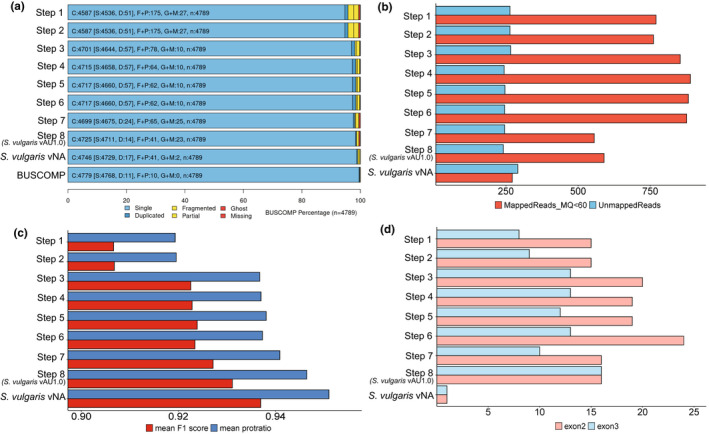
*Sturnus vulgaris* vAU assembly steps overview. Quality and completeness assessments for eight sequential assembly steps: Step 1 (supernova assembly), step 2 (diploidocus primary assembly), step 3 (sspace‐longreads scaffolding), step 4 (gapfinisher gapfilling), step 5 (L_rna_scaffolder), step 6 (pilon polishing), step 7 (diploidocus clean up), and step 8 (satsuma2 chromosome scaffolding; *S. vulgaris* vAU1.0), and *S. vulgaris* vNA. (a) buscomp completeness results for the 4779 busco genes identified as single copy and complete in one or more assembly stages. The final buscomp row compiles the best rating for each gene across all eight steps (complete: 95% + coverage in a single contig/scaffold; duplicated: 95% + coverage in 2+ contigs/scaffolds; fragmented: 95% + combined coverage but not in any single contig/scaffold; partial: 40%–95% combined coverage; ghost: Hits meeting local cutoff but <40% combined coverage; missing: No hits meeting local cutoff). (b) a bar plot of the number of Iso‐Seq reads that for each assembly step failed to map (blue) or fell below a mapQ score of 60 (red). (c) saaga annotation scores of mean protein length ratio (blue) and F1 score (red) (see methods for details). (d) Total count of MHCIIB exon 2 (red) and exon3 (blue) sequences identified in each assembly

Sequential steps of scaffolding, polishing, and quality control (Figure 1, Figure S2, Table S4) improved the genome assembly statistics considerably from the initial supernova *S. vulgaris* assembly (Figure S5). We found that buscomp completeness, compiled from the busco scores of all sequential assembly steps (Figure S6a) was approximately 98.7%, which was largely achieved by the initial assembly (95.8%), but somewhat improved over the additional assembly steps (Figure 3a). We identified that only 70 buscomps (1.4%) of the 4915 Aves busco genes were found to be “Missing” from all assembly versions, with 4779 (97.2%) rated “Complete” in at least one stage or in *S. vulgaris* vNA (Figure 3a).

The final assembly had the fewest unmapped Iso‐Seq reads (Figure 3b), with the largest improvement seen post gap‐filling, followed by chromosome scaffolding. We observed an increase in missing Iso‐Seq transcripts after scaffolding with the Iso‐Seq reads themselves, and post long‐read scaffolding, due to reads no longer partially matching at scaffold ends. Polishing caused a minimal improvement on the total number of mapped Iso‐Seq reads, and none were lost during scaffold clean‐up with diploidocus (runmode purgehaplotig and vecscreen). Of the 33,454 high‐quality isoform transcripts in the PacBio Iso‐Seq data, we found only 241 failed to map to the final genome assembly, a 17.2% decrease compared to the 291 that failed to map to *S. vulgaris* vNA. In contrast, *S. vulgaris* vNA had fewer mapped reads with a mapping quality score below 60 when compared to *S. vulgaris* vAU.

Assessment using gemoma annotation and saaga revealed that across these assembly steps we see a generally consistent increase in the quality of predicted proteins (Figure 3c), with the largest increases occurring post long‐read scaffolding, followed by chromosome scaffolding, and then scaffold clean‐up. Mean predicted gene quality scores were slightly higher for *S. vulgaris* vNA than *S. vulgaris* vAU. We investigated the MHCIIB gene complex as a means of assessing completes of the harder to assemble regions of the genome. We identified more copies of variable exon 2 than conserved exon 3 across all assembly steps, until the final synteny‐based scaffolding (i.e., *S. vulgaris* vAU), which reported 16 copies of both exons. While the exact number of MHCIIB copies within avian genomes is unknown (Miller & Taylor, 2016; Peona et al., 2021), these results stand in sharp contrast to those of *S. vulgaris* vNA, in which we identified only one copy of each exon, consistent with the short‐read assembly collapsing multicopy loci during assembly.

Using assembly repeat content as a means of assessment resulted in similar repeat type profiles across all genome assembly steps, with an increase in overall repeat overage found post gap‐filling, and a decrease following haplotig removal (Figure S6b). Comparison of *S. vulgaris* vAU and *S. vulgaris* vNA repeat landscape can be found below (see section 3.4 *Sturnus vulgaris* genome‐wide patterns of genomics features). K‐mer completeness assessment was found to follow similar patterns to repeat content assessment, with the only major difference being that synteny‐based scaffolding appeared to recover most of the genome coverage that was lost during haplotig removal (Table S4: Assembly statistics summary).

#### 3.2.2 Final genome assembly size, heterozygosity, and contiguity

**FIGURE 4:**
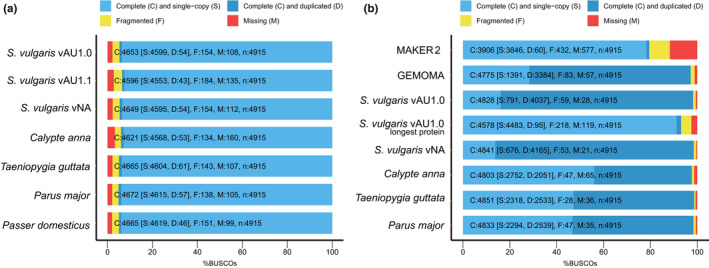
Assembly and annotation of *Sturnus vulgaris* in comparison to other avian reference assemblies and annotations. (a) busco (Aves) assessments of assembly completeness of *S. vulgaris* vAU1.0, and the NCBI uploaded genome *S. vulgaris* vAU1.1, presented alongside *S. vulgaris* vNA and four recent high‐quality avian reference genomes (*Taeniopygia guttata* assembly accession GCF_008822105.2, *Passer domesticus* assembly accession GCA_001700915.1, *Calypte anna* assembly accession GCA_003957555.2, *Parus major* assembly accession GCA_001522545.3). (b) busco (Aves) assessments of initial maker2 and gemoma assemblies, the merged *S. vulgaris* vAU1.0 annotation, the merged annotation with the longest protein‐per‐gene extracted using saaga, the final *S. vulgaris* vNA annotation (combined gemoma and maker2 annotation), and the ensemble annotations of three additional avian genomes

The *S. vulgaris* vAU assembly of 1,049,838,585 bp covers approximately 93.78% of the total estimated 1.119 Gb genome size (Appendix S3 Validation of supernova genome size prediction using jellyfish). We report a similar estimation of genome completeness by k‐mer analysis toolkit (kat), with the raw read1s (forward reads) estimating a genome completeness of 96.7% (estimated genome size 1.125 Gb, estimated heterozygosity rate 0.57%) and read2s (reverse reads) estimating a genome completeness of 95.92% (estimated genome size 1.135 Gb, estimated heterozygosity rate 0.54%) (Figure S7). Predicted genome sizes based on either read1s or read2s using kat were slightly larger than the estimation generated by jellyfish using all the read data; however, the length range was relatively consistent (1.119–1.135 Gb). busco completeness is comparable to other high‐quality passerine genomes (Figure 4a). *S. vulgaris* vAU has a scaffold N50 of 72.5 Mb and L50 of 5, with a total of 1628 scaffolds (Table 2); 98.6% (1,035,260,756 bp) of the sequence length has been assigned to the 32 super‐scaffolds, which serve as putative nuclear chromosomes (identified via the T. guttata version 3.2.4 assembly). The final assembly contains 14 putative macrochromosomes (>20 Mb, as described in Backström et al., 2010), with relative sizes appearing in consensus with known karyotype of *S. vulgaris* (Calafati & Capanna, 1981). These largest scaffolds account for 81.9% of the total assembly size, with the remainder on putative microchromosomes (16.9%) or unplaced scaffolds. While these large scaffolds are only proposed chromosomes assuming karyotype orthology, we found an increase in assembly quality scores post synteny‐based alignment across almost all assembly assessment metrics, including (protein‐coding) functional completeness and quality (Figure 3, Supporting Materials Table S4, Figure S6).

**TABLE 2:** *Sturnus vulgaris* overview of assembly statistics for vAU1.0, vAU1.1, and vNA, assessed using buscomp

	*Sturnus vulgaris* vAU1.0	*Sturnus vulgaris* vAU1.1	*Sturnus vulgaris* vNA
Total length (bp)	1,049,838,585	1,043,825,671	1,036,755,994
Number of scaffolds	1628	1344	2361
Scaffold N50 (bp)	72,525,610	72,244,370	3,416,708
Scaffold L50	5	5	89
Largest scaffold (bp)	151,927,750	151,503,485	11,828,398
Mean scaffold length (bp)	644,864.0	776,656.01	439,117.3
Median scaffold length (bp)	1337	1343	4856
Number of contigs	23,815	23,340	22,666
Contig N50 (bp)	145,864	147,322	147,183
Contig L50	2030	2010	1908
Gap (N) length (bp)	13,242,113 (1.26%)	7(0.74%)	23,939,528 (2.31%)
GC (guanine‐cytosine) content (%)	41.73%	41.72%	41.49%

### 3.3 *Sturnus vulgaris* genome annotation

The initial annotation produced by gemoma, informed by the 26 avian genome annotations available at the time on Ensembl (Table S1), predicted 21,539 protein coding genes, with 97.2% busco completeness (93.1% complete when longest protein‐per‐gene extracted with saaga) (Figure 4b). The initial maker2 annotation reported 13,495 genes, and a busco completeness of 79.5% (Figure 4b, Figure S8). The merged final annotation reported a busco completeness of 98.2% (Figure 4a), and this annotation predicted a total of 21,863 protein‐coding genes and 79,359 mRNAs (Table S5). The ratio in predicted maker2 and gemoma was more biased towards the homology‐based predictor, with an approximate ratio of 1:5 between maker2 and gemoma (Figure S8). Merging of the maker2 annotation to the gemoma annotation resulted in an increase in 1.1% in busco completeness. Duplication levels were much higher in the gemoma annotation when compared to maker2 (Figure 4b). This is not surprising, as the gemoma annotation will be biased towards well‐characterized genes and so may contain more transcripts per gene (Figure S8), whereas maker2 will inform the prediction of more taxon or possibly species‐specific coding sequences.

**FIGURE 5:**
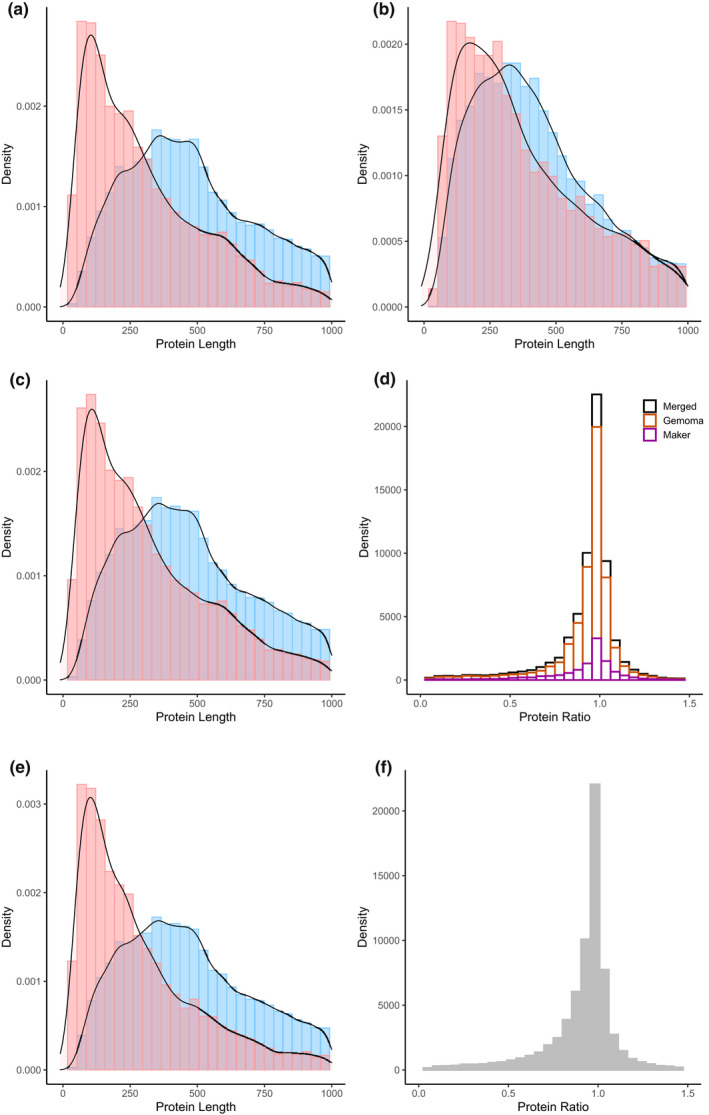
Summary of predicted annotated proteins. (a) Protein lengths for known proteins (blue, with a located Swiss‐Prot comparison) and unknown proteins (red, those that did not map to Swiss‐Prot) for the gemoma annotation compared to Swiss‐Prot. (b) Protein lengths of known and unknown proteins for the maker2 annotation compared to Swiss‐Prot. (c) Protein lengths of known and unknown proteins for the merged gemoma and maker2 annotation compared to Swiss‐Prot. (d) Protein length ratio between output from saaga for all known Swiss‐Prot proteins (where a score close to 1 indicates a high‐quality gene annotation, protein length ratio calculated as annotated protein length/best Swiss‐Prot reference protein length) (merged annotation, black; gemoma annotation, orange; maker2 annotation, purple). (e) Protein lengths of known and unknown proteins for the merged gemoma and maker2 annotation compared to *Gallus gallus* reference proteome (UP000000539_9031). (f) Protein length ratio between output from saaga for the merged annotation against the *Gallus gallus* reference proteome

The predicted transcripts were mapped using saaga to the Swiss‐Prot database, with 66,890 transcripts returning successful hits (84.3%) and 12,469 transcripts remaining unknown (15.7%) for the final annotation (Figure 5a). The known proteins had an average length of 652 amino acids (aa) and the unknown proteins had an average length of 426 aa (Figure 5a). Most of the predicted proteins were of high quality, with around 56% of them having an F1 score (see Methods) of greater than 0.95 (Figure 5b). Similar results were seen when the *Gallus gallus* reference proteome was used, with 69,714 known proteins of average length of 646 aa, 9645 known proteins of average length of 401 aa, and the final merged annotation having the same F1 score distribution, with an average F1 score of 89.8 (Figure 5c,d).

The gemoma annotation had similar protein quality patterns, with 57,026 known proteins (average length 664 aa), and 10,400 unknown proteins (average length 401 aa) (Figure 5e). The maker2 displayed much greater similarity in protein length histogram between known and unknown proteins, with shorter proteins with known homologues (average length 565 aa), but longer unknown proteins (average length 549 aa) (Figure 5f). The *S. vulgaris* vNA annotation performed similarly to the final *S. vulgaris* vAU annotation, with an average known protein length of 650 aa, and an average unknown protein length of 407 aa (Figure S9), and a Fl score of 88.7 when the *Gallus gallus* reference proteome was used.

### 3.4 *Sturnus vulgaris* genome‐wide patterns of genomics features

**FIGURE 6:**
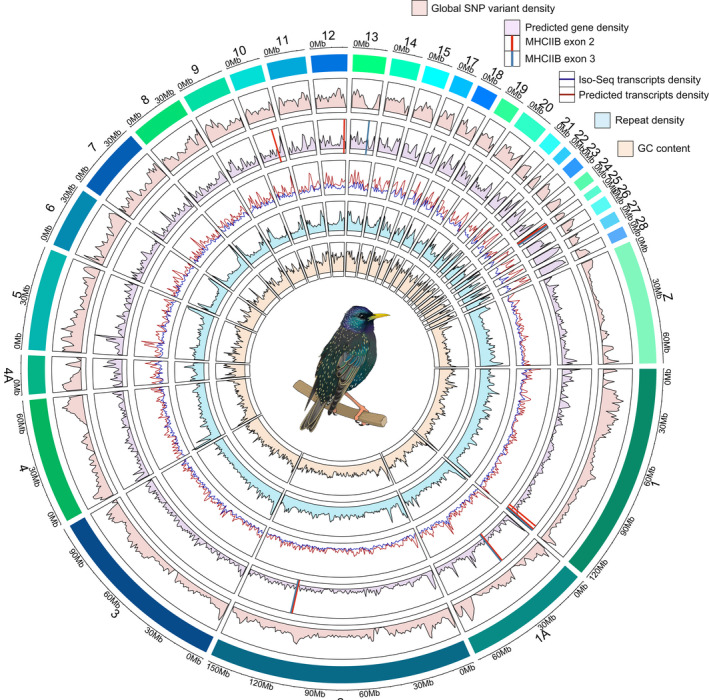
CIRCLIZE plot of the 32 main super‐scaffolds (32 putative autosome chromosomes) in the *Sturnus vulgaris* (*S. vulgaris* vAU) genome assembly (>98% of the total assembly length). The tracks denote variable values in 1,000,000 bp sliding windows. From the outermost track in, the variables displayed are track 1 (variant density, red area), track 2 (final annotation gene density, purple area; MHCIIB exon 2, red line; MHCIIB exon 3, blue line), track 3 (Iso‐Seq transcripts, blue line; final annotation transcripts, red line), and track 4 (repeat density, blue area), track 5 (GC content, yellow area). Of the 16 MHCIIB exon 2 and exon 3 hits on the final *S. vulgaris* vAU1.0, a total of 7 and 5 for exons 2 and 3 respectively were located on the main super‐scaffolds, with the remaining hits being located on unplaced scaffolds not graphed here

We plotted global whole genome variant data (Figure 6; track 1) and revealed genomic regions where variant density is low or nonexistent, indicative of high genetic conservation across the species, and genomic regions where variant density peaks are indicative of variant hotspots. We observed regions of high conservation corresponding to peaks in gene and/or transcript numbers (e.g., midway through chromosome 4), which may be indicative of regions of highly conserved genes and possibly centromere locations.

Final predicted gene densities (Figure 6; track 2) were largely following the patterns seen in transcript densities. We found that the transcript density compared between mapped Iso‐Seq reads and predicted transcripts in the final annotation displayed similar patterns, with some minor variation in patterns between the two (Figure 6; track 3). Patterns of transcript and gene numbers across the genome track relatively consistently to GC content (Figure 6; track 5). Of the copies of MHCIIB exons identified in the final assembly, seven out of 16 for exon 2, and six out of 16 for exon 3, where placed along the largest 32 super‐scaffolds, with four locations (putative chromosomes 1, 1A, 2, and 25) found to have an exon 2 and exon 3 sequence identified within a short genomic distance of one another (probably the same copy of the MCH gene) (Figure 6, track 2). Finally, repeat density varied across the super‐scaffolds, with often the super‐scaffold ends having highest repeat content (Figure 6, track 4).

#### 3.4.1 Synteny analysis

Through chromsyn analysis and visualization we identified that the synteny‐based assemblies resulting from *T. guttata, P. domesticus, and P. major* shared strong consensus of the local of identified busco genes on the super‐scaffolds, with *C. anna*, reporting the highest number of cross‐scaffold events (Figure S10). Across the four synteny‐based *S. vulgaris* vAU assemblies, we found many large blocks of linked genomic regions, with particularly high synteny evident across the micro‐ and Z chromosomes.

#### 3.4.2 TE composition and repeat content analysis across *S. vulgaris* vAU and vNA

**FIGURE 7:**
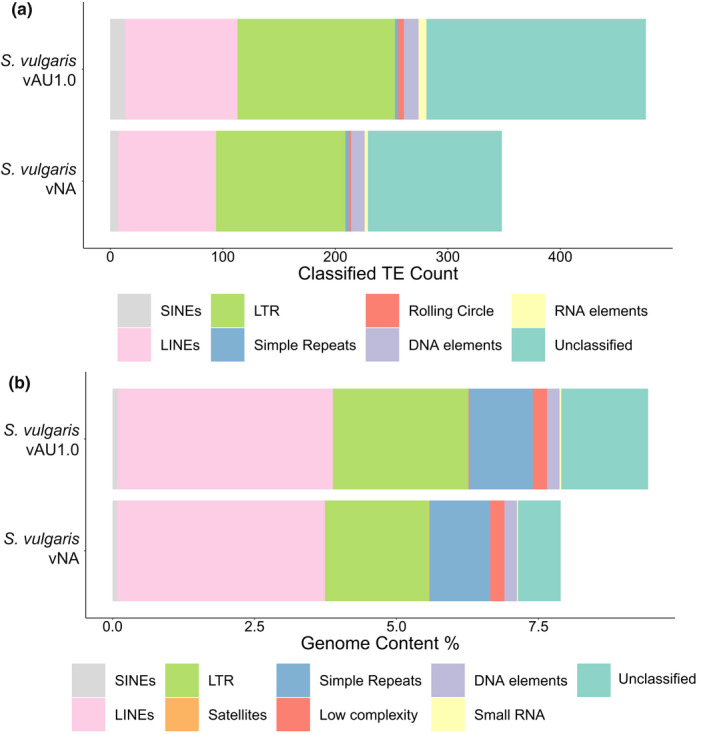
Transposable element and repeat element annotation of *S. vulgaris* vAU and vNA genome. (a) De novo transposable element annotation using repeatmodeler2, with the counts of each classified TE family based on consensus sequence output. (b) Repeat annotation using repeatmasker, using Aves repeat libraries and repeatmodeler2 output as the database

Through both de novo TE annotation and genome repeat elements analysis, we found a higher percentage of repeat genome coverage in *S. vulgaris* vAU than vNA, with the biggest difference attributed to LINEs and LTR for the TE annotation, and LTR and interspersed elements for the repeat element analysis (Figure 7a,b).

## 4 DISCUSSION

Here, we present a high‐quality, near‐complete reference genome for the European starling, *S. vulgaris* vAU as the primary reference for the species, with synteny‐based super‐scaffolding that assigns 98.6% of the genome assembly length to 32 putative nuclear chromosome scaffolds. We demonstrate the utility of a wide range of assembly assessment tools in the *S. vulgaris* vAU assembly process, including whole transcript mapping, two new assembly tools buscomp and saaga, along with a diverse range of other approaches. We also present a second genome assembly from a starling sampled in North America (*S. vulgaris* vNA) and discuss the genomic landscape of this globally significant species.

### 4.1 *Sturnus vulgaris* vAU transcriptome

When comparing the completeness of this new starling transcriptome data to existing Illumina short read transcript data produced using liver tissue (Richardson et al., 2017), we found an increase of about 20% in busco completeness, with a particularly large increasein the number of duplicated busco, a result of the alternate transcript isoforms captured through the Iso‐Seq. Assessing the effect the tama pipeline had on busco completeness, we found a small drop in complete buscos (Figure 2a) that appear to have been lost during the mapping to genome assembly step. Finally, comparing our final transcriptome to two other avian Iso‐Seq transcriptomes gives an indication of how much unique transcript information is added by the addition of tissues into pooled Iso‐Seq sequencing runs. The single tissue Iso‐Seq liver transcriptome of *Calypte anna* (Anna's hummingbird) (Workman et al., 2018) yielded similar busco completeness to the short read *S. vulgaris* liver transcriptome. The eight tissue Iso‐Seq transcriptome of *Anas platyrhynchos* (mallard) (Yin et al., 2019) yielded an increase of 30% in complete buscos, consistent with the expectation that our three‐tissue Iso‐Seq library will be missing a number of tissue‐specific genes.

### 4.2 Constructing an avian genome

During the construction of the primary genome assembly presented in this manuscript, *S. vulgaris* vAU, we found that scaffolding with the low coverage ONT long reads generally yielded the greatest improvement in assembly metrics. It has previously been shown that even low coverage of ONT data in conjunction with 10× may produce high‐quality genome assemblies (Ma et al., 2019). This was true for our data, which demonstrates the utility of even low coverage, long read sequencing (approximately 4.5% coverage based on the estimated genome size of 1.119 Gb) in greatly improving the contiguity of scaffolds generated by short read genome assemblers (though with steadily decreasing costs, Hi‐C data may serve this purpose at a lower cost to scaffold ratio and may assist in identifying misassemblies, which is often not a focus of long‐read scaffolding tools). While additional scaffolding using the Iso‐Seq whole transcripts did not result in a large increase in continuity, the Iso‐Seq reads served to minimize the number of fragmented genes in the final assembly, helping downstream analysis and gene prediction models. Synteny‐based scaffolding of the *S. vulgaris* genome against that of *T. guttata* produced super‐scaffolds, with putative chromosomal identities assigned and to 98.6% of the assembly length. In support of the assumed synteny of this step, we found varying increases in assembly quality and completeness in nearly all assembly assessment metrics, with alternate‐reference synteny‐based scaffolded assemblies concurring about general scaffolding placement within super‐scaffolds.

Broadly, the assessment tools agreed with one another about relative improvements across each subsequent assembly step, though each provided additional fine‐scale feedback on assembly improvements achieved by each assembly step. Across the eight assembly steps of the *S. vulgaris* vAU genome, applying buscomp helped to validate whether the improved or reduced busco scores at each assembly step were legitimate (e.g., between busco reported a drop in complete sequences post whole transcript scaffolding, which buscomp suggests is a result of sequence independent assembly changes). We found buscomp, Iso‐seq mapping and MHCIIB gene annotation followed similar trends (Figure 3), though the latter was more sensitive to improvements post genome polishing, probably due to the length and stringency of the sequences being utilized. These results suggest that buscomp, together with the mapped Iso‐Seq reads, can deal with the unpolished intermediary genome steps, and does not suffer the same sequence identification accuracy issues as the traditional stand‐alone busco analysis. Interestingly, polishing was the only step that resulted in a decrease in saaga metrics, which though negligible, could have been a result of reduced protein sequences matches to the reference database. Repeat annotation yielded the most different results relative to the other metrics used (Figure S6a), reporting no improvements during scaffolding steps, only during gap‐filling. Lastly, assembly duplication analysis using kat agreed with busco results (Table S4), indicating there was little final assembly sequence duplication when comparing to raw read k‐mer counts. These analyses highlight the benefits of these complementary assessment approaches in ensuring that aspects of genome quality are not sacrificed to improve nonspecific assembly quality metrics, such as N50, and provide broader perspective on improvements to a range of aspects of the genome.

Of the different assessment methods used, the mapping of the high‐quality Iso‐Seq reads proved to be the fastest method of assessment (33,454 Iso‐seq sequences mapped in <5 mins with 16 CPU cores), while the gemoma and saaga took the most amount of compute time at 12 h per assembly was roughly comparable to busco (approximately 50 CPU h per assembly on an average machine), though more computationally intensive (gemoma ran for approximately 200 CPU h per assembly, and saaga ran for approximately 8 CPU h per assembly).

For the final *S. vulgaris* vAU annotation, the predicted proteins of unknown origin (those that failed to map to Swiss‐Prot database or *Gallus gallus* proteome) had a smaller average length than those with known homologues (Figure 5a,c). Similar results were found when this approach was used to assess genes predicted in the *R. marina* genome assembly (Edwards et al., 2018), and are indicative that these “unknown” proteins are fragmented and lower quality predictions that may be due to underlying assembly issues with contiguity or frameshifting indels. The known proteins predicted by maker2 (Figure 5f) were of apparent lower quality than those reported by gemoma as indicated by their shorter lengths and lower protein ratios (Figure 5e), which may be a result from a combination of incorrect gene predictions, and the high‐quality reference homologues inflating quality scores of the gemoma annotation in comparison. Predicted genes were more commonly shorter than their closest reference protein hits, indicative there might still be some truncated gene predictions, consistent with the large number of assembly gaps. Nevertheless, the final annotation has a strong protein ratio peak around 1.0 for known proteins (Figure 5b,d), indicating that the bulk of these predicted genes were of lengths similar to their Swiss‐Prot homologues and hence deemed high quality.

### 4.3 Sturnus vulgaris vAU and Sturnus vulgaris vNA

In this manuscript, we present a second genome assembly of a sampled collected in North America (*S. vulgaris* vNA; GCF_001447265.1) alongside the primary *S. vulgaris* vAU assembly. There is increasing recognition of the importance of pan‐genomes (genome assemblies that differentiate between genes/regions shared by all members of the species, and dispensable or rare genes/regions) (Hirsch et al., 2014; Sherman & Salzberg, 2020), which are essential for many model organisms (Vernikos et al., 2015). Having these two high‐quality assemblies from different populations will improve future genomic work on the global invasive populations of this species, and facilitate review of structural variation (e.g., inversions) that may exist across different populations. It should be noted, however, that the final scaffolding step for *S. vulgaris* vAU assumed structural conservation between the starling and zebra finch and thus future synteny analyses may want to use the earlier assembly step with additional scaffolding data. Further, as these two genomes were constructed using different types of raw data and thus different assembly pipelines, we cannot directly attribute differences between the genomes to their lineages. Hence we discuss the main differences below to provide perspective for future studies seeking to interpret data generated using these genomic resources.

Overall, the *S. vulgaris* vAU assembly improved genome assembly statistics over the *S. vulgaris* vNA genome, with a greater percentage of the estimated 1.119 Gb genome represented (94% vs. 93%), an increase of scaffold N50 from 3.42 to 72.5 Mb, a decrease in scaffold L50 from 89 to 5. The *S. vulgaris* vNA nevertheless has good assembly statistics including a roughly similar contig N50 (147,183 vs. 145,864 in vAU) (Table 2, Table S4) and performed slightly better under a few coding‐region related assessments (buscomp, saaga using Swiss‐Prot reference). This was probably due to a higher average base call accuracy and/or collapsed repetitive regions, making reads easier to uniquely map to the assembly, as would be indicated by the higher saaga F1 scores for vAU when the reference proteome was restricted to just a single avian reference *Gallus gallus*. In contrast to this result, *S. vulgaris* vNA performed more poorly during assessments of harder to assemble regions of the genome (MHCIIB gene exons and repeat content) (Figure 3, Figure 7), again probably due to collapsed repeat regions and smaller genome size. Taken together, this suggests that *S. vulgaris* vNA may have slightly better base accuracy for conserved gene models, but *S. vulgaris* vAU should be used as a reference for studies seeking to characterize more complex genomic elements during resequencing studies.

Near identical annotation pipelines were used for the two genome assemblies, and yielded similar final annotation statistics, but with the *S. vulgaris* vNA assembly resulting in slightly more predicted genes (Table S5) and having a larger predicted gene coverage over the genome (59.09% gene coverage vs. 55.23%). This indicates that this increase in predicted genes is not just a result of more overlapped predictions, though it could be a result of smaller assembly size and higher gene duplication (Figure 4a). The known protein lengths were similar across the *S. vulgaris* vAU and vNA annotations (652 vs. 650 aa), though there was a slightly larger difference in average unknown protein length (426 vs. 407 aa). Although this increase in *S. vulgaris* vAU is very slight, it may indicate increased quality of unknown protein predictions in the vAU annotation, possibly due to more Iso‐Seq data mapping to the vAU genome (Figure 3b) or the higher assembly contiguity.

### 4.4 Structure and function of the *Sturnus vulgaris* genome

Analysis of the *S. vulgaris* assemblies provide insights into the genomic landscape of this species. We identified high congruence between Iso‐Seq and predicted transcript numbers across the *S. vulgaris* vAU genome (Figure 6). In a few regions, we observe dissimilar (usually lower) Iso‐Seq transcript density compared to predicted transcript density. We interpret this as either genomic regions producing tissue‐specific transcripts not captured by the brain, testes, or muscle tissue analyses, or possibly annotated transcript overprediction.

Comparing the annotated TE and repeat content landscape of *S. vulgaris* vAU and *S. vulgaris* vNA revealed a high proportion of LTRs and LINEs (Figure 7b), with highest diversity in sequence diversity in LTRs (Figure 7b). A major component of many avian genome is LTRs, with specifically CR1 (chicken repeat 1) contributing to this abundance (Mason et al., 2016). In *S. vulgaris* we found LTR coverage of approximately 2.5%, which is moderate compared to other avian species but on the lower end of LTR content for passerines (Boman et al., 2019; Gao et al., 2017).

The MCHIIB region in avian genomes reside on chromosome 16, a notoriously difficult avian chromosome to assemble to its high GC and repeat content, which often remains fragmented within assemblies (including ours) (Miller & Taylor, 2016). The MHCIIB annotation we conducted identified more exon 2 sequences than exon 3 sequences for most assembly steps, concurring with previous such analysis of the *Lycocorax pyrrhopterus obiensis* (Peona et al., 2021). Further, this previous study identified a similar spread of MHCIIB sequences across super‐scaffolds verses unplaced scaffold (possibly the segments of chromosome 16), particularly for avian genome assemblies that did not incorporate proximity ligation data to assist resolving genome wide structure (Peona et al., 2021). It is promising that of the exon sequences that were placed on the *S. vulgaris* vAU super‐scaffolds, roughly half of these again found an exon 2 and exon 3 occurring in close proximity to one another, indicating a probably legitimate MHCIIB sequence (although sequence placement itself does not guarantee it is not a misassembled region).

Synteny analysis supported assumptions of synteny across the microchromosomes of the reference avian species and subsequent synteny‐based assemblies, aligning with previous notions regarding the highly conserved nature of these avian genomic sequences (Waters et al., 2021). Across the four synteny‐based assemblies we identified large regions of conserved synteny across the macrochromosomes, though we do observe some gene position shifts across super‐scaffolds that will require further exploration with additional long read, proximity ligation or optical mapping data.

## 5 CONCLUSION

In this manuscript, we present two high‐quality genomes, a primary assembly *S. vulgaris* vAU, and a second assembly, *S. vulgaris* vNA. These genomes, coupled with species‐specific whole transcript reads, provide vital resources for characterizing the diverse and changing genomic landscape of this globally important avian. In addition to improving the completeness of gene annotation, we demonstrate the utility of long‐read transcript data for genome quality assessment and assembly scaffolding. We also present the complementary assembly assessment tools saaga and buscomp, which can identify and resolve potential artefacts, inform assembly pipeline decisions, and highlight the importance of diverse assessment tools in the assembly and assessment of reference genomes and annotations.

## AUTHOR CONTRIBUTIONS

Project conception: all authors. Sample collection: Katarina C. Stuart, Scott J. Werner, Matthew C. Brandley. Laboratory work: Katarina C. Stuart, Yuanyuan Cheng, Lee A. Rollins, Wesley C. Warren. Data Analysis: Katarina C. Stuart, Richard J. Edwards, Yuanyuan Cheng, Wesley C. Warren. Program development: Richard J. Edwards. Manuscript writing: Katarina C. Stuart, Richard J. Edwards. Manuscript editing: All authors.

## CONFLICT OF INTEREST

The authors declare no conflict of interest.

### OPEN RESEARCH BADGES

This article has earned an Open Data badge for making publicly available the digitally‐shareable data necessary to reproduce the reported results. The data is available at [[insert provided URL from Open Research Disclosure Form]].

## Supporting information


Appendix S1

Appendix S2

## Data Availability

buscomp documentation: https://slimsuite.github.io/buscomp/; diploidocus documentation: https://slimsuite.github.io/diploidocus/; saaga documentation: https://slimsuite.github.io/saaga/; The data have been deposited with links to BioProject accession no. PRJNA706841 in the NCBI BioProject database (https://www.ncbi.nlm.nih.gov/bioproject/). The two *S. vulgaris* assembly files are available via NCBI (*S. vulgaris* vAU1.1 at GCA_023376015.1, *S. vulgaris* vNA at GCF_001447265.1). Supporting Information Files 1 and 2 are direct BUSCOMP program outputs and are available as HTML files on Dryad, along with key project code and output files (doi:10.5061/dryad.02v6wwq5z). Any scripts or data not covered by the above can either be retrieved from GitHub (https://github.com/katarinastuart/Sv3_StarlingGenome) or directly from the corresponding author.
