# Supplementary material for: Transcript‐ and annotation‐guided genome assembly of the European starling
Source: Mol Ecol Resour. 2022 Jul 18;22(8):3141–60. doi: 10.1111/1755-0998.13679 (PMC9796300; doi:10.1111/1755-0998.13679)
Supplement: Supplementary file 1 — Appendix S1 [file MEN-22-3141-s001.zip › MEN_13679_Sv3.10_Supplementary_File_1_starling10xV3.N3L20ID0U.full_compressed.pdf]

1 BUSCOMP Run Summary
2 Genome Summary
3 BUSCO Ratings
4 BUSCOMP Ratings
5 BUSCO and BUSCOMP Comparisons
6 Appendix: BUSCOMP run details

BUSCOMP Full Report

starling10xV3 BUSCOMP Analysis

2021-03-04

1 BUSCOMP Run Summary

```
BUSCOMP V0.11.0: run Thu Mar  4 13:38:02 2021
```

See the **run details appendix** end of this document for details of the **log file (/scratch/tmp/z3452659/projects/BUSCOMP-Jan19/analysis/2021-03-03.10xStarling/buscompV3/starling10xV3.log)**, **commandline parameters** and runtime **BUSCOMP errors and warnings**.

NOTE: To edit this document, open `starling10xV3.N3L20ID0U.full.Rmd` in RStudio, edit and re-knit to HTML.

1.1 BUSCOMP Results Summary

Assemblies can be assessed on a number of criteria, but the main ones (in the absence of a reference "truth" genome) are either to judge contiguity or completeness. NG50 and LG50 values are based on a genome size of 1.1 Gb. If the `genomesize=X` parameter was not set (see command list in **appendix**), this will be based on the longest assembly (see sequence stats, below).

Of the 24 assemblies analysed (24 BUSCO; 24 fasta; 24 both), 23 genomes were rated as the "best" by at least one criterion:

- `starling10x.revcomp` : MaxLength.
- `starling10x.pribusco` : MaxLength, NoBUSCO.
- `starling10x.dipnr` : MaxLength, Complete, Missing.
- `starling10x.pri` : MaxLength, Complete, Missing.
- `starling10x.shuffle3` : MaxLength.
- `starling10x.triplicate` : MaxLength, BUSCO.
- `starling10x.3n` : MaxLength.
- `starling10x.duplicate` : MaxLength.
- `starling10x.4n` : NG50Length, LG50Count, MaxLength.
- `starling10x.rep6` : MaxLength, NoBUSCO.
- `starling10x.rep7` : MaxLength, NoBUSCO.
- `starling10x.rep4` : MaxLength, NoBUSCO.
- `starling10x.rep5` : MaxLength, NoBUSCO.
- `starling10x.rep2` : MaxLength, NoBUSCO.
- `starling10x.rep3` : MaxLength, NoBUSCO.
- `starling10x.rep0` : MaxLength, NoBUSCO.
- `starling10x.rep1` : MaxLength, NoBUSCO.
- `starling10x.rep8` : MaxLength, NoBUSCO.
- `starling10x.rep9` : MaxLength, NoBUSCO.
- `starling10x.shuffle1` : MaxLength.
- `starling10x.shuffle2` : MaxLength.
- `starling10x.2n` : MaxLength.
- `starling10x.copy` : MaxLength, NoBUSCO.

Best assemblies by assembly contiguity critera:

- **NG50Length.** Longest NG50 contig/scaffold length (6,889,770 bp): `starling10x.4n`
- **LG50Count.** Smallest LG50 contig/scaffold count (67): `starling10x.4n`
- **MaxLength.** Maximum contig/scaffold length (12,884,419 bp): `starling10x.2n` , `starling10x.3n` , `starling10x.4n` , `starling10x.copy` , `starling10x.dipnr` , `starling10x.duplicate` , `starling10x.pri` , `starling10x.pribusco` , `starling10x.rep0` , `starling10x.rep1` , `starling10x.rep2` , `starling10x.rep3` , `starling10x.rep4` , `starling10x.rep5` , `starling10x.rep6` , `starling10x.rep7` , `starling10x.rep8` , `starling10x.rep9` , `starling10x.revcomp` , `starling10x.shuffle1` , `starling10x.shuffle2` , `starling10x.shuffle3` , `starling10x.triplicate`

Best assemblies by completeness critera:

- **Complete.** Most Complete (Single & Duplicated) BUSCOMP sequences (99.9 %): `starling10x.dipnr` , `starling10x.pri`
- **Missing.** Fewest Missing BUSCOMP sequences (0.0 %): `starling10x.dipnr` , `starling10x.pri`
- **BUSCO.** Most Complete (Single & Duplicated) BUSCO sequences (94.0 %): `starling10x.triplicate`
- **NoBUSCO.** Fewest Missing BUSCO sequences (2.3 %): `starling10x.copy` , `starling10x.pribusco` , `starling10x.rep0` , `starling10x.rep1` , `starling10x.rep2` , `starling10x.rep3` , `starling10x.rep4` , `starling10x.rep5` , `starling10x.rep6` , `starling10x.rep7` , `starling10x.rep8` , `starling10x.rep9`

2 Genome Summary

The following genomes and BUSCO results were analysed by BUSCOMP:

- **starling10x.dipnr**. [`BUSCO` | `Fasta`] Pseudodiploid assembly
- **starling10x.pri**. [`BUSCO` | `Fasta`] Primary scaffolds
- **starling10x.alt**. [`BUSCO` | `Fasta`] Alternative scaffolds
- **starling10x.pribusco**. [`BUSCO` | `Fasta`] Primary BUSCO-containing scaffolds
- **starling10x.revcomp**. [`BUSCO` | `Fasta`] Reverse complemented BUSCO scaffolds
- **starling10x.copy**. [`BUSCO` | `Fasta`] Direct copy of primary BUSCO scaffolds
- **starling10x.duplicate**. [`BUSCO` | `Fasta`] Primary BUSCOs plus reverse complement
- **starling10x.triplicate**. [`BUSCO` | `Fasta`] Two primary BUSCO copies plus reverse complement
- **starling10x.shuffle1**. [`BUSCO` | `Fasta`] Randomly shuffled primary BUSCO scaffolds (1)
- **starling10x.shuffle2**. [`BUSCO` | `Fasta`] Randomly shuffled primary BUSCO scaffolds (2)
- **starling10x.shuffle3**. [`BUSCO` | `Fasta`] Randomly shuffled primary BUSCO scaffolds (3)
- **starling10x.2n**. [`BUSCO` | `Fasta`] Primary BUSCO scaffolds plus one shuffled copy
- **starling10x.3n**. [`BUSCO` | `Fasta`] Primary BUSCO scaffolds plus two shuffled copies
- **starling10x.4n**. [`BUSCO` | `Fasta`] Primary BUSCO scaffolds plus three shuffled copies
- **starling10x.rep0**. [`BUSCO` | `Fasta`] Primary BUSCO replication 0
- **starling10x.rep1**. [`BUSCO` | `Fasta`] Primary BUSCO replication 1
- **starling10x.rep2**. [`BUSCO` | `Fasta`] Primary BUSCO replication 2
- **starling10x.rep3**. [`BUSCO` | `Fasta`] Primary BUSCO replication 3
- **starling10x.rep4**. [`BUSCO` | `Fasta`] Primary BUSCO replication 4
- **starling10x.rep5**. [`BUSCO` | `Fasta`] Primary BUSCO replication 5
- **starling10x.rep6**. [`BUSCO` | `Fasta`] Primary BUSCO replication 6
- **starling10x.rep7**. [`BUSCO` | `Fasta`] Primary BUSCO replication 7
- **starling10x.rep8**. [`BUSCO` | `Fasta`] Primary BUSCO replication 8
- **starling10x.rep9**. [`BUSCO` | `Fasta`] Primary BUSCO replication 9

Details of the directories and files are below:

	Directory <chr>
1	../busco3/run_00_starling10x.dipnr
2	../busco3/run_01_starling10x.pri
3	../busco3/run_02_starling10x.alt
5	../busco3/run_04_starling10x.pribusco
6	../busco3/run_05_starling10x.revcomp
8	../busco3/run_11_starling10x.copy
9	../busco3/run_12_starling10x.duplicate
10	../busco3/run_13_starling10x.triplicate
12	../busco3/run_21_starling10x.shuffle1
13	../busco3/run_22_starling10x.shuffle2

1-10 of 24 rows | 1-2 of 6 columns **Previous 1 2 3 Next**

Genomes with a **Directory** listed had BUSCO results available. If **Sequences** is `True`, these would be have been compiled to generate the BUSCOMP sequence set (unless `buscompseq=F`, or alternative sequences were provided with `buscofas=FASFILE`). Genomes with a **Fasta** listed had sequence data available for BUSCOMP searches.

2.1 Genome statistics

The following genome statistics were also calculated by `RJE_SeqList` for each genome (table, below):

- **SeqNum**: The total number of scaffolds/contigs in the assembly.
- **TotLength**: The total combined length of scaffolds/contigs in the assembly.
- **MinLength**: The length of the shortest scaffold/contig in the assembly.
- **MaxLength**: The length of the longest scaffold/contig in the assembly.
- **MeanLength**: The mean length of scaffolds/contigs in the assembly.
- **MedLength**: The median length of scaffolds/contigs in the assembly.
- **N50Length**: At least half of the assembly is contained on scaffolds/contigs of this length or greater.
- **L50Count**: The smallest number scaffolds/contigs needed to cover half the the assembly.
- **CtgNum**: Number of contigs (`SeqNum` + `GapCount`).
- **N50Ctg**: At least half of the assembly is contained on contigs of this length or greater.
- **L50Ctg**: The smallest number contigs needed to cover half the the assembly.
- **NG50Length**: At least half of the genome is contained on scaffolds/contigs of this length or greater. This is based on `genomesize=X`. If no genome size is given, it will be relative to the biggest assembly.
- **LG50Count**: The smallest number scaffolds/contigs needed to cover half the the genome. This is based on `genomesize=X`. If no genome size is given, it will be relative to the biggest assembly.
- **GapLength**: The total number of undefined "gap" (`N`) nucleotides in the assembly.
- **GapCount**: The total number of undefined "gap" (`N`) regions in the assembly.
- **GC**: The %GC content of the assembly.

Genome	Description	SeqNum	TotLength	MinLength	MaxLength	MeanLength	MedLength	N50Length	L50Count	CtgNum	N50Ctg
starling10x.dipnr	Pseudodiploid assembly	19346	1887443050	1000	12884419	97562.44	2307.5	1907593	245	49415	142122

Genome	Description	SeqNum	TotLength	MinLength	MaxLength	MeanLength	MedLength	N50Length	L50Count	CtgNum	N50Ctg
starling10x.pri	Primary scaffolds	18439	1040106492	1000	12884419	56407.97	2153.0	1764435	146	37354	132973
starling10x.alt	Alternative scaffolds	907	847336558	1620	12880535	934218.92	450476.0	2203301	102	12061	152010
starling10x.pribusco	Primary BUSCO-containing scaffolds	968	876348215	1833	12884419	905318.40	409882.5	2277667	104	11579	154535
starling10x.revcomp	Reverse complemented BUSCO scaffolds	968	876348215	1833	12884419	905318.40	409882.5	2277667	104	11579	154535
starling10x.copy	Direct copy of primary BUSCO scaffolds	968	876348215	1833	12884419	905318.40	409882.5	2277667	104	11579	154535
starling10x.duplicate	Primary BUSCOs plus reverse complement	1936	1752696430	1833	12884419	905318.40	409882.5	2277667	208	23158	154535
starling10x.triplicate	Two primary BUSCO copies plus reverse complement	2904	2629044645	1833	12884419	905318.40	409882.5	2277667	312	34737	154535
starling10x.shuffle1	Randomly shuffled primary BUSCO scaffolds (1)	968	876348215	1833	12884419	905318.40	409882.5	2277667	104	971	2277667
starling10x.shuffle2	Randomly shuffled primary BUSCO scaffolds (2)	968	876348215	1833	12884419	905318.40	409882.5	2277667	104	973	2277667
starling10x.shuffle3	Randomly shuffled primary BUSCO scaffolds (3)	968	876348215	1833	12884419	905318.40	409882.5	2277667	104	969	2277667
starling10x.2n	Primary BUSCO scaffolds plus one shuffled copy	1936	1752696430	1833	12884419	905318.40	409882.5	2277667	208	12550	436368
starling10x.3n	Primary BUSCO scaffolds plus two shuffled copies	2904	2629044645	1833	12884419	905318.40	409882.5	2277667	312	13523	1002636
starling10x.4n	Primary BUSCO scaffolds plus three shuffled copies	3872	3505392860	1833	12884419	905318.40	409882.5	2277667	416	14492	1397823
starling10x.rep0	Primary BUSCO replication 0	968	876348215	1833	12884419	905318.40	409882.5	2277667	104	11579	154535
starling10x.rep1	Primary BUSCO replication 1	968	876348215	1833	12884419	905318.40	409882.5	2277667	104	11579	154535
starling10x.rep2	Primary BUSCO replication 2	968	876348215	1833	12884419	905318.40	409882.5	2277667	104	11579	154535
starling10x.rep3	Primary BUSCO replication 3	968	876348215	1833	12884419	905318.40	409882.5	2277667	104	11579	154535

Genome	Description	SeqNum	TotLength	MinLength	MaxLength	MeanLength	MedLength	N50Length	L50Count	CtgNum	N50Ctg
starling10x.rep4	Primary BUSCO replication 4	968	876348215	1833	12884419	905318.40	409882.5	2277667	104	11579	154535
starling10x.rep5	Primary BUSCO replication 5	968	876348215	1833	12884419	905318.40	409882.5	2277667	104	11579	154535
starling10x.rep6	Primary BUSCO replication 6	968	876348215	1833	12884419	905318.40	409882.5	2277667	104	11579	154535
starling10x.rep7	Primary BUSCO replication 7	968	876348215	1833	12884419	905318.40	409882.5	2277667	104	11579	154535
starling10x.rep8	Primary BUSCO replication 8	968	876348215	1833	12884419	905318.40	409882.5	2277667	104	11579	154535
starling10x.rep9	Primary BUSCO replication 9	968	876348215	1833	12884419	905318.40	409882.5	2277667	104	11579	154535

NOTE: `NG50Length` and `LG50Count` statistics use `genomesize=X` or the biggest assembly loaded (1.10 Gb). If BUSCOMP has been run more than once on the same data (*e.g.* to update descriptions or sorting), please make sure that a consistent genome size is used, or these values may be wrong. If in doubt, run with `force=T` and force regeneration of statistics.

2.2 Genome coverage assessment plots

In general, a good assembly will be approx. the same size as the genome and in as few pieces as possible. Any assembly smaller than the predicted genome size is clearly missing coverage. Assemblies bigger than the genome size might still be missing chunks of the genome if redundancy/duplication is a problem. In the following plot, the grey line marks the given genome size of 1.1 Gb.

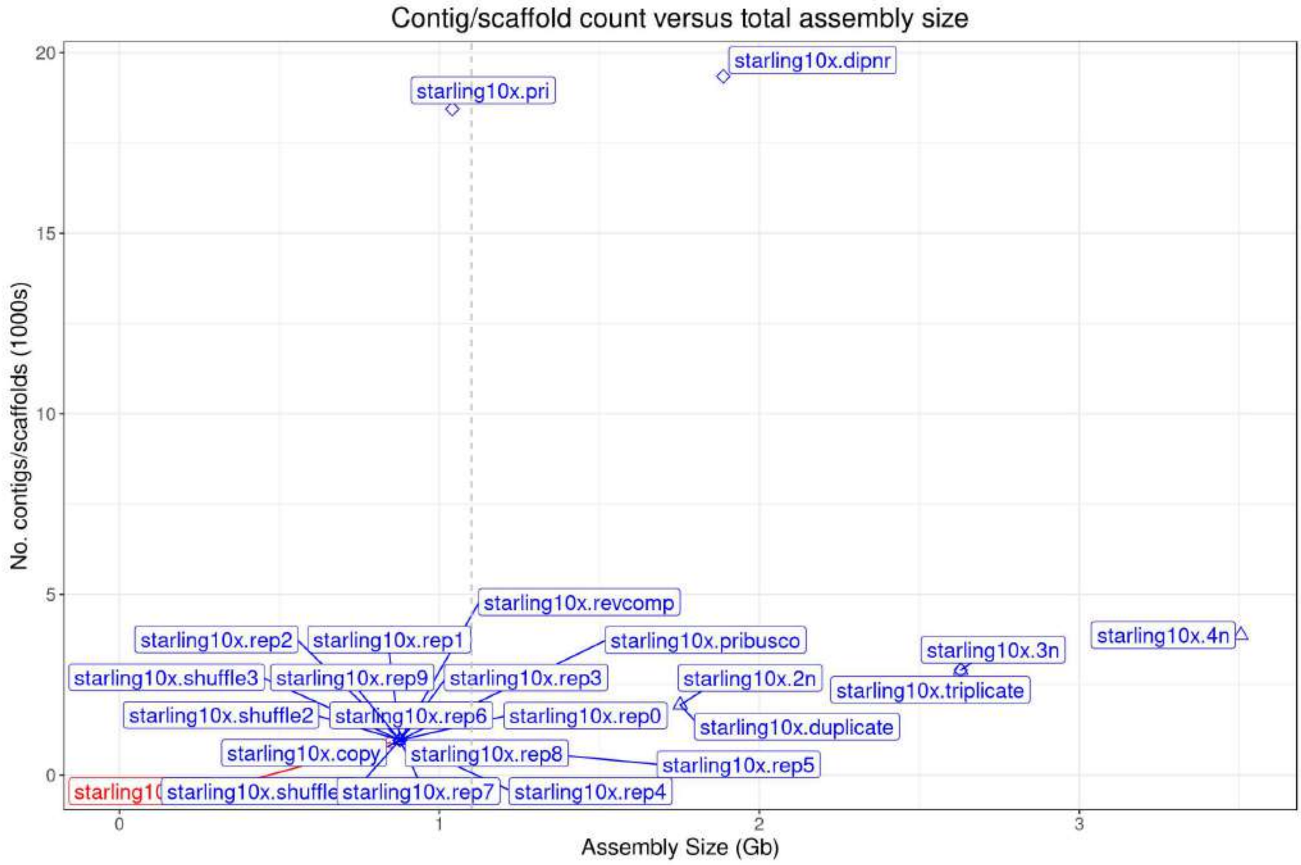

A better indicator of the overall coverage of the genome is the number of `Missing` BUSCO genes. As BUSCO is highly dependent on the accuracy of the sequence and the gene models it makes, the `Missing` BUSCOMP ratings arguably give a more consistent proxy for genome completeness. NOTE: this says nothing about the fragmentation or completeness of the genes themselves.

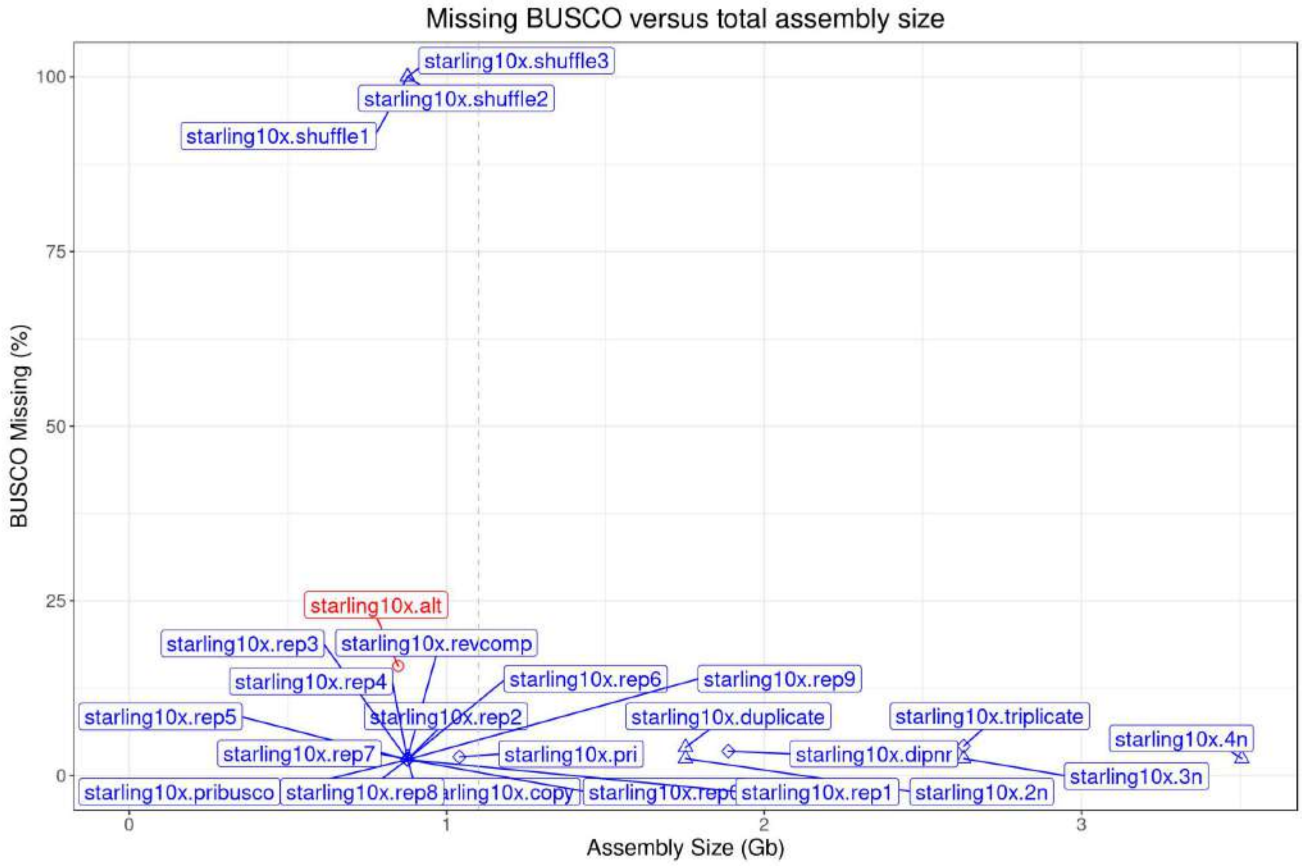
Missing BUSCO versus total assembly size
BUSCO Missing (%)
Assembly Size (Gb)
0
25
50
75
100
0
1
2
3
starling10x.shuffle3
starling10x.shuffle2
starling10x.shuffle1
starling10x.alt
starling10x.rep3
starling10x.revcomp
starling10x.rep4
starling10x.rep6
starling10x.rep9
starling10x.rep5
starling10x.rep2
starling10x.duplicate
starling10x.triplicate
starling10x.4n
starling10x.rep7
starling10x.pri
starling10x.dipnr
starling10x.3n
starling10x.pribusco
starling10x.rep8
starling10x.rep1
starling10x.2n

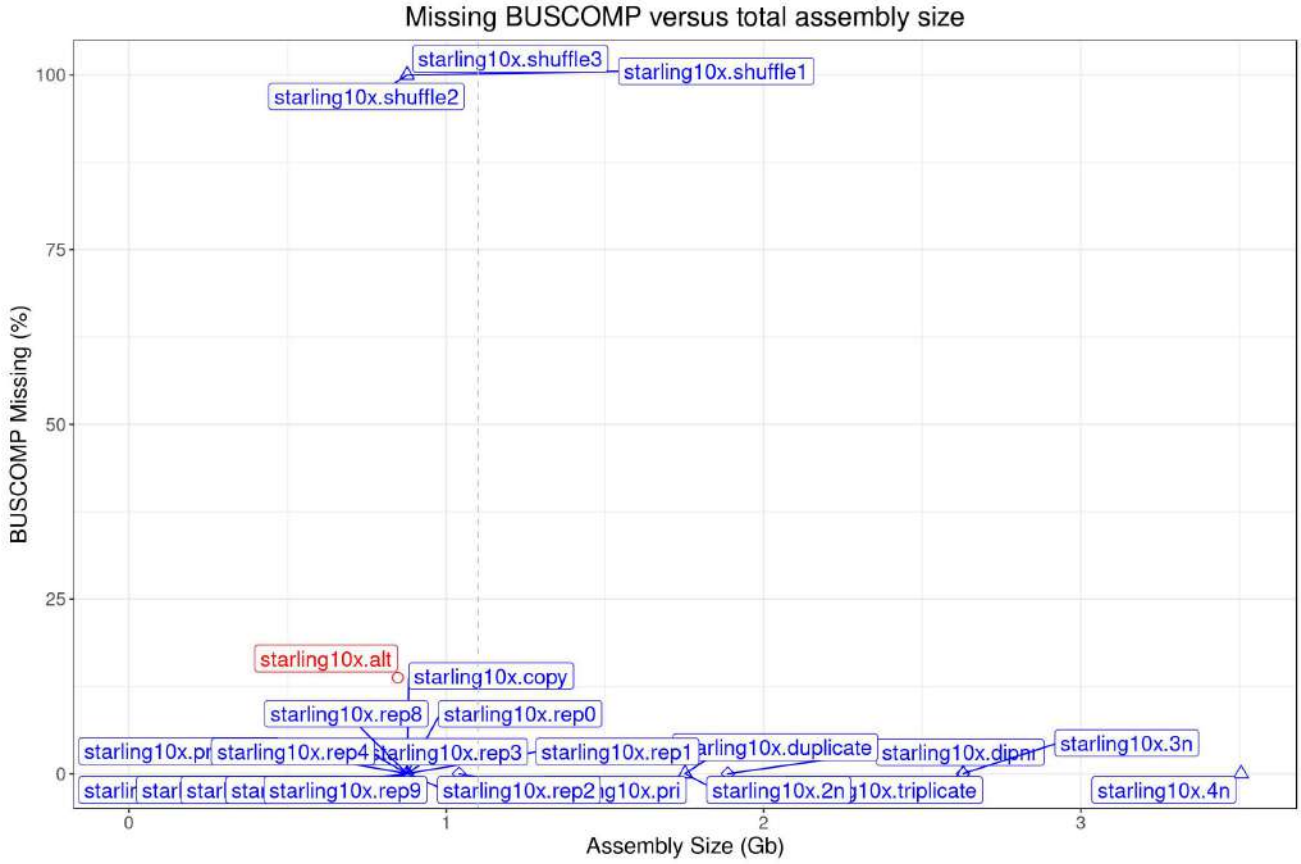
Missing BUSCOMP versus total assembly size
BUSCOMP Missing (%)
Assembly Size (Gb)
0
25
50
75
100
0
1
2
3
starling10x.shuffle3
starling10x.shuffle1
starling10x.shuffle2
starling10x.alt
starling10x.copy
starling10x.rep8
starling10x.rep0
starling10x.rep4
starling10x.rep3
starling10x.rep1
starling10x.dipnr
starling10x.3n
starling10x.rep9
starling10x.rep2
starling10x.2n
starling10x.4n

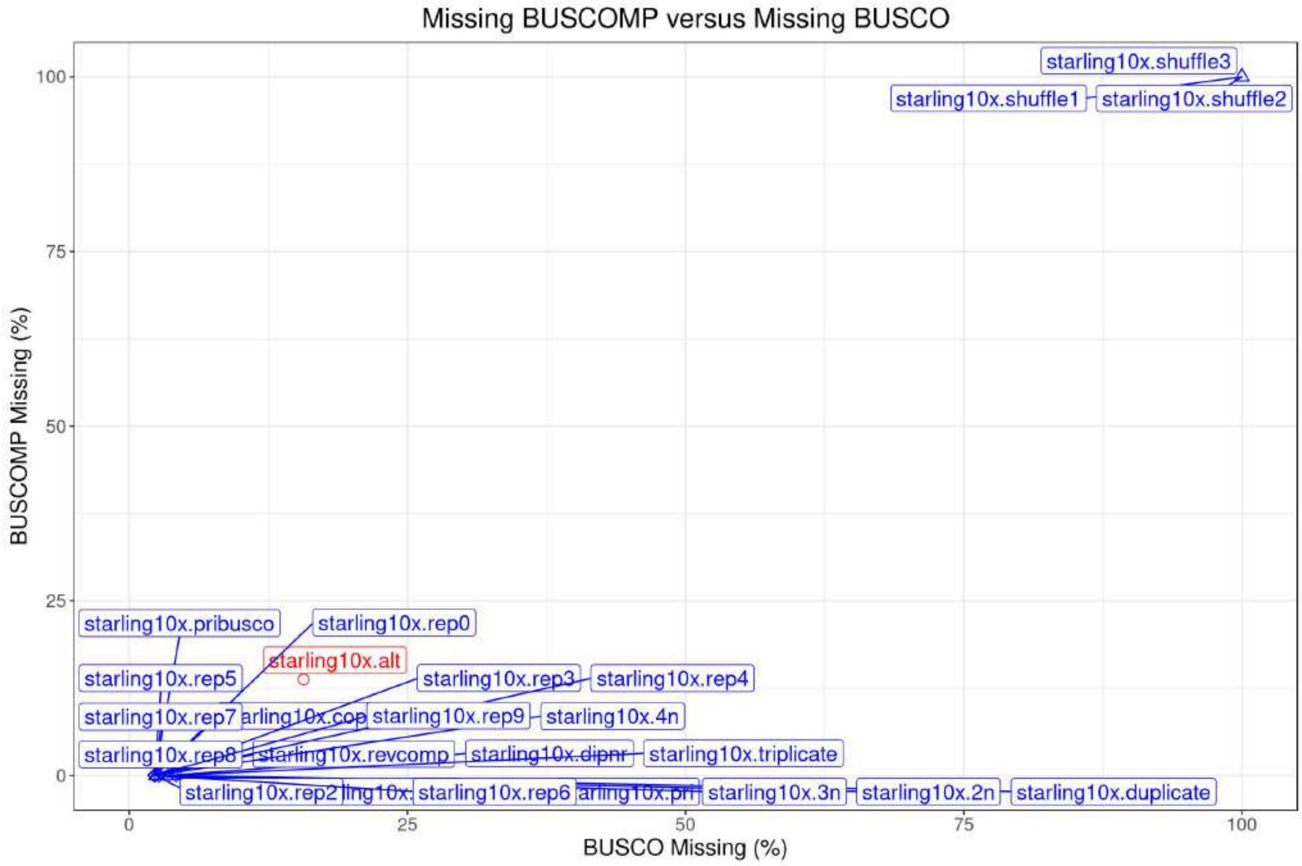

2.3 Genome contiguity assessment plots

In general, a good assembly will be in fewer, bigger pieces. This is approximated using NG50 and LG50, which are the min. length and number of contigs/scaffolds required to cover at least half the genome. These stats use the given genome size of 1.1 Gb.

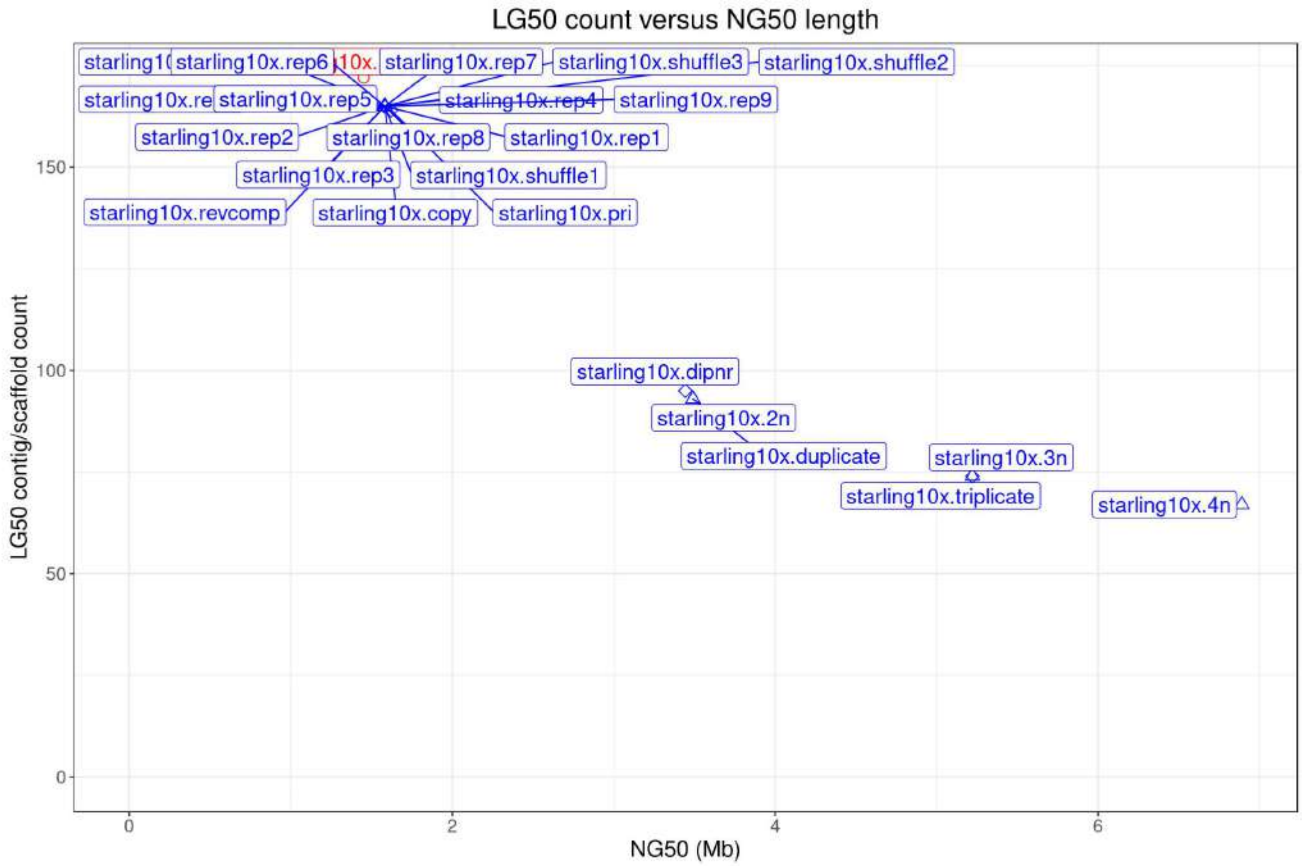

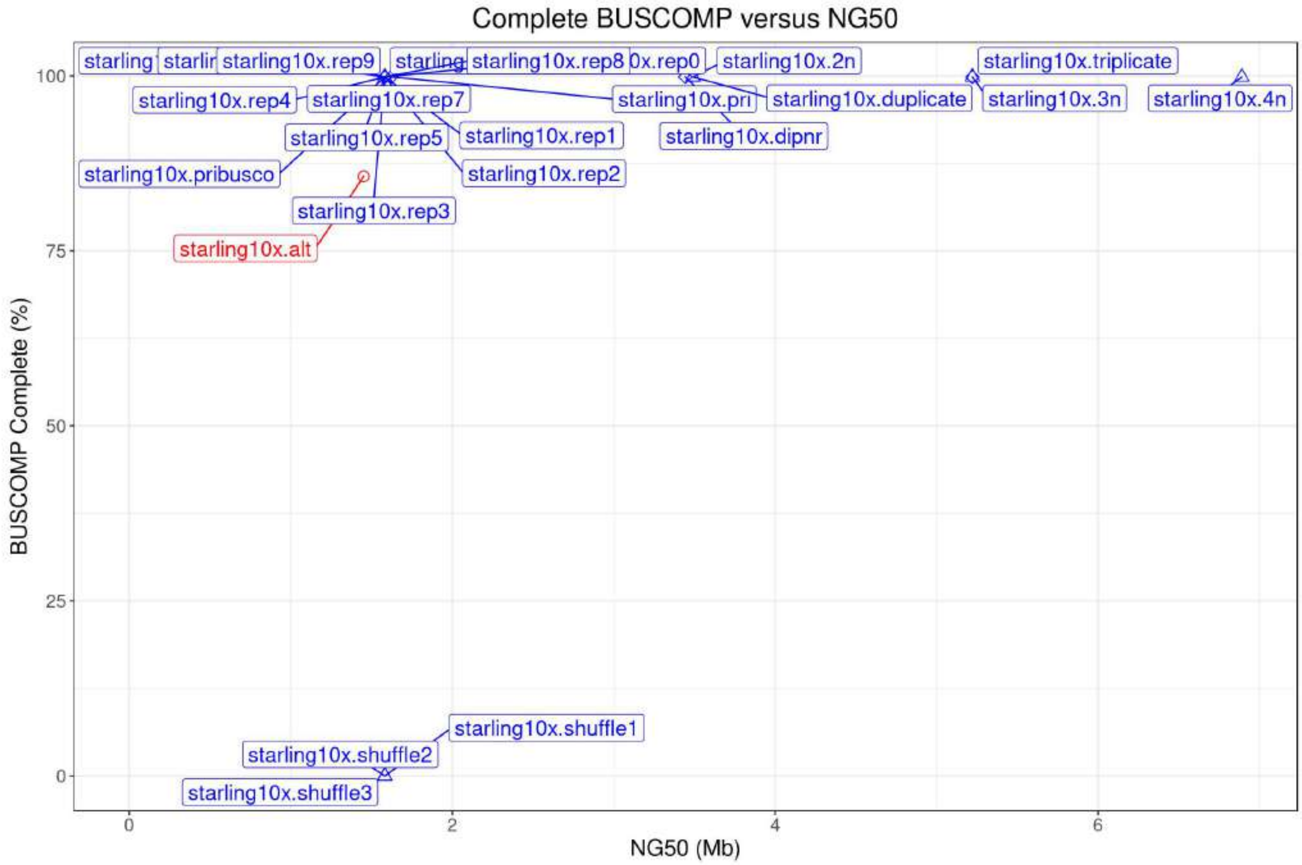
Complete BUSCOMP versus NG50
starling10x.rep9
starling10x.rep8
starling10x.2n
starling10x.triplicate
starling10x.rep4
starling10x.rep7
starling10x.pri
starling10x.duplicate
starling10x.3n
starling10x.4n
starling10x.rep5
starling10x.rep1
starling10x.dipnr
starling10x.pribusco
starling10x.rep2
starling10x.rep3
starling10x.alt
starling10x.shuffle1
starling10x.shuffle2
starling10x.shuffle3
BUSCOMP Complete (%)
NG50 (Mb)
0
25
50
75
100
0
2
4
6

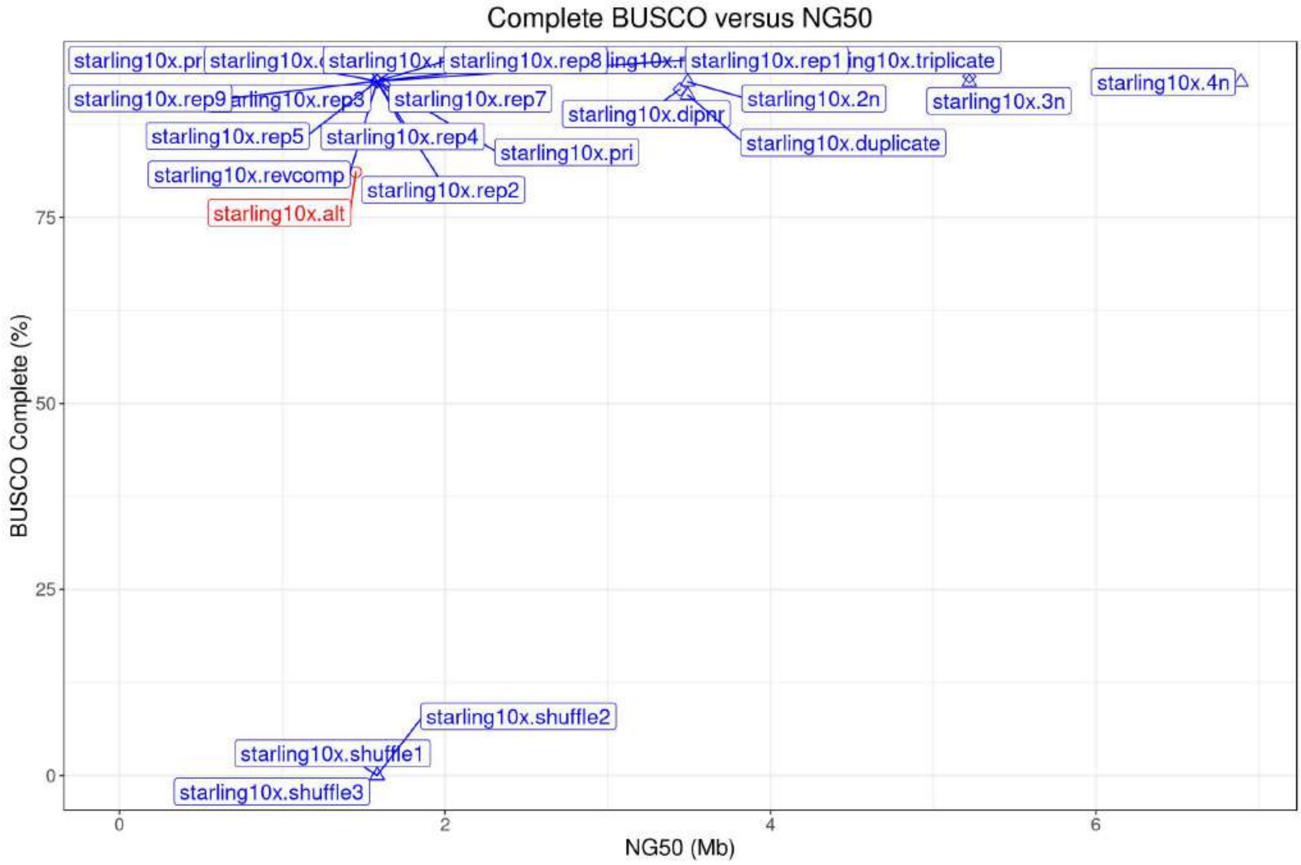
Complete BUSCO versus NG50
starling10x.rep8
starling10x.rep1
starling10x.4n
starling10x.rep9
starling10x.rep7
starling10x.2n
starling10x.3n
starling10x.rep5
starling10x.rep4
starling10x.pri
starling10x.duplicate
starling10x.revcomp
starling10x.rep2
starling10x.alt
starling10x.shuffle2
starling10x.shuffle3
BUSCO Complete (%)
NG50 (Mb)
0
25
50
75
0
2
4
6

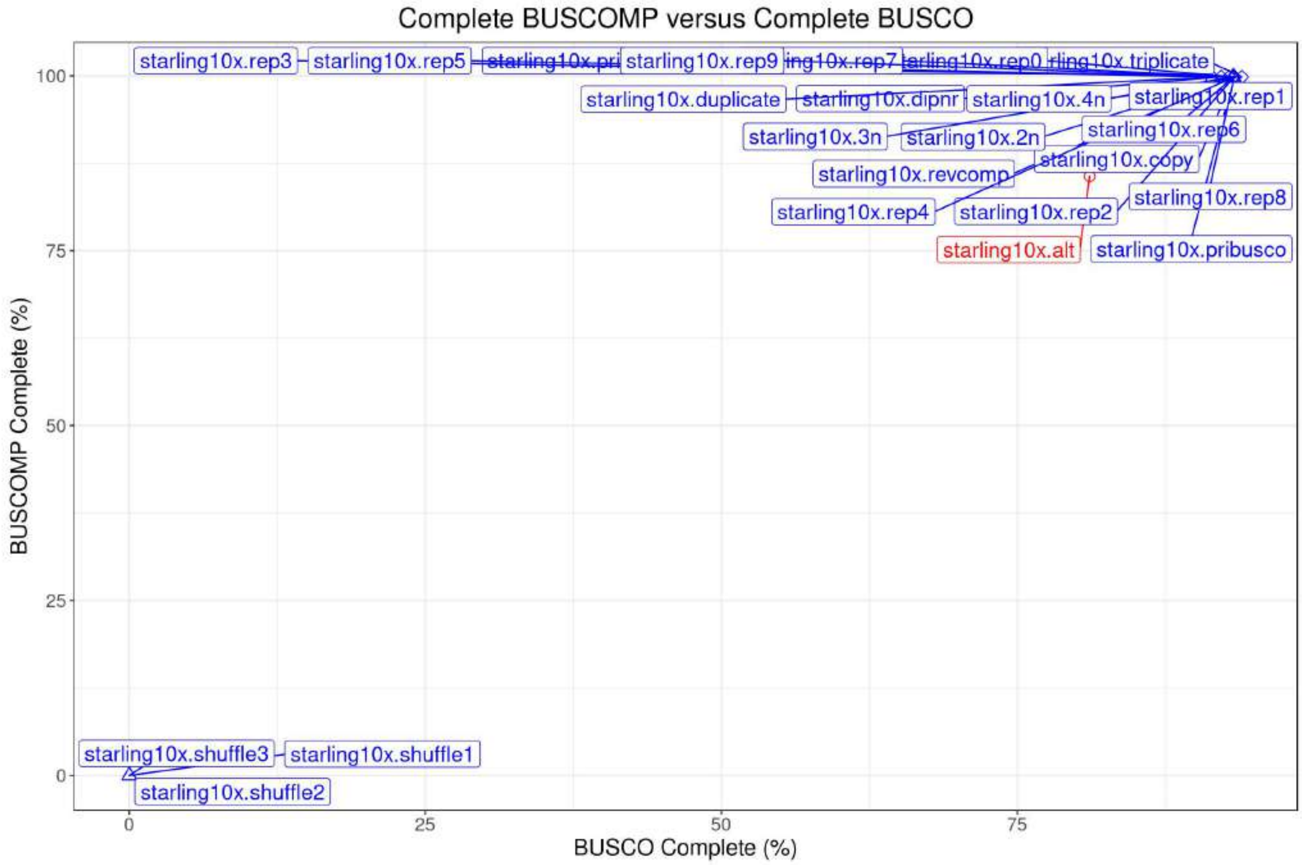

NOTE: To modify these plots and tables, edit the `*.genomes.tdt` and `*.NxLxxIDxx.rdata.tdt` files and re-knit the `*.NxLxxIDxx.Rmd` file.

3 BUSCO Ratings

Compiled BUSCO results for 24 assemblies and 6 groups have been saved in `starling10xV3.genomes.tdt`. BUSCO ratings are defined (quoting from the **BUSCO v3 User Guide (http://gitlab.com/ezlab/busco/raw/master/BUSCO_v3_userguide.pdf)** as:

- `Complete` : Single-copy hits where "BUSCO matches have scored within the expected range of scores and within the expected range of length alignments to the BUSCO profile."
- `Duplicated` : As `Complete` but 2+ copies.
- `Fragmented` : "BUSCO matches … within the range of scores but not within the range of length alignments to the BUSCO profile."
- `Missing` : "Either no significant matches at all, or the BUSCO matches scored below the range of scores for the BUSCO profile."

Genome	N	Complete	Single	Duplicated	Fragmented	Missing
starling10x.dipnr	4915	4532	842	3690	212	171
starling10x.pri	4915	4565	4470	95	219	131
starling10x.alt	4915	3986	3948	38	159	770
Pseudodip	4915	4629	4553	76	185	101
starling10x.pribusco	4915	4586	4490	96	217	112
starling10x.revcomp	4915	4584	4504	80	204	127
Core	4915	4760	4704	56	81	74
starling10x.copy	4915	4586	4490	96	217	112
starling10x.duplicate	4915	4496	144	4352	221	198
starling10x.triplicate	4915	4618	427	4191	90	207
Duplication	4915	4750	4651	99	88	77
starling10x.shuffle1	4915	0	0	0	0	4915
starling10x.shuffle2	4915	0	0	0	0	4915
starling10x.shuffle3	4915	0	0	0	0	4915
starling10x.2n	4915	4582	4487	95	214	119
starling10x.3n	4915	4581	4486	95	215	119
starling10x.4n	4915	4581	4486	95	215	119
Size	4915	4582	4487	95	215	118
starling10x.rep0	4915	4586	4490	96	217	112
starling10x.rep1	4915	4586	4490	96	217	112

Genome	N	Complete	Single	Duplicated	Fragmented	Missing
starling10x.rep2	4915	4586	4490	96	217	112
starling10x.rep3	4915	4586	4490	96	217	112
starling10x.rep4	4915	4586	4490	96	217	112
starling10x.rep5	4915	4586	4490	96	217	112
starling10x.rep6	4915	4586	4490	96	217	112
starling10x.rep7	4915	4586	4490	96	217	112
starling10x.rep8	4915	4586	4490	96	217	112
starling10x.rep9	4915	4586	4490	96	217	112
Replicates	4915	4586	4490	96	217	112
BUSCOMP	4915	4764	4707	57	80	71

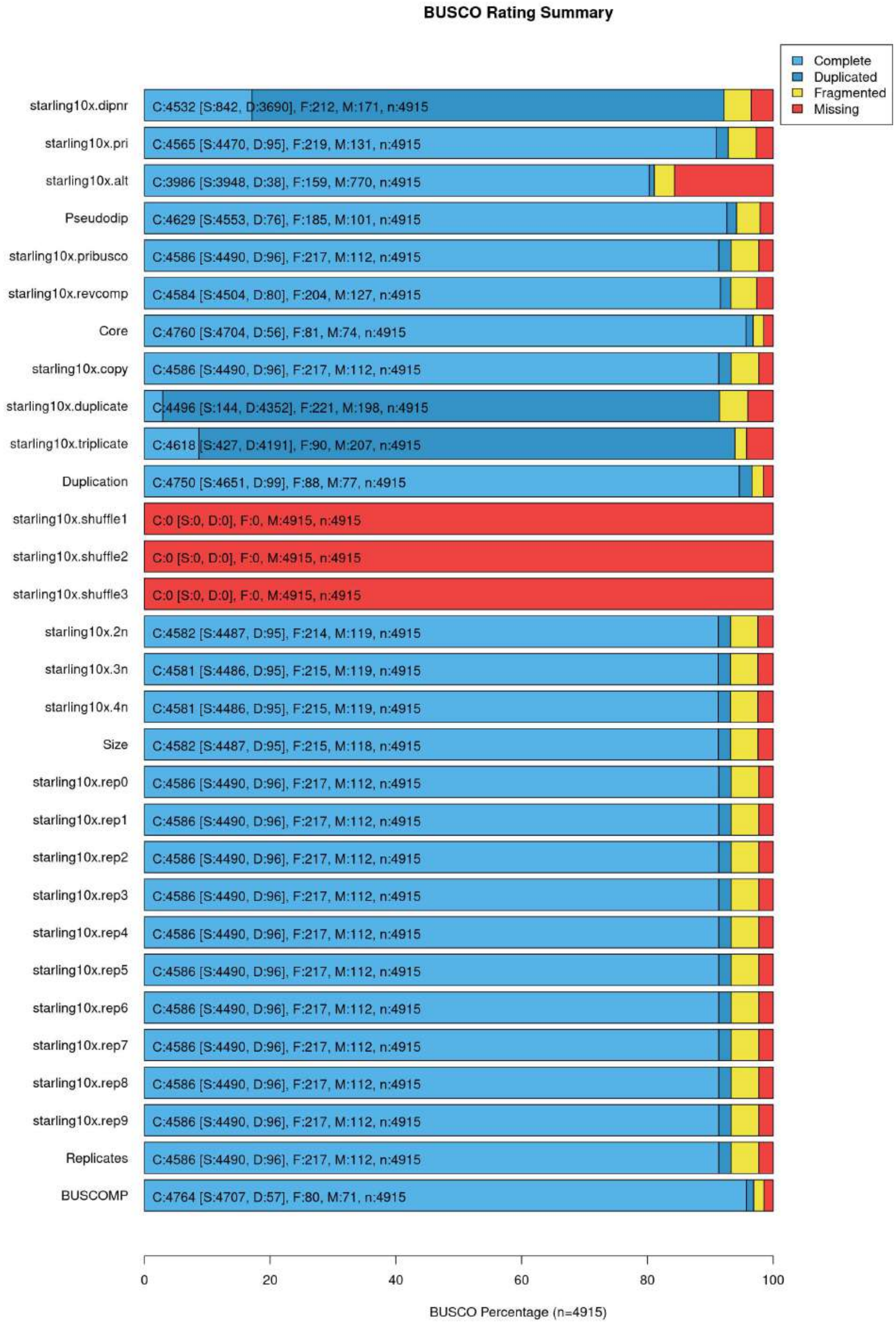

3.1 Genome Groups

`BUSCOMP` compiled the following groups of genomes (where BUSCO data was loaded), keeping the "best" rating for each BUSCO gene across the group:

- **Pseudodip**: `starling10x.dipnr` `starling10x.pri` `starling10x.alt`
- **Core**: `starling10x.dipnr` `starling10x.pri` `starling10x.alt` `starling10x.pribusco` `starling10x.revcomp`
- **Duplication**: `starling10x.copy` `starling10x.duplicate` `starling10x.triplicate`
- **Size**: `starling10x.shuffle1` `starling10x.shuffle2` `starling10x.shuffle3` `starling10x.2n` `starling10x.3n` `starling10x.4n`

- **Replicates**: `starling10x.rep0` `starling10x.rep1` `starling10x.rep2` `starling10x.rep3` `starling10x.rep4` `starling10x.rep5` `starling10x.rep6` `starling10x.rep7` `starling10x.rep8` `starling10x.rep9`
- **BUSCOMP**: `starling10x.dipnr` `starling10x.pri` `starling10x.alt` `starling10x.pribusco` `starling10x.revcomp` `starling10x.copy` `starling10x.duplicate` `starling10x.triplicate` `starling10x.shuffle1` `starling10x.shuffle2` `starling10x.shuffle3` `starling10x.2n` `starling10x.3n` `starling10x.4n` `starling10x.rep0` `starling10x.rep1` `starling10x.rep2` `starling10x.rep3` `starling10x.rep4` `starling10x.rep5` `starling10x.rep6` `starling10x.rep7` `starling10x.rep8` `starling10x.rep9`

3.2 BUSCO Summary

```
starling10x.dipnr BUSCO Results:
        C:92.2%[S:17.1%,D:75.1%],F:4.3%,M:3.5%,n:4915

starling10x.pri BUSCO Results:
        C:92.9%[S:90.9%,D:1.9%],F:4.5%,M:2.7%,n:4915

starling10x.alt BUSCO Results:
        C:81.1%[S:80.3%,D:0.8%],F:3.2%,M:15.7%,n:4915

Pseudodip BUSCO Results:
        C:94.2%[S:92.6%,D:1.5%],F:3.8%,M:2.1%,n:4915

starling10x.pribusco BUSCO Results:
        C:93.3%[S:91.4%,D:2.0%],F:4.4%,M:2.3%,n:4915

starling10x.revcomp BUSCO Results:
        C:93.3%[S:91.6%,D:1.6%],F:4.2%,M:2.6%,n:4915

Core BUSCO Results:
        C:96.8%[S:95.7%,D:1.1%],F:1.6%,M:1.5%,n:4915

starling10x.copy BUSCO Results:
        C:93.3%[S:91.4%,D:2.0%],F:4.4%,M:2.3%,n:4915

starling10x.duplicate BUSCO Results:
        C:91.5%[S:2.9%,D:88.5%],F:4.5%,M:4.0%,n:4915

starling10x.triplicate BUSCO Results:
        C:94.0%[S:8.7%,D:85.3%],F:1.8%,M:4.2%,n:4915

Duplication BUSCO Results:
        C:96.6%[S:94.6%,D:2.0%],F:1.8%,M:1.6%,n:4915

starling10x.shuffle1 BUSCO Results:
        C:0.0%[S:0.0%,D:0.0%],F:0.0%,M:100.0%,n:4915

starling10x.shuffle2 BUSCO Results:
        C:0.0%[S:0.0%,D:0.0%],F:0.0%,M:100.0%,n:4915

starling10x.shuffle3 BUSCO Results:
        C:0.0%[S:0.0%,D:0.0%],F:0.0%,M:100.0%,n:4915

starling10x.2n BUSCO Results:
        C:93.2%[S:91.3%,D:1.9%],F:4.4%,M:2.4%,n:4915

starling10x.3n BUSCO Results:
        C:93.2%[S:91.3%,D:1.9%],F:4.4%,M:2.4%,n:4915

starling10x.4n BUSCO Results:
        C:93.2%[S:91.3%,D:1.9%],F:4.4%,M:2.4%,n:4915

Size BUSCO Results:
        C:93.2%[S:91.3%,D:1.9%],F:4.4%,M:2.4%,n:4915

starling10x.rep0 BUSCO Results:
        C:93.3%[S:91.4%,D:2.0%],F:4.4%,M:2.3%,n:4915

starling10x.rep1 BUSCO Results:
        C:93.3%[S:91.4%,D:2.0%],F:4.4%,M:2.3%,n:4915

starling10x.rep2 BUSCO Results:
        C:93.3%[S:91.4%,D:2.0%],F:4.4%,M:2.3%,n:4915

starling10x.rep3 BUSCO Results:
        C:93.3%[S:91.4%,D:2.0%],F:4.4%,M:2.3%,n:4915

starling10x.rep4 BUSCO Results:
        C:93.3%[S:91.4%,D:2.0%],F:4.4%,M:2.3%,n:4915

starling10x.rep5 BUSCO Results:
        C:93.3%[S:91.4%,D:2.0%],F:4.4%,M:2.3%,n:4915

starling10x.rep6 BUSCO Results:
        C:93.3%[S:91.4%,D:2.0%],F:4.4%,M:2.3%,n:4915

starling10x.rep7 BUSCO Results:
        C:93.3%[S:91.4%,D:2.0%],F:4.4%,M:2.3%,n:4915

starling10x.rep8 BUSCO Results:
        C:93.3%[S:91.4%,D:2.0%],F:4.4%,M:2.3%,n:4915

starling10x.rep9 BUSCO Results:
```

```
        C:93.3%[S:91.4%,D:2.0%],F:4.4%,M:2.3%,n:4915

Replicates BUSCO Results:
        C:93.3%[S:91.4%,D:2.0%],F:4.4%,M:2.3%,n:4915

BUSCOMP BUSCO Results:
        C:96.9%[S:95.8%,D:1.2%],F:1.6%,M:1.4%,n:4915
```

3.3 BUSCO Gene Details

Full BUSCO results with ratings for each gene have been compiled in `starling10xV3.busco.tdt`:

BuscoID <chr>	starling10x.dipnr <chr>	starling10x.pri <chr>
EOG090F001C	Duplicated	Complete
EOG090F0023	Duplicated	Complete
EOG090F0028	Duplicated	Complete
EOG090F002E	Duplicated	Complete
EOG090F0034	Missing	Missing
EOG090F003F	Duplicated	Complete
EOG090F003R	Duplicated	Complete
EOG090F0040	Duplicated	Complete
EOG090F0042	Duplicated	Complete
EOG090F0043	Duplicated	Complete

1-10 of 4,915 rows | 1-3 of 31 columns Previous 1 2 3 4 5 6 ... 492 Next

3.4 Genome Group BUSCO charts

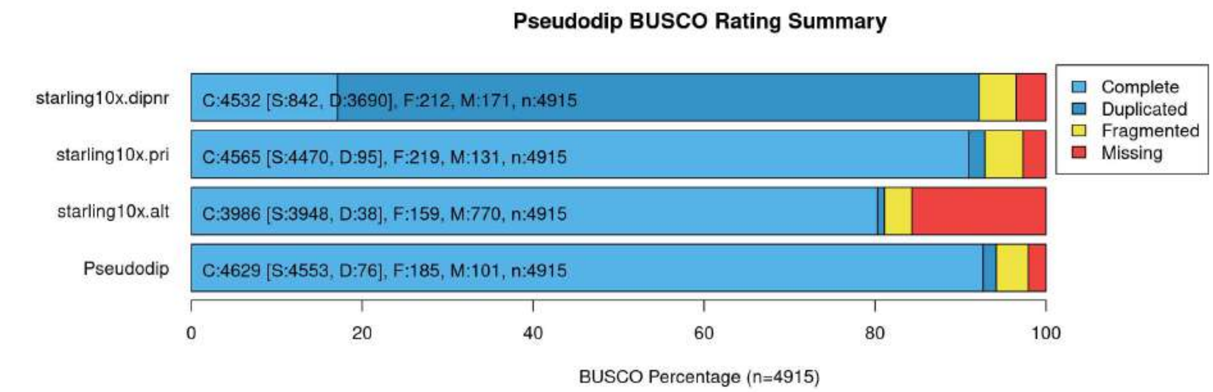

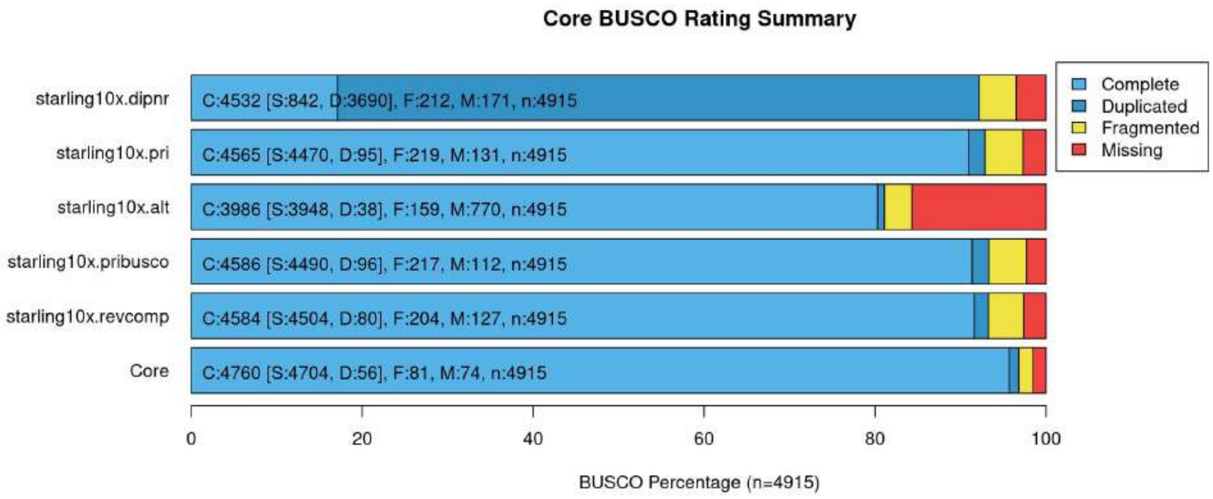

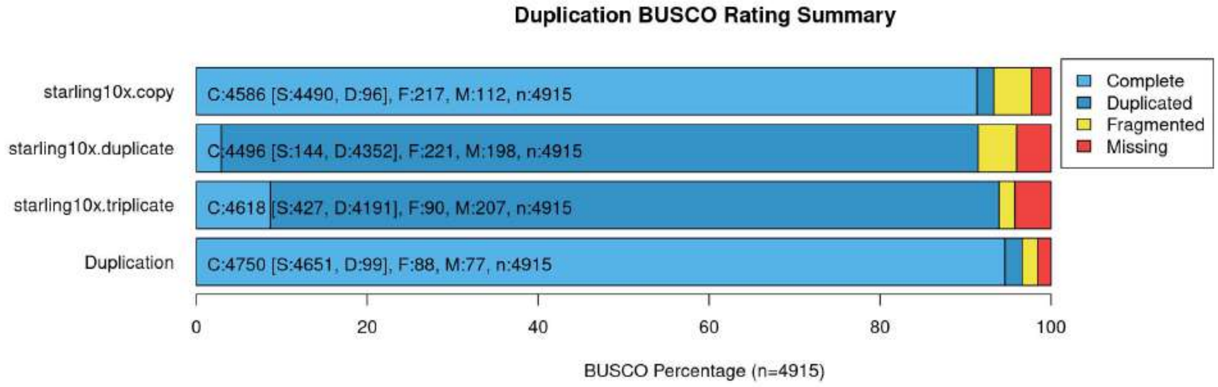
Duplication BUSCO Rating Summary
starling10x.copy
C:4586 [S:4490, D:96], F:217, M:112, n:4915
starling10x.duplicate
C:4496 [S:144, D:4352], F:221, M:198, n:4915
starling10x.triplicate
C:4618 [S:427, D:4191], F:90, M:207, n:4915
Duplication
C:4750 [S:4651, D:99], F:88, M:77, n:4915
Complete
Duplicated
Fragmented
Missing
0
20
40
60
80
100
BUSCO Percentage (n=4915)

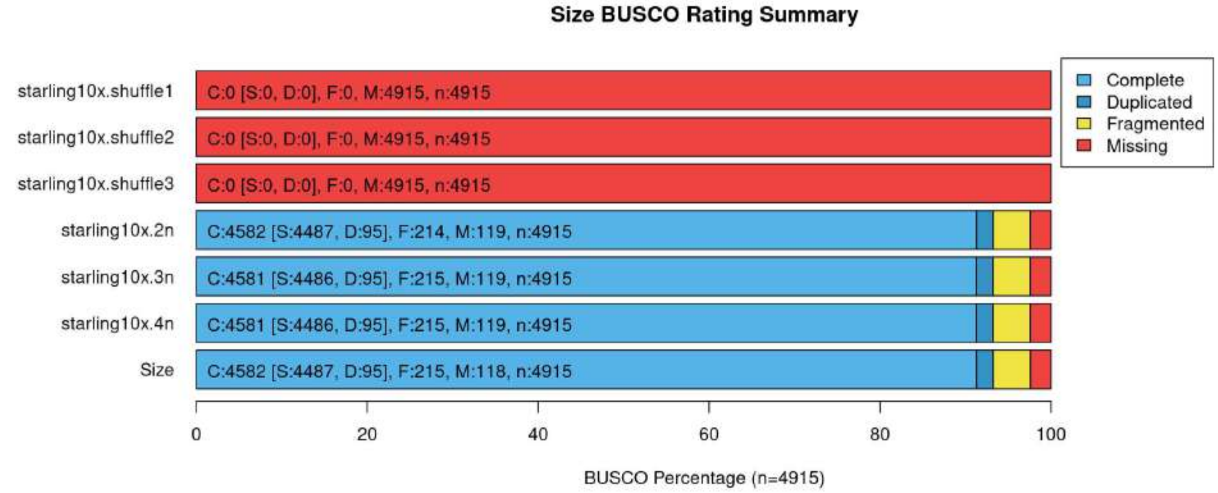
Size BUSCO Rating Summary
starling10x.shuffle1
C:0 [S:0, D:0], F:0, M:4915, n:4915
starling10x.shuffle2
C:0 [S:0, D:0], F:0, M:4915, n:4915
starling10x.shuffle3
C:0 [S:0, D:0], F:0, M:4915, n:4915
starling10x.2n
C:4582 [S:4487, D:95], F:214, M:119, n:4915
starling10x.3n
C:4581 [S:4486, D:95], F:215, M:119, n:4915
starling10x.4n
C:4581 [S:4486, D:95], F:215, M:119, n:4915
Size
C:4582 [S:4487, D:95], F:215, M:118, n:4915
Complete
Duplicated
Fragmented
Missing
0
20
40
60
80
100
BUSCO Percentage (n=4915)

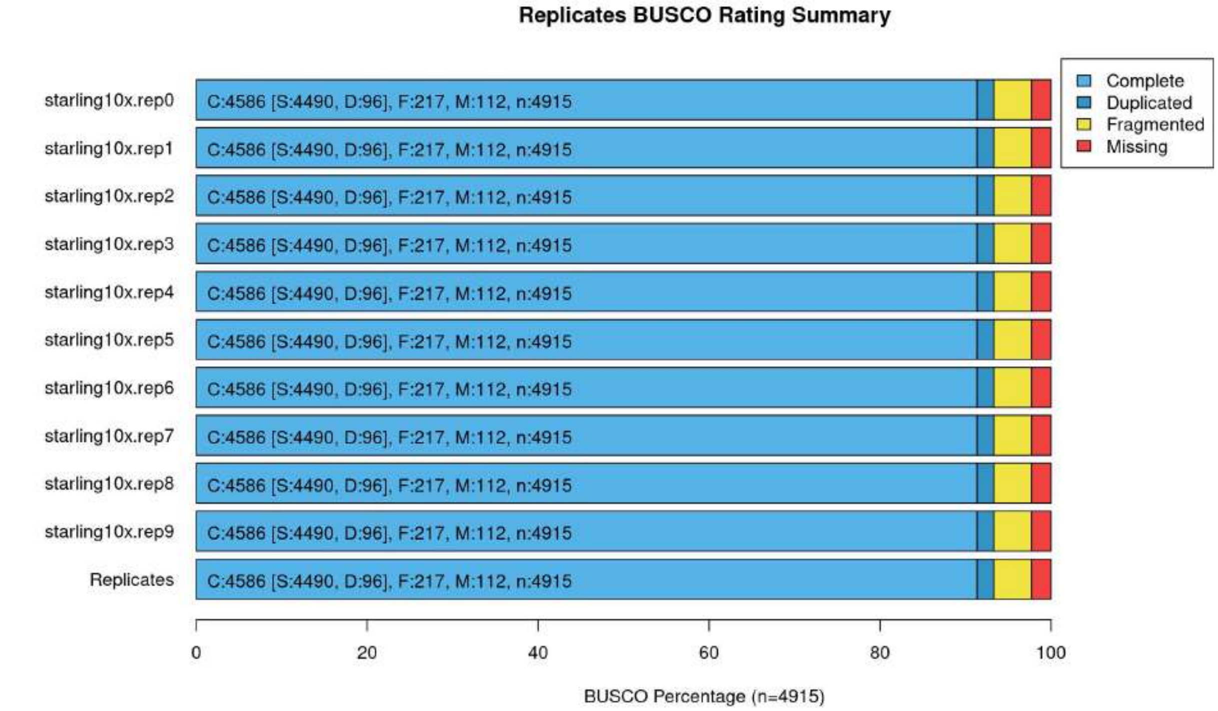
Replicates BUSCO Rating Summary
starling10x.rep0
C:4586 [S:4490, D:96], F:217, M:112, n:4915
starling10x.rep1
C:4586 [S:4490, D:96], F:217, M:112, n:4915
starling10x.rep2
C:4586 [S:4490, D:96], F:217, M:112, n:4915
starling10x.rep3
C:4586 [S:4490, D:96], F:217, M:112, n:4915
starling10x.rep4
C:4586 [S:4490, D:96], F:217, M:112, n:4915
starling10x.rep5
C:4586 [S:4490, D:96], F:217, M:112, n:4915
starling10x.rep6
C:4586 [S:4490, D:96], F:217, M:112, n:4915
starling10x.rep7
C:4586 [S:4490, D:96], F:217, M:112, n:4915
starling10x.rep8
C:4586 [S:4490, D:96], F:217, M:112, n:4915
starling10x.rep9
C:4586 [S:4490, D:96], F:217, M:112, n:4915
Replicates
C:4586 [S:4490, D:96], F:217, M:112, n:4915
Complete
Duplicated
Fragmented
Missing
0
20
40
60
80
100
BUSCO Percentage (n=4915)

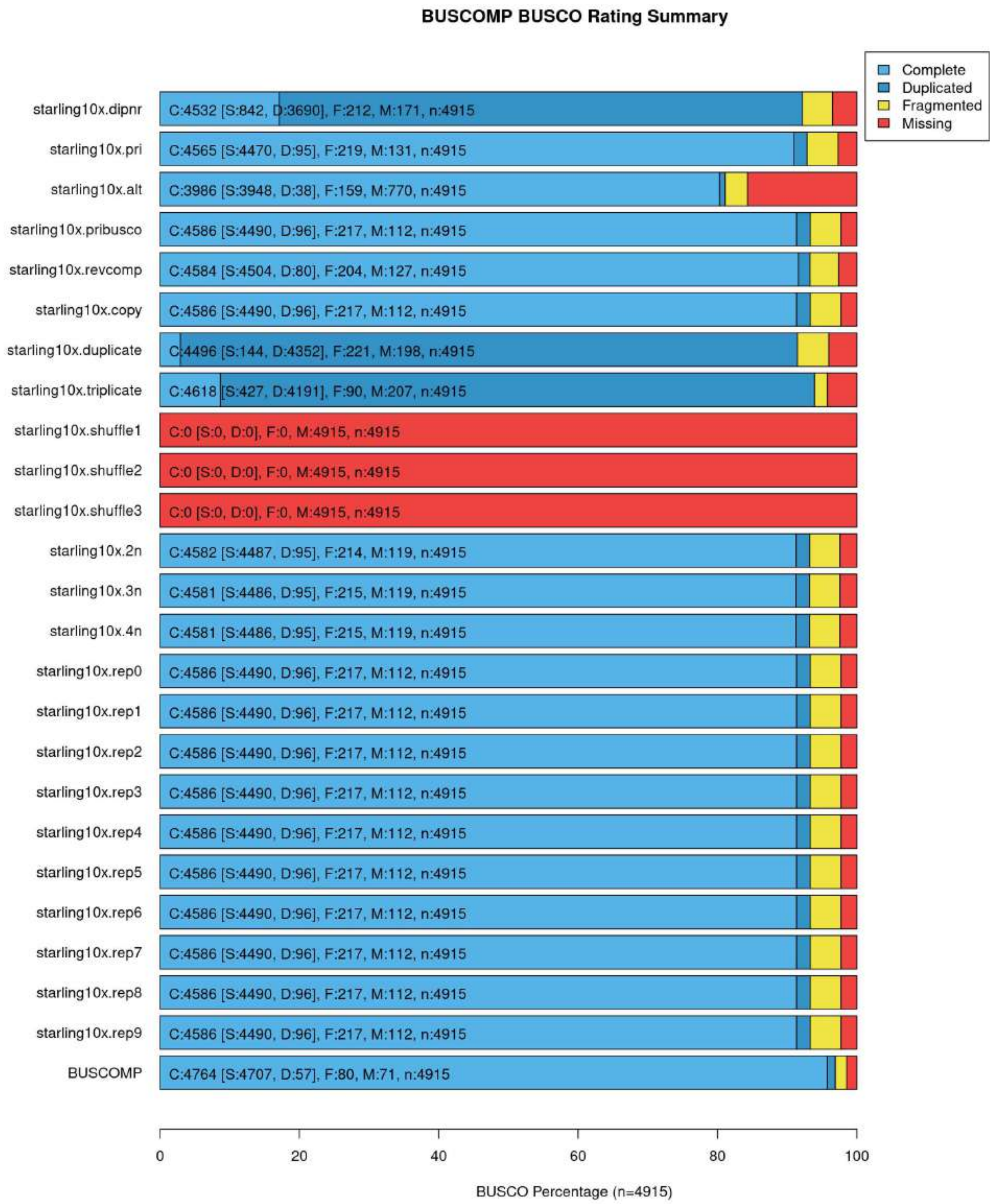

4 BUSCOMP Ratings

The best complete BUSCO hit results (based on `Score` and `Length`) have been compiled in `starling10xV3.buscoseq.tdt`. The `Genome` field indicates the assembly with the best hit, which is followed by details of that hit (`Contig`, `Start`, `End`, `Score`, `Length`). BUSCOMP ratings for each assembly are then given in subsequent fields:

```
* `Identical`: 100% coverage and 100% identity in at least one contig/scaffold.
* `Complete`: 95%+ Coverage in a single contig/scaffold. (Note: accuracy/identity is not considered.)
* `Duplicated`: 95%+ Coverage in 2+ contigs/scaffolds.
* `Fragmented`: 95%+ combined coverage but not in any single contig/scaffold.
* `Partial`: 40-95% combined coverage.
* `Ghost`: Hits meeting local cutoff but <40% combined coverage.
* `Missing`: No hits meeting local cutoff.
```

Genome <chr>	BuscoID <chr>	Status <chr>
starling10x.2n	EOG090F001C	Complete
starling10x.2n	EOG090F0023	Complete

Genome <chr>	BuscoID <chr>	Status <chr>
starling10x.revcomp	EOG090F0028	Complete
starling10x.alt	EOG090F002E	Complete
Missing	EOG090F0034	Missing
starling10x.revcomp	EOG090F003F	Complete
starling10x.2n	EOG090F003R	Complete
starling10x.alt	EOG090F0040	Complete
starling10x.alt	EOG090F0042	Complete
starling10x.revcomp	EOG090F0043	Complete

1-10 of 4,915 rows | 1-3 of 8 columns Previous 1 2 3 4 5 6 ... 492 Next

4.1 BUSCOSeq Rating Summary

BUSCOMP ratings (see above) are compiled to summary statistics in `starling10xV3.N3L20ID0U.ratings.tdt`. Note that `Identical` ratings in this table will also be rated as `Complete`, which in turn are `Single` or `Duplicated`. Percentage summaries are plotted below, along with a BUSCO-style one-line summary per assembly/group.

NOTE: Group summaries do not include `Identical` ratings.

X.	Genome	N	Identical	Complete	Single	Duplicated	Fragmented	Partial	Ghost	Missing
1	starling10x.dipnr	4707	3828	4700	657	4043	0	7	0	0
2	starling10x.pri	4707	3448	4700	4675	25	0	7	0	0
3	starling10x.alt	4707	1549	4031	4024	7	0	21	8	647
4	Pseudodip	4707	0	4700	4681	19	0	7	0	0
5	starling10x.pribusco	4707	3448	4699	4679	20	0	7	0	1
6	starling10x.revcomp	4707	3448	4699	4679	20	0	7	0	1
7	Core	4707	0	4700	4685	15	0	7	0	0
8	starling10x.copy	4707	3448	4699	4679	20	0	7	0	1
9	starling10x.duplicate	4707	3448	4699	0	4699	0	7	0	1
10	starling10x.triplicate	4707	3455	4699	0	4699	0	7	0	1
11	Duplication	4707	0	4699	4679	20	0	7	0	1
12	starling10x.shuffle1	4707	0	0	0	0	0	0	0	4707
13	starling10x.shuffle2	4707	0	0	0	0	0	0	0	4707
14	starling10x.shuffle3	4707	0	0	0	0	0	0	0	4707
15	starling10x.2n	4707	3448	4699	4679	20	0	7	0	1
16	starling10x.3n	4707	3448	4699	4679	20	0	7	0	1
17	starling10x.4n	4707	3448	4699	4679	20	0	7	0	1
18	Size	4707	0	4699	4679	20	0	7	0	1
19	starling10x.rep0	4707	3448	4699	4679	20	0	7	0	1
20	starling10x.rep1	4707	3448	4699	4679	20	0	7	0	1
21	starling10x.rep2	4707	3448	4699	4679	20	0	7	0	1
22	starling10x.rep3	4707	3448	4699	4679	20	0	7	0	1
23	starling10x.rep4	4707	3448	4699	4679	20	0	7	0	1
24	starling10x.rep5	4707	3448	4699	4679	20	0	7	0	1
25	starling10x.rep6	4707	3448	4699	4679	20	0	7	0	1
26	starling10x.rep7	4707	3448	4699	4679	20	0	7	0	1
27	starling10x.rep8	4707	3448	4699	4679	20	0	7	0	1
28	starling10x.rep9	4707	3448	4699	4679	20	0	7	0	1
29	Replicates	4707	0	4699	4679	20	0	7	0	1
30	BUSCOMP	4707	0	4700	4685	15	0	7	0	0

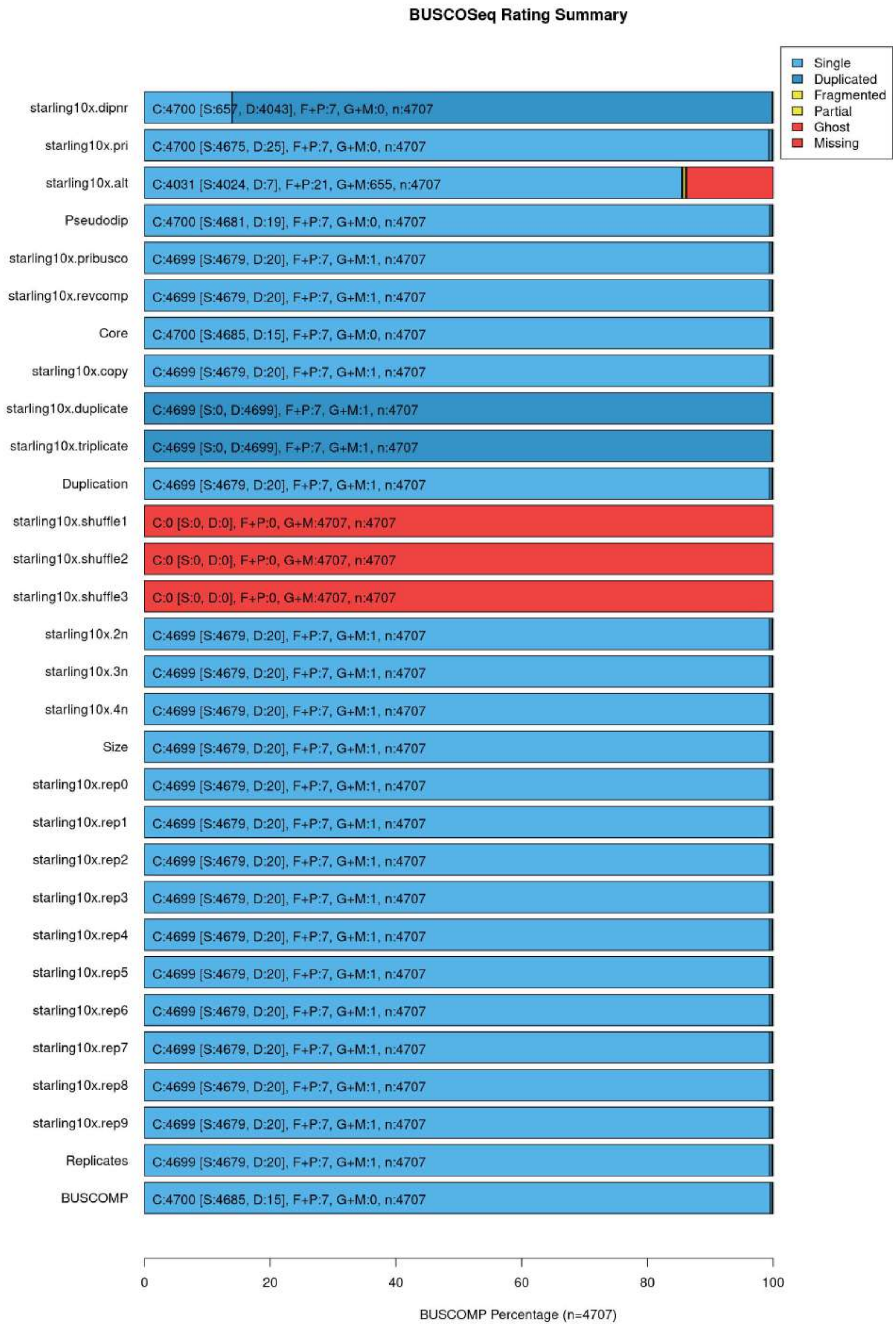
BUSCOSeq Rating Summary
Single
Duplicated
Fragmented
Partial
Ghost
Missing
starling10x.dipnr
C:4700 [S:657, D:4043], F+P:7, G+M:0, n:4707
starling10x.pri
C:4700 [S:4675, D:25], F+P:7, G+M:0, n:4707
starling10x.alt
C:4031 [S:4024, D:7], F+P:21, G+M:655, n:4707
Pseudodip
C:4700 [S:4681, D:19], F+P:7, G+M:0, n:4707
starling10x.pribusco
C:4699 [S:4679, D:20], F+P:7, G+M:1, n:4707
starling10x.revcomp
C:4699 [S:4679, D:20], F+P:7, G+M:1, n:4707
Core
C:4700 [S:4685, D:15], F+P:7, G+M:0, n:4707
starling10x.copy
C:4699 [S:4679, D:20], F+P:7, G+M:1, n:4707
starling10x.duplicate
C:4699 [S:0, D:4699], F+P:7, G+M:1, n:4707
starling10x.triplicate
C:4699 [S:0, D:4699], F+P:7, G+M:1, n:4707
Duplication
C:4699 [S:4679, D:20], F+P:7, G+M:1, n:4707
starling10x.shuffle1
C:0 [S:0, D:0], F+P:0, G+M:4707, n:4707
starling10x.shuffle2
C:0 [S:0, D:0], F+P:0, G+M:4707, n:4707
starling10x.shuffle3
C:0 [S:0, D:0], F+P:0, G+M:4707, n:4707
starling10x.2n
C:4699 [S:4679, D:20], F+P:7, G+M:1, n:4707
starling10x.3n
C:4699 [S:4679, D:20], F+P:7, G+M:1, n:4707
starling10x.4n
C:4699 [S:4679, D:20], F+P:7, G+M:1, n:4707
Size
C:4699 [S:4679, D:20], F+P:7, G+M:1, n:4707
starling10x.rep0
C:4699 [S:4679, D:20], F+P:7, G+M:1, n:4707
starling10x.rep1
C:4699 [S:4679, D:20], F+P:7, G+M:1, n:4707
starling10x.rep2
C:4699 [S:4679, D:20], F+P:7, G+M:1, n:4707
starling10x.rep3
C:4699 [S:4679, D:20], F+P:7, G+M:1, n:4707
starling10x.rep4
C:4699 [S:4679, D:20], F+P:7, G+M:1, n:4707
starling10x.rep5
C:4699 [S:4679, D:20], F+P:7, G+M:1, n:4707
starling10x.rep6
C:4699 [S:4679, D:20], F+P:7, G+M:1, n:4707
starling10x.rep7
C:4699 [S:4679, D:20], F+P:7, G+M:1, n:4707
starling10x.rep8
C:4699 [S:4679, D:20], F+P:7, G+M:1, n:4707
starling10x.rep9
C:4699 [S:4679, D:20], F+P:7, G+M:1, n:4707
Replicates
C:4699 [S:4679, D:20], F+P:7, G+M:1, n:4707
BUSCOMP
C:4700 [S:4685, D:15], F+P:7, G+M:0, n:4707
0
20
40
60
80
100
BUSCOMP Percentage (n=4707)

```
BUSCOMP BUSCOMP Results [4764 (96.93%) Complete BUSCOs; 0 (0.00%) BUSCOMP Seqs]:
        C:99.9%[S:99.5%,D:0.3%],F:0.0%,P:0.1%,G:0.0%,M:0.0%,n:4707

Core BUSCOMP Results [4760 (96.85%) Complete BUSCOs; 0 (0.00%) BUSCOMP Seqs]:
        C:99.9%[S:99.5%,D:0.3%],F:0.0%,P:0.1%,G:0.0%,M:0.0%,n:4707

Duplication BUSCOMP Results [4750 (96.64%) Complete BUSCOs; 0 (0.00%) BUSCOMP Seqs]:
        C:99.8%[S:99.4%,D:0.4%],F:0.0%,P:0.1%,G:0.0%,M:0.0%,n:4707

Pseudodip BUSCOMP Results [4629 (94.18%) Complete BUSCOs; 0 (0.00%) BUSCOMP Seqs]:
        C:99.9%[S:99.4%,D:0.4%],F:0.0%,P:0.1%,G:0.0%,M:0.0%,n:4707

Replicates BUSCOMP Results [4586 (93.31%) Complete BUSCOs; 0 (0.00%) BUSCOMP Seqs]:
        C:99.8%[S:99.4%,D:0.4%],F:0.0%,P:0.1%,G:0.0%,M:0.0%,n:4707

Size BUSCOMP Results [4582 (93.22%) Complete BUSCOs; 0 (0.00%) BUSCOMP Seqs]:
        C:99.8%[S:99.4%,D:0.4%],F:0.0%,P:0.1%,G:0.0%,M:0.0%,n:4707


starling10x.2n BUSCOMP Results [4582 (93.22%) Complete BUSCOs; 2820 (59.91%) BUSCOMP Seqs]:
        C:99.8%[S:99.4%,D:0.4%,I:73.3%],F:0.0%,P:0.1%,G:0.0%,M:0.0%,n:4707

starling10x.3n BUSCOMP Results [4581 (93.20%) Complete BUSCOs; 4 (0.08%) BUSCOMP Seqs]:
        C:99.8%[S:99.4%,D:0.4%,I:73.3%],F:0.0%,P:0.1%,G:0.0%,M:0.0%,n:4707

starling10x.4n BUSCOMP Results [4581 (93.20%) Complete BUSCOs; 1 (0.02%) BUSCOMP Seqs]:
        C:99.8%[S:99.4%,D:0.4%,I:73.3%],F:0.0%,P:0.1%,G:0.0%,M:0.0%,n:4707

starling10x.alt BUSCOMP Results [3986 (81.10%) Complete BUSCOs; 566 (12.02%) BUSCOMP Seqs]:
        C:85.6%[S:85.5%,D:0.1%,I:32.9%],F:0.0%,P:0.4%,G:0.2%,M:13.7%,n:4707

starling10x.copy BUSCOMP Results [4586 (93.31%) Complete BUSCOs; 47 (1.00%) BUSCOMP Seqs]:
        C:99.8%[S:99.4%,D:0.4%,I:73.3%],F:0.0%,P:0.1%,G:0.0%,M:0.0%,n:4707

starling10x.dipnr BUSCOMP Results [4532 (92.21%) Complete BUSCOs; 11 (0.23%) BUSCOMP Seqs]:
        C:99.9%[S:14.0%,D:85.9%,I:81.3%],F:0.0%,P:0.1%,G:0.0%,M:0.0%,n:4707

starling10x.duplicate BUSCOMP Results [4496 (91.48%) Complete BUSCOs; 1 (0.02%) BUSCOMP Seqs]:
        C:99.8%[S:0.0%,D:99.8%,I:73.3%],F:0.0%,P:0.1%,G:0.0%,M:0.0%,n:4707

starling10x.pri BUSCOMP Results [4565 (92.88%) Complete BUSCOs; 1 (0.02%) BUSCOMP Seqs]:
        C:99.9%[S:99.3%,D:0.5%,I:73.3%],F:0.0%,P:0.1%,G:0.0%,M:0.0%,n:4707

starling10x.pribusco BUSCOMP Results [4586 (93.31%) Complete BUSCOs; 0 (0.00%) BUSCOMP Seqs]:
        C:99.8%[S:99.4%,D:0.4%,I:73.3%],F:0.0%,P:0.1%,G:0.0%,M:0.0%,n:4707

starling10x.rep0 BUSCOMP Results [4586 (93.31%) Complete BUSCOs; 0 (0.00%) BUSCOMP Seqs]:
        C:99.8%[S:99.4%,D:0.4%,I:73.3%],F:0.0%,P:0.1%,G:0.0%,M:0.0%,n:4707

starling10x.rep1 BUSCOMP Results [4586 (93.31%) Complete BUSCOs; 0 (0.00%) BUSCOMP Seqs]:
        C:99.8%[S:99.4%,D:0.4%,I:73.3%],F:0.0%,P:0.1%,G:0.0%,M:0.0%,n:4707

starling10x.rep2 BUSCOMP Results [4586 (93.31%) Complete BUSCOs; 0 (0.00%) BUSCOMP Seqs]:
        C:99.8%[S:99.4%,D:0.4%,I:73.3%],F:0.0%,P:0.1%,G:0.0%,M:0.0%,n:4707

starling10x.rep3 BUSCOMP Results [4586 (93.31%) Complete BUSCOs; 0 (0.00%) BUSCOMP Seqs]:
        C:99.8%[S:99.4%,D:0.4%,I:73.3%],F:0.0%,P:0.1%,G:0.0%,M:0.0%,n:4707

starling10x.rep4 BUSCOMP Results [4586 (93.31%) Complete BUSCOs; 0 (0.00%) BUSCOMP Seqs]:
        C:99.8%[S:99.4%,D:0.4%,I:73.3%],F:0.0%,P:0.1%,G:0.0%,M:0.0%,n:4707

starling10x.rep5 BUSCOMP Results [4586 (93.31%) Complete BUSCOs; 0 (0.00%) BUSCOMP Seqs]:
        C:99.8%[S:99.4%,D:0.4%,I:73.3%],F:0.0%,P:0.1%,G:0.0%,M:0.0%,n:4707

starling10x.rep6 BUSCOMP Results [4586 (93.31%) Complete BUSCOs; 0 (0.00%) BUSCOMP Seqs]:
        C:99.8%[S:99.4%,D:0.4%,I:73.3%],F:0.0%,P:0.1%,G:0.0%,M:0.0%,n:4707

starling10x.rep7 BUSCOMP Results [4586 (93.31%) Complete BUSCOs; 0 (0.00%) BUSCOMP Seqs]:
        C:99.8%[S:99.4%,D:0.4%,I:73.3%],F:0.0%,P:0.1%,G:0.0%,M:0.0%,n:4707

starling10x.rep8 BUSCOMP Results [4586 (93.31%) Complete BUSCOs; 0 (0.00%) BUSCOMP Seqs]:
        C:99.8%[S:99.4%,D:0.4%,I:73.3%],F:0.0%,P:0.1%,G:0.0%,M:0.0%,n:4707

starling10x.rep9 BUSCOMP Results [4586 (93.31%) Complete BUSCOs; 0 (0.00%) BUSCOMP Seqs]:
        C:99.8%[S:99.4%,D:0.4%,I:73.3%],F:0.0%,P:0.1%,G:0.0%,M:0.0%,n:4707

starling10x.revcomp BUSCOMP Results [4584 (93.27%) Complete BUSCOs; 1238 (26.30%) BUSCOMP Seqs]:
        C:99.8%[S:99.4%,D:0.4%,I:73.3%],F:0.0%,P:0.1%,G:0.0%,M:0.0%,n:4707

starling10x.shuffle1 BUSCOMP Results [0 (0.00%) Complete BUSCOs; 0 (0.00%) BUSCOMP Seqs]:
        C:0.0%[S:0.0%,D:0.0%,I:0.0%],F:0.0%,P:0.0%,G:0.0%,M:100.0%,n:4707

starling10x.shuffle2 BUSCOMP Results [0 (0.00%) Complete BUSCOs; 0 (0.00%) BUSCOMP Seqs]:
```

```
        C:0.0%[S:0.0%,D:0.0%,I:0.0%],F:0.0%,P:0.0%,G:0.0%,M:100.0%,n:4707

starling10x.shuffle3 BUSCOMP Results [0 (0.00%) Complete BUSCOs; 0 (0.00%) BUSCOMP Seqs]:
        C:0.0%[S:0.0%,D:0.0%,I:0.0%],F:0.0%,P:0.0%,G:0.0%,M:100.0%,n:4707

starling10x.triplicate BUSCOMP Results [4618 (93.96%) Complete BUSCOs; 18 (0.38%) BUSCOMP Seqs]:
        C:99.8%[S:0.0%,D:99.8%,I:73.4%],F:0.0%,P:0.1%,G:0.0%,M:0.0%,n:4707
```

4.2 BUSCOSeq Full Results Table

Full BUSCOMP results with ratings for each gene in every assembly and group have been compiled in `starling10xV3.N3L20ID0U.buscomp.tdt`:

BuscoID <chr>	Genome <chr>	starling10x.dipnr <chr>	starling10x.pri <chr>
EOG090F001C	BUSCOFas	Duplicated	Complete
EOG090F0023	BUSCOFas	Duplicated	Complete
EOG090F0028	BUSCOFas	Duplicated	Complete
EOG090F002E	BUSCOFas	Duplicated	Complete
EOG090F003F	BUSCOFas	Duplicated	Complete
EOG090F003R	BUSCOFas	Duplicated	Complete
EOG090F0040	BUSCOFas	Duplicated	Complete
EOG090F0042	BUSCOFas	Duplicated	Complete
EOG090F0043	BUSCOFas	Duplicated	Complete
EOG090F005C	BUSCOFas	Duplicated	Complete

1-10 of 4,707 rows | 1-4 of 32 columns **Previous 1 2 3 4 5 6 ... 471 Next**

4.3 Genome Group BUSCOMP charts

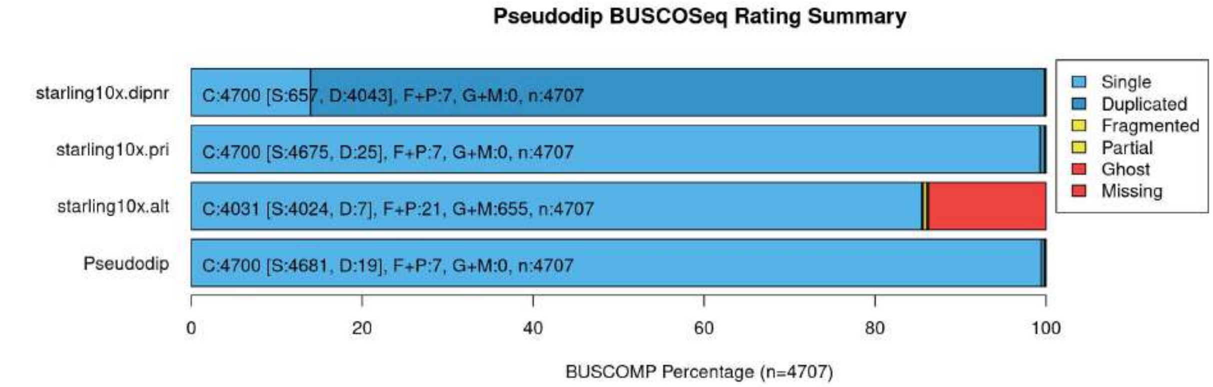

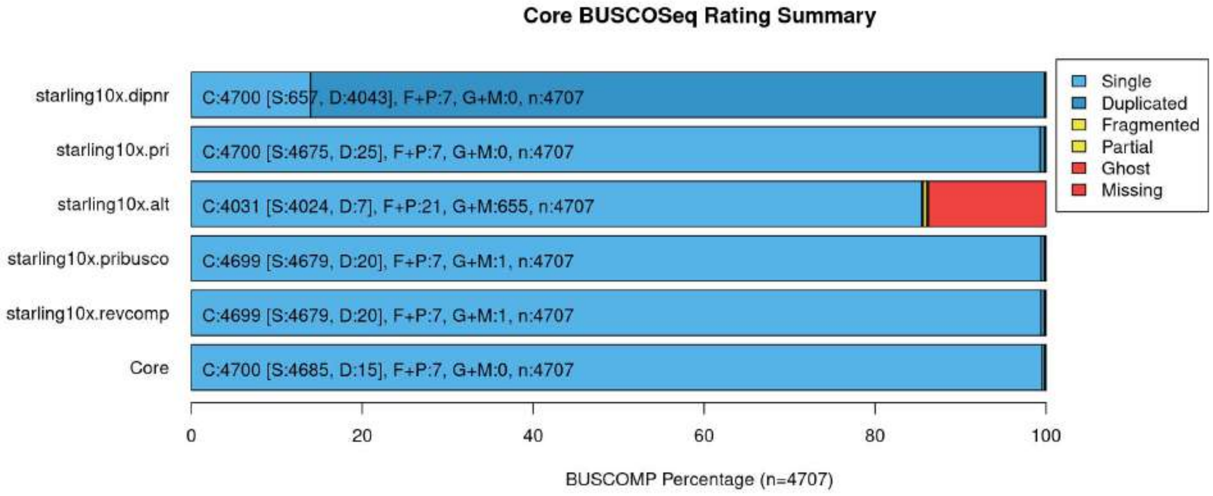

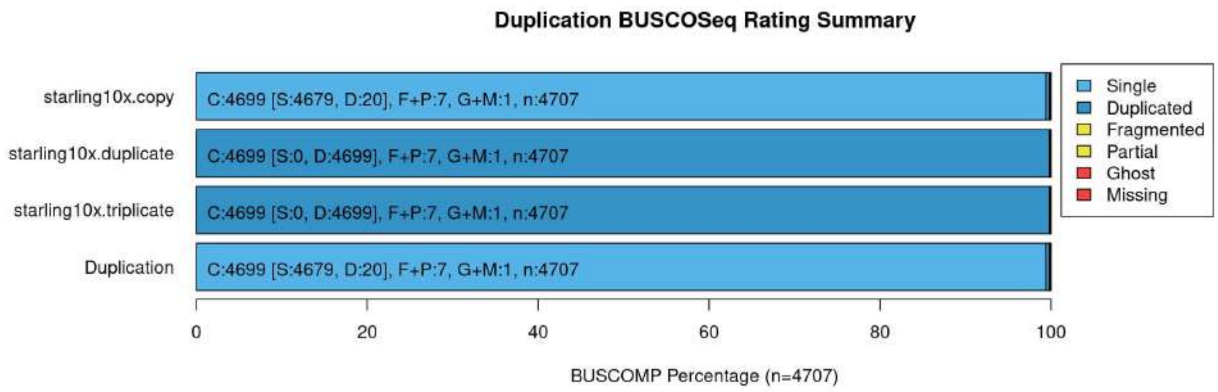
Duplication BUSCOSeq Rating Summary
starling10x.copy
C:4699 [S:4679, D:20], F+P:7, G+M:1, n:4707
starling10x.duplicate
C:4699 [S:0, D:4699], F+P:7, G+M:1, n:4707
starling10x.triplicate
C:4699 [S:0, D:4699], F+P:7, G+M:1, n:4707
Duplication
C:4699 [S:4679, D:20], F+P:7, G+M:1, n:4707
Single
Duplicated
Fragmented
Partial
Ghost
Missing
0
20
40
60
80
100
BUSCOMP Percentage (n=4707)

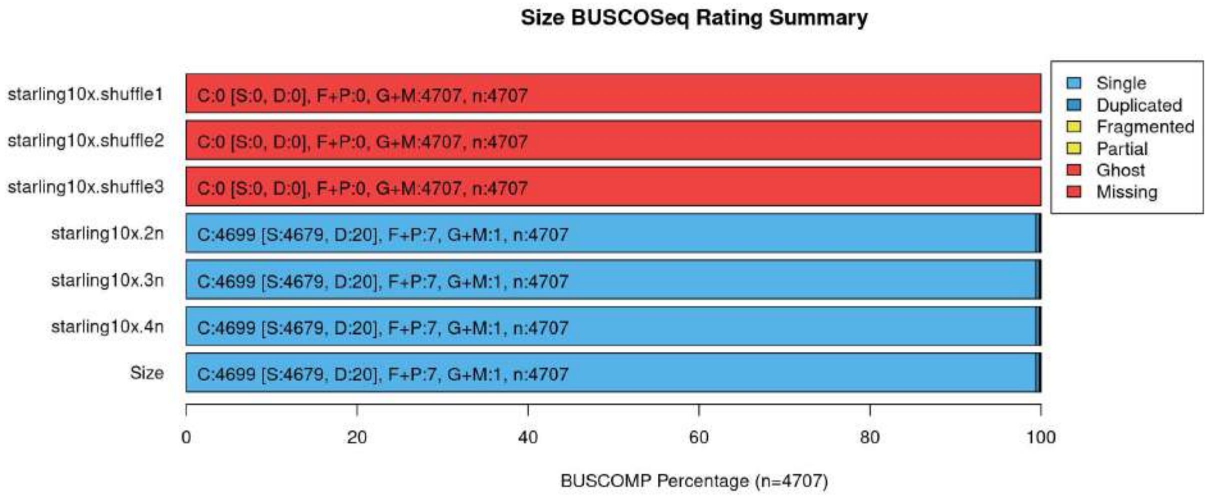
Size BUSCOSeq Rating Summary
starling10x.shuffle1
C:0 [S:0, D:0], F+P:0, G+M:4707, n:4707
starling10x.shuffle2
C:0 [S:0, D:0], F+P:0, G+M:4707, n:4707
starling10x.shuffle3
C:0 [S:0, D:0], F+P:0, G+M:4707, n:4707
starling10x.2n
C:4699 [S:4679, D:20], F+P:7, G+M:1, n:4707
starling10x.3n
C:4699 [S:4679, D:20], F+P:7, G+M:1, n:4707
starling10x.4n
C:4699 [S:4679, D:20], F+P:7, G+M:1, n:4707
Size
C:4699 [S:4679, D:20], F+P:7, G+M:1, n:4707
Single
Duplicated
Fragmented
Partial
Ghost
Missing
0
20
40
60
80
100
BUSCOMP Percentage (n=4707)

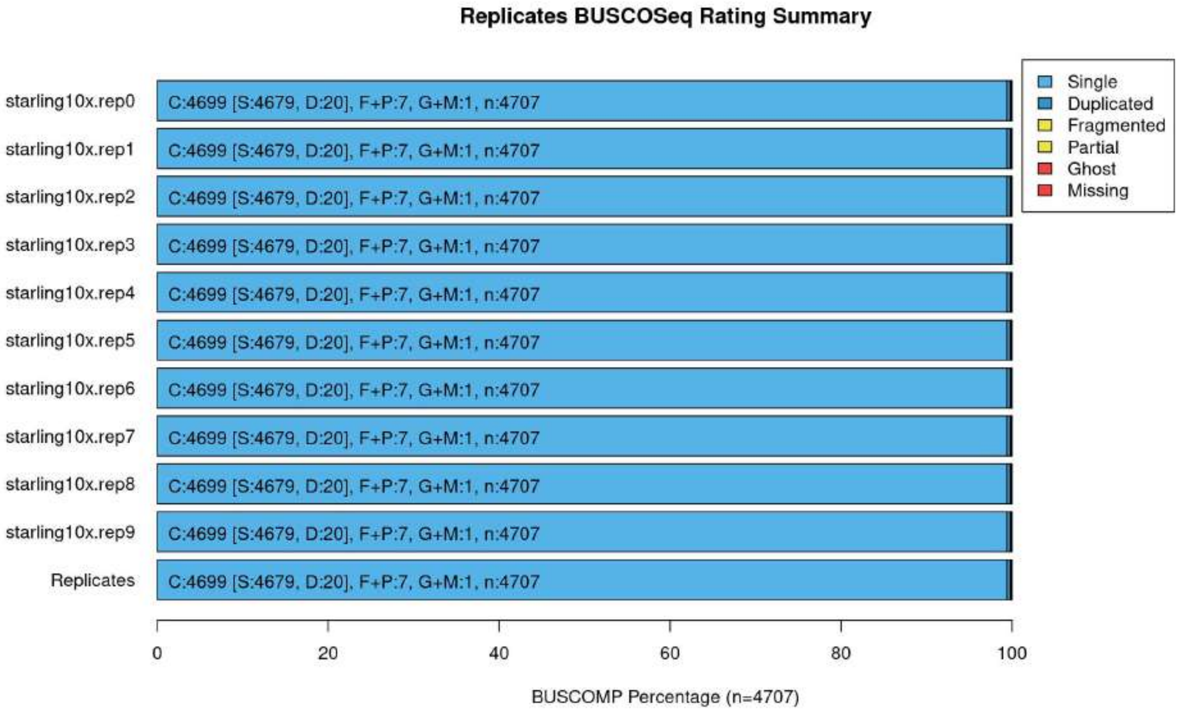
Replicates BUSCOSeq Rating Summary
starling10x.rep0
C:4699 [S:4679, D:20], F+P:7, G+M:1, n:4707
starling10x.rep1
C:4699 [S:4679, D:20], F+P:7, G+M:1, n:4707
starling10x.rep2
C:4699 [S:4679, D:20], F+P:7, G+M:1, n:4707
starling10x.rep3
C:4699 [S:4679, D:20], F+P:7, G+M:1, n:4707
starling10x.rep4
C:4699 [S:4679, D:20], F+P:7, G+M:1, n:4707
starling10x.rep5
C:4699 [S:4679, D:20], F+P:7, G+M:1, n:4707
starling10x.rep6
C:4699 [S:4679, D:20], F+P:7, G+M:1, n:4707
starling10x.rep7
C:4699 [S:4679, D:20], F+P:7, G+M:1, n:4707
starling10x.rep8
C:4699 [S:4679, D:20], F+P:7, G+M:1, n:4707
starling10x.rep9
C:4699 [S:4679, D:20], F+P:7, G+M:1, n:4707
Replicates
C:4699 [S:4679, D:20], F+P:7, G+M:1, n:4707
Single
Duplicated
Fragmented
Partial
Ghost
Missing
0
20
40
60
80
100
BUSCOMP Percentage (n=4707)

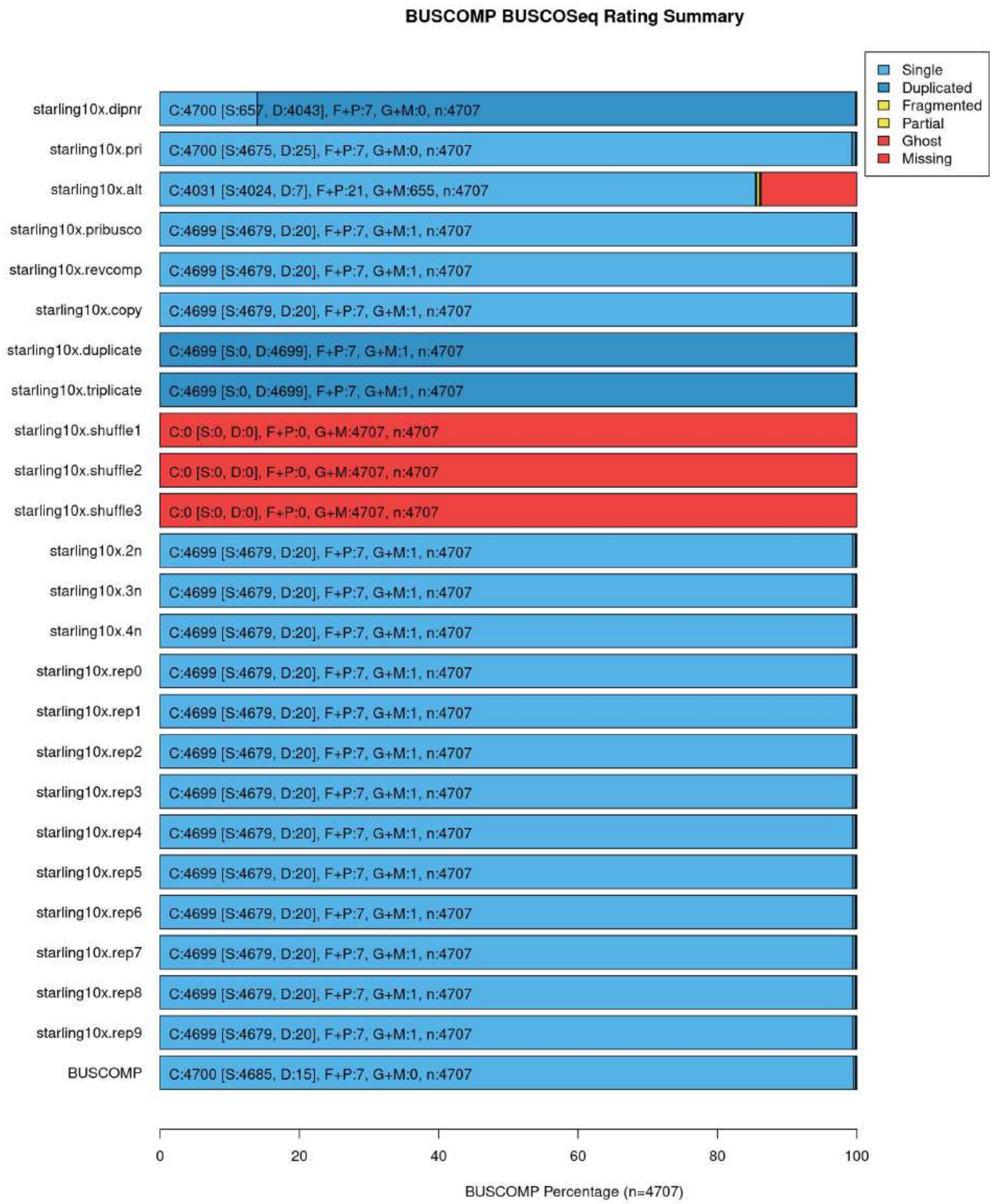

5 BUSCO and BUSCOMP Comparisons

5.1 BUSCO to BUSCOMP Rating Changes

Ratings changes from BUSCO to BUSCOMP (where `NULL` ratings indicate no BUSCOMP sequence):

BUSCO	BUSCOMP	starling10x.dipnr	starling10x.pri	starling10x.alt	starling10x.pribusco	starling10x.revcomp	starling10x.copy	starling10x.dupl
Complete	Complete	616	4450	3869	4475	4485	4475	
Complete	Duplicated	225	14	5	9	14	9	
Complete	Ghost	0	0	1	0	0	0	
Complete	Missing	0	0	64	0	0	0	
Complete	Partial	1	6	9	6	5	6	
Duplicated	Complete	7	32	15	32	22	32	
Duplicated	Duplicated	3624	9	2	10	6	10	
Duplicated	Missing	0	0	5	0	0	0	

BUSCO	BUSCOMP	starling10x.dipnr	starling10x.pri	starling10x.alt	starling10x.pribusco	starling10x.revcomp	starling10x.copy	starling10x.dupl
Duplicated	NULL	54	54	16	54	52	54	
Duplicated	Partial	5	0	0	0	0	0	
Fragmented	Complete	22	139	110	136	127	136	
Fragmented	Duplicated	116	1	0	1	0	1	
Fragmented	Missing	0	0	5	0	0	0	
Fragmented	NULL	73	78	43	79	76	79	
Fragmented	Partial	1	1	1	1	1	1	
Missing	Complete	12	54	30	36	45	36	
Missing	Duplicated	78	1	0	0	0	0	
Missing	Ghost	0	0	7	0	0	0	
Missing	Missing	0	0	573	1	1	1	
Missing	NULL	81	76	149	75	80	75	
Missing	Partial	0	0	11	0	1	0	

Full table of Ratings changes from by gene:

BuscoID <chr>	starling10x.dipnr <chr>	starling10x.pri <chr>
EOG090F001C	DD	CC
EOG090F0023	DD	CC
EOG090F0028	DD	CC
EOG090F002E	DD	CC
EOG090F0034	MN	MN
EOG090F003F	DD	CC
EOG090F003R	DD	CC
EOG090F0040	DD	CC
EOG090F0042	DD	CC
EOG090F0043	DD	CC

1-10 of 4,915 rows | 1-3 of 25 columns Previous 1 2 3 4 5 6 ... 492 Next

Complete, **D**uplicated, **F**ragmented, **P**artial, **G**host, **M**issing, **N**ULL (no BUSCOMP sequence)

5.1.1 BUSCOMP Gain test

There is a risk that performing a low stringency search will identify homologues or pseudogenes of the desired BUSCO gene in error. If there is a second copy of a gene in the genome that is detectable by the search then we would expect the same genes that go from `Missing` to `Complete` in some genomes to go from `Single` to `Duplicated` in others.

To test this, data is reduced for each pair of genomes to BUSCO-BUSCOMP rating pairs of:

- `Single` - `Single`
- `Single` - `Duplicated`
- `Missing` - `Missing`
- `Missing` - `Single`

This is then converted in to `G` ain ratings (`Single` - `Duplicated` & `Missing` - `Single`) or `N` o Gain ratings (`Single` - `Single` & `Missing` - `Missing`). The `Single` - `Duplicated` shift in one genome is then used to set the expected `Missing` - `Single` shift in the other, and assess the probability of observing the `Missing` - `Single` shift using a cumulative binomial distribution, where:

- `k` is the number of observed `GG` pairs (`Single` - `Duplicated` *and* `Missing` - `Single`)
- `n` is the number of `Missing` - `Single` `G` ains in the focal genome (`NG` + `GG`)
- `p` is the proportion of `Single` - `Duplicated` `G` ains in the background genome (`GN` + `GG` / (`GN` + `GG` + `NN` + `NG`))
- `pB` is the probability of observing `k+` `Missing` - `Single` gains, given `p` and `n`

This is output to `*.gain.tdt` , where each row is a Genome and each field gives the probability of the row genome's `Missing` - `Single` gains, given the column genome's `Single` - `Duplicated` gains:

Genome <chr>	starling10x.dipnr <dbl>
starling10x.2n	7.38e-01
starling10x.3n	6.83e-01
starling10x.4n	6.83e-01
starling10x.alt	1.16e-07
starling10x.copy	7.38e-01

Genome <chr>	starling10x.dipnr <dbl>
starling10x.dipnr	1.00e+00
starling10x.duplicate	1.00e+00
starling10x.pri	7.38e-01
starling10x.pribusco	7.38e-01
starling10x.rep0	7.38e-01

1-10 of 24 rows | 1-2 of 25 columns Previous 1 2 3 Next

Low probabilities indicate that BUSCOMP might be rating paralogues or pseudogenes and not functional orthologues of the BUSCO gene. Note that there is *no* correction for multiple testing, nor any adjustment for lack of independence between samples.

5.2 Unique BUSCO and BUSCOMP Complete Genes

BUSCO and BUSCOMP `Complete` ratings were compared for each BUSCO gene to identify those genes unique to either a single assembly or a group of assemblies. The `BUSCOMP` group is excluded from this analysis, as (typically) are other redundant groups wholly contained within another group. (Inclusion of such groups is guaranteed to result in 2+ groups containing any `Complete` BUSCOs they have.)

```
starling10x.dipnr unique Complete genes: 0 BUSCO; 0 BUSCOMP
starling10x.pri unique Complete genes: 0 BUSCO; 0 BUSCOMP
starling10x.alt unique Complete genes: 4 BUSCO; 0 BUSCOMP
starling10x.pribusco unique Complete genes: 0 BUSCO; 0 BUSCOMP
starling10x.revcomp unique Complete genes: 3 BUSCO; 0 BUSCOMP
starling10x.copy unique Complete genes: 0 BUSCO; 0 BUSCOMP
starling10x.duplicate unique Complete genes: 2 BUSCO; 0 BUSCOMP
starling10x.triplicate unique Complete genes: 2 BUSCO; 0 BUSCOMP
starling10x.shuffle1 unique Complete genes: 0 BUSCO; 0 BUSCOMP
starling10x.shuffle2 unique Complete genes: 0 BUSCO; 0 BUSCOMP
starling10x.shuffle3 unique Complete genes: 0 BUSCO; 0 BUSCOMP
starling10x.2n unique Complete genes: 0 BUSCO; 0 BUSCOMP
starling10x.3n unique Complete genes: 0 BUSCO; 0 BUSCOMP
starling10x.4n unique Complete genes: 0 BUSCO; 0 BUSCOMP
starling10x.rep0 unique Complete genes: 0 BUSCO; 0 BUSCOMP
starling10x.rep1 unique Complete genes: 0 BUSCO; 0 BUSCOMP
starling10x.rep2 unique Complete genes: 0 BUSCO; 0 BUSCOMP
starling10x.rep3 unique Complete genes: 0 BUSCO; 0 BUSCOMP
starling10x.rep4 unique Complete genes: 0 BUSCO; 0 BUSCOMP
starling10x.rep5 unique Complete genes: 0 BUSCO; 0 BUSCOMP
starling10x.rep6 unique Complete genes: 0 BUSCO; 0 BUSCOMP
starling10x.rep7 unique Complete genes: 0 BUSCO; 0 BUSCOMP
starling10x.rep8 unique Complete genes: 0 BUSCO; 0 BUSCOMP
starling10x.rep9 unique Complete genes: 0 BUSCO; 0 BUSCOMP
Core unique Complete genes: 0 BUSCO; 0 BUSCOMP
Duplication unique Complete genes: 0 BUSCO; 0 BUSCOMP
Pseudodip unique Complete genes: 0 BUSCO; 0 BUSCOMP
Replicates unique Complete genes: 0 BUSCO; 0 BUSCOMP
Size unique Complete genes: 0 BUSCO; 0 BUSCOMP
```

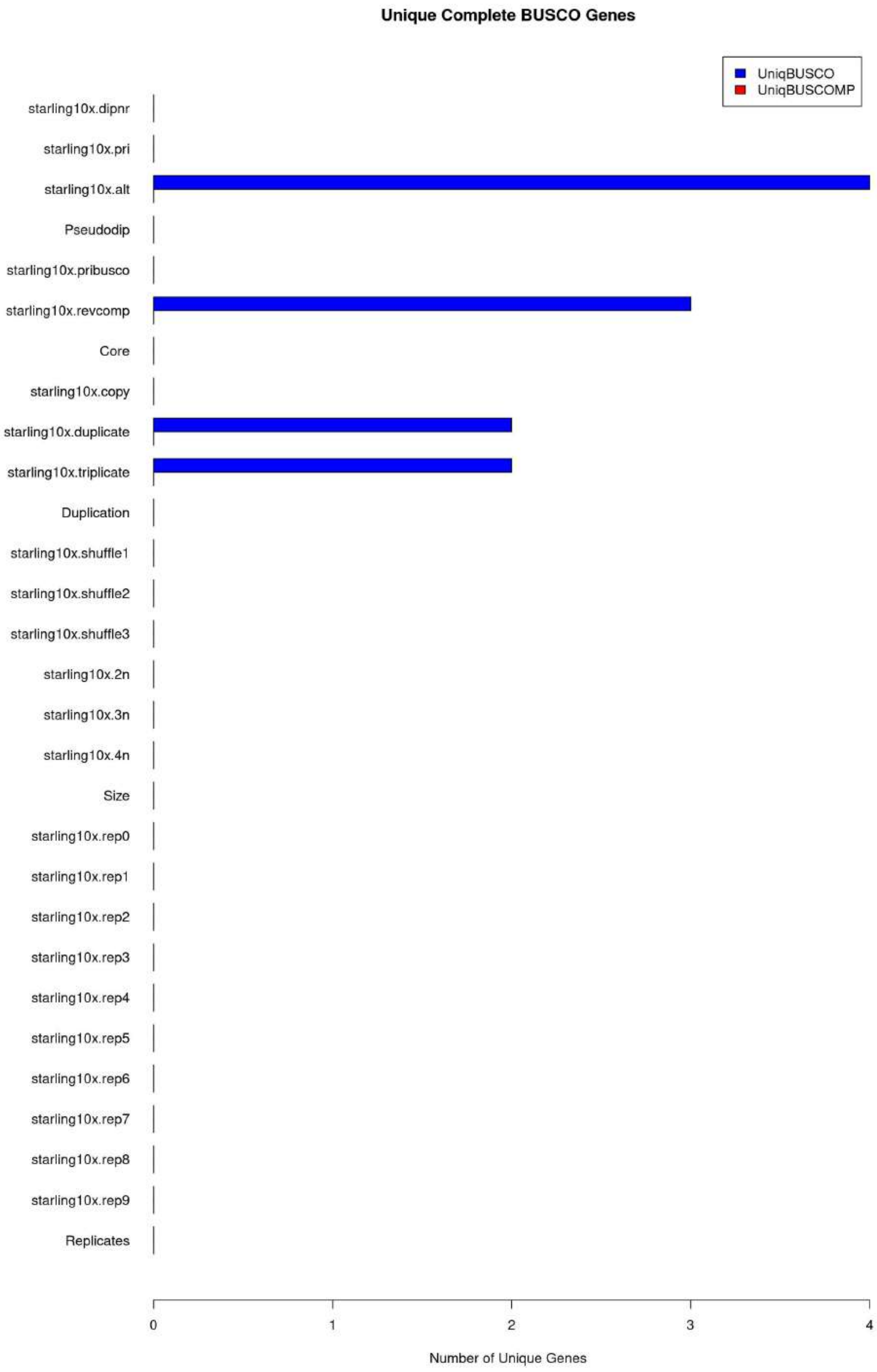

5.3 Ratings for Missing BUSCO genes

In addition to the unique ratings (above), it can be useful to know how genes `Missing` from one assembly/group are rated in the others. These plots are generated for each assembly/group in turn. The full BUSCO (`*.busco.tdt`) and BUSCOMP (`*.LnnIDxx.buscomp.tdt`) tables are reduced to the subset of genes that are missing in the assembly/group of interest, and then the summary ratings recalculated for that subset.

In each case, three plots are made (assuming both BUSCO and BUSCOMP data is available):

1. BUSCO ratings for missing BUSCO genes.

2. BUSCOMP ratings for missing BUSCO genes. As well as being more relaxed than pure BUSCO results, this will indicate when BUSCOMP has found a gene in the focal assembly/group where BUSCO did not.
3. BUSCOMP ratings for missing BUSCOMP genes. It is expected that assemblies will be much more similar in terms of BUSCOMP coverage.

5.4 Missing starling10x.dipnr BUSCO genes

BUSCO ratings for `Missing` starling10x.dipnr BUSCO genes:

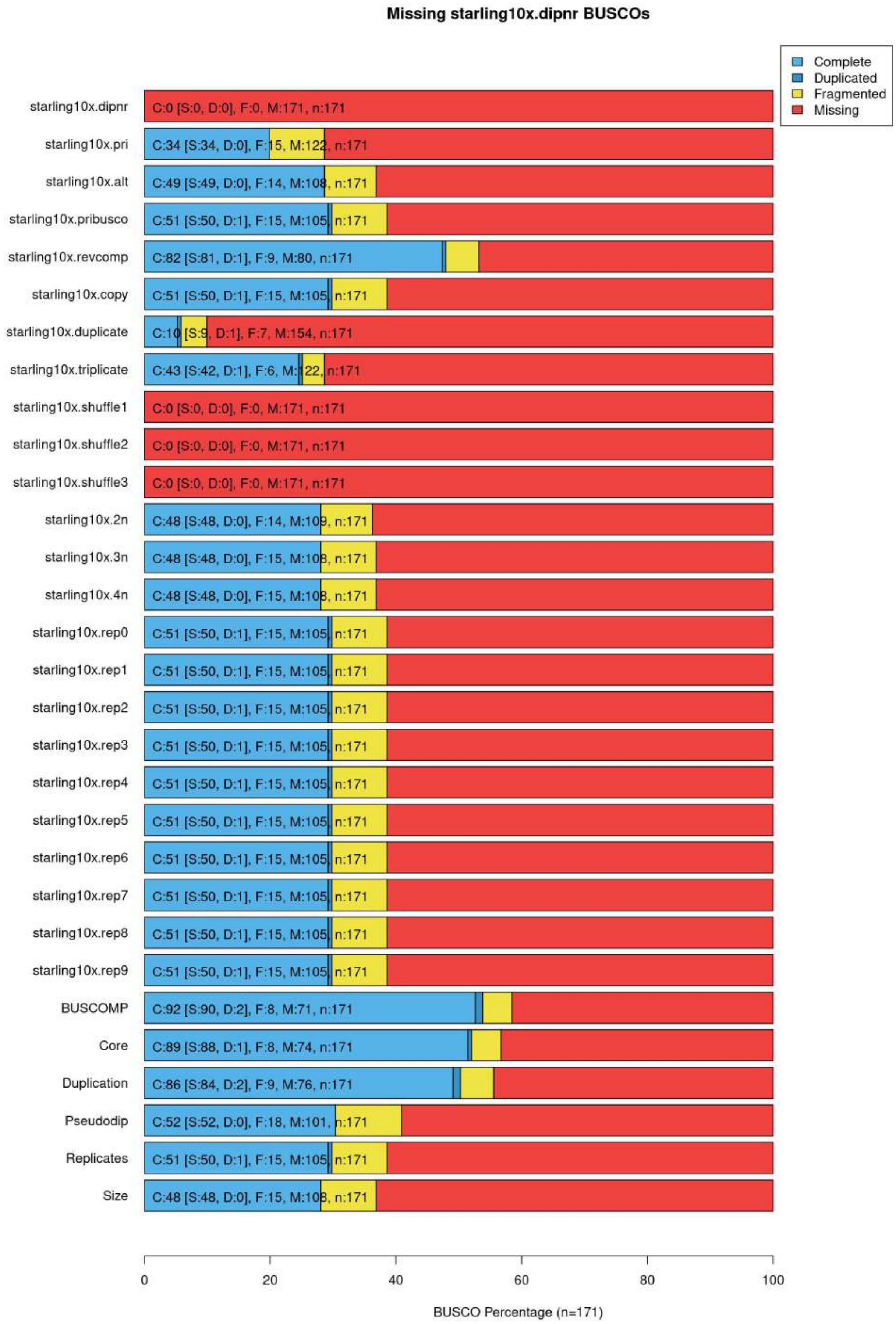

BUSCOMP ratings for `Missing` starling10x.dipnr BUSCO genes:

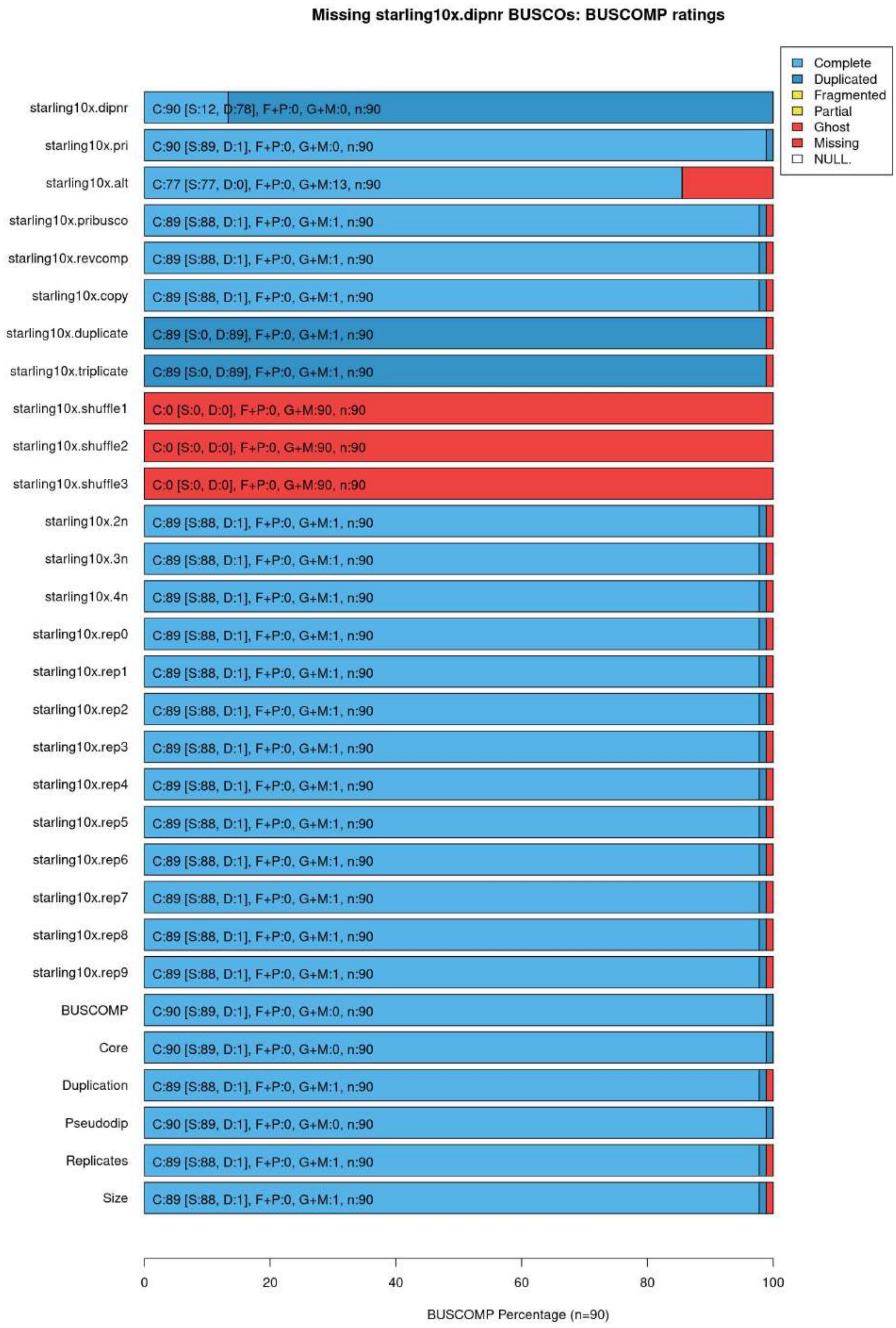

BUSCOMP ratings for `Missing` starling10x.dipnr BUSCOMP genes:

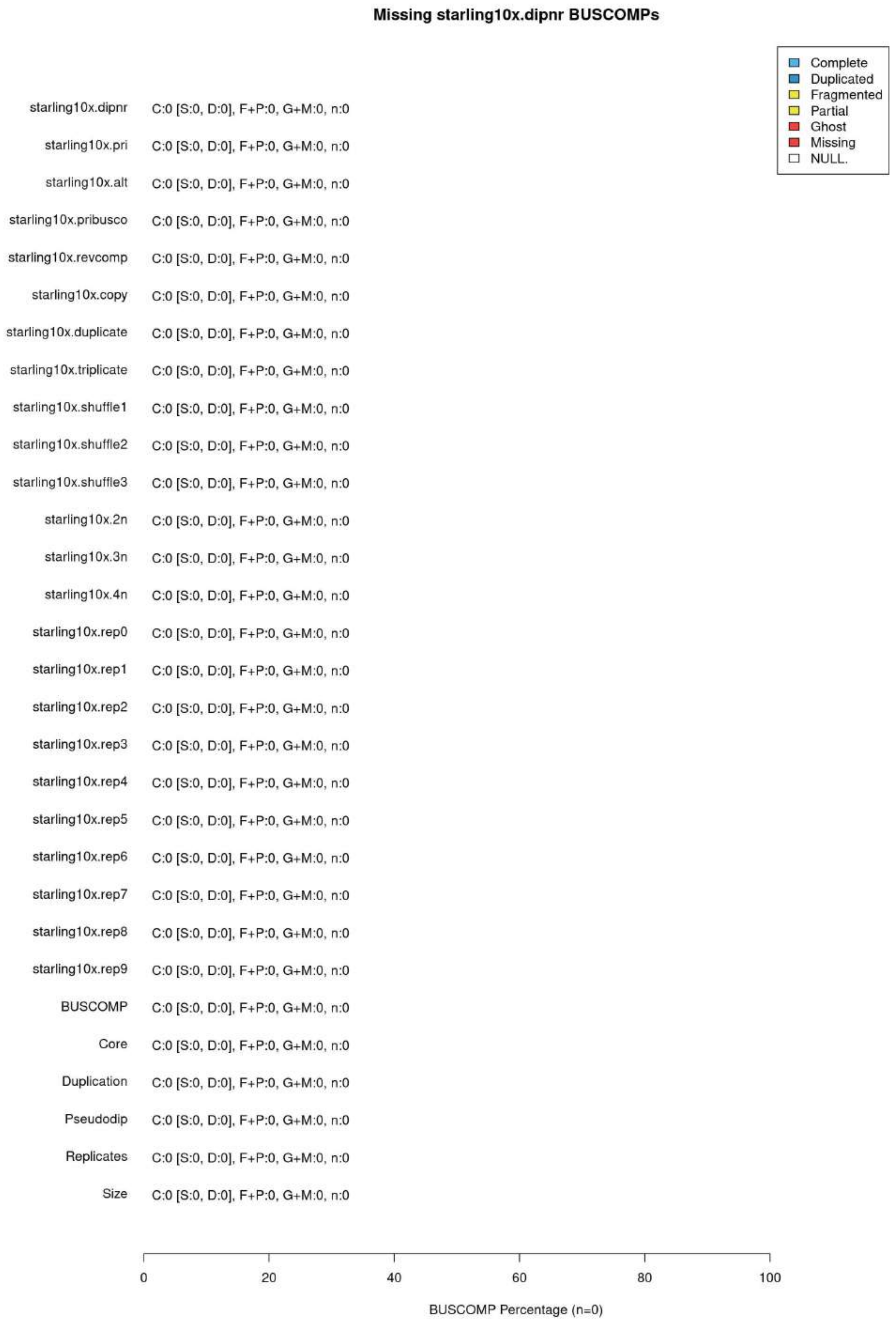

5.5 Missing starling10x.pri BUSCO genes

BUSCO ratings for `Missing` starling10x.pri BUSCO genes:

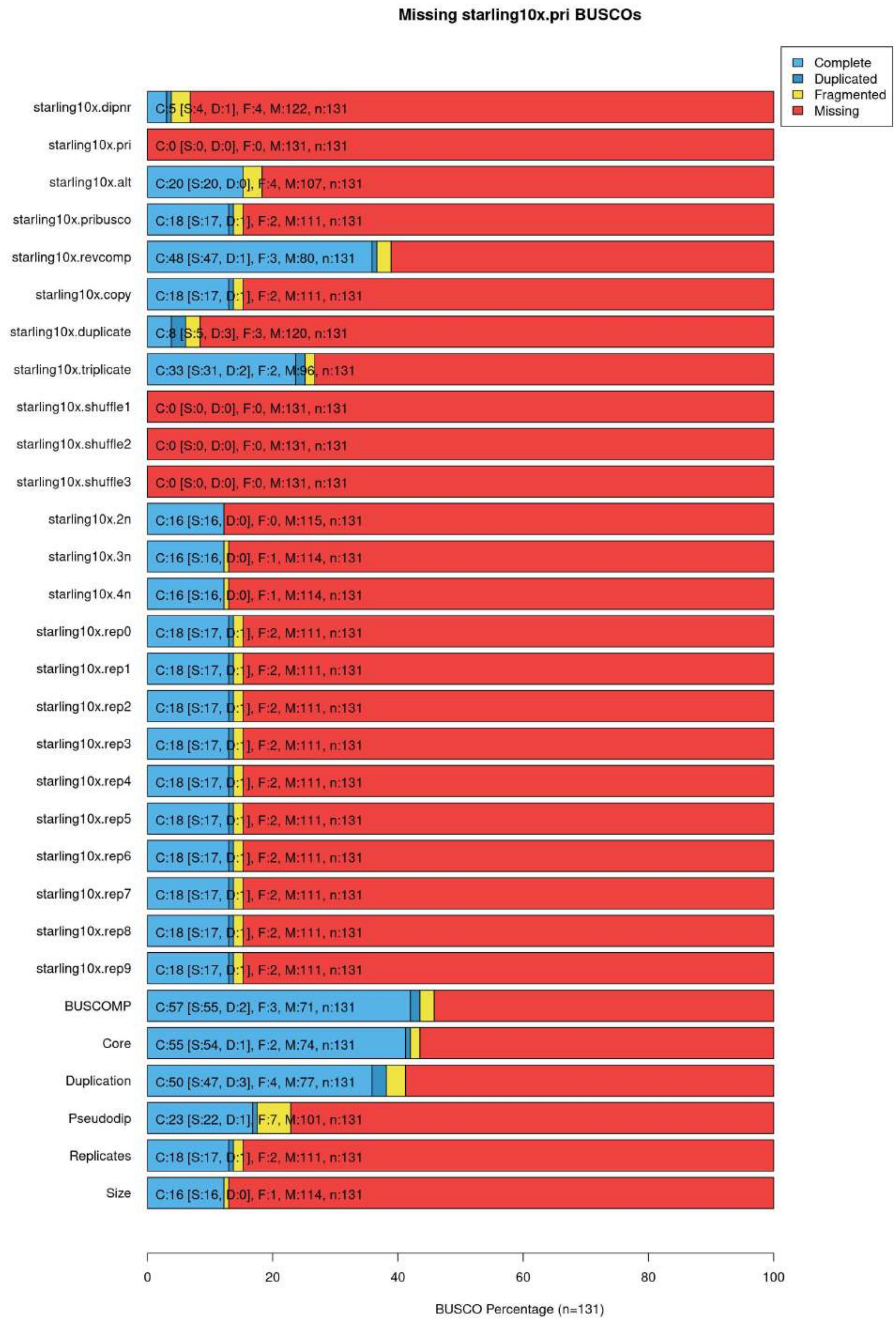

BUSCOMP ratings for `Missing` starling10x.pri BUSCO genes:

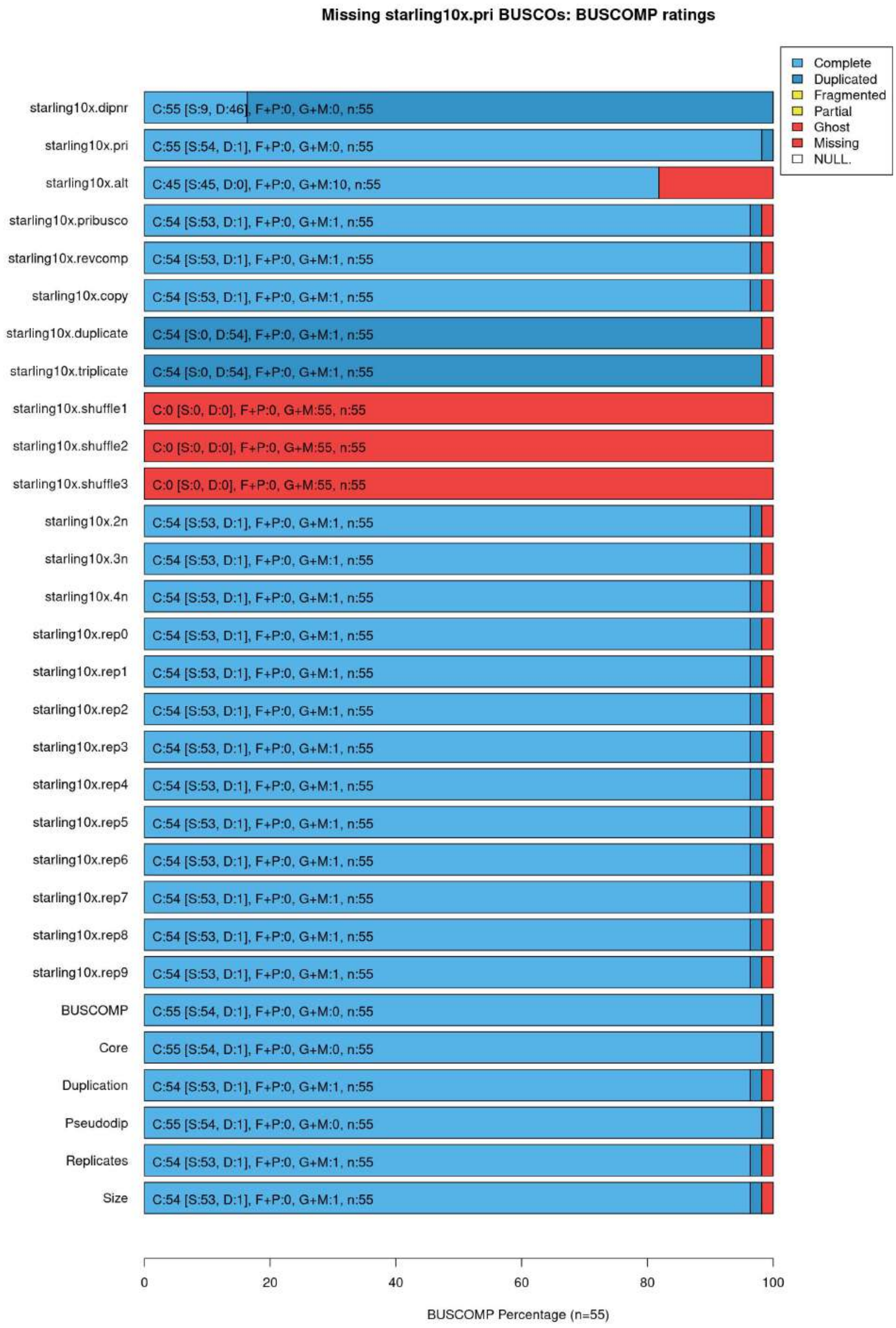

BUSCOMP ratings for `Missing` starling10x.pri BUSCOMP genes:

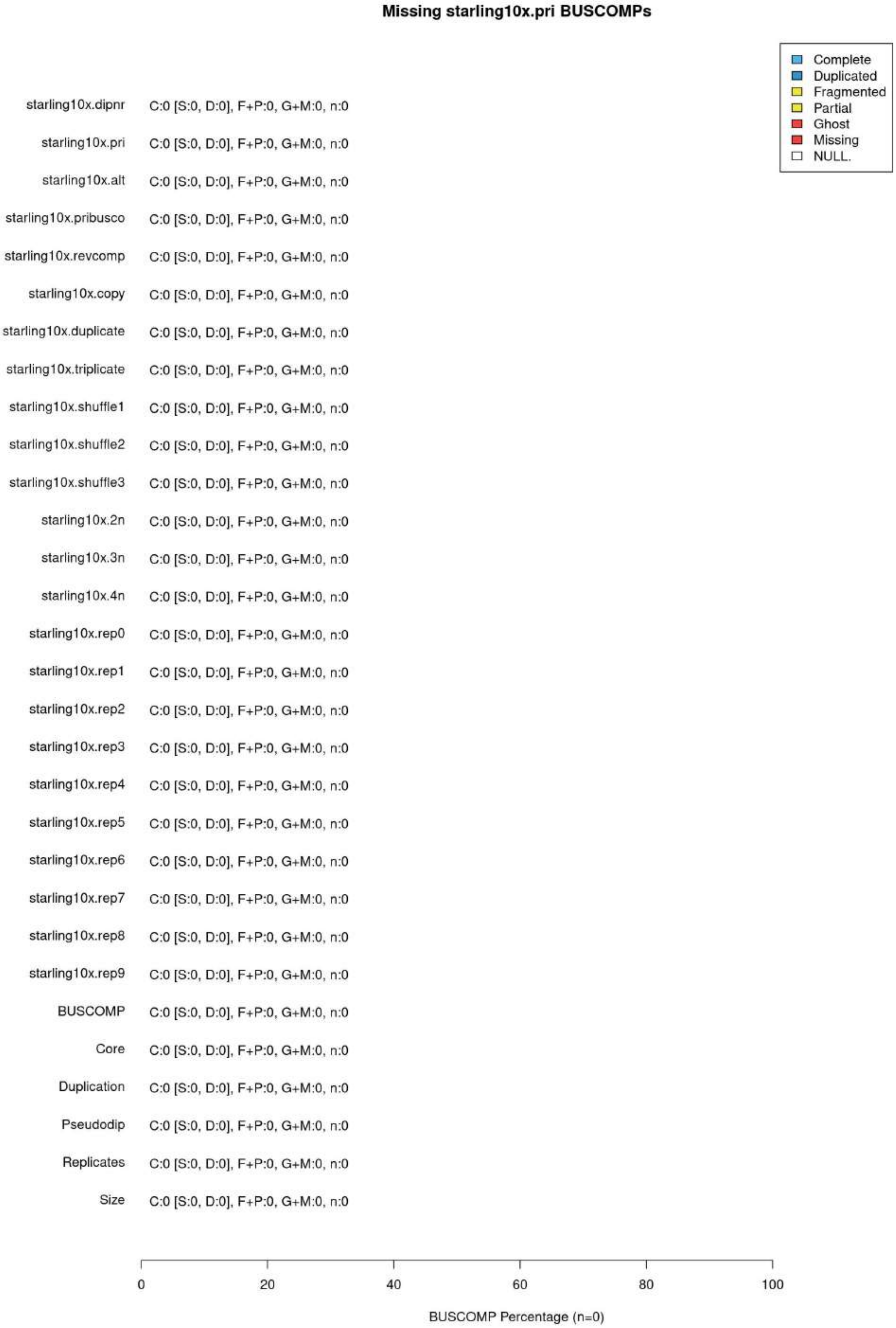

5.6 Missing starling10x.alt BUSCO genes

BUSCO ratings for `Missing` starling10x.alt BUSCO genes:

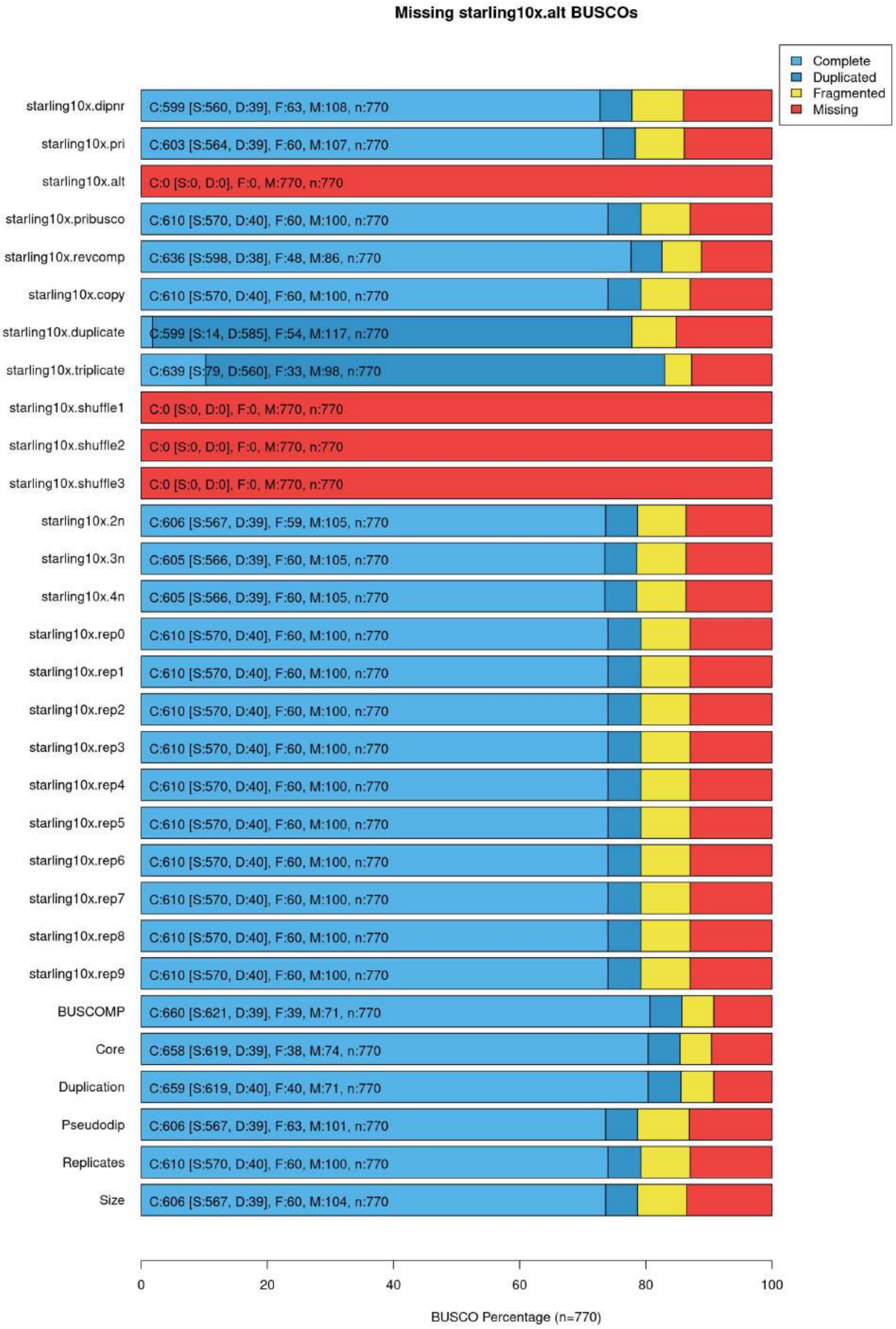

BUSCOMP ratings for `Missing` starling10x.alt BUSCO genes:

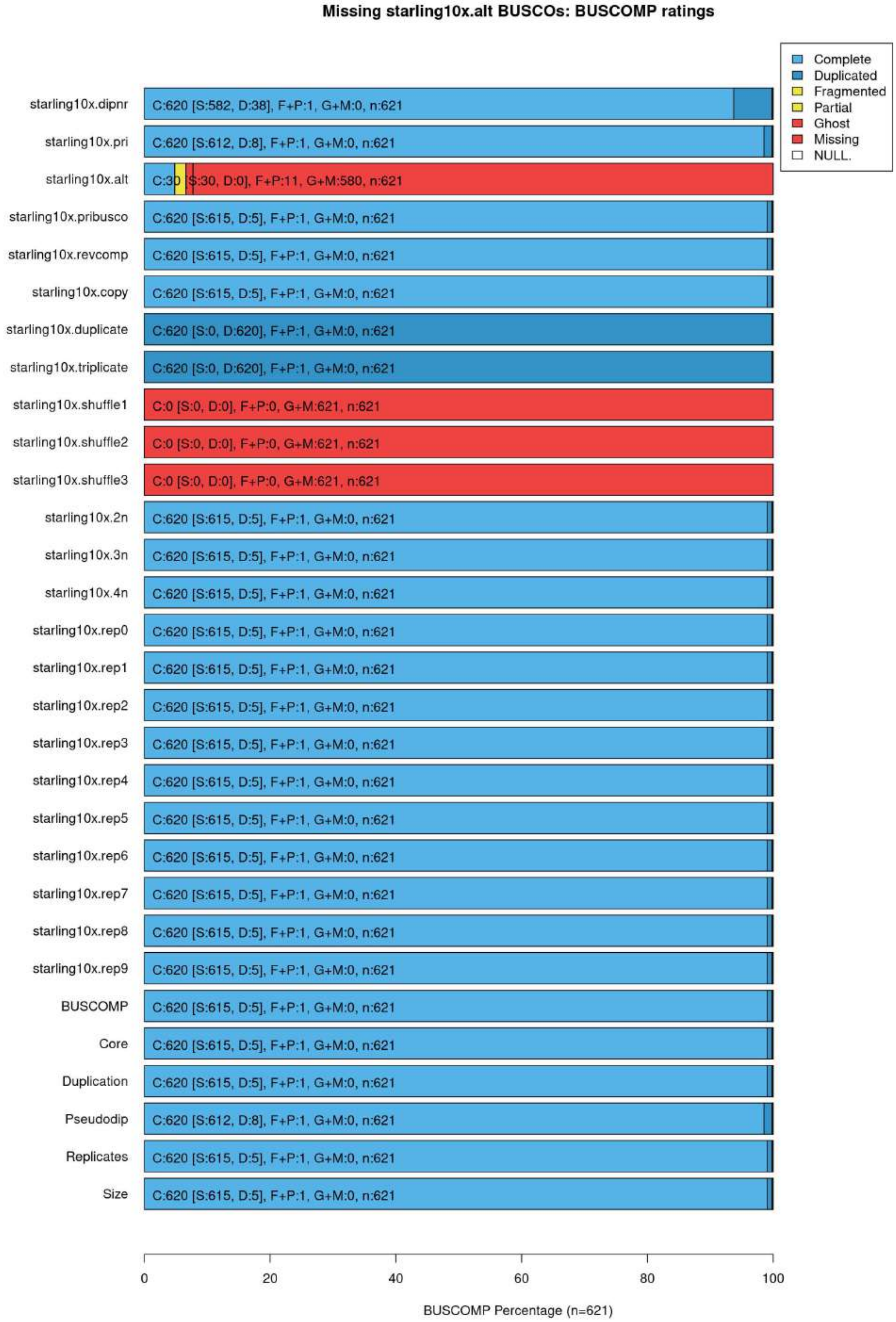

BUSCOMP ratings for `Missing` starling10x.alt BUSCOMP genes:

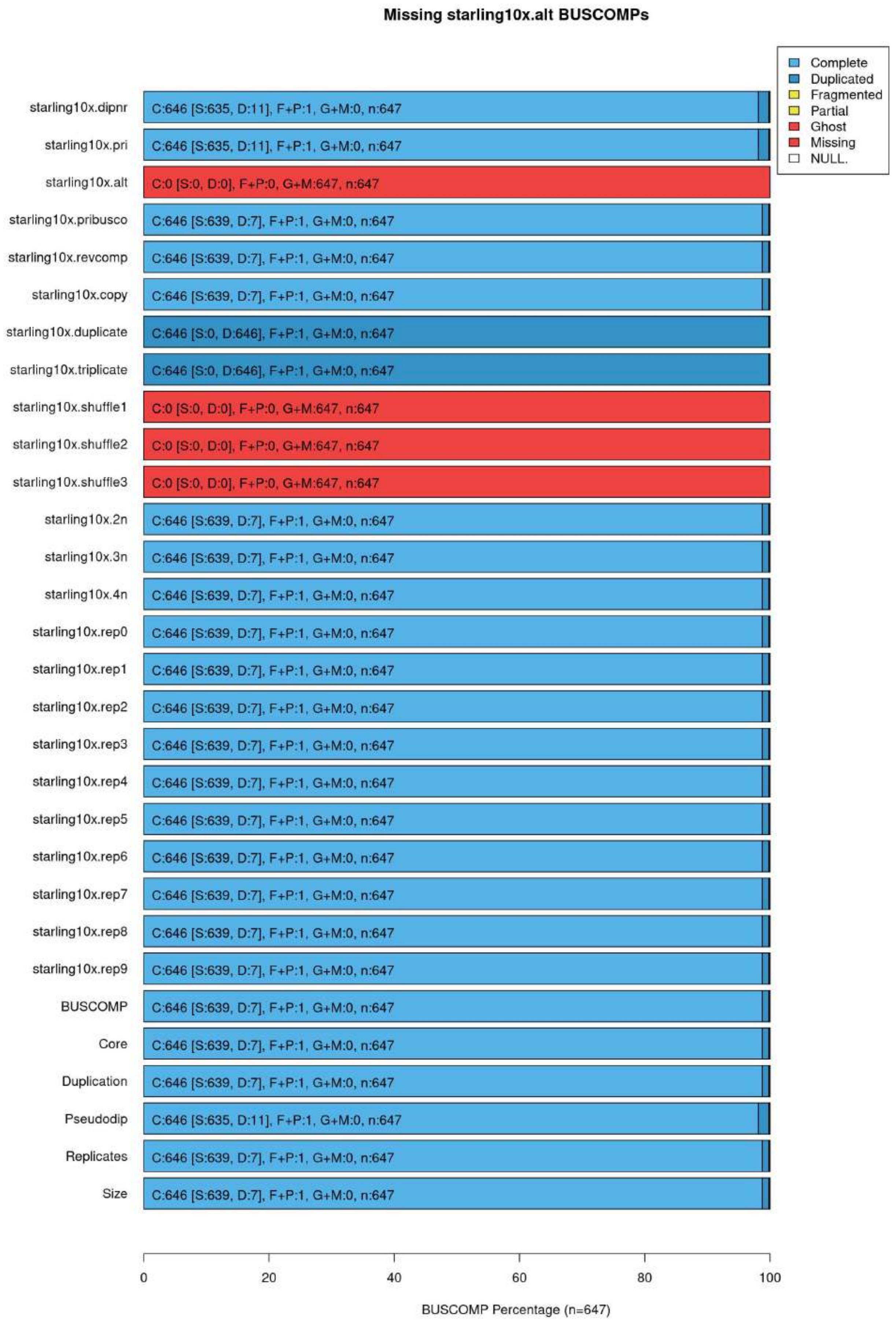

5.7 Missing Pseudodip BUSCO genes

BUSCO ratings for `Missing` Pseudodip BUSCO genes:

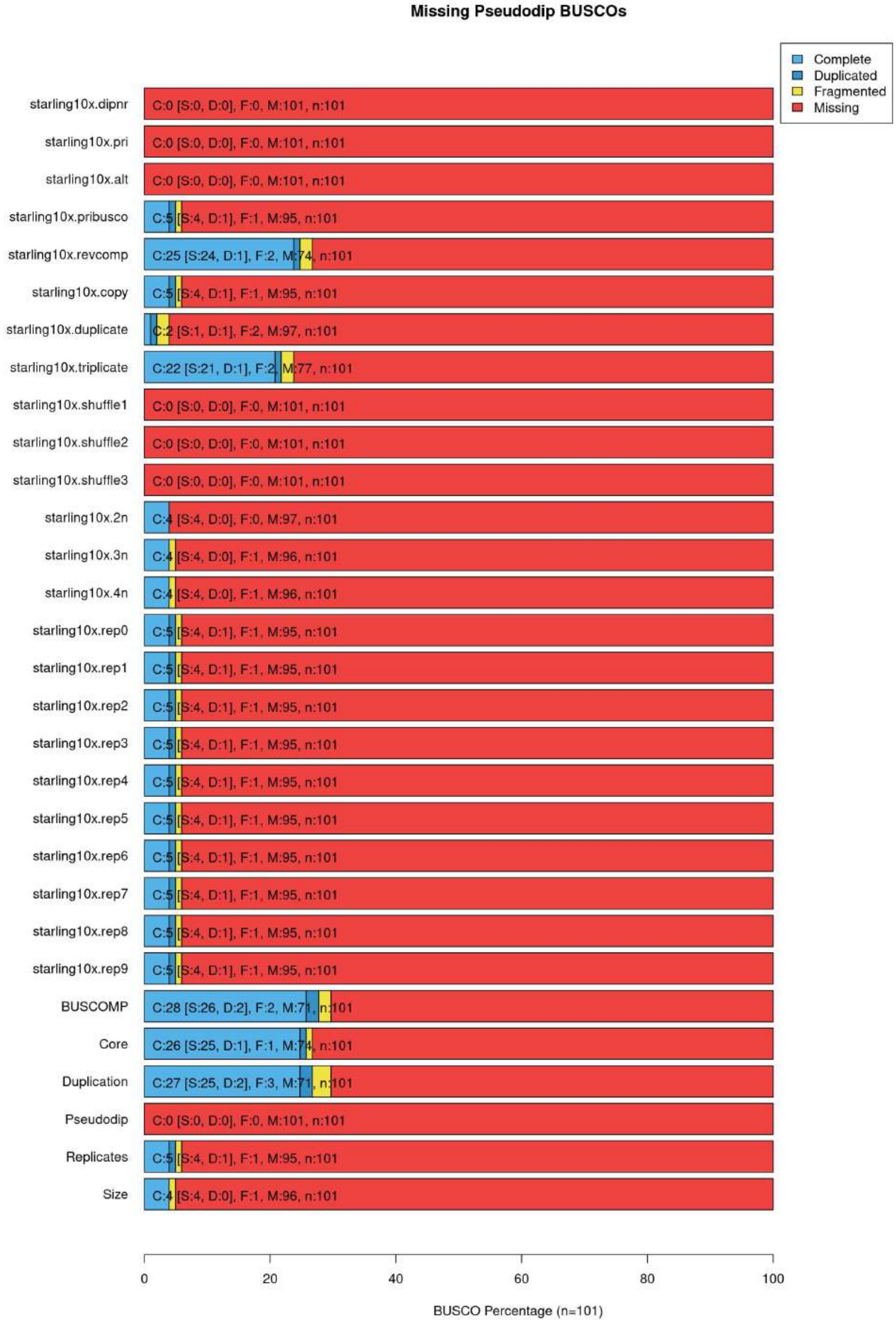

BUSCOMP ratings for `Missing` Pseudodip BUSCO genes:

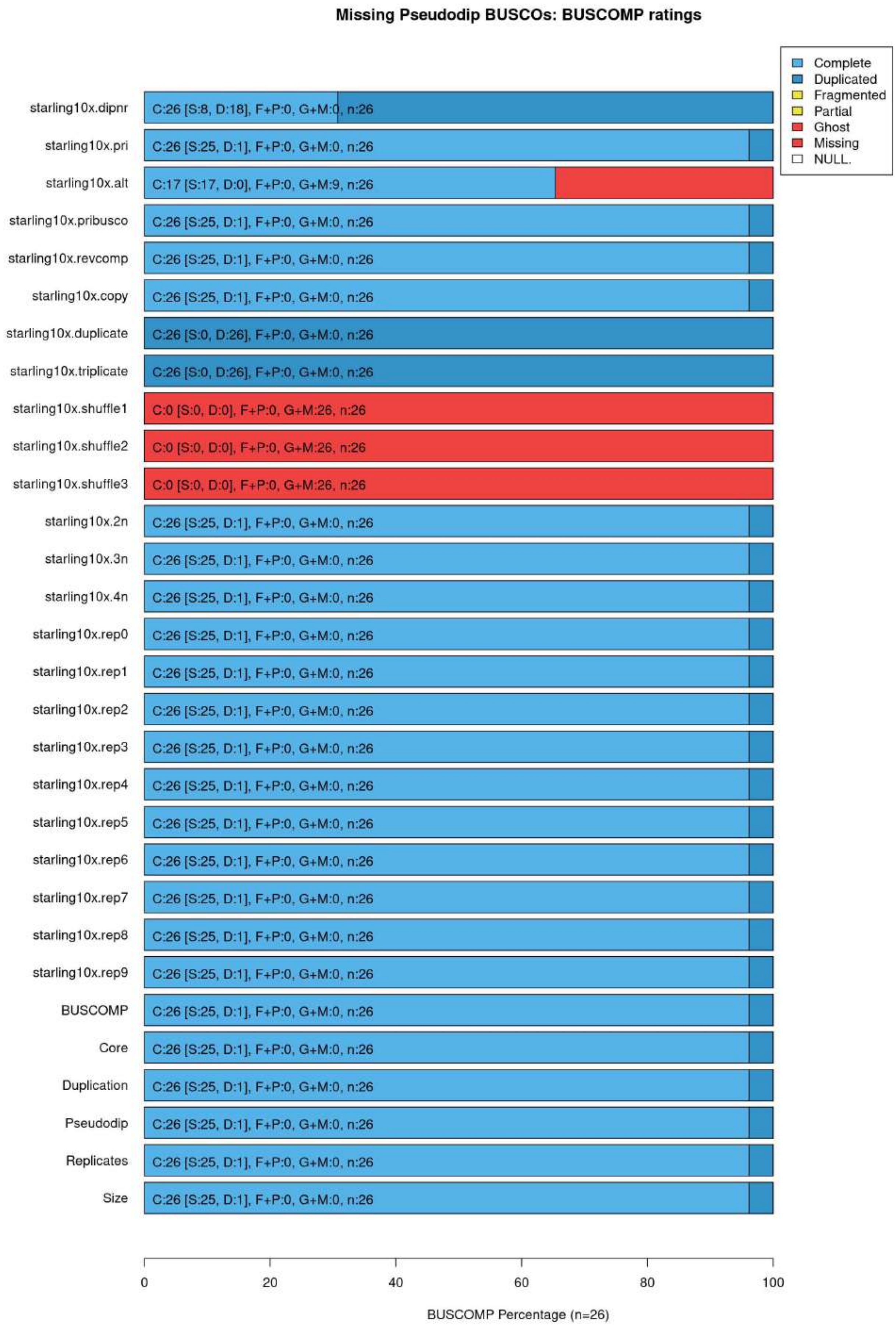

BUSCOMP ratings for `Missing` Pseudodip BUSCOMP genes:

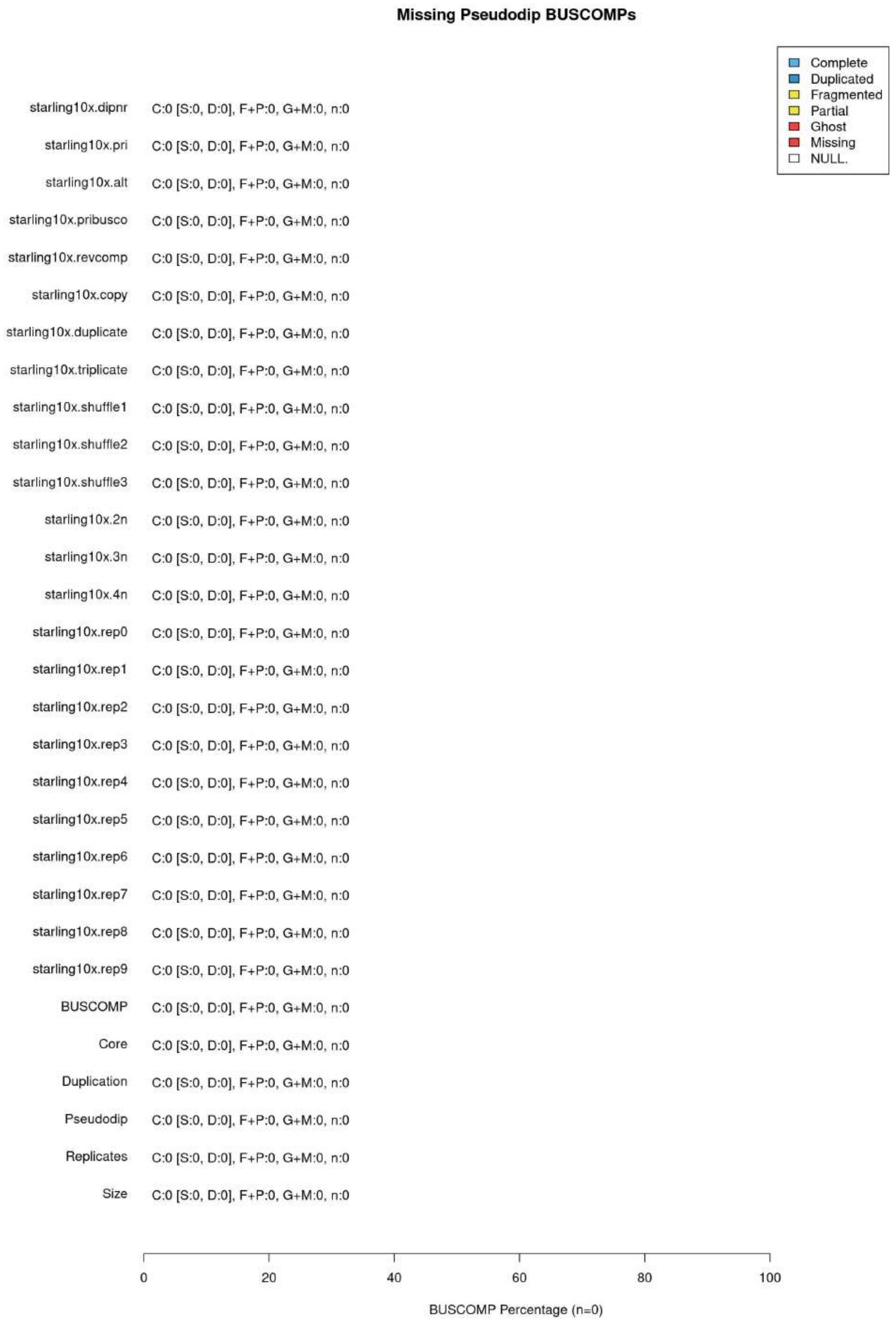

5.8 Missing starling10x.pribusco BUSCO genes

BUSCO ratings for `Missing` starling10x.pribusco BUSCO genes:

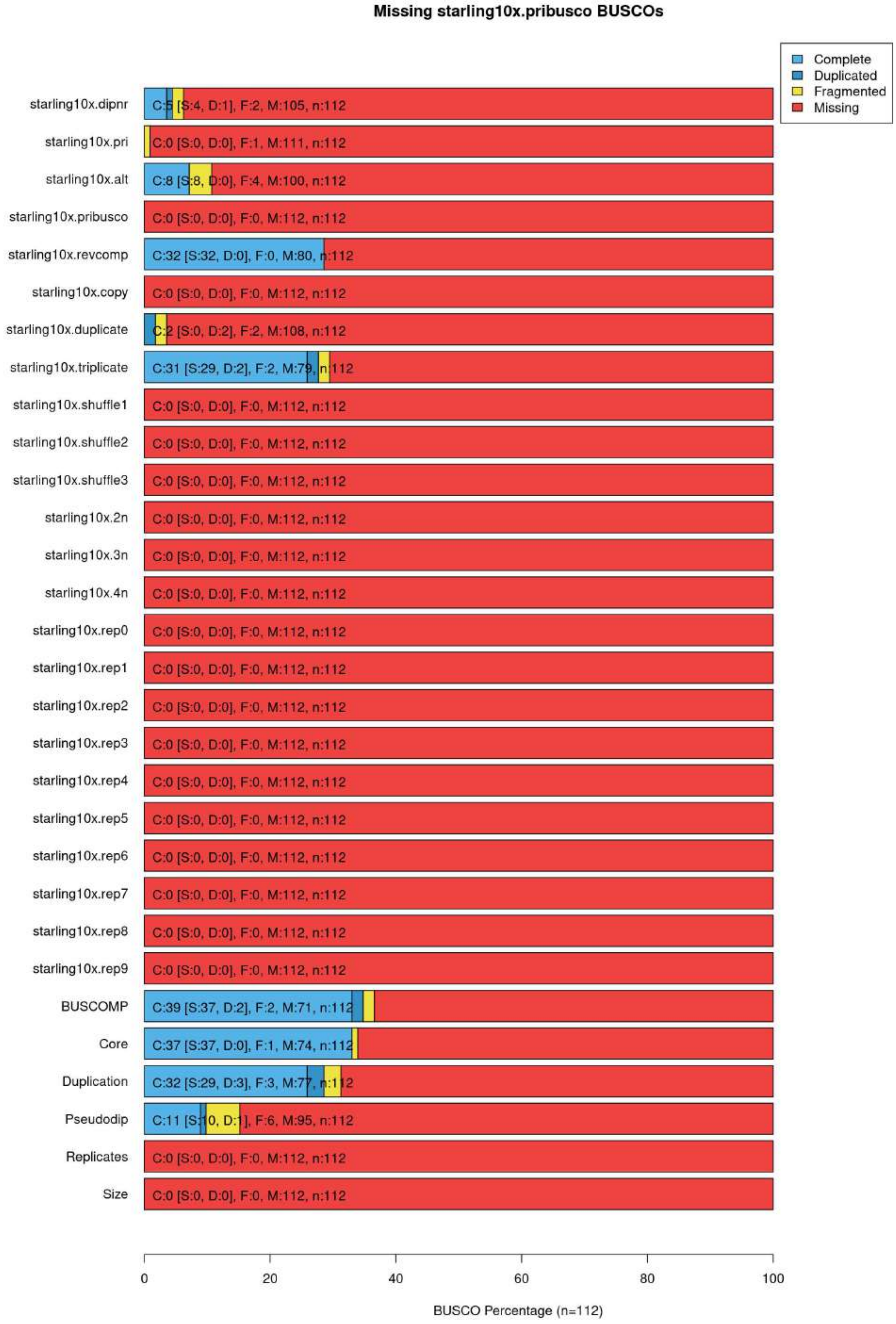

BUSCOMP ratings for `Missing` starling10x.pribusco BUSCO genes:

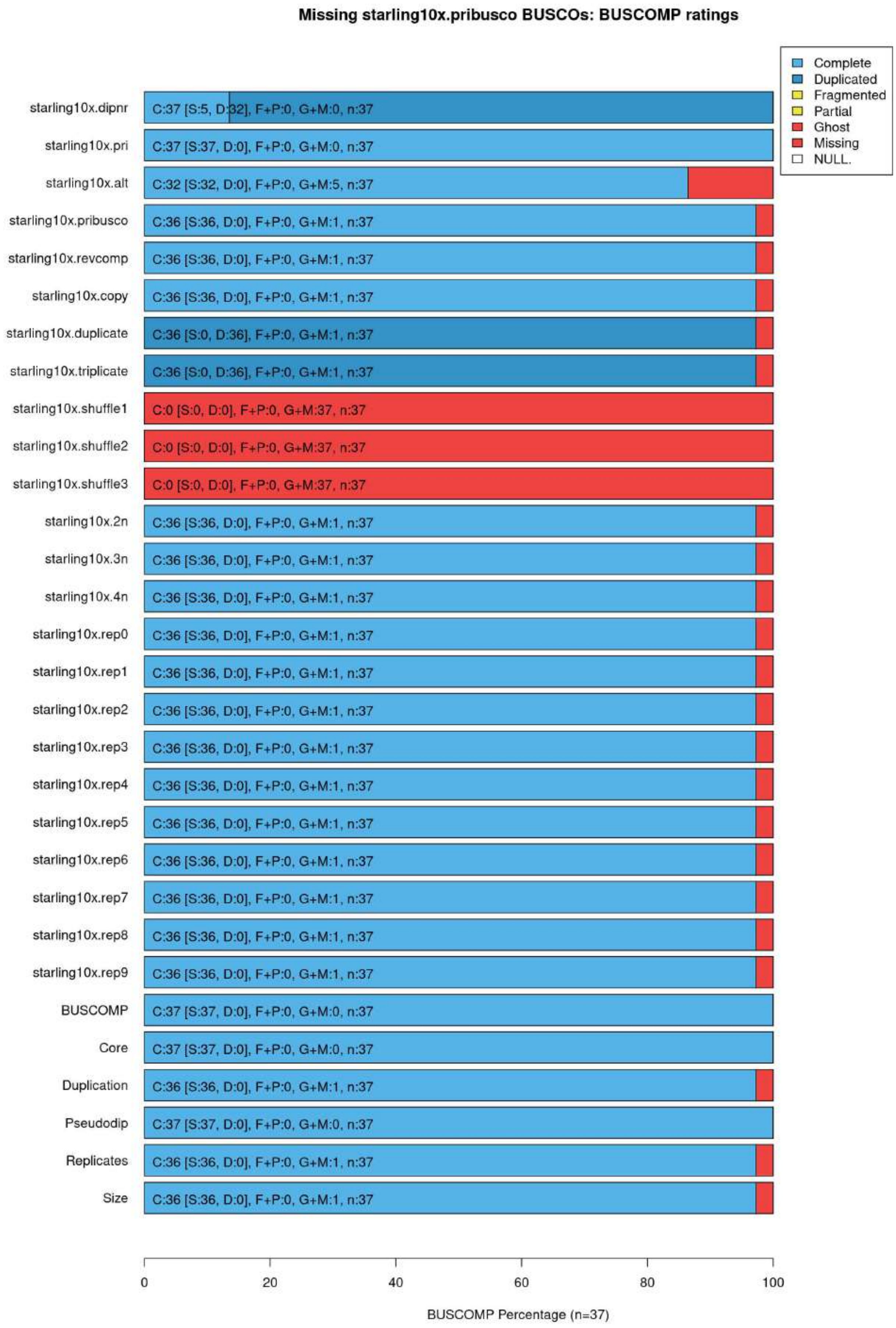

BUSCOMP ratings for `Missing` starling10x.pribusco BUSCOMP genes:

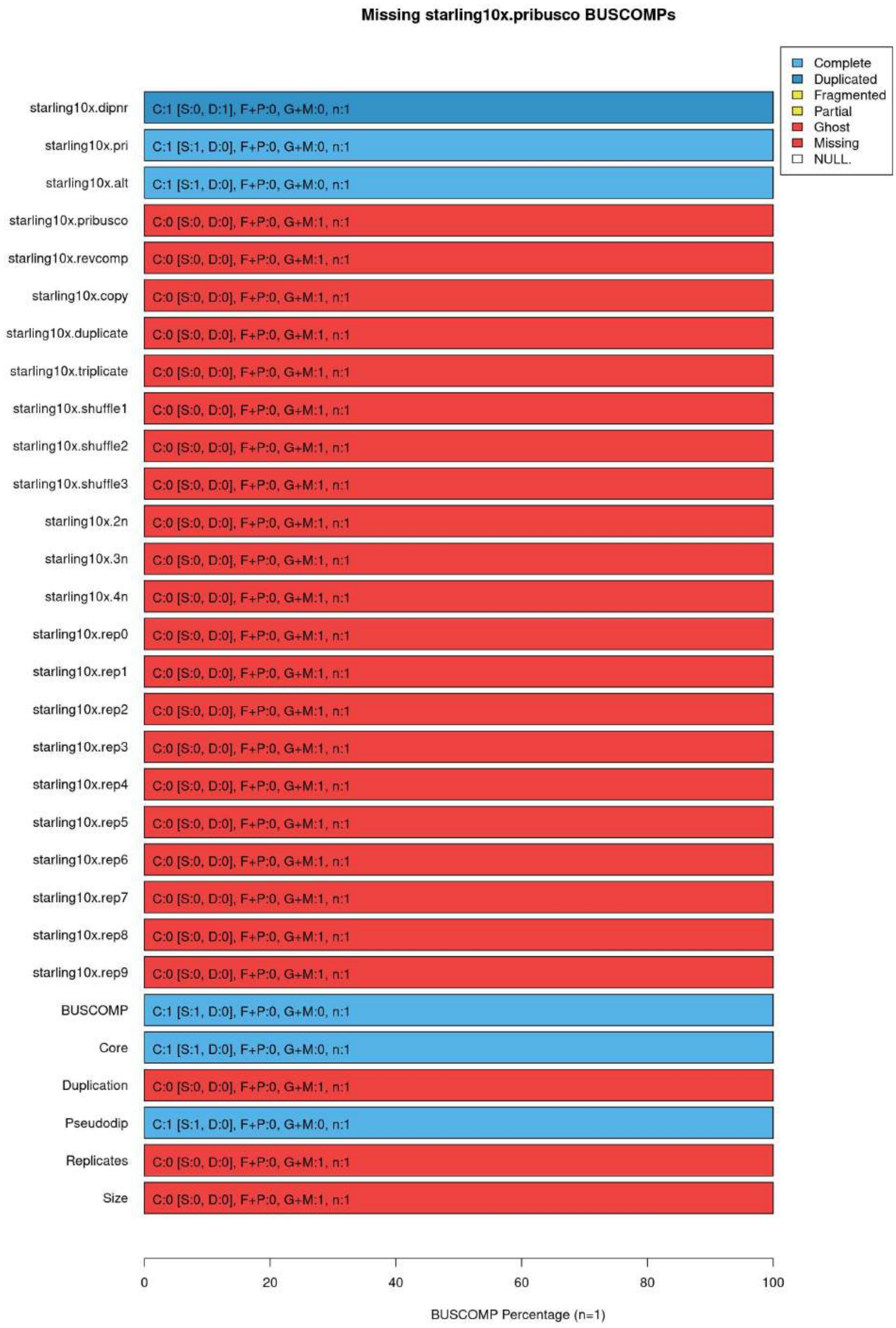

5.9 Missing starling10x.revcomp BUSCO genes

BUSCO ratings for `Missing` starling10x.revcomp BUSCO genes:

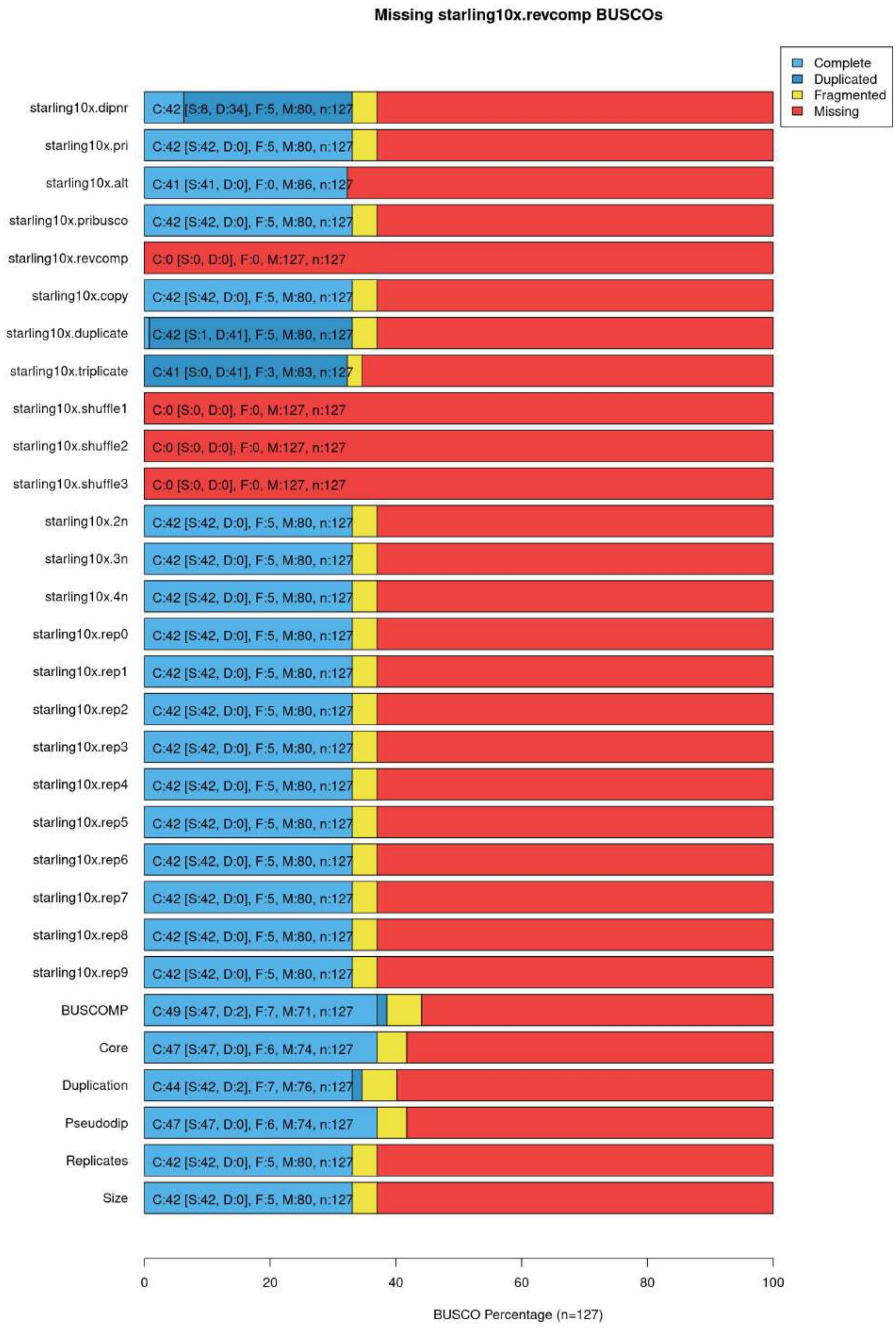

BUSCOMP ratings for `Missing` starling10x.revcomp BUSCO genes:

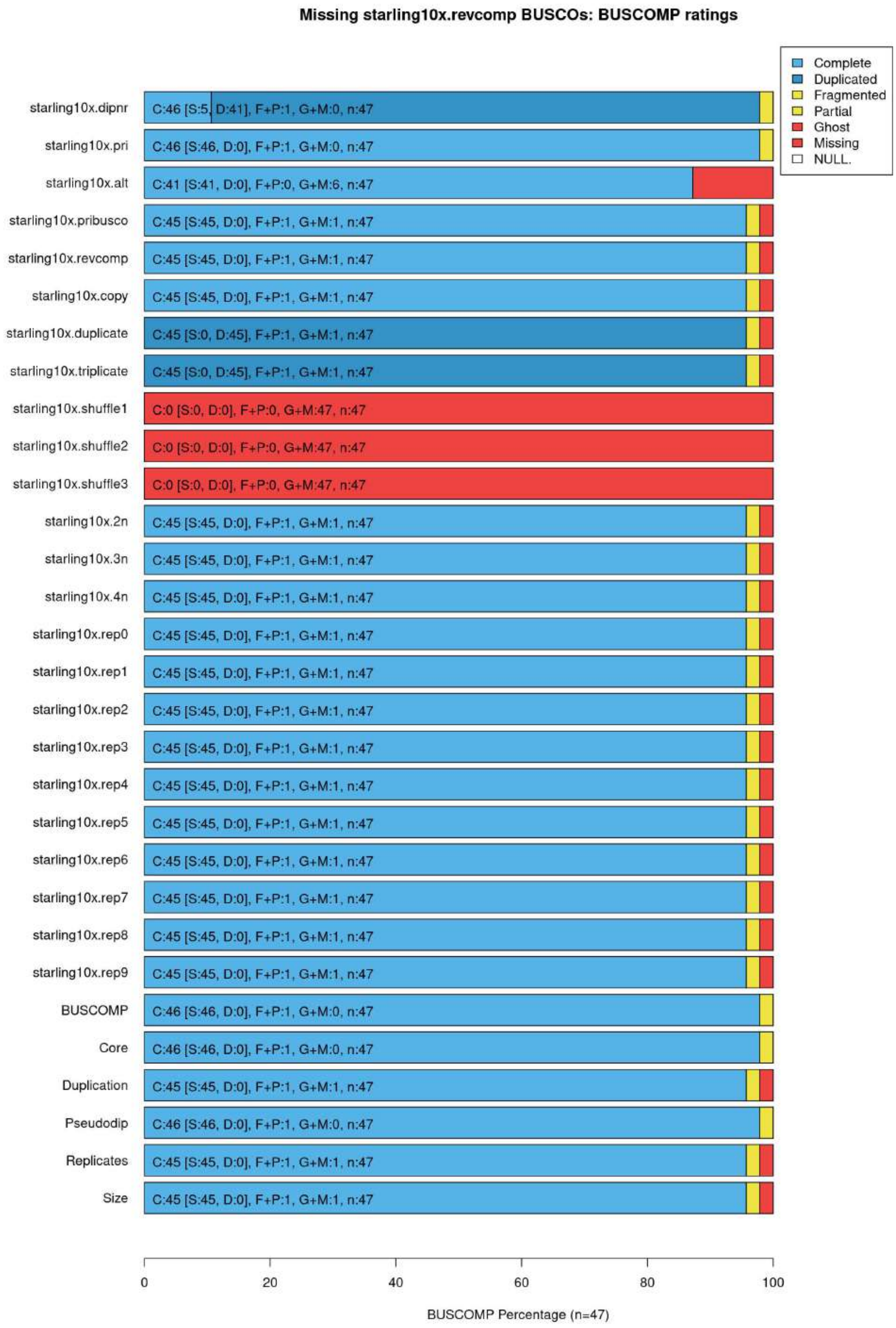

BUSCOMP ratings for `Missing` starling10x.revcomp BUSCOMP genes:

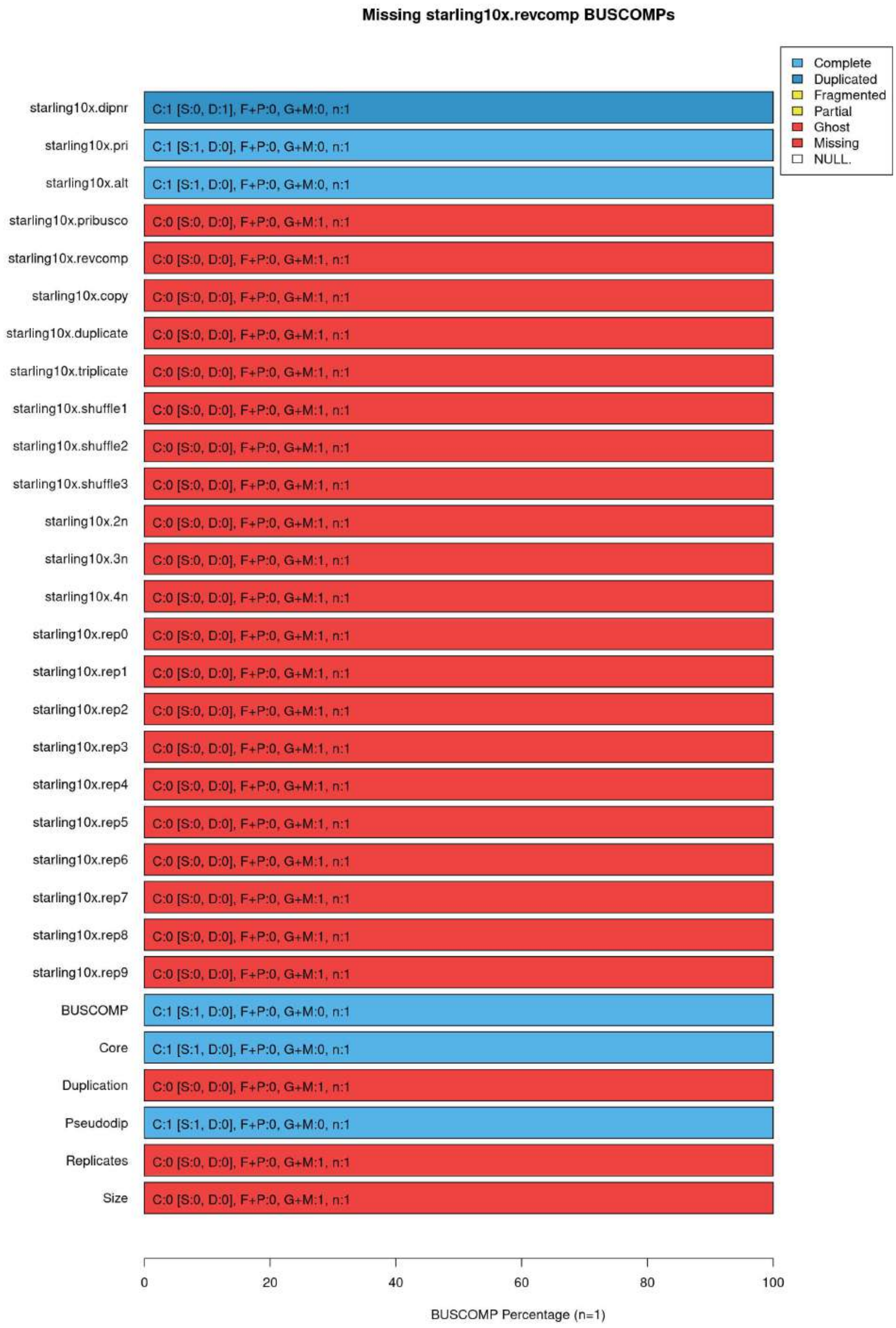

5.10 Missing Core BUSCO genes

BUSCO ratings for `Missing` Core BUSCO genes:

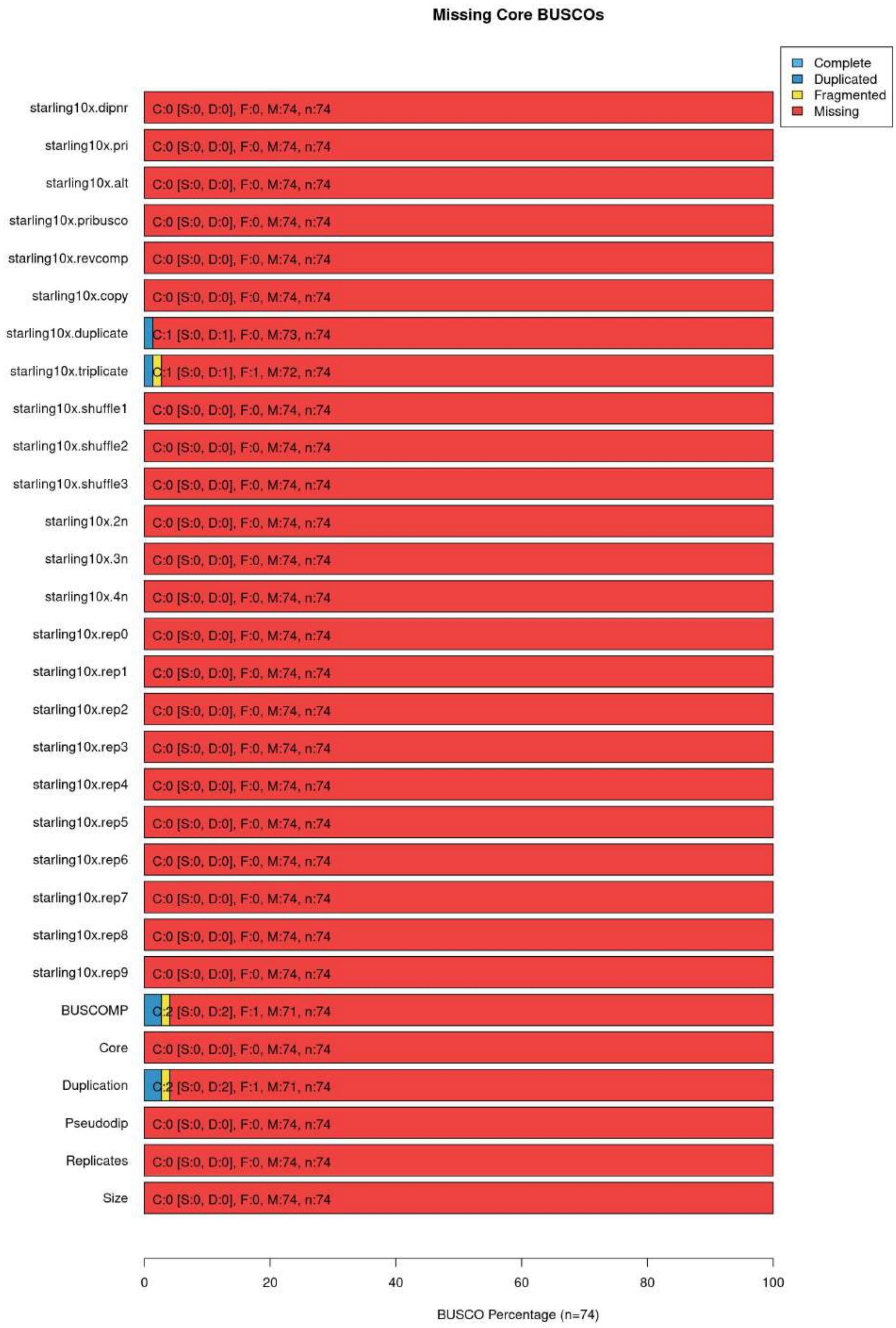

BUSCOMP ratings for `Missing` Core BUSCO genes:

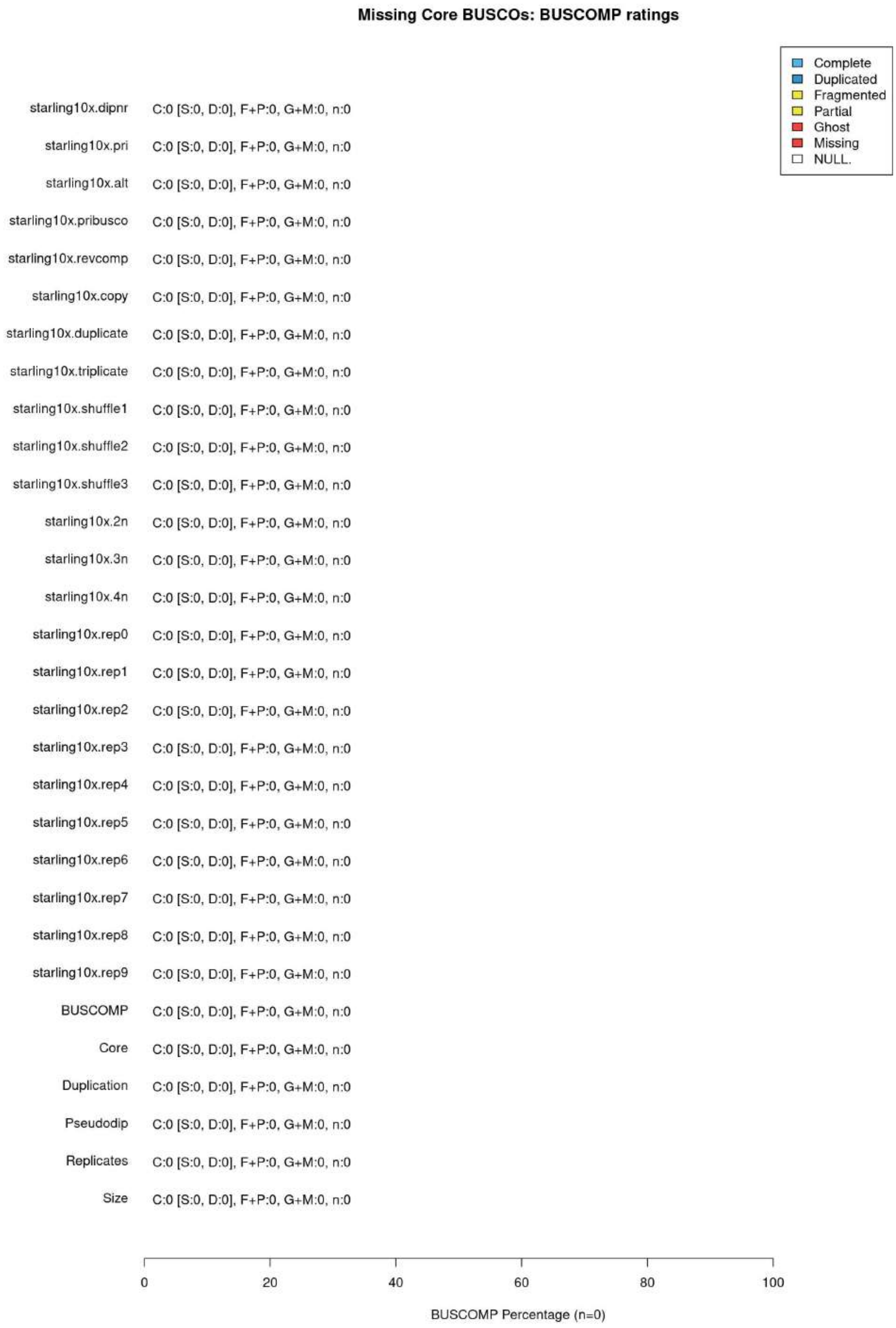

BUSCOMP ratings for `Missing` Core BUSCOMP genes:

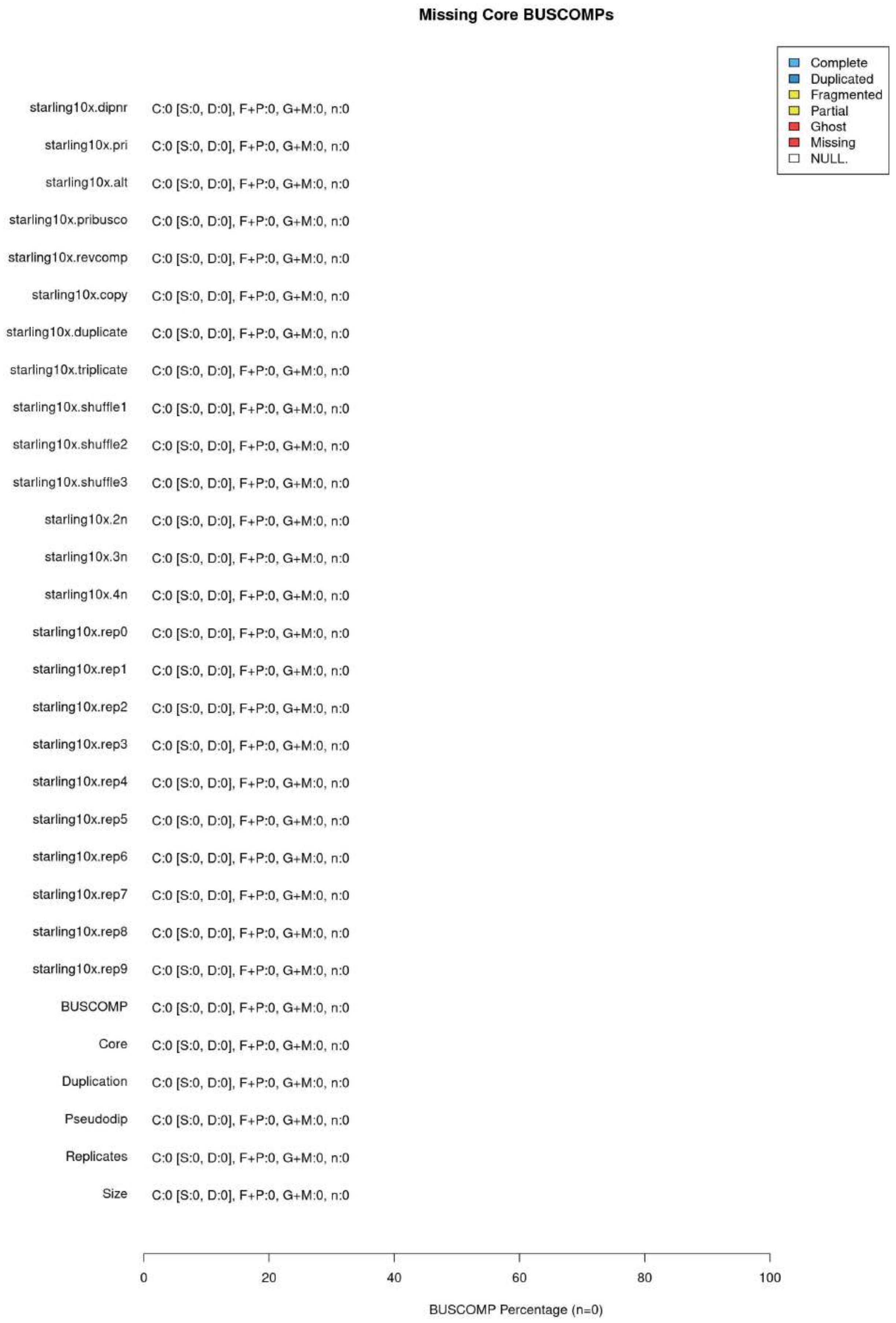

5.11 Missing starling10x.copy BUSCO genes

BUSCO ratings for `Missing` starling10x.copy BUSCO genes:

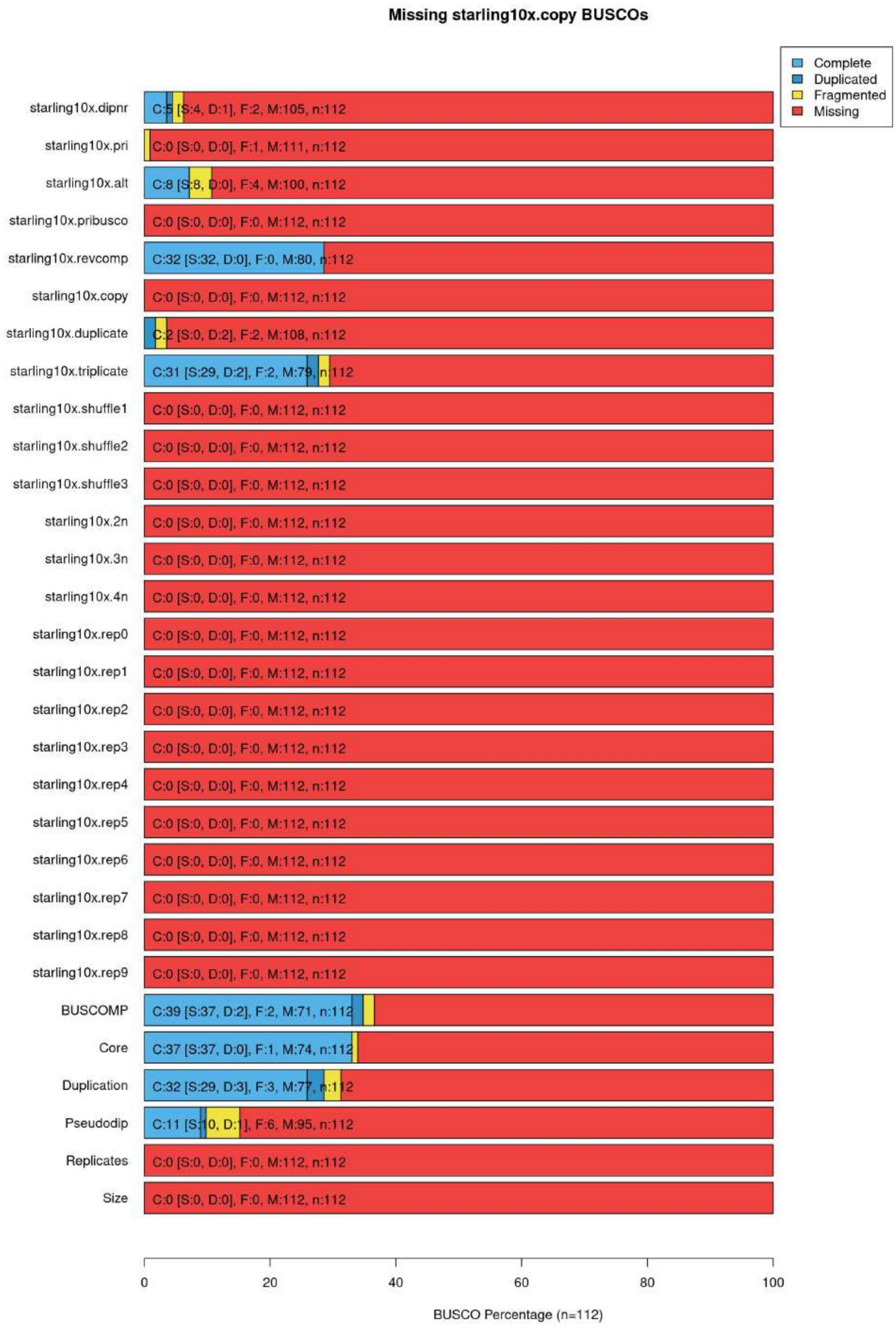

BUSCOMP ratings for `Missing` starling10x.copy BUSCO genes:

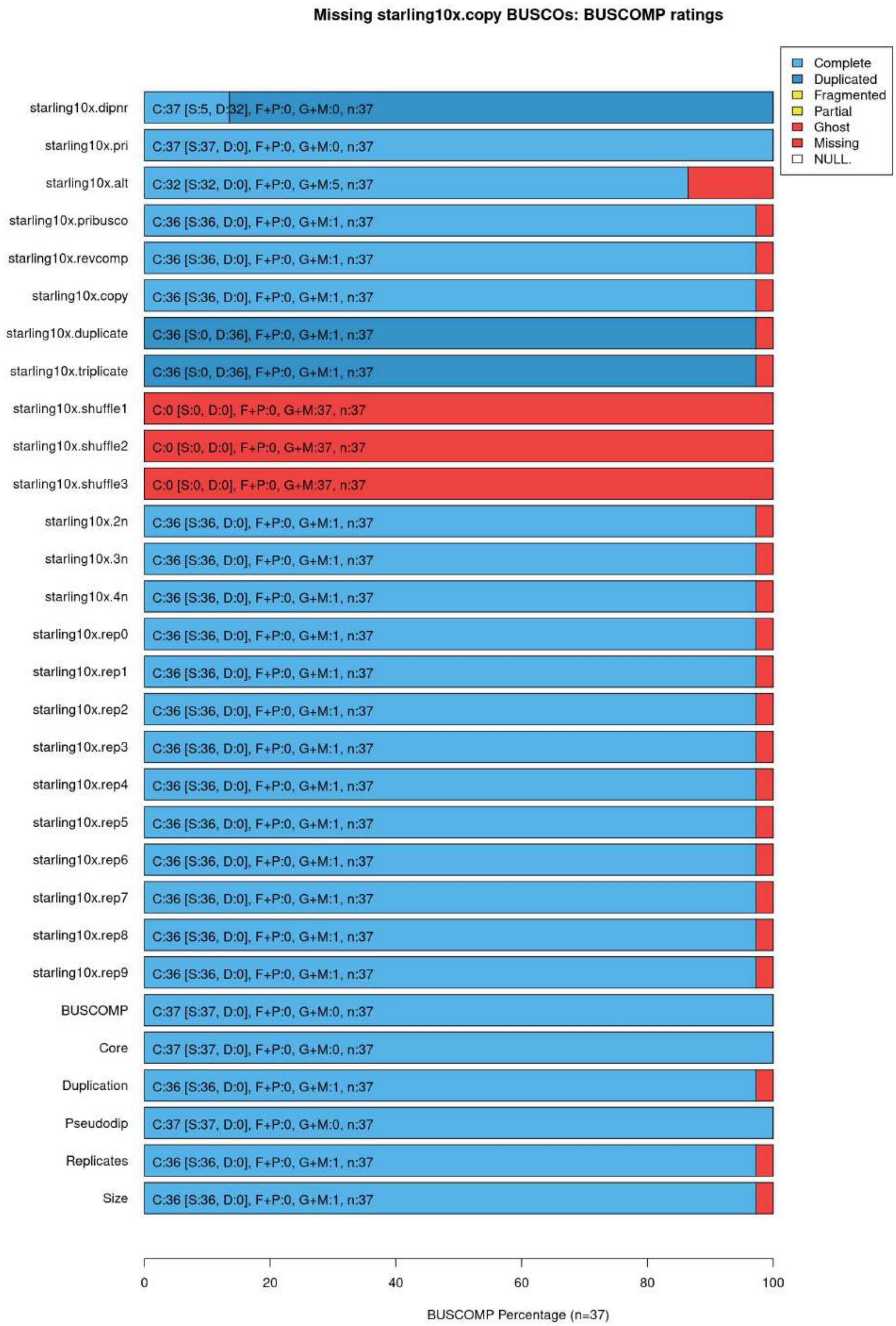

BUSCOMP ratings for `Missing` starling10x.copy BUSCOMP genes:

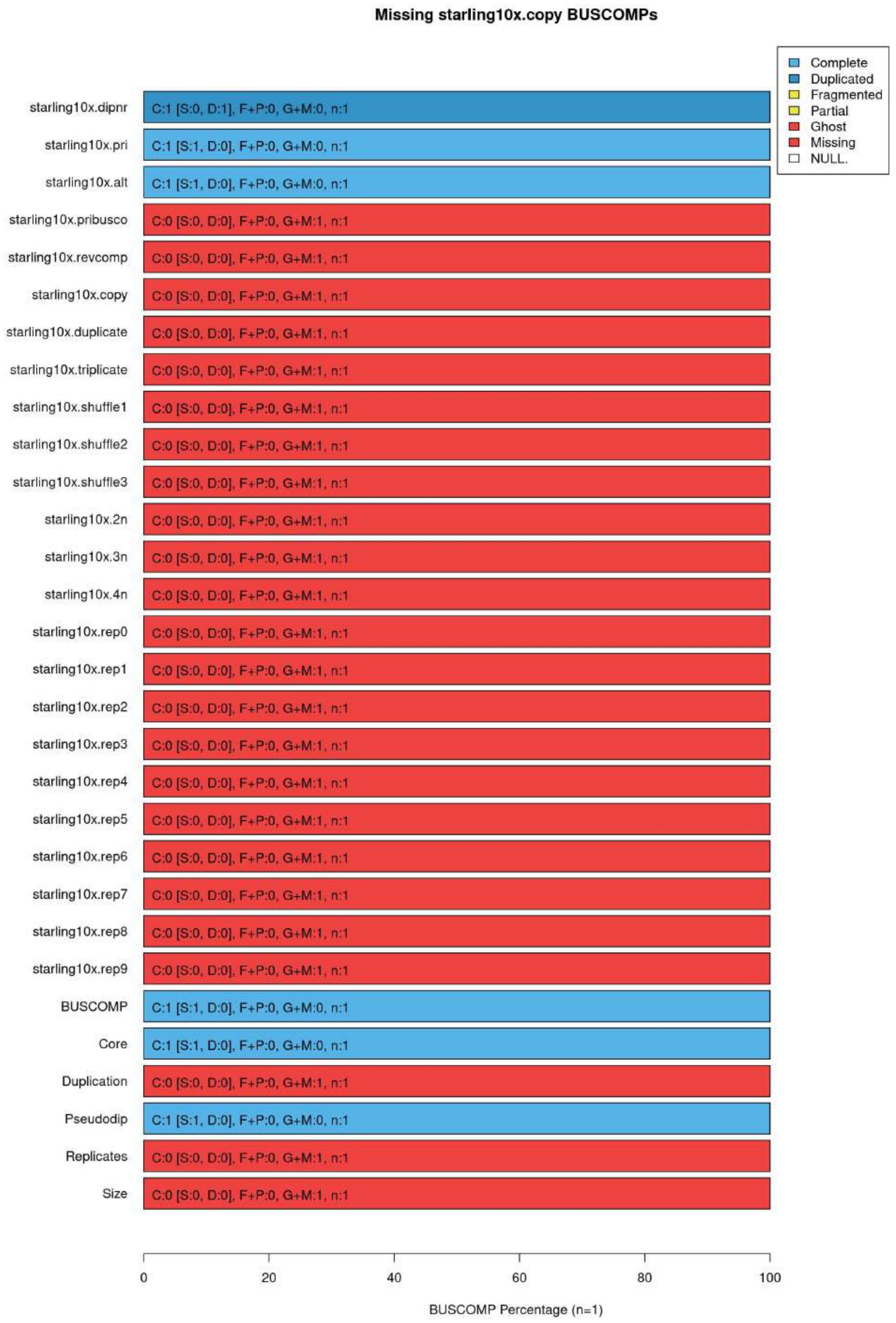

5.12 Missing starling10x.duplicate BUSCO genes

BUSCO ratings for `Missing` starling10x.duplicate BUSCO genes:

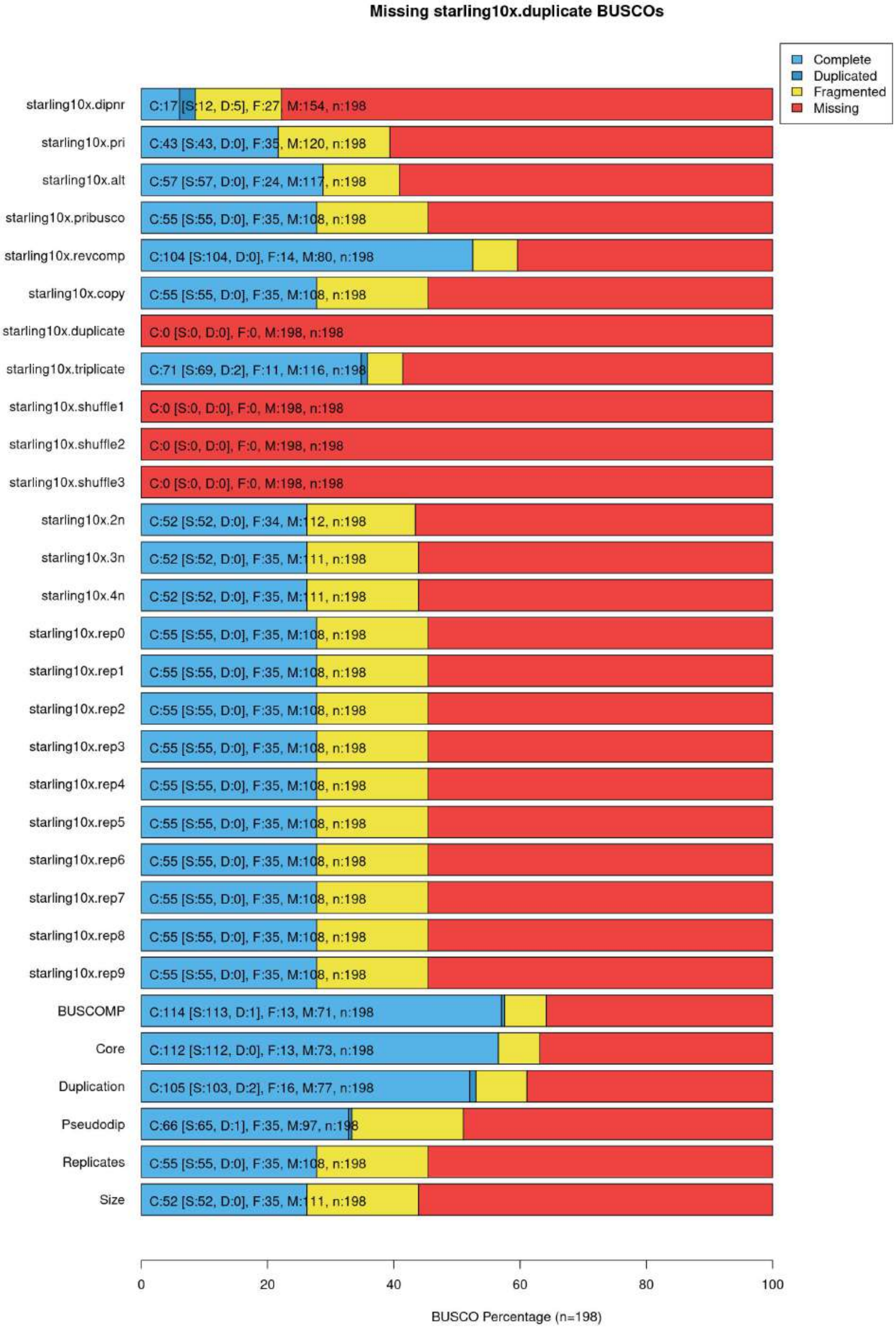

BUSCOMP ratings for `Missing` starling10x.duplicate BUSCO genes:

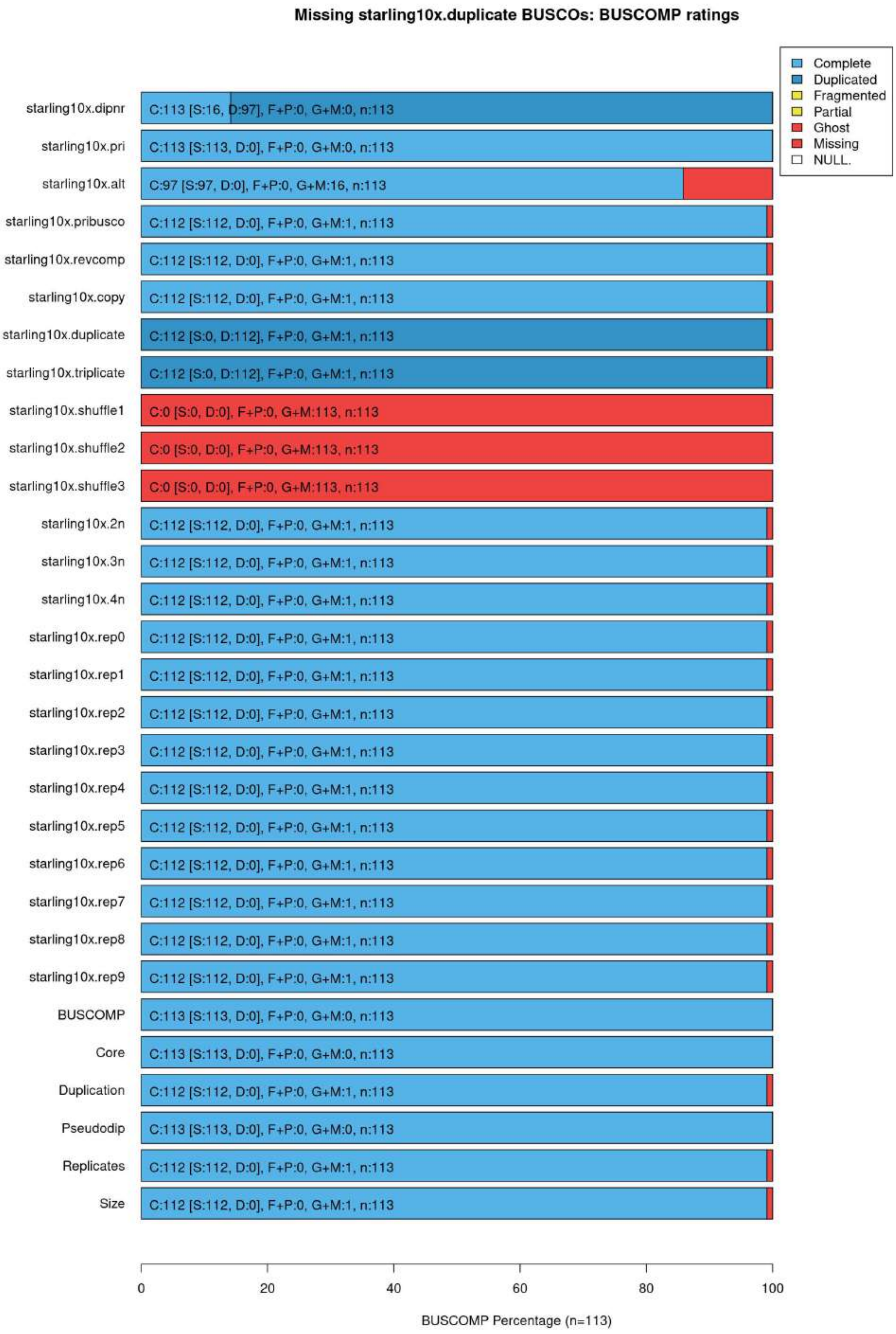

BUSCOMP ratings for `Missing` starling10x.duplicate BUSCOMP genes:

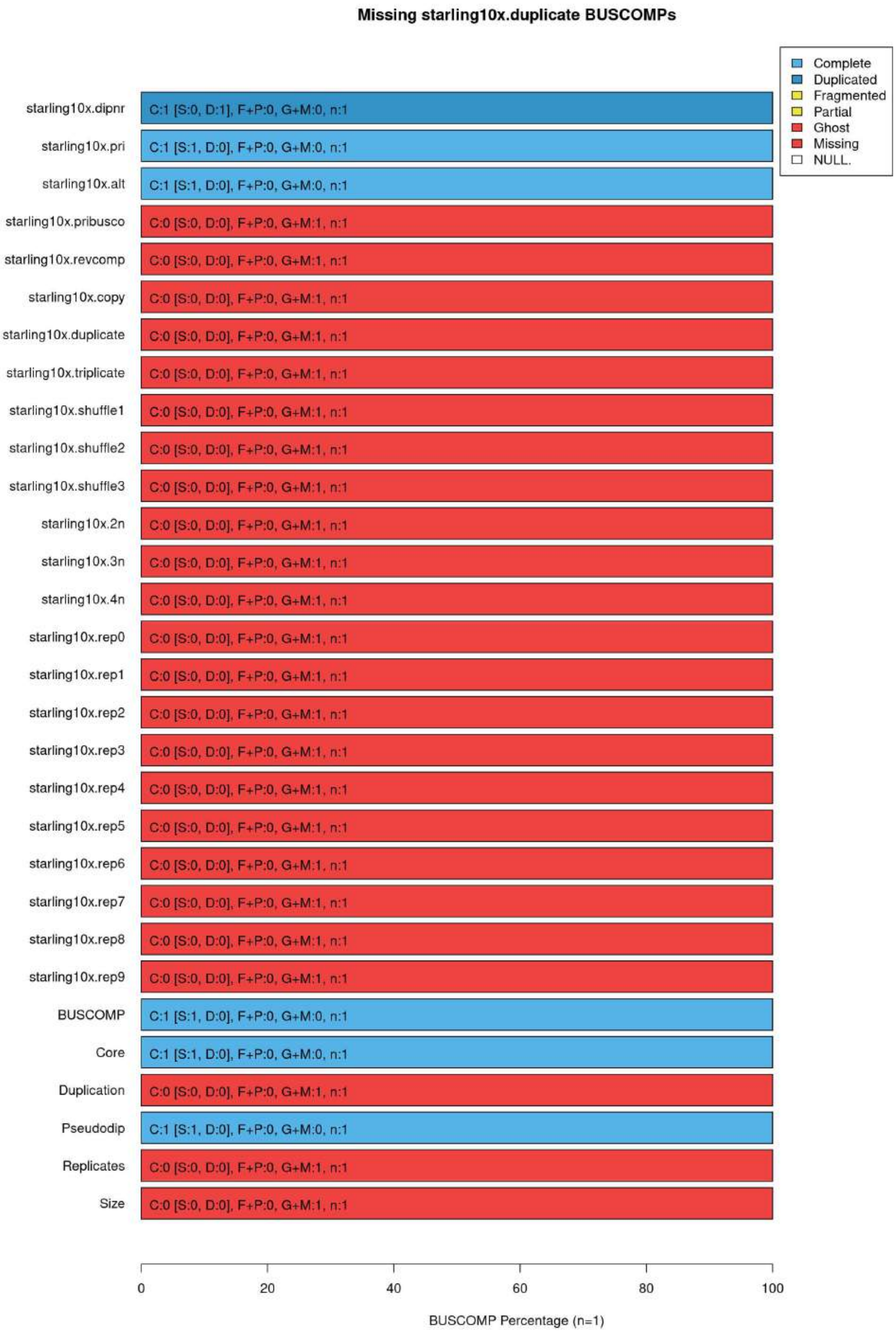

5.13 Missing starling10x.triplicate BUSCO genes

BUSCO ratings for `Missing` starling10x.triplicate BUSCO genes:

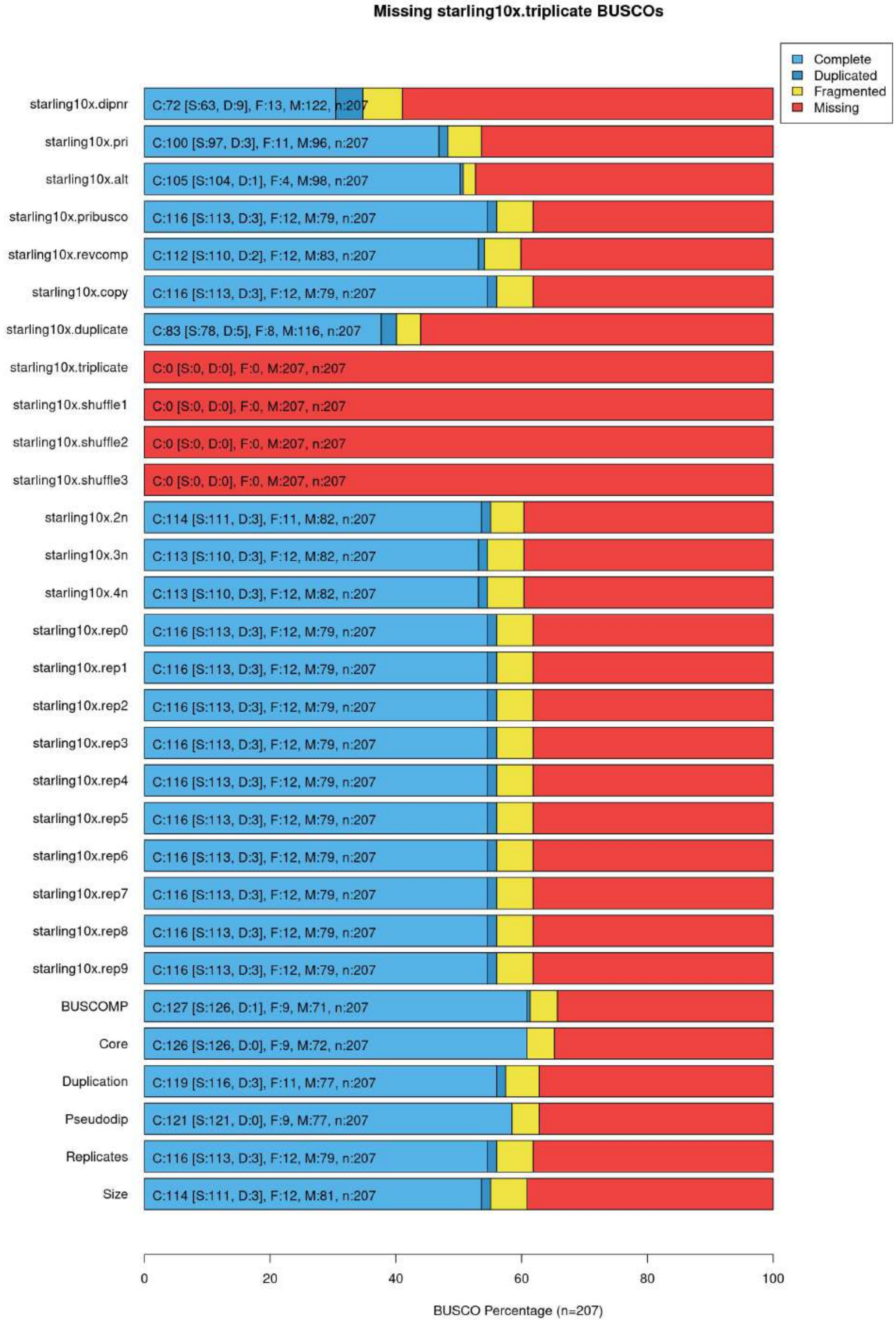

BUSCOMP ratings for `Missing` starling10x.triplicate BUSCO genes:

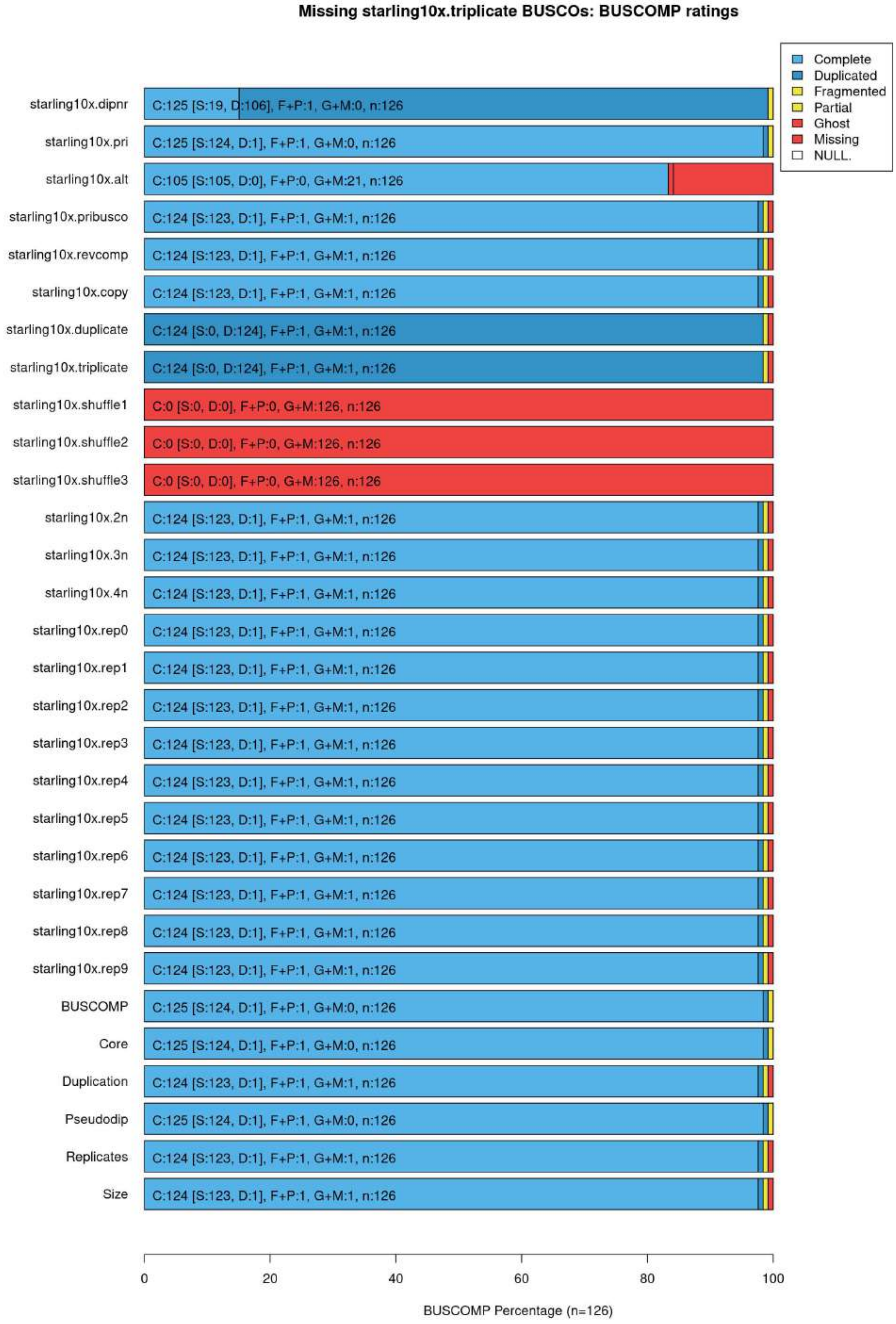

BUSCOMP ratings for `Missing` starling10x.triplicate BUSCOMP genes:

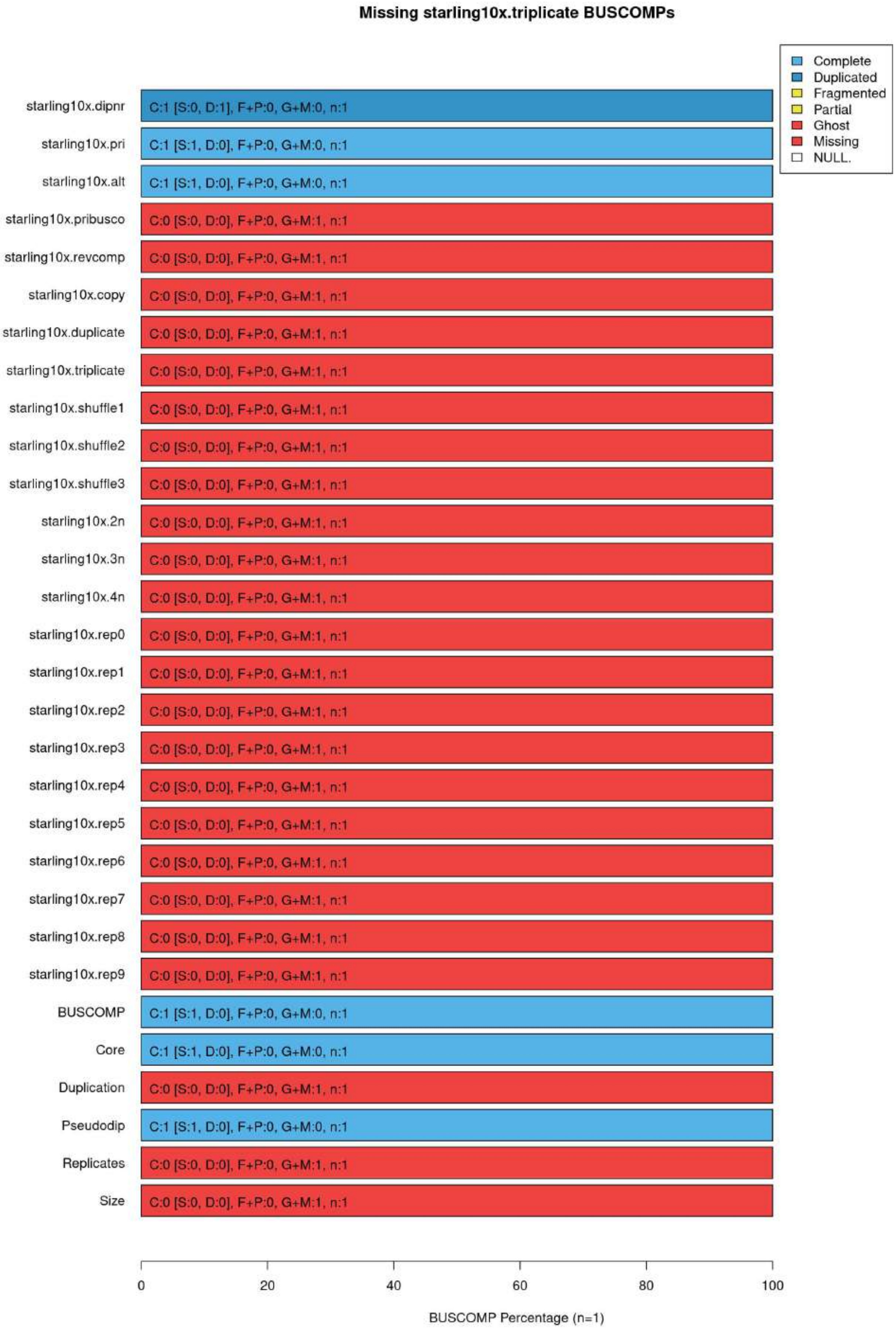

5.14 Missing Duplication BUSCO genes

BUSCO ratings for `Missing` Duplication BUSCO genes:

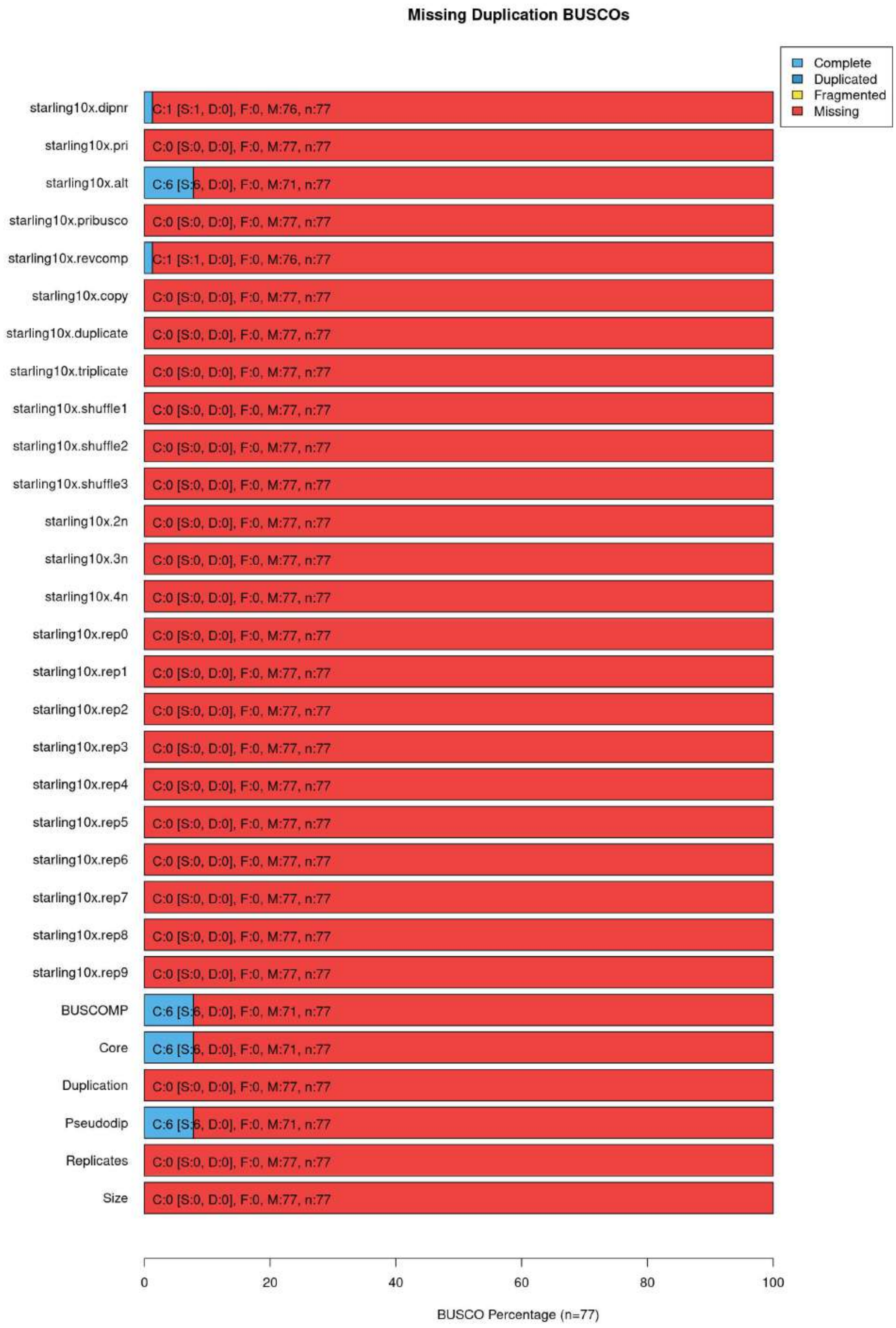

BUSCOMP ratings for `Missing` Duplication BUSCO genes:

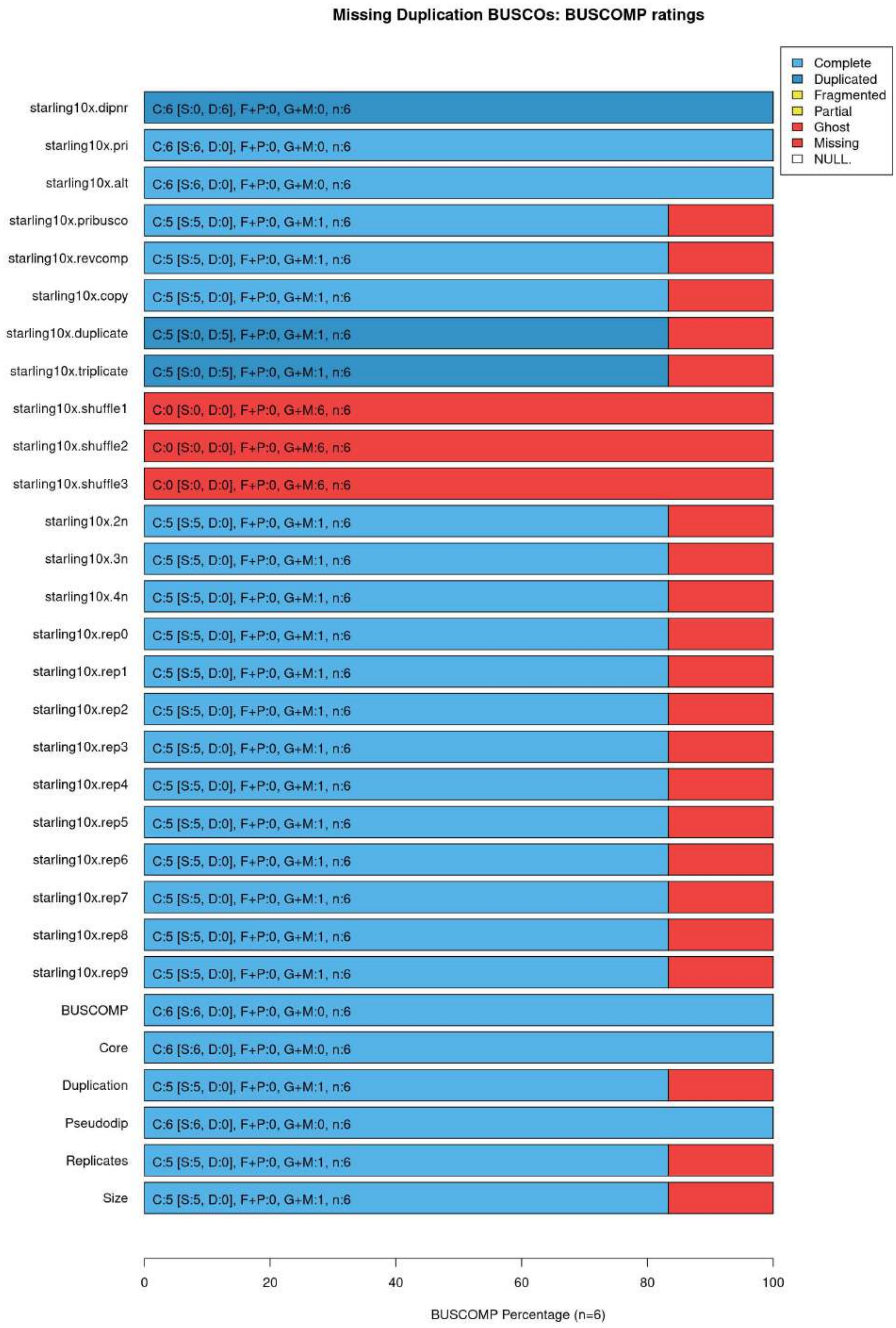

BUSCOMP ratings for `Missing` Duplication BUSCOMP genes:

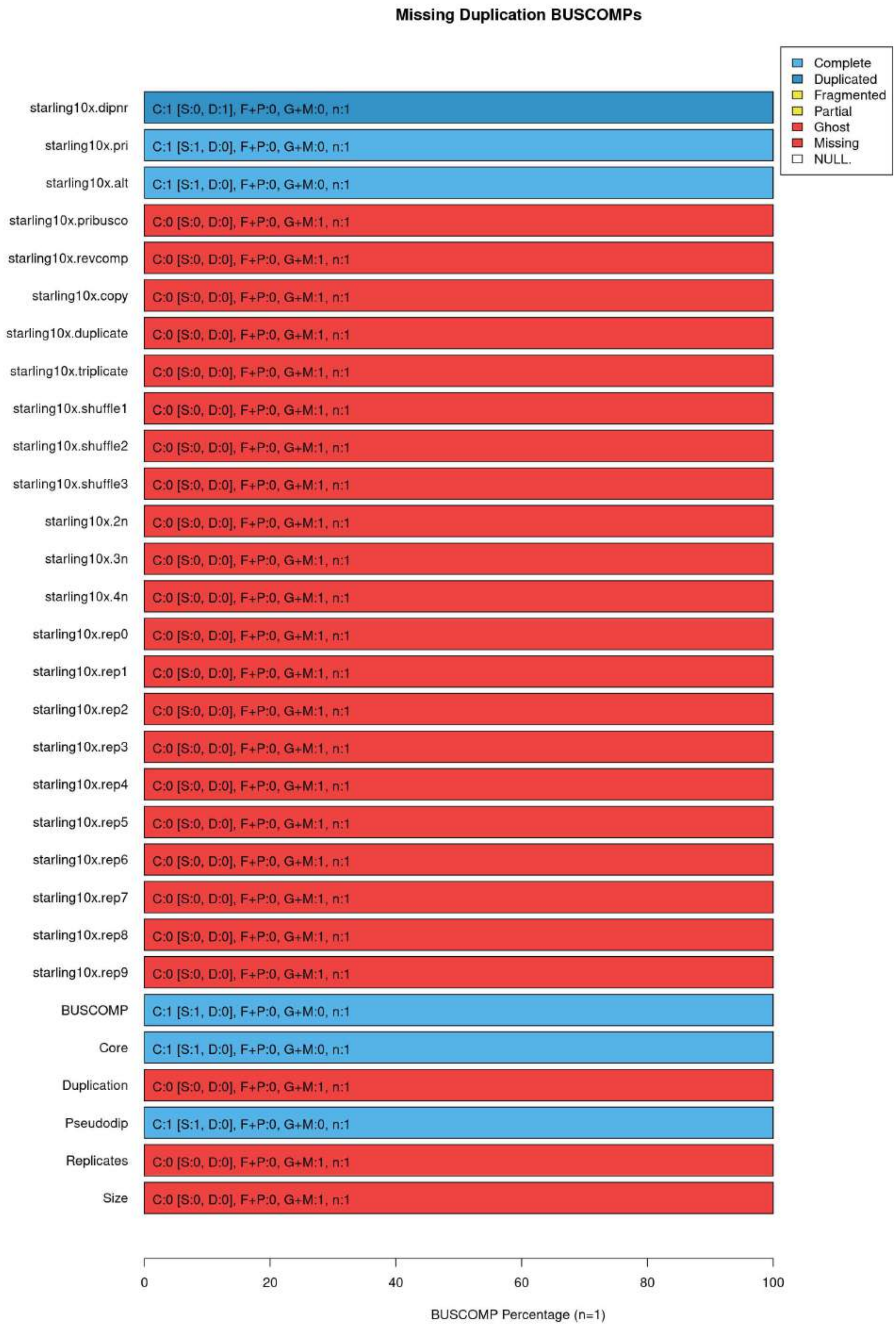

5.15 Missing starling10x.shuffle1 BUSCO genes

BUSCO ratings for `Missing` starling10x.shuffle1 BUSCO genes:

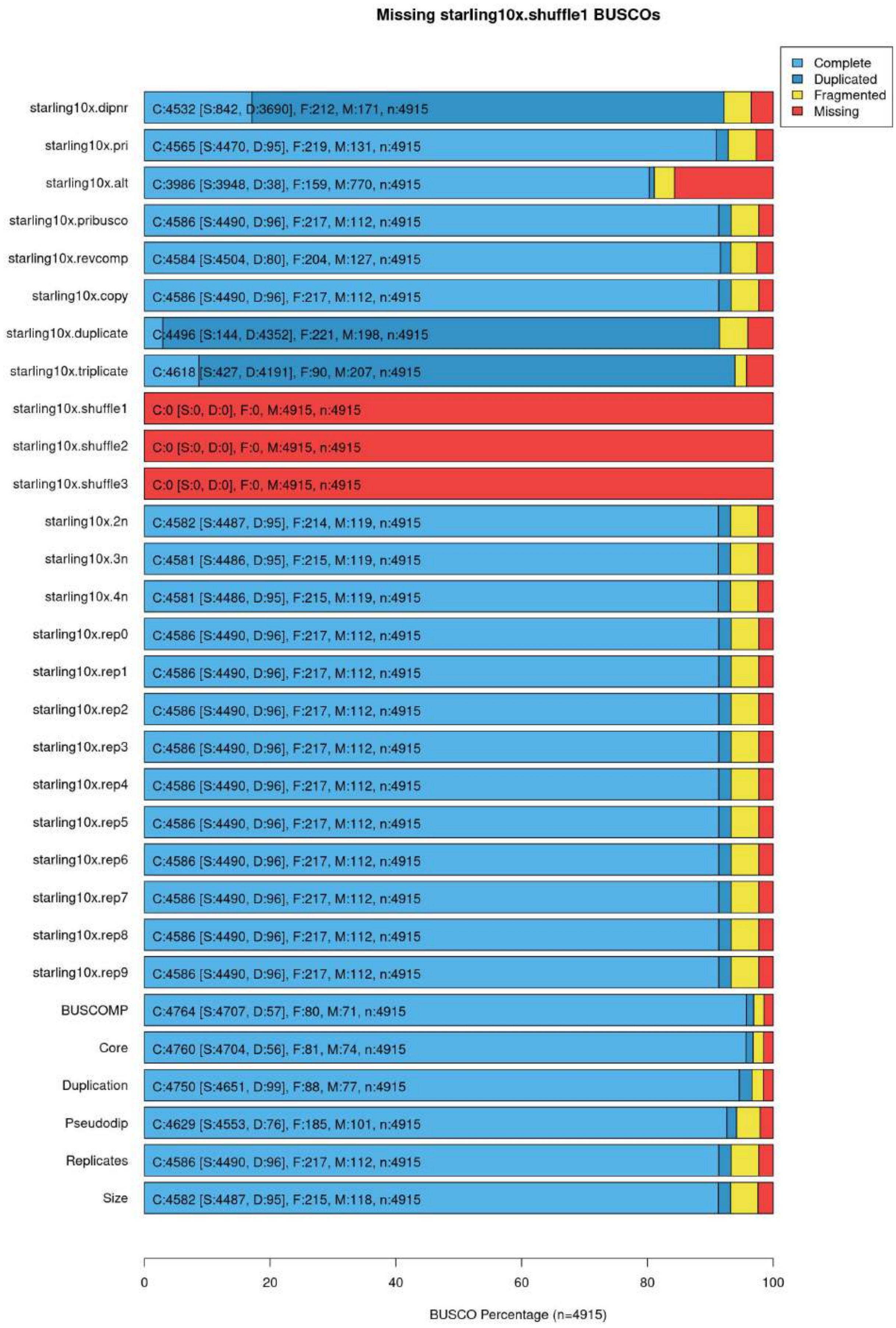

BUSCOMP ratings for `Missing` starling10x.shuffle1 BUSCO genes:

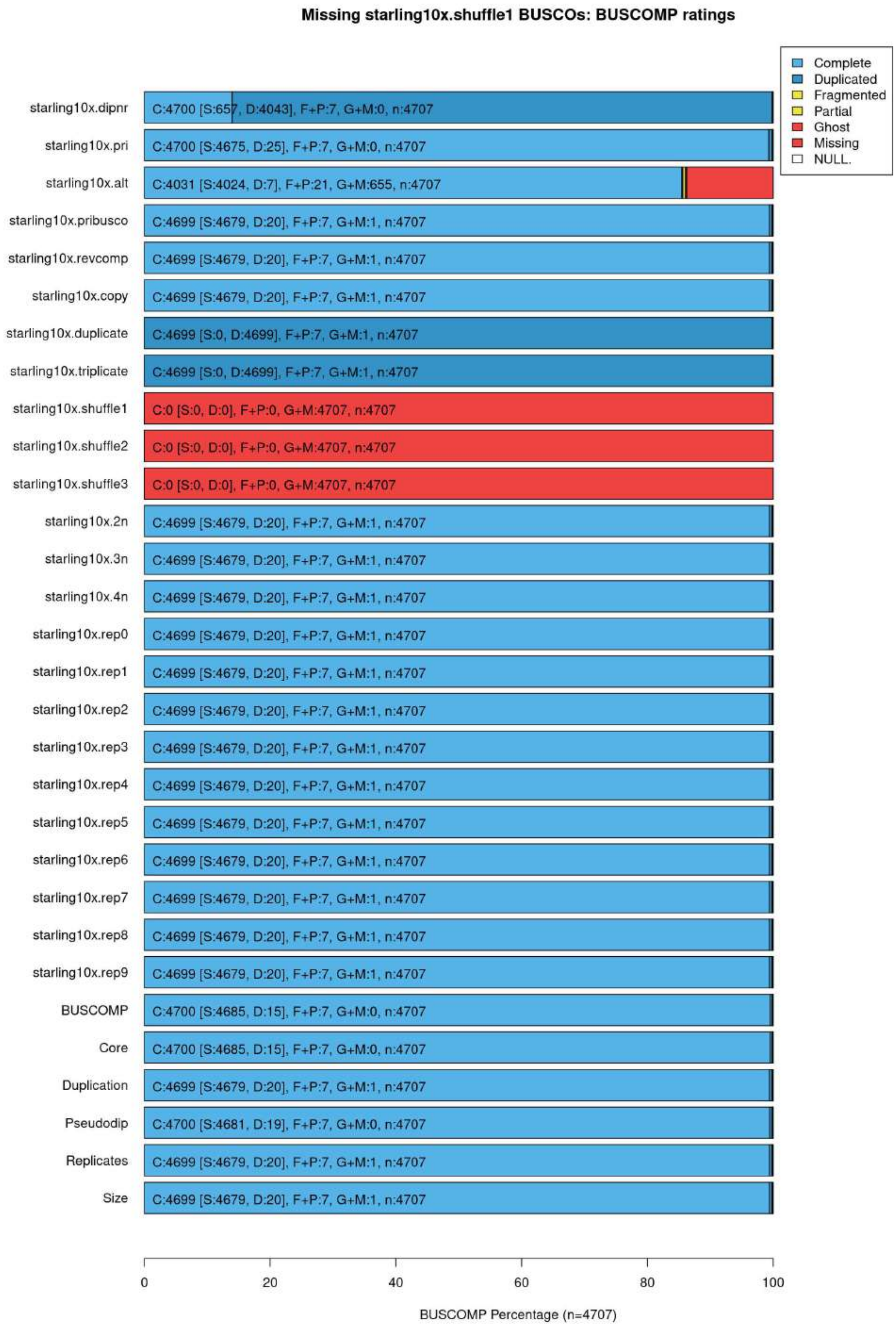

BUSCOMP ratings for `Missing` starling10x.shuffle1 BUSCOMP genes:

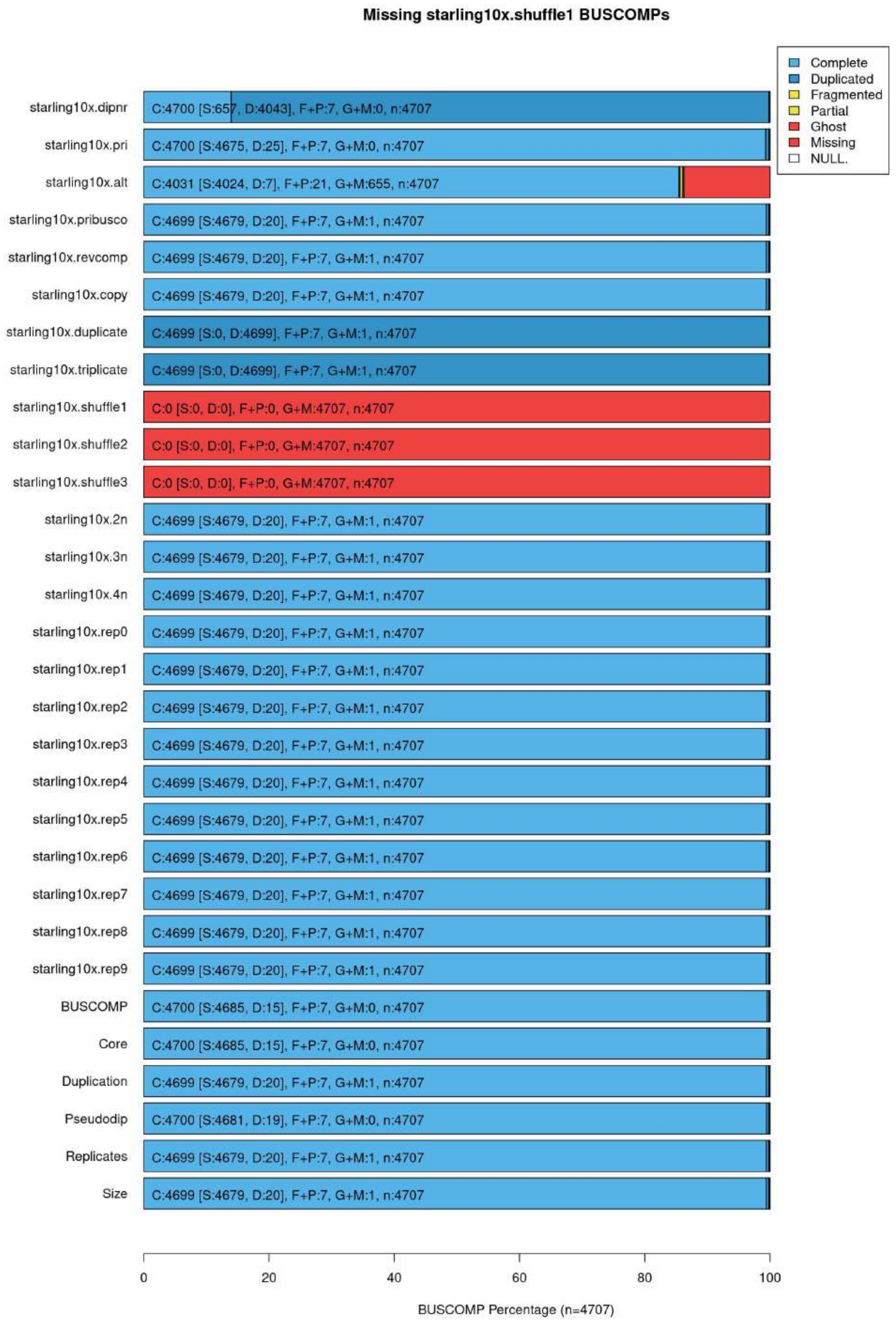

5.16 Missing starling10x.shuffle2 BUSCO genes

BUSCO ratings for `Missing` starling10x.shuffle2 BUSCO genes:

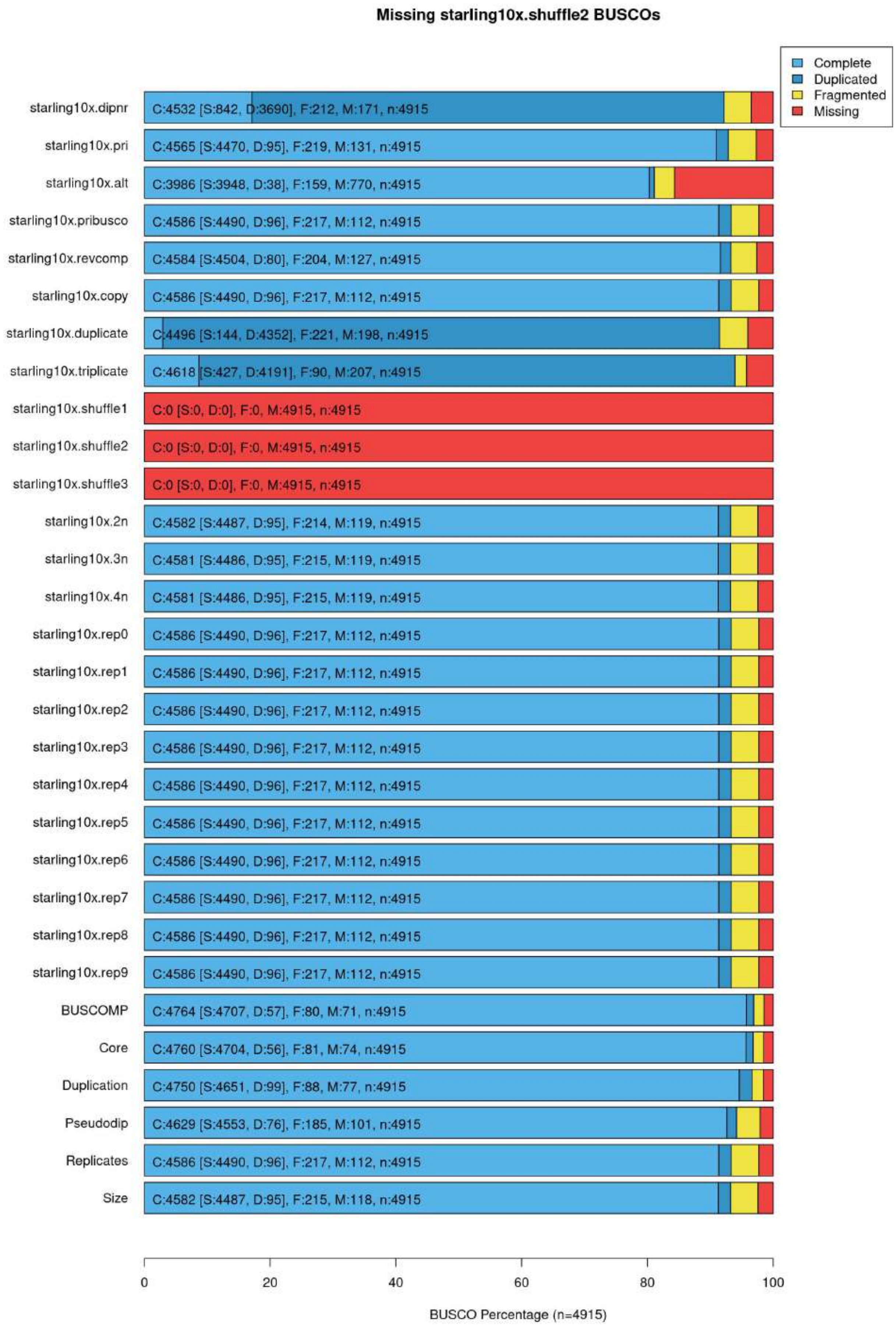

BUSCOMP ratings for `Missing` starling10x.shuffle2 BUSCO genes:

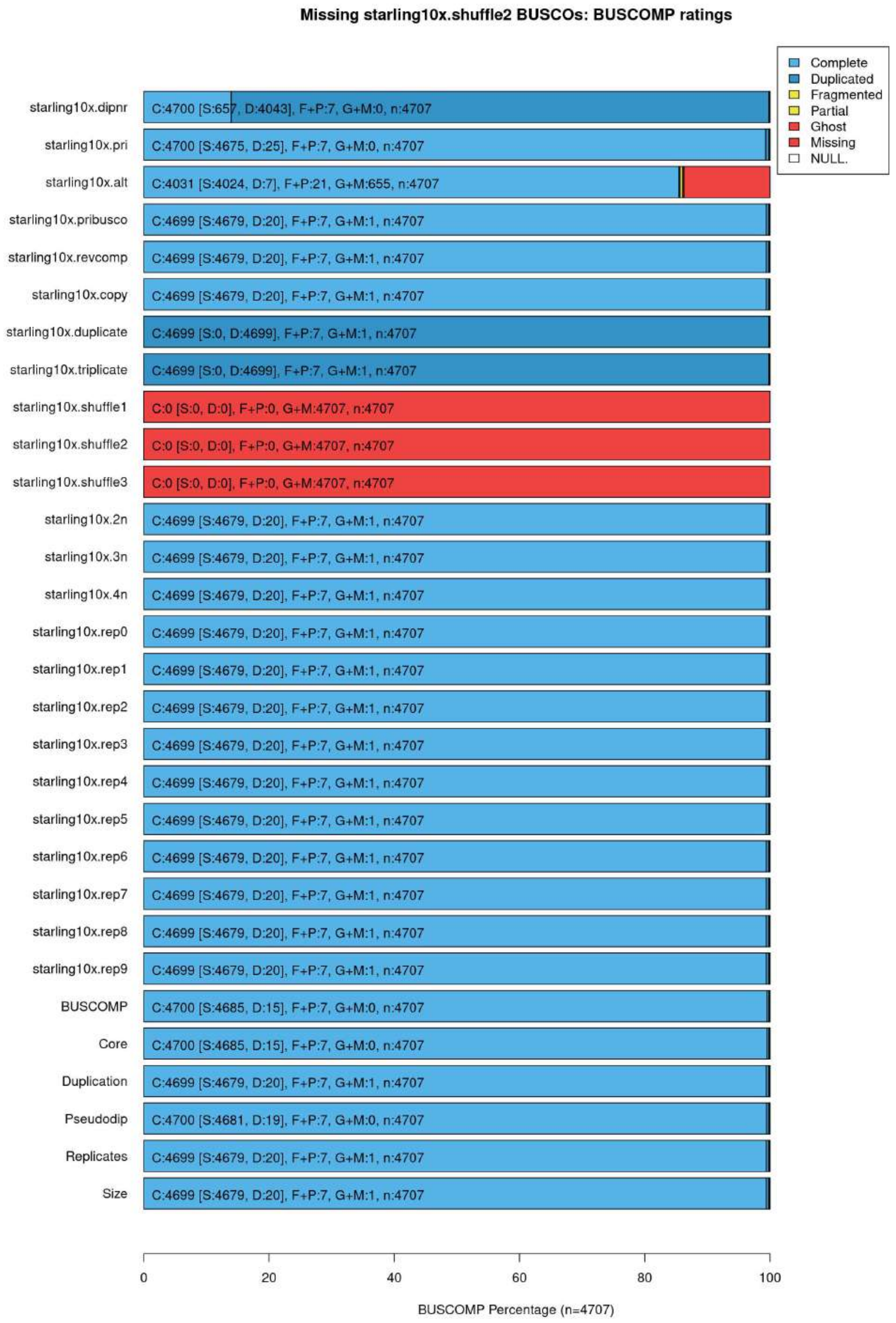

BUSCOMP ratings for `Missing` starling10x.shuffle2 BUSCOMP genes:

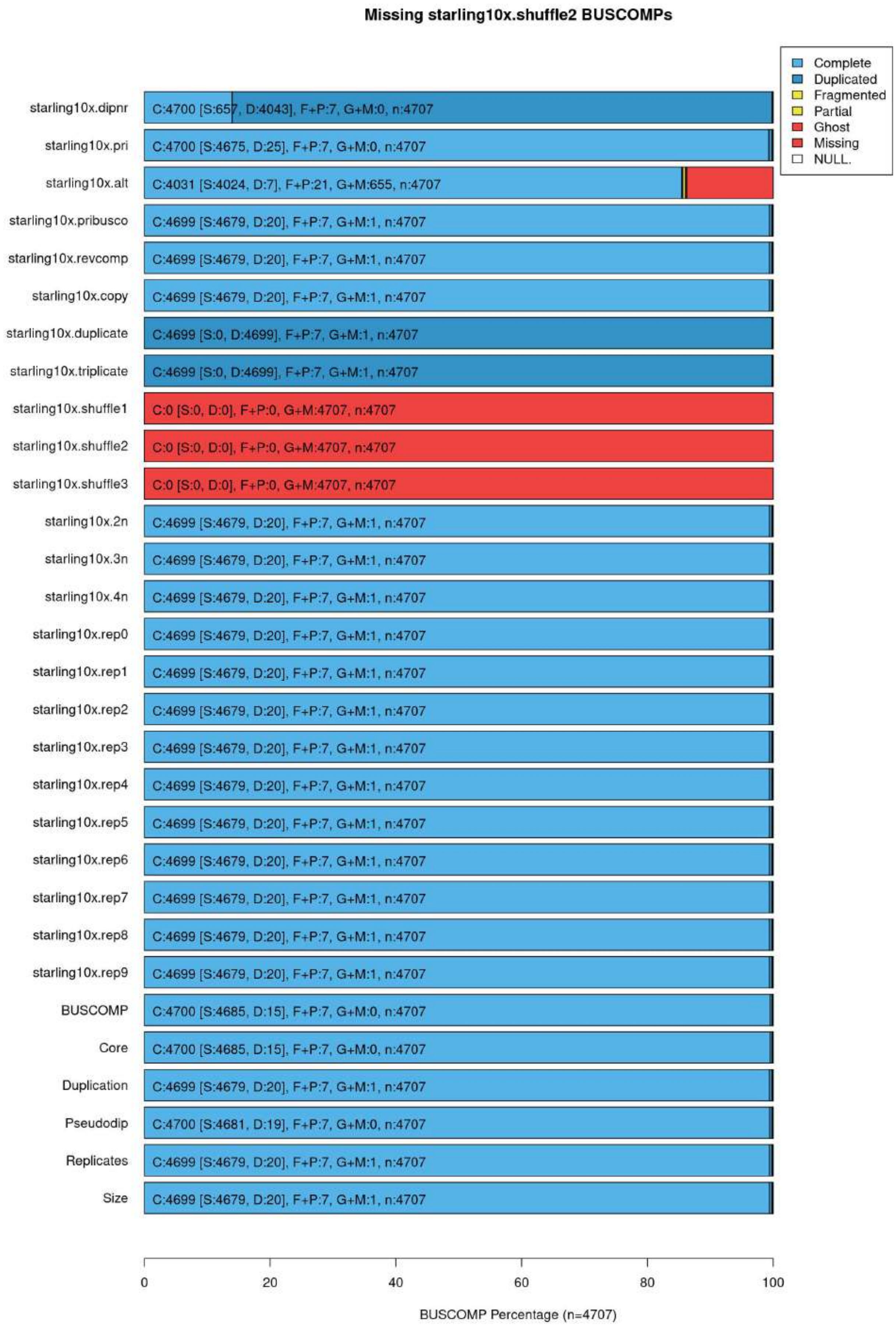

5.17 Missing starling10x.shuffle3 BUSCO genes

BUSCO ratings for `Missing` starling10x.shuffle3 BUSCO genes:

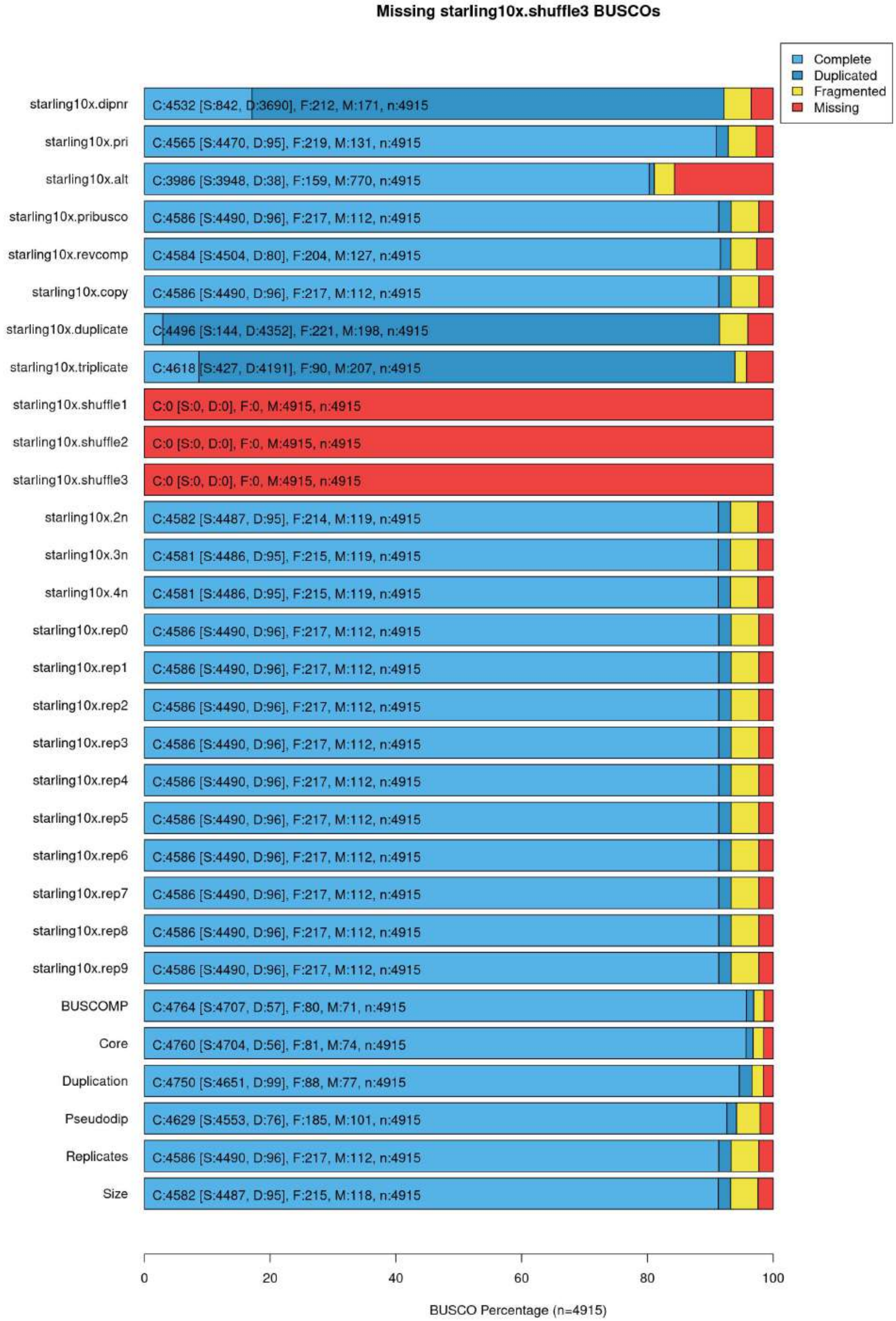

BUSCOMP ratings for `Missing` starling10x.shuffle3 BUSCO genes:

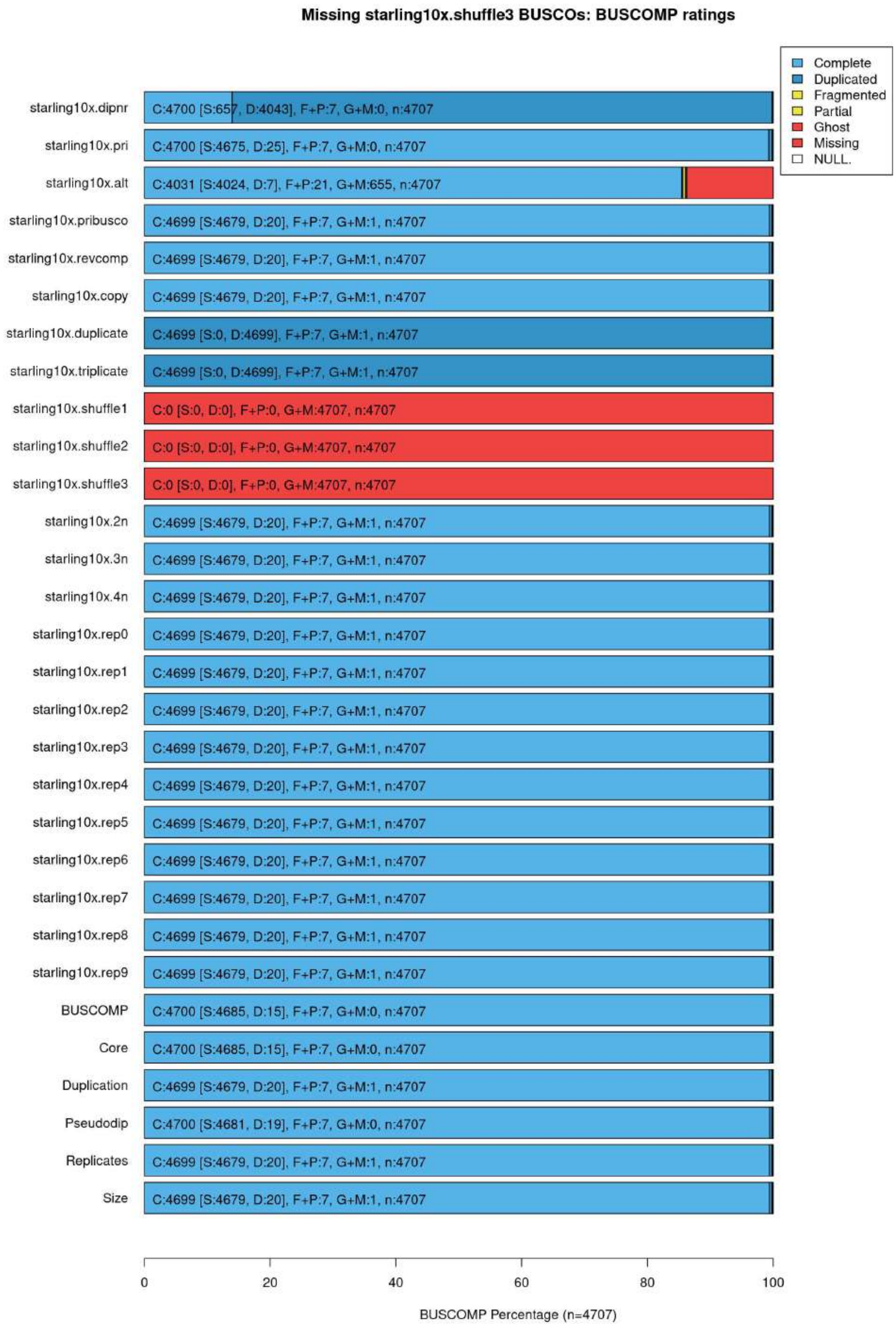

BUSCOMP ratings for `Missing` starling10x.shuffle3 BUSCOMP genes:

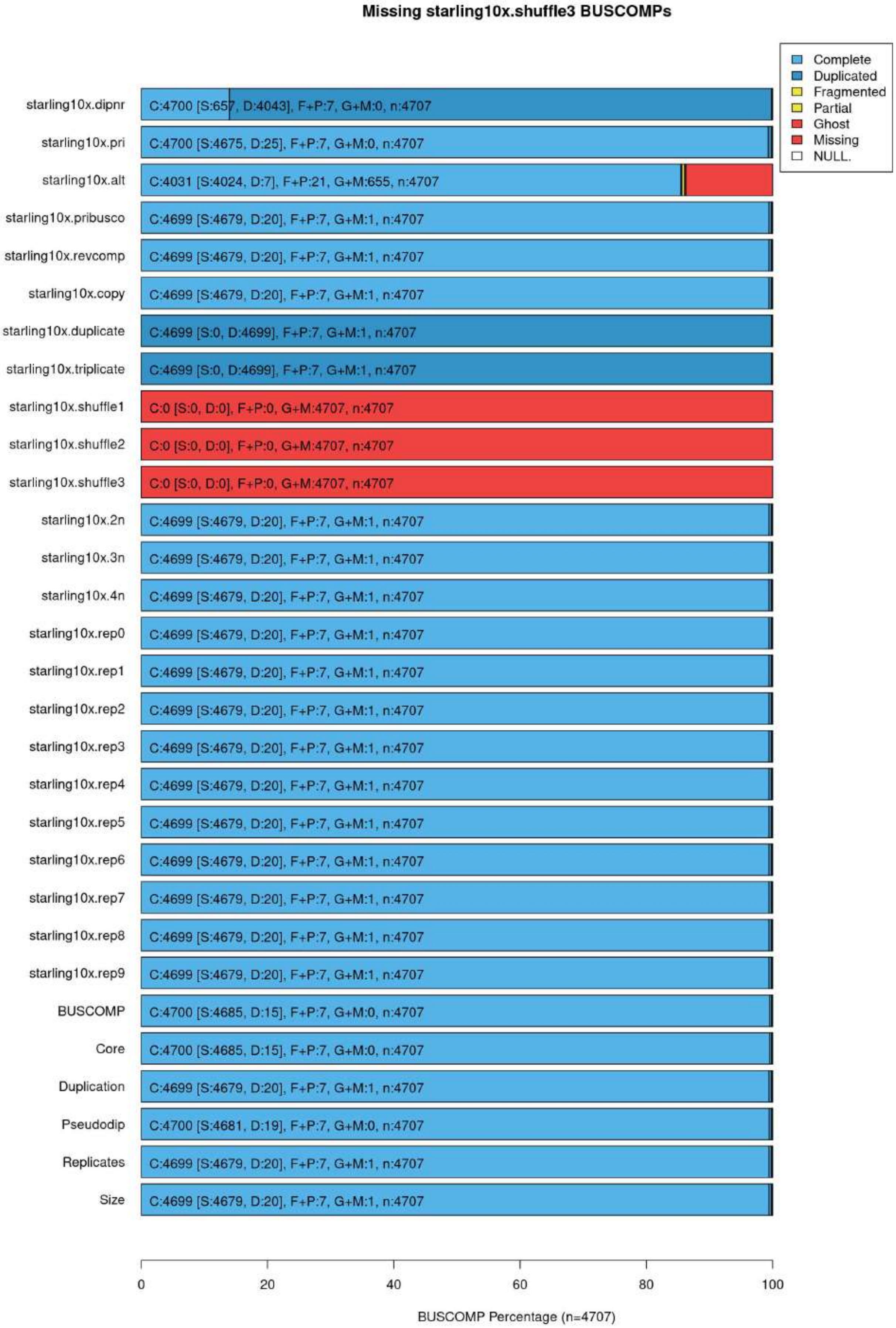

5.18 Missing starling10x.2n BUSCO genes

BUSCO ratings for `Missing` starling10x.2n BUSCO genes:

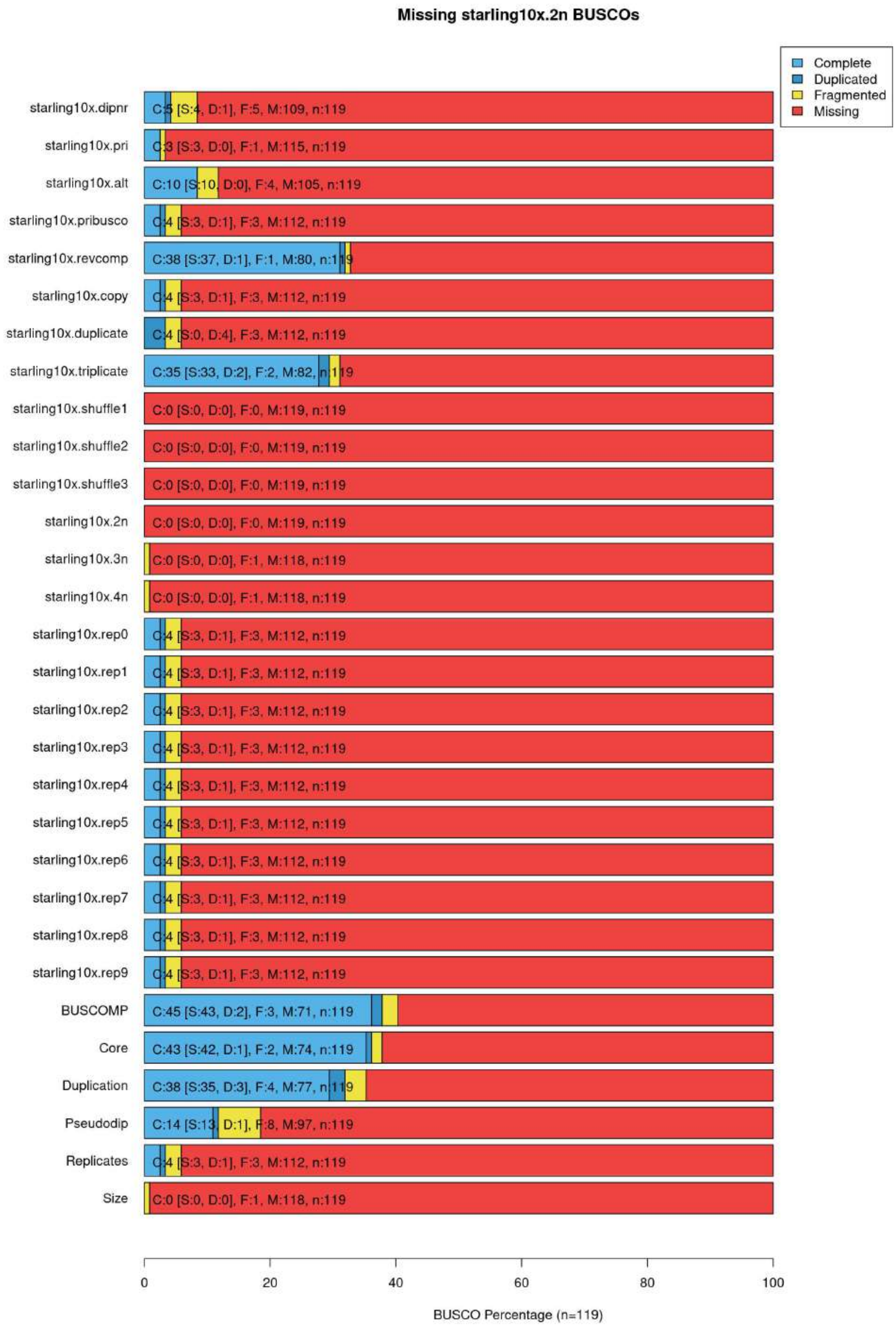

BUSCOMP ratings for `Missing` starling10x.2n BUSCO genes:

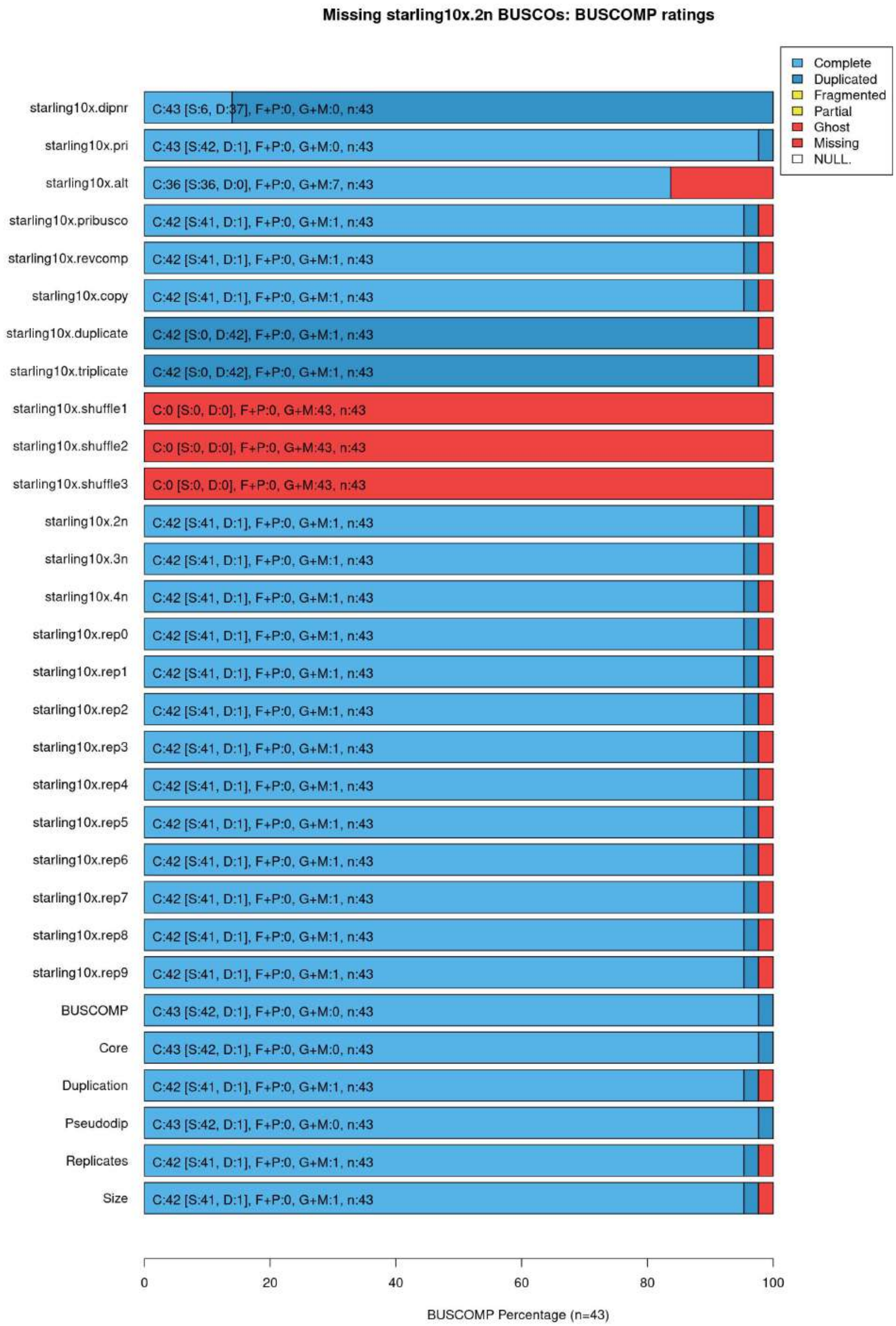

BUSCOMP ratings for `Missing` starling10x.2n BUSCOMP genes:

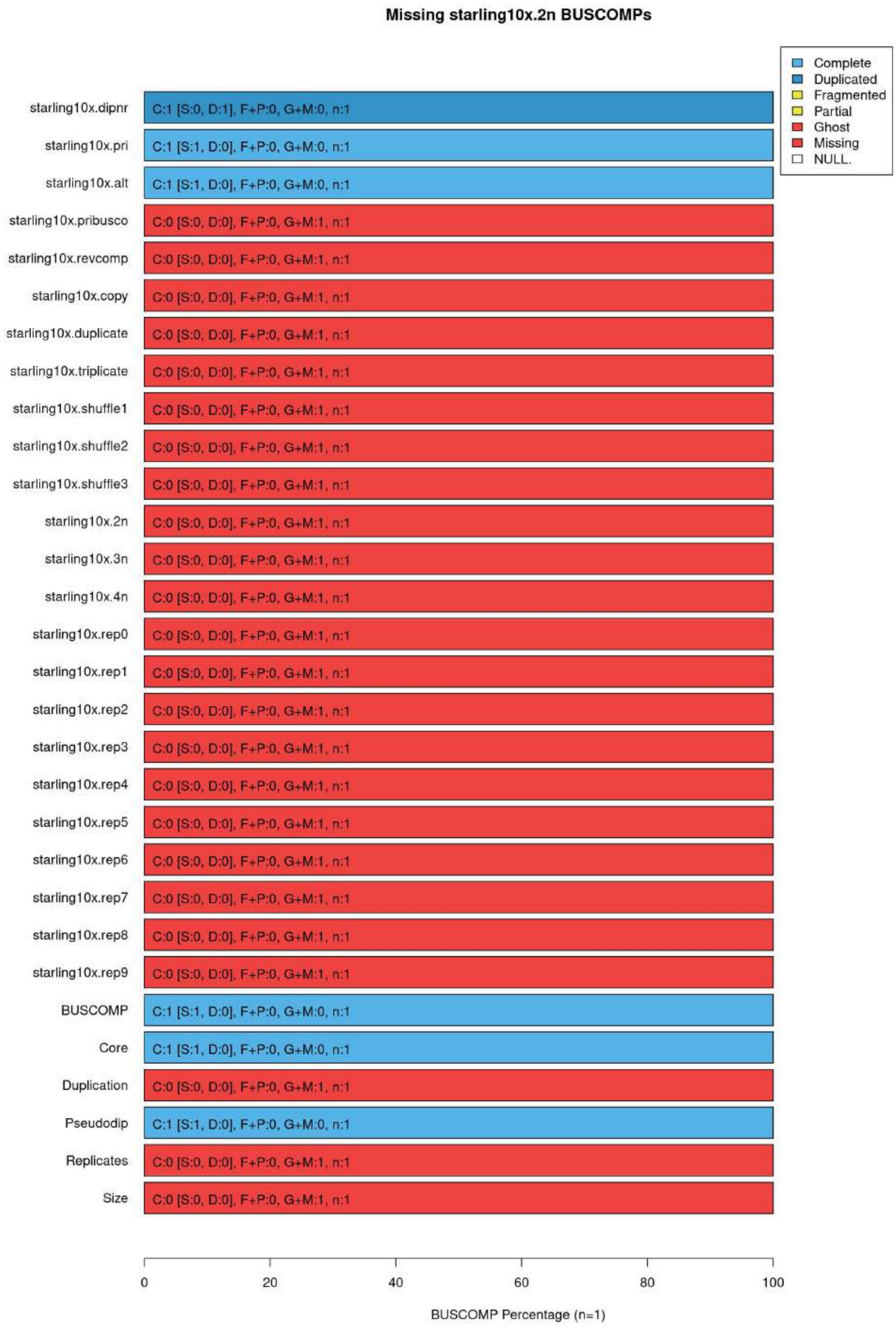

5.19 Missing starling10x.3n BUSCO genes

BUSCO ratings for `Missing` starling10x.3n BUSCO genes:

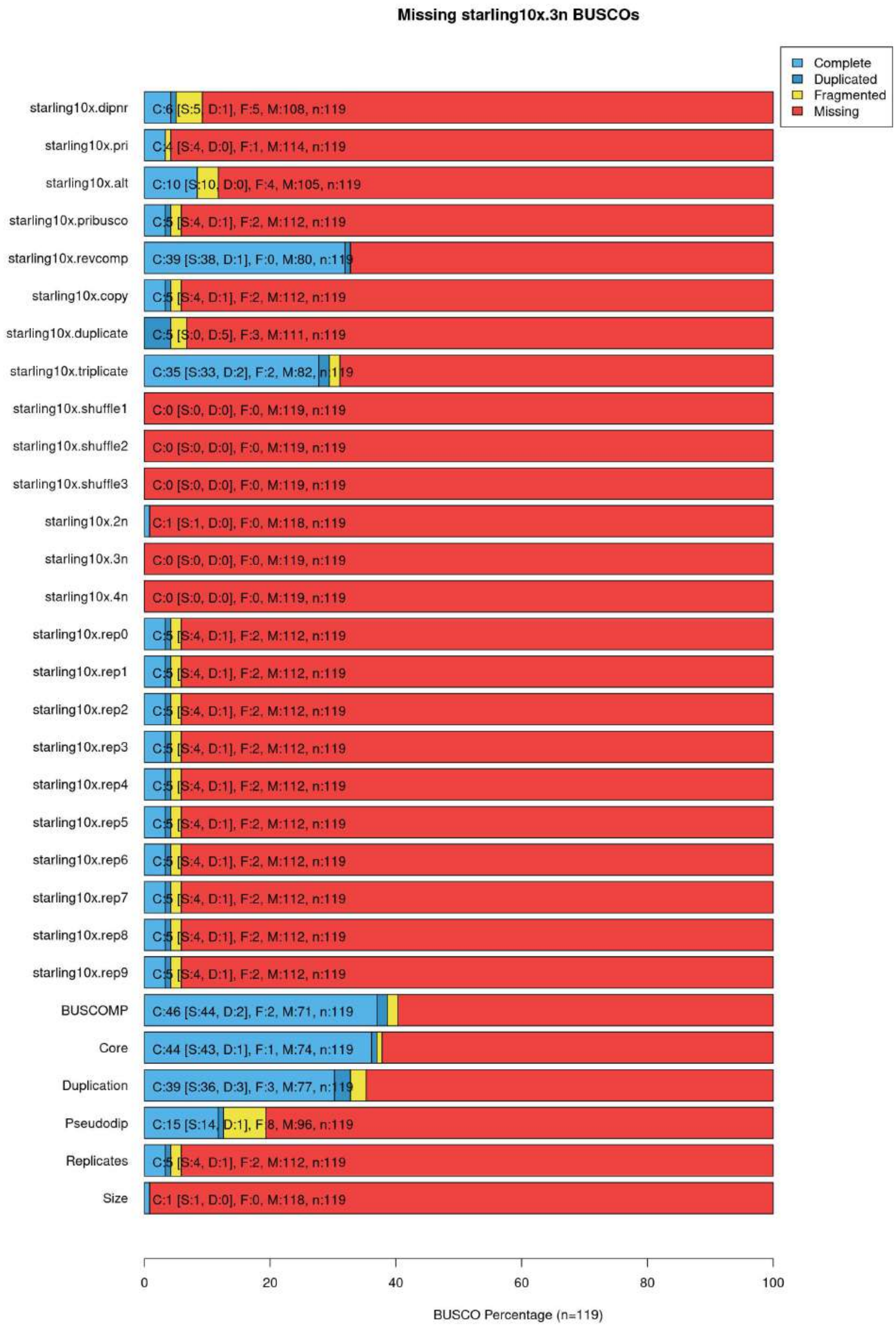

BUSCOMP ratings for `Missing` starling10x.3n BUSCO genes:

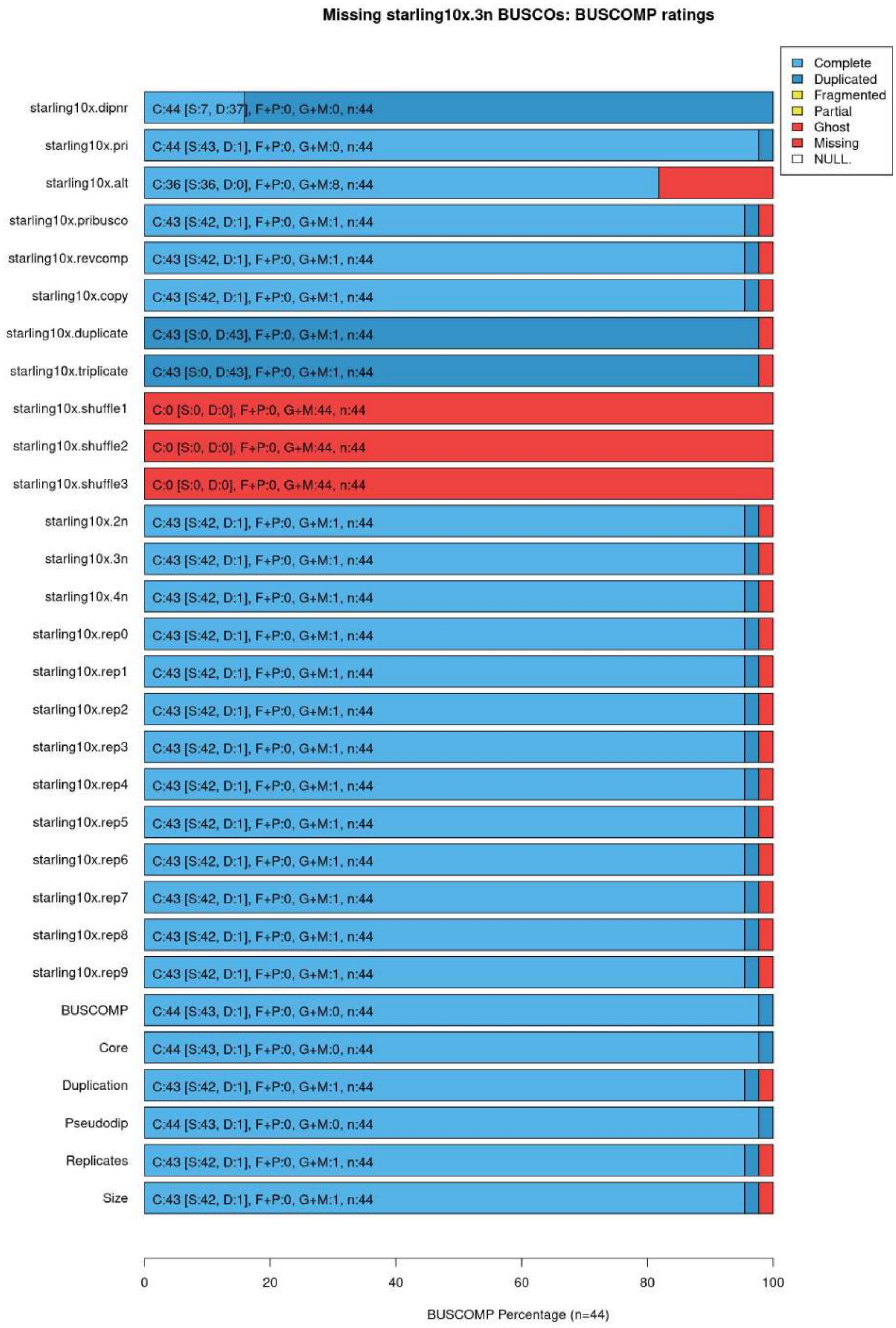

BUSCOMP ratings for `Missing` starling10x.3n BUSCOMP genes:

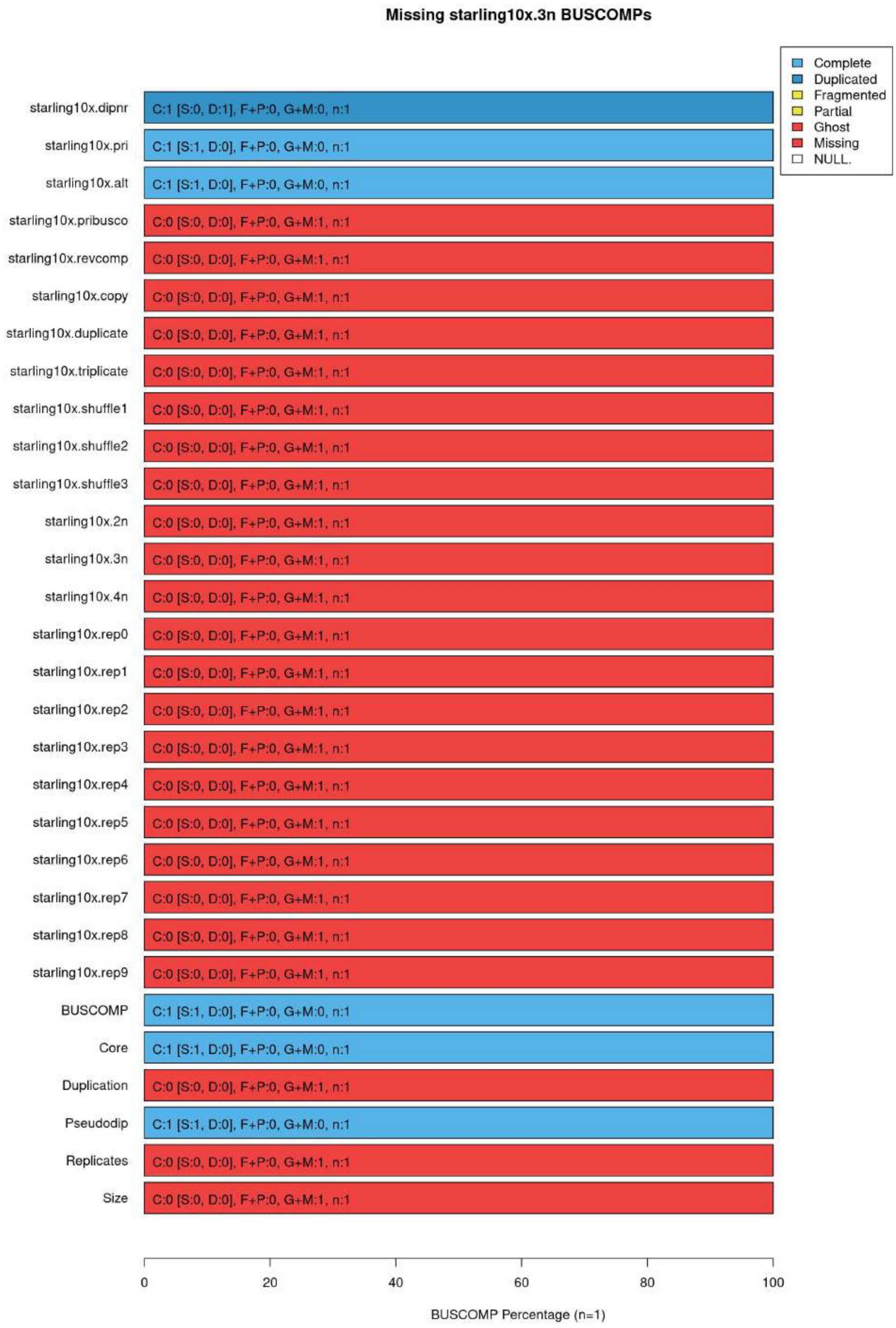

5.20 Missing starling10x.4n BUSCO genes

BUSCO ratings for `Missing` starling10x.4n BUSCO genes:

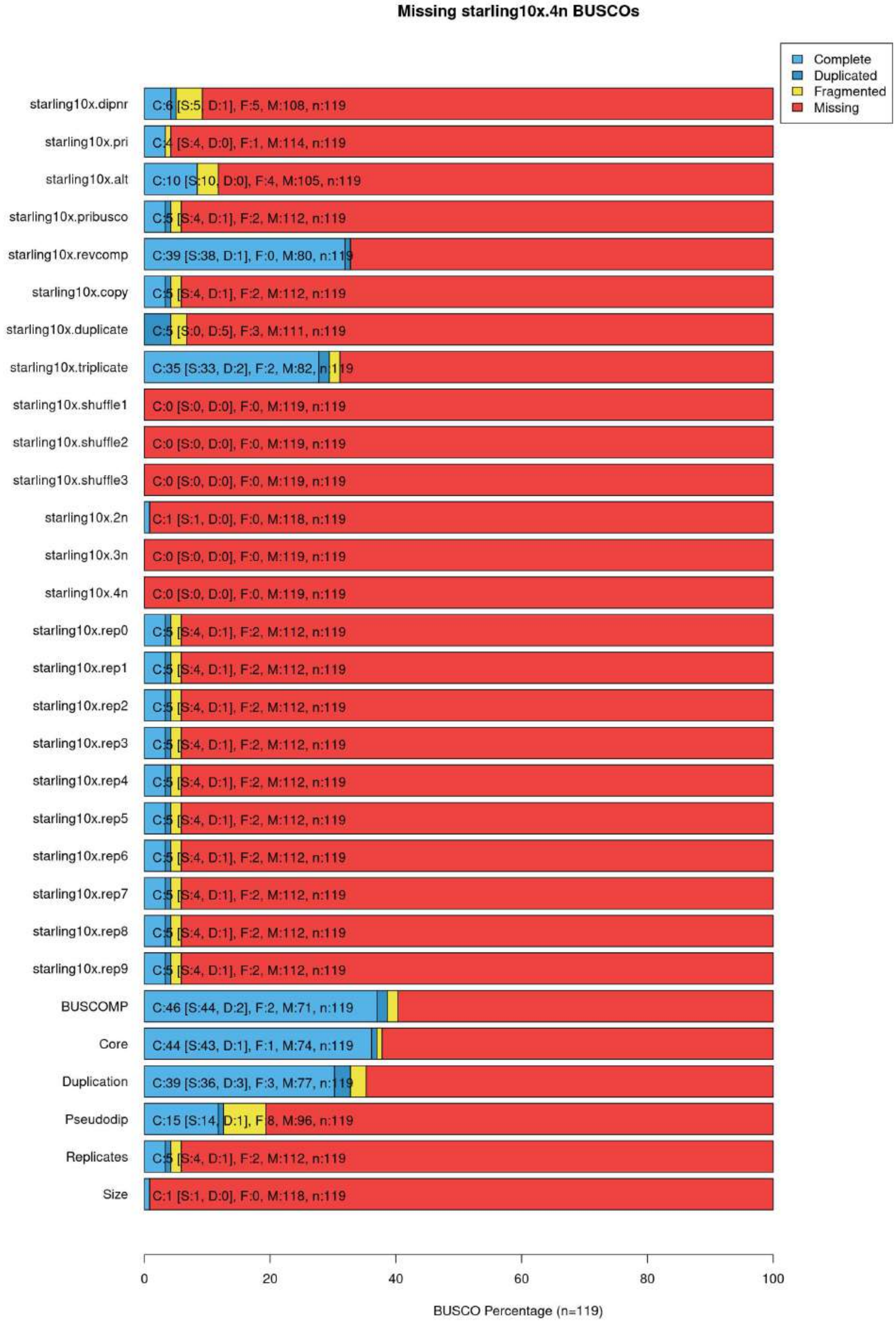

BUSCOMP ratings for `Missing` starling10x.4n BUSCO genes:

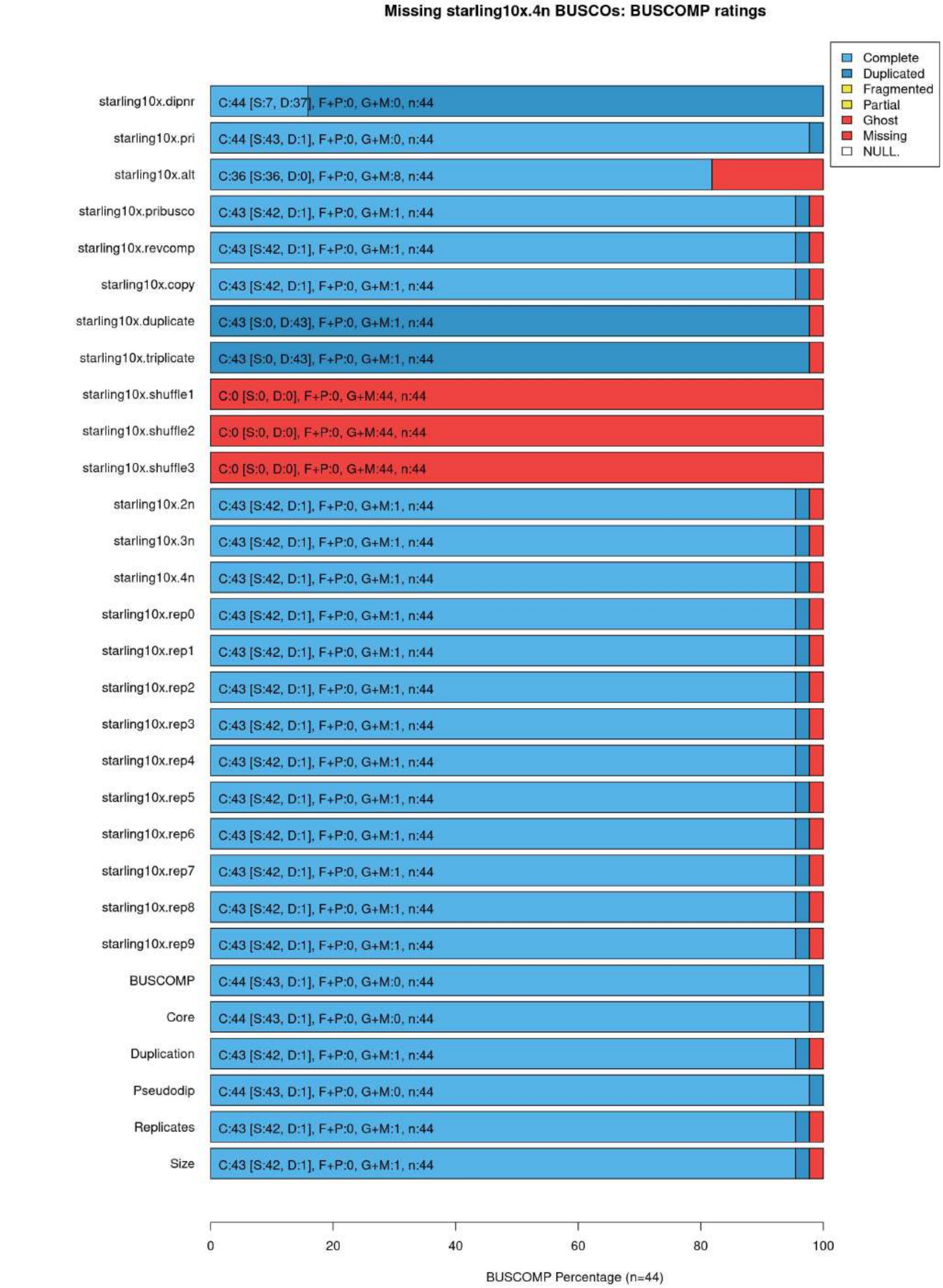

BUSCOMP ratings for `Missing` starling10x.4n BUSCOMP genes:

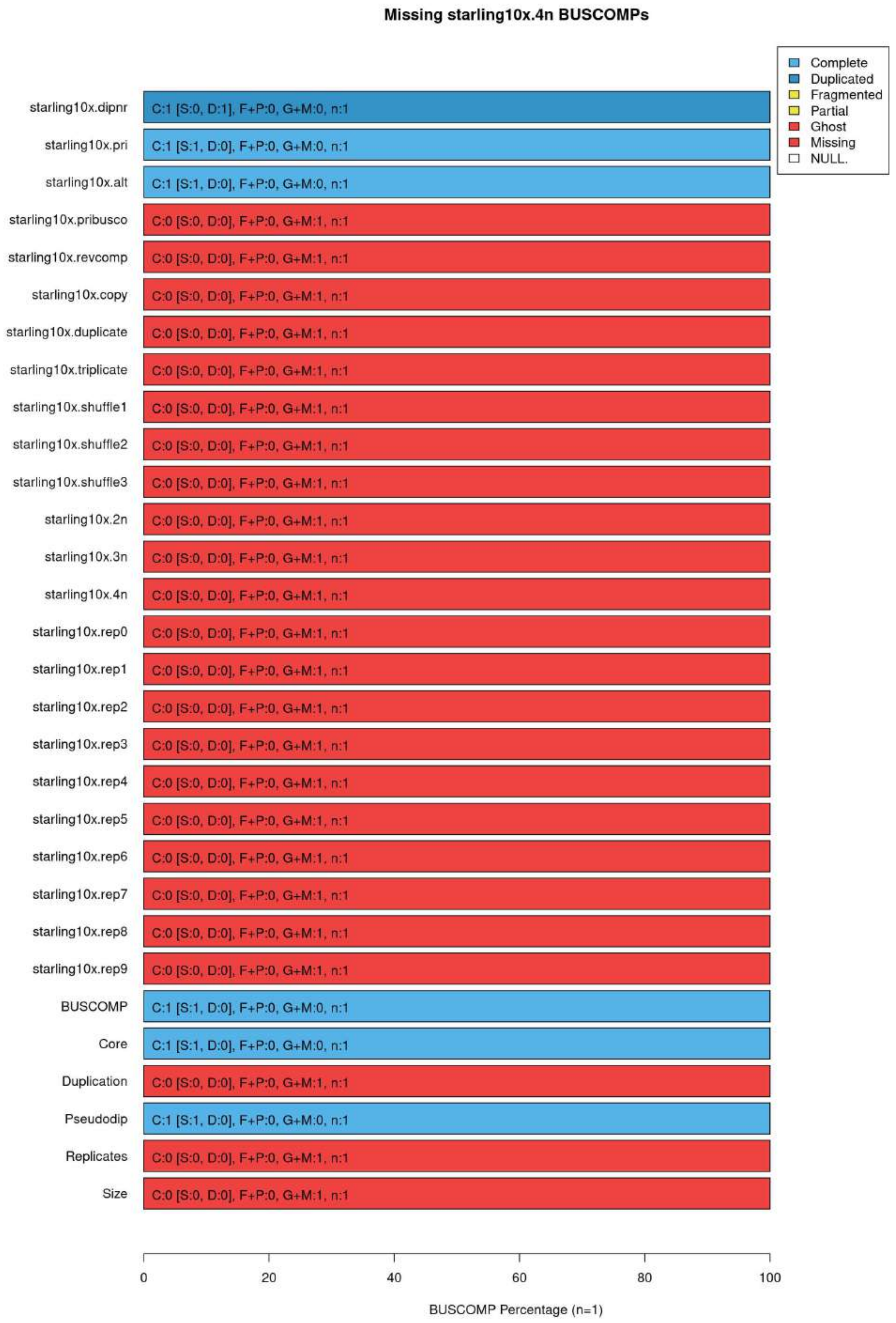

5.21 Missing Size BUSCO genes

BUSCO ratings for `Missing` Size BUSCO genes:

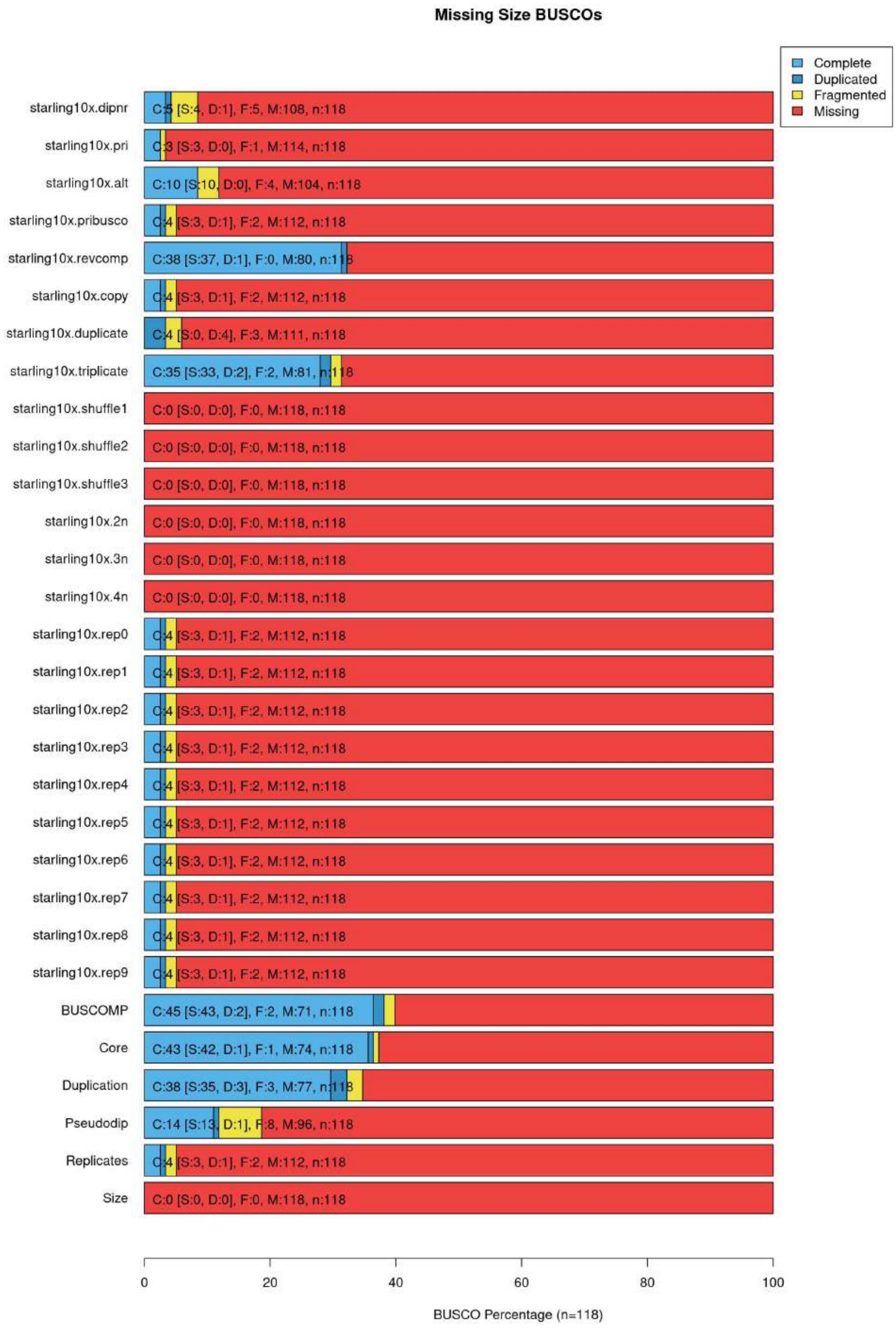

BUSCOMP ratings for `Missing` Size BUSCO genes:

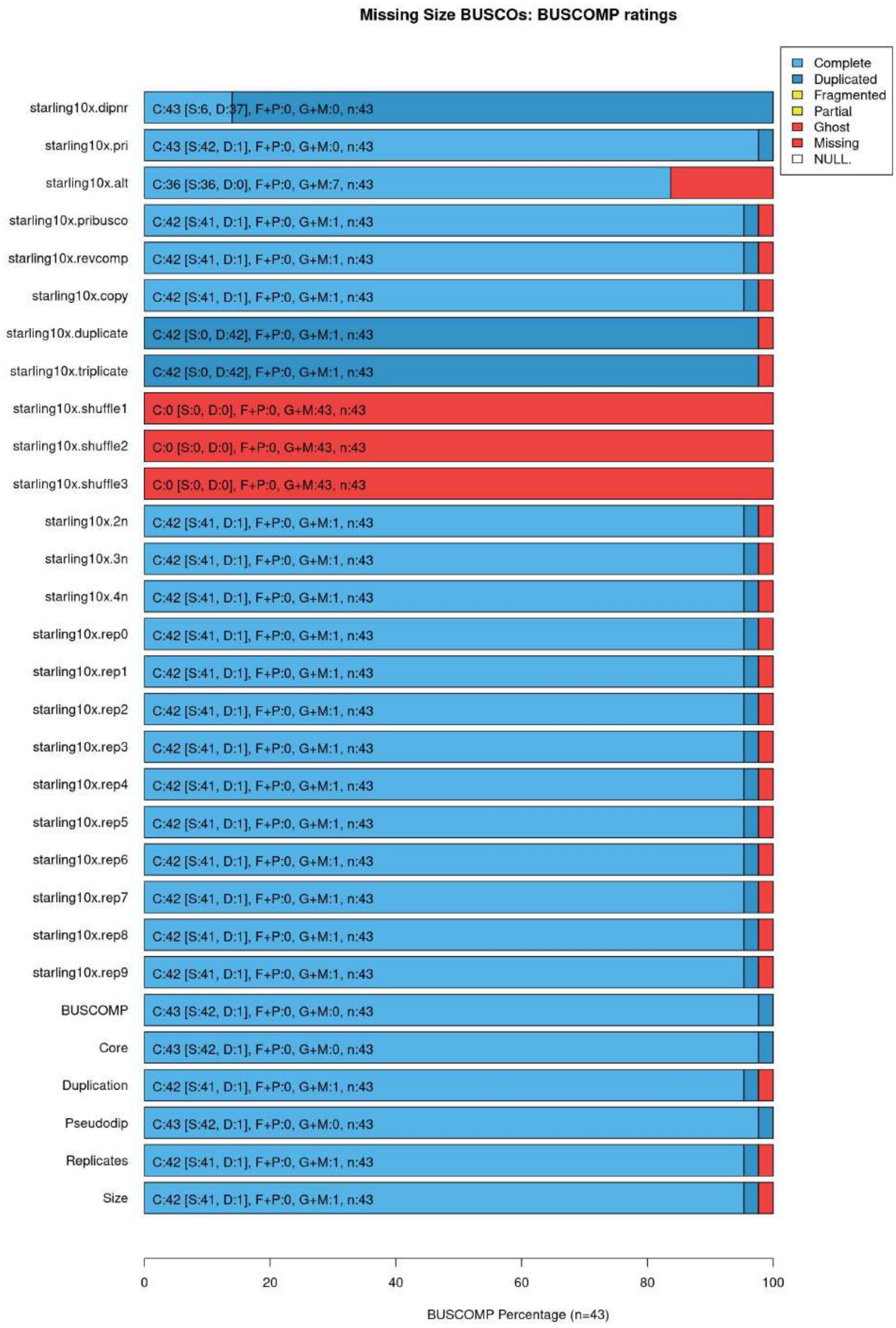

BUSCOMP ratings for `Missing` Size BUSCOMP genes:

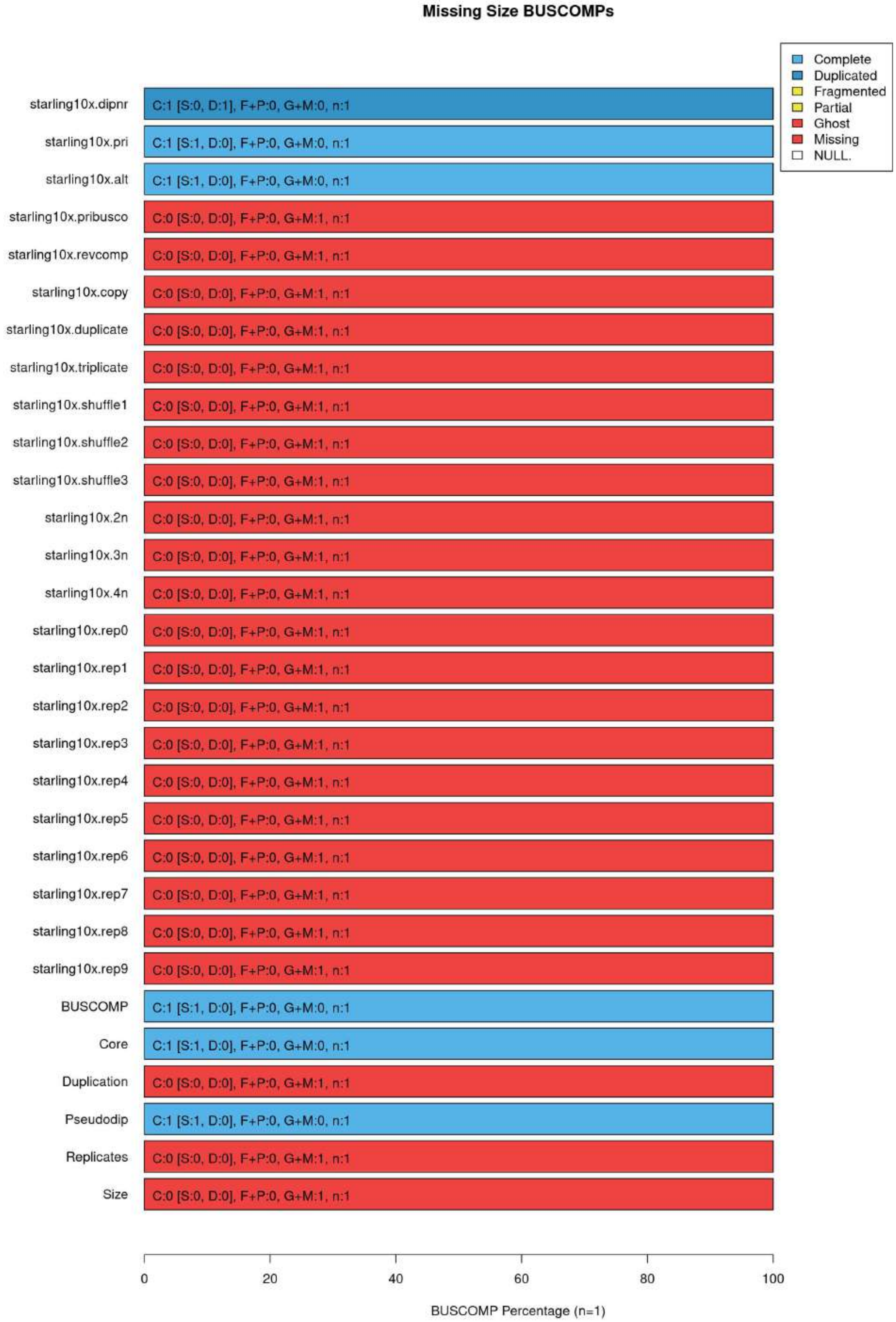

5.22 Missing starling10x.rep0 BUSCO genes

BUSCO ratings for `Missing` starling10x.rep0 BUSCO genes:

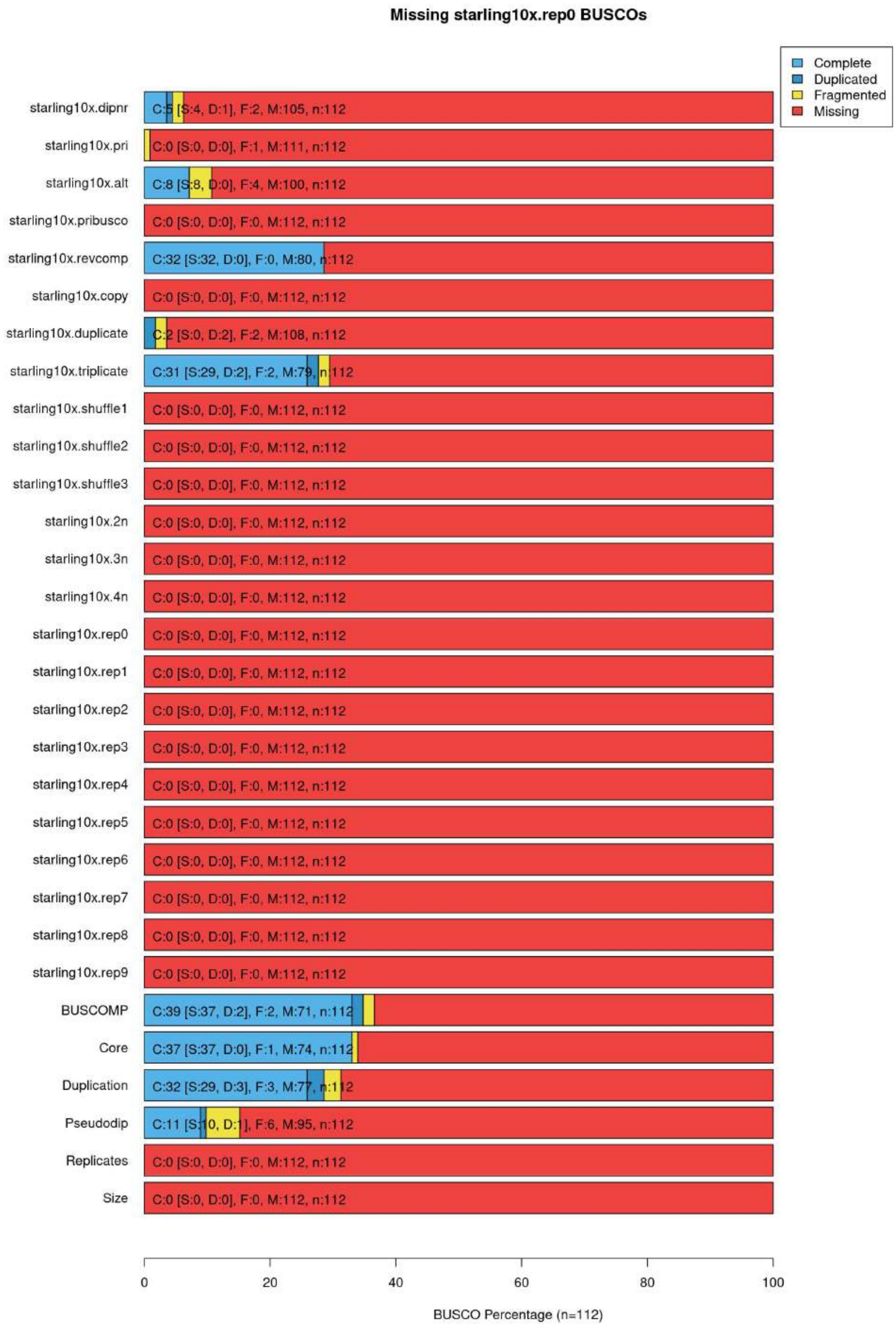

BUSCOMP ratings for `Missing` starling10x.rep0 BUSCO genes:

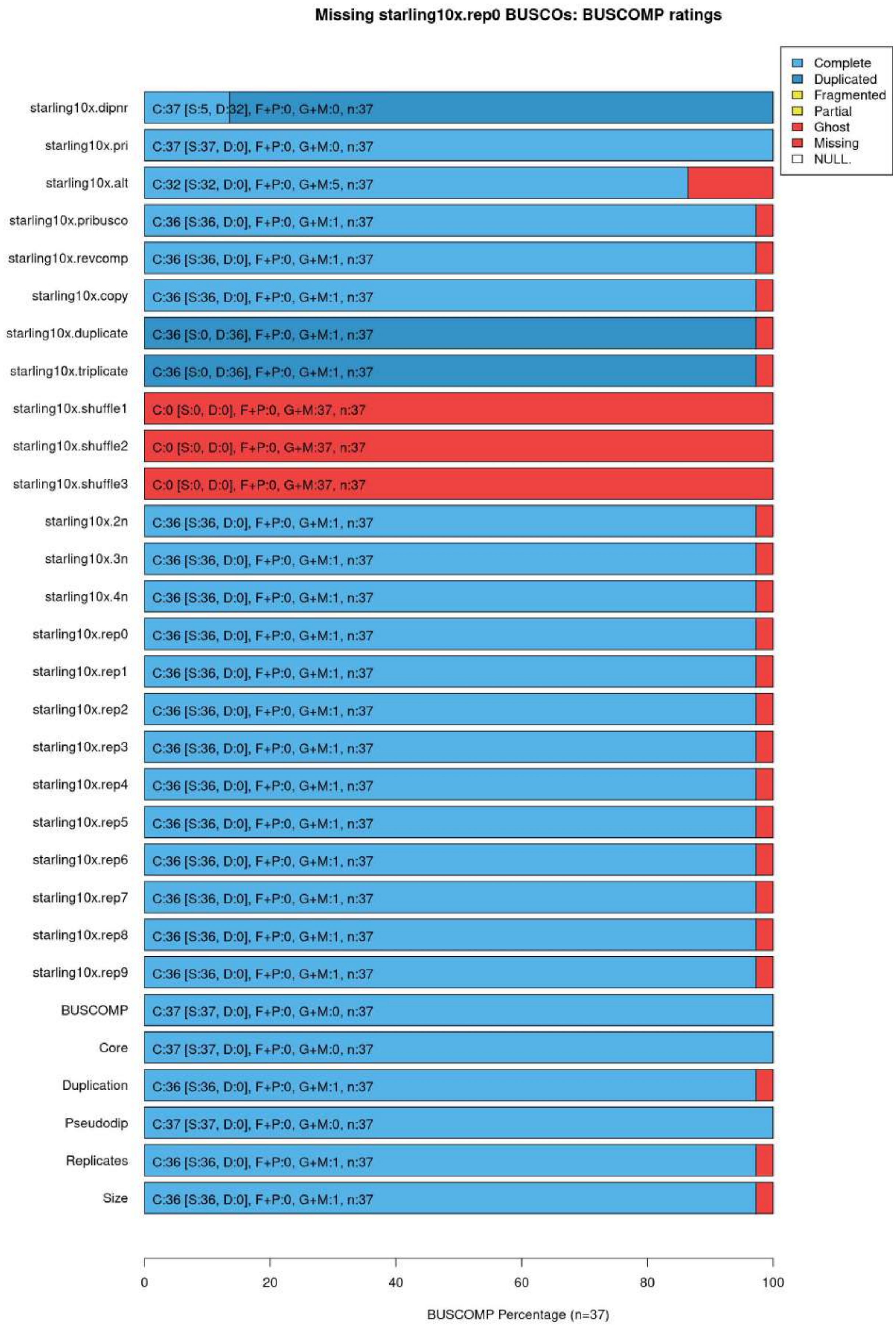

BUSCOMP ratings for `Missing` starling10x.rep0 BUSCOMP genes:

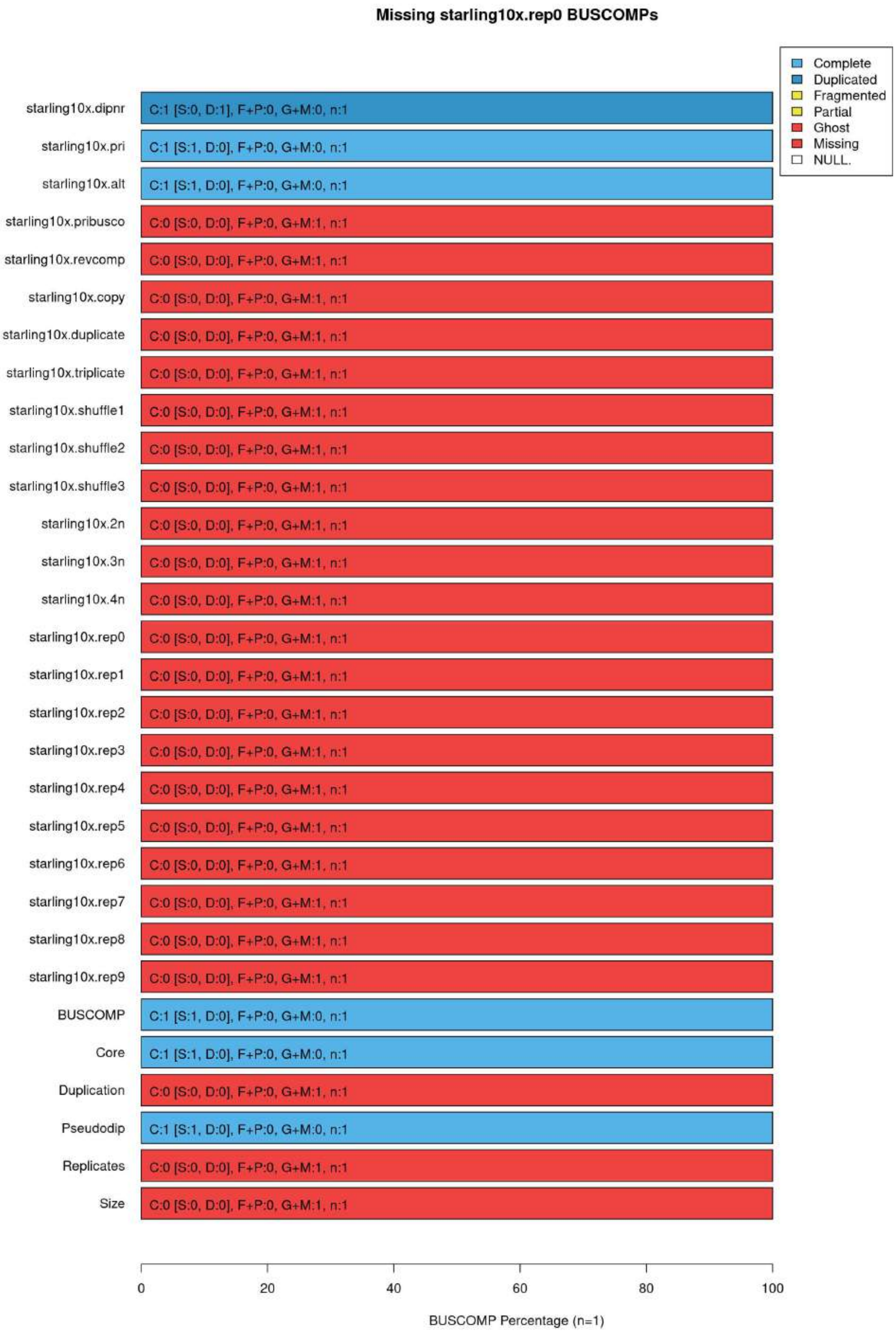

5.23 Missing starling10x.rep1 BUSCO genes

BUSCO ratings for `Missing` starling10x.rep1 BUSCO genes:

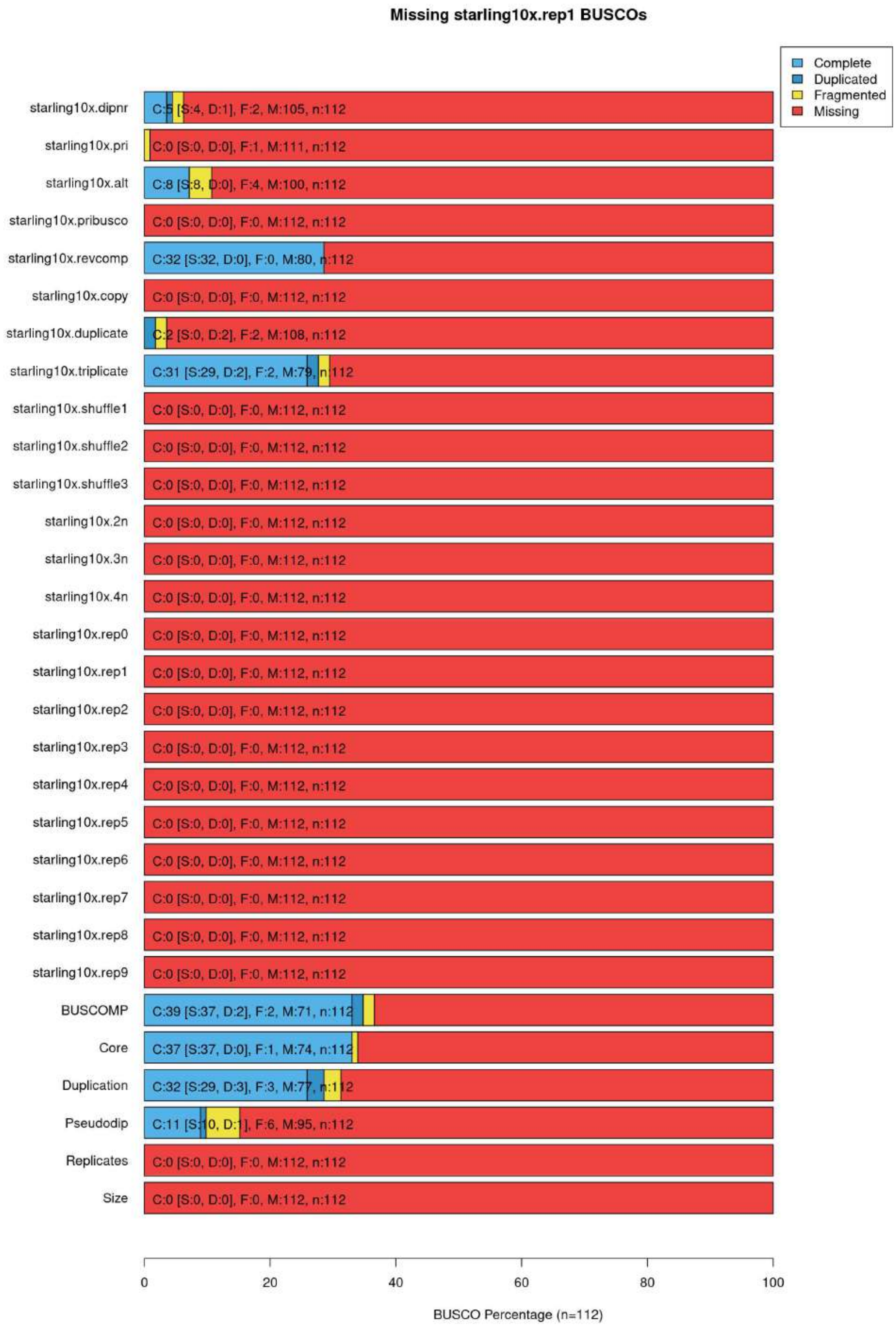

BUSCOMP ratings for `Missing` starling10x.rep1 BUSCO genes:

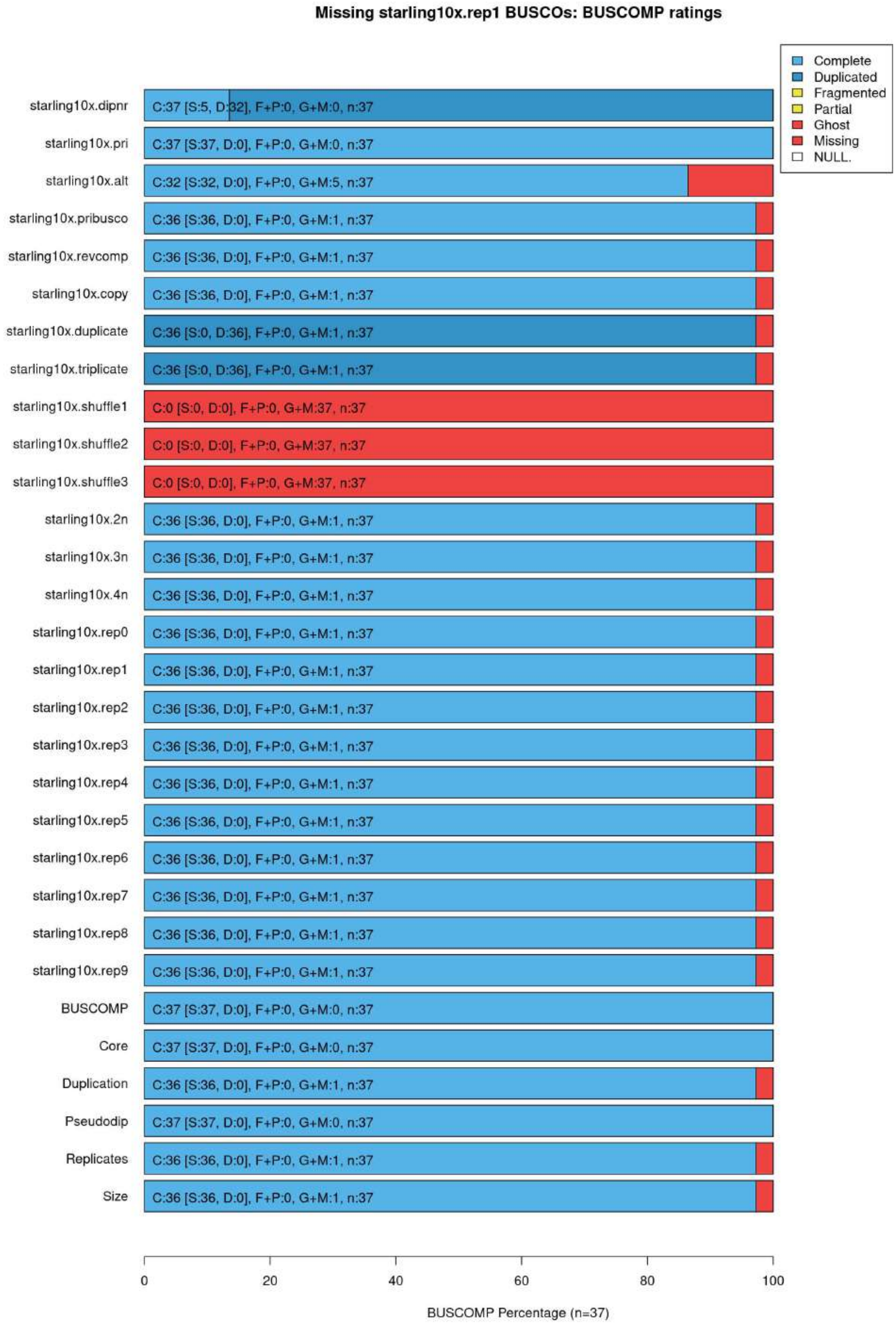

BUSCOMP ratings for `Missing` starling10x.rep1 BUSCOMP genes:

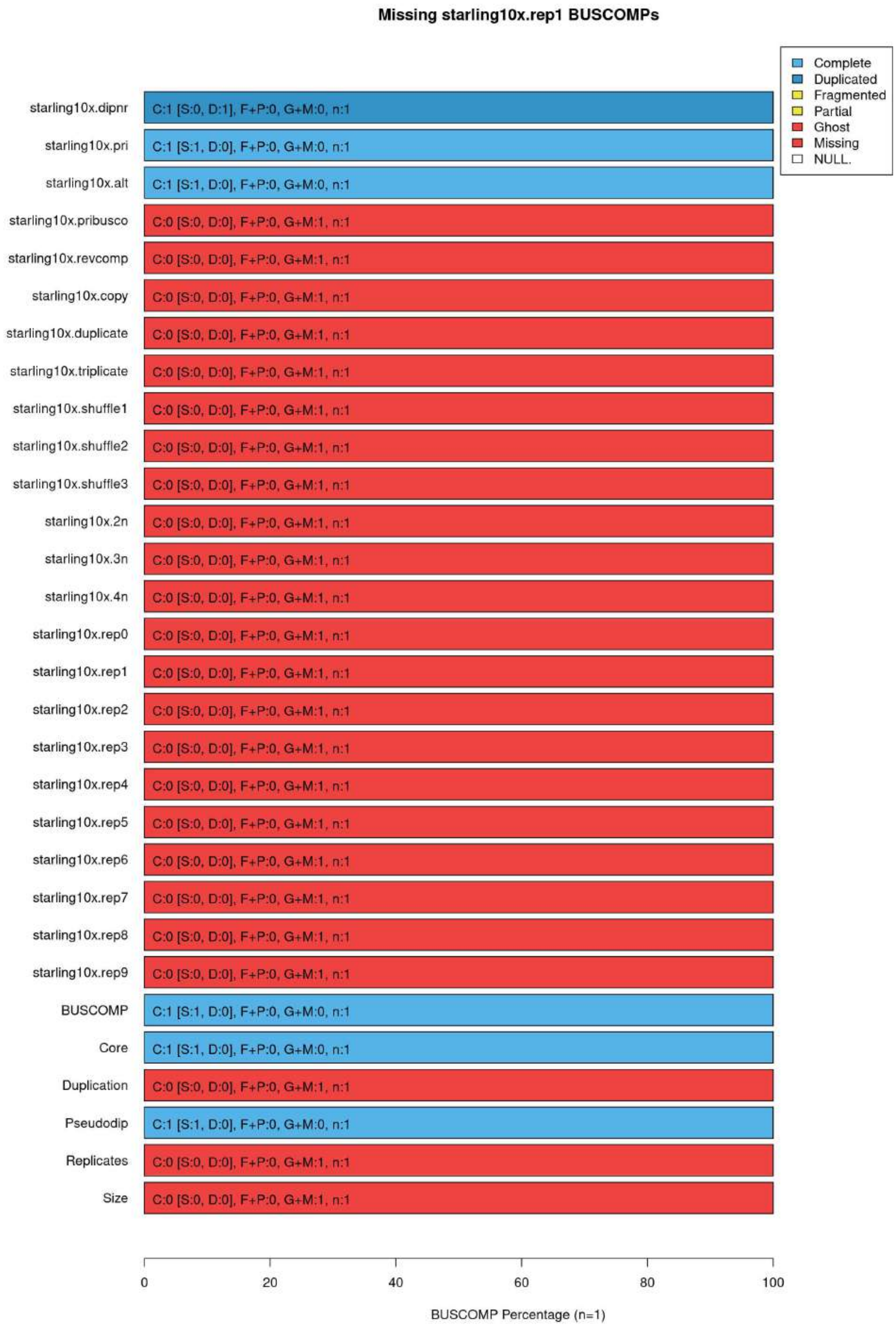

5.24 Missing starling10x.rep2 BUSCO genes

BUSCO ratings for `Missing` starling10x.rep2 BUSCO genes:

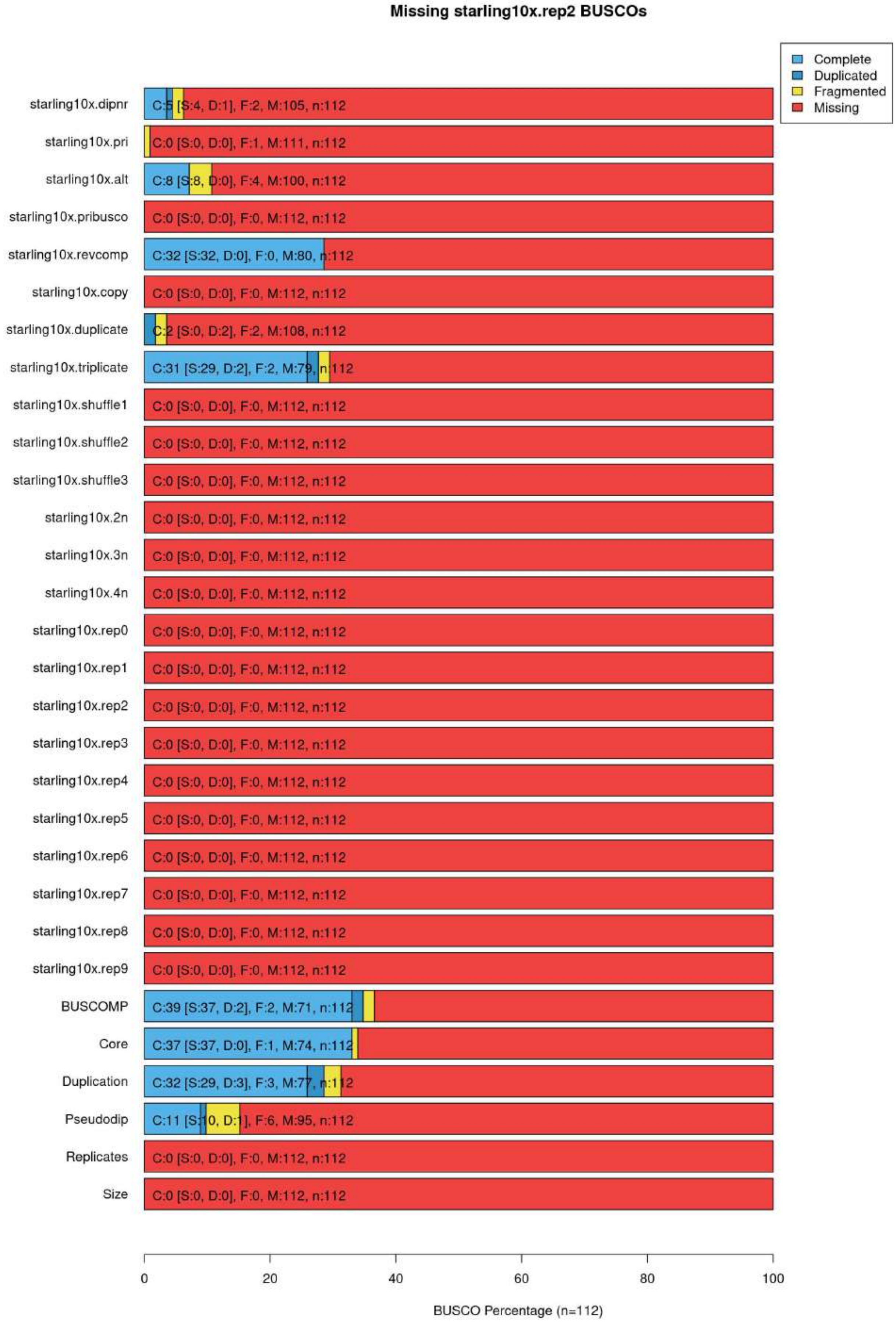

BUSCOMP ratings for `Missing` starling10x.rep2 BUSCO genes:

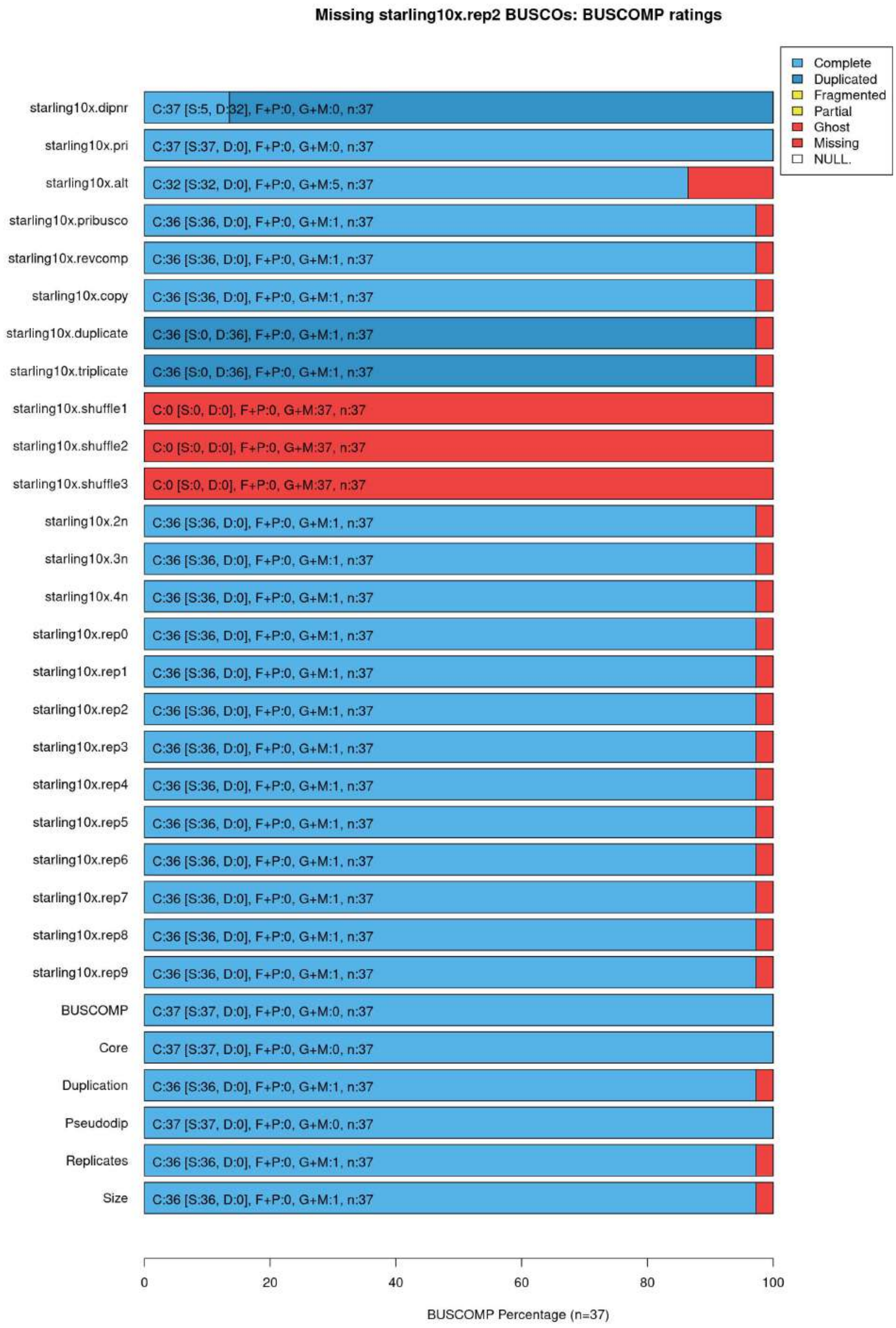

BUSCOMP ratings for `Missing` starling10x.rep2 BUSCOMP genes:

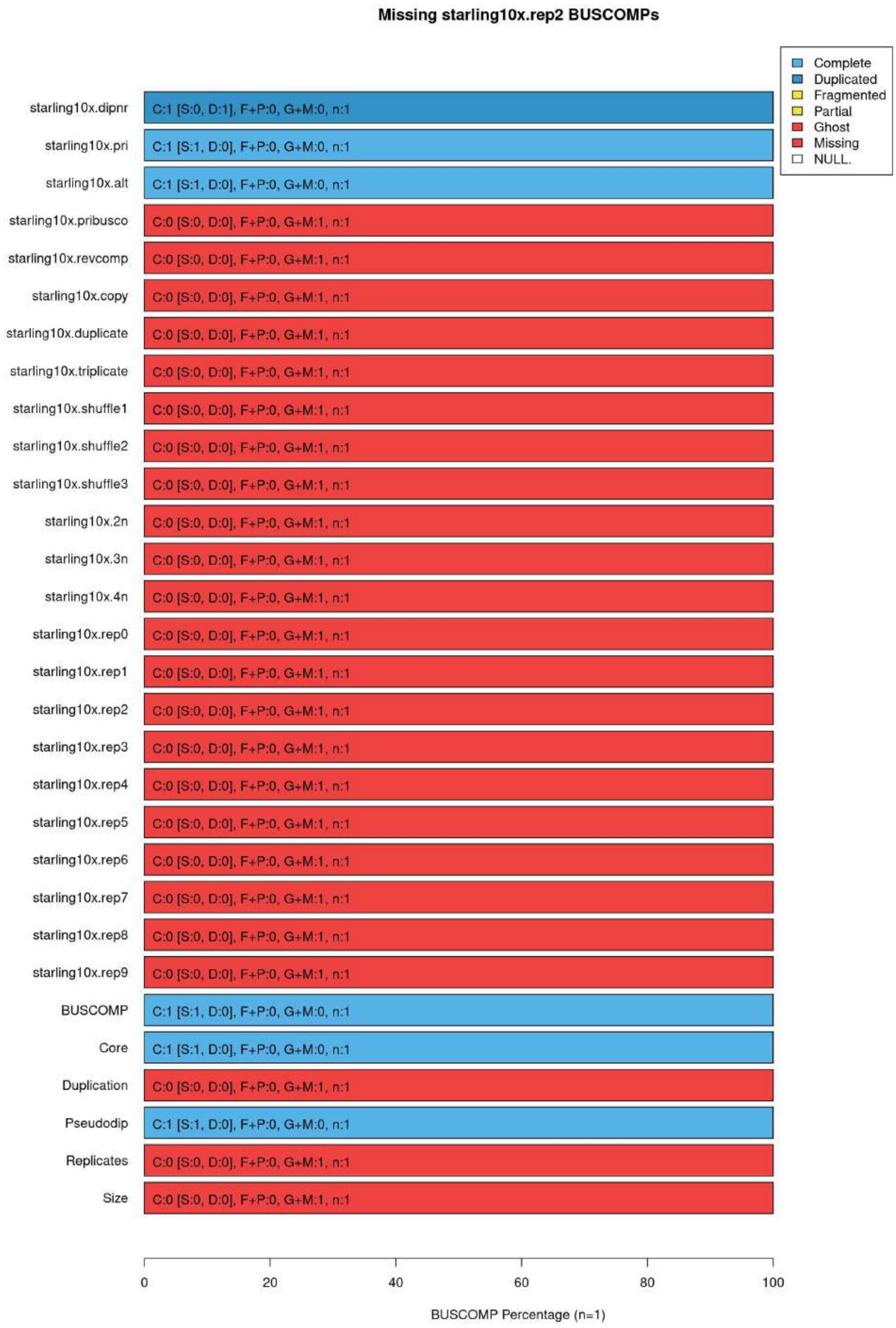

5.25 Missing starling10x.rep3 BUSCO genes

BUSCO ratings for `Missing` starling10x.rep3 BUSCO genes:

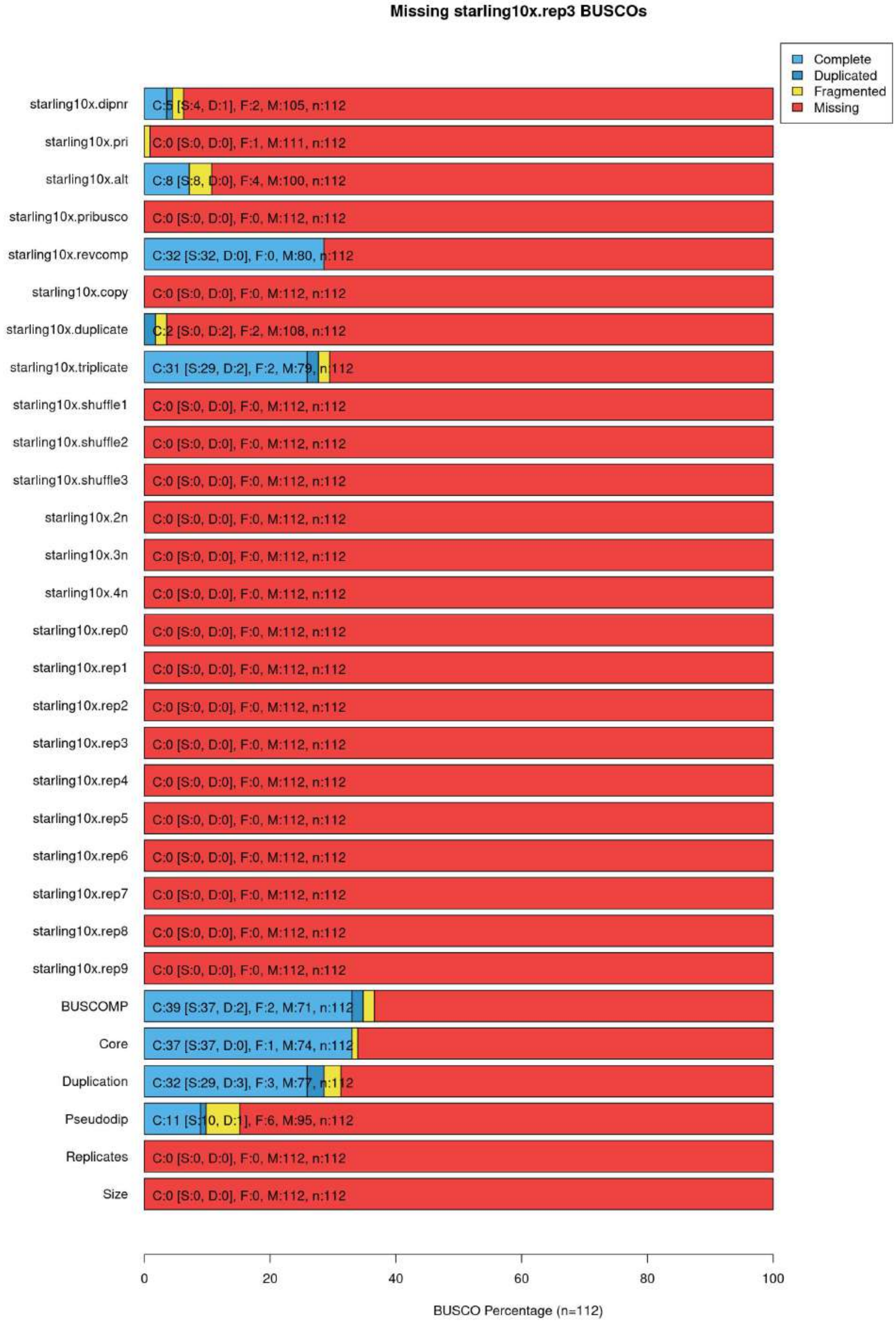

BUSCOMP ratings for `Missing` starling10x.rep3 BUSCO genes:

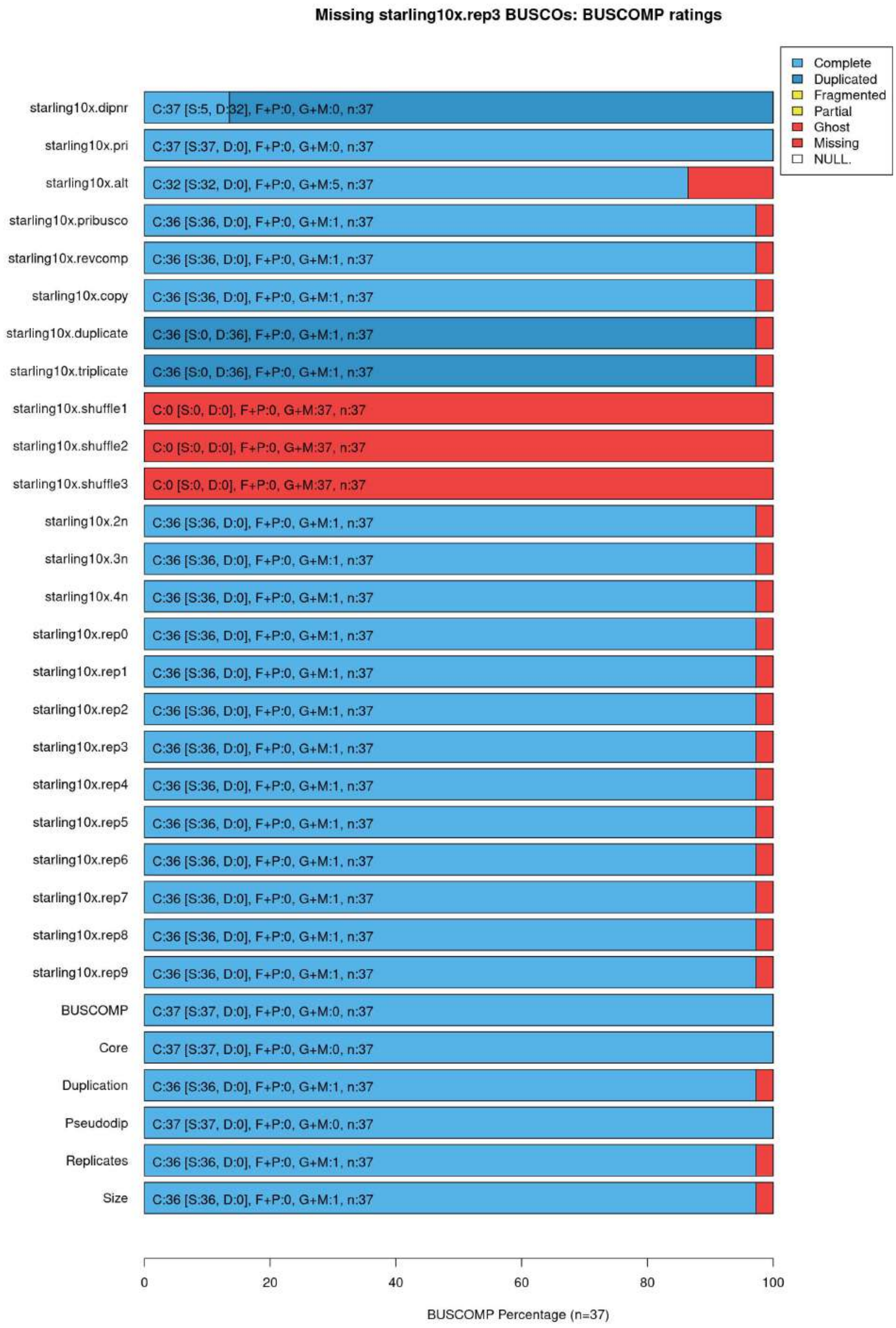

BUSCOMP ratings for `Missing` starling10x.rep3 BUSCOMP genes:

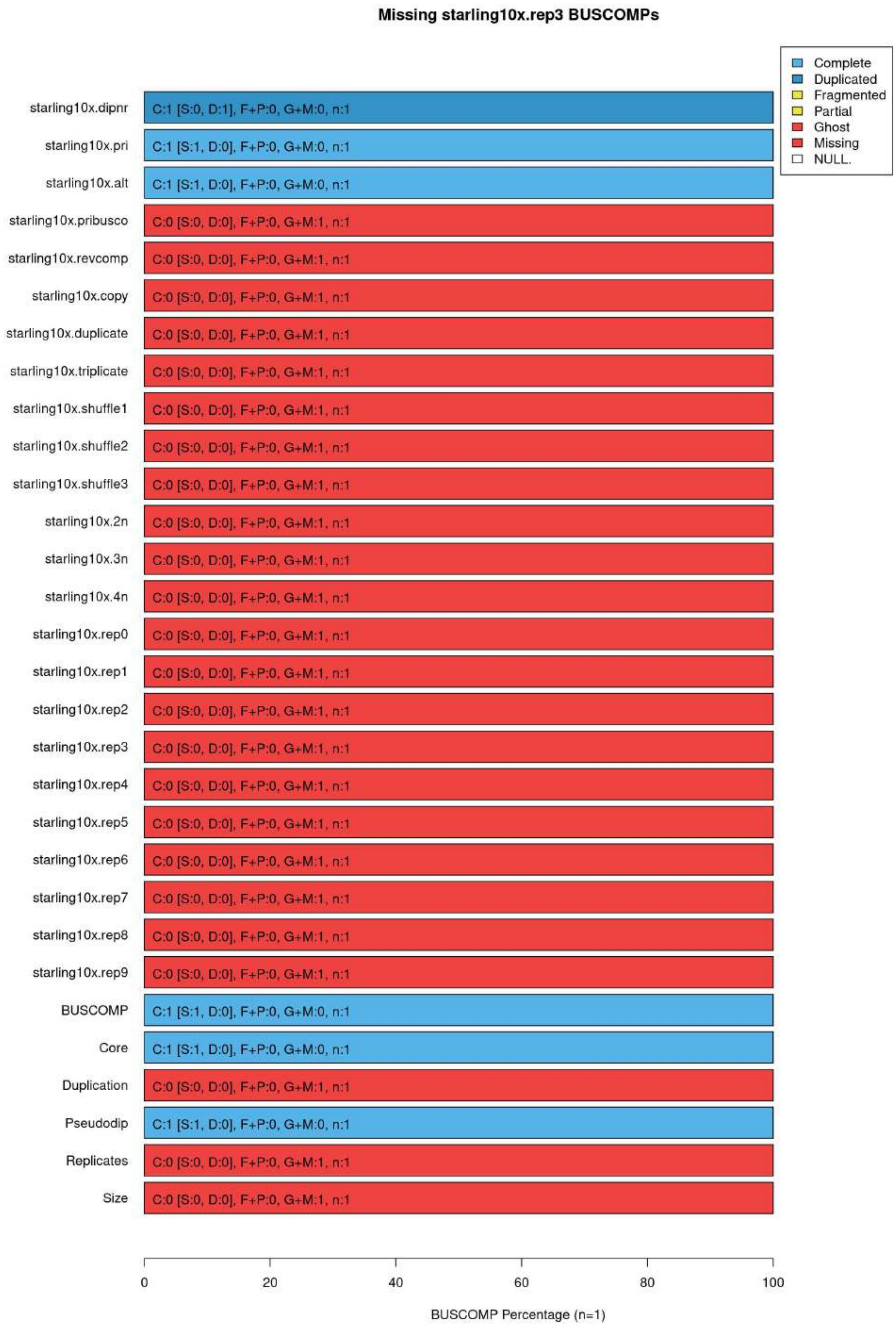

5.26 Missing starling10x.rep4 BUSCO genes

BUSCO ratings for `Missing` starling10x.rep4 BUSCO genes:

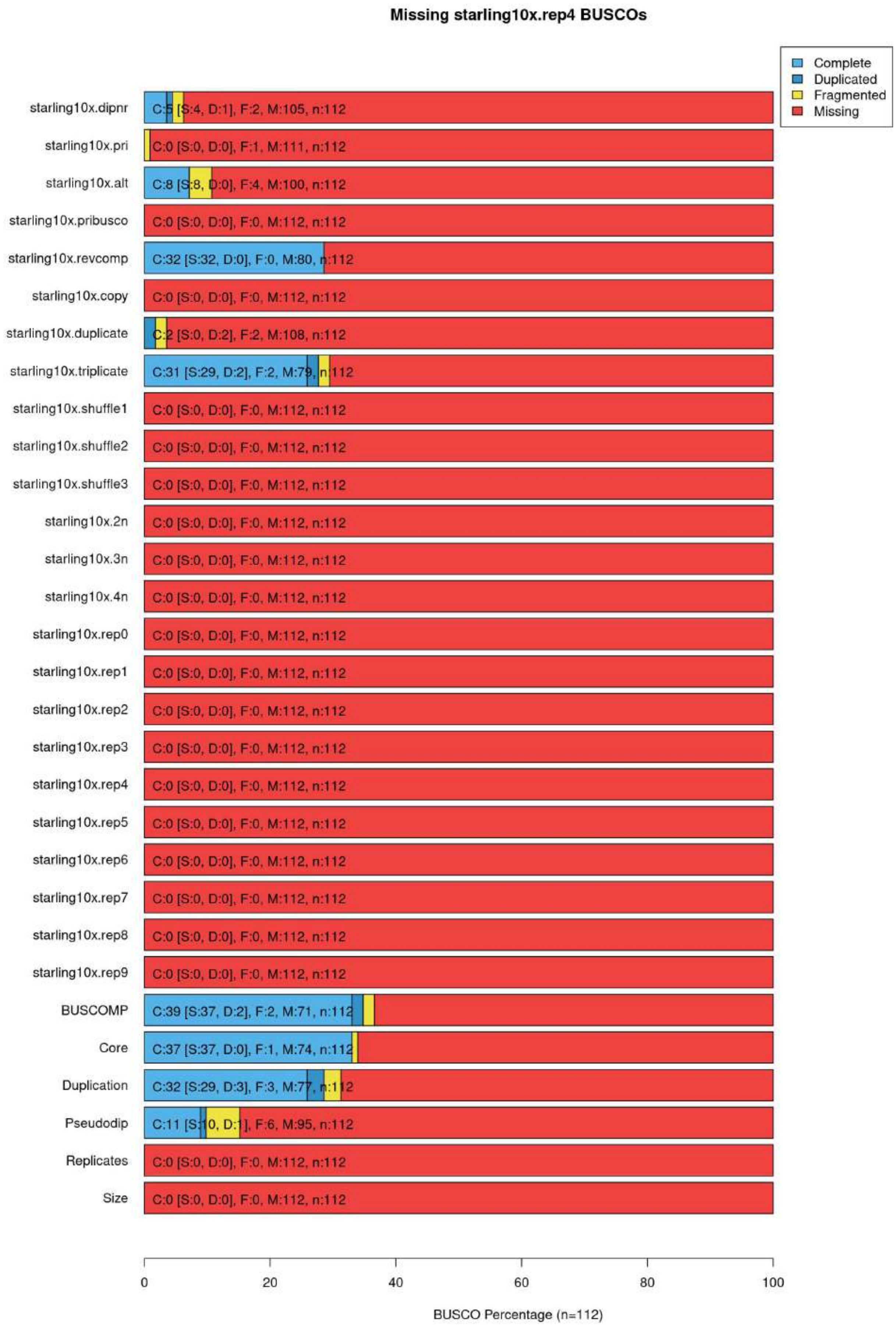

BUSCOMP ratings for `Missing` starling10x.rep4 BUSCO genes:

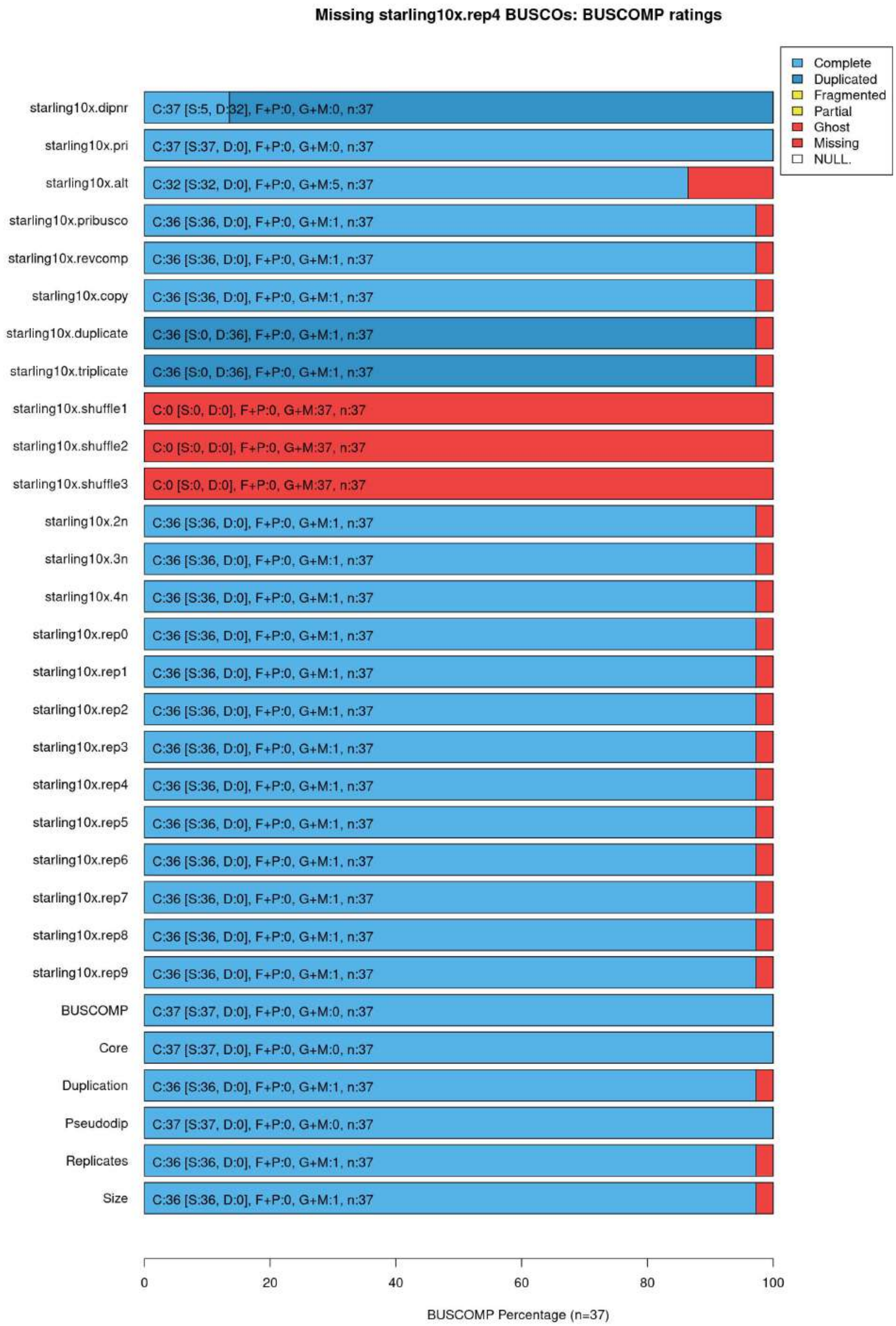

BUSCOMP ratings for `Missing` starling10x.rep4 BUSCOMP genes:

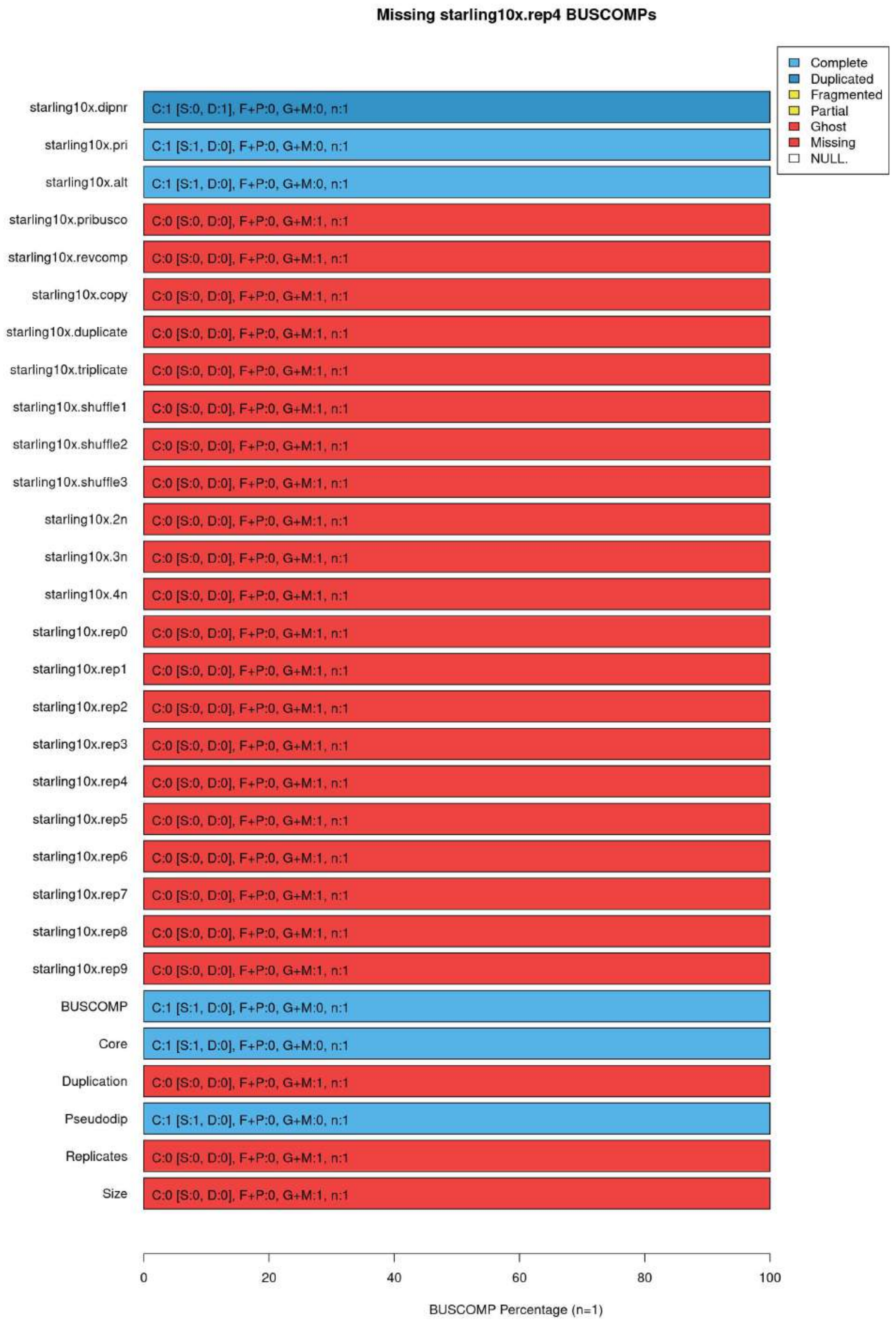

5.27 Missing starling10x.rep5 BUSCO genes

BUSCO ratings for `Missing` starling10x.rep5 BUSCO genes:

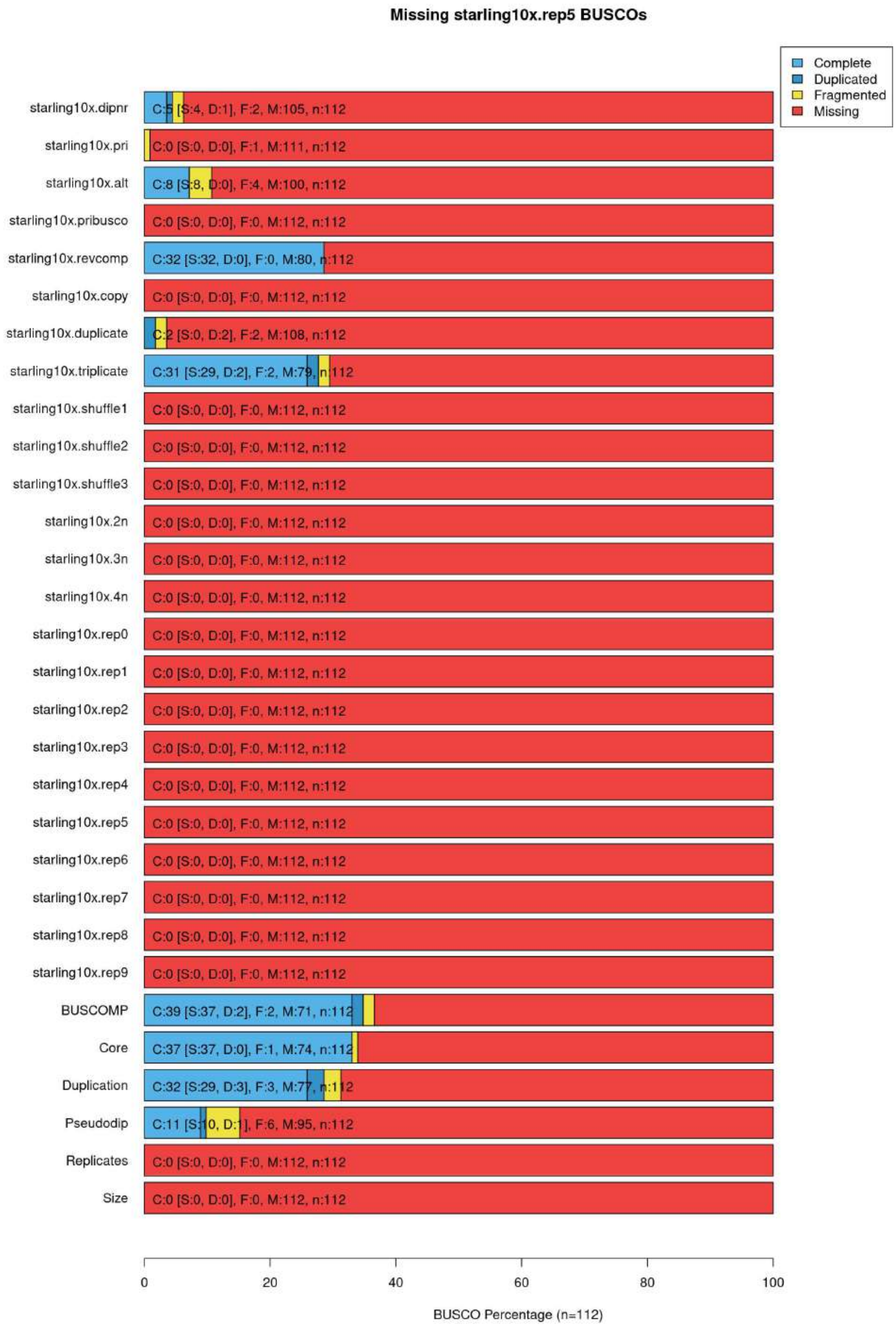

BUSCOMP ratings for `Missing` starling10x.rep5 BUSCO genes:

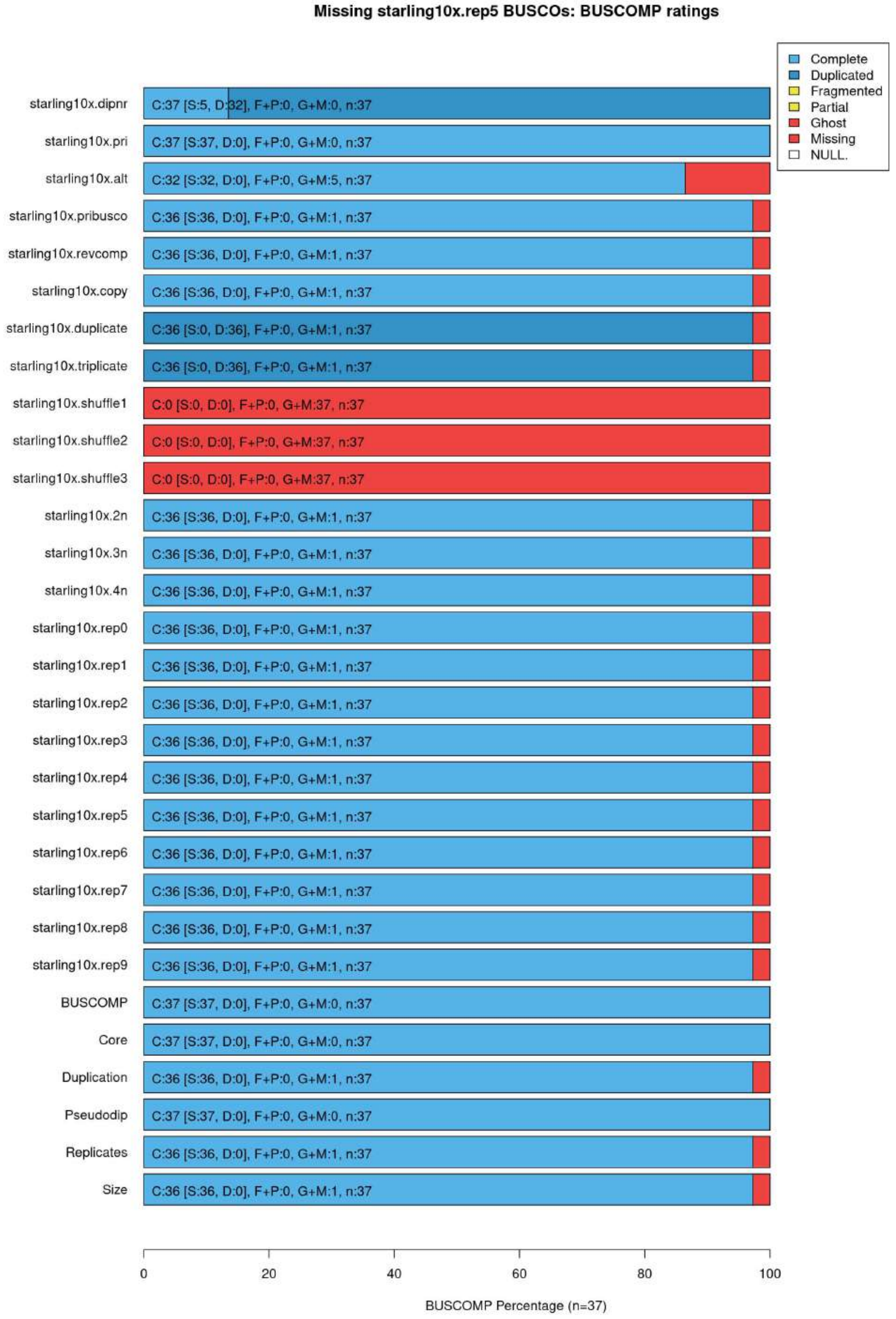

BUSCOMP ratings for `Missing` starling10x.rep5 BUSCOMP genes:

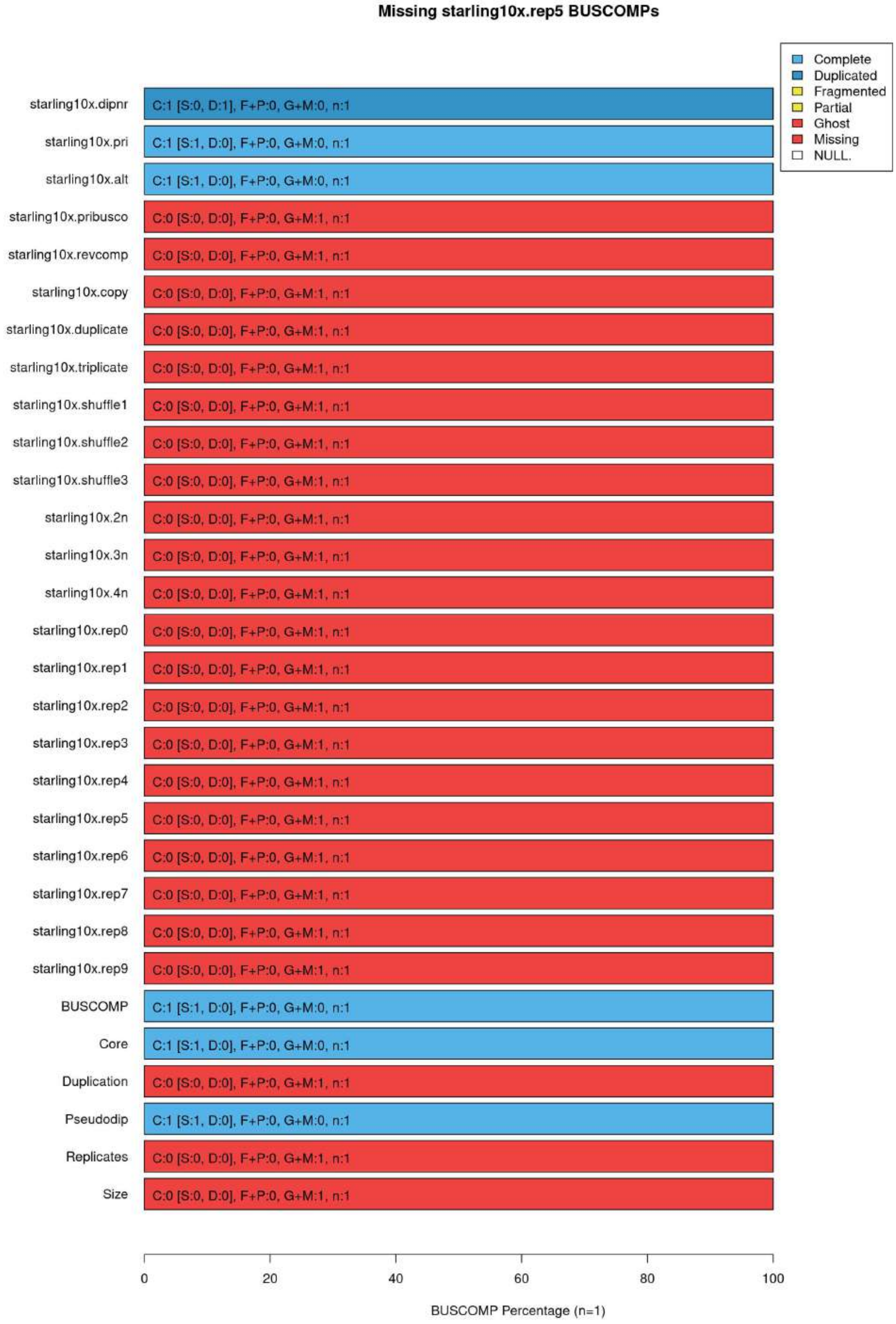

5.28 Missing starling10x.rep6 BUSCO genes

BUSCO ratings for `Missing` starling10x.rep6 BUSCO genes:

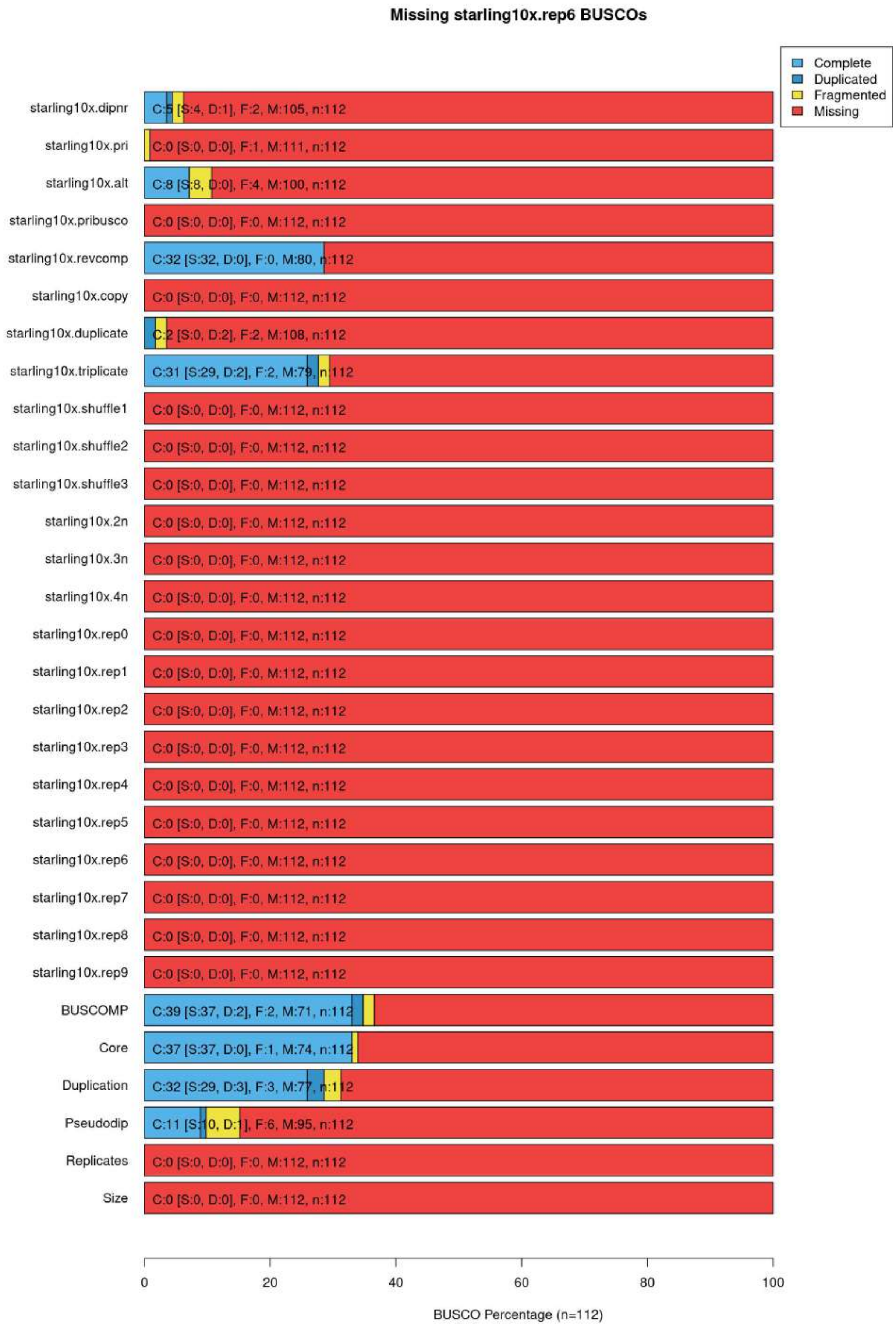

BUSCOMP ratings for `Missing` starling10x.rep6 BUSCO genes:

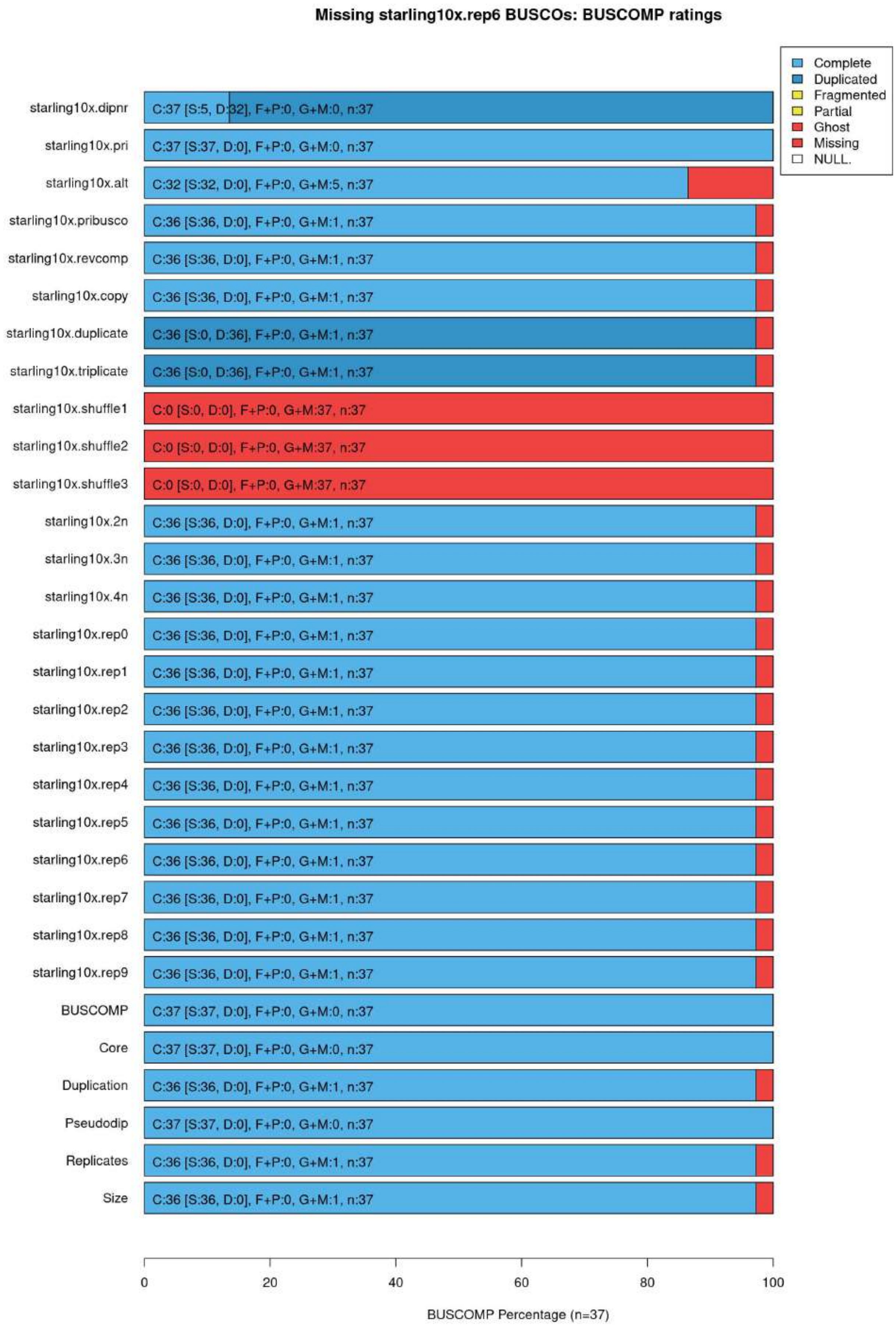

BUSCOMP ratings for `Missing` starling10x.rep6 BUSCOMP genes:

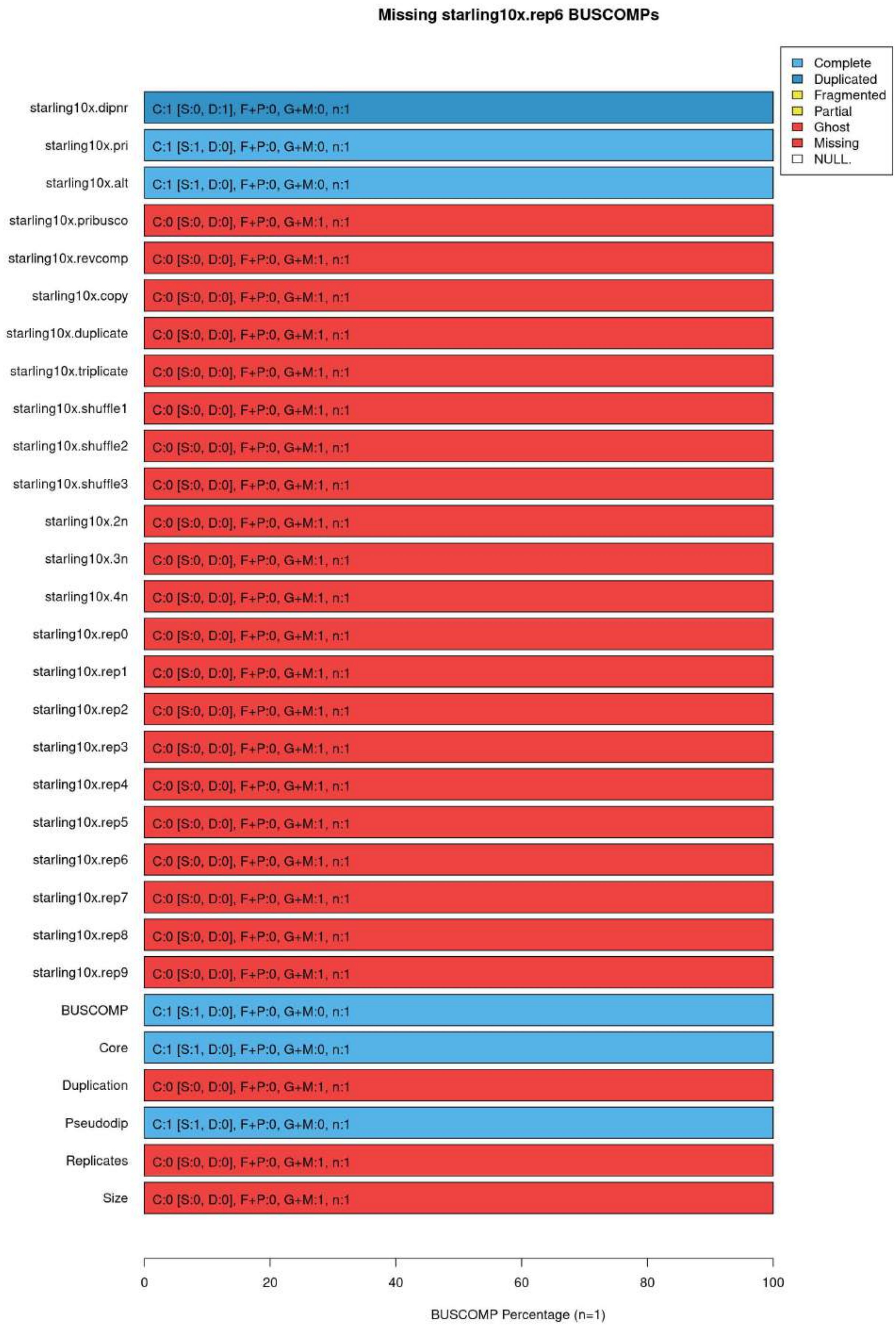

5.29 Missing starling10x.rep7 BUSCO genes

BUSCO ratings for `Missing` starling10x.rep7 BUSCO genes:

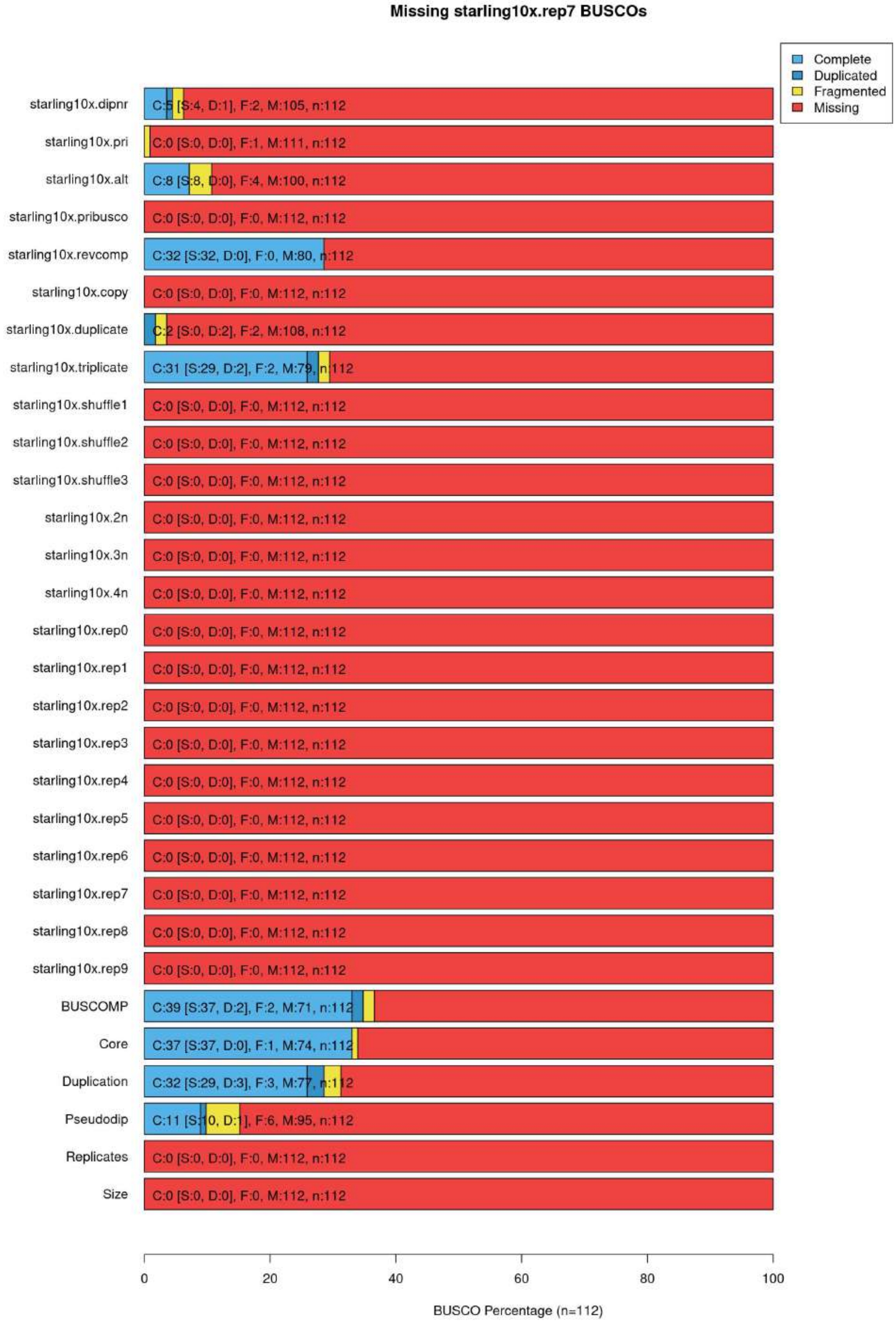

BUSCOMP ratings for `Missing` starling10x.rep7 BUSCO genes:

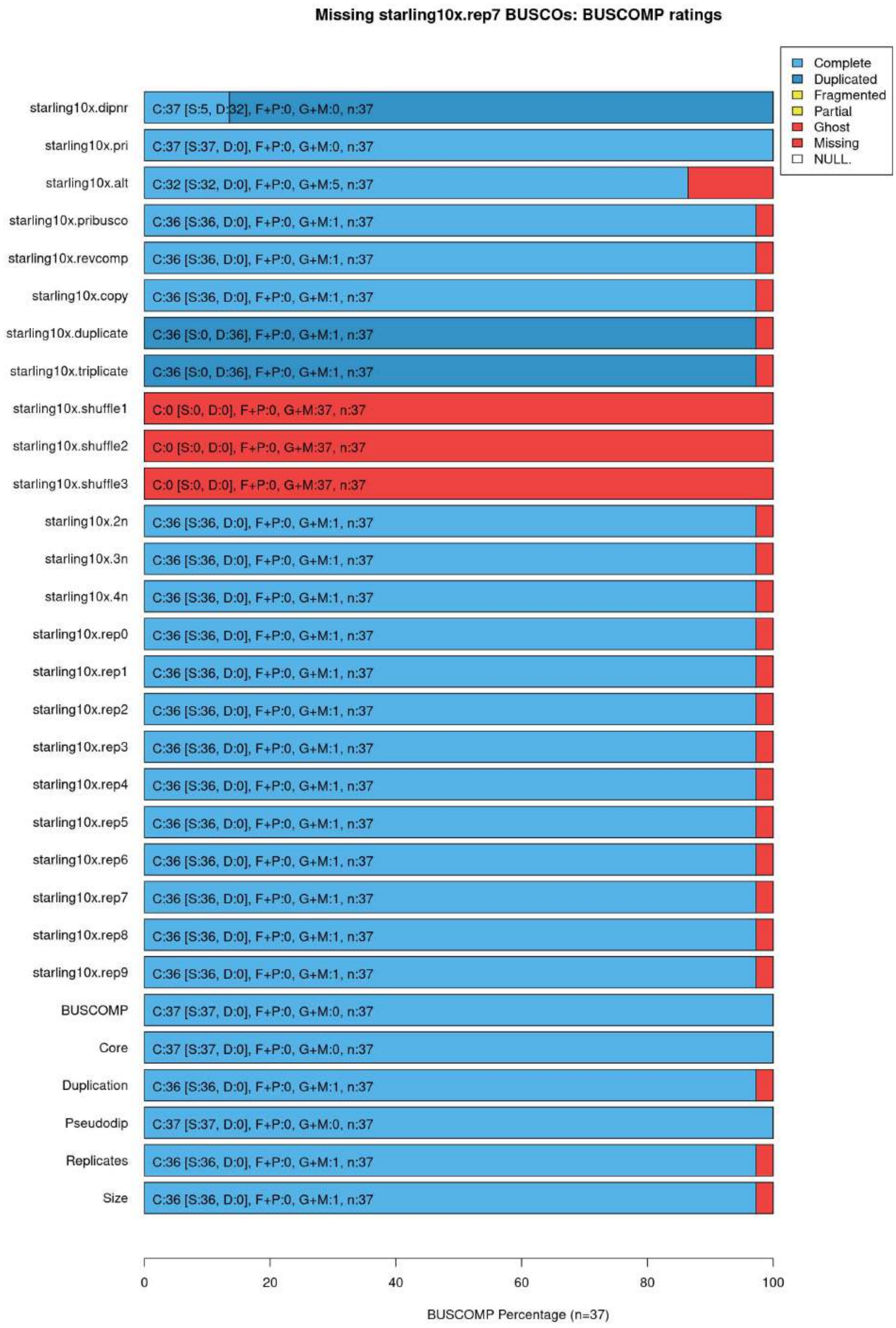

BUSCOMP ratings for `Missing` starling10x.rep7 BUSCOMP genes:

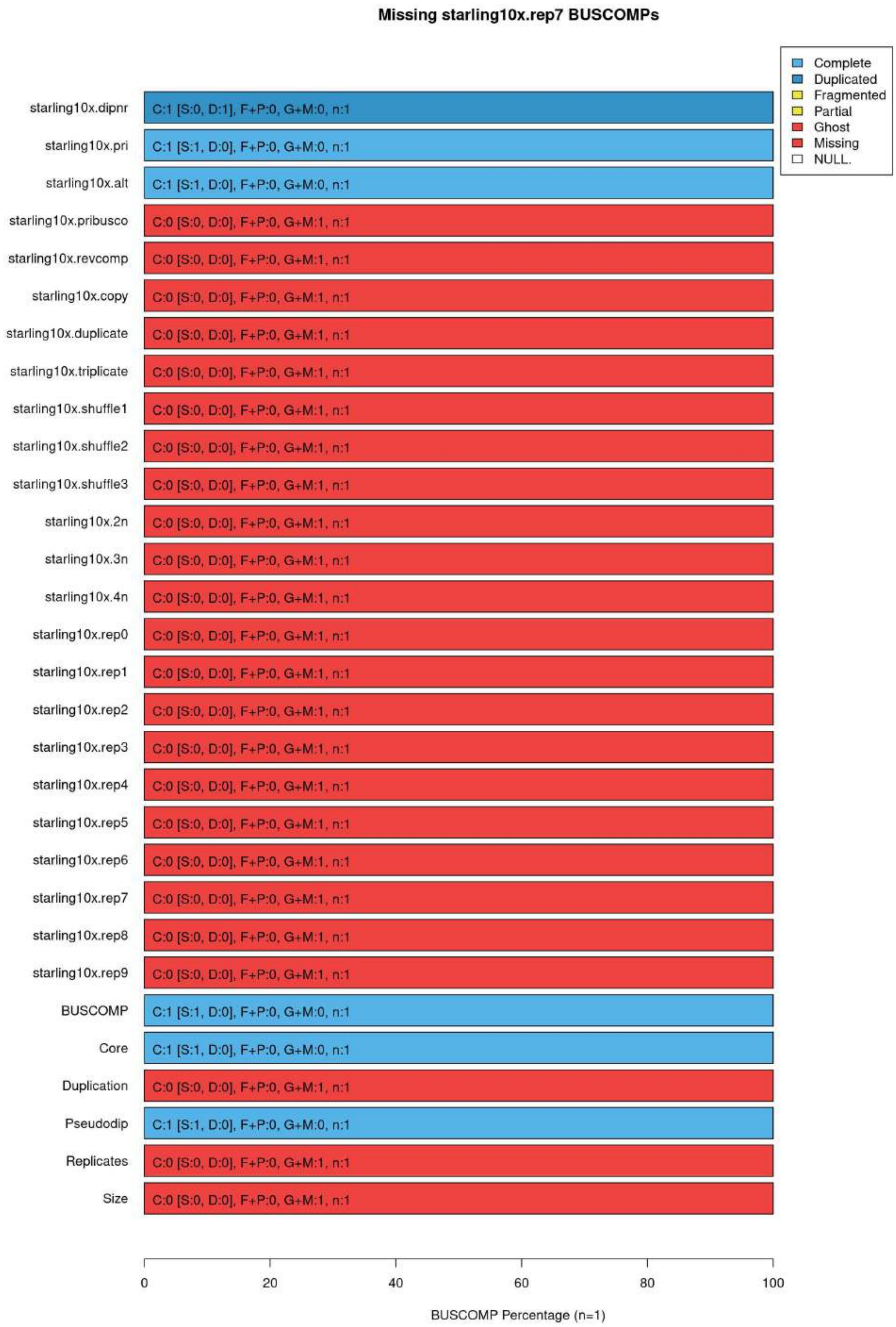

5.30 Missing starling10x.rep8 BUSCO genes

BUSCO ratings for `Missing` starling10x.rep8 BUSCO genes:

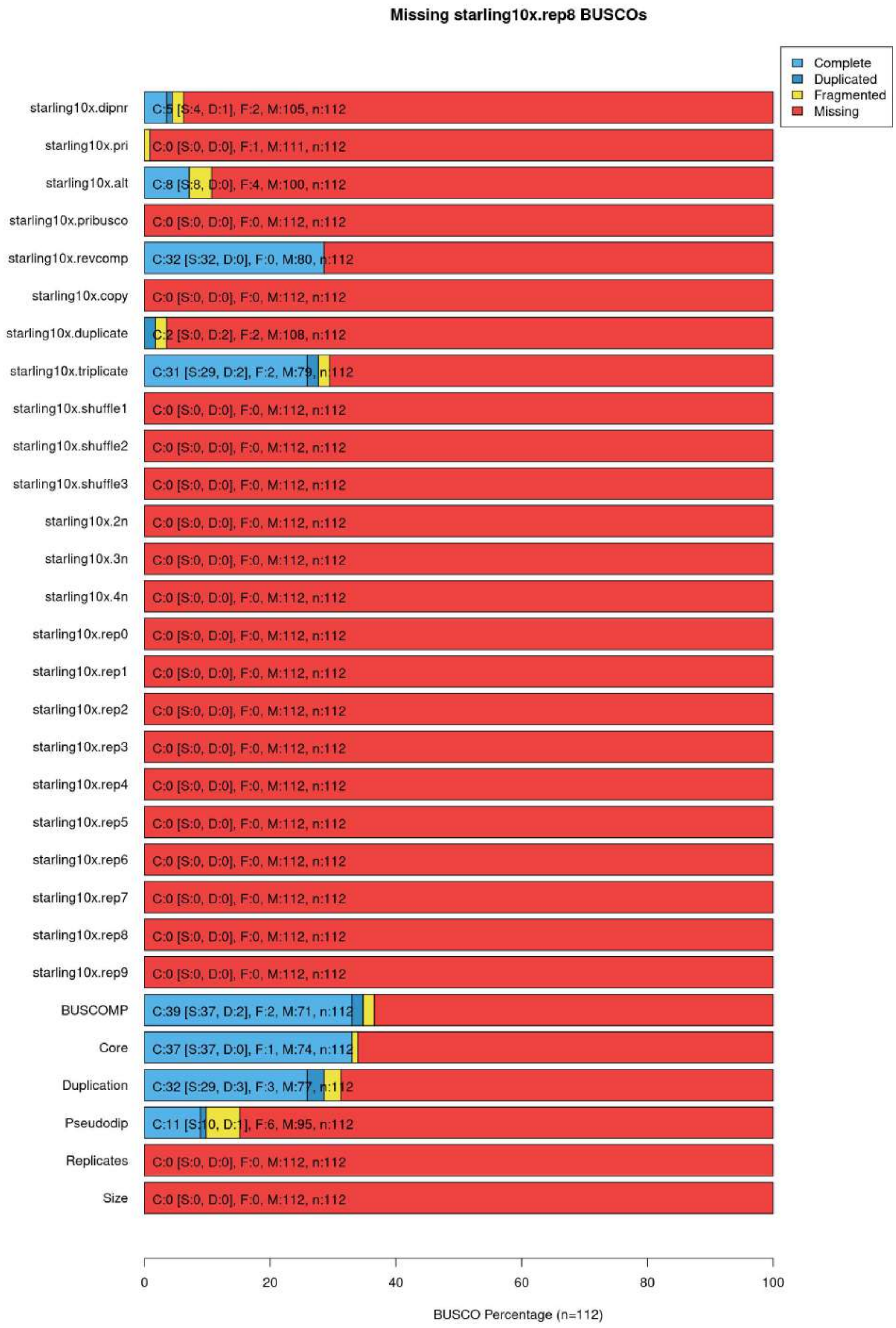

BUSCOMP ratings for `Missing` starling10x.rep8 BUSCO genes:

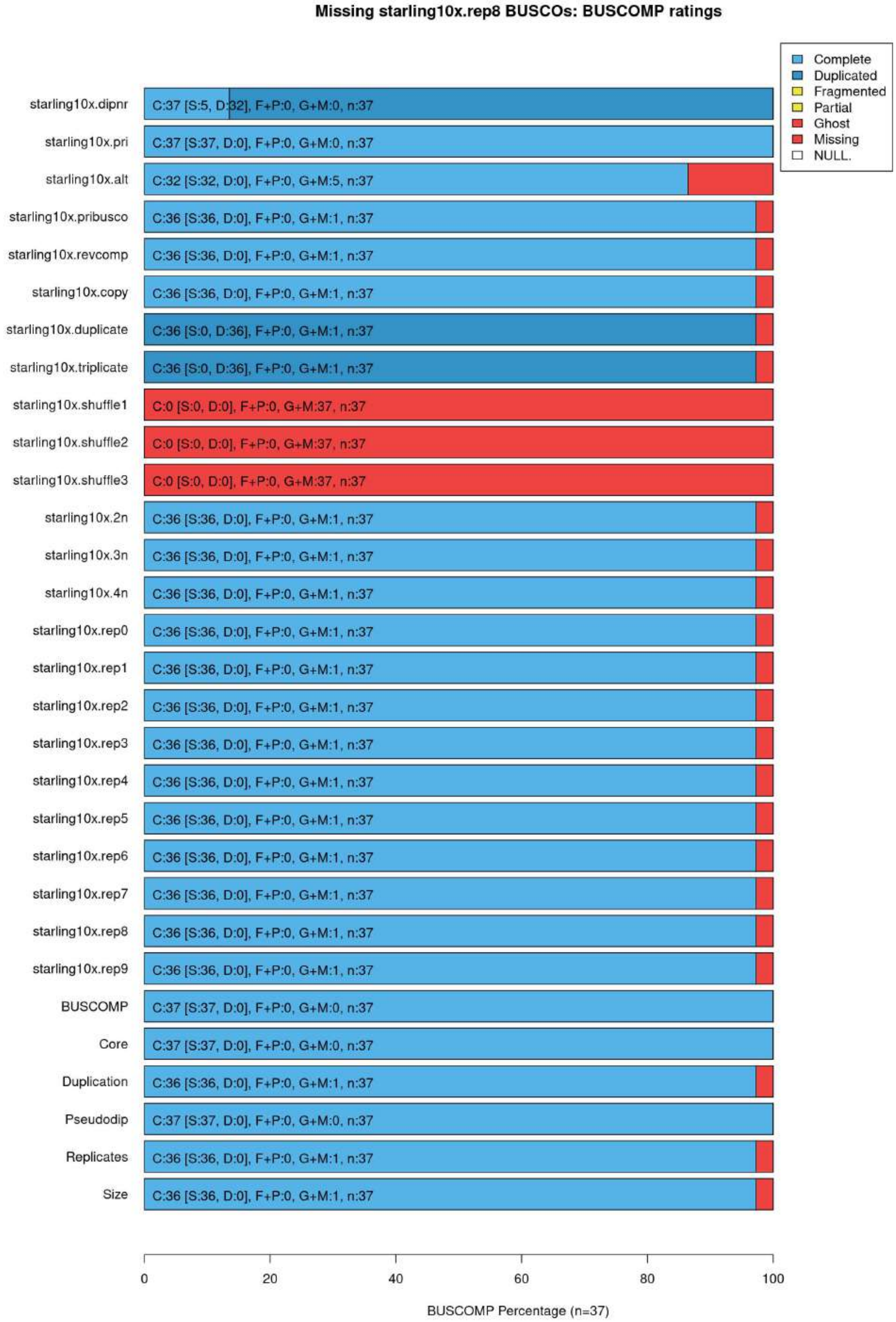

BUSCOMP ratings for `Missing` starling10x.rep8 BUSCOMP genes:

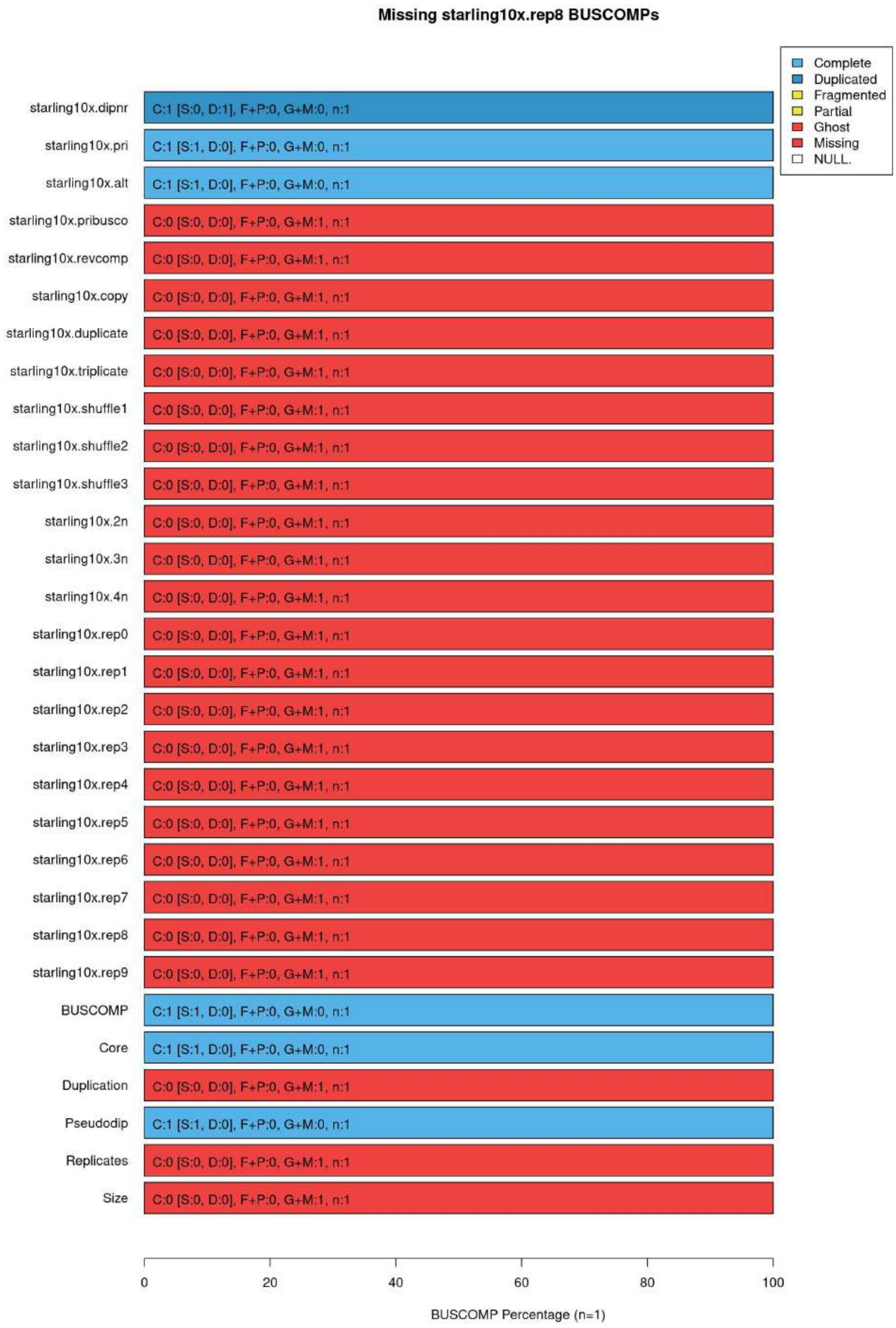

5.31 Missing starling10x.rep9 BUSCO genes

BUSCO ratings for `Missing` starling10x.rep9 BUSCO genes:

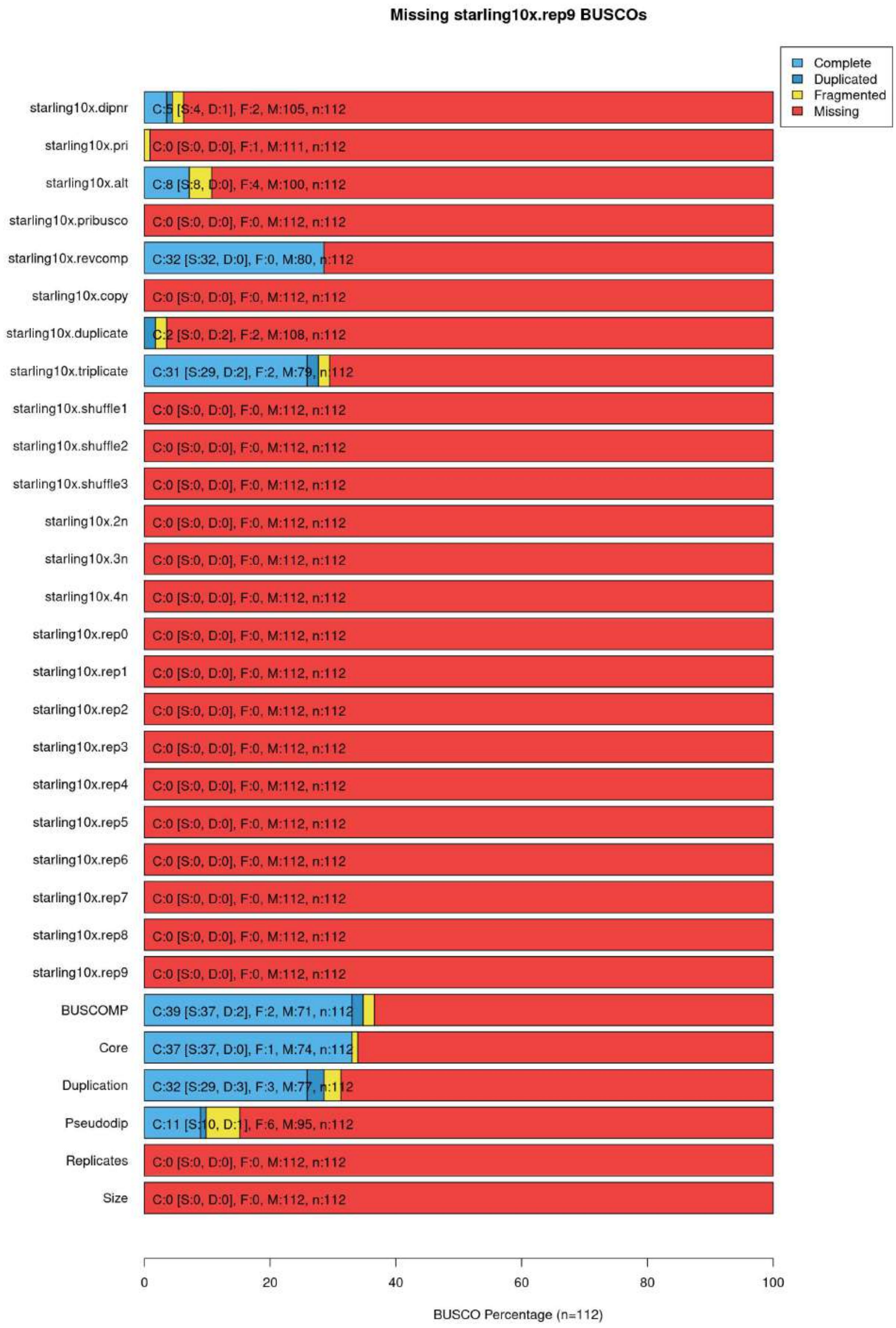

BUSCOMP ratings for `Missing` starling10x.rep9 BUSCO genes:

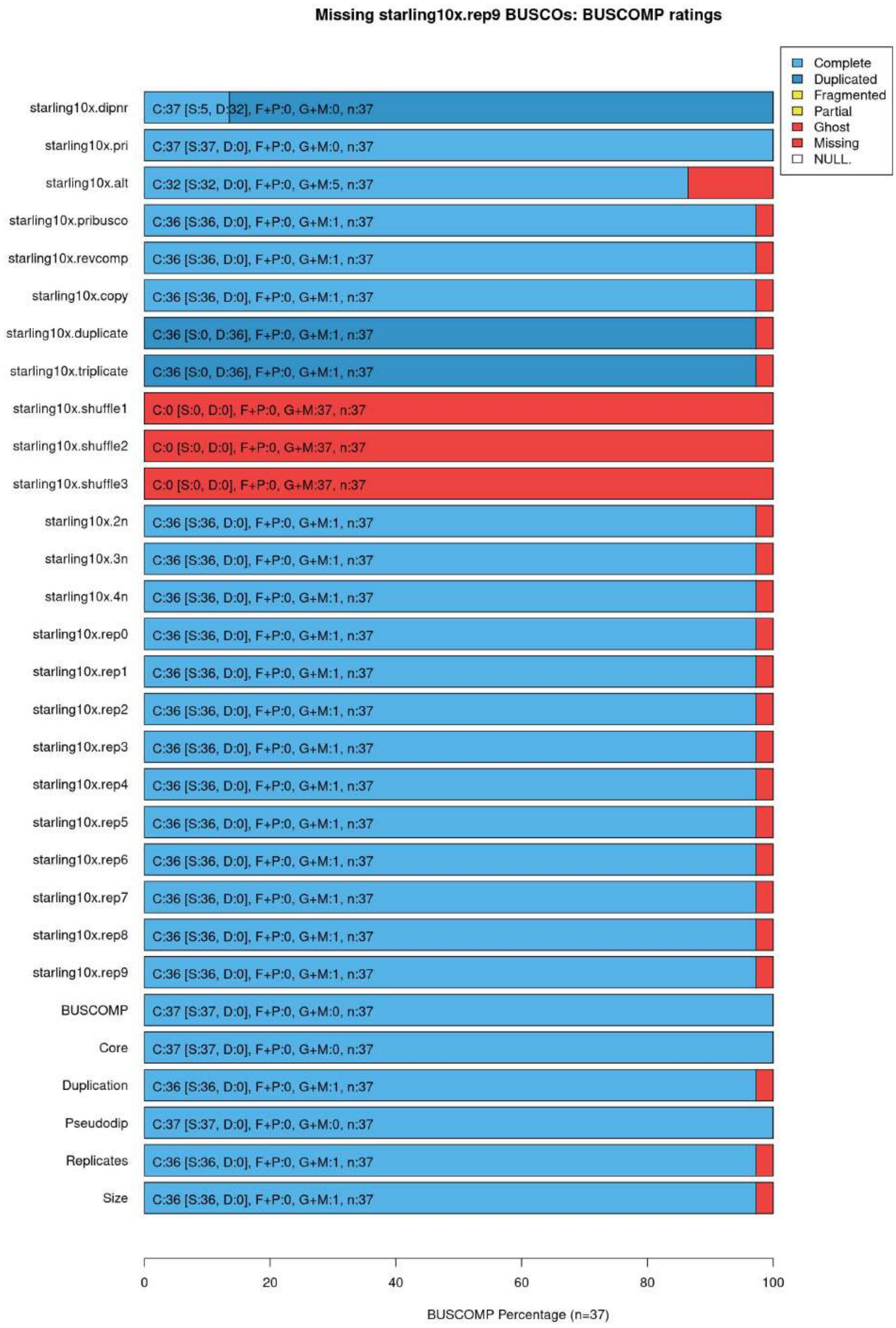

BUSCOMP ratings for `Missing` starling10x.rep9 BUSCOMP genes:

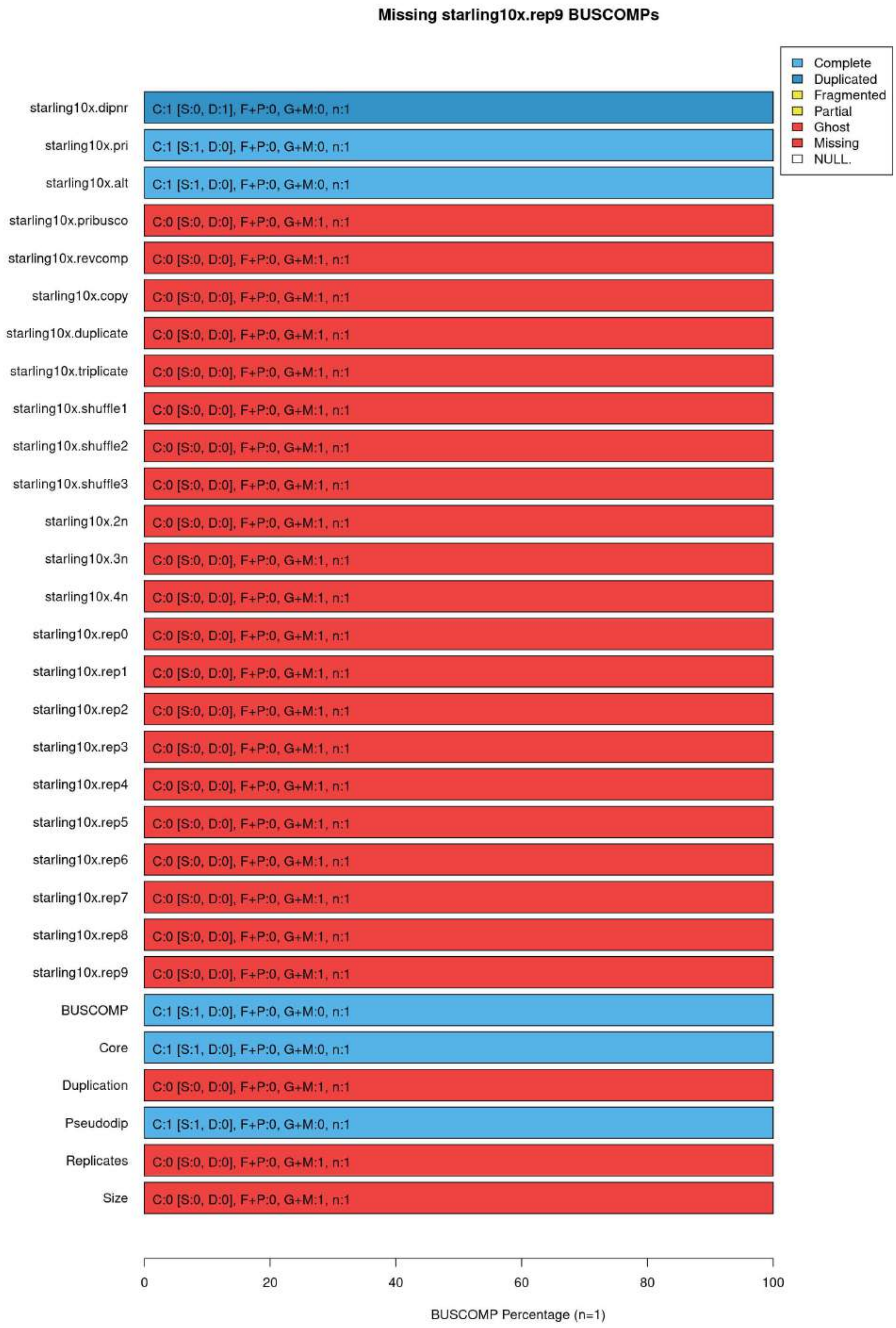

5.32 Missing Replicates BUSCO genes

BUSCO ratings for `Missing` Replicates BUSCO genes:

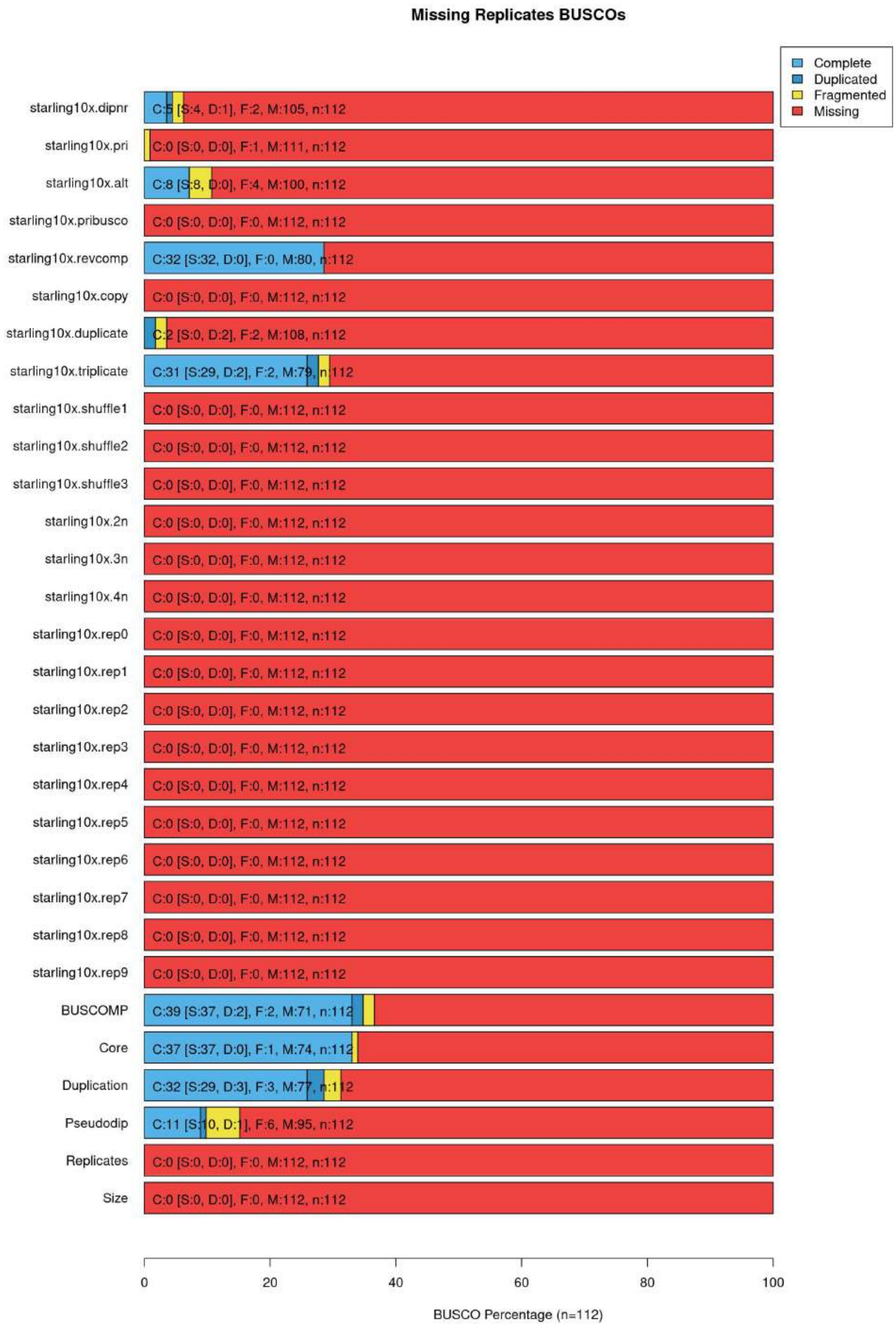

BUSCOMP ratings for `Missing` Replicates BUSCO genes:

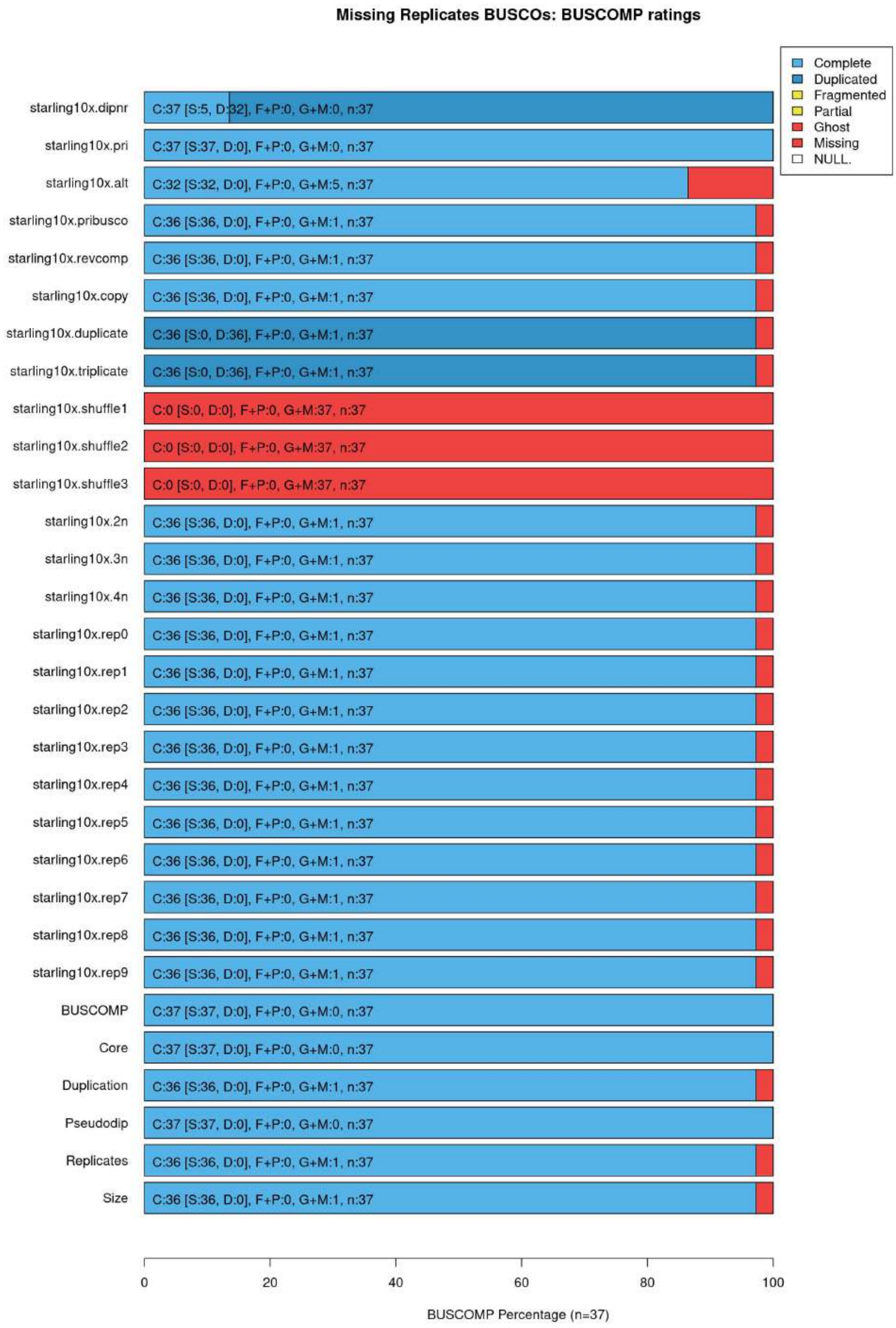

BUSCOMP ratings for `Missing` Replicates BUSCOMP genes:

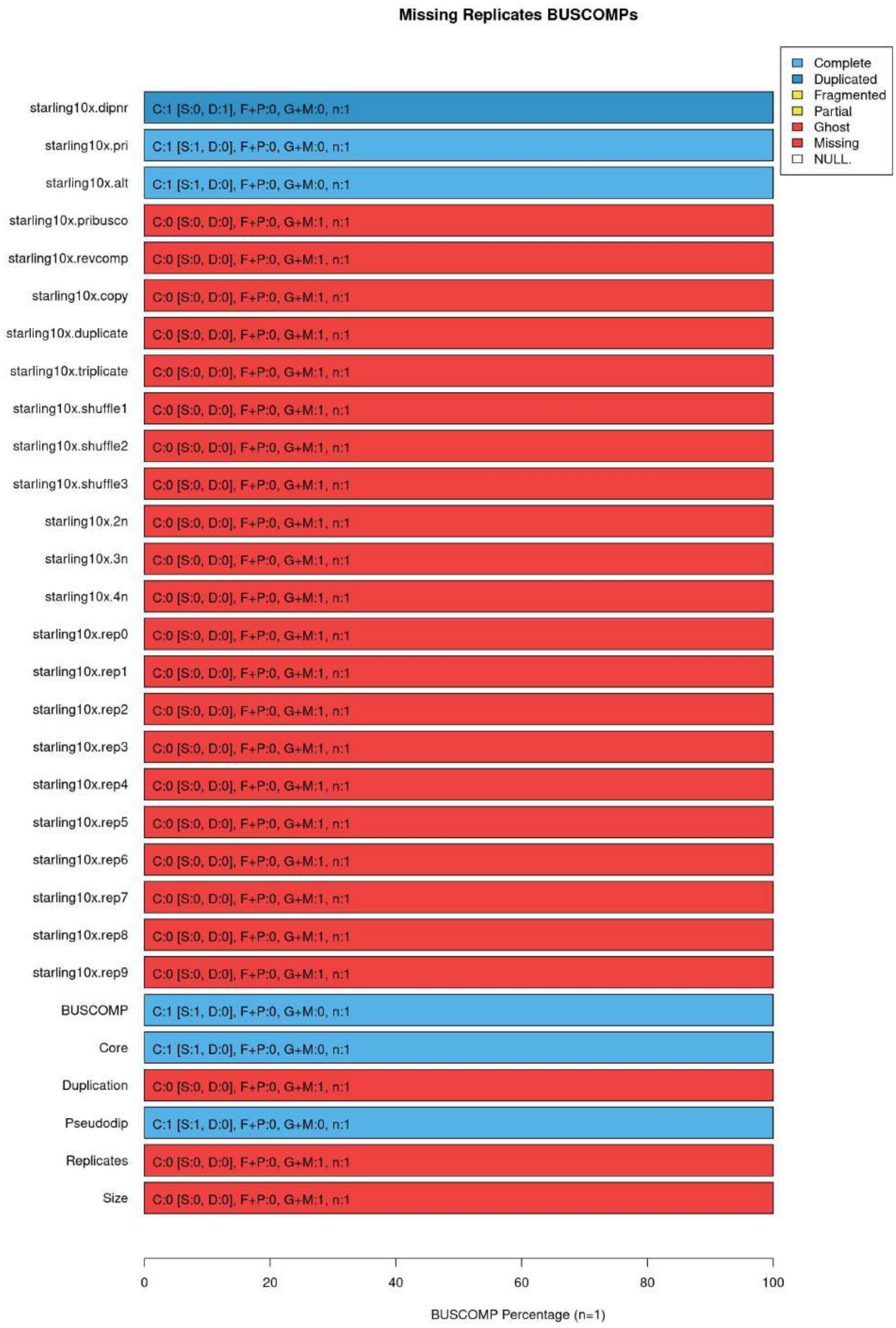

5.33 Missing BUSCOMP BUSCO genes

BUSCO ratings for `Missing` BUSCOMP BUSCO genes:

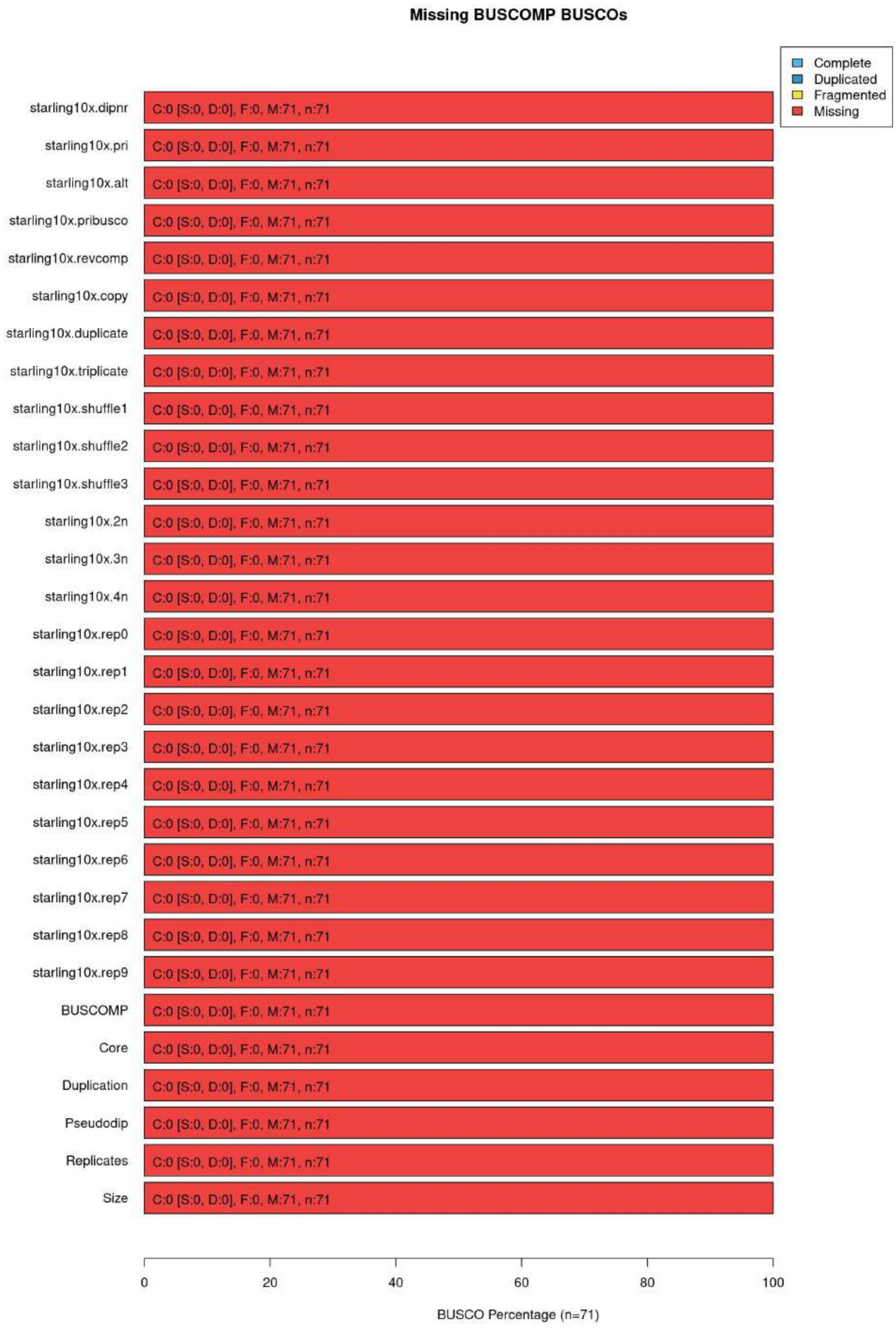

BUSCOMP ratings for `Missing` BUSCOMP BUSCO genes:

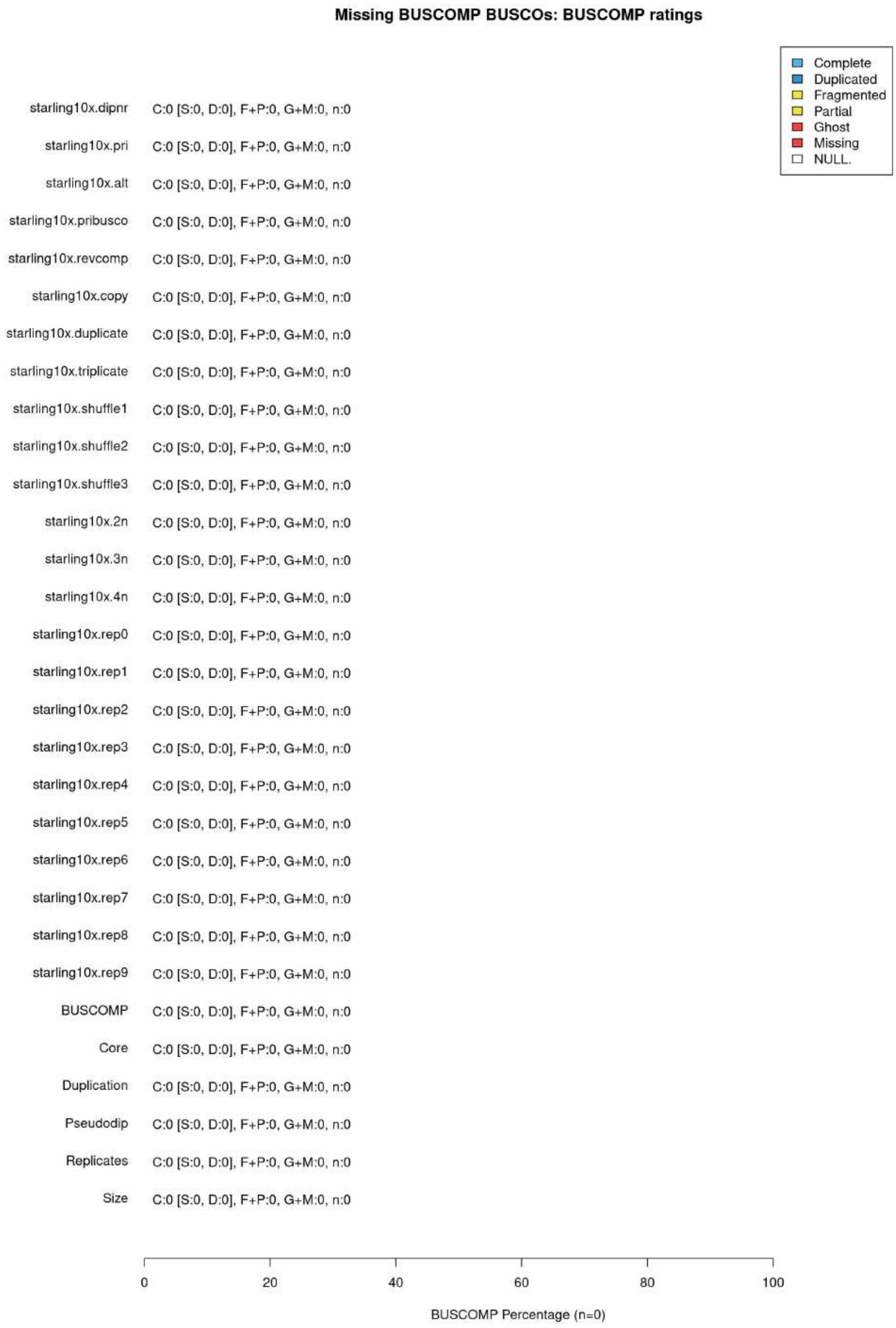

BUSCOMP ratings for `Missing` BUSCOMP BUSCOMP genes:

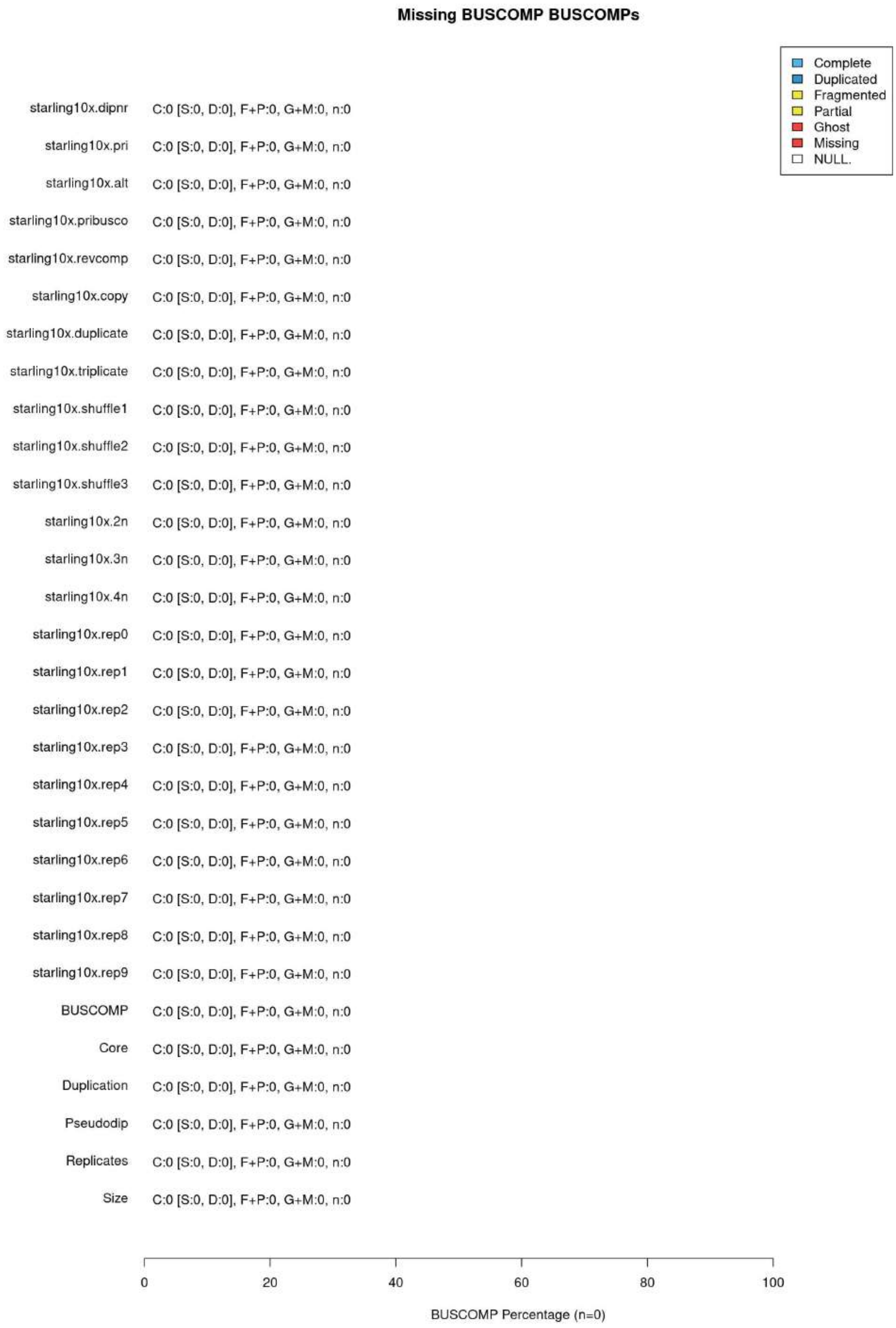

6 Appendix: BUSCOMP run details

```
BUSCOMP V0.11.0: run Thu Mar  4 13:38:02 2021
```

This analysis was run in:

```
/scratch/tmp/z3452659/projects/BUSCOMP-Jan19/analysis/2021-03-03.10xStarling/buscompV3
```

- Log file: `/scratch/tmp/z3452659/projects/BUSCOMP-Jan19/analysis/2021-03-03.10xStarling/buscompV3/starling10xV3.log` **(/scratch/tmp/z3452659/projects/BUSCOMP-Jan19/analysis/2021-03-03.10xStarling/buscompV3/starling10xV3.log)**
- Commandline arguments: `runs=../busco3/run_*` `fastadir=../fasta/` `ratefas=../fasta/*.fasta` `genomesize=1.1e9` `basefile=starling10xV3` `forks=24`
- Full Command List: `mafft=mafft` `clustalo=clustalo` `clustalw=clustalw2` `blast+path=` `iupath=/home/z3452659/bioware/iupred/iupred` `iuchdir=T` `minregion=5` `iucut=0.2` `rpath=R` `modpurge=T` `runs=../busco3/run_*` `fastadir=../fasta/` `ratefas=../fasta/*.fasta` `genomesize=1.1e9` `basefile=starling10xV3` `forks=24`

6.1 BUSCOMP errors

BUSCOMP returned no runtime errors.

6.2 BUSCOMP warnings

See run log for further details:

```
#WARN   00:30:30    34 Length/AlnLen mismatches from PAF file. (May be disparity with N treatment.)
#WARN   00:30:30    19 Identity mismatches from PAF file (PAF identity ignores Ns).
#WARN   00:34:58    16 Length/AlnLen mismatches from PAF file. (May be disparity with N treatment.)
#WARN   00:34:58    9 Identity mismatches from PAF file (PAF identity ignores Ns).
#WARN   00:37:41    18 Length/AlnLen mismatches from PAF file. (May be disparity with N treatment.)
#WARN   00:37:41    10 Identity mismatches from PAF file (PAF identity ignores Ns).
#WARN   00:40:07    16 Length/AlnLen mismatches from PAF file. (May be disparity with N treatment.)
#WARN   00:40:07    9 Identity mismatches from PAF file (PAF identity ignores Ns).
#WARN   00:42:44    14 Length/AlnLen mismatches from PAF file. (May be disparity with N treatment.)
#WARN   00:42:44    9 Identity mismatches from PAF file (PAF identity ignores Ns).
#WARN   00:45:19    16 Length/AlnLen mismatches from PAF file. (May be disparity with N treatment.)
#WARN   00:45:19    9 Identity mismatches from PAF file (PAF identity ignores Ns).
#WARN   00:48:29    32 Length/AlnLen mismatches from PAF file. (May be disparity with N treatment.)
#WARN   00:48:29    18 Identity mismatches from PAF file (PAF identity ignores Ns).
#WARN   00:55:23    46 Length/AlnLen mismatches from PAF file. (May be disparity with N treatment.)
#WARN   00:55:23    27 Identity mismatches from PAF file (PAF identity ignores Ns).
#WARN   01:04:54    16 Length/AlnLen mismatches from PAF file. (May be disparity with N treatment.)
#WARN   01:04:54    9 Identity mismatches from PAF file (PAF identity ignores Ns).
#WARN   01:08:23    16 Length/AlnLen mismatches from PAF file. (May be disparity with N treatment.)
#WARN   01:08:23    9 Identity mismatches from PAF file (PAF identity ignores Ns).
#WARN   01:12:22    16 Length/AlnLen mismatches from PAF file. (May be disparity with N treatment.)
#WARN   01:12:22    9 Identity mismatches from PAF file (PAF identity ignores Ns).
#WARN   01:14:43    16 Length/AlnLen mismatches from PAF file. (May be disparity with N treatment.)
#WARN   01:14:43    9 Identity mismatches from PAF file (PAF identity ignores Ns).
#WARN   01:17:10    16 Length/AlnLen mismatches from PAF file. (May be disparity with N treatment.)
#WARN   01:17:10    9 Identity mismatches from PAF file (PAF identity ignores Ns).
#WARN   01:19:38    16 Length/AlnLen mismatches from PAF file. (May be disparity with N treatment.)
#WARN   01:19:38    9 Identity mismatches from PAF file (PAF identity ignores Ns).
#WARN   01:22:05    16 Length/AlnLen mismatches from PAF file. (May be disparity with N treatment.)
#WARN   01:22:05    9 Identity mismatches from PAF file (PAF identity ignores Ns).
#WARN   01:24:32    16 Length/AlnLen mismatches from PAF file. (May be disparity with N treatment.)
#WARN   01:24:32    9 Identity mismatches from PAF file (PAF identity ignores Ns).
#WARN   01:27:01    16 Length/AlnLen mismatches from PAF file. (May be disparity with N treatment.)
#WARN   01:27:01    9 Identity mismatches from PAF file (PAF identity ignores Ns).
#WARN   01:29:29    16 Length/AlnLen mismatches from PAF file. (May be disparity with N treatment.)
#WARN   01:29:29    9 Identity mismatches from PAF file (PAF identity ignores Ns).
#WARN   01:31:57    16 Length/AlnLen mismatches from PAF file. (May be disparity with N treatment.)
#WARN   01:31:57    9 Identity mismatches from PAF file (PAF identity ignores Ns).
#WARN   01:34:24    16 Length/AlnLen mismatches from PAF file. (May be disparity with N treatment.)
#WARN   01:34:24    9 Identity mismatches from PAF file (PAF identity ignores Ns).
#WARN   01:36:51    16 Length/AlnLen mismatches from PAF file. (May be disparity with N treatment.)
#WARN   01:36:51    9 Identity mismatches from PAF file (PAF identity ignores Ns).
```

Report contents:

- **Run summary**
- **BUSCOMP summary**
- **Genome summary**
- **BUSCO Ratings**
- **BUSCO full results compilation**
- **BUSCOMP Sequence details and rating**
- **BUSCOMP re-rating of genomes**
- **BUSCOMP re-rating full results**
- **Unique BUSCO and BUSCOMP Complete genes**
- **Ratings for Missing BUSCO genes**
- **Appendix: BUSCOMP run details**

Output generated by BUSCOMP v0.11.0 © 2019 Richard Edwards | **richard.edwards@unsw.edu.au (mailto:richard.edwards@unsw.edu.au)**
