# Supplementary material for: Transcript‐ and annotation‐guided genome assembly of the European starling
Source: Mol Ecol Resour. 2022 Jul 18;22(8):3141–60. doi: 10.1111/1755-0998.13679 (PMC9796300; doi:10.1111/1755-0998.13679)
Supplement: Supplementary file 1 — Appendix S1 [file MEN-22-3141-s001.zip › MEN_13679_Sv3.10_Supplementary_File_2_starling10xV3.N3L20ID0U.full_compressed.pdf]

1 BUSCOMP Run Summary
2 Genome Summary
3 BUSCO Ratings
4 BUSCOMP Ratings
5 BUSCO and BUSCOMP Comparisons
6 Appendix: BUSCOMP run details

BUSCOMP Full Report

starling10xV5 BUSCOMP Analysis

2021-03-04

1 BUSCOMP Run Summary

```
BUSCOMP V0.11.0: run Thu Mar  4 16:10:40 2021
```

See the **run details appendix** end of this document for details of the **log file (/scratch/tmp/z3452659/projects/BUSCOMP-Jan19/analysis/2021-03-03.10xStarling/buscompV5/starling10xV5.log)**, **commandline parameters** and runtime **BUSCOMP errors and warnings**.

NOTE: To edit this document, open `starling10xV5.N3L20ID0U.full.Rmd` in RStudio, edit and re-knit to HTML.

1.1 BUSCOMP Results Summary

Assemblies can be assessed on a number of criteria, but the main ones (in the absence of a reference "truth" genome) are either to judge contiguity or completeness. NG50 and LG50 values are based on a genome size of 1.1 Gb. If the `genomesize=X` parameter was not set (see command list in **appendix**), this will be based on the longest assembly (see sequence stats, below).

Of the 24 assemblies analysed (24 BUSCO; 24 fasta; 24 both), 23 genomes were rated as the "best" by at least one criterion:

- `starling10x.shuffle2` : MaxLength.
- `starling10x.pribusco` : MaxLength.
- `starling10x.dipnr` : MaxLength, Complete, Missing.
- `starling10x.pri` : MaxLength, Complete, Missing, BUSCO, NoBUSCO.
- `starling10x.shuffle3` : MaxLength.
- `starling10x.triplicate` : MaxLength.
- `starling10x.3n` : MaxLength.
- `starling10x.duplicate` : MaxLength.
- `starling10x.4n` : NG50Length, LG50Count, MaxLength.
- `starling10x.rep6` : MaxLength.
- `starling10x.rep7` : MaxLength.
- `starling10x.rep4` : MaxLength.
- `starling10x.rep5` : MaxLength.
- `starling10x.rep2` : MaxLength.
- `starling10x.rep3` : MaxLength.
- `starling10x.rep0` : MaxLength.
- `starling10x.rep1` : MaxLength.
- `starling10x.rep8` : MaxLength.
- `starling10x.rep9` : MaxLength.
- `starling10x.shuffle1` : MaxLength.
- `starling10x.revcomp` : MaxLength.
- `starling10x.2n` : MaxLength.
- `starling10x.copy` : MaxLength.

Best assemblies by assembly contiguity critera:

- **NG50Length.** Longest NG50 contig/scaffold length (6,889,770 bp): `starling10x.4n`
- **LG50Count.** Smallest LG50 contig/scaffold count (67): `starling10x.4n`
- **MaxLength.** Maximum contig/scaffold length (12,884,419 bp): `starling10x.2n` , `starling10x.3n` , `starling10x.4n` , `starling10x.copy` , `starling10x.dipnr` , `starling10x.duplicate` , `starling10x.pri` , `starling10x.pribusco` , `starling10x.rep0` , `starling10x.rep1` , `starling10x.rep2` , `starling10x.rep3` , `starling10x.rep4` , `starling10x.rep5` , `starling10x.rep6` , `starling10x.rep7` , `starling10x.rep8` , `starling10x.rep9` , `starling10x.revcomp` , `starling10x.shuffle1` , `starling10x.shuffle2` , `starling10x.shuffle3` , `starling10x.triplicate`

Best assemblies by completeness critera:

- **Complete.** Most Complete (Single & Duplicated) BUSCOMP sequences (99.9 %): `starling10x.dipnr` , `starling10x.pri`
- **Missing.** Fewest Missing BUSCOMP sequences (0.0 %): `starling10x.dipnr` , `starling10x.pri`
- **BUSCO.** Most Complete (Single & Duplicated) BUSCO sequences (94.8 %): `starling10x.pri`
- **NoBUSCO.** Fewest Missing BUSCO sequences (3.3 %): `starling10x.pri`

2 Genome Summary

The following genomes and BUSCO results were analysed by BUSCOMP:

- **starling10x.dipnr**. [`BUSCO` | `Fasta`] Pseudodiploid assembly
- **starling10x.pri**. [`BUSCO` | `Fasta`] Primary scaffolds

- **starling10x.alt**. [`BUSCO` | `Fasta`] Alternative scaffolds
- **starling10x.pribusco**. [`BUSCO` | `Fasta`] Primary BUSCO-containing scaffolds
- **starling10x.revcomp**. [`BUSCO` | `Fasta`] Reverse complemented BUSCO scaffolds
- **starling10x.copy**. [`BUSCO` | `Fasta`] Direct copy of primary BUSCO scaffolds
- **starling10x.duplicate**. [`BUSCO` | `Fasta`] Primary BUSCOs plus reverse complement
- **starling10x.triplicate**. [`BUSCO` | `Fasta`] Two primary BUSCO copies plus reverse complement
- **starling10x.shuffle1**. [`Fasta`] Randomly shuffled primary BUSCO scaffolds (1)
- **starling10x.shuffle2**. [`Fasta`] Randomly shuffled primary BUSCO scaffolds (2)
- **starling10x.shuffle3**. [`Fasta`] Randomly shuffled primary BUSCO scaffolds (3)
- **starling10x.2n**. [`BUSCO` | `Fasta`] Primary BUSCO scaffolds plus one shuffled copy
- **starling10x.3n**. [`BUSCO` | `Fasta`] Primary BUSCO scaffolds plus two shuffled copies
- **starling10x.4n**. [`BUSCO` | `Fasta`] Primary BUSCO scaffolds plus three shuffled copies
- **starling10x.rep0**. [`BUSCO` | `Fasta`] Primary BUSCO replication 0
- **starling10x.rep1**. [`BUSCO` | `Fasta`] Primary BUSCO replication 1
- **starling10x.rep2**. [`BUSCO` | `Fasta`] Primary BUSCO replication 2
- **starling10x.rep3**. [`BUSCO` | `Fasta`] Primary BUSCO replication 3
- **starling10x.rep4**. [`BUSCO` | `Fasta`] Primary BUSCO replication 4
- **starling10x.rep5**. [`BUSCO` | `Fasta`] Primary BUSCO replication 5
- **starling10x.rep6**. [`BUSCO` | `Fasta`] Primary BUSCO replication 6
- **starling10x.rep7**. [`BUSCO` | `Fasta`] Primary BUSCO replication 7
- **starling10x.rep8**. [`BUSCO` | `Fasta`] Primary BUSCO replication 8
- **starling10x.rep9**. [`BUSCO` | `Fasta`] Primary BUSCO replication 9

Details of the directories and files are below:

	**Directory** <chr>
1	../busco5/run_00_starling10x.dipnr
2	../busco5/run_01_starling10x.pri
3	../busco5/run_02_starling10x.alt
5	../busco5/run_04_starling10x.pribusco
6	../busco5/run_05_starling10x.revcomp
8	../busco5/run_11_starling10x.copy
9	../busco5/run_12_starling10x.duplicate
10	../busco5/run_13_starling10x.triplicate
12	
13	

1-10 of 24 rows | 1-2 of 6 columns **Previous 1 2 3 Next**

Genomes with a **Directory** listed had BUSCO results available. If **Sequences** is `True`, these would be have been compiled to generate the BUSCOMP sequence set (unless `buscompseq=F`, or alternative sequences were provided with `buscofas=FASFILE`). Genomes with a **Fasta** listed had sequence data available for BUSCOMP searches.

2.1 Genome statistics

The following genome statistics were also calculated by `RJE_SeqList` for each genome (table, below):

- **SeqNum**: The total number of scaffolds/contigs in the assembly.
- **TotLength**: The total combined length of scaffolds/contigs in the assembly.
- **MinLength**: The length of the shortest scaffold/contig in the assembly.
- **MaxLength**: The length of the longest scaffold/contig in the assembly.
- **MeanLength**: The mean length of scaffolds/contigs in the assembly.
- **MedLength**: The median length of scaffolds/contigs in the assembly.
- **N50Length**: At least half of the assembly is contained on scaffolds/contigs of this length or greater.
- **L50Count**: The smallest number scaffolds/contigs needed to cover half the the assembly.
- **CtgNum**: Number of contigs (`SeqNum` + `GapCount`).
- **N50Ctg**: At least half of the assembly is contained on contigs of this length or greater.
- **L50Ctg**: The smallest number contigs needed to cover half the the assembly.
- **NG50Length**: At least half of the genome is contained on scaffolds/contigs of this length or greater. This is based on `genomesize=X`. If no genome size is given, it will be relative to the biggest assembly.
- **LG50Count**: The smallest number scaffolds/contigs needed to cover half the the genome. This is based on `genomesize=X`. If no genome size is given, it will be relative to the biggest assembly.
- **GapLength**: The total number of undefined "gap" (`N`) nucleotides in the assembly.
- **GapCount**: The total number of undefined "gap" (`N`) regions in the assembly.
- **GC**: The %GC content of the assembly.

Genome	Description	SeqNum	TotLength	MinLength	MaxLength	MeanLength	MedLength	N50Length	L50Count	CtgNum	N50Ctg
starling10x.dipnr	Pseudodiploid assembly	19346	1887443050	1000	12884419	97562.44	2307.5	1907593	245	49415	142122
starling10x.pri	Primary scaffolds	18439	1040106492	1000	12884419	56407.97	2153.0	1764435	146	37354	132973

Genome	Description	SeqNum	TotLength	MinLength	MaxLength	MeanLength	MedLength	N50Length	L50Count	CtgNum	N50Ctg
starling10x.alt	Alternative scaffolds	907	847336558	1620	12880535	934218.92	450476.0	2203301	102	12061	152010
starling10x.pribusco	Primary BUSCO-containing scaffolds	968	876348215	1833	12884419	905318.40	409882.5	2277667	104	11579	154535
starling10x.revcomp	Reverse complemented BUSCO scaffolds	968	876348215	1833	12884419	905318.40	409882.5	2277667	104	11579	154535
starling10x.copy	Direct copy of primary BUSCO scaffolds	968	876348215	1833	12884419	905318.40	409882.5	2277667	104	11579	154535
starling10x.duplicate	Primary BUSCOs plus reverse complement	1936	1752696430	1833	12884419	905318.40	409882.5	2277667	208	23158	154535
starling10x.triplicate	Two primary BUSCO copies plus reverse complement	2904	2629044645	1833	12884419	905318.40	409882.5	2277667	312	34737	154535
starling10x.shuffle1	Randomly shuffled primary BUSCO scaffolds (1)	968	876348215	1833	12884419	905318.40	409882.5	2277667	104	971	2277667
starling10x.shuffle2	Randomly shuffled primary BUSCO scaffolds (2)	968	876348215	1833	12884419	905318.40	409882.5	2277667	104	973	2277667
starling10x.shuffle3	Randomly shuffled primary BUSCO scaffolds (3)	968	876348215	1833	12884419	905318.40	409882.5	2277667	104	969	2277667
starling10x.2n	Primary BUSCO scaffolds plus one shuffled copy	1936	1752696430	1833	12884419	905318.40	409882.5	2277667	208	12550	436368
starling10x.3n	Primary BUSCO scaffolds plus two shuffled copies	2904	2629044645	1833	12884419	905318.40	409882.5	2277667	312	13523	1002636
starling10x.4n	Primary BUSCO scaffolds plus three shuffled copies	3872	3505392860	1833	12884419	905318.40	409882.5	2277667	416	14492	1397823
starling10x.rep0	Primary BUSCO replication 0	968	876348215	1833	12884419	905318.40	409882.5	2277667	104	11579	154535
starling10x.rep1	Primary BUSCO replication 1	968	876348215	1833	12884419	905318.40	409882.5	2277667	104	11579	154535
starling10x.rep2	Primary BUSCO replication 2	968	876348215	1833	12884419	905318.40	409882.5	2277667	104	11579	154535
starling10x.rep3	Primary BUSCO replication 3	968	876348215	1833	12884419	905318.40	409882.5	2277667	104	11579	154535

Genome	Description	SeqNum	TotLength	MinLength	MaxLength	MeanLength	MedLength	N50Length	L50Count	CtgNum	N50Ctg
starling10x.rep4	Primary BUSCO replication 4	968	876348215	1833	12884419	905318.40	409882.5	2277667	104	11579	154535
starling10x.rep5	Primary BUSCO replication 5	968	876348215	1833	12884419	905318.40	409882.5	2277667	104	11579	154535
starling10x.rep6	Primary BUSCO replication 6	968	876348215	1833	12884419	905318.40	409882.5	2277667	104	11579	154535
starling10x.rep7	Primary BUSCO replication 7	968	876348215	1833	12884419	905318.40	409882.5	2277667	104	11579	154535
starling10x.rep8	Primary BUSCO replication 8	968	876348215	1833	12884419	905318.40	409882.5	2277667	104	11579	154535
starling10x.rep9	Primary BUSCO replication 9	968	876348215	1833	12884419	905318.40	409882.5	2277667	104	11579	154535

NOTE: `NG50Length` and `LG50Count` statistics use `genomesize=X` or the biggest assembly loaded (1.10 Gb). If BUSCOMP has been run more than once on the same data (*e.g.* to update descriptions or sorting), please make sure that a consistent genome size is used, or these values may be wrong. If in doubt, run with `force=T` and force regeneration of statistics.

2.2 Genome coverage assessment plots

In general, a good assembly will be approx. the same size as the genome and in as few pieces as possible. Any assembly smaller than the predicted genome size is clearly missing coverage. Assemblies bigger than the genome size might still be missing chunks of the genome if redundancy/duplication is a problem. In the following plot, the grey line marks the given genome size of 1.1 Gb.

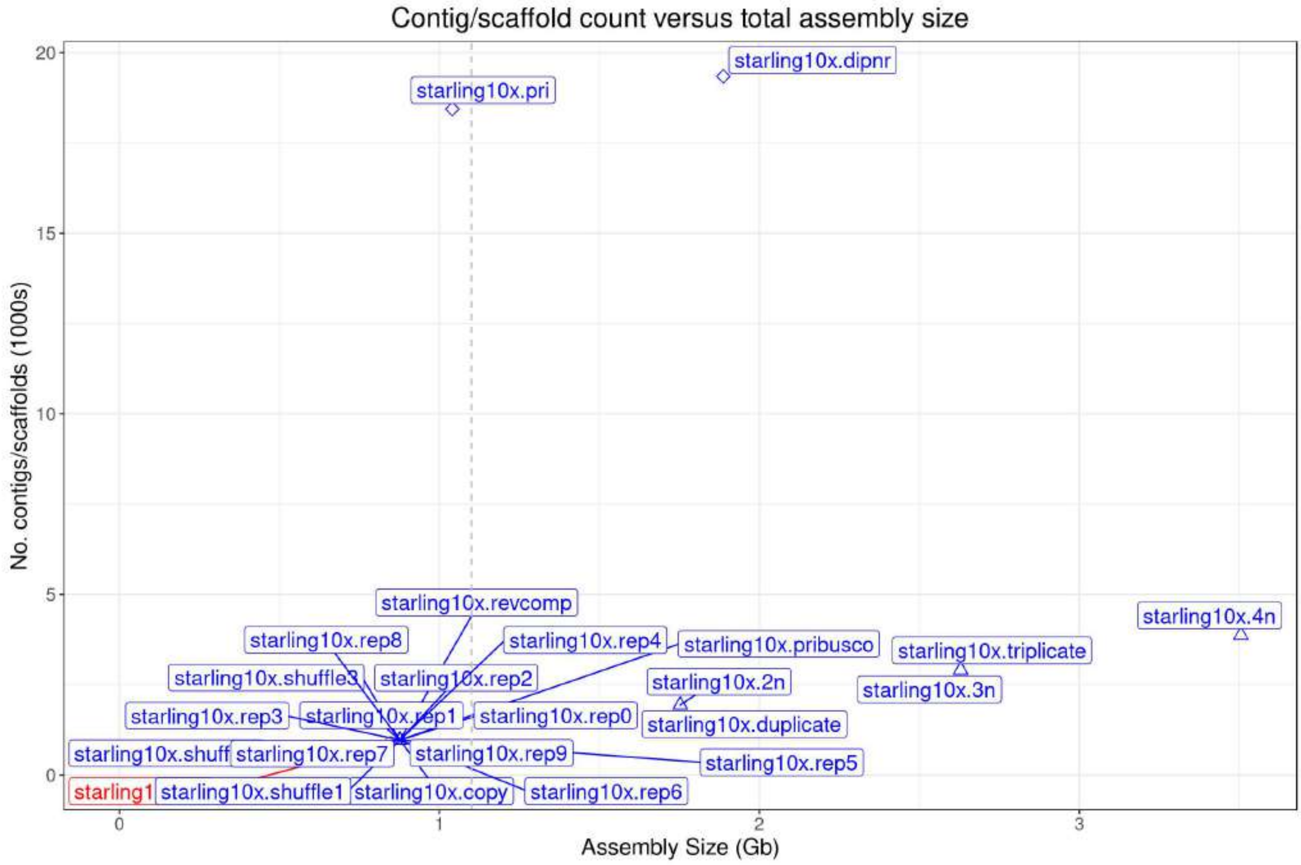

A better indicator of the overall coverage of the genome is the number of `Missing` BUSCO genes. As BUSCO is highly dependent on the accuracy of the sequence and the gene models it makes, the `Missing` BUSCOMP ratings arguably give a more consistent proxy for genome completeness. NOTE: this says nothing about the fragmentation or completeness of the genes themselves.

Missing BUSCO versus total assembly size

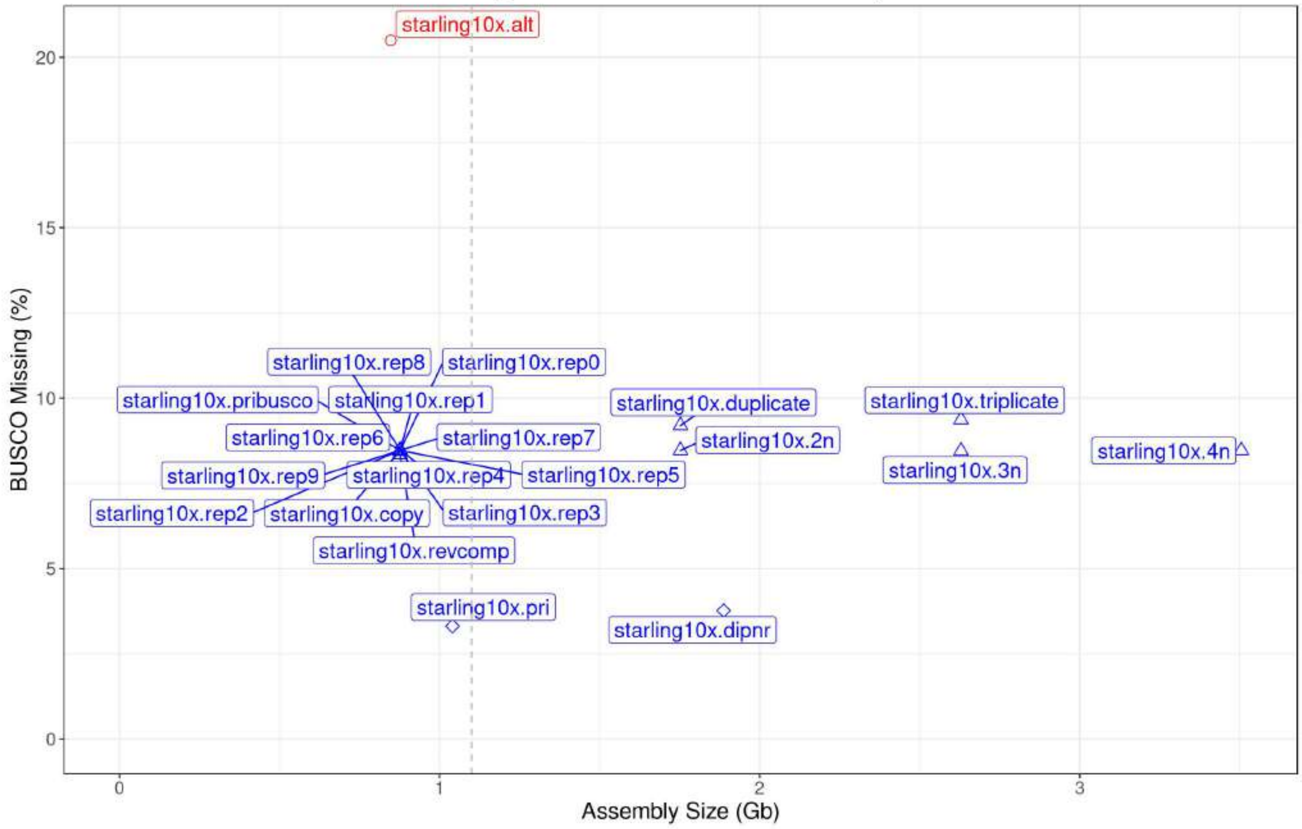

Missing BUSCOMP versus total assembly size

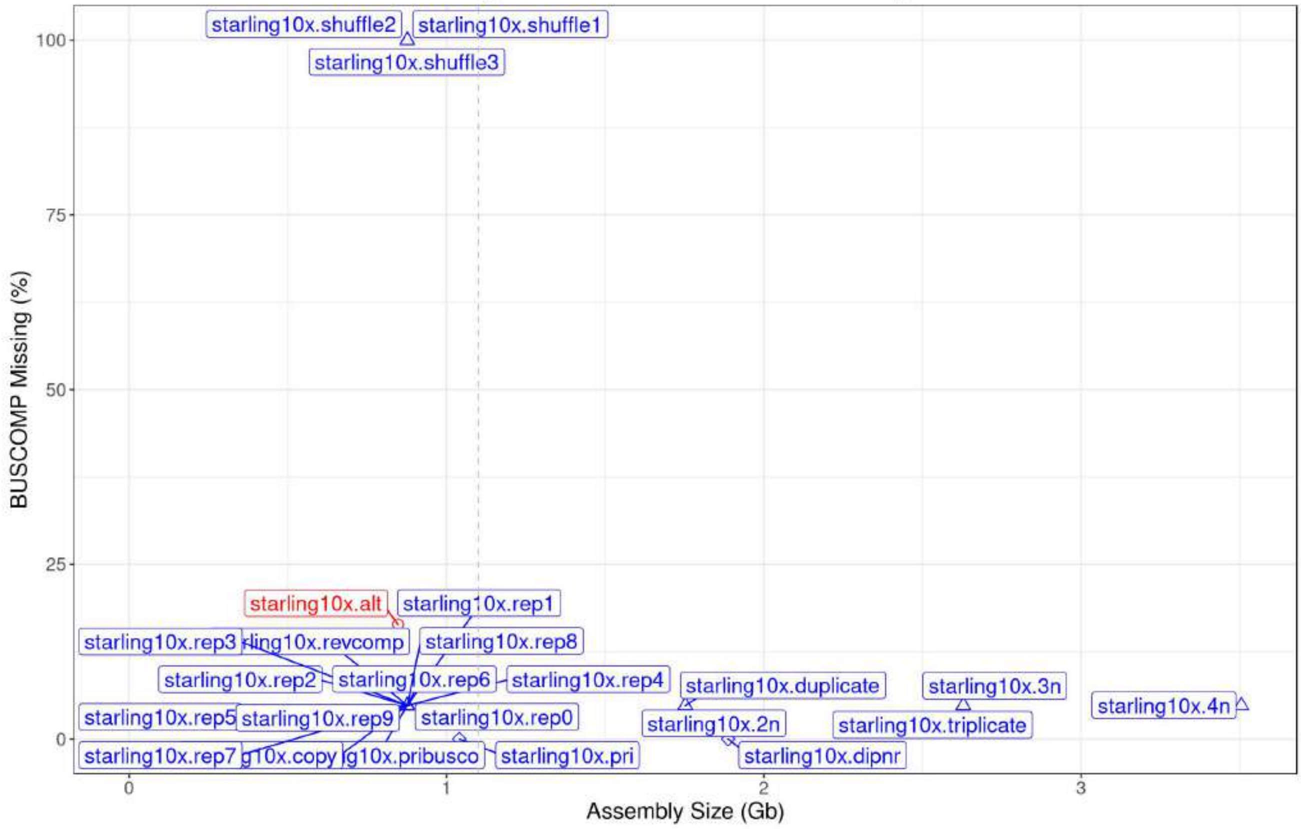

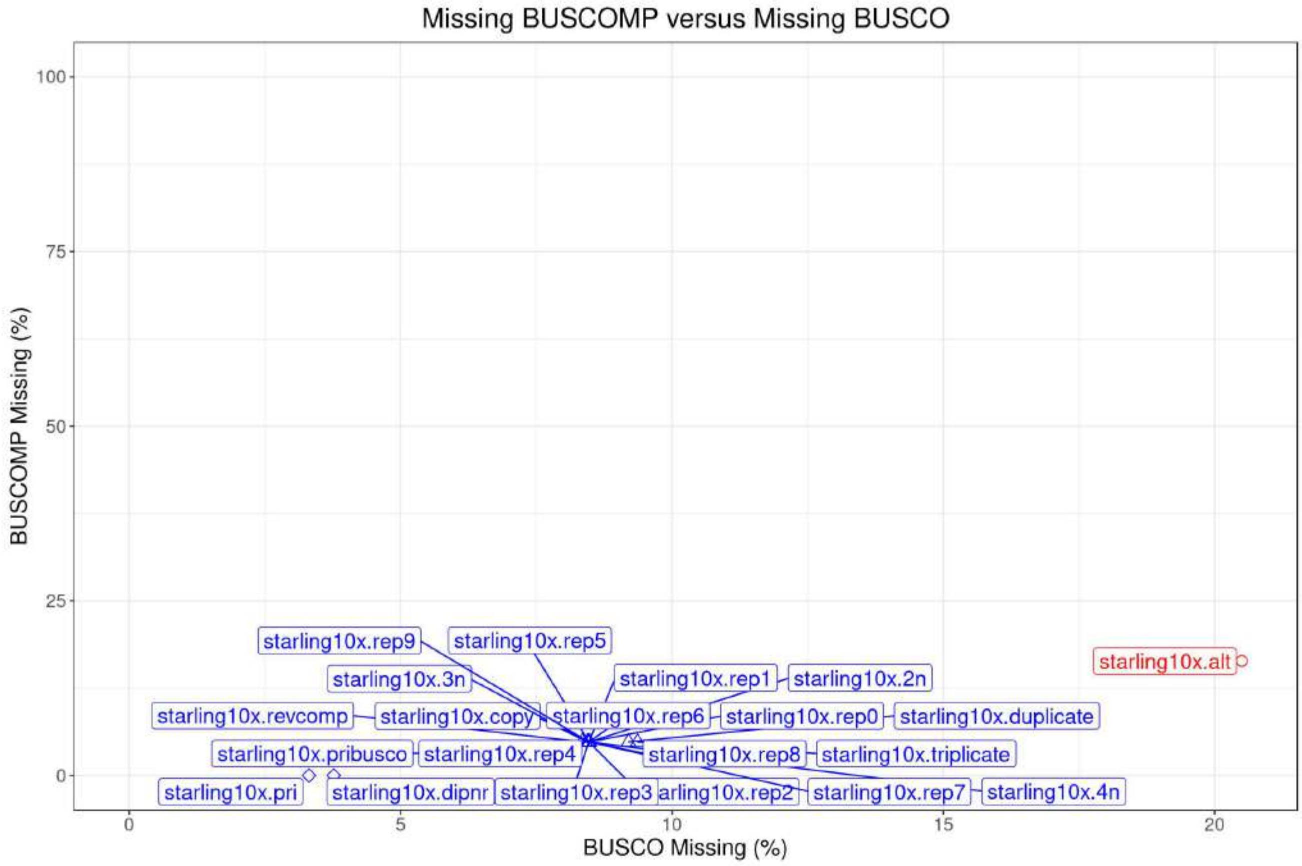

2.3 Genome contiguity assessment plots

In general, a good assembly will be in fewer, bigger pieces. This is approximated using NG50 and LG50, which are the min. length and number of contigs/scaffolds required to cover at least half the genome. These stats use the given genome size of 1.1 Gb.

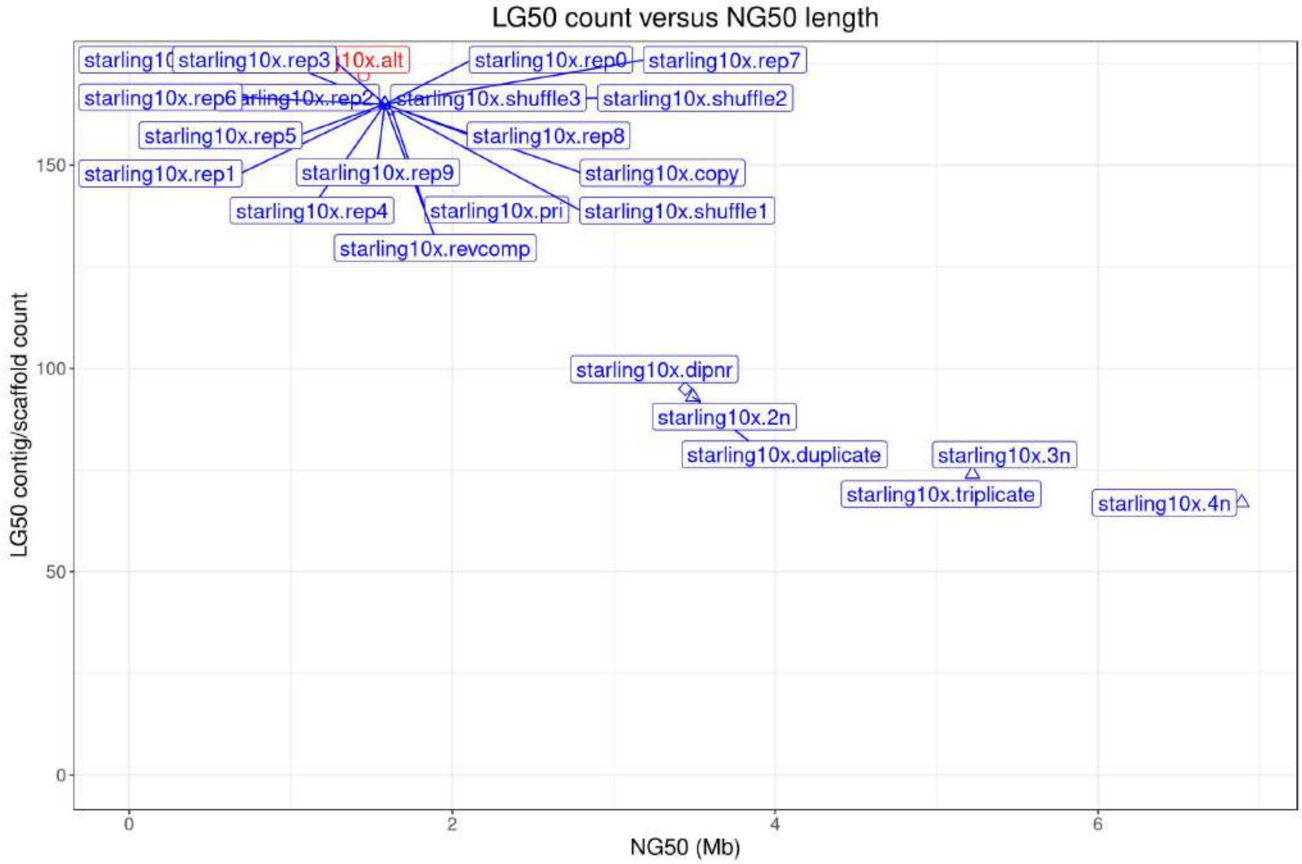

Complete BUSCOMP versus NG50

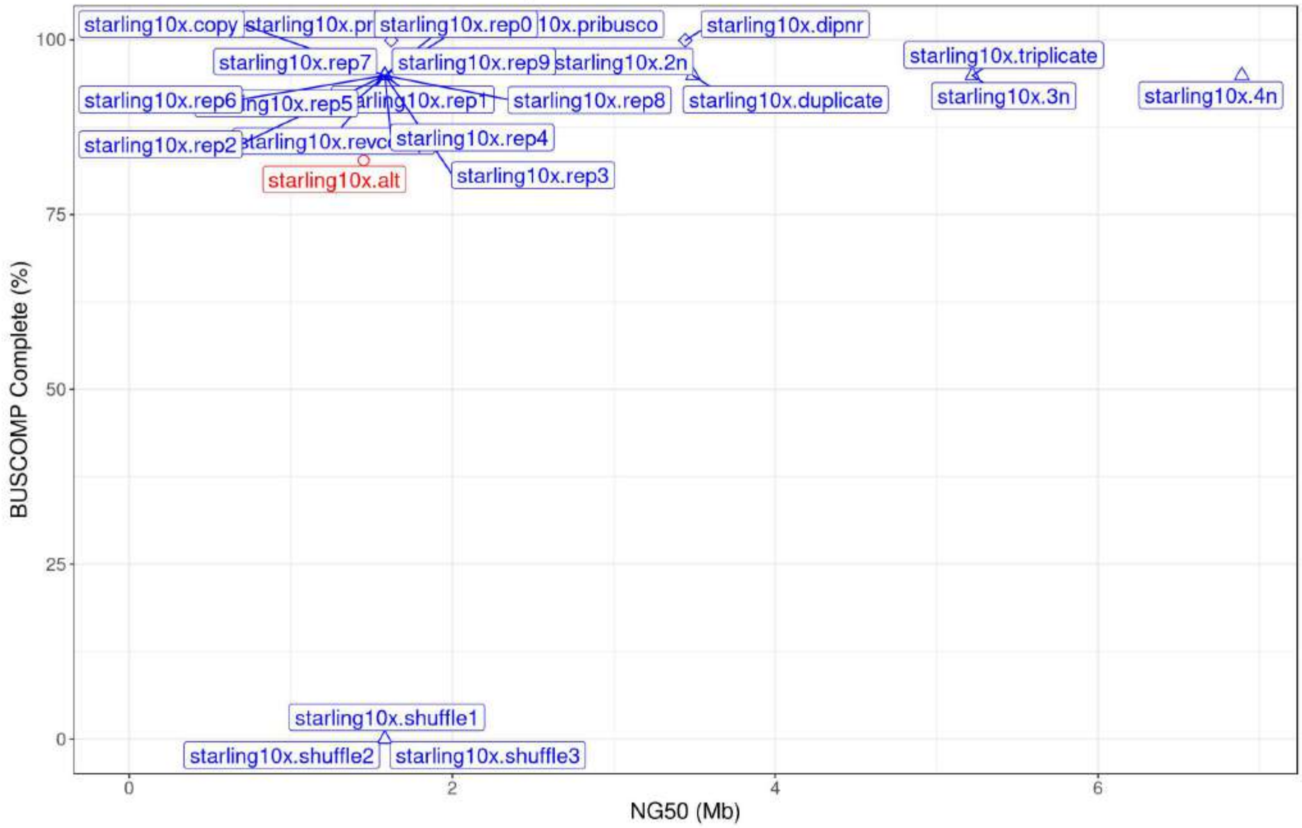

Complete BUSCO versus NG50

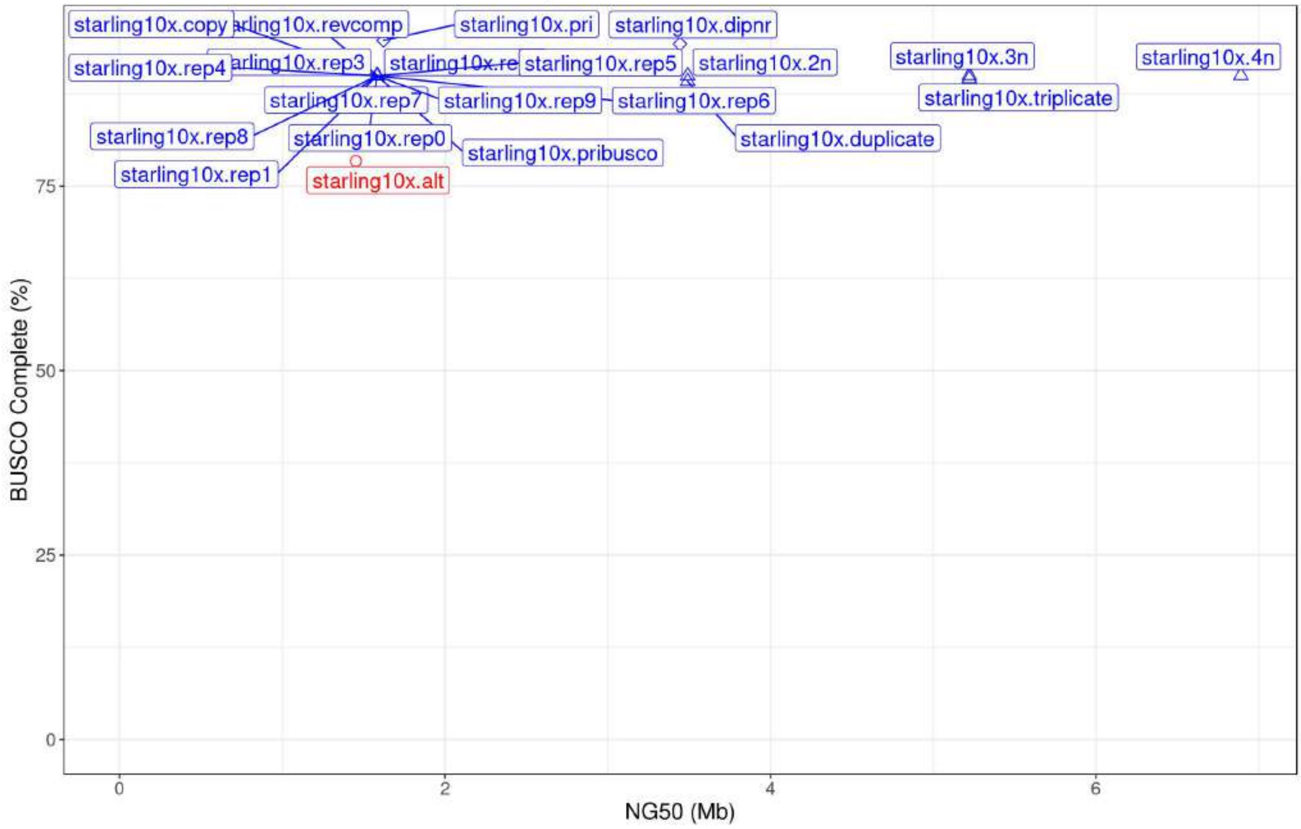

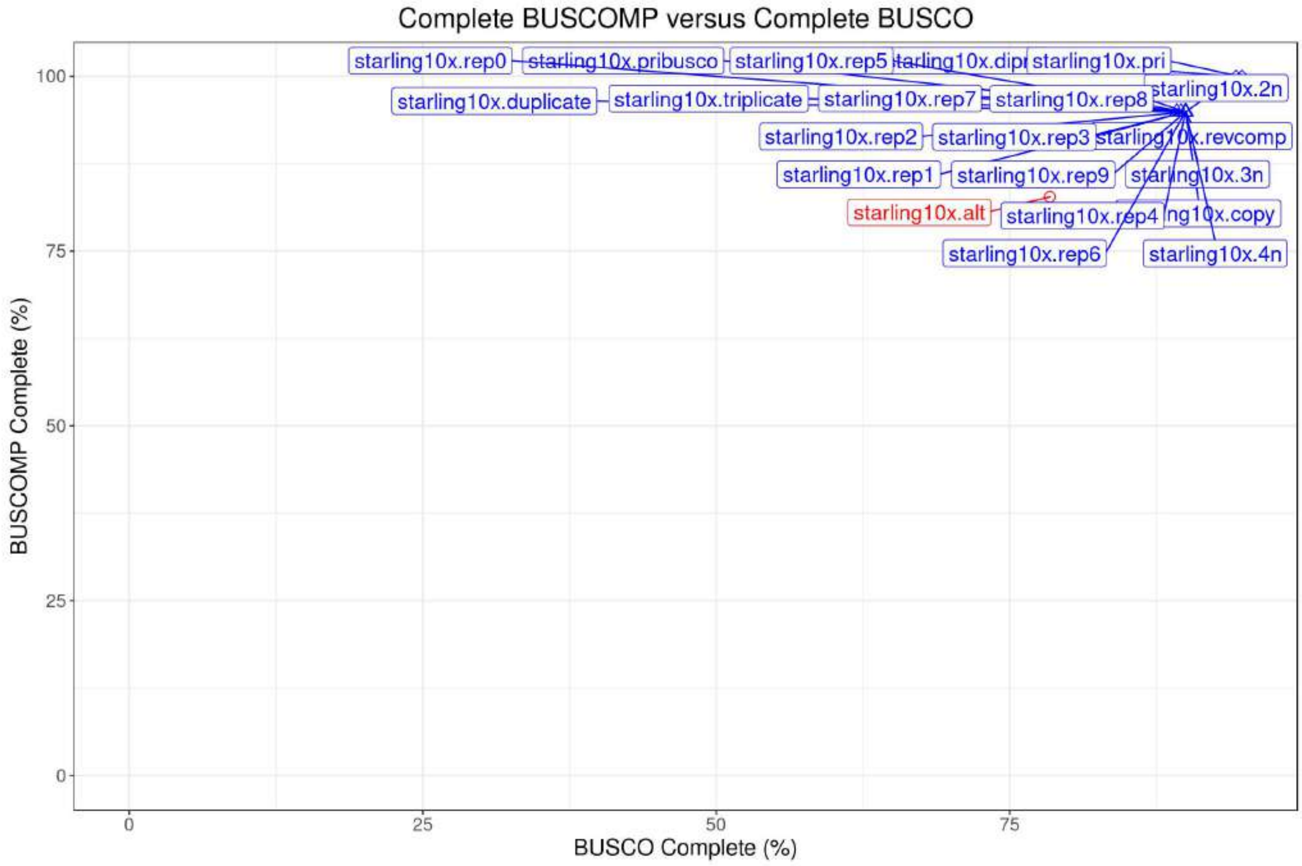

NOTE: To modify these plots and tables, edit the `*.genomes.tdt` and `*.NxLxxIDxx.rdata.tdt` files and re-knit the `*.NxLxxIDxx.Rmd` file.

3 BUSCO Ratings

Compiled BUSCO results for 24 assemblies and 6 groups have been saved in `starling10xV5.genomes.tdt`. BUSCO ratings are defined (quoting from the **BUSCO v3 User Guide (http://gitlab.com/ezlab/busco/raw/master/BUSCO_v3_userguide.pdf)** as:

- `Complete` : Single-copy hits where "BUSCO matches have scored within the expected range of scores and within the expected range of length alignments to the BUSCO profile."
- `Duplicated` : As `Complete` but 2+ copies.
- `Fragmented` : "BUSCO matches … within the range of scores but not within the range of length alignments to the BUSCO profile."
- `Missing` : "Either no significant matches at all, or the BUSCO matches scored below the range of scores for the BUSCO profile."

Genome	N	Complete	Single	Duplicated	Fragmented	Missing
starling10x.dipnr	8338	7862	1545	6317	162	314
starling10x.pri	8338	7903	7783	120	159	276
starling10x.alt	8338	6539	6527	12	90	1709
Pseudodip	8338	7946	7841	105	157	235
starling10x.pribusco	8338	7501	7431	70	131	706
starling10x.revcomp	8338	7507	7436	71	135	696
Core	8338	8057	7958	99	119	162
starling10x.copy	8338	7503	7433	70	131	704
starling10x.duplicate	8338	7440	176	7264	131	767
starling10x.triplicate	8338	7471	358	7113	86	781
Duplicate	8338	7630	7553	77	87	621
starling10x.shuffle1	0	0	0	0	0	0
starling10x.shuffle2	0	0	0	0	0	0
starling10x.shuffle3	0	0	0	0	0	0
starling10x.2n	8338	7503	7435	68	129	706
starling10x.3n	8338	7505	7437	68	128	705
starling10x.4n	8338	7505	7437	68	127	706
Size	8338	7505	7437	68	128	705
starling10x.rep0	8338	7501	7431	70	131	706
starling10x.rep1	8338	7501	7431	70	131	706

Genome	N	Complete	Single	Duplicated	Fragmented	Missing
starling10x.rep2	8338	7501	7431	70	131	706
starling10x.rep3	8338	7501	7431	70	131	706
starling10x.rep4	8338	7501	7431	70	131	706
starling10x.rep5	8338	7501	7431	70	131	706
starling10x.rep6	8338	7501	7431	70	131	706
starling10x.rep7	8338	7501	7431	70	131	706
starling10x.rep8	8338	7501	7431	70	130	707
starling10x.rep9	8338	7501	7431	70	131	706
Replicate	8338	7501	7431	70	131	706
BUSCOMP	8338	8065	7963	102	116	157

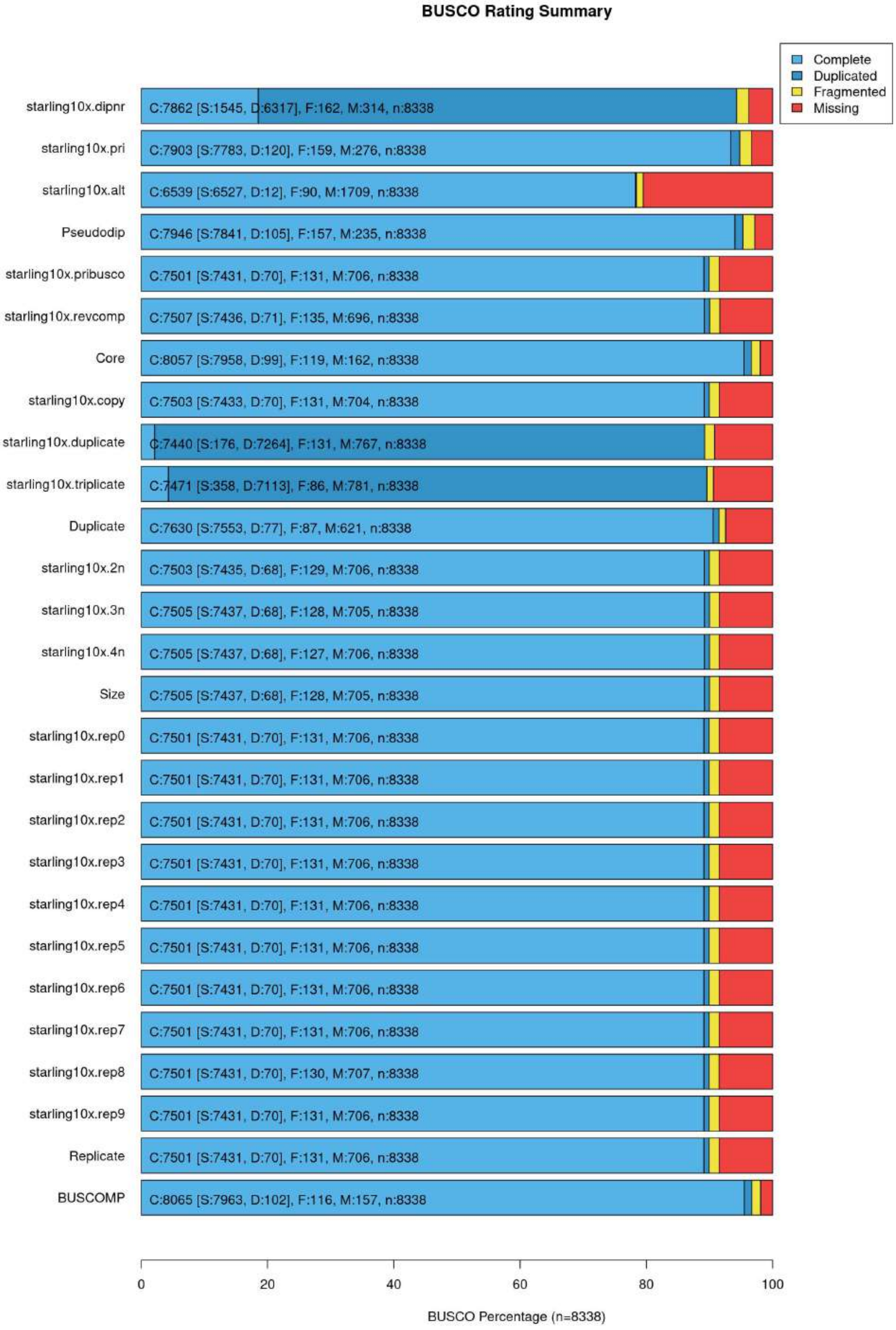

3.1 Genome Groups

`BUSCOMP` compiled the following groups of genomes (where BUSCO data was loaded), keeping the "best" rating for each BUSCO gene across the group:

- **Pseudodip**: `starling10x.dipnr` `starling10x.pri` `starling10x.alt`
- **Core**: `starling10x.dipnr` `starling10x.pri` `starling10x.alt` `starling10x.pribusco` `starling10x.revcomp`
- **Duplicate**: `starling10x.copy` `starling10x.duplicate` `starling10x.triplicate`
- **Size**: (`starling10x.shuffle1`) (`starling10x.shuffle2`) (`starling10x.shuffle3`) `starling10x.2n` `starling10x.3n` `starling10x.4n`

- **Replicate**: `starling10x.rep0` `starling10x.rep1` `starling10x.rep2` `starling10x.rep3` `starling10x.rep4` `starling10x.rep5` `starling10x.rep6` `starling10x.rep7` `starling10x.rep8` `starling10x.rep9`
- **BUSCOMP**: `starling10x.dipnr` `starling10x.pri` `starling10x.alt` `starling10x.pribusco` `starling10x.revcomp` `starling10x.copy` `starling10x.duplicate` `starling10x.triplicate` (`starling10x.shuffle1`)(`starling10x.shuffle2`)(`starling10x.shuffle3`) `starling10x.2n` `starling10x.3n` `starling10x.4n` `starling10x.rep0` `starling10x.rep1` `starling10x.rep2` `starling10x.rep3` `starling10x.rep4` `starling10x.rep5` `starling10x.rep6` `starling10x.rep7` `starling10x.rep8` `starling10x.rep9`

3.2 BUSCO Summary

```
starling10x.dipnr BUSCO Results:
        C:94.3%[S:18.5%,D:75.8%],F:1.9%,M:3.8%,n:8338

starling10x.pri BUSCO Results:
        C:94.8%[S:93.3%,D:1.4%],F:1.9%,M:3.3%,n:8338

starling10x.alt BUSCO Results:
        C:78.4%[S:78.3%,D:0.1%],F:1.1%,M:20.5%,n:8338

Pseudodip BUSCO Results:
        C:95.3%[S:94.0%,D:1.3%],F:1.9%,M:2.8%,n:8338

starling10x.pribusco BUSCO Results:
        C:90.0%[S:89.1%,D:0.8%],F:1.6%,M:8.5%,n:8338

starling10x.revcomp BUSCO Results:
        C:90.0%[S:89.2%,D:0.9%],F:1.6%,M:8.3%,n:8338

Core BUSCO Results:
        C:96.6%[S:95.4%,D:1.2%],F:1.4%,M:1.9%,n:8338

starling10x.copy BUSCO Results:
        C:90.0%[S:89.1%,D:0.8%],F:1.6%,M:8.4%,n:8338

starling10x.duplicate BUSCO Results:
        C:89.2%[S:2.1%,D:87.1%],F:1.6%,M:9.2%,n:8338

starling10x.triplicate BUSCO Results:
        C:89.6%[S:4.3%,D:85.3%],F:1.0%,M:9.4%,n:8338

Duplicate BUSCO Results:
        C:91.5%[S:90.6%,D:0.9%],F:1.0%,M:7.4%,n:8338

starling10x.shuffle1 BUSCO Results:
        C:0.0%[S:0.0%,D:0.0%],F:0.0%,M:0.0%,n:0

starling10x.shuffle2 BUSCO Results:
        C:0.0%[S:0.0%,D:0.0%],F:0.0%,M:0.0%,n:0

starling10x.shuffle3 BUSCO Results:
        C:0.0%[S:0.0%,D:0.0%],F:0.0%,M:0.0%,n:0

starling10x.2n BUSCO Results:
        C:90.0%[S:89.2%,D:0.8%],F:1.5%,M:8.5%,n:8338

starling10x.3n BUSCO Results:
        C:90.0%[S:89.2%,D:0.8%],F:1.5%,M:8.5%,n:8338

starling10x.4n BUSCO Results:
        C:90.0%[S:89.2%,D:0.8%],F:1.5%,M:8.5%,n:8338

Size BUSCO Results:
        C:90.0%[S:89.2%,D:0.8%],F:1.5%,M:8.5%,n:8338

starling10x.rep0 BUSCO Results:
        C:90.0%[S:89.1%,D:0.8%],F:1.6%,M:8.5%,n:8338

starling10x.rep1 BUSCO Results:
        C:90.0%[S:89.1%,D:0.8%],F:1.6%,M:8.5%,n:8338

starling10x.rep2 BUSCO Results:
        C:90.0%[S:89.1%,D:0.8%],F:1.6%,M:8.5%,n:8338

starling10x.rep3 BUSCO Results:
        C:90.0%[S:89.1%,D:0.8%],F:1.6%,M:8.5%,n:8338

starling10x.rep4 BUSCO Results:
        C:90.0%[S:89.1%,D:0.8%],F:1.6%,M:8.5%,n:8338

starling10x.rep5 BUSCO Results:
        C:90.0%[S:89.1%,D:0.8%],F:1.6%,M:8.5%,n:8338

starling10x.rep6 BUSCO Results:
        C:90.0%[S:89.1%,D:0.8%],F:1.6%,M:8.5%,n:8338

starling10x.rep7 BUSCO Results:
        C:90.0%[S:89.1%,D:0.8%],F:1.6%,M:8.5%,n:8338

starling10x.rep8 BUSCO Results:
        C:90.0%[S:89.1%,D:0.8%],F:1.6%,M:8.5%,n:8338

starling10x.rep9 BUSCO Results:
```

```
        C:90.0%[S:89.1%,D:0.8%],F:1.6%,M:8.5%,n:8338

Replicate BUSCO Results:
        C:90.0%[S:89.1%,D:0.8%],F:1.6%,M:8.5%,n:8338

BUSCOMP BUSCO Results:
        C:96.7%[S:95.5%,D:1.2%],F:1.4%,M:1.9%,n:8338
```

3.3 BUSCO Gene Details

Full BUSCO results with ratings for each gene have been compiled in `starling10xV5.busco.tdt`:

BuscoID <chr>	starling10x.dipnr <chr>	starling10x.pri <chr>
10004at8782	Duplicated	Complete
10005at8782	Duplicated	Complete
10020at8782	Duplicated	Complete
10021at8782	Duplicated	Complete
10029at8782	Complete	Complete
10031at8782	Duplicated	Complete
10032at8782	Duplicated	Complete
10042at8782	Duplicated	Complete
10045at8782	Duplicated	Complete
10049at8782	Duplicated	Complete

1-10 of 8,338 rows | 1-3 of 28 columns Previous 1 2 3 4 5 6 ... 834 Next

3.4 Genome Group BUSCO charts

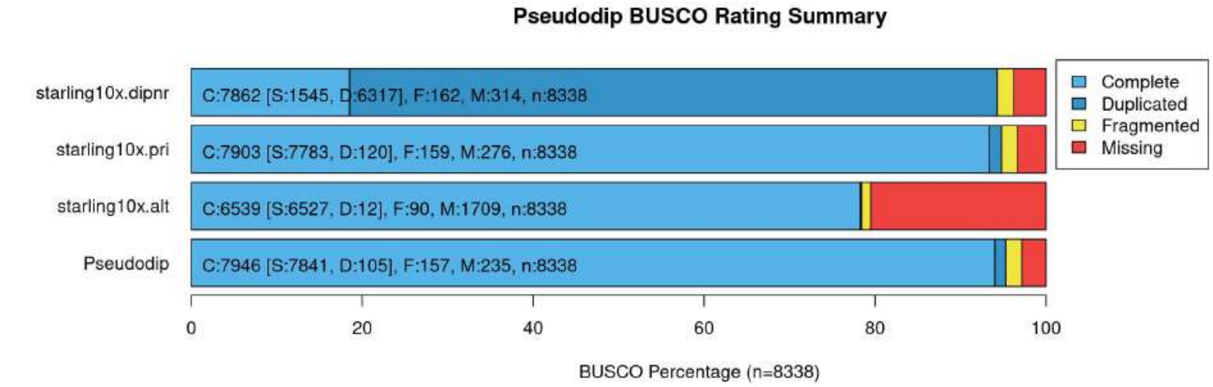

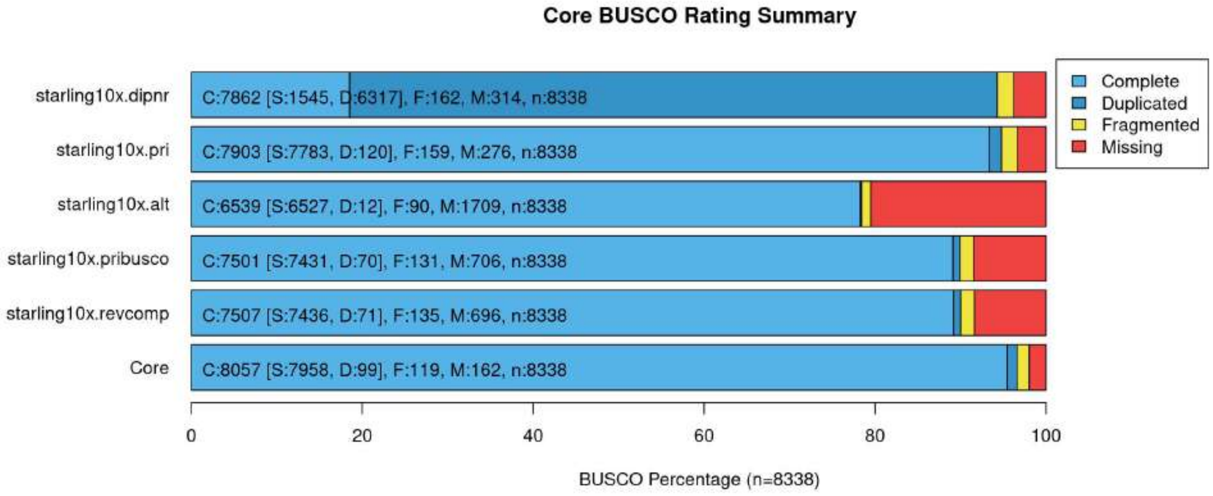

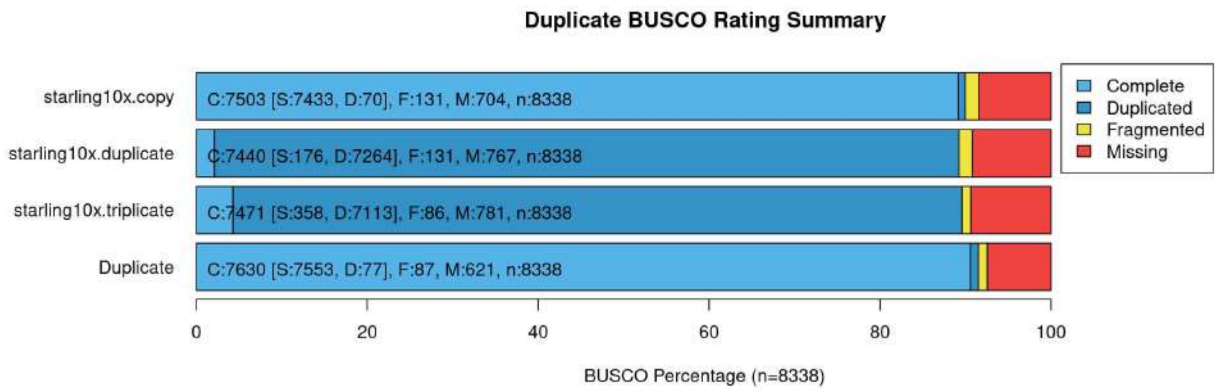
Duplicate BUSCO Rating Summary
starling10x.copy
C:7503 [S:7433, D:70], F:131, M:704, n:8338
starling10x.duplicate
C:7440 [S:176, D:7264], F:131, M:767, n:8338
starling10x.triplicate
C:7471 [S:358, D:7113], F:86, M:781, n:8338
Duplicate
C:7630 [S:7553, D:77], F:87, M:621, n:8338
Complete
Duplicated
Fragmented
Missing
0
20
40
60
80
100
BUSCO Percentage (n=8338)

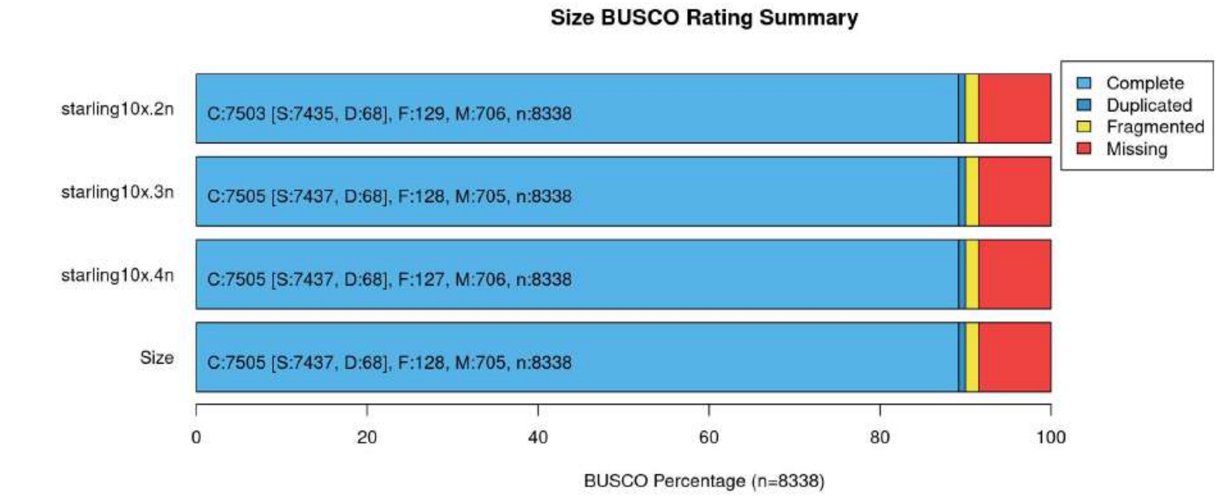
Size BUSCO Rating Summary
starling10x.2n
C:7503 [S:7435, D:68], F:129, M:706, n:8338
starling10x.3n
C:7505 [S:7437, D:68], F:128, M:705, n:8338
starling10x.4n
C:7505 [S:7437, D:68], F:127, M:706, n:8338
Size
C:7505 [S:7437, D:68], F:128, M:705, n:8338
Complete
Duplicated
Fragmented
Missing
0
20
40
60
80
100
BUSCO Percentage (n=8338)

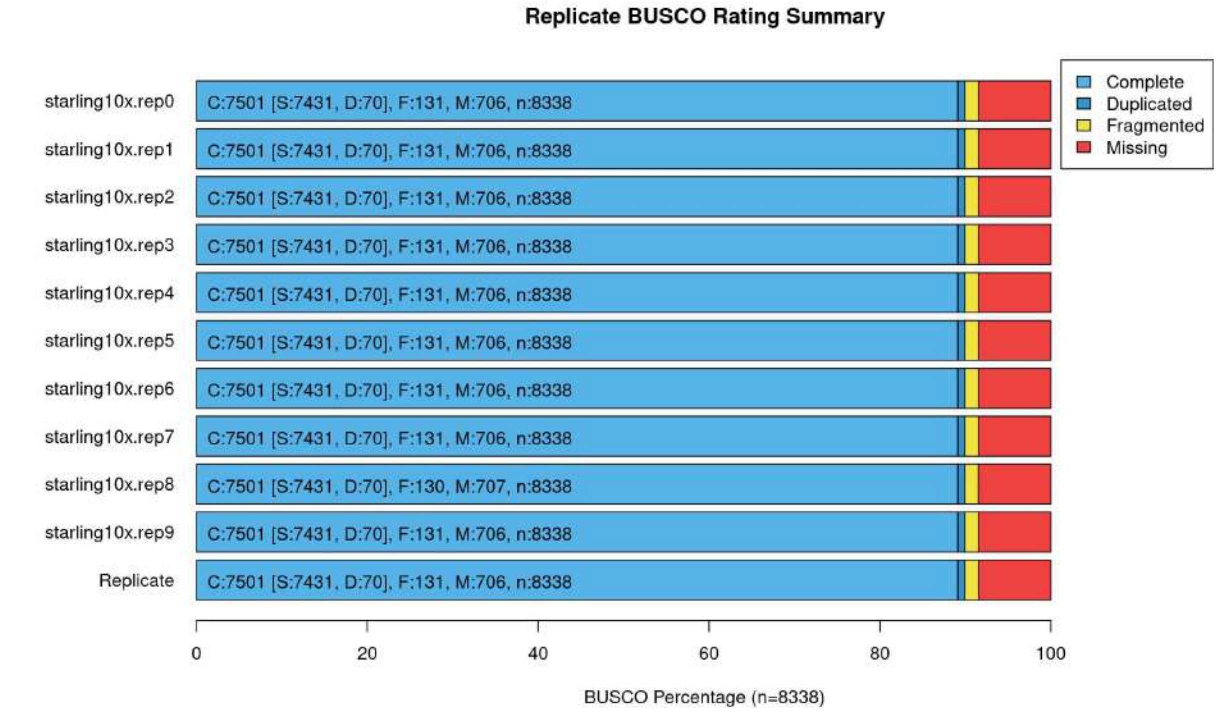
Replicate BUSCO Rating Summary
starling10x.rep0
C:7501 [S:7431, D:70], F:131, M:706, n:8338
starling10x.rep1
C:7501 [S:7431, D:70], F:131, M:706, n:8338
starling10x.rep2
C:7501 [S:7431, D:70], F:131, M:706, n:8338
starling10x.rep3
C:7501 [S:7431, D:70], F:131, M:706, n:8338
starling10x.rep4
C:7501 [S:7431, D:70], F:131, M:706, n:8338
starling10x.rep5
C:7501 [S:7431, D:70], F:131, M:706, n:8338
starling10x.rep6
C:7501 [S:7431, D:70], F:131, M:706, n:8338
starling10x.rep7
C:7501 [S:7431, D:70], F:131, M:706, n:8338
starling10x.rep8
C:7501 [S:7431, D:70], F:130, M:707, n:8338
starling10x.rep9
C:7501 [S:7431, D:70], F:131, M:706, n:8338
Replicate
C:7501 [S:7431, D:70], F:131, M:706, n:8338
Complete
Duplicated
Fragmented
Missing
0
20
40
60
80
100
BUSCO Percentage (n=8338)

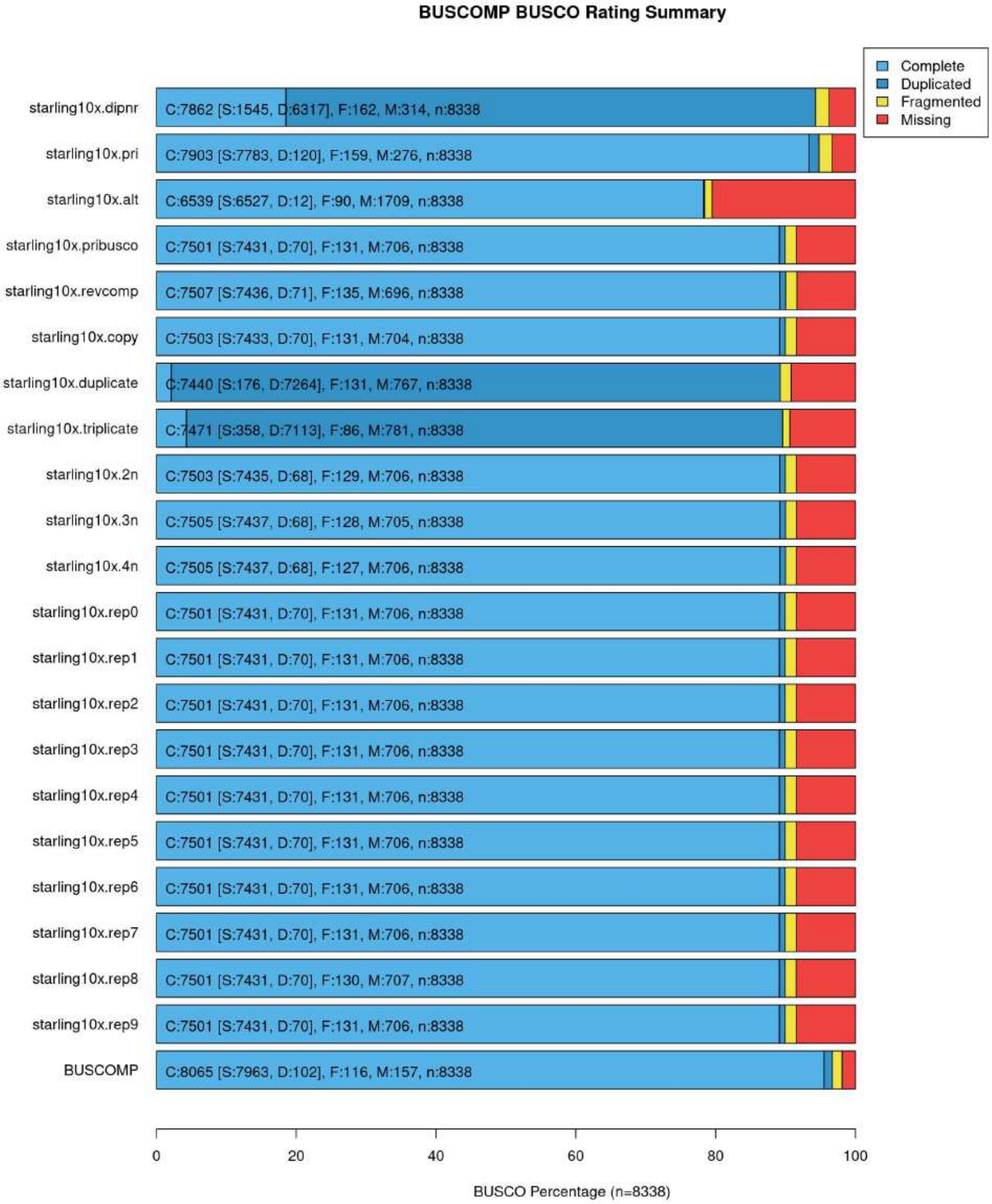

4 BUSCOMP Ratings

The best complete BUSCO hit results (based on `Score` and `Length`) have been compiled in `starling10xV5.buscoseq.tdt`. The `Genome` field indicates the assembly with the best hit, which is followed by details of that hit (`Contig`, `Start`, `End`, `Score`, `Length`). BUSCOMP ratings for each assembly are then given in subsequent fields:

```
* `Identical`: 100% coverage and 100% identity in at least one contig/scaffold.
* `Complete`: 95%+ Coverage in a single contig/scaffold. (Note: accuracy/identity is not considered.)
* `Duplicated`: 95%+ Coverage in 2+ contigs/scaffolds.
* `Fragmented`: 95%+ combined coverage but not in any single contig/scaffold.
* `Partial`: 40-95% combined coverage.
* `Ghost`: Hits meeting local cutoff but <40% combined coverage.
* `Missing`: No hits meeting local cutoff.
```

Genome <chr>	BuscoID <chr>	Status <chr>
starling10x.2n	10004at8782	Complete
starling10x.2n	10005at8782	Complete

Genome <chr>	BuscoID <chr>	Status <chr>
starling10x.alt	10020at8782	Complete
starling10x.revcomp	10021at8782	Complete
starling10x.2n	10029at8782	Complete
starling10x.alt	10031at8782	Complete
starling10x.alt	10032at8782	Complete
starling10x.revcomp	10042at8782	Complete
starling10x.2n	10045at8782	Complete
starling10x.revcomp	10049at8782	Complete

1-10 of 8,338 rows | 1-3 of 11 columns Previous 1 2 3 4 5 6 ... 834 Next

4.1 BUSCOSeq Rating Summary

BUSCOMP ratings (see above) are compiled to summary statistics in `starling10xV5.N3L20ID0U.ratings.tdt`. Note that `Identical` ratings in this table will also be rated as `Complete`, which in turn are `Single` or `Duplicated`. Percentage summaries are plotted below, along with a BUSCO-style one-line summary per assembly/group.

NOTE: Group summaries do not include `Identical` ratings.

X.	Genome	N	Identical	Complete	Single	Duplicated	Fragmented	Partial	Ghost	Missing
1	starling10x.dipnr	7963	6847	7957	1350	6607	0	6	0	0
2	starling10x.pri	7963	5972	7957	7925	32	0	6	0	0
3	starling10x.alt	7963	2629	6589	6580	9	0	39	29	1306
4	Pseudodip	7963	0	7957	7930	27	0	6	0	0
5	starling10x.pribusco	7963	5720	7555	7536	19	0	17	9	382
6	starling10x.revcomp	7963	5714	7554	7535	19	1	16	9	383
7	Core	7963	0	7957	7935	22	0	6	0	0
8	starling10x.copy	7963	5720	7555	7536	19	0	17	9	382
9	starling10x.duplicate	7963	5720	7555	0	7555	0	17	9	382
10	starling10x.triplicate	7963	5729	7555	0	7555	0	17	9	382
11	Duplicate	7963	0	7555	7536	19	0	17	9	382
12	starling10x.shuffle1	7963	0	0	0	0	0	0	0	7963
13	starling10x.shuffle2	7963	0	0	0	0	0	0	0	7963
14	starling10x.shuffle3	7963	0	0	0	0	0	0	0	7963
15	starling10x.2n	7963	5720	7555	7535	20	0	17	9	382
16	starling10x.3n	7963	5720	7555	7535	20	0	17	9	382
17	starling10x.4n	7963	5720	7555	7536	19	0	17	9	382
18	Size	7963	0	7555	7536	19	0	17	9	382
19	starling10x.rep0	7963	5720	7555	7536	19	0	17	9	382
20	starling10x.rep1	7963	5720	7555	7536	19	0	17	9	382
21	starling10x.rep2	7963	5720	7555	7536	19	0	17	9	382
22	starling10x.rep3	7963	5720	7555	7536	19	0	17	9	382
23	starling10x.rep4	7963	5720	7555	7536	19	0	17	9	382
24	starling10x.rep5	7963	5720	7555	7536	19	0	17	9	382
25	starling10x.rep6	7963	5720	7555	7536	19	0	17	9	382
26	starling10x.rep7	7963	5720	7555	7536	19	0	17	9	382
27	starling10x.rep8	7963	5720	7555	7536	19	0	17	9	382
28	starling10x.rep9	7963	5720	7555	7536	19	0	17	9	382
29	Replicate	7963	0	7555	7536	19	0	17	9	382
30	BUSCOMP	7963	0	7957	7935	22	0	6	0	0

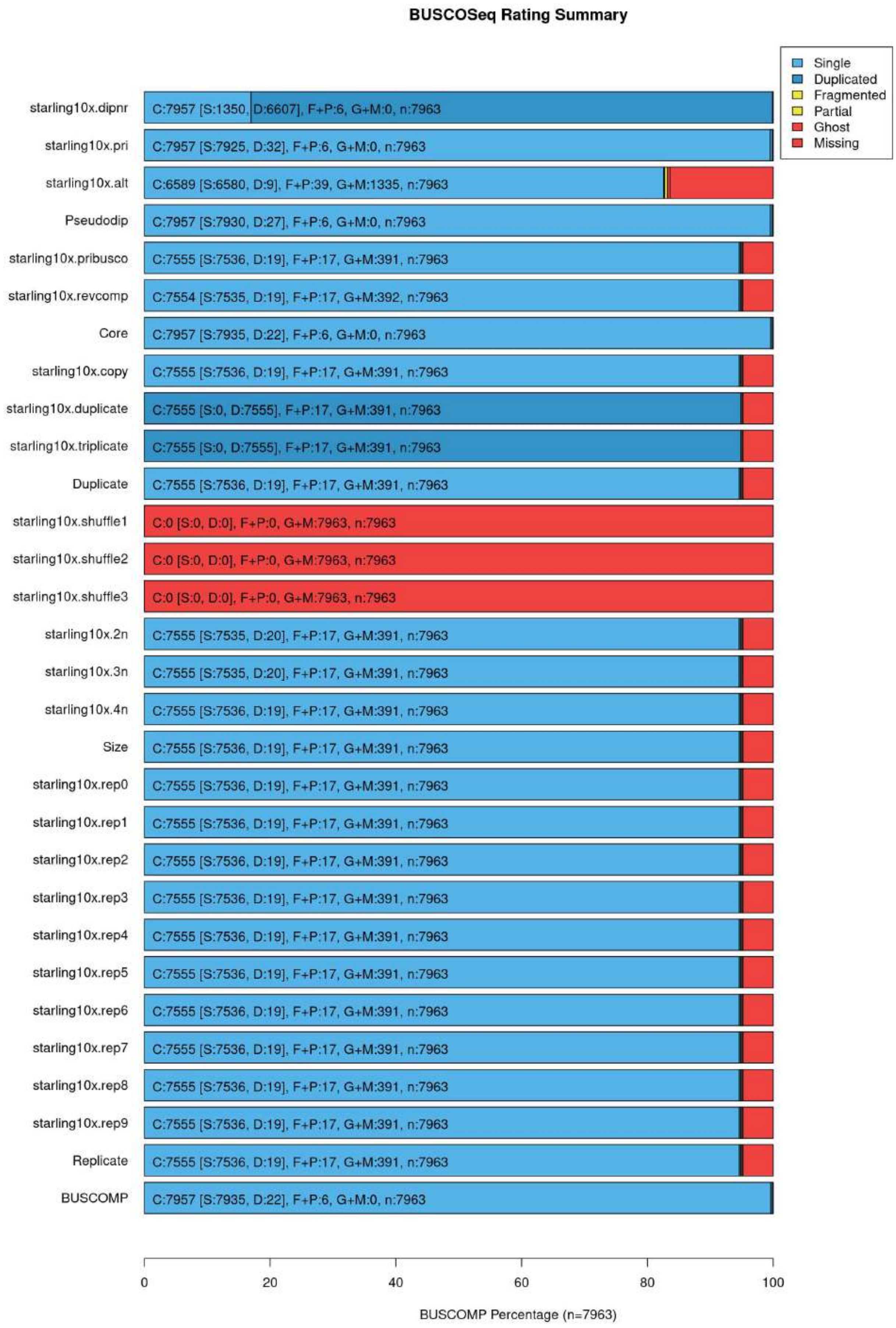
BUSCOSeq Rating Summary
Single
Duplicated
Fragmented
Partial
Ghost
Missing
starling10x.dipnr C:7957 [S:1350, D:6607], F+P:6, G+M:0, n:7963
starling10x.pri C:7957 [S:7925, D:32], F+P:6, G+M:0, n:7963
starling10x.alt C:6589 [S:6580, D:9], F+P:39, G+M:1335, n:7963
Pseudodip C:7957 [S:7930, D:27], F+P:6, G+M:0, n:7963
starling10x.pribusco C:7555 [S:7536, D:19], F+P:17, G+M:391, n:7963
starling10x.revcomp C:7554 [S:7535, D:19], F+P:17, G+M:392, n:7963
Core C:7957 [S:7935, D:22], F+P:6, G+M:0, n:7963
starling10x.copy C:7555 [S:7536, D:19], F+P:17, G+M:391, n:7963
starling10x.duplicate C:7555 [S:0, D:7555], F+P:17, G+M:391, n:7963
starling10x.triplicate C:7555 [S:0, D:7555], F+P:17, G+M:391, n:7963
Duplicate C:7555 [S:7536, D:19], F+P:17, G+M:391, n:7963
starling10x.shuffle1 C:0 [S:0, D:0], F+P:0, G+M:7963, n:7963
starling10x.shuffle2 C:0 [S:0, D:0], F+P:0, G+M:7963, n:7963
starling10x.shuffle3 C:0 [S:0, D:0], F+P:0, G+M:7963, n:7963
starling10x.2n C:7555 [S:7535, D:20], F+P:17, G+M:391, n:7963
starling10x.3n C:7555 [S:7535, D:20], F+P:17, G+M:391, n:7963
starling10x.4n C:7555 [S:7536, D:19], F+P:17, G+M:391, n:7963
Size C:7555 [S:7536, D:19], F+P:17, G+M:391, n:7963
starling10x.rep0 C:7555 [S:7536, D:19], F+P:17, G+M:391, n:7963
starling10x.rep1 C:7555 [S:7536, D:19], F+P:17, G+M:391, n:7963
starling10x.rep2 C:7555 [S:7536, D:19], F+P:17, G+M:391, n:7963
starling10x.rep3 C:7555 [S:7536, D:19], F+P:17, G+M:391, n:7963
starling10x.rep4 C:7555 [S:7536, D:19], F+P:17, G+M:391, n:7963
starling10x.rep5 C:7555 [S:7536, D:19], F+P:17, G+M:391, n:7963
starling10x.rep6 C:7555 [S:7536, D:19], F+P:17, G+M:391, n:7963
starling10x.rep7 C:7555 [S:7536, D:19], F+P:17, G+M:391, n:7963
starling10x.rep8 C:7555 [S:7536, D:19], F+P:17, G+M:391, n:7963
starling10x.rep9 C:7555 [S:7536, D:19], F+P:17, G+M:391, n:7963
Replicate C:7555 [S:7536, D:19], F+P:17, G+M:391, n:7963
BUSCOMP C:7957 [S:7935, D:22], F+P:6, G+M:0, n:7963
0
20
40
60
80
100
BUSCOMP Percentage (n=7963)

```
BUSCOMP BUSCOMP Results [8065 (96.73%) Complete BUSCOs; 0 (0.00%) BUSCOMP Seqs]:
        C:99.9%[S:99.6%,D:0.3%],F:0.0%,P:0.1%,G:0.0%,M:0.0%,n:7963

Core BUSCOMP Results [8057 (96.63%) Complete BUSCOs; 0 (0.00%) BUSCOMP Seqs]:
        C:99.9%[S:99.6%,D:0.3%],F:0.0%,P:0.1%,G:0.0%,M:0.0%,n:7963

Duplicate BUSCOMP Results [7630 (91.51%) Complete BUSCOs; 0 (0.00%) BUSCOMP Seqs]:
        C:94.9%[S:94.6%,D:0.2%],F:0.0%,P:0.2%,G:0.1%,M:4.8%,n:7963

Pseudodip BUSCOMP Results [7946 (95.30%) Complete BUSCOs; 0 (0.00%) BUSCOMP Seqs]:
        C:99.9%[S:99.6%,D:0.3%],F:0.0%,P:0.1%,G:0.0%,M:0.0%,n:7963

Replicate BUSCOMP Results [7501 (89.96%) Complete BUSCOs; 0 (0.00%) BUSCOMP Seqs]:
        C:94.9%[S:94.6%,D:0.2%],F:0.0%,P:0.2%,G:0.1%,M:4.8%,n:7963

Size BUSCOMP Results [7505 (90.01%) Complete BUSCOs; 0 (0.00%) BUSCOMP Seqs]:
        C:94.9%[S:94.6%,D:0.2%],F:0.0%,P:0.2%,G:0.1%,M:4.8%,n:7963


starling10x.2n BUSCOMP Results [7503 (89.99%) Complete BUSCOs; 5191 (65.19%) BUSCOMP Seqs]:
        C:94.9%[S:94.6%,D:0.3%,I:71.8%],F:0.0%,P:0.2%,G:0.1%,M:4.8%,n:7963

starling10x.3n BUSCOMP Results [7505 (90.01%) Complete BUSCOs; 1 (0.01%) BUSCOMP Seqs]:
        C:94.9%[S:94.6%,D:0.3%,I:71.8%],F:0.0%,P:0.2%,G:0.1%,M:4.8%,n:7963

starling10x.4n BUSCOMP Results [7505 (90.01%) Complete BUSCOs; 0 (0.00%) BUSCOMP Seqs]:
        C:94.9%[S:94.6%,D:0.2%,I:71.8%],F:0.0%,P:0.2%,G:0.1%,M:4.8%,n:7963

starling10x.alt BUSCOMP Results [6539 (78.42%) Complete BUSCOs; 1159 (14.55%) BUSCOMP Seqs]:
        C:82.7%[S:82.6%,D:0.1%,I:33.0%],F:0.0%,P:0.5%,G:0.4%,M:16.4%,n:7963

starling10x.copy BUSCOMP Results [7503 (89.99%) Complete BUSCOs; 59 (0.74%) BUSCOMP Seqs]:
        C:94.9%[S:94.6%,D:0.2%,I:71.8%],F:0.0%,P:0.2%,G:0.1%,M:4.8%,n:7963

starling10x.dipnr BUSCOMP Results [7862 (94.29%) Complete BUSCOs; 238 (2.99%) BUSCOMP Seqs]:
        C:99.9%[S:17.0%,D:83.0%,I:86.0%],F:0.0%,P:0.1%,G:0.0%,M:0.0%,n:7963

starling10x.duplicate BUSCOMP Results [7440 (89.23%) Complete BUSCOs; 5 (0.06%) BUSCOMP Seqs]:
        C:94.9%[S:0.0%,D:94.9%,I:71.8%],F:0.0%,P:0.2%,G:0.1%,M:4.8%,n:7963

starling10x.pri BUSCOMP Results [7903 (94.78%) Complete BUSCOs; 44 (0.55%) BUSCOMP Seqs]:
        C:99.9%[S:99.5%,D:0.4%,I:75.0%],F:0.0%,P:0.1%,G:0.0%,M:0.0%,n:7963

starling10x.pribusco BUSCOMP Results [7501 (89.96%) Complete BUSCOs; 3 (0.04%) BUSCOMP Seqs]:
        C:94.9%[S:94.6%,D:0.2%,I:71.8%],F:0.0%,P:0.2%,G:0.1%,M:4.8%,n:7963

starling10x.rep0 BUSCOMP Results [7501 (89.96%) Complete BUSCOs; 0 (0.00%) BUSCOMP Seqs]:
        C:94.9%[S:94.6%,D:0.2%,I:71.8%],F:0.0%,P:0.2%,G:0.1%,M:4.8%,n:7963

starling10x.rep1 BUSCOMP Results [7501 (89.96%) Complete BUSCOs; 0 (0.00%) BUSCOMP Seqs]:
        C:94.9%[S:94.6%,D:0.2%,I:71.8%],F:0.0%,P:0.2%,G:0.1%,M:4.8%,n:7963

starling10x.rep2 BUSCOMP Results [7501 (89.96%) Complete BUSCOs; 0 (0.00%) BUSCOMP Seqs]:
        C:94.9%[S:94.6%,D:0.2%,I:71.8%],F:0.0%,P:0.2%,G:0.1%,M:4.8%,n:7963

starling10x.rep3 BUSCOMP Results [7501 (89.96%) Complete BUSCOs; 0 (0.00%) BUSCOMP Seqs]:
        C:94.9%[S:94.6%,D:0.2%,I:71.8%],F:0.0%,P:0.2%,G:0.1%,M:4.8%,n:7963

starling10x.rep4 BUSCOMP Results [7501 (89.96%) Complete BUSCOs; 0 (0.00%) BUSCOMP Seqs]:
        C:94.9%[S:94.6%,D:0.2%,I:71.8%],F:0.0%,P:0.2%,G:0.1%,M:4.8%,n:7963

starling10x.rep5 BUSCOMP Results [7501 (89.96%) Complete BUSCOs; 0 (0.00%) BUSCOMP Seqs]:
        C:94.9%[S:94.6%,D:0.2%,I:71.8%],F:0.0%,P:0.2%,G:0.1%,M:4.8%,n:7963

starling10x.rep6 BUSCOMP Results [7501 (89.96%) Complete BUSCOs; 0 (0.00%) BUSCOMP Seqs]:
        C:94.9%[S:94.6%,D:0.2%,I:71.8%],F:0.0%,P:0.2%,G:0.1%,M:4.8%,n:7963

starling10x.rep7 BUSCOMP Results [7501 (89.96%) Complete BUSCOs; 0 (0.00%) BUSCOMP Seqs]:
        C:94.9%[S:94.6%,D:0.2%,I:71.8%],F:0.0%,P:0.2%,G:0.1%,M:4.8%,n:7963

starling10x.rep8 BUSCOMP Results [7501 (89.96%) Complete BUSCOs; 0 (0.00%) BUSCOMP Seqs]:
        C:94.9%[S:94.6%,D:0.2%,I:71.8%],F:0.0%,P:0.2%,G:0.1%,M:4.8%,n:7963

starling10x.rep9 BUSCOMP Results [7501 (89.96%) Complete BUSCOs; 0 (0.00%) BUSCOMP Seqs]:
        C:94.9%[S:94.6%,D:0.2%,I:71.8%],F:0.0%,P:0.2%,G:0.1%,M:4.8%,n:7963

starling10x.revcomp BUSCOMP Results [7507 (90.03%) Complete BUSCOs; 1251 (15.71%) BUSCOMP Seqs]:
        C:94.9%[S:94.6%,D:0.2%,I:71.8%],F:0.0%,P:0.2%,G:0.1%,M:4.8%,n:7963

starling10x.shuffle1 BUSCOMP Results [No BUSCO analysis]:
        C:0.0%[S:0.0%,D:0.0%,I:0.0%],F:0.0%,P:0.0%,G:0.0%,M:100.0%,n:7963

starling10x.shuffle2 BUSCOMP Results [No BUSCO analysis]:
```

```
        C:0.0%[S:0.0%,D:0.0%,I:0.0%],F:0.0%,P:0.0%,G:0.0%,M:100.0%,n:7963

starling10x.shuffle3 BUSCOMP Results [No BUSCO analysis]:
        C:0.0%[S:0.0%,D:0.0%,I:0.0%],F:0.0%,P:0.0%,G:0.0%,M:100.0%,n:7963

starling10x.triplicate BUSCOMP Results [7471 (89.60%) Complete BUSCOs; 12 (0.15%) BUSCOMP Seqs]:
        C:94.9%[S:0.0%,D:94.9%,I:71.9%],F:0.0%,P:0.2%,G:0.1%,M:4.8%,n:7963
```

4.2 BUSCOSeq Full Results Table

Full BUSCOMP results with ratings for each gene in every assembly and group have been compiled in `starling10xV5.N3L20ID0U.buscomp.tdt` :

BuscoID <chr>	Genome <chr>	starling10x.dipnr <chr>	starling10x.pri <chr>
10004at8782	BUSCOFas	Duplicated	Complete
10005at8782	BUSCOFas	Duplicated	Complete
10020at8782	BUSCOFas	Duplicated	Complete
10021at8782	BUSCOFas	Duplicated	Complete
10029at8782	BUSCOFas	Complete	Complete
10031at8782	BUSCOFas	Duplicated	Complete
10032at8782	BUSCOFas	Duplicated	Complete
10042at8782	BUSCOFas	Duplicated	Complete
10045at8782	BUSCOFas	Duplicated	Complete
10049at8782	BUSCOFas	Duplicated	Complete

1-10 of 7,963 rows | 1-4 of 32 columns Previous 1 2 3 4 5 6 ... 797 Next

4.3 Genome Group BUSCOMP charts

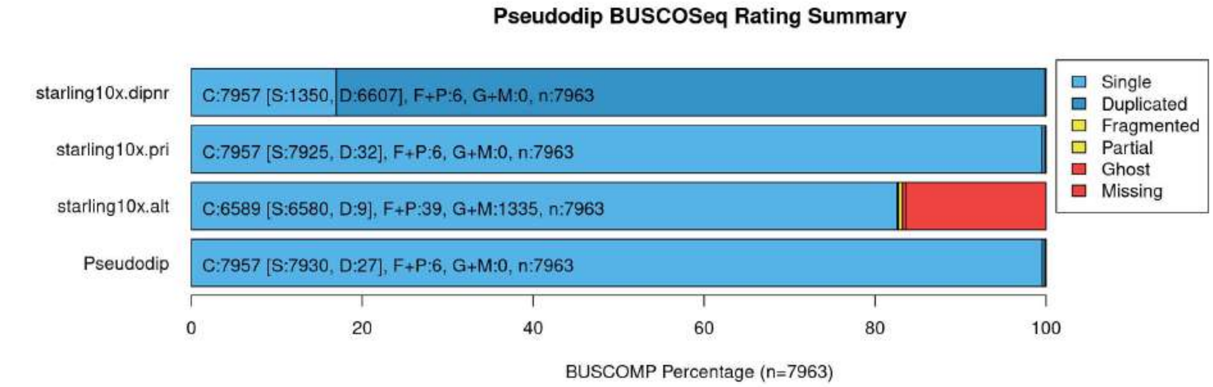

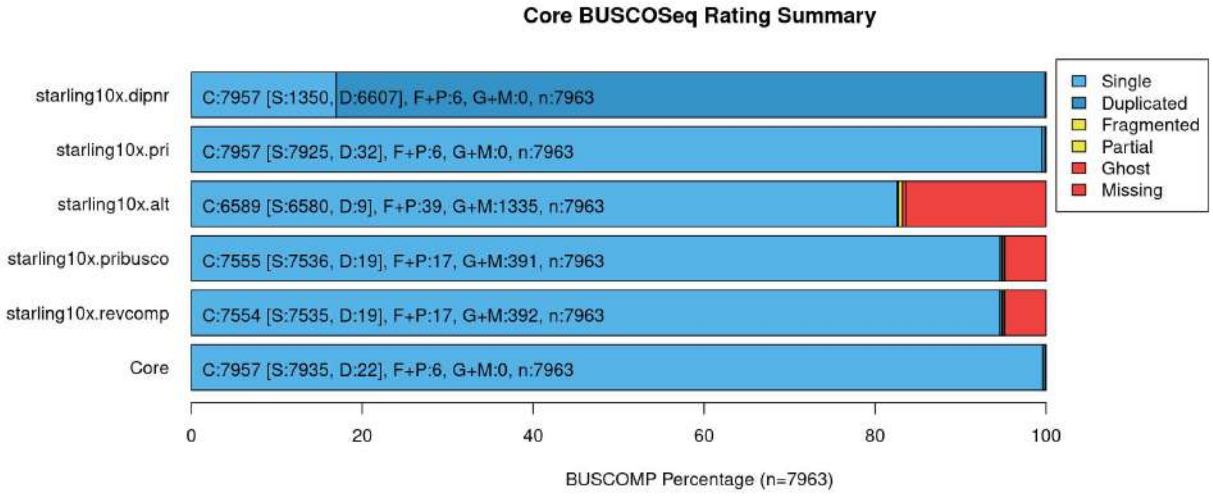

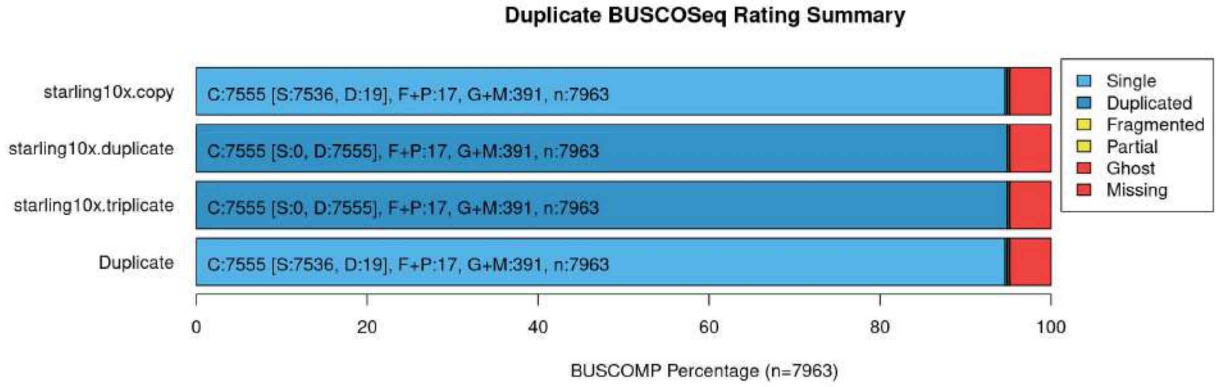
Duplicate BUSCOSeq Rating Summary
starling10x.copy
C:7555 [S:7536, D:19], F+P:17, G+M:391, n:7963
starling10x.duplicate
C:7555 [S:0, D:7555], F+P:17, G+M:391, n:7963
starling10x.triplicate
C:7555 [S:0, D:7555], F+P:17, G+M:391, n:7963
Duplicate
C:7555 [S:7536, D:19], F+P:17, G+M:391, n:7963
Single
Duplicated
Fragmented
Partial
Ghost
Missing
0
20
40
60
80
100
BUSCOMP Percentage (n=7963)

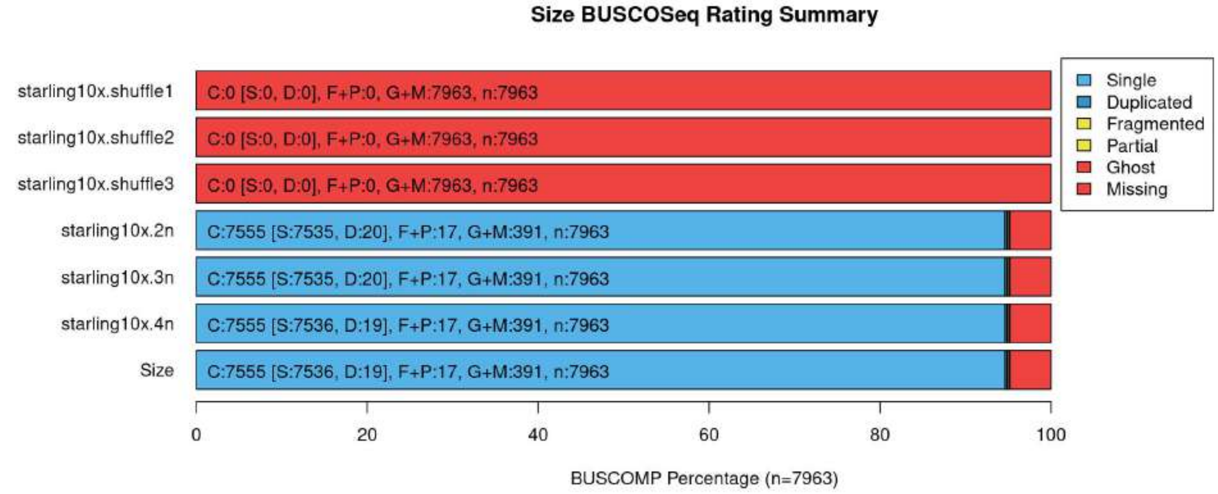
Size BUSCOSeq Rating Summary
starling10x.shuffle1
C:0 [S:0, D:0], F+P:0, G+M:7963, n:7963
starling10x.shuffle2
C:0 [S:0, D:0], F+P:0, G+M:7963, n:7963
starling10x.shuffle3
C:0 [S:0, D:0], F+P:0, G+M:7963, n:7963
starling10x.2n
C:7555 [S:7535, D:20], F+P:17, G+M:391, n:7963
starling10x.3n
C:7555 [S:7535, D:20], F+P:17, G+M:391, n:7963
starling10x.4n
C:7555 [S:7536, D:19], F+P:17, G+M:391, n:7963
Size
C:7555 [S:7536, D:19], F+P:17, G+M:391, n:7963
Single
Duplicated
Fragmented
Partial
Ghost
Missing
0
20
40
60
80
100
BUSCOMP Percentage (n=7963)

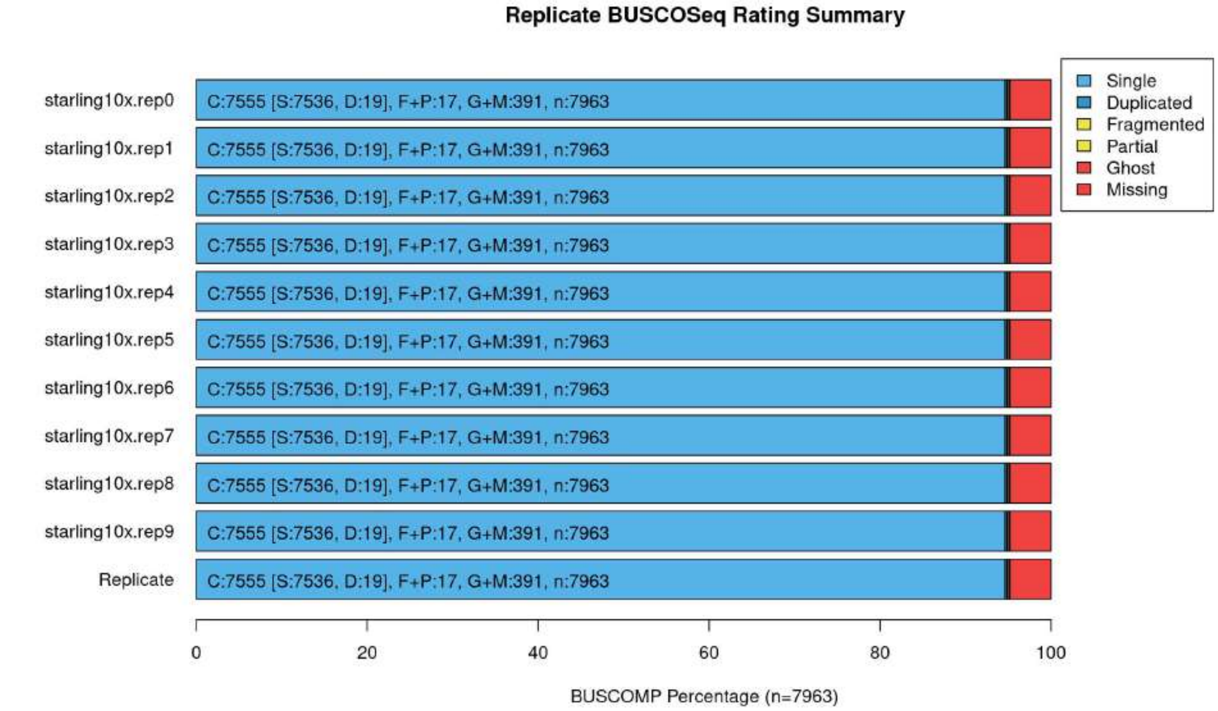
Replicate BUSCOSeq Rating Summary
starling10x.rep0
C:7555 [S:7536, D:19], F+P:17, G+M:391, n:7963
starling10x.rep1
C:7555 [S:7536, D:19], F+P:17, G+M:391, n:7963
starling10x.rep2
C:7555 [S:7536, D:19], F+P:17, G+M:391, n:7963
starling10x.rep3
C:7555 [S:7536, D:19], F+P:17, G+M:391, n:7963
starling10x.rep4
C:7555 [S:7536, D:19], F+P:17, G+M:391, n:7963
starling10x.rep5
C:7555 [S:7536, D:19], F+P:17, G+M:391, n:7963
starling10x.rep6
C:7555 [S:7536, D:19], F+P:17, G+M:391, n:7963
starling10x.rep7
C:7555 [S:7536, D:19], F+P:17, G+M:391, n:7963
starling10x.rep8
C:7555 [S:7536, D:19], F+P:17, G+M:391, n:7963
starling10x.rep9
C:7555 [S:7536, D:19], F+P:17, G+M:391, n:7963
Replicate
C:7555 [S:7536, D:19], F+P:17, G+M:391, n:7963
Single
Duplicated
Fragmented
Partial
Ghost
Missing
0
20
40
60
80
100
BUSCOMP Percentage (n=7963)

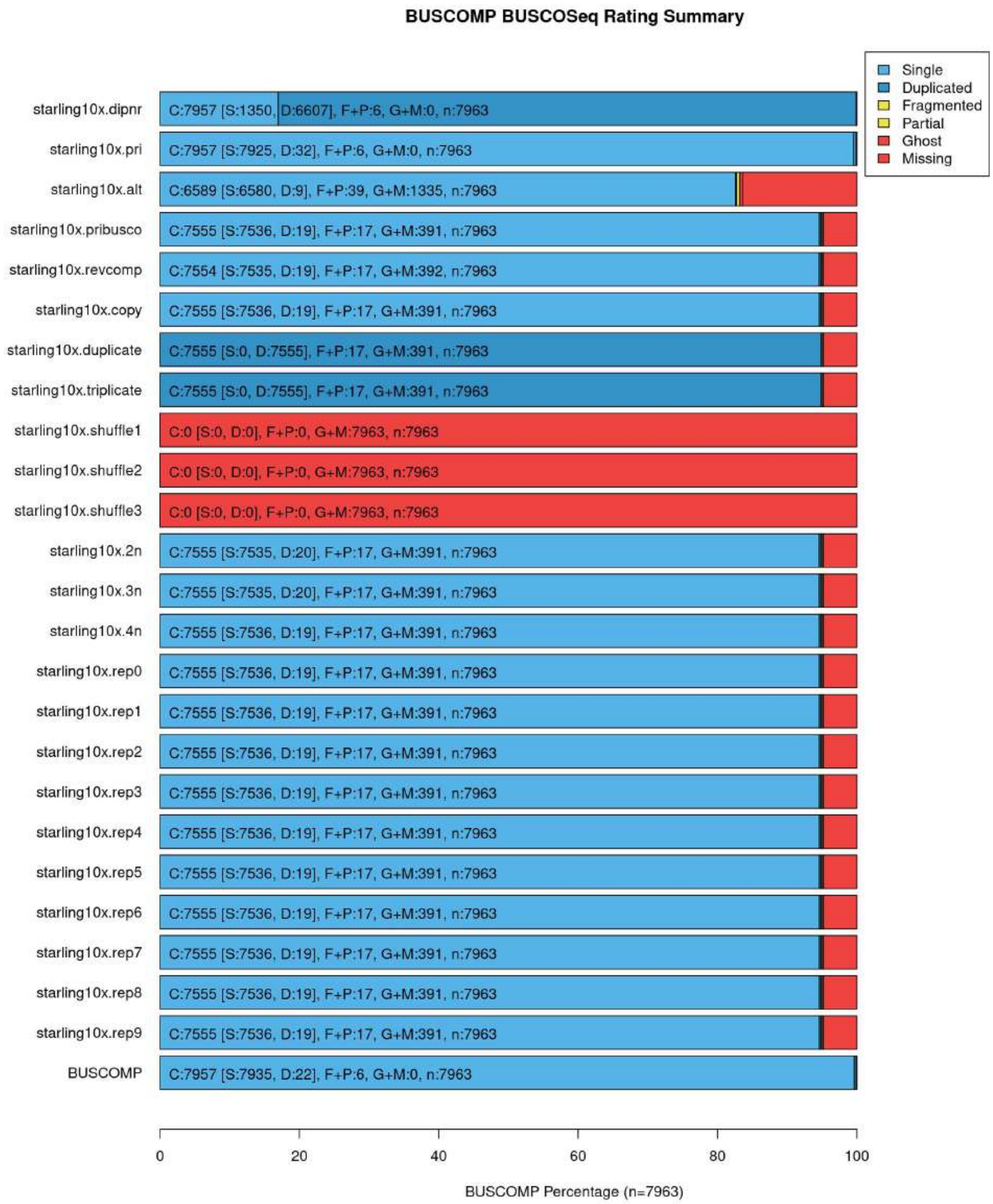

5 BUSCO and BUSCOMP Comparisons

5.1 BUSCO to BUSCOMP Rating Changes

Ratings changes from BUSCO to BUSCOMP (where `NULL` ratings indicate no BUSCOMP sequence):

BUSCO	BUSCOMP	starling10x.dipnr	starling10x.pri	starling10x.alt	starling10x.pribusco	starling10x.revcomp	starling10x.copy	starling10x.dup
Complete	Complete	1314	7760	6465	7402	7403	7404	
Complete	Duplicated	230	17	7	11	13	11	
Complete	Fragmented	0	0	0	0	1	0	
Complete	Ghost	0	0	1	0	0	0	
Complete	Missing	0	0	43	12	12	12	
Complete	Partial	1	6	11	6	7	6	
Duplicated	Complete	3	12	2	5	6	5	
Duplicated	Duplicated	6213	12	0	6	6	6	

BUSCO	BUSCOMP	starling10x.dipnr	starling10x.pri	starling10x.alt	starling10x.pribusco	starling10x.revcomp	starling10x.copy	starling10x.dup
Duplicated	Missing	0	0	4	2	2	2	
Duplicated	NULL	96	96	5	57	57	57	
Duplicated	Partial	5	0	1	0	0	0	
Fragmented	Complete	14	51	40	50	53	50	
Fragmented	Duplicated	40	0	0	0	0	0	
Fragmented	Missing	0	0	6	3	4	3	
Fragmented	NULL	108	108	44	77	78	77	
Fragmented	Partial	0	0	0	1	0	1	
Missing	Complete	19	102	73	79	73	77	
Missing	Duplicated	124	3	2	2	0	2	
Missing	Ghost	0	0	28	9	9	9	
Missing	Missing	0	0	1253	365	365	365	
Missing	NULL	171	171	326	241	240	241	
Missing	Partial	0	0	27	10	9	10	

Full table of Ratings changes from by gene:

BuscoID <chr>	starling10x.dipnr <chr>	starling10x.pri <chr>
10004at8782	DD	CC
10005at8782	DD	CC
10020at8782	DD	CC
10021at8782	DD	CC
10029at8782	CC	CC
10031at8782	DD	CC
10032at8782	DD	CC
10042at8782	DD	CC
10045at8782	DD	CC
10049at8782	DD	CC

1-10 of 8,338 rows | 1-3 of 22 columns Previous 1 2 3 4 5 6 ... 834 Next

Complete, **D**uplicated, **F**ragmented, **P**artial, **G**host, **M**issing, **N**ULL (no BUSCOMP sequence)

5.1.1 BUSCOMP Gain test

There is a risk that performing a low stringency search will identify homologues or pseudogenes of the desired BUSCO gene in error. If there is a second copy of a gene in the genome that is detectable by the search then we would expect the same genes that go from `Missing` to `Complete` in some genomes to go from `Single` to `Duplicated` in others.

To test this, data is reduced for each pair of genomes to BUSCO-BUSCOMP rating pairs of:

- `Single` - `Single`
- `Single` - `Duplicated`
- `Missing` - `Missing`
- `Missing` - `Single`

This is then converted in to `G` ain ratings (`Single` - `Duplicated` & `Missing` - `Single`) or `N` o Gain ratings (`Single` - `Single` & `Missing` - `Missing`). The `Single` - `Duplicated` shift in one genome is then used to set the expected `Missing` - `Single` shift in the other, and assess the probability of observing the `Missing` - `Single` shift using a cumulative binomial distribution, where:

- `k` is the number of observed `GG` pairs (`Single` - `Duplicated` *and* `Missing` - `Single`)
- `n` is the number of `Missing` - `Single` `G` ains in the focal genome (`NG` + `GG`)
- `p` is the proportion of `Single` - `Duplicated` `G` ains in the background genome (`GN` + `GG` / (`GN` + `GG` + `NN` + `NG`))
- `pB` is the probability of observing `k+` `Missing` - `Single` gains, given `p` and `n`

This is output to `*.gain.tdt` , where each row is a Genome and each field gives the probability of the row genome's `Missing` - `Single` gains, given the column genome's `Single` - `Duplicated` gains:

Genome <chr>	starling10x.dipnr <dbl>
starling10x.2n	4.23e-05
starling10x.3n	4.23e-05
starling10x.4n	4.23e-05
starling10x.alt	1.60e-14

Genome <chr>	starling10x.dipnr <dbl>
starling10x.copy	4.02e-06
starling10x.dipnr	1.00e+00
starling10x.duplicate	1.00e+00
starling10x.pri	7.38e-01
starling10x.pribusco	4.16e-07
starling10x.rep0	4.16e-07

1-10 of 21 rows | 1-2 of 22 columns Previous 1 2 3 Next

Low probabilities indicate that BUSCOMP might be rating paralogues or pseudogenes and not functional orthologues of the BUSCO gene. Note that there is *no* correction for multiple testing, nor any adjustment for lack of independence between samples.

5.2 Unique BUSCO and BUSCOMP Complete Genes

BUSCO and BUSCOMP `Complete` ratings were compared for each BUSCO gene to identify those genes unique to either a single assembly or a group of assemblies. The `BUSCOMP` group is excluded from this analysis, as (typically) are other redundant groups wholly contained within another group. (Inclusion of such groups is guaranteed to result in 2+ groups containing any `Complete` BUSCOs they have.)

```
starling10x.dipnr unique Complete genes: 0 BUSCO; 0 BUSCOMP
starling10x.pri unique Complete genes: 1 BUSCO; 0 BUSCOMP
starling10x.alt unique Complete genes: 4 BUSCO; 0 BUSCOMP
starling10x.pribusco unique Complete genes: 0 BUSCO; 0 BUSCOMP
starling10x.revcomp unique Complete genes: 1 BUSCO; 0 BUSCOMP
starling10x.copy unique Complete genes: 0 BUSCO; 0 BUSCOMP
starling10x.duplicate unique Complete genes: 3 BUSCO; 0 BUSCOMP
starling10x.triplicate unique Complete genes: 4 BUSCO; 0 BUSCOMP
starling10x.shuffle1 unique Complete genes: 0 BUSCO; 0 BUSCOMP
starling10x.shuffle2 unique Complete genes: 0 BUSCO; 0 BUSCOMP
starling10x.shuffle3 unique Complete genes: 0 BUSCO; 0 BUSCOMP
starling10x.2n unique Complete genes: 0 BUSCO; 0 BUSCOMP
starling10x.3n unique Complete genes: 0 BUSCO; 0 BUSCOMP
starling10x.4n unique Complete genes: 0 BUSCO; 0 BUSCOMP
starling10x.rep0 unique Complete genes: 0 BUSCO; 0 BUSCOMP
starling10x.rep1 unique Complete genes: 0 BUSCO; 0 BUSCOMP
starling10x.rep2 unique Complete genes: 0 BUSCO; 0 BUSCOMP
starling10x.rep3 unique Complete genes: 0 BUSCO; 0 BUSCOMP
starling10x.rep4 unique Complete genes: 0 BUSCO; 0 BUSCOMP
starling10x.rep5 unique Complete genes: 0 BUSCO; 0 BUSCOMP
starling10x.rep6 unique Complete genes: 0 BUSCO; 0 BUSCOMP
starling10x.rep7 unique Complete genes: 0 BUSCO; 0 BUSCOMP
starling10x.rep8 unique Complete genes: 0 BUSCO; 0 BUSCOMP
starling10x.rep9 unique Complete genes: 0 BUSCO; 0 BUSCOMP
Core unique Complete genes: 0 BUSCO; 0 BUSCOMP
Duplicate unique Complete genes: 0 BUSCO; 0 BUSCOMP
Pseudodip unique Complete genes: 0 BUSCO; 0 BUSCOMP
Replicate unique Complete genes: 0 BUSCO; 0 BUSCOMP
Size unique Complete genes: 0 BUSCO; 0 BUSCOMP
```

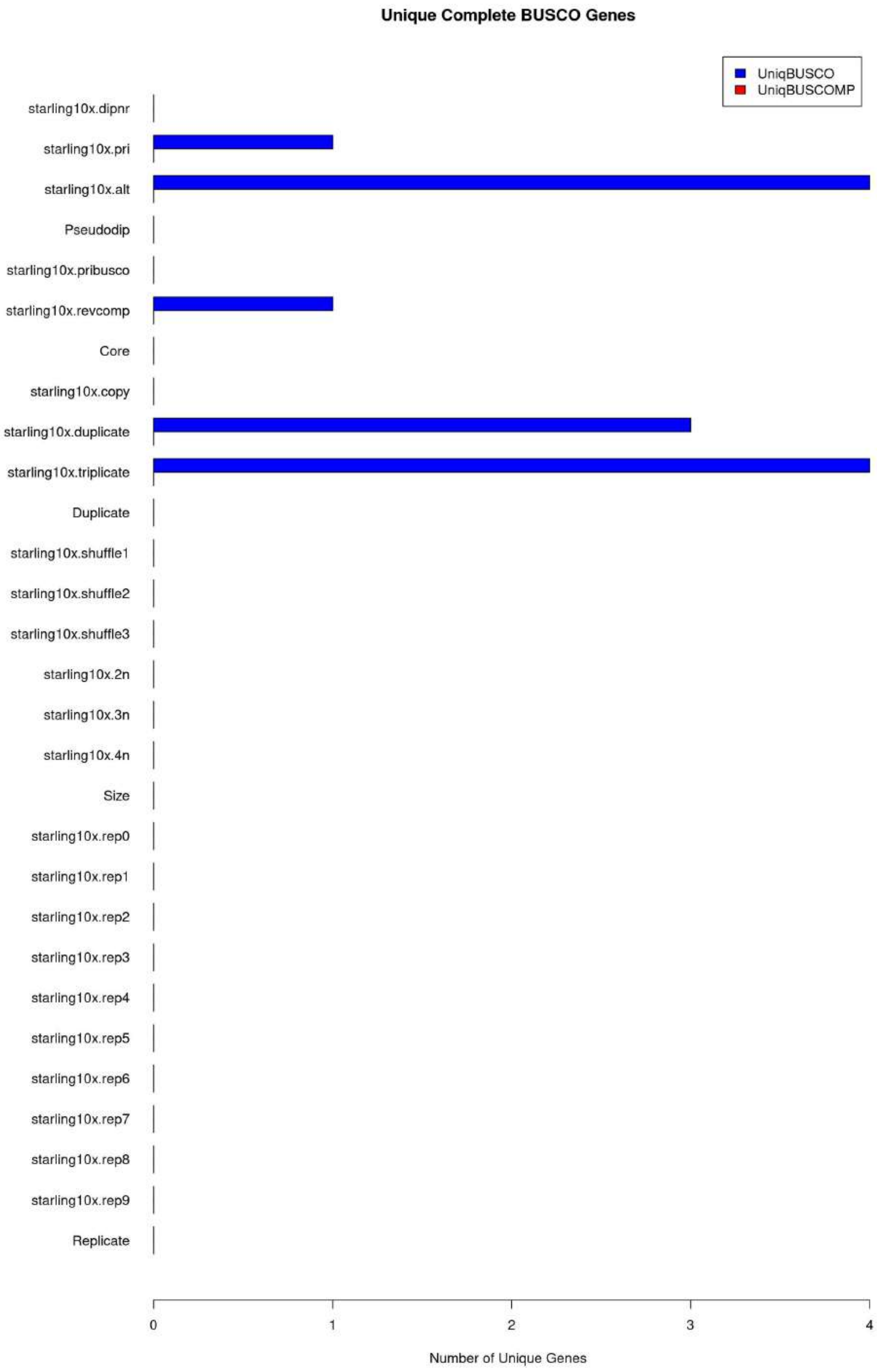

5.3 Ratings for Missing BUSCO genes

In addition to the unique ratings (above), it can be useful to know how genes `Missing` from one assembly/group are rated in the others. These plots are generated for each assembly/group in turn. The full BUSCO (`*.busco.tdt`) and BUSCOMP (`*.LnnIDxx.buscomp.tdt`) tables are reduced to the subset of genes that are missing in the assembly/group of interest, and then the summary ratings recalculated for that subset.

In each case, three plots are made (assuming both BUSCO and BUSCOMP data is available):

1. BUSCO ratings for missing BUSCO genes.

2. BUSCOMP ratings for missing BUSCO genes. As well as being more relaxed than pure BUSCO results, this will indicate when BUSCOMP has found a gene in the focal assembly/group where BUSCO did not.
3. BUSCOMP ratings for missing BUSCOMP genes. It is expected that assemblies will be much more similar in terms of BUSCOMP coverage.

5.4 Missing starling10x.dipnr BUSCO genes

BUSCO ratings for `Missing` starling10x.dipnr BUSCO genes:

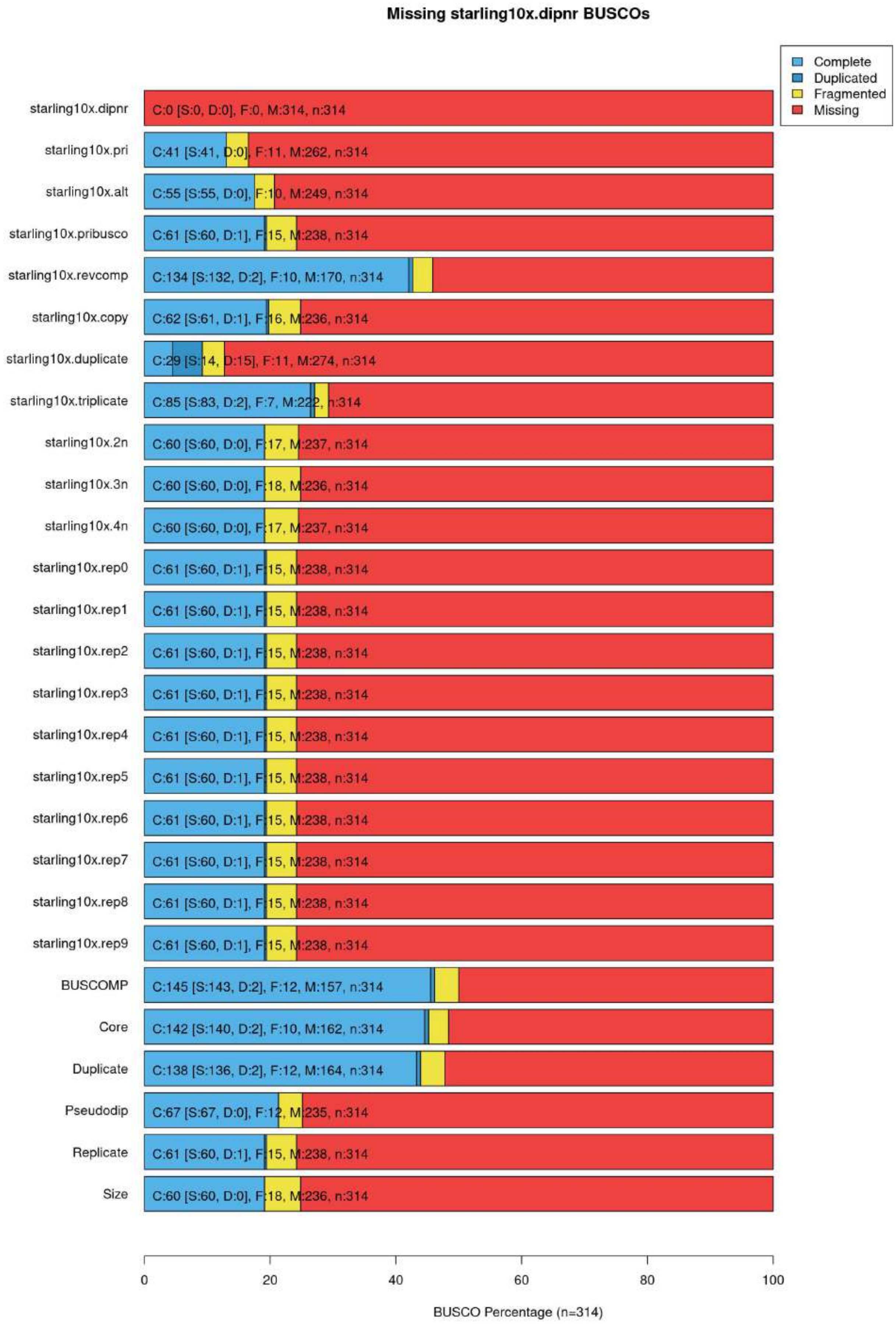

BUSCOMP ratings for `Missing` starling10x.dipnr BUSCO genes:

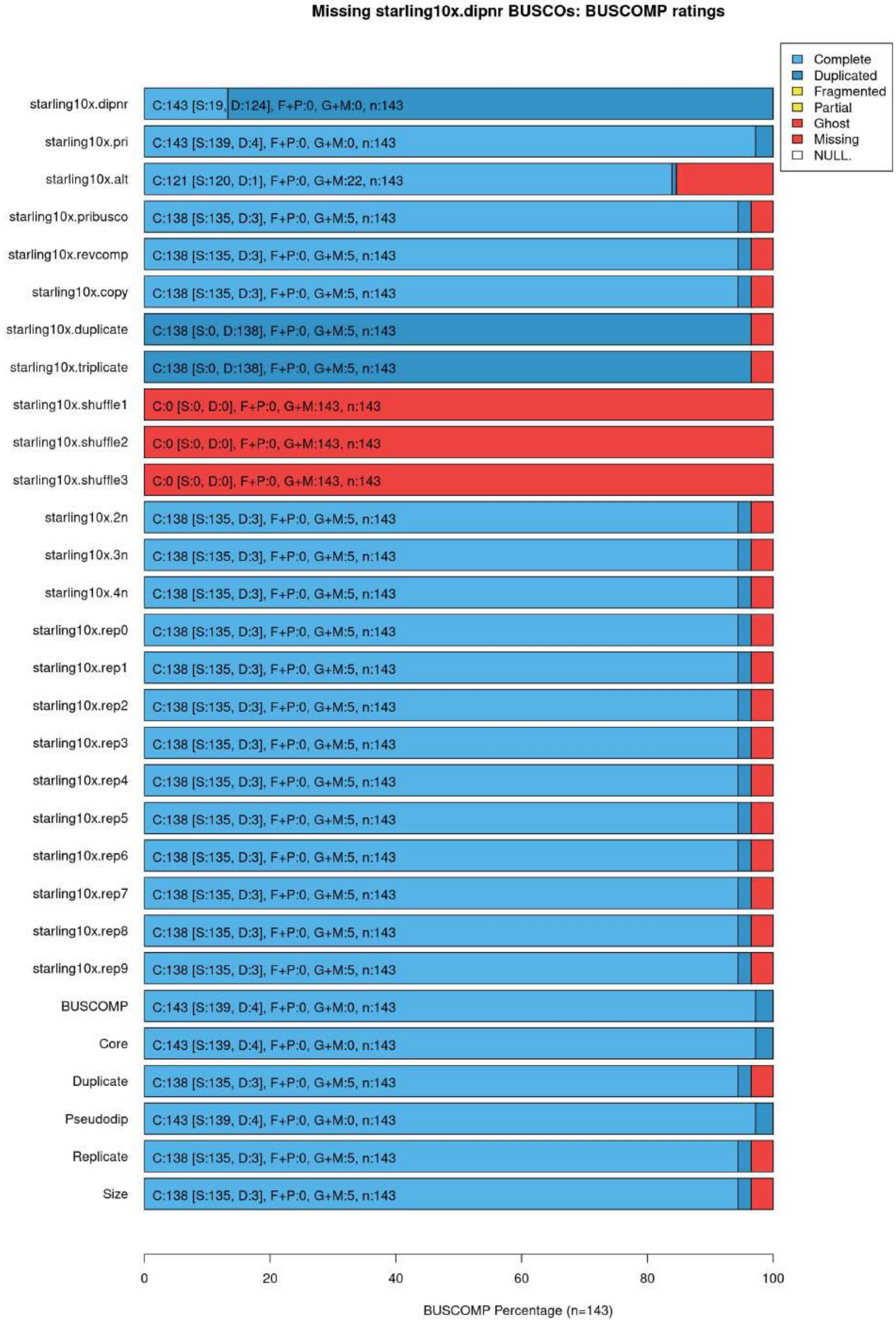

BUSCOMP ratings for `Missing` starling10x.dipnr BUSCOMP genes:

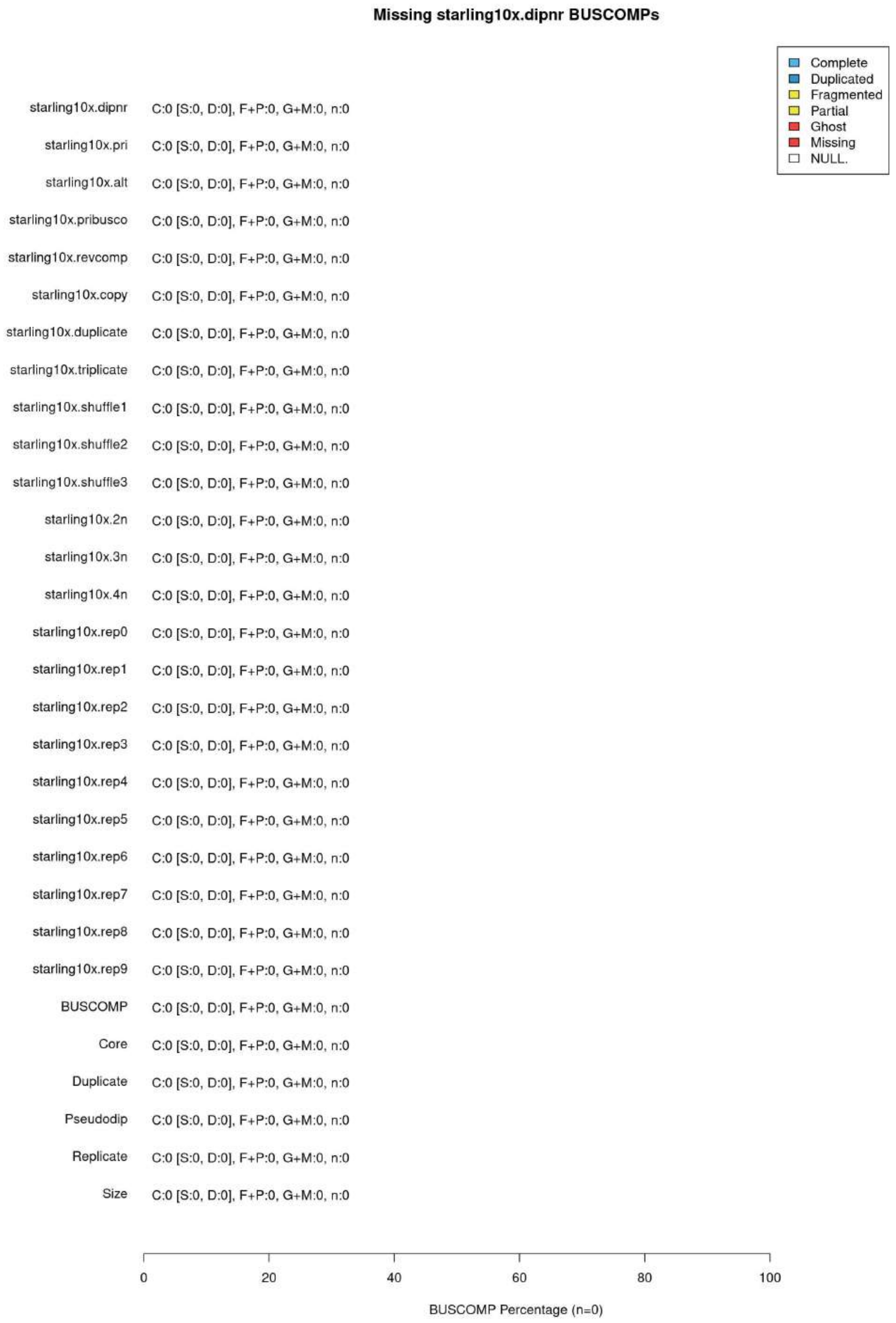

5.5 Missing starling10x.pri BUSCO genes

BUSCO ratings for `Missing` starling10x.pri BUSCO genes:

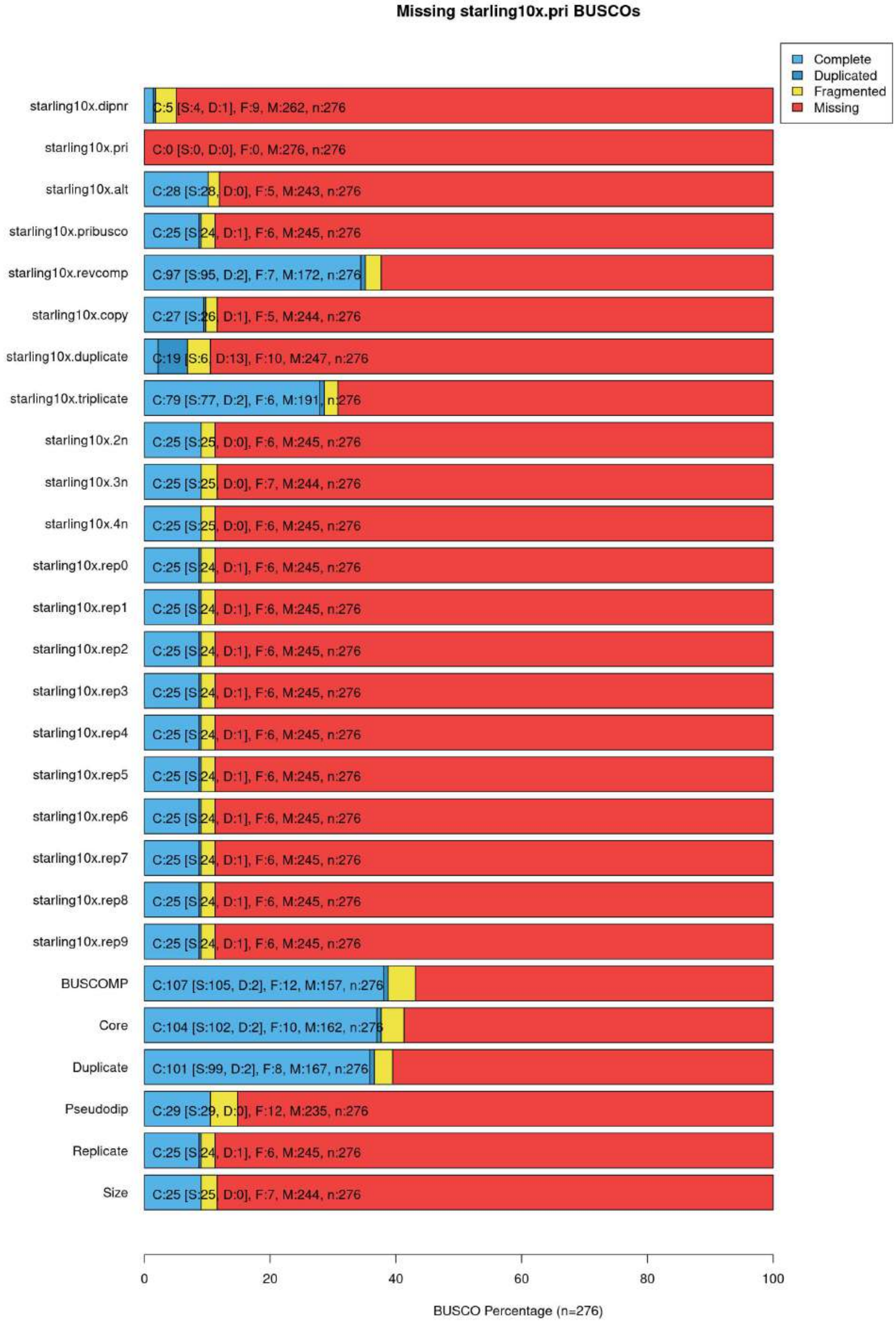

BUSCOMP ratings for `Missing` starling10x.pri BUSCO genes:

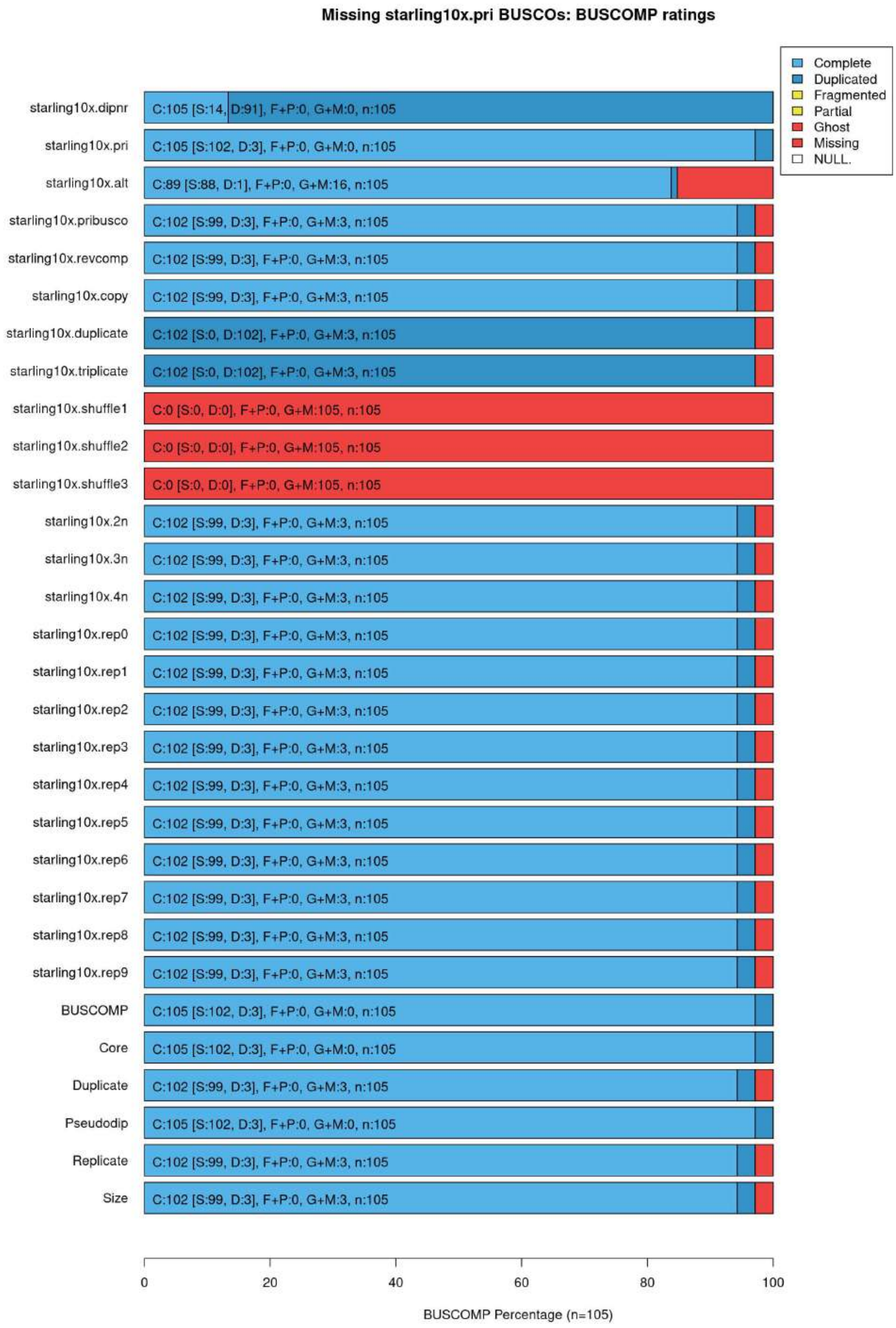

BUSCOMP ratings for `Missing` starling10x.pri BUSCOMP genes:

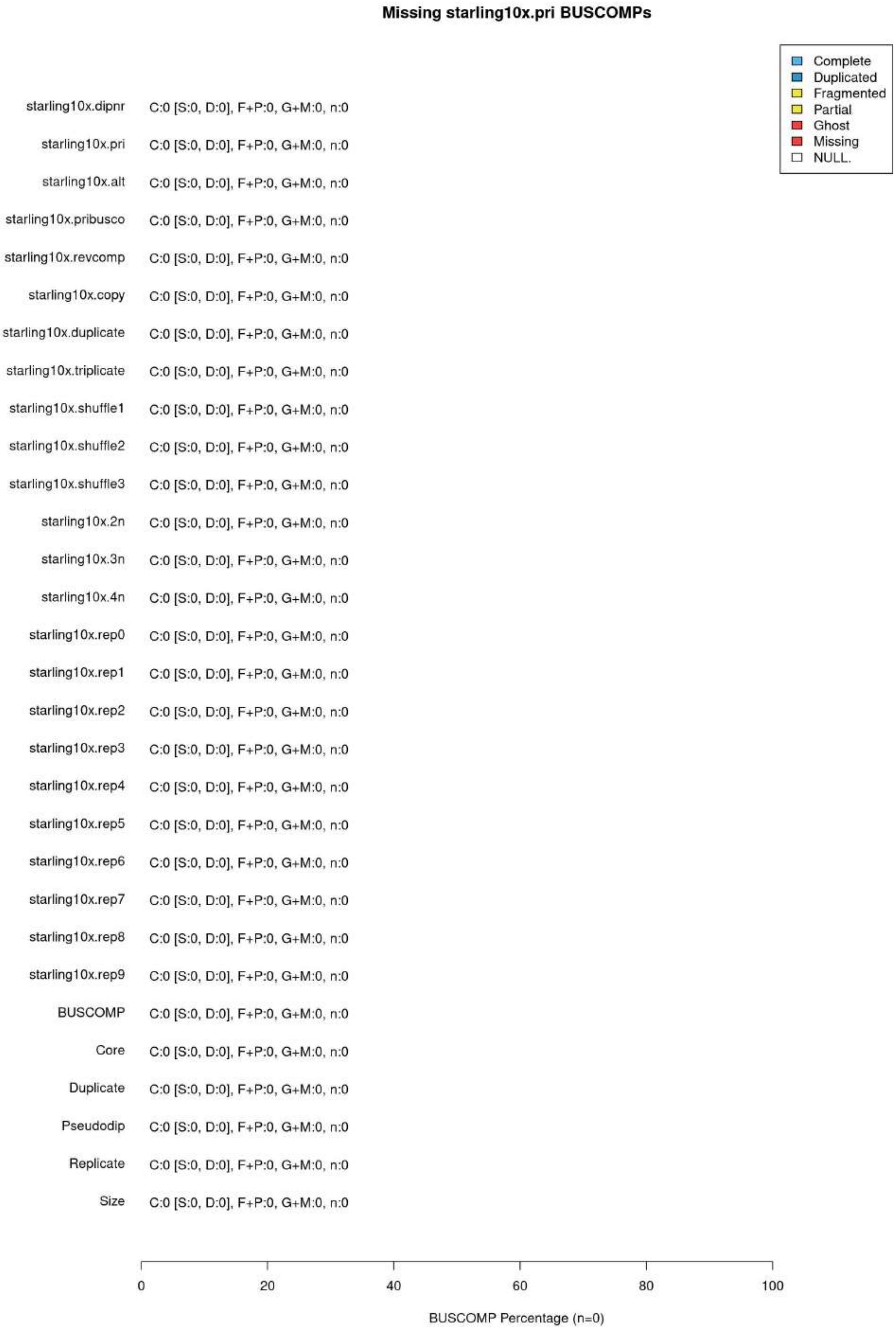

5.6 Missing starling10x.alt BUSCO genes

BUSCO ratings for `Missing` starling10x.alt BUSCO genes:

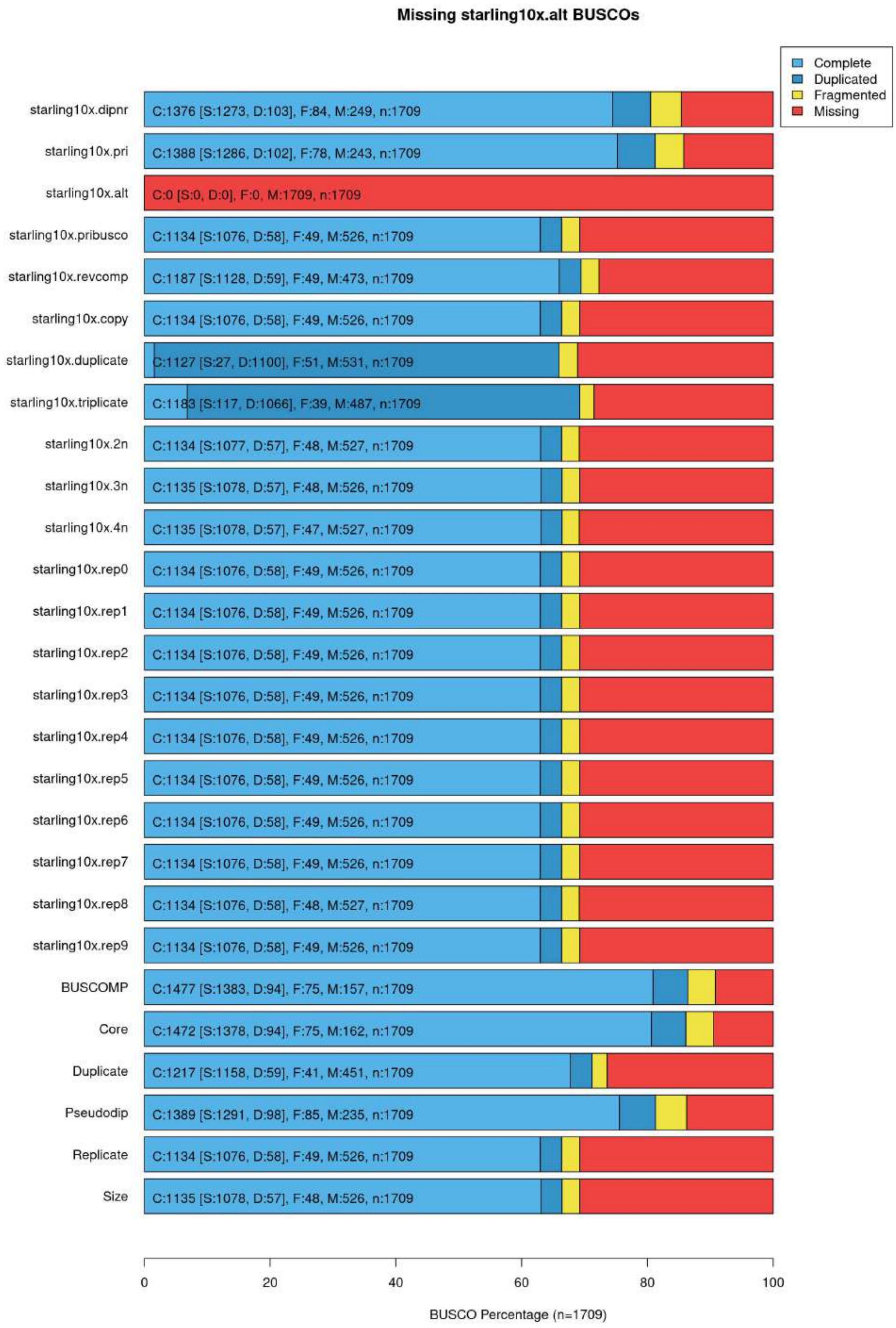

BUSCOMP ratings for `Missing` starling10x.alt BUSCO genes:

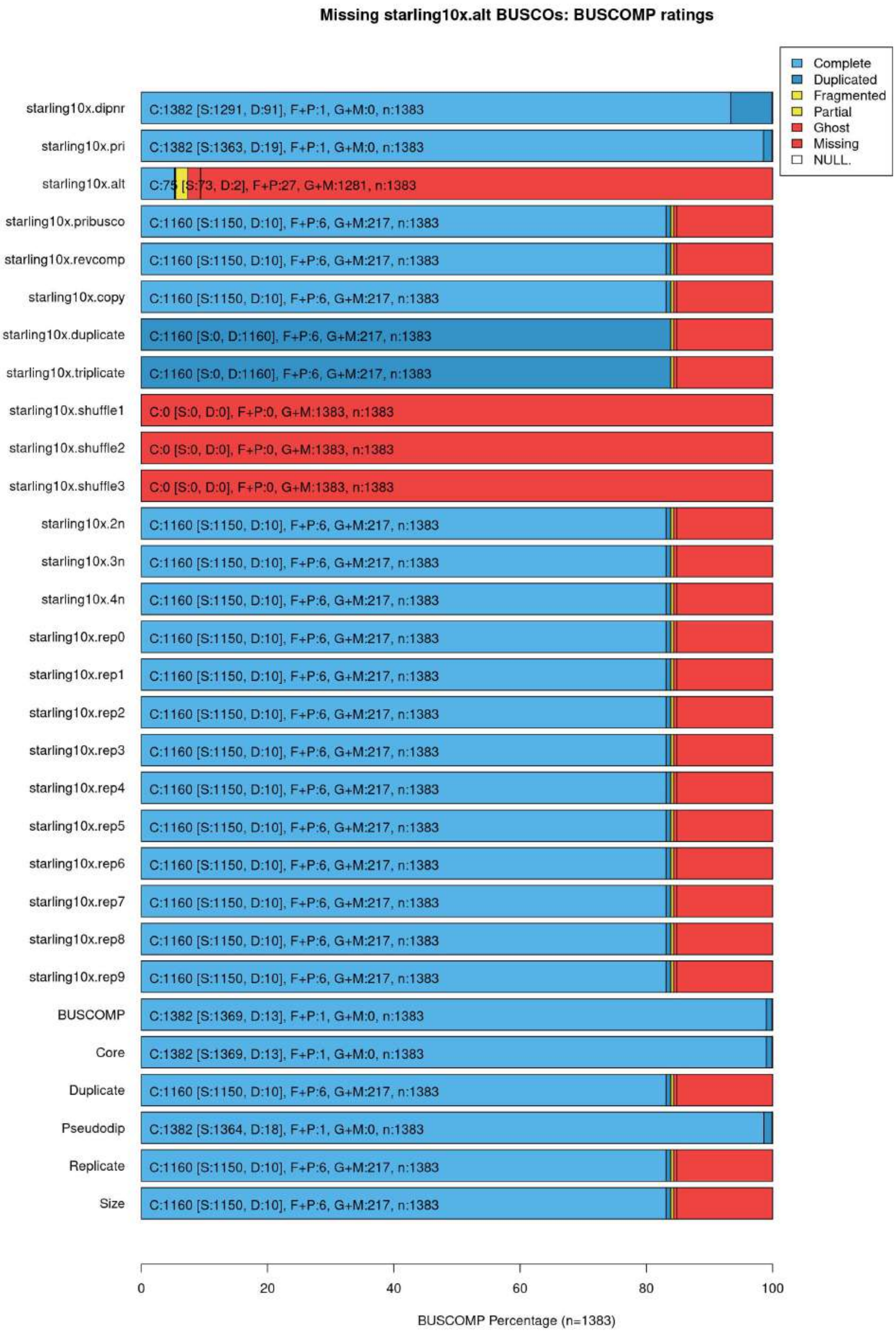

BUSCOMP ratings for `Missing` starling10x.alt BUSCOMP genes:

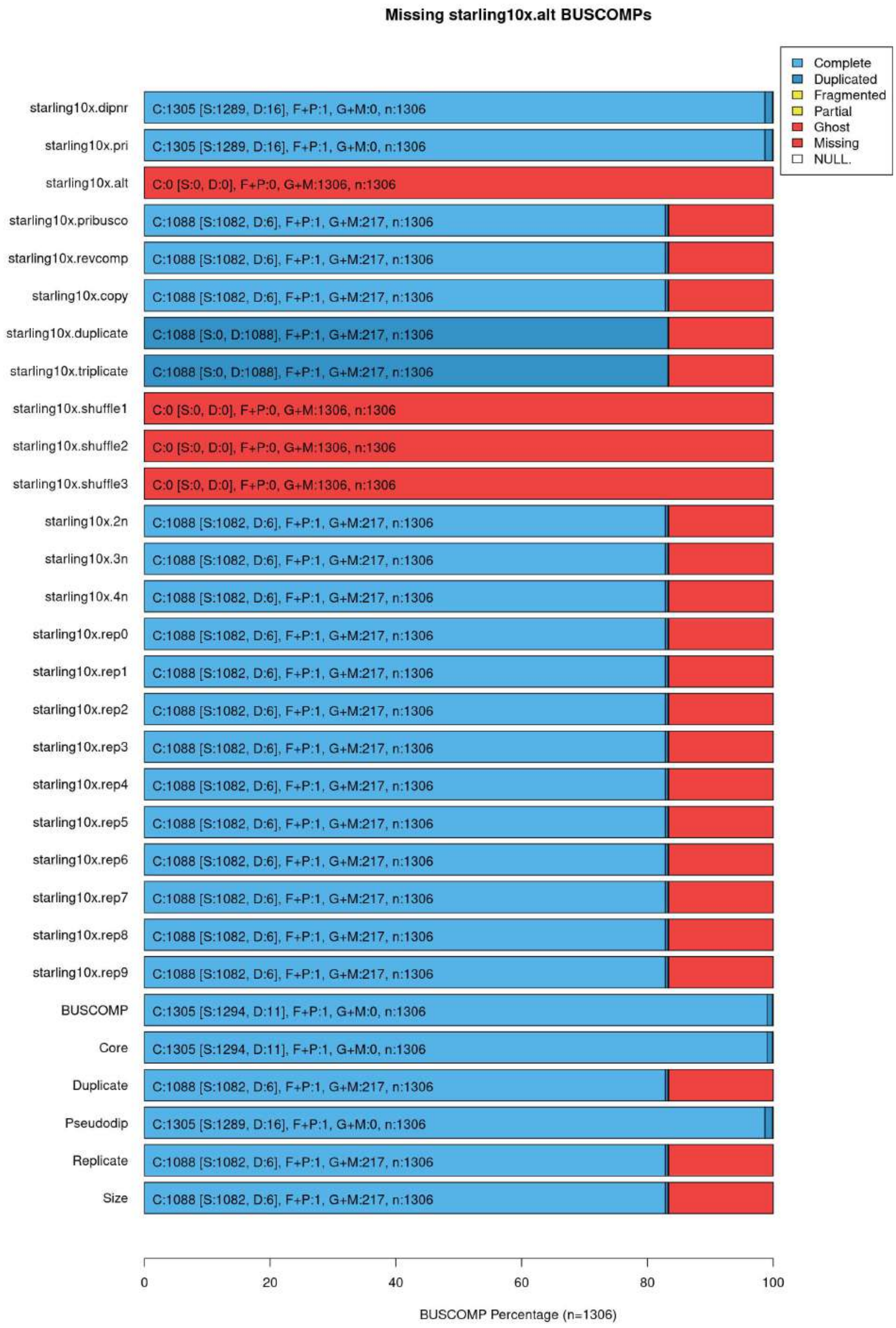

5.7 Missing Pseudodip BUSCO genes

BUSCO ratings for `Missing` Pseudodip BUSCO genes:

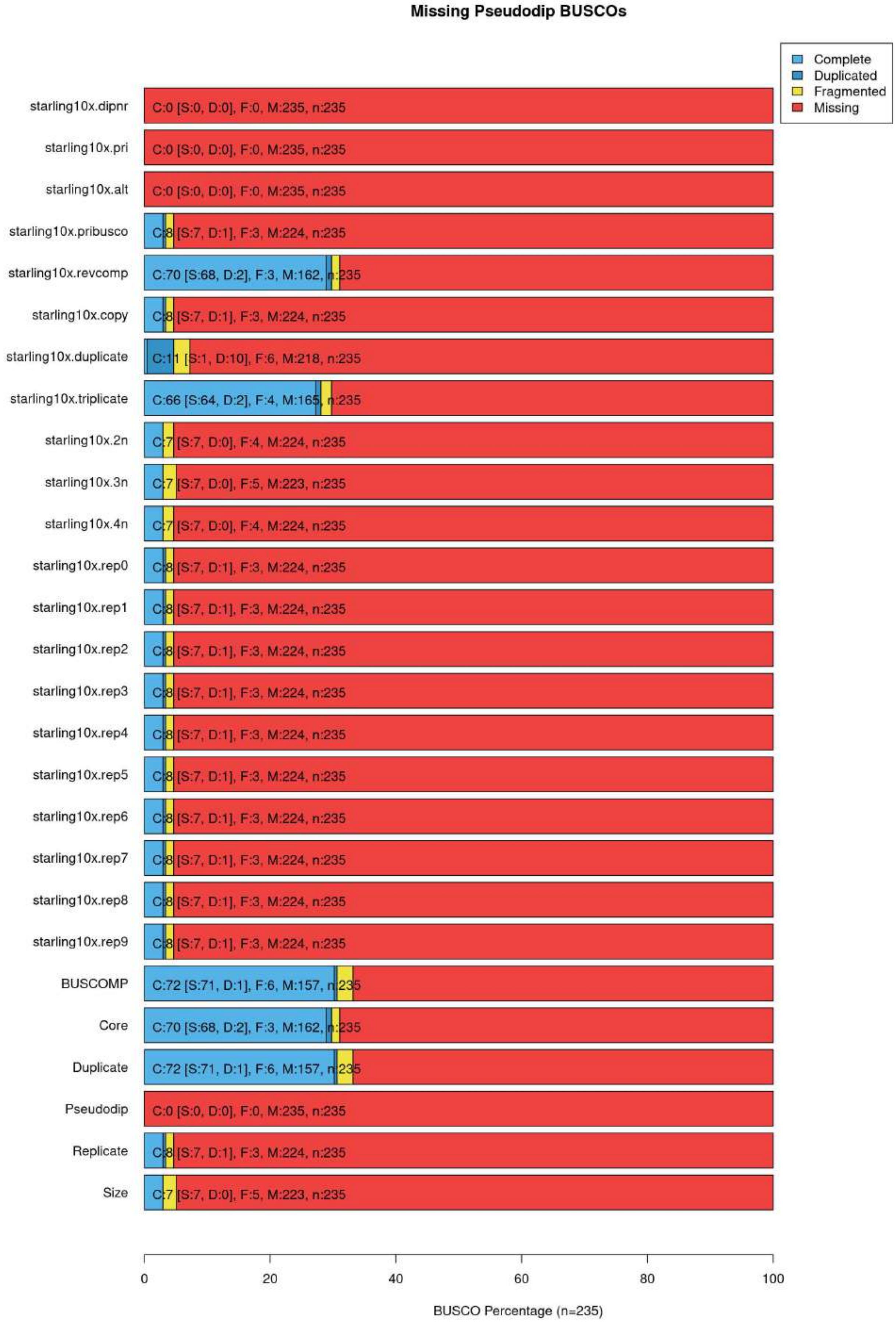

BUSCOMP ratings for `Missing` Pseudodip BUSCO genes:

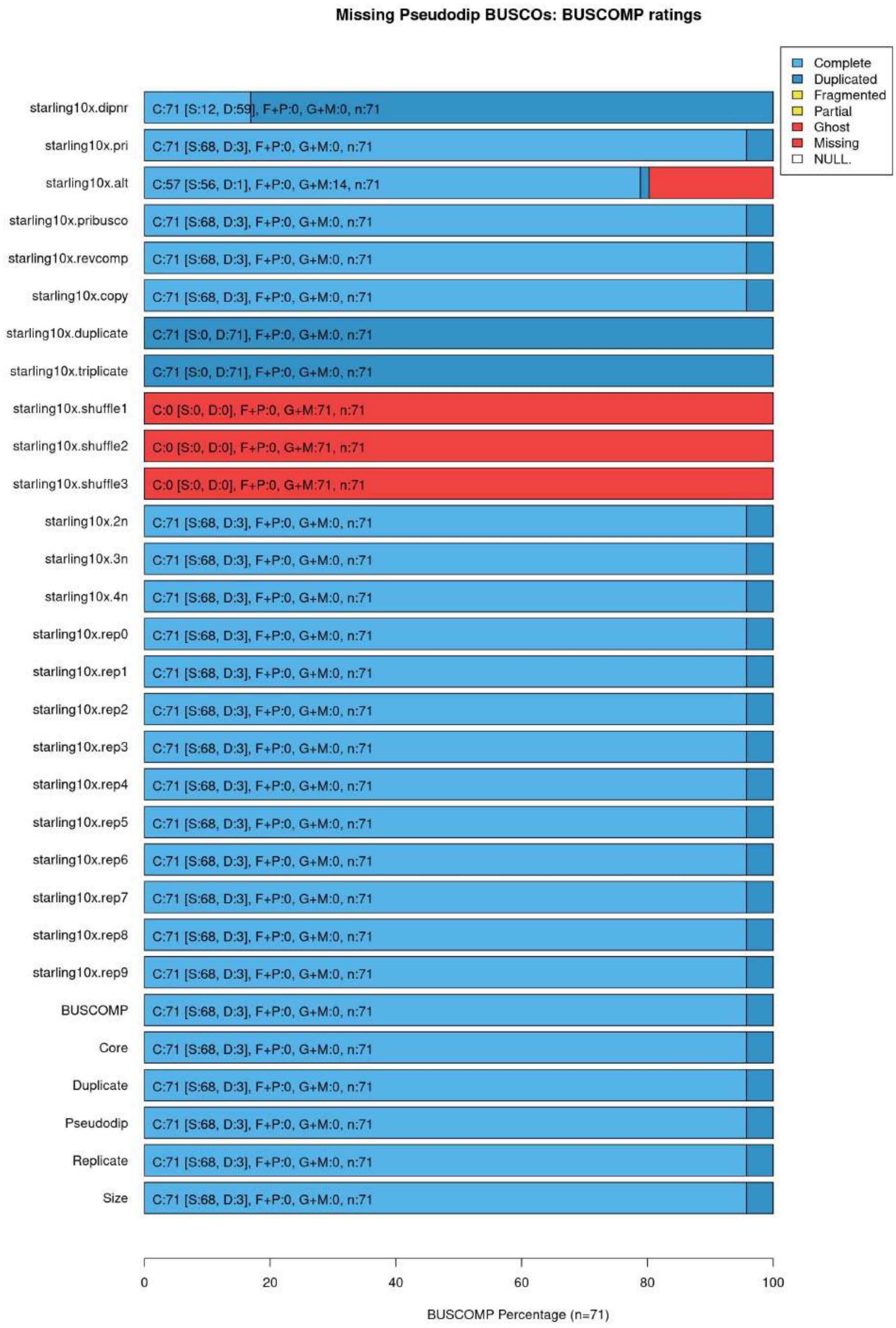

BUSCOMP ratings for `Missing` Pseudodip BUSCOMP genes:

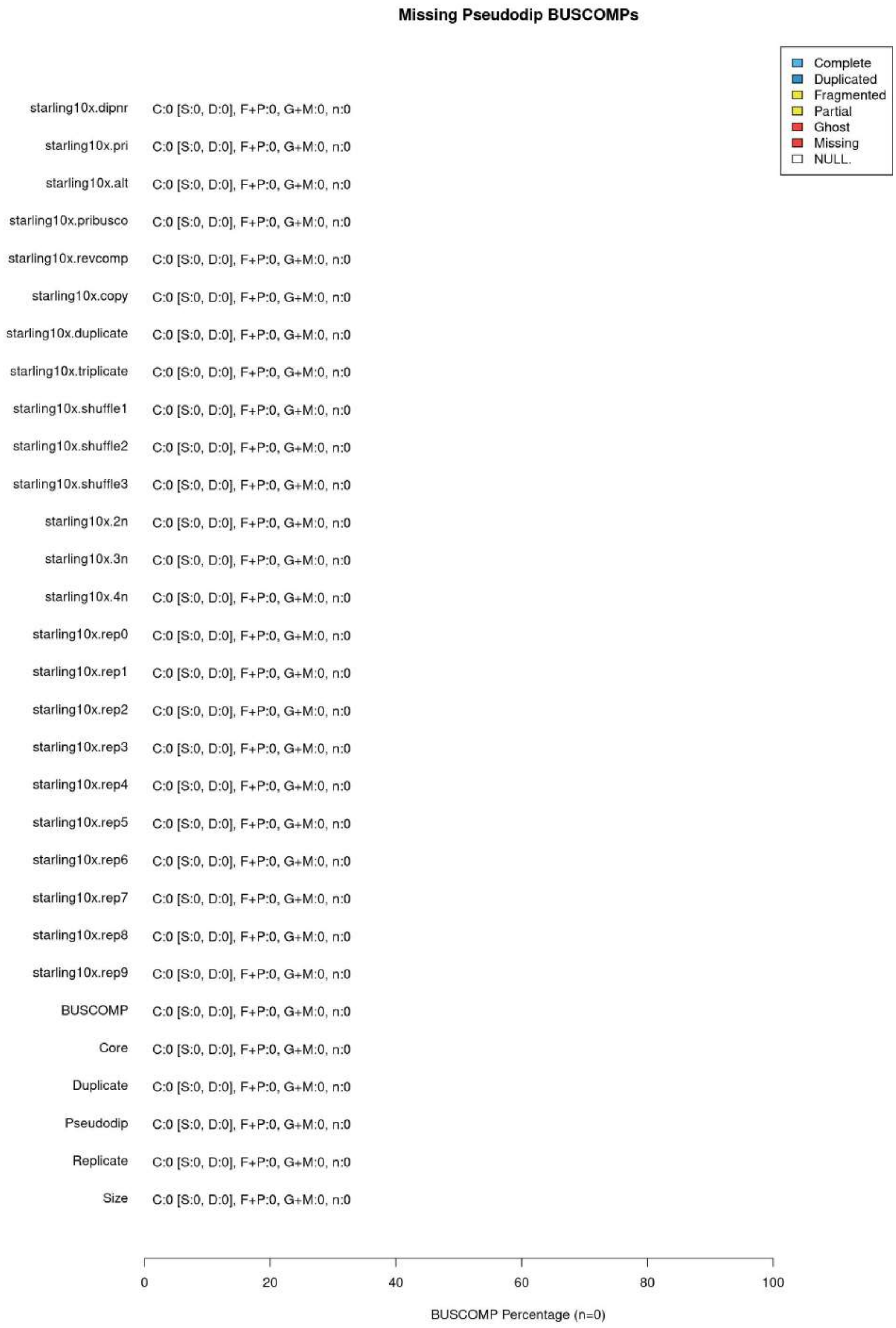

5.8 Missing starling10x.pribusco BUSCO genes

BUSCO ratings for `Missing` starling10x.pribusco BUSCO genes:

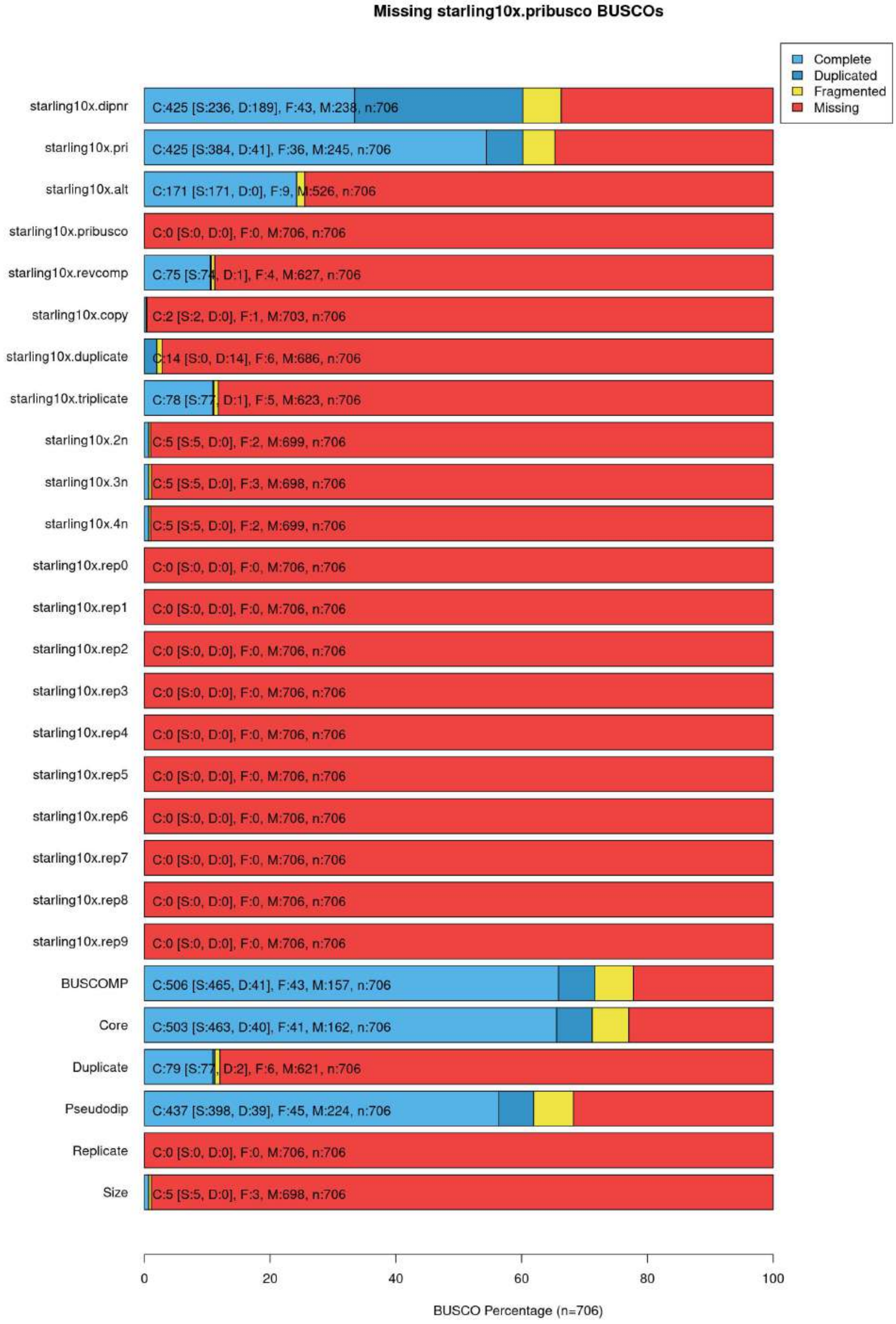

BUSCOMP ratings for `Missing` starling10x.pribusco BUSCO genes:

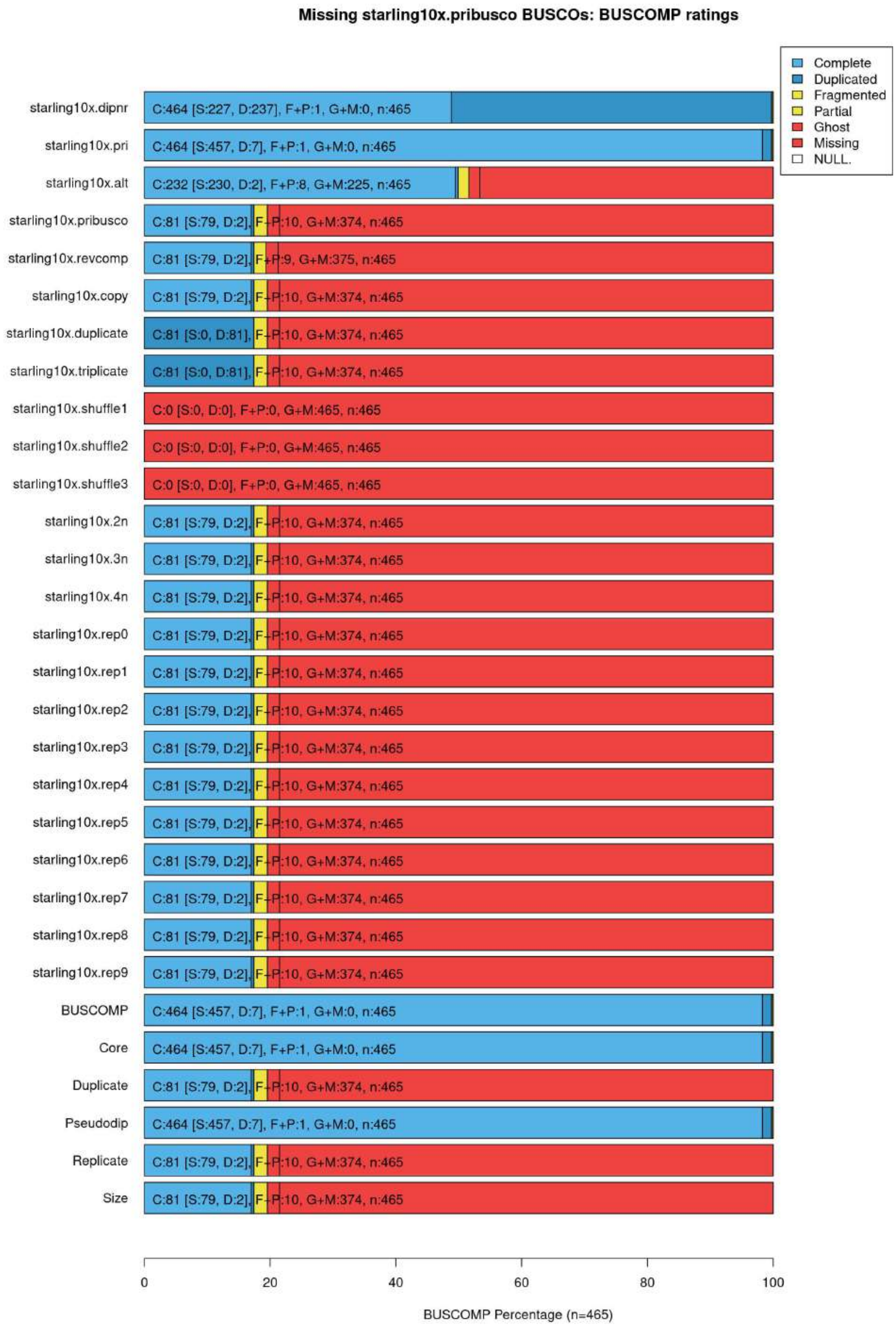

BUSCOMP ratings for `Missing` starling10x.pribusco BUSCOMP genes:

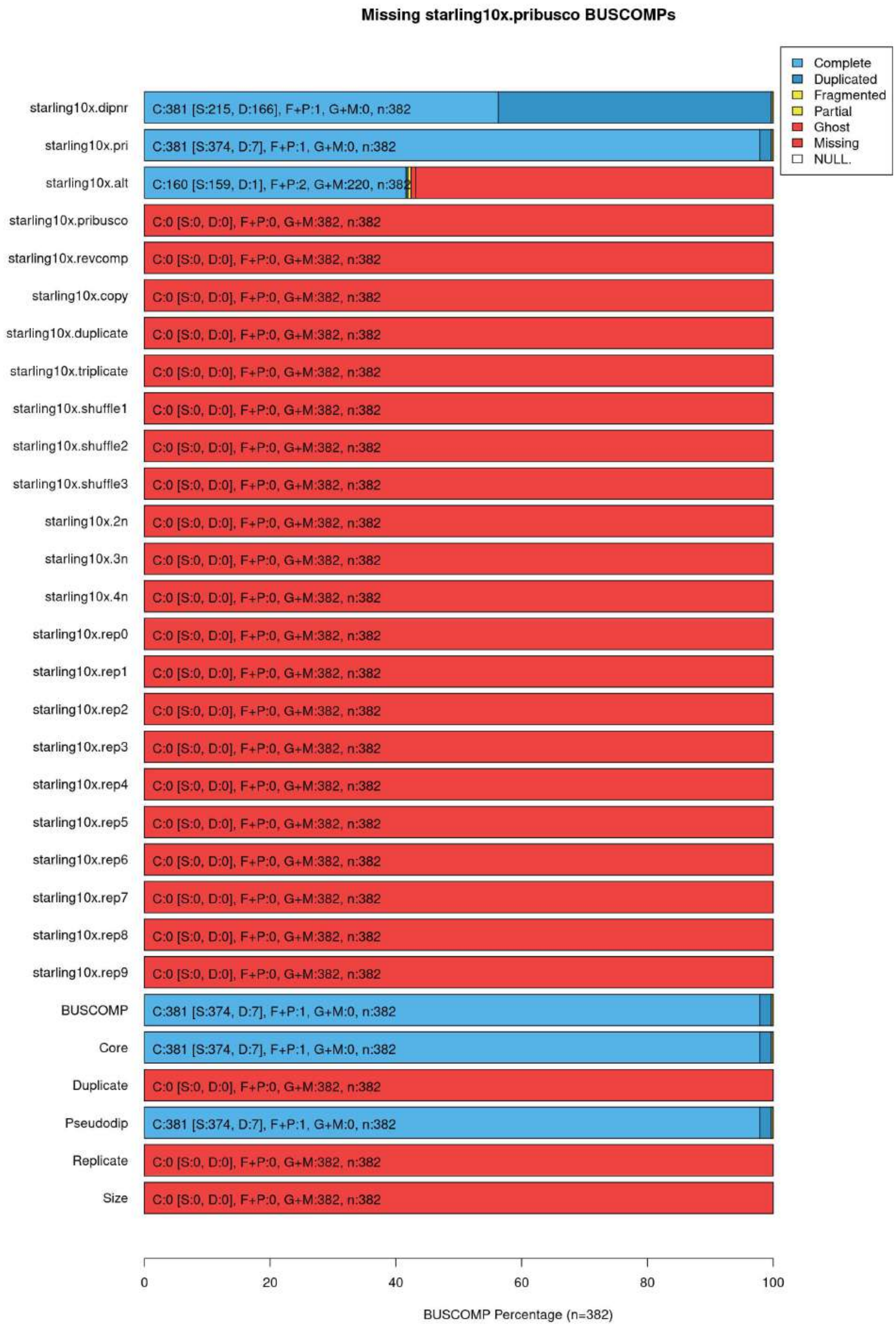

5.9 Missing starling10x.revcomp BUSCO genes

BUSCO ratings for `Missing` starling10x.revcomp BUSCO genes:

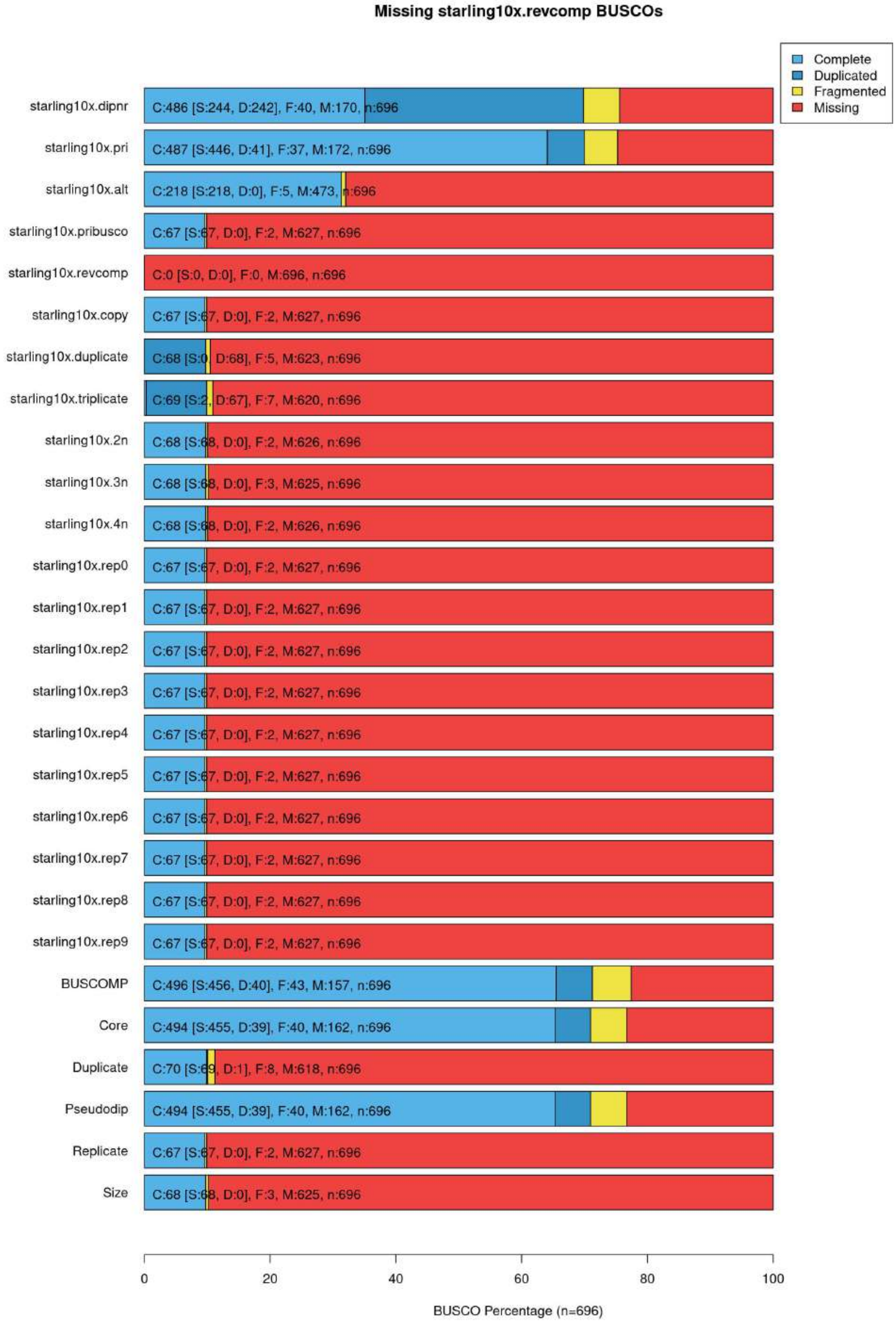

BUSCOMP ratings for `Missing` starling10x.revcomp BUSCO genes:

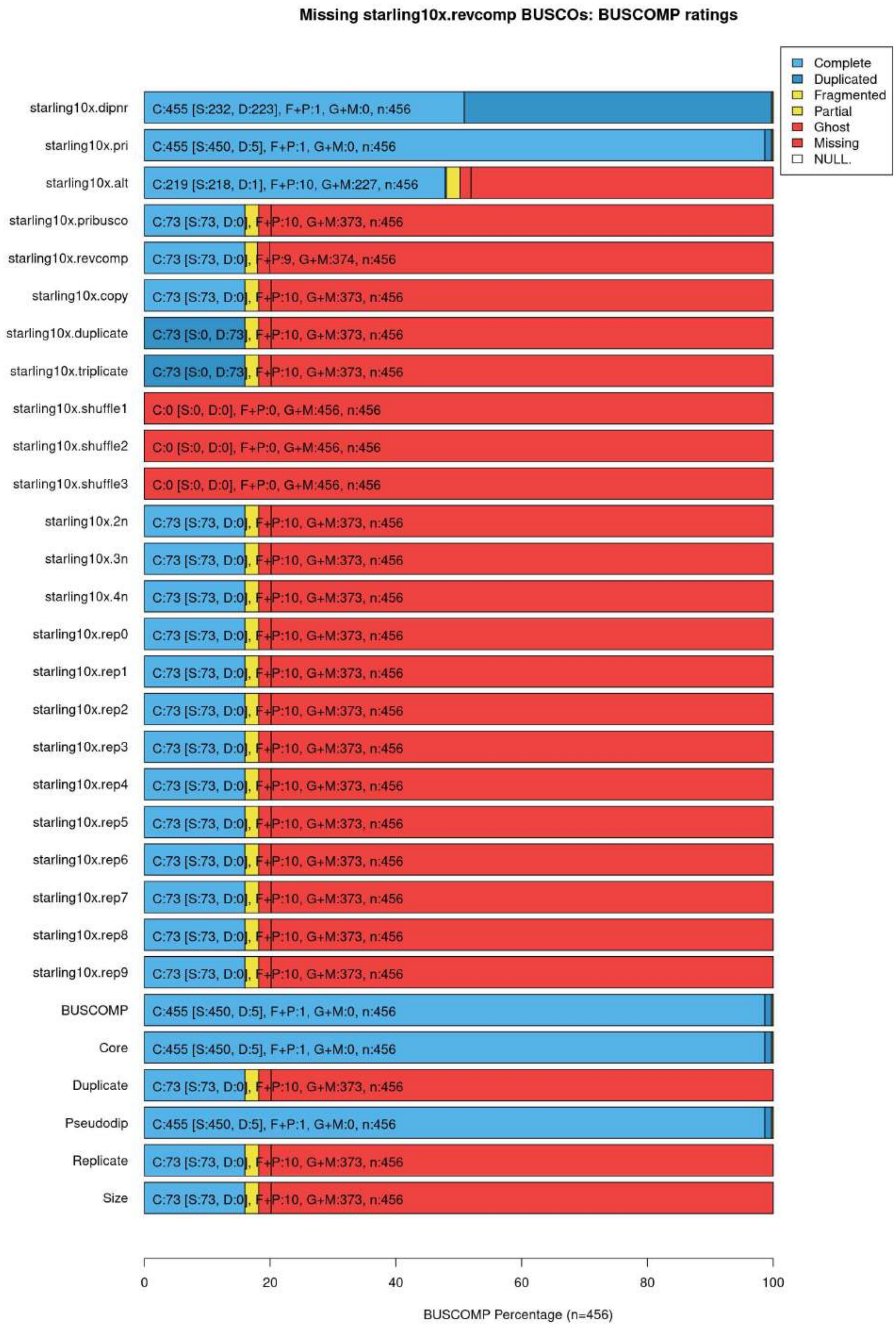

BUSCOMP ratings for `Missing` starling10x.revcomp BUSCOMP genes:

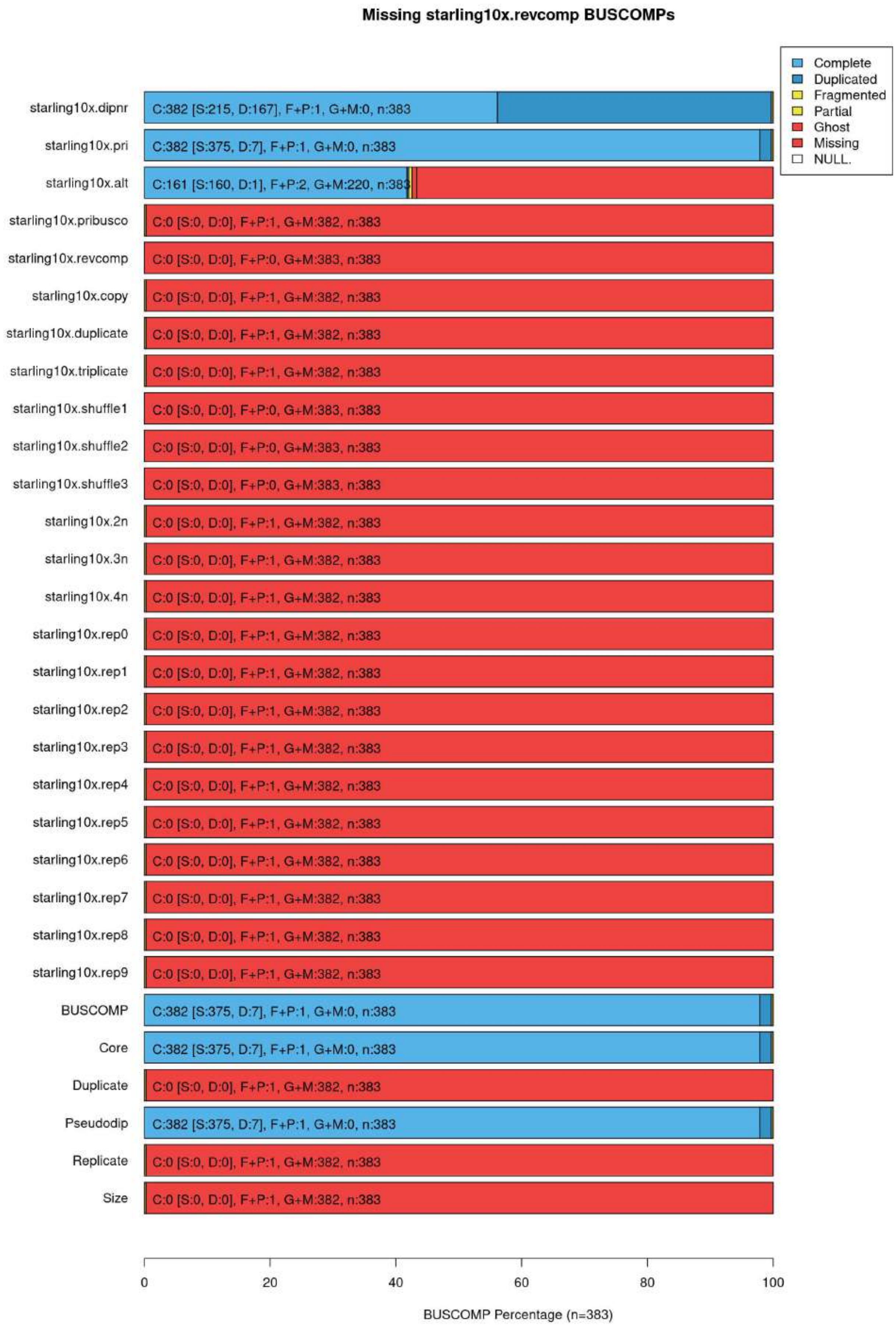

5.10 Missing Core BUSCO genes

BUSCO ratings for `Missing` Core BUSCO genes:

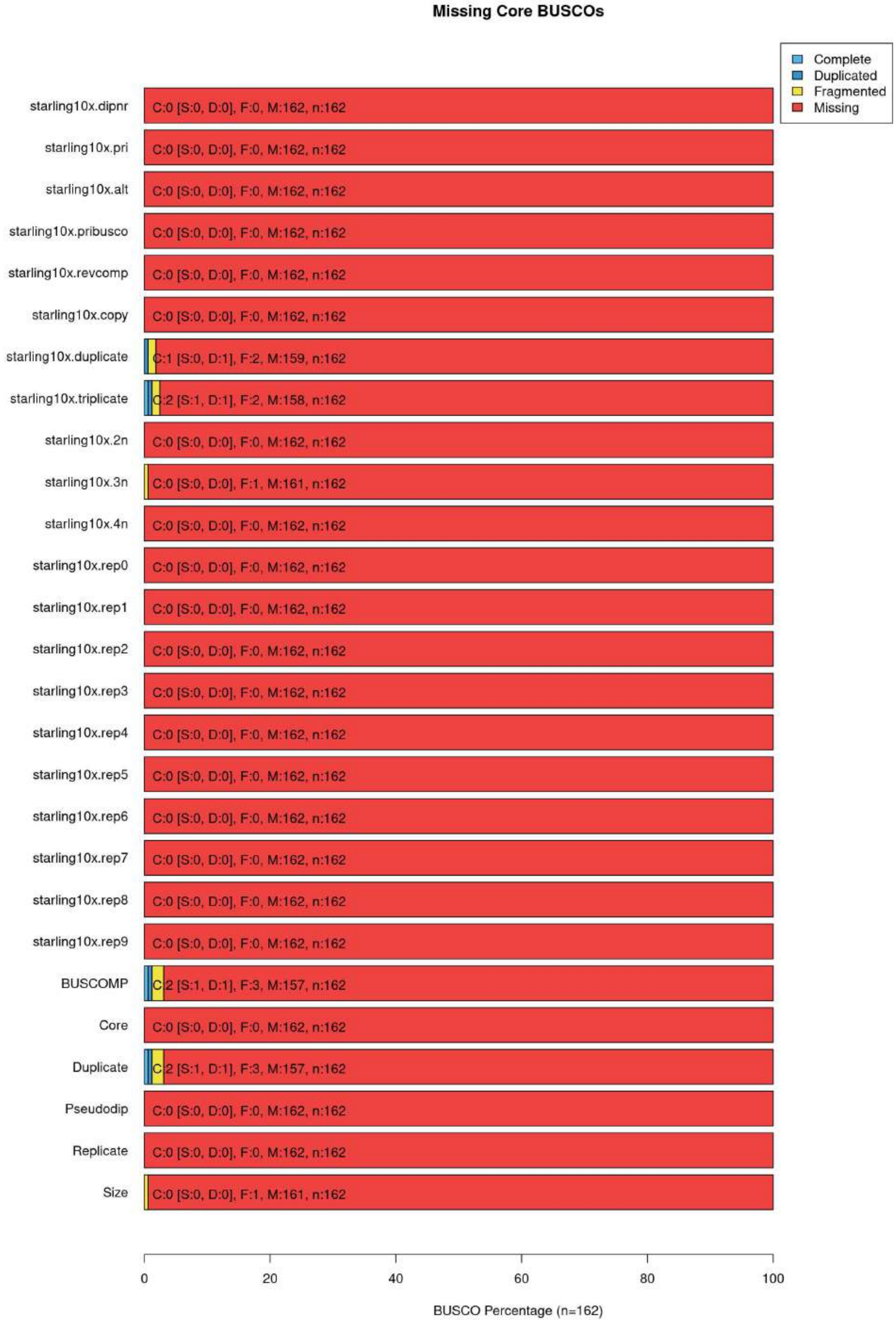

BUSCOMP ratings for `Missing` Core BUSCO genes:

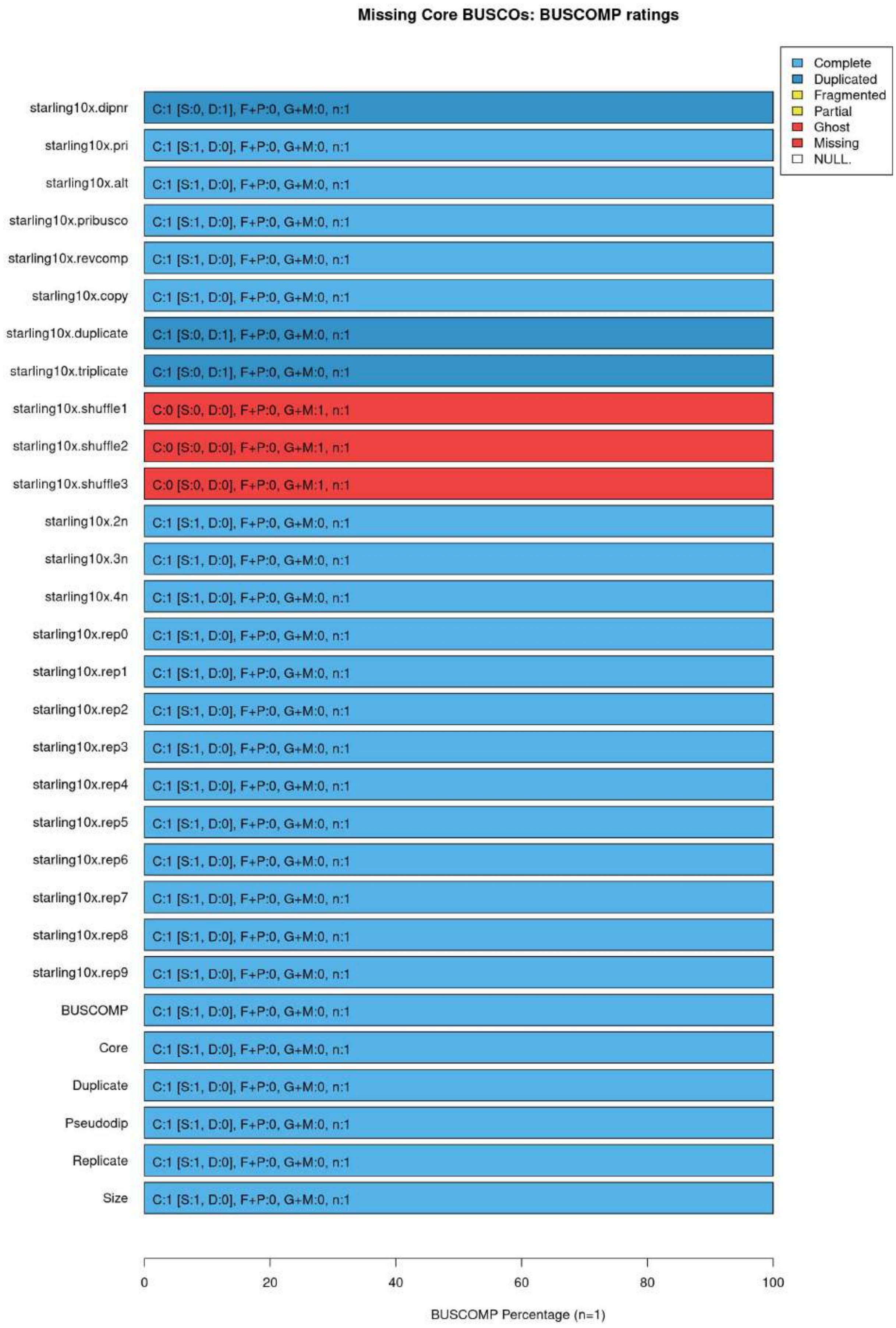

BUSCOMP ratings for `Missing` Core BUSCOMP genes:

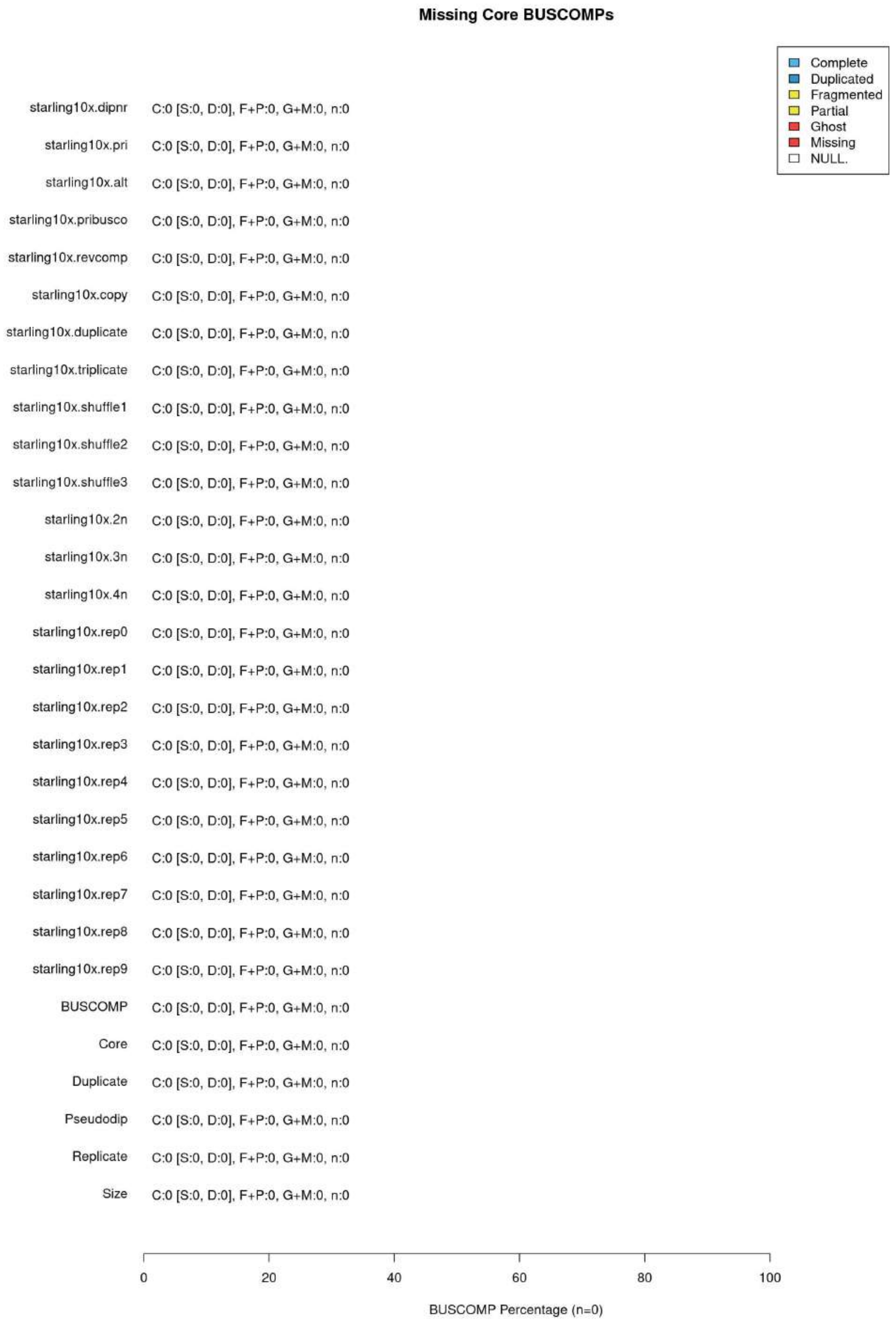

5.11 Missing starling10x.copy BUSCO genes

BUSCO ratings for `Missing` starling10x.copy BUSCO genes:

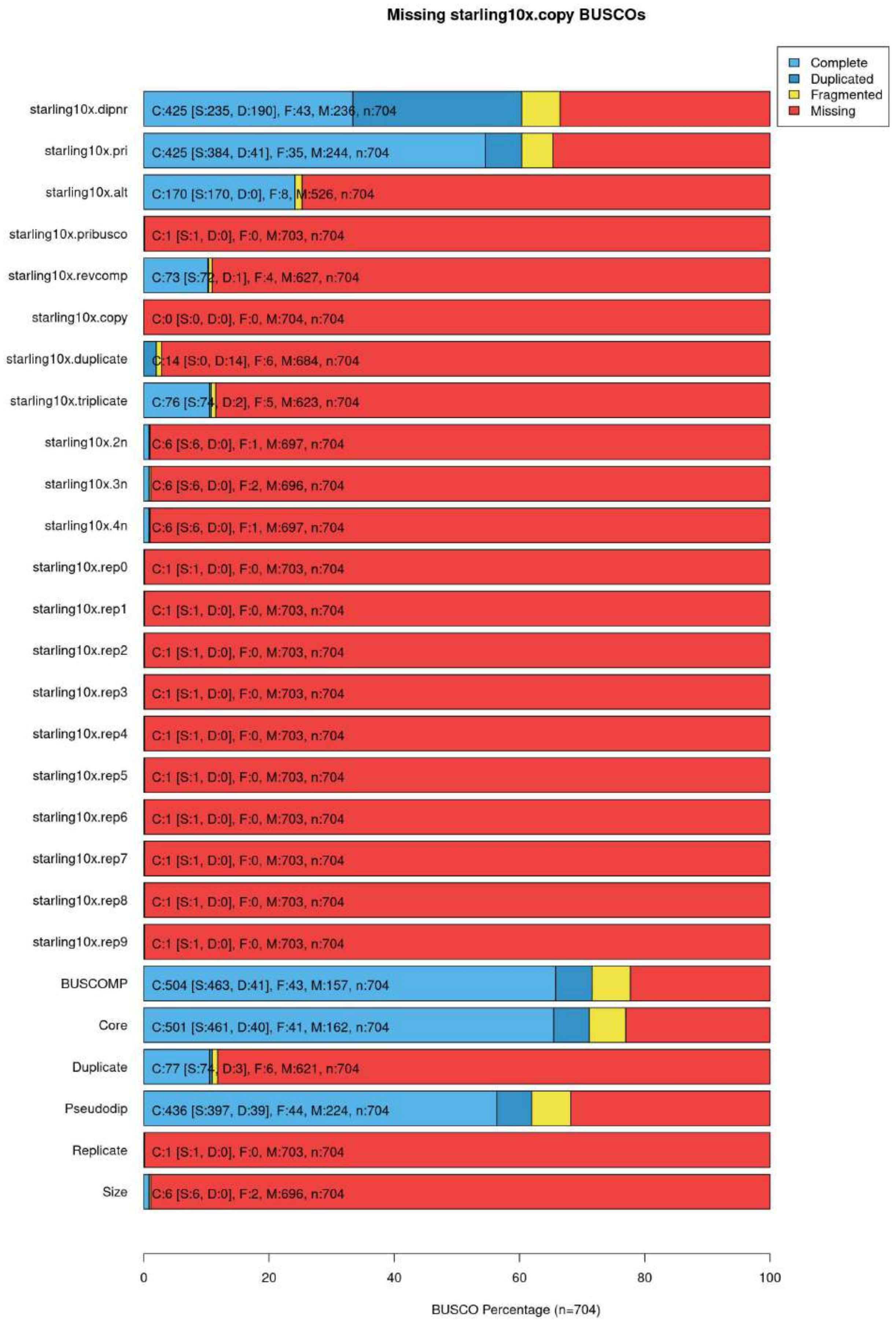

BUSCOMP ratings for `Missing` starling10x.copy BUSCO genes:

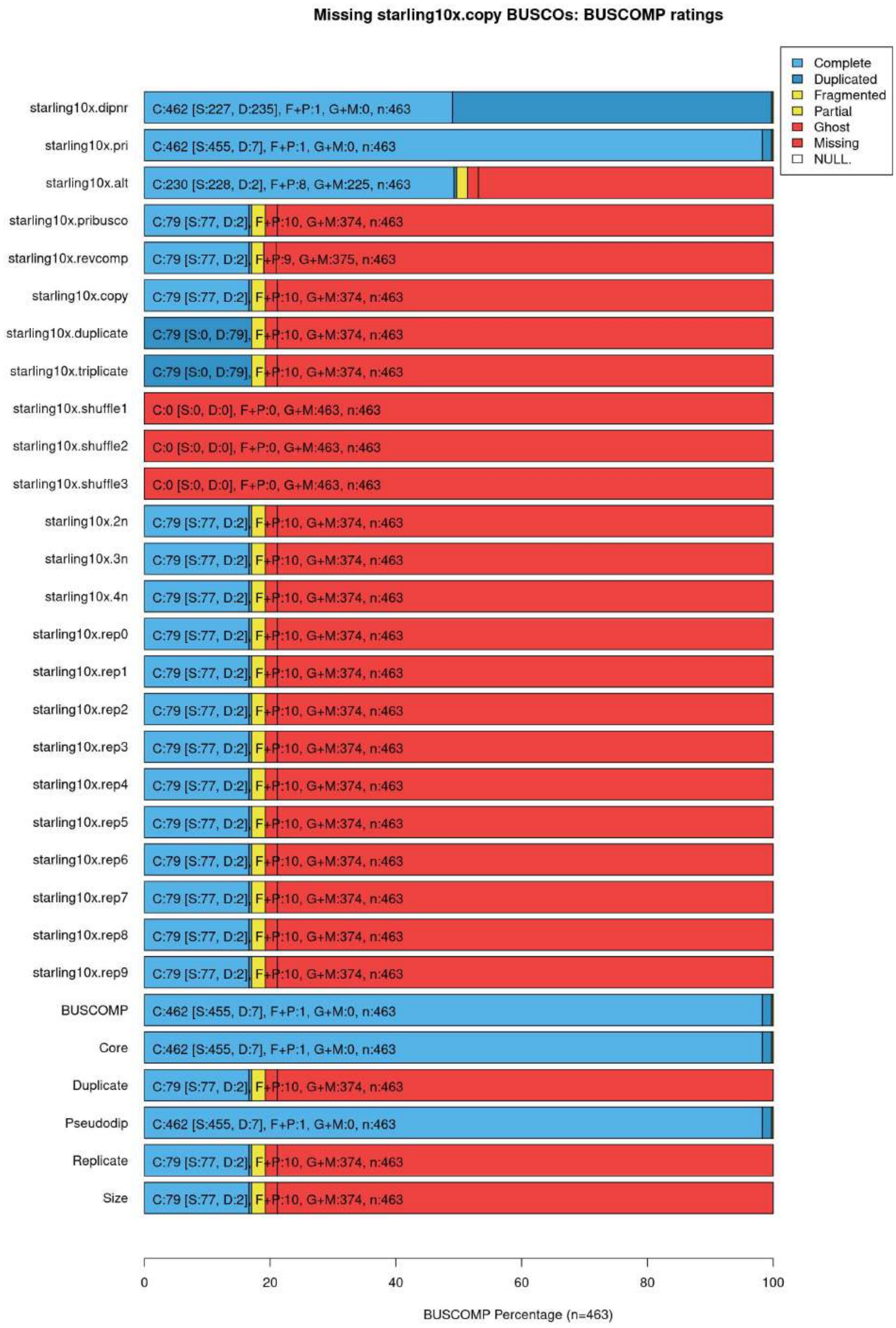

BUSCOMP ratings for `Missing` starling10x.copy BUSCOMP genes:

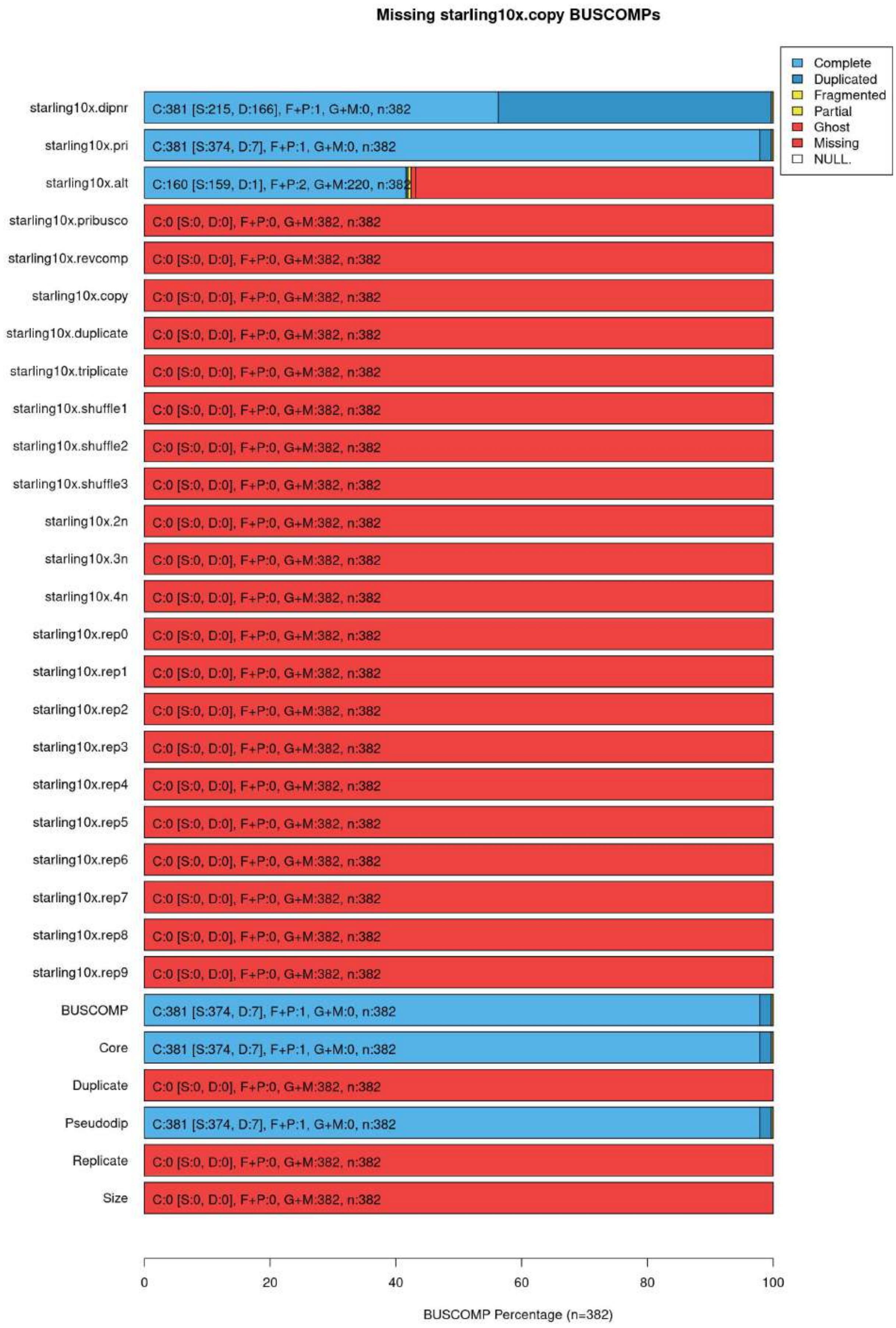

5.12 Missing starling10x.duplicate BUSCO genes

BUSCO ratings for `Missing` starling10x.duplicate BUSCO genes:

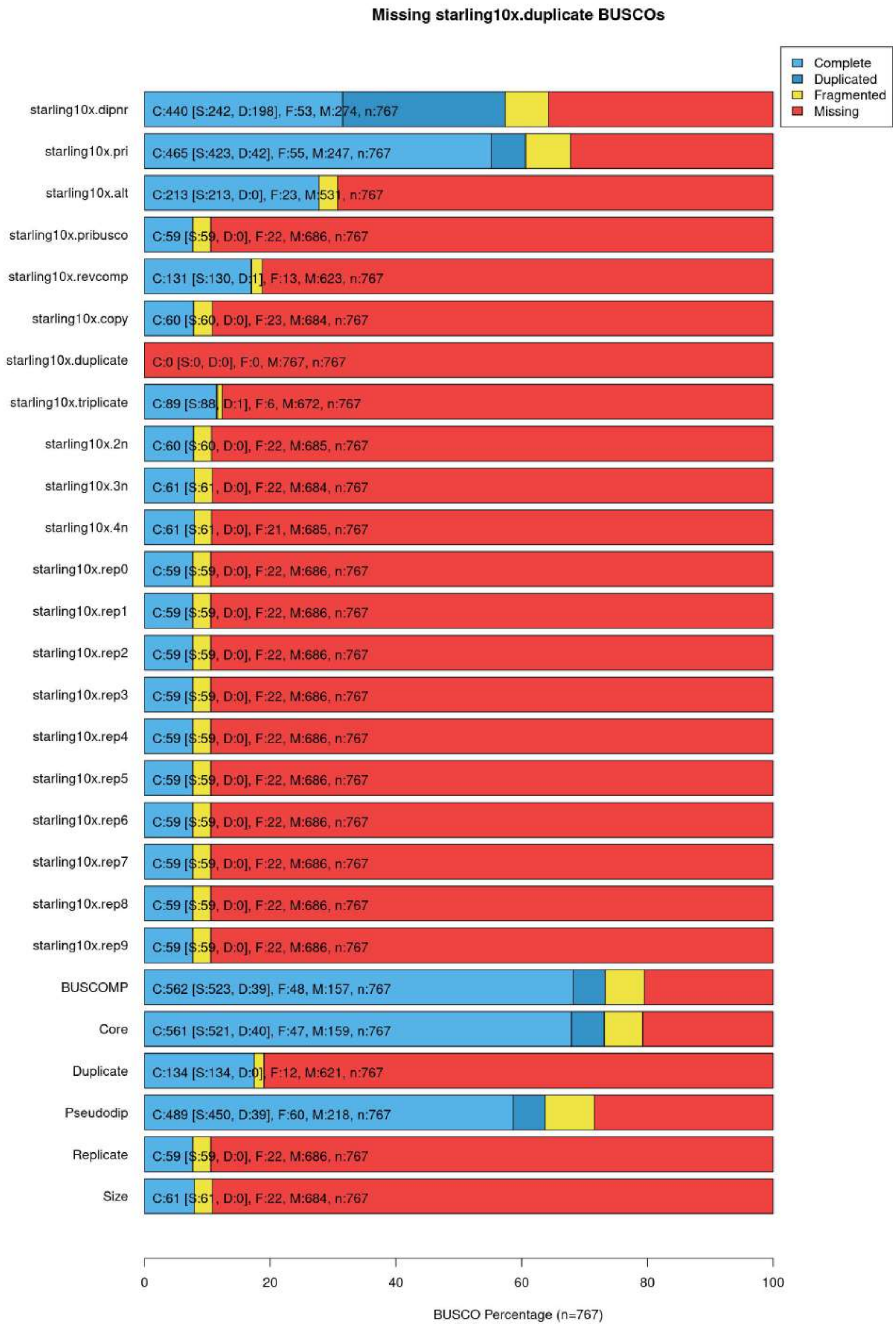

BUSCOMP ratings for `Missing` starling10x.duplicate BUSCO genes:

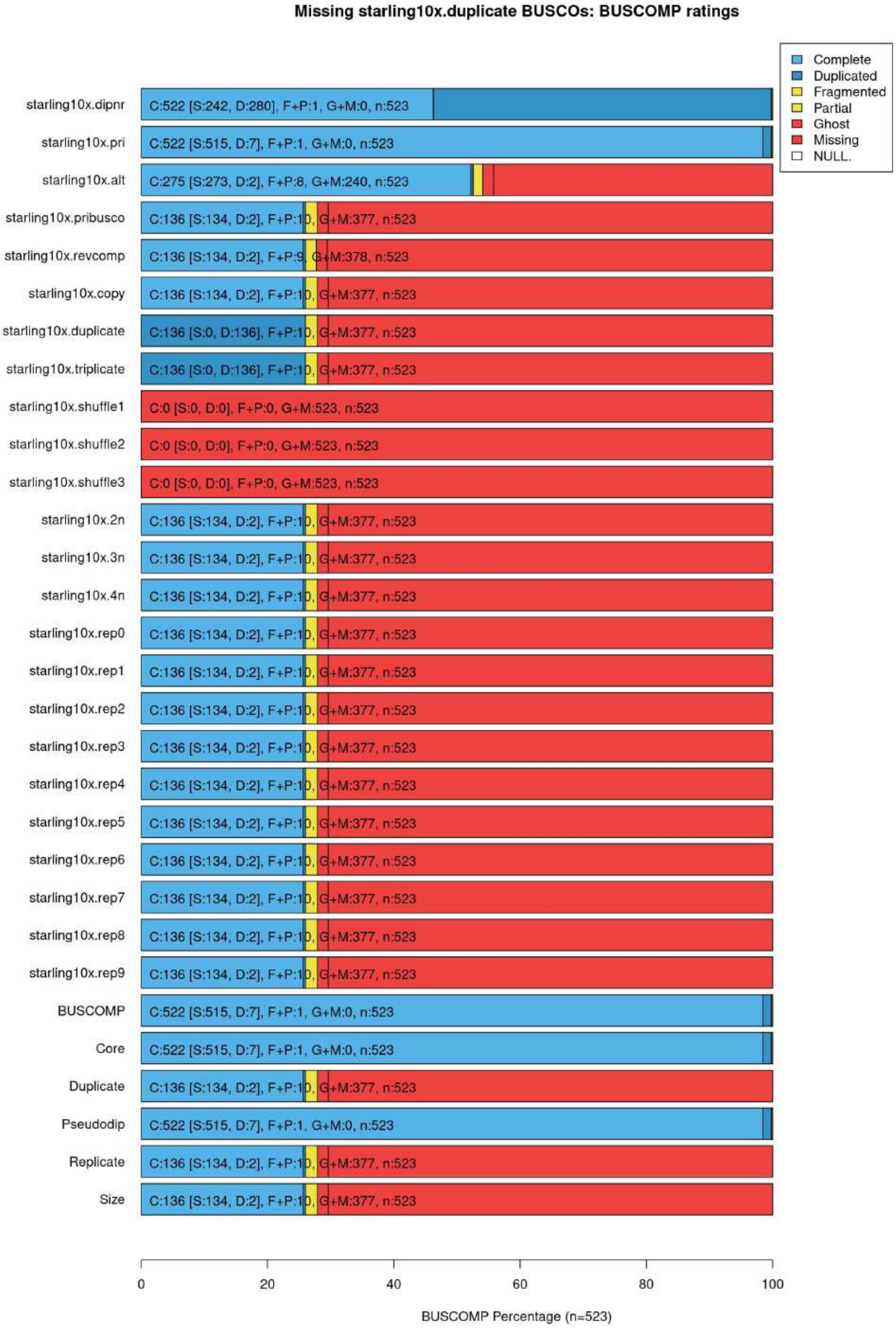

BUSCOMP ratings for `Missing` starling10x.duplicate BUSCOMP genes:

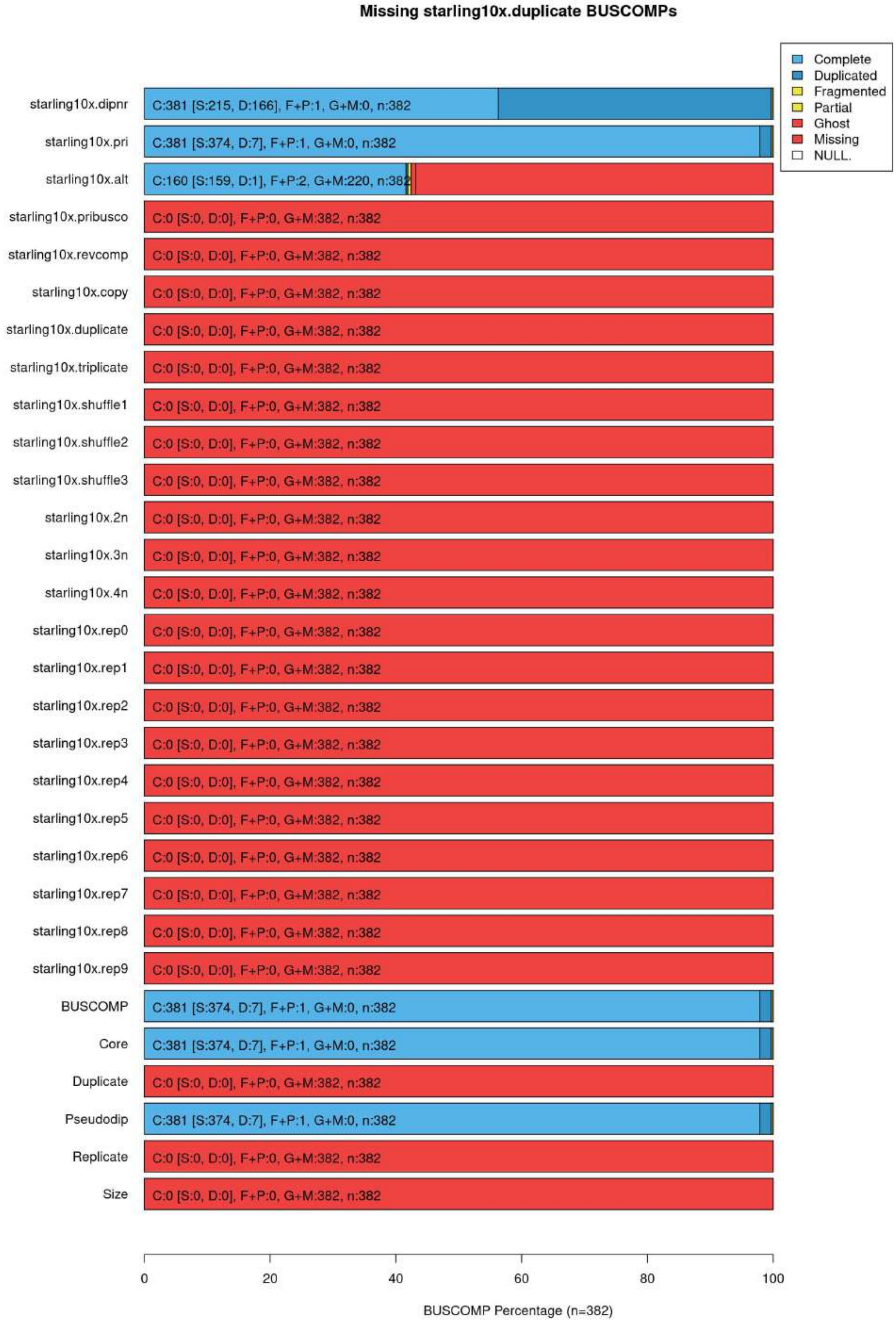

5.13 Missing starling10x.triplicate BUSCO genes

BUSCO ratings for `Missing` starling10x.triplicate BUSCO genes:

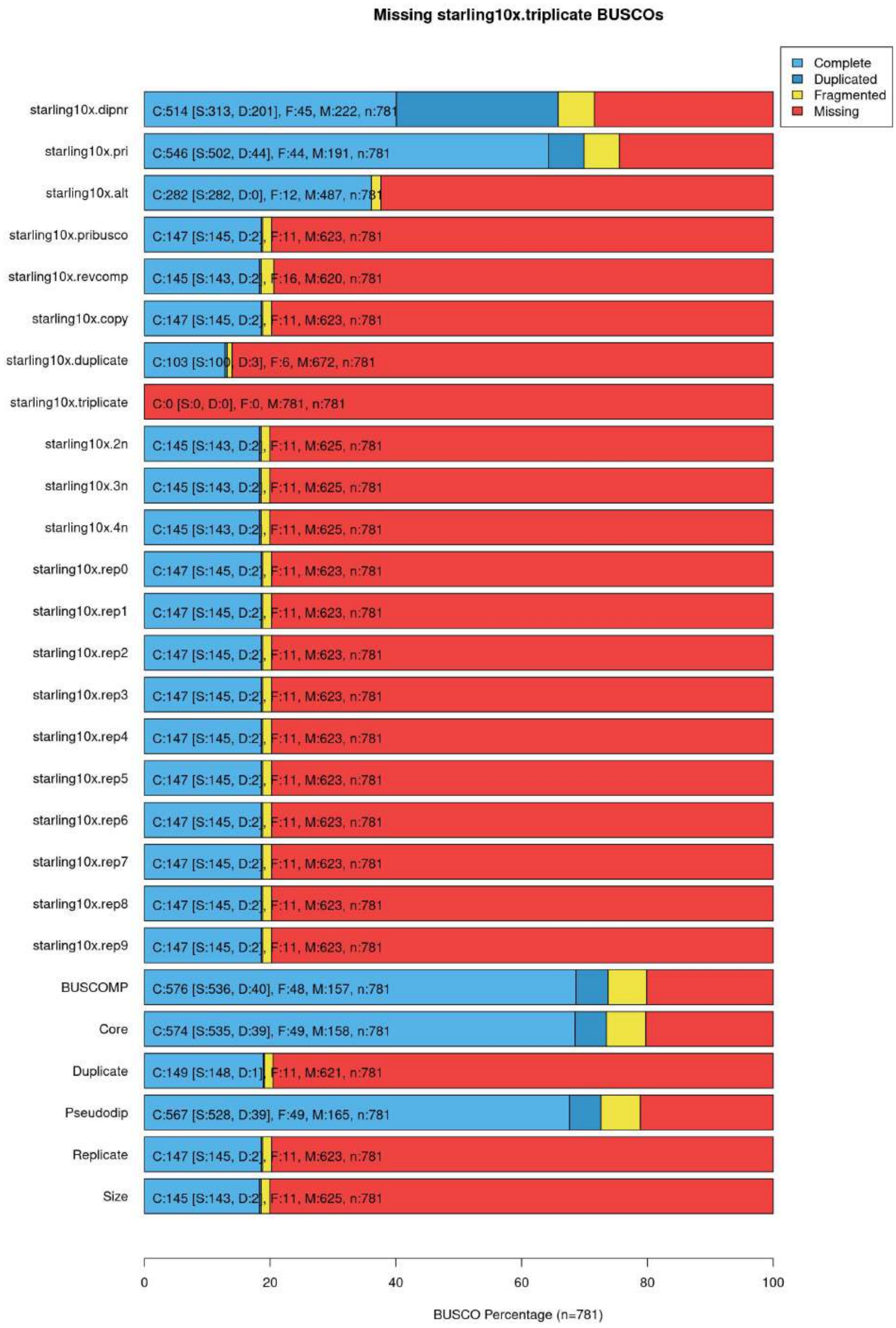

BUSCOMP ratings for `Missing` starling10x.triplicate BUSCO genes:

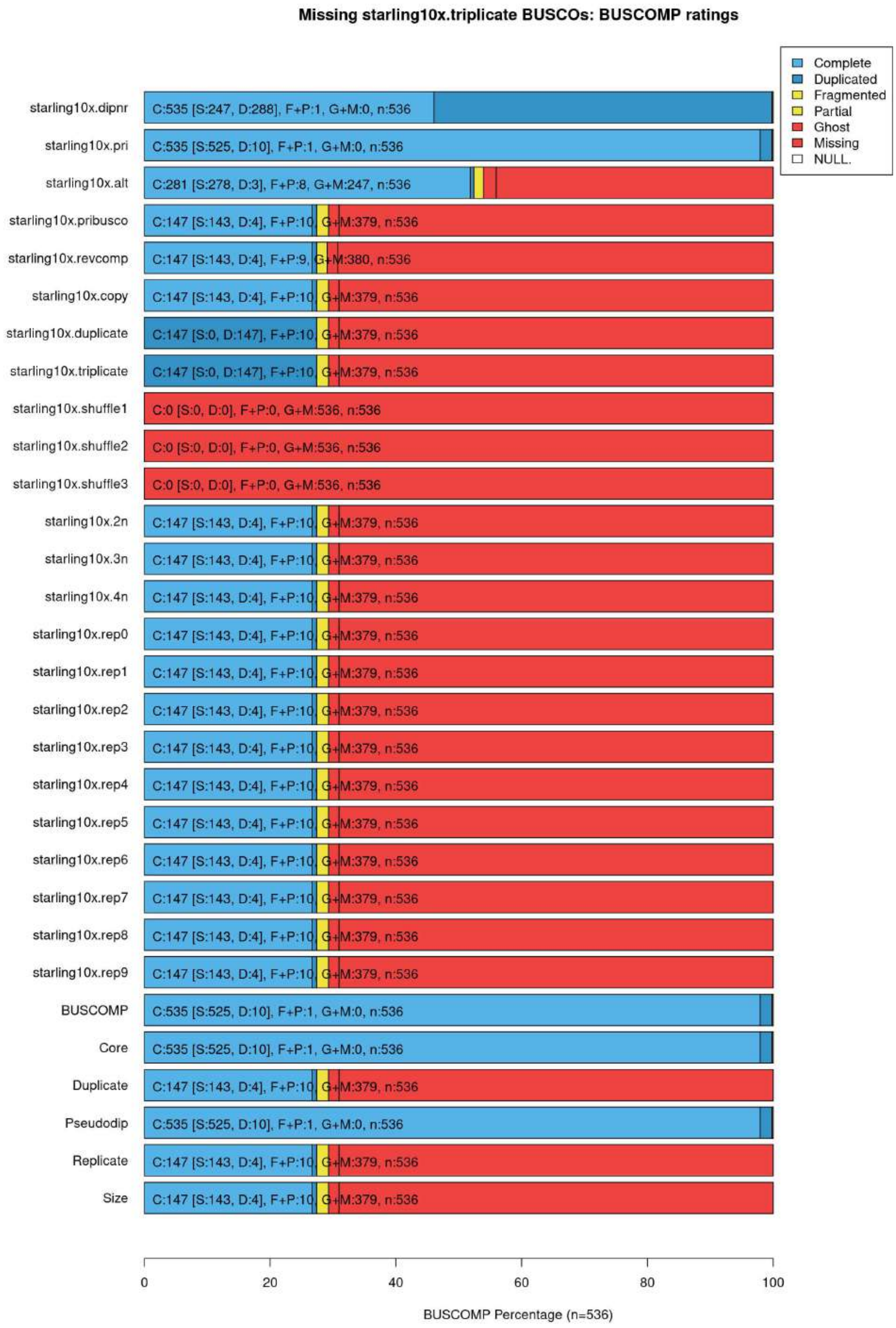

BUSCOMP ratings for `Missing` starling10x.triplicate BUSCOMP genes:

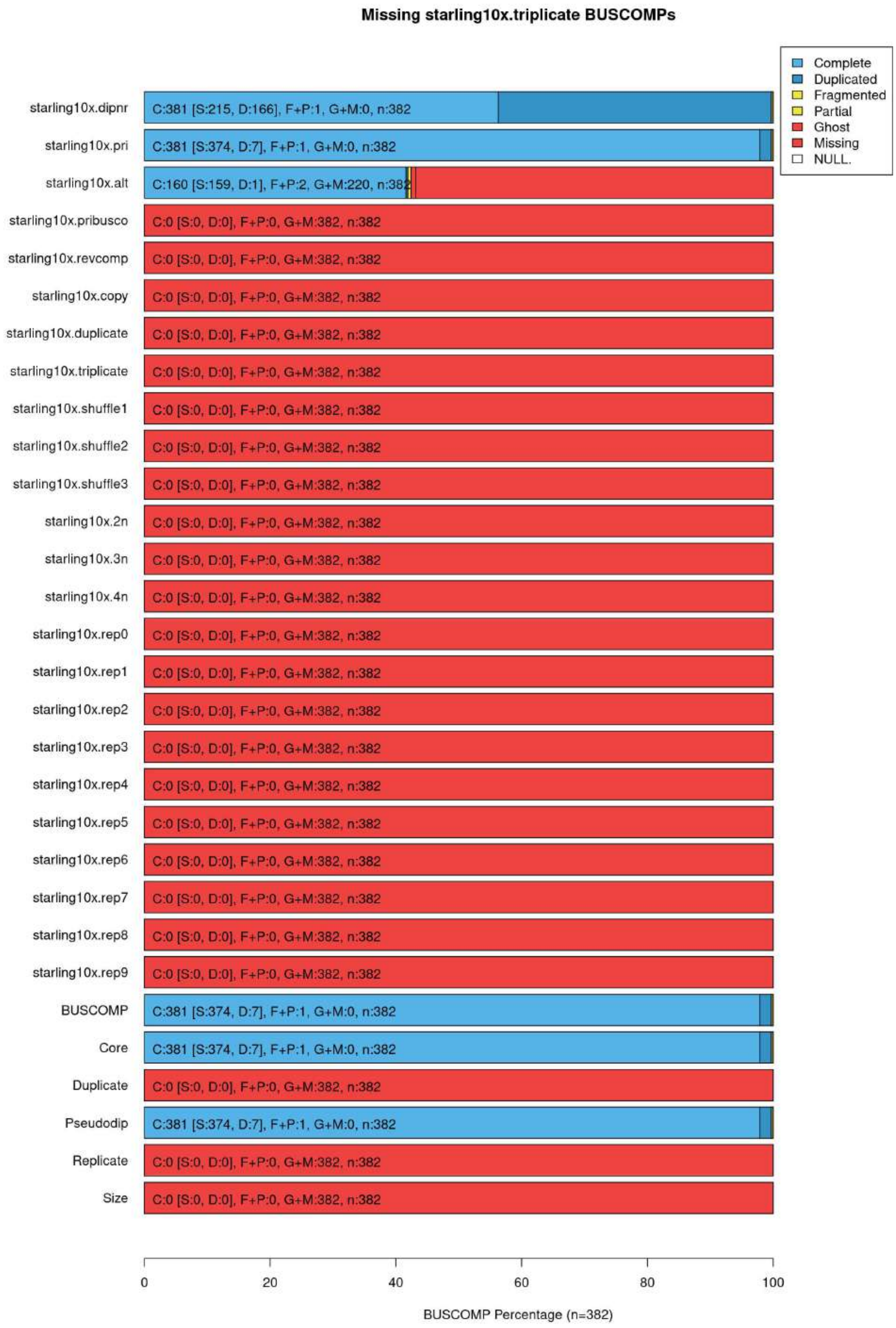

5.14 Missing Duplicate BUSCO genes

BUSCO ratings for `Missing` Duplicate BUSCO genes:

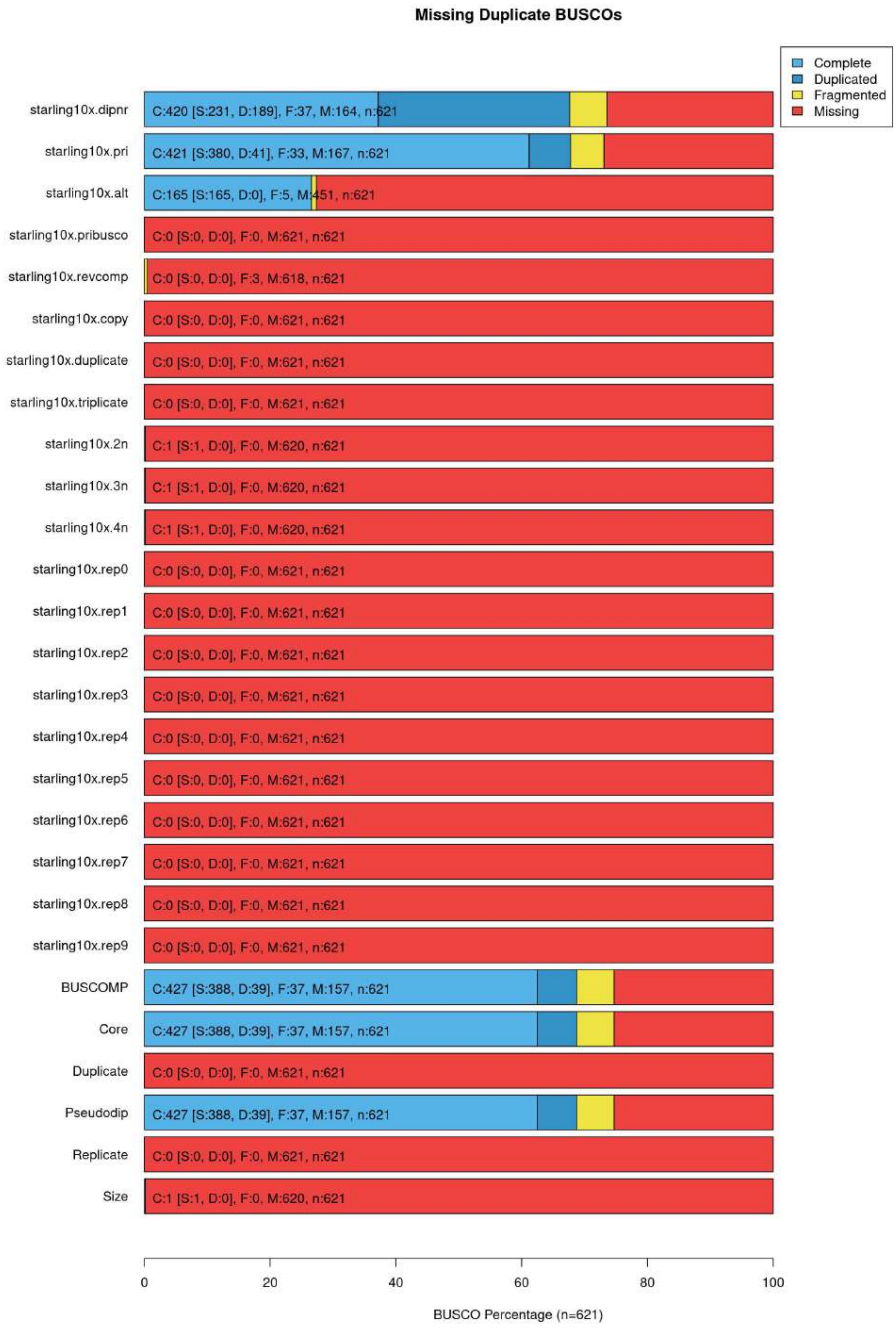

BUSCOMP ratings for `Missing` Duplicate BUSCO genes:

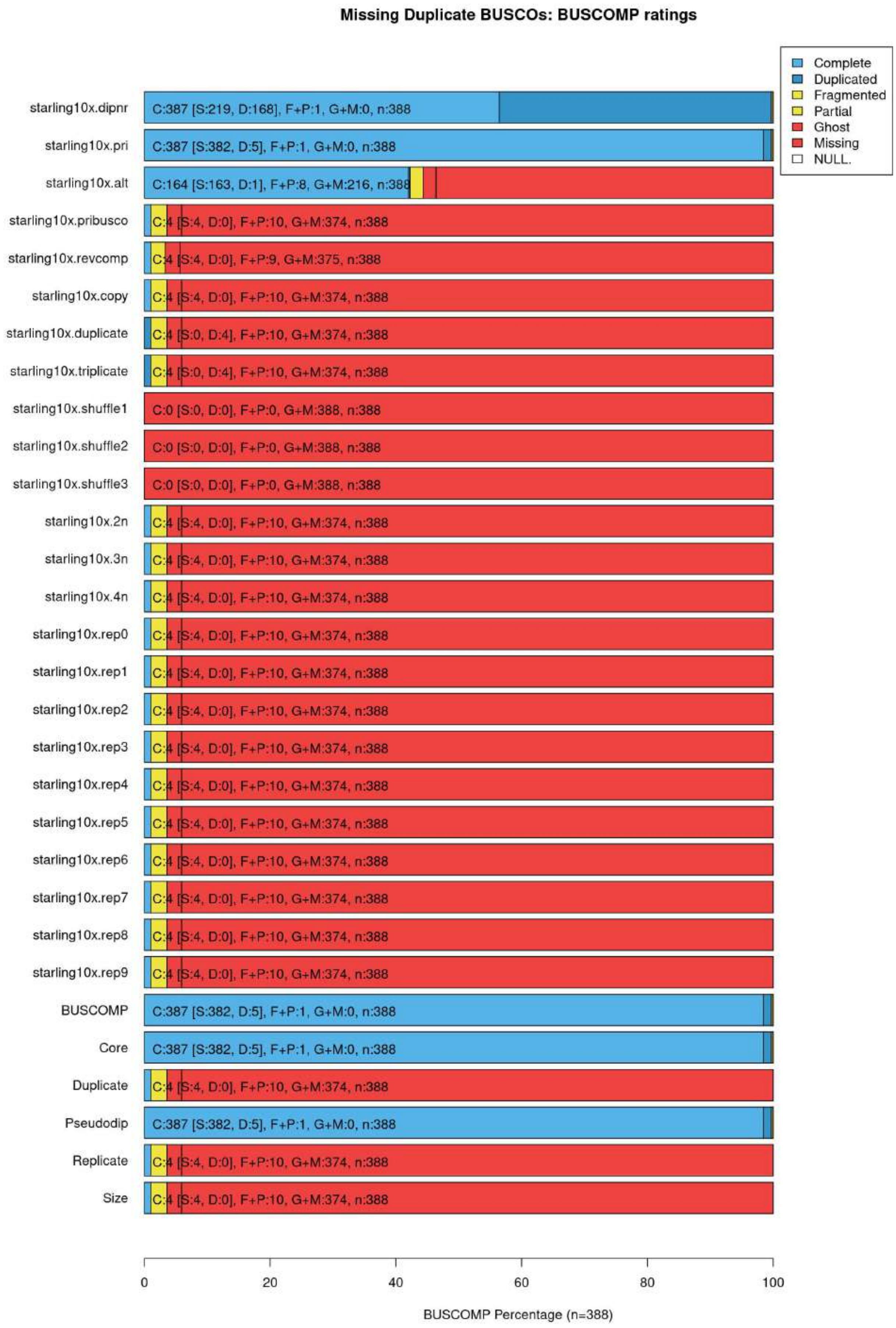

BUSCOMP ratings for `Missing` Duplicate BUSCOMP genes:

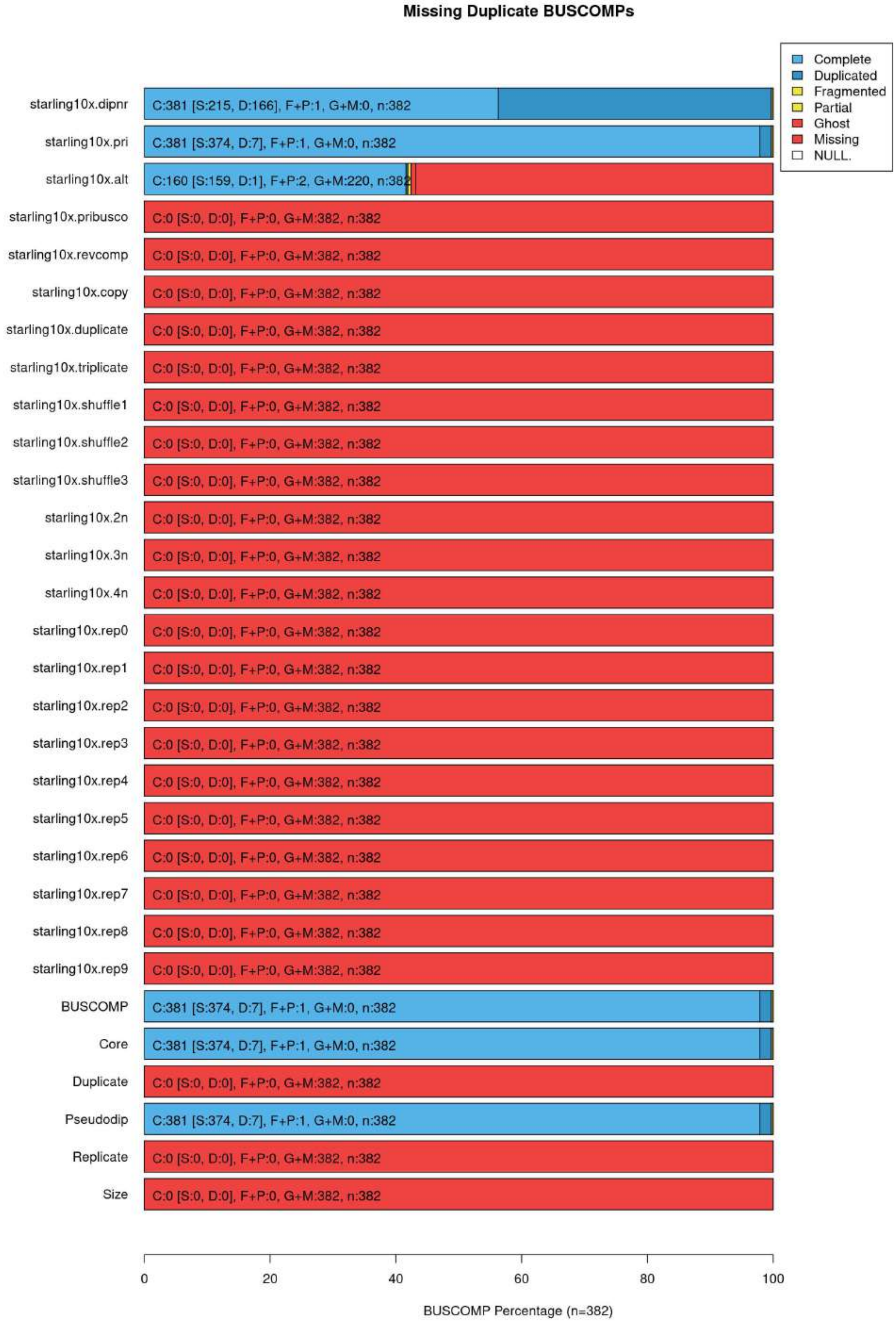

5.15 Missing starling10x.shuffle1 BUSCO genes

BUSCOMP ratings for `Missing` starling10x.shuffle1 BUSCOMP genes:

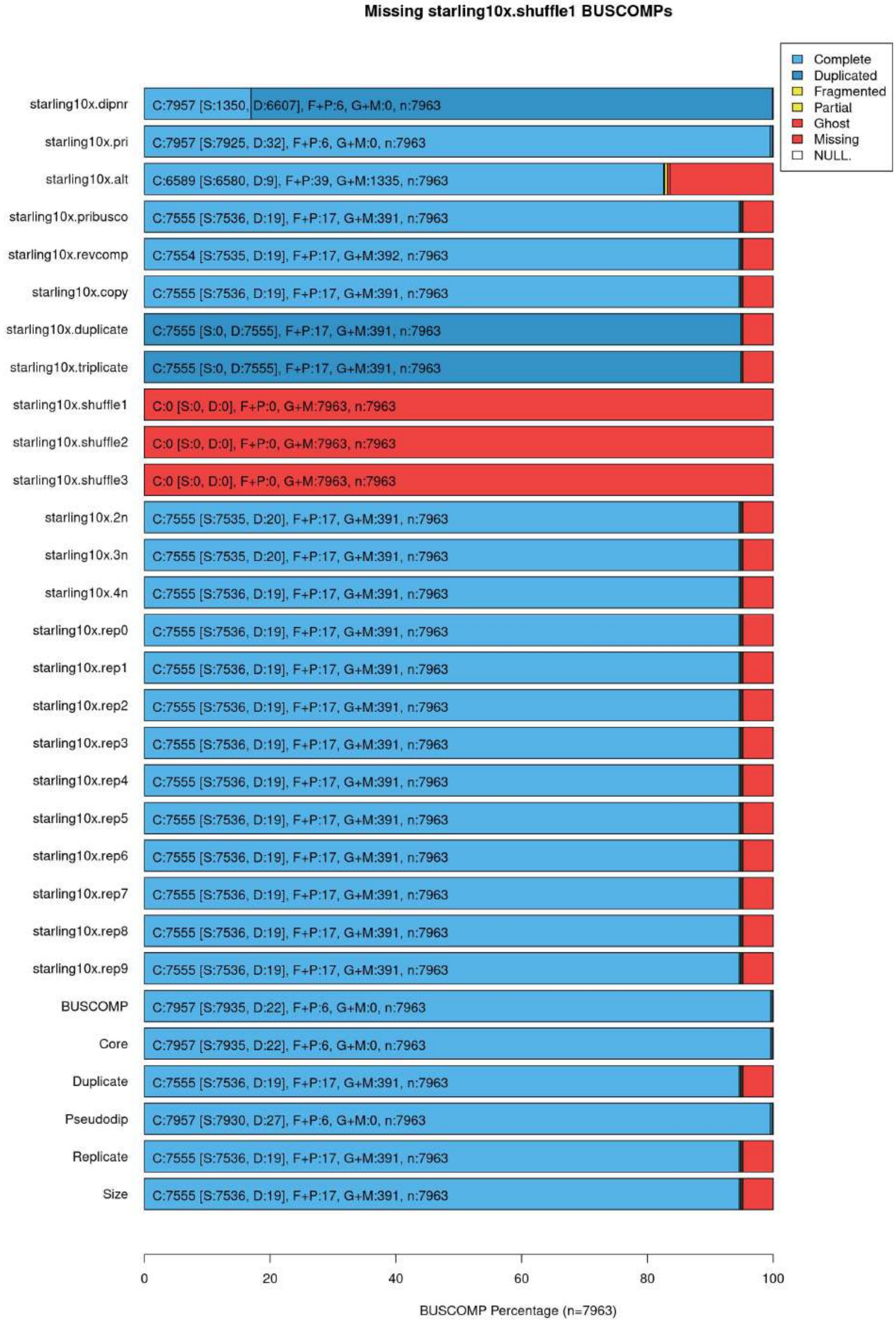

5.16 Missing starling10x.shuffle2 BUSCO genes

BUSCOMP ratings for `Missing` starling10x.shuffle2 BUSCOMP genes:

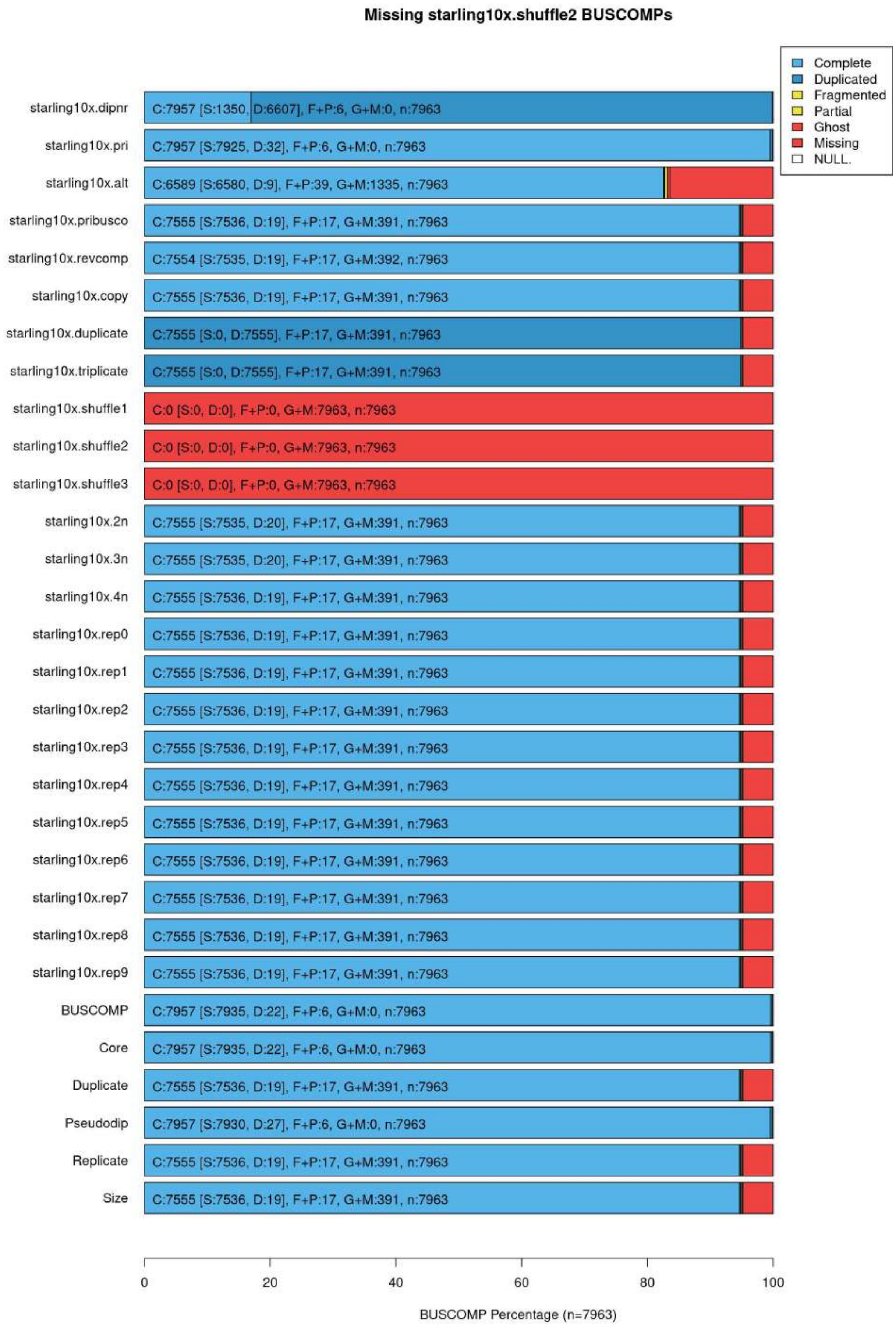

5.17 Missing starling10x.shuffle3 BUSCO genes

BUSCOMP ratings for `Missing` starling10x.shuffle3 BUSCOMP genes:

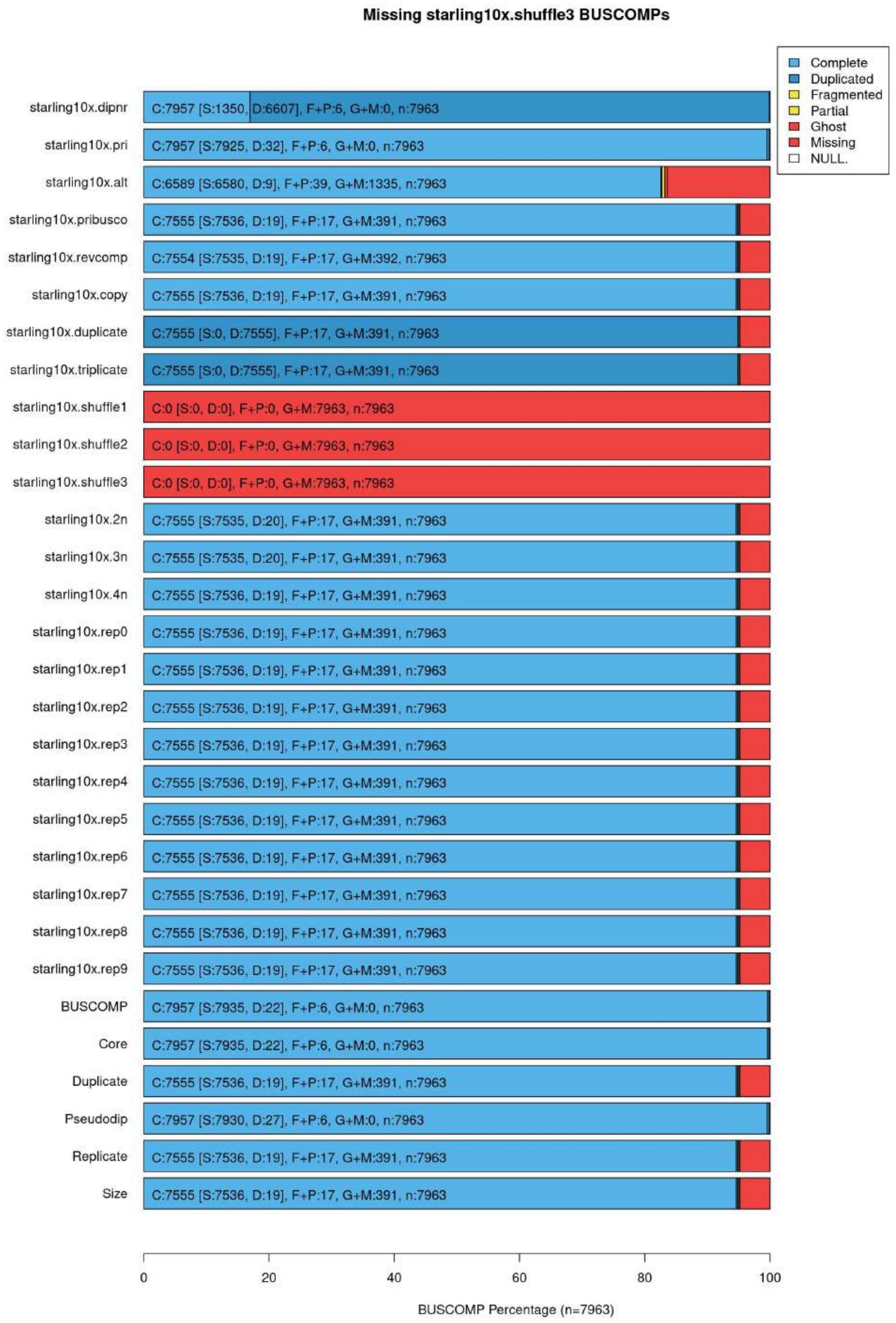

5.18 Missing starling10x.2n BUSCO genes

BUSCO ratings for `Missing` starling10x.2n BUSCO genes:

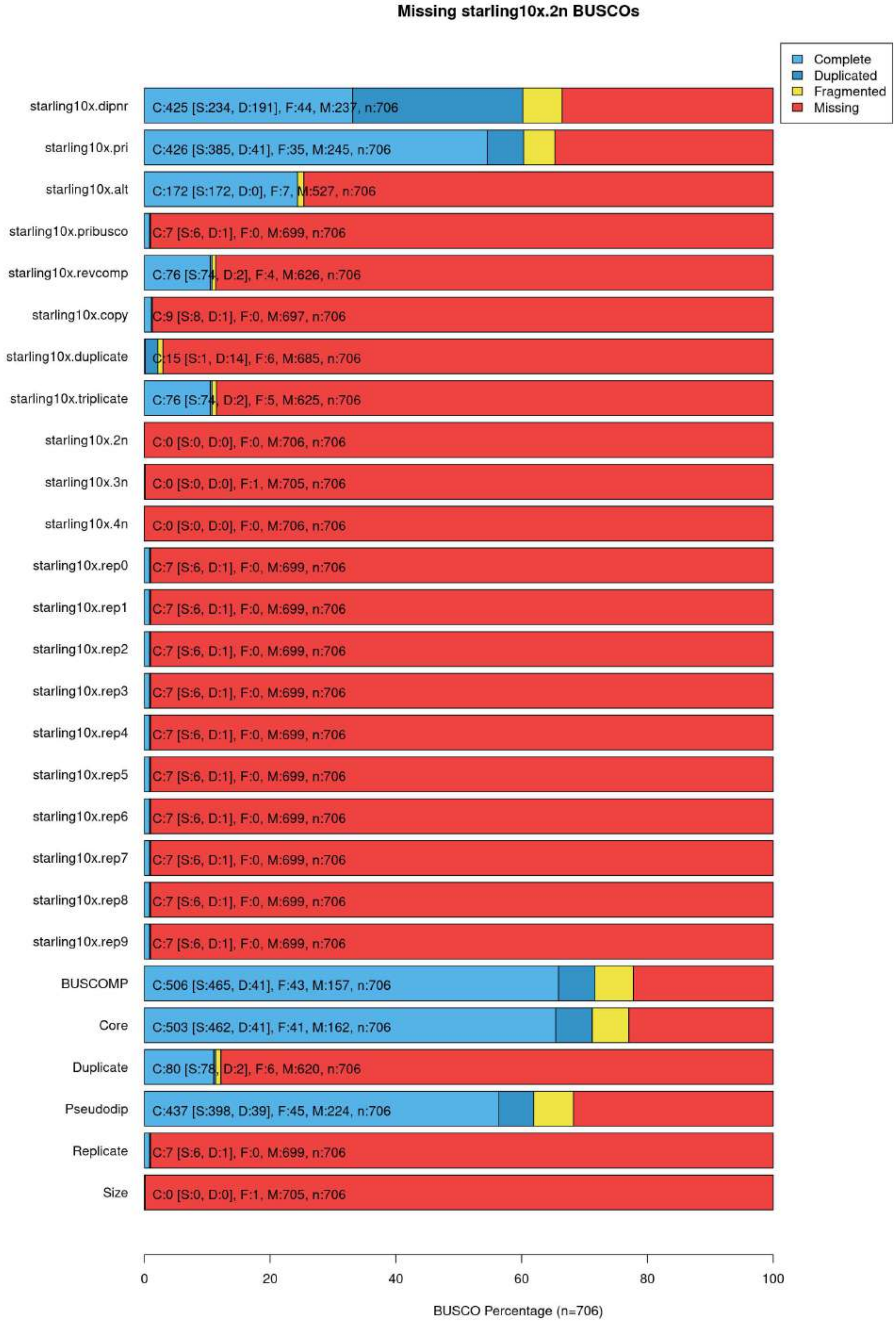

BUSCOMP ratings for `Missing` starling10x.2n BUSCO genes:

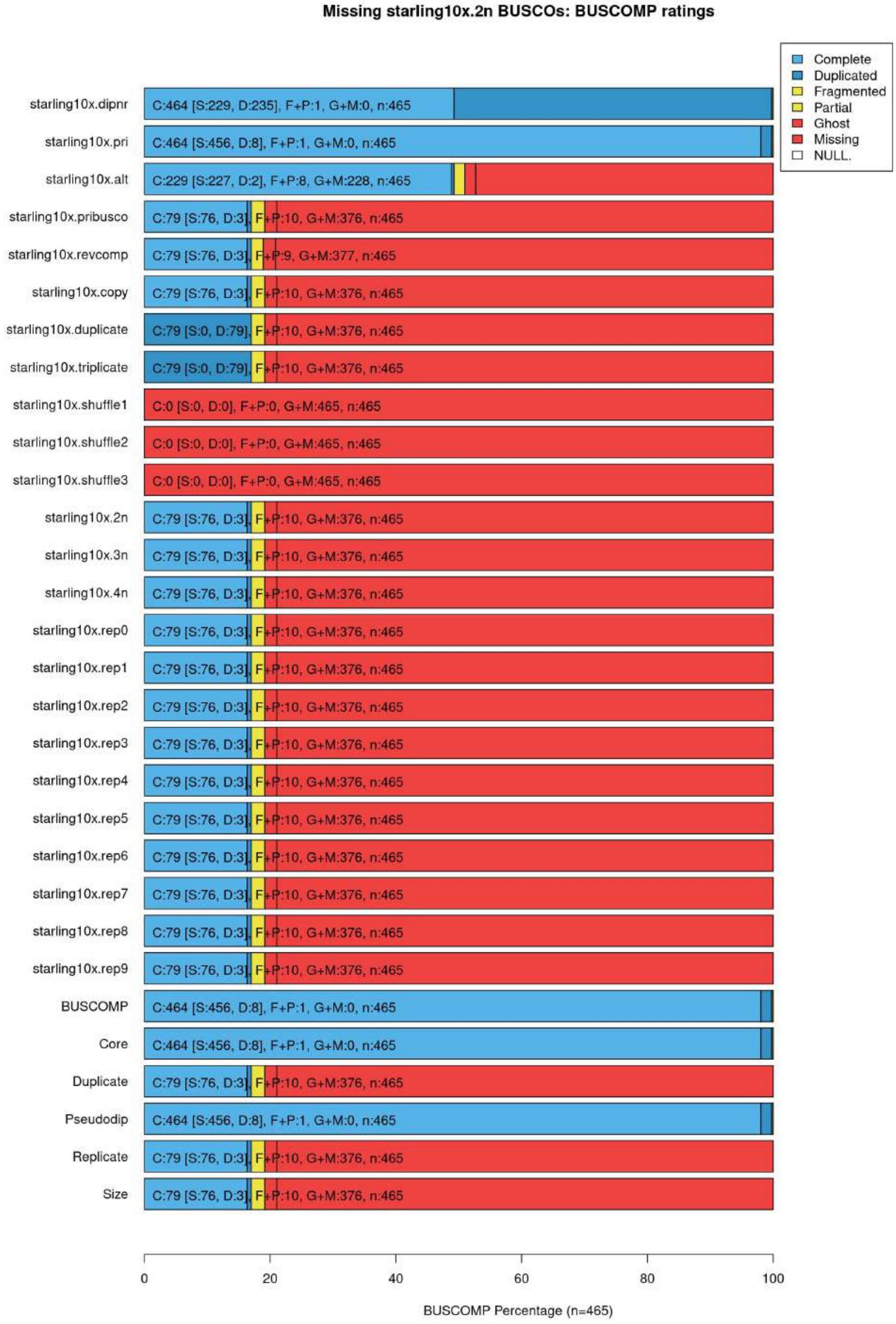

BUSCOMP ratings for `Missing` starling10x.2n BUSCOMP genes:

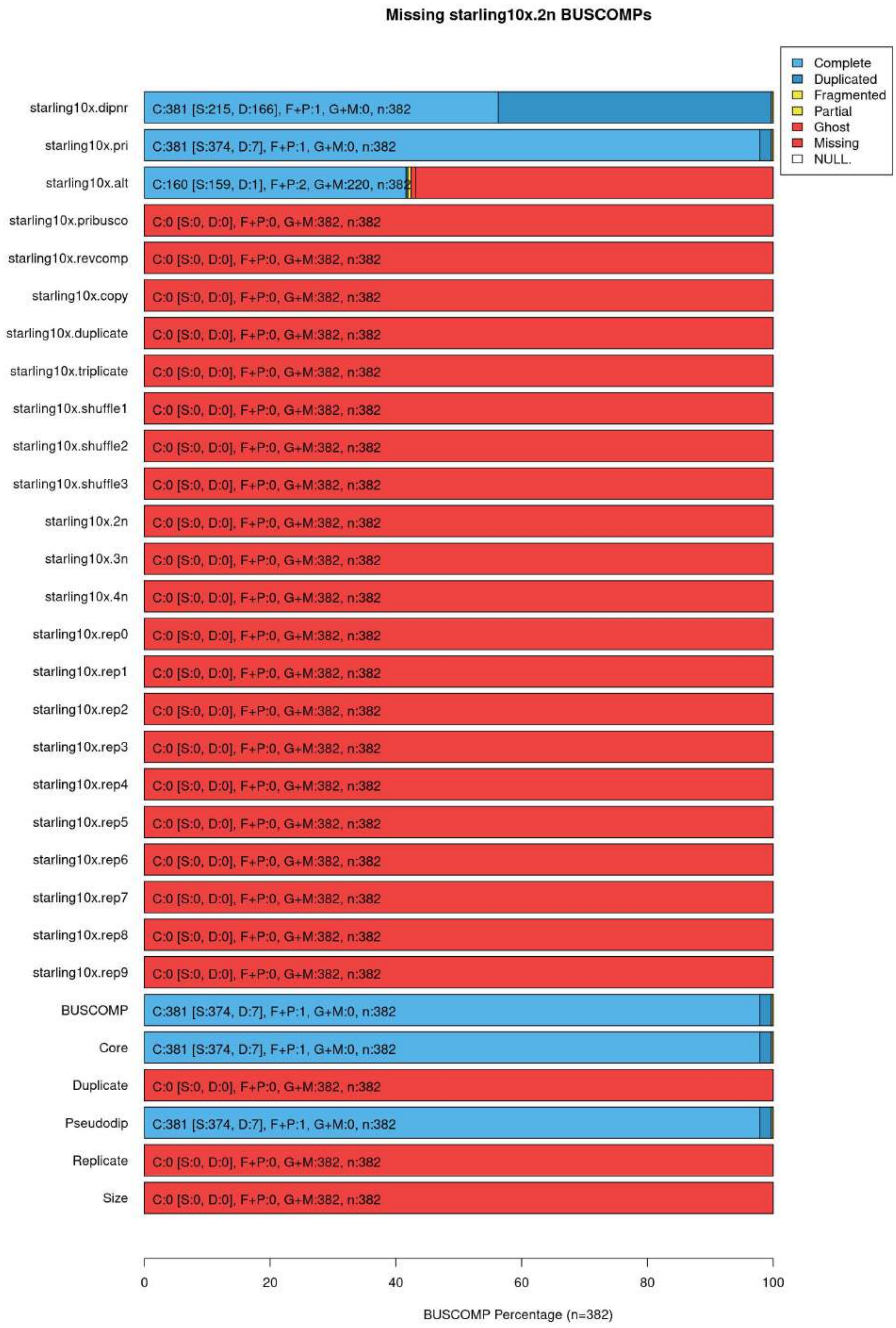

5.19 Missing starling10x.3n BUSCO genes

BUSCO ratings for `Missing` starling10x.3n BUSCO genes:

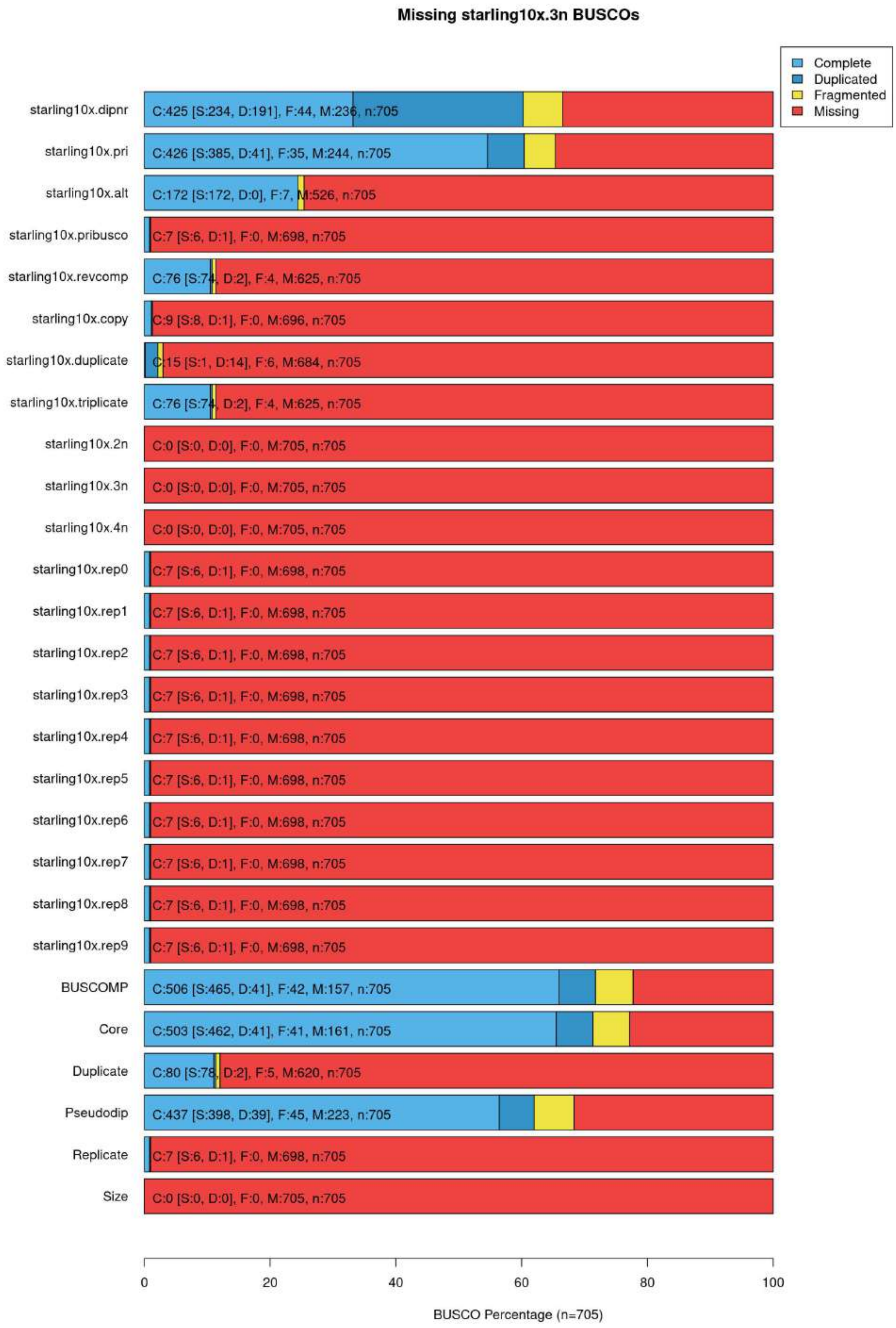

BUSCOMP ratings for `Missing` starling10x.3n BUSCO genes:

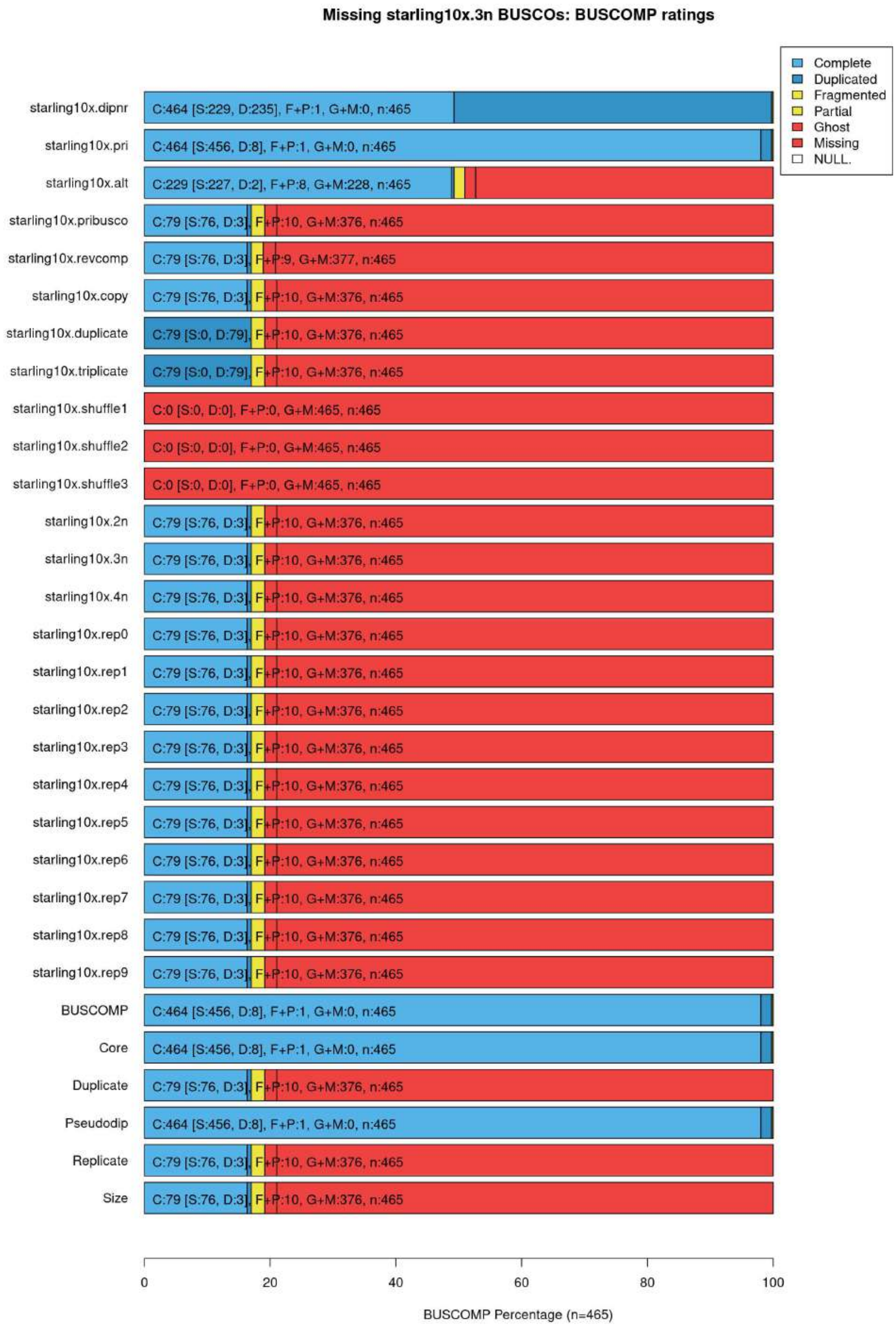

BUSCOMP ratings for `Missing` starling10x.3n BUSCOMP genes:

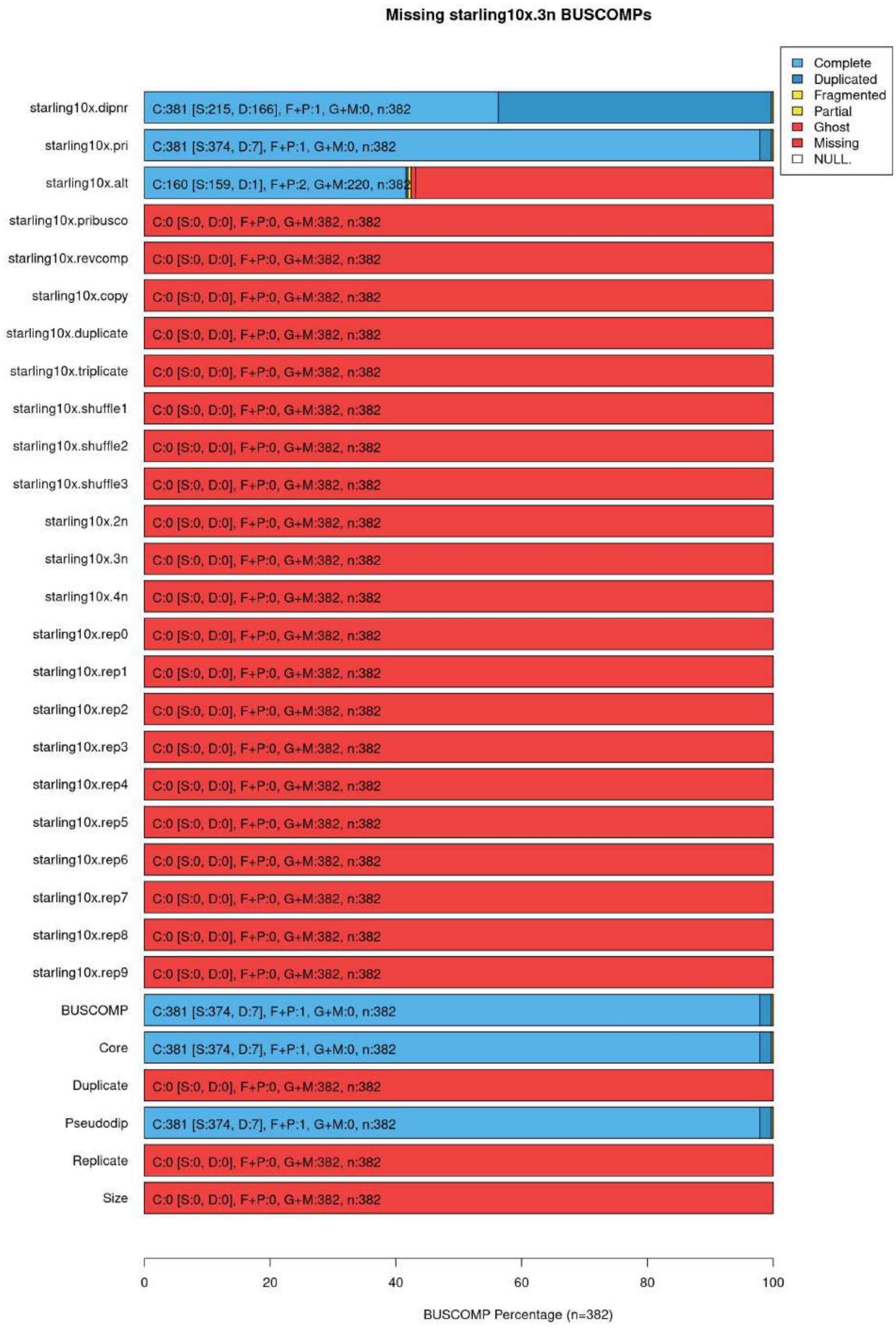

5.20 Missing starling10x.4n BUSCO genes

BUSCO ratings for `Missing` starling10x.4n BUSCO genes:

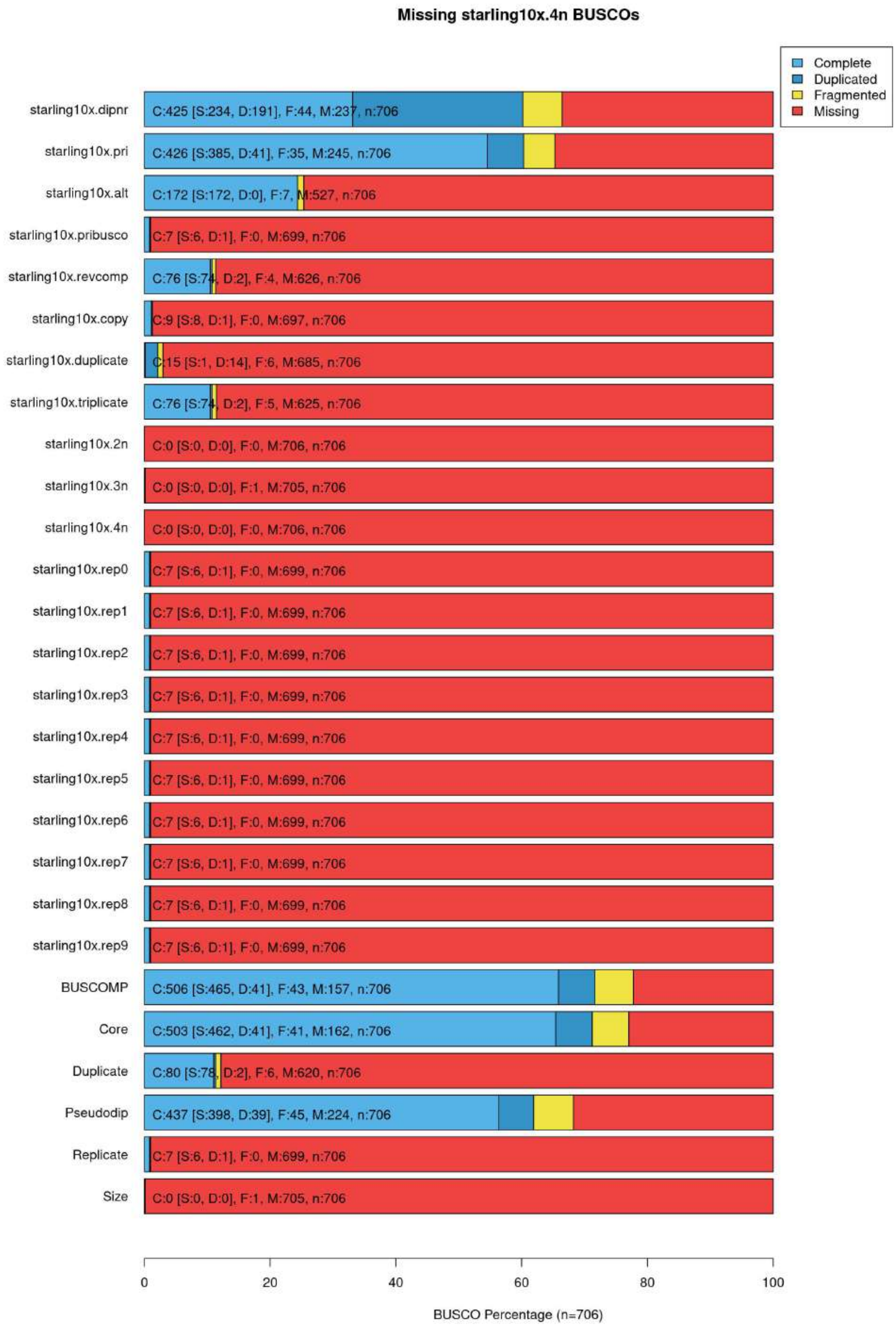

BUSCOMP ratings for `Missing` starling10x.4n BUSCO genes:

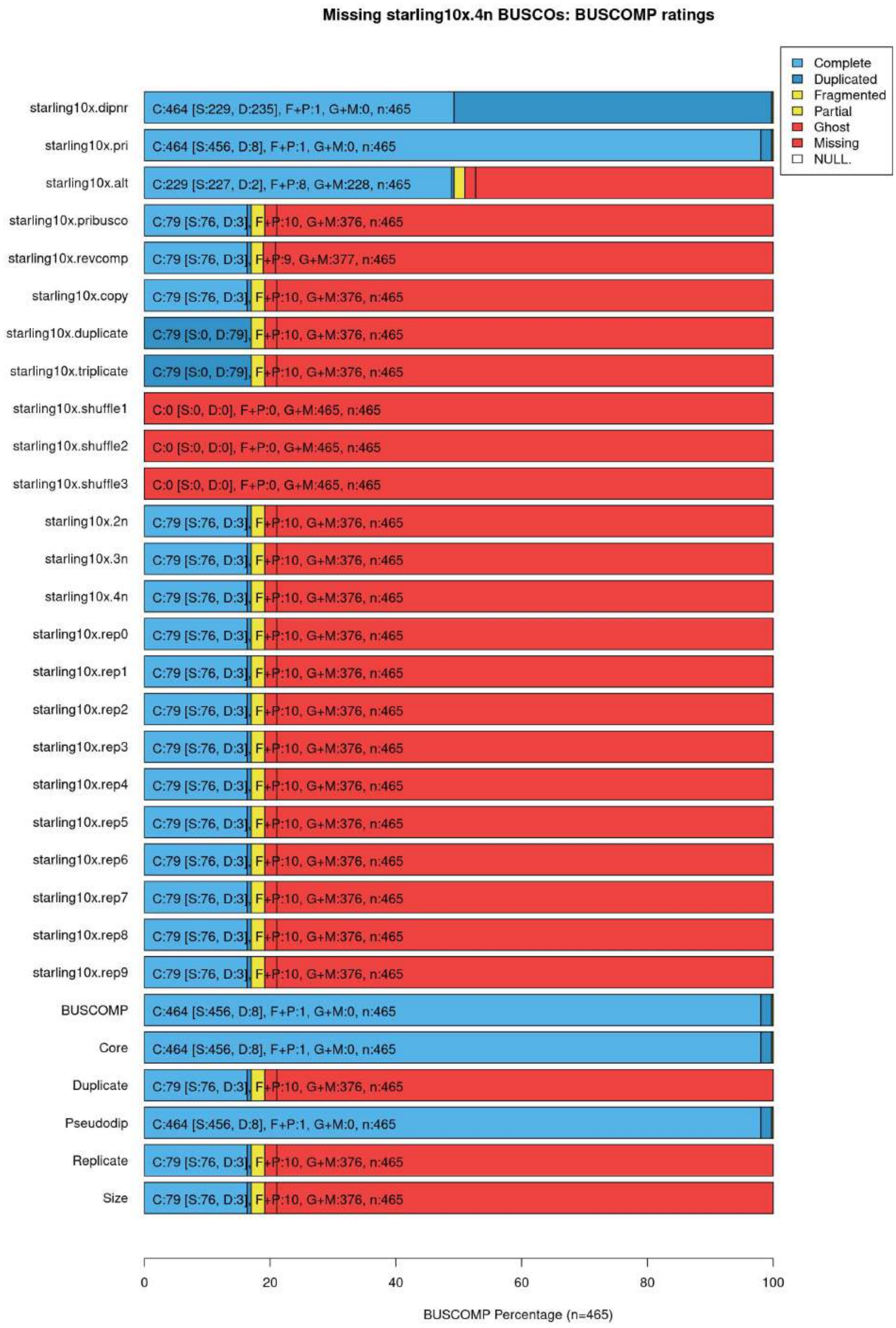

BUSCOMP ratings for `Missing` starling10x.4n BUSCOMP genes:

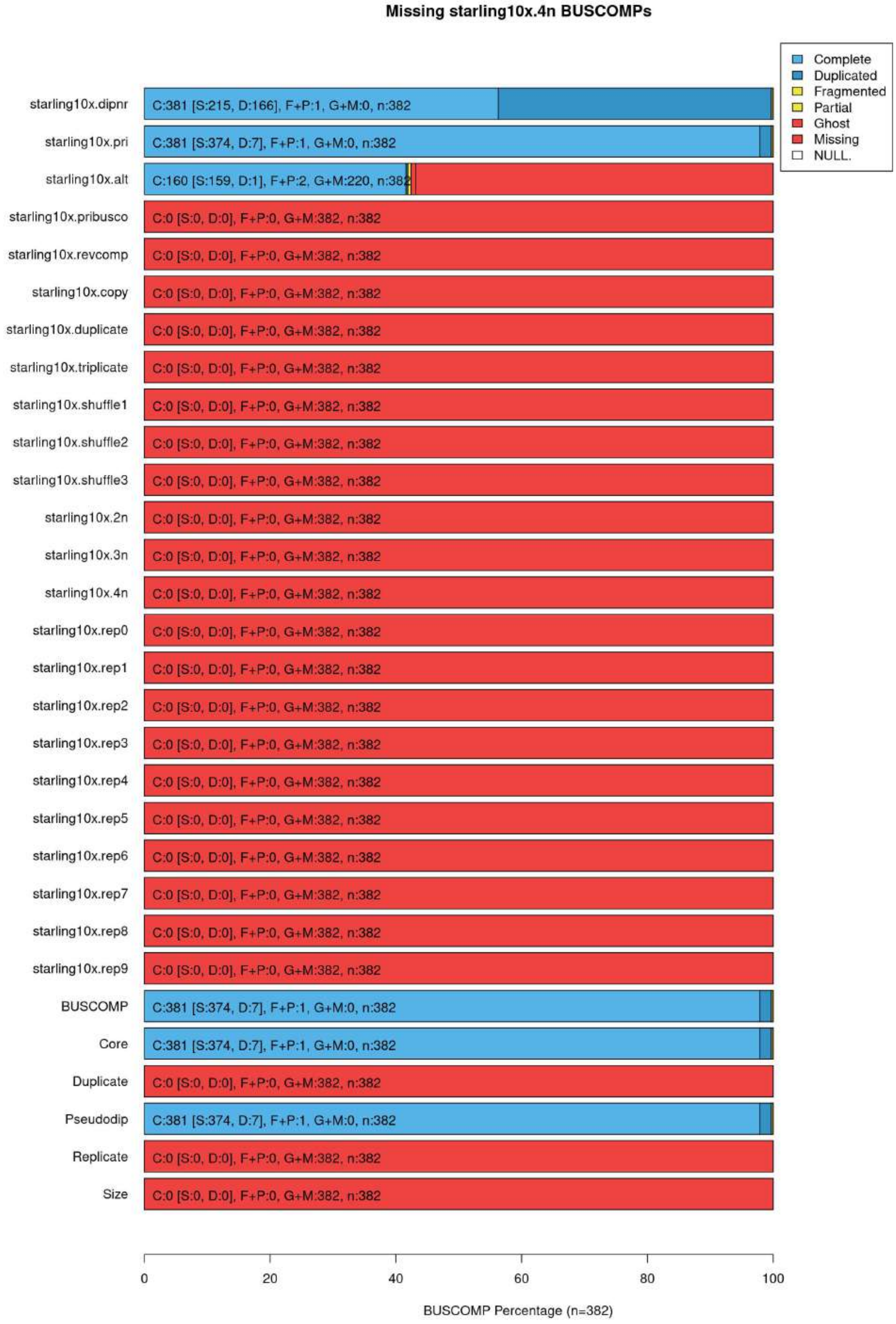

5.21 Missing Size BUSCO genes

BUSCO ratings for `Missing` Size BUSCO genes:

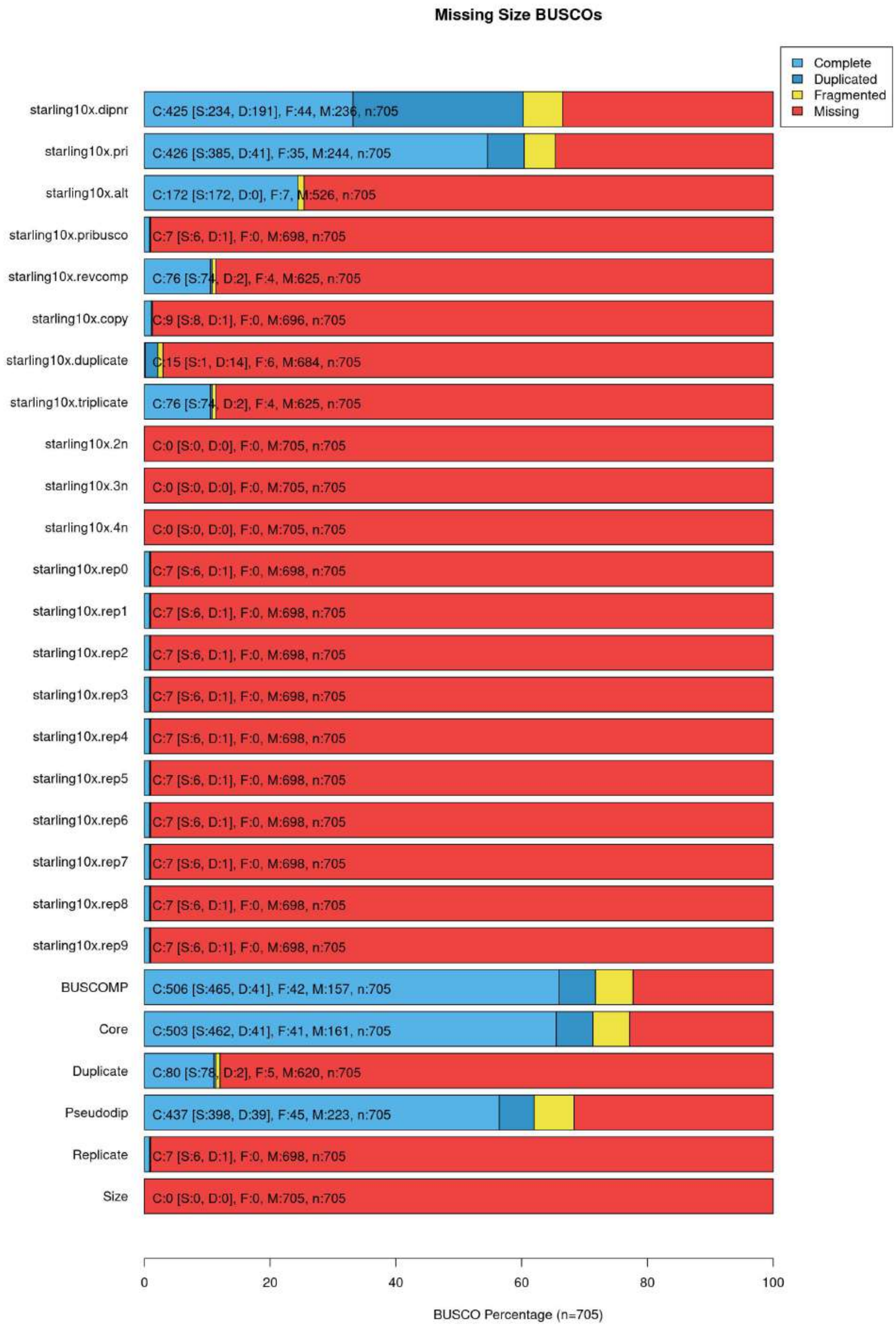

BUSCOMP ratings for `Missing` Size BUSCO genes:

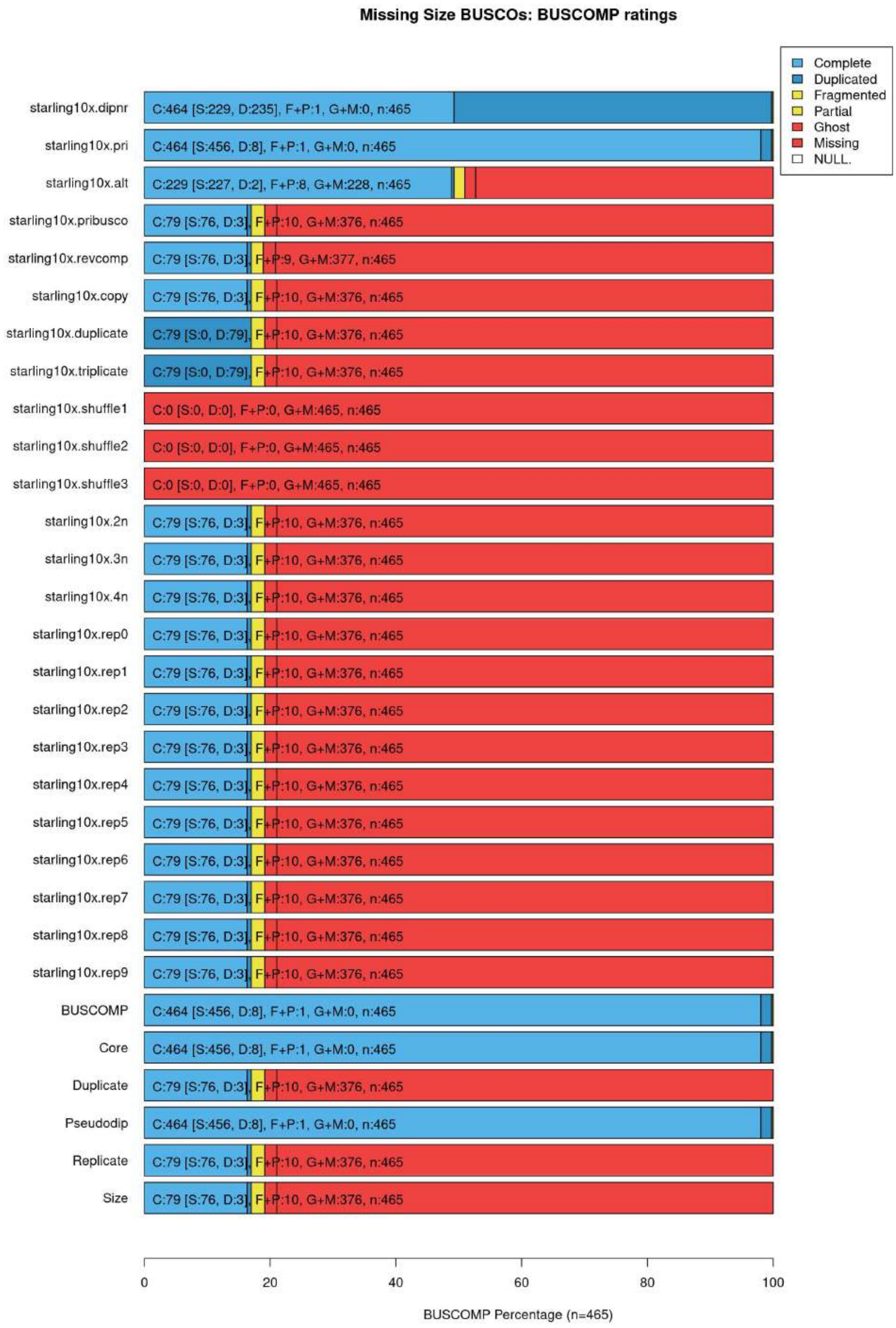

BUSCOMP ratings for `Missing` Size BUSCOMP genes:

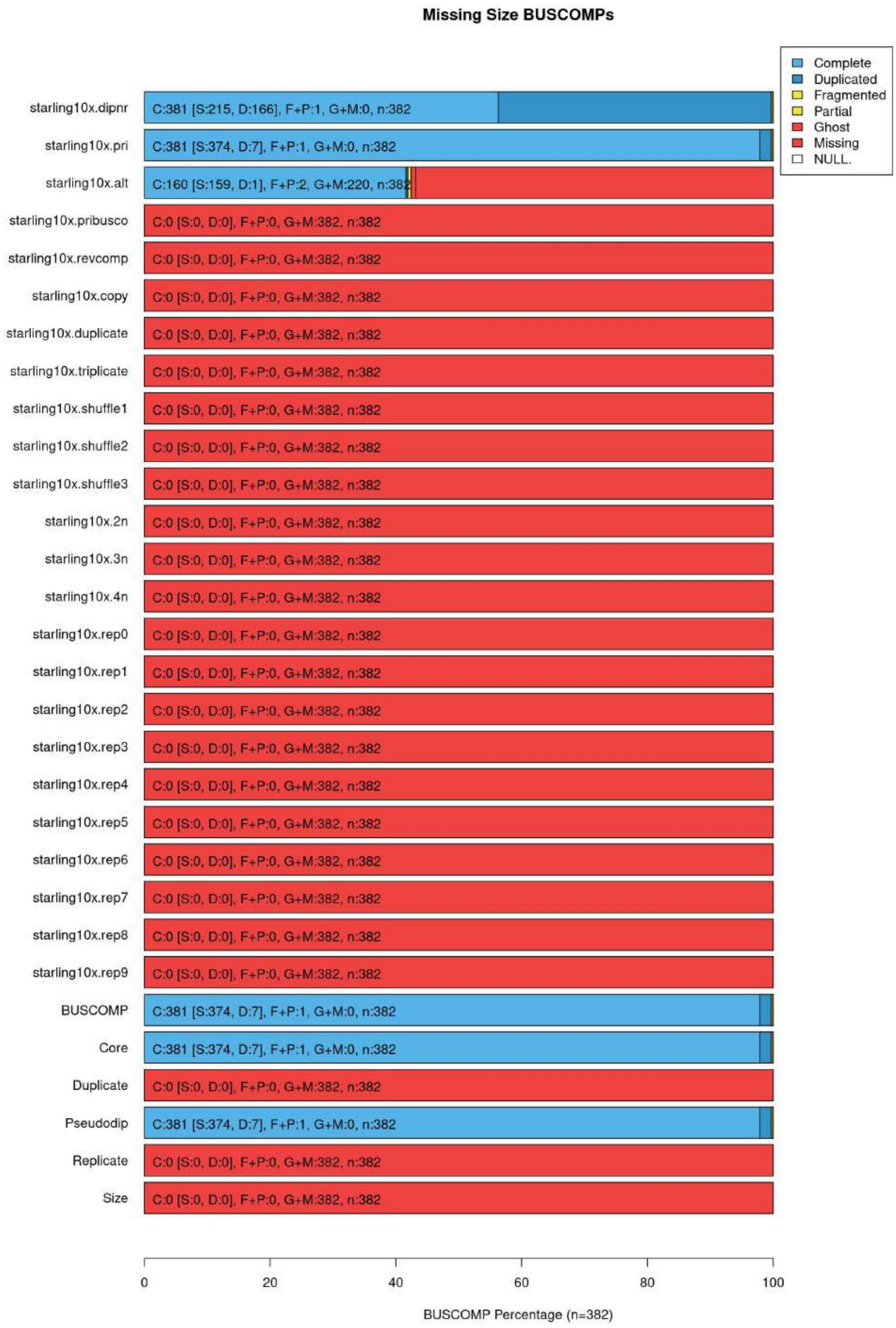

5.22 Missing starling10x.rep0 BUSCO genes

BUSCO ratings for `Missing` starling10x.rep0 BUSCO genes:

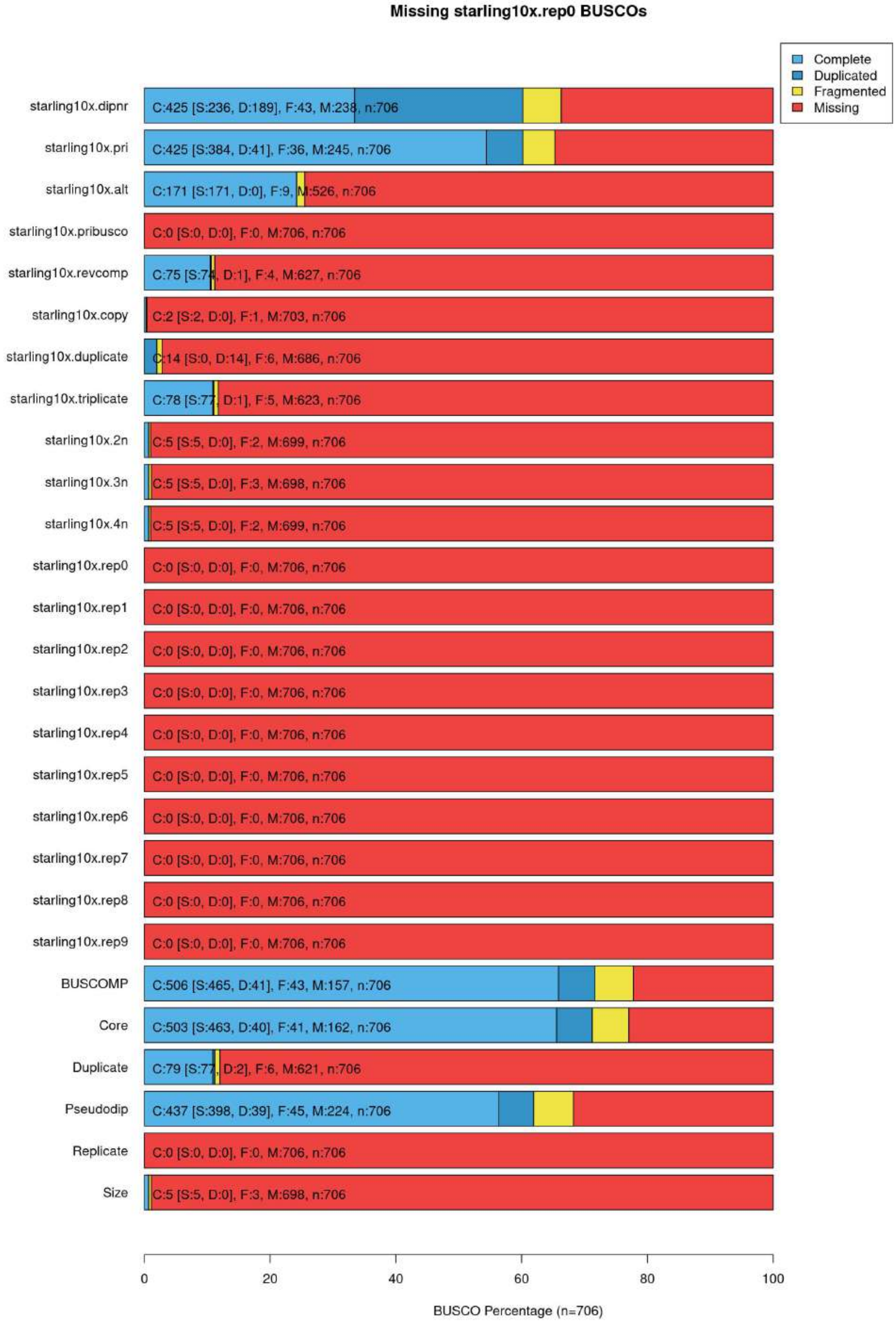

BUSCOMP ratings for `Missing` starling10x.rep0 BUSCO genes:

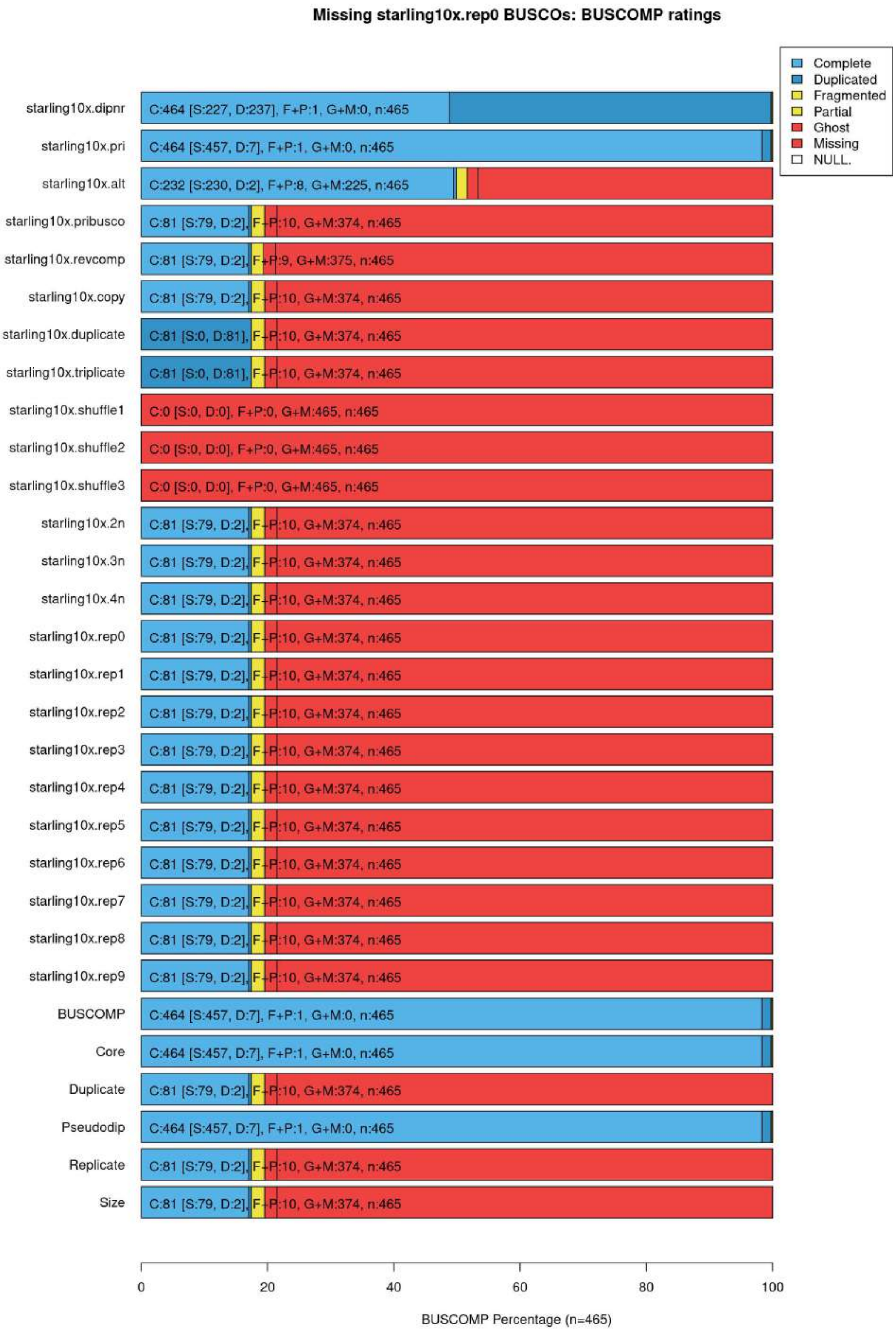

BUSCOMP ratings for `Missing` starling10x.rep0 BUSCOMP genes:

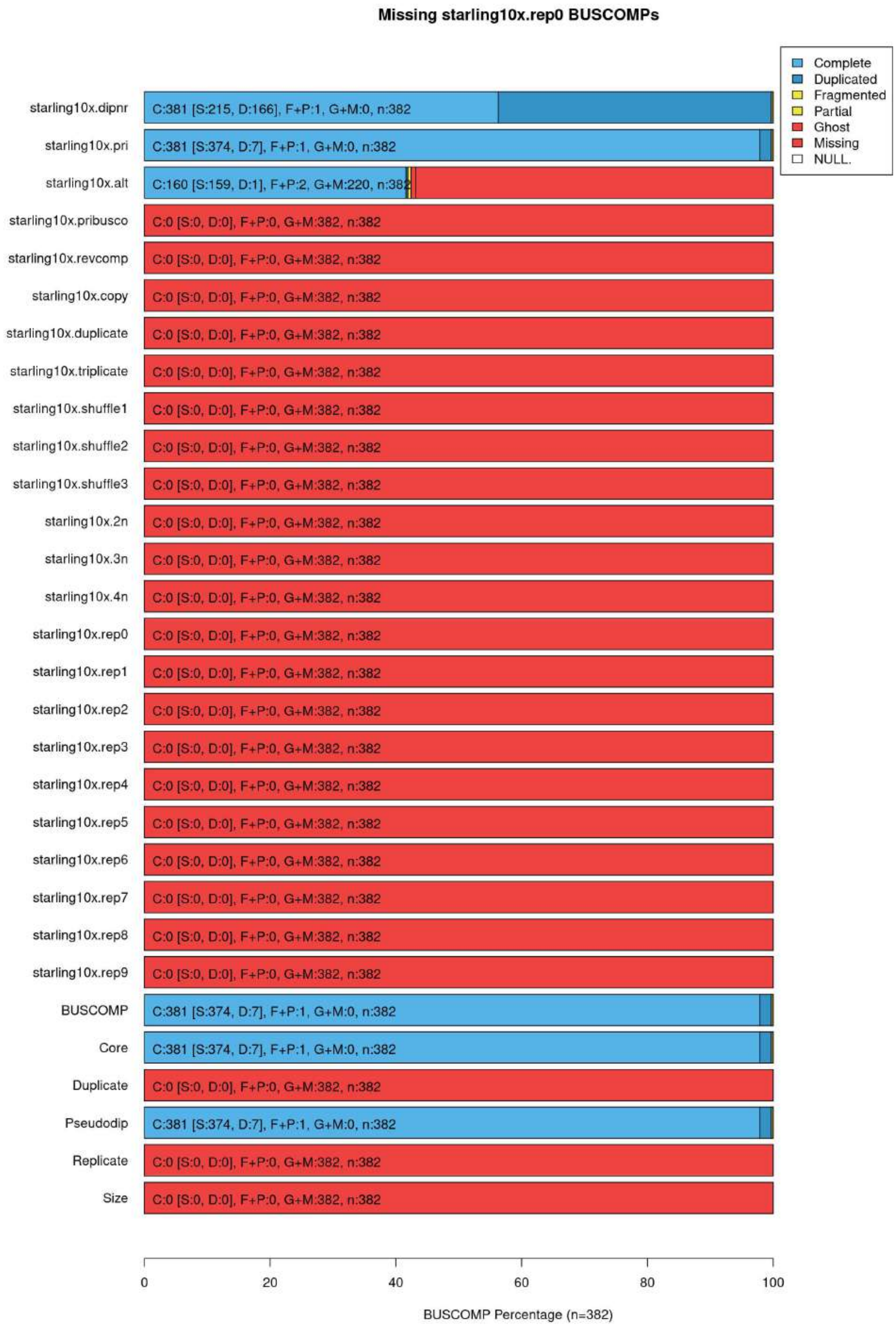

5.23 Missing starling10x.rep1 BUSCO genes

BUSCO ratings for `Missing` starling10x.rep1 BUSCO genes:

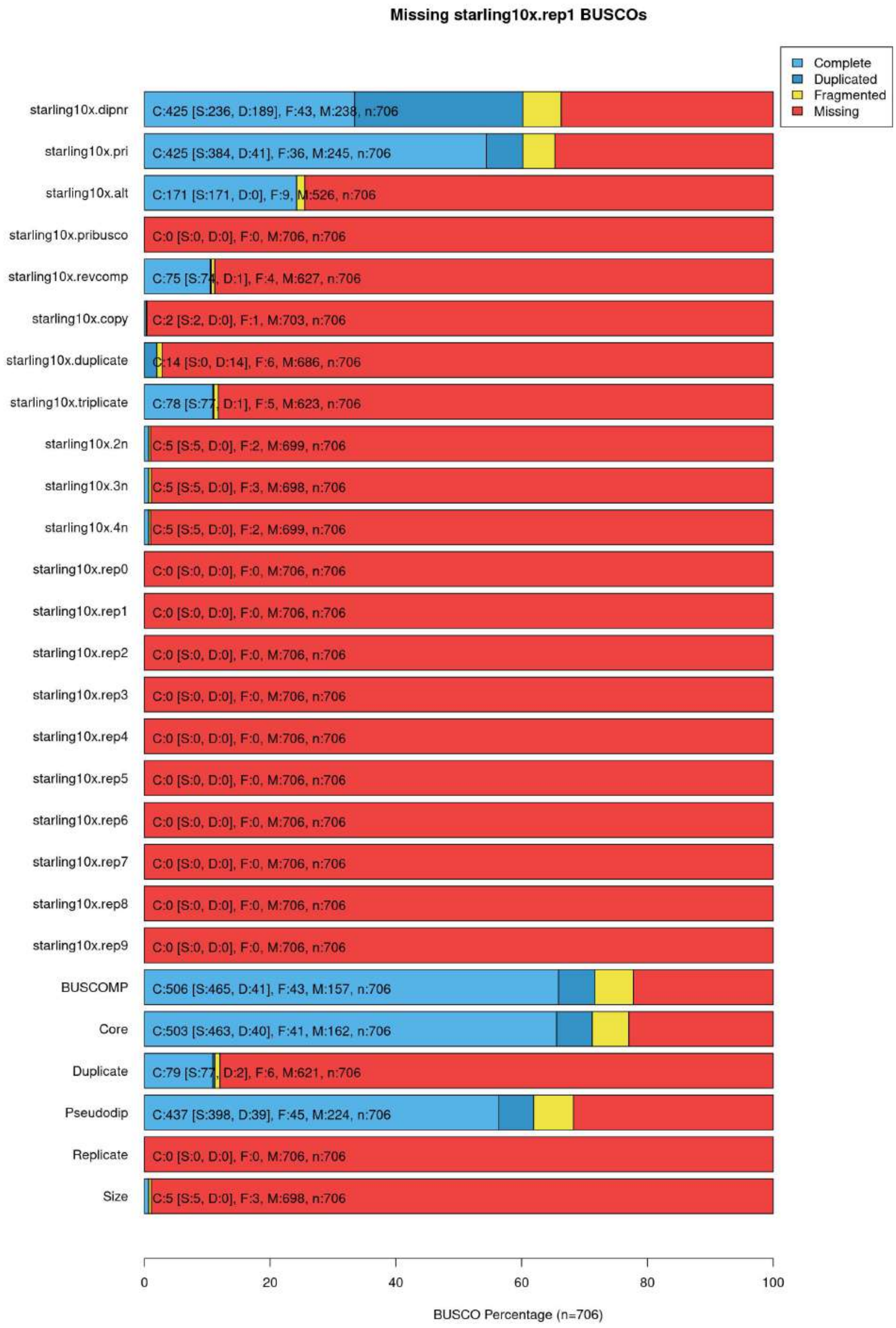

BUSCOMP ratings for `Missing` starling10x.rep1 BUSCO genes:

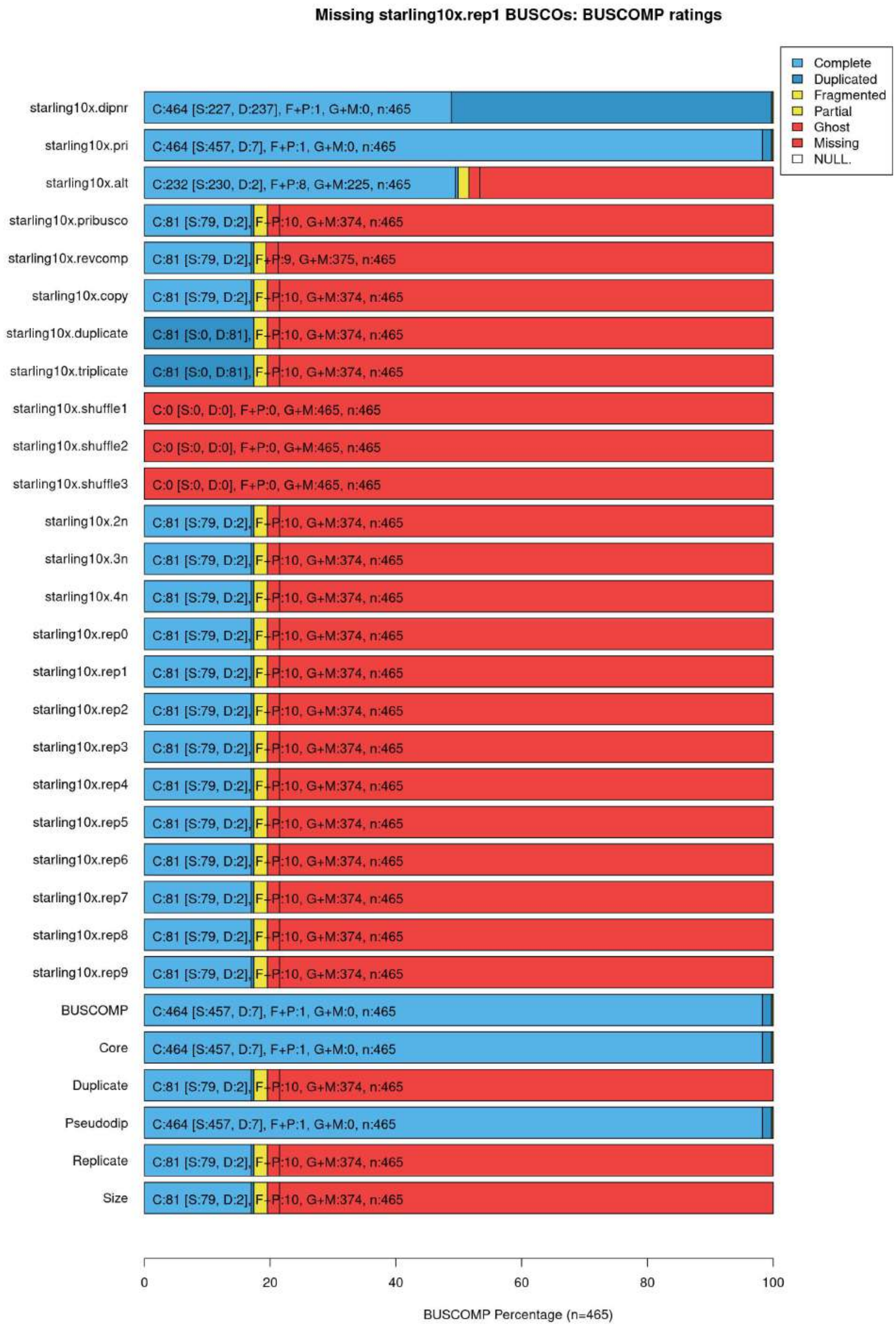

BUSCOMP ratings for `Missing` starling10x.rep1 BUSCOMP genes:

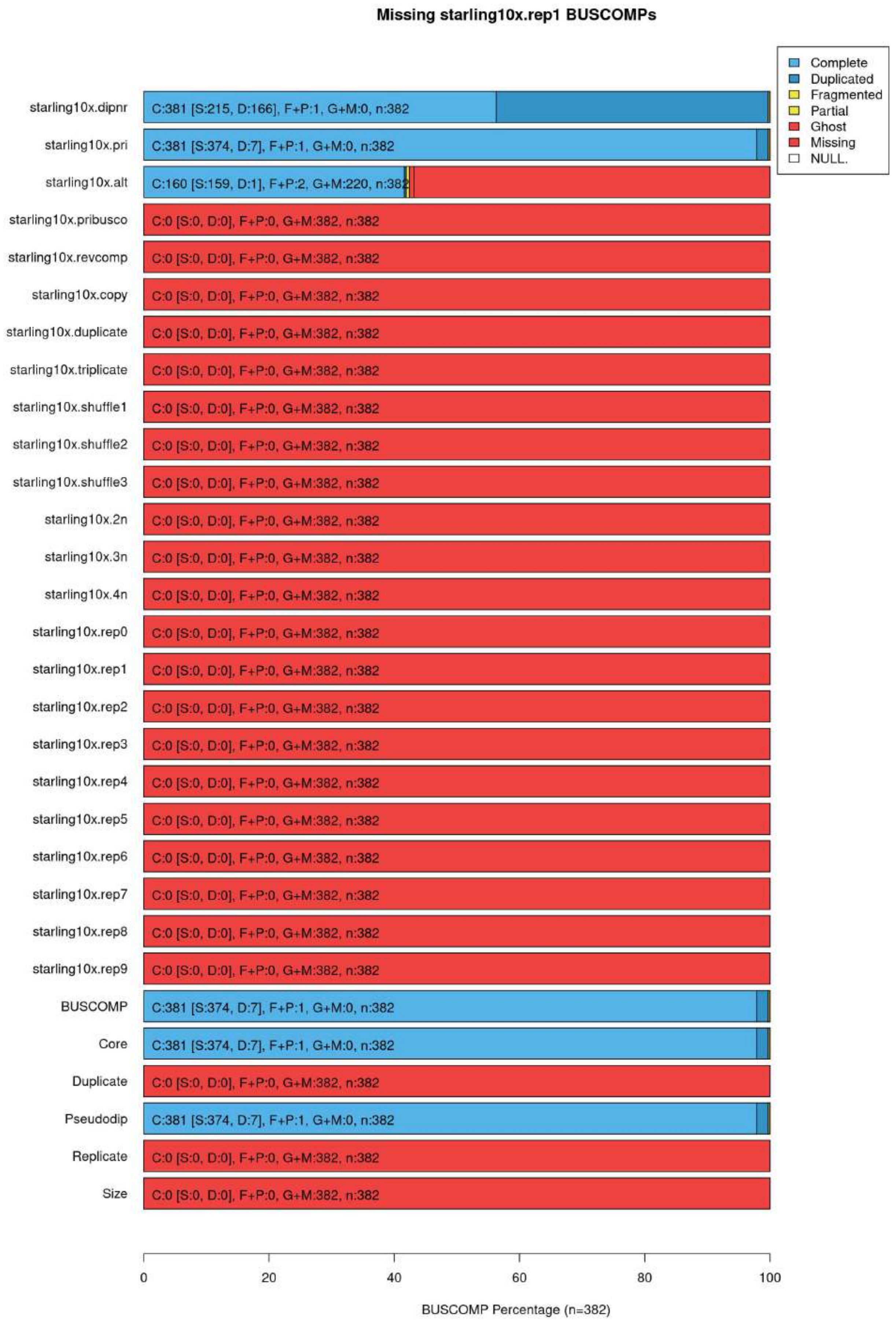

5.24 Missing starling10x.rep2 BUSCO genes

BUSCO ratings for `Missing` starling10x.rep2 BUSCO genes:

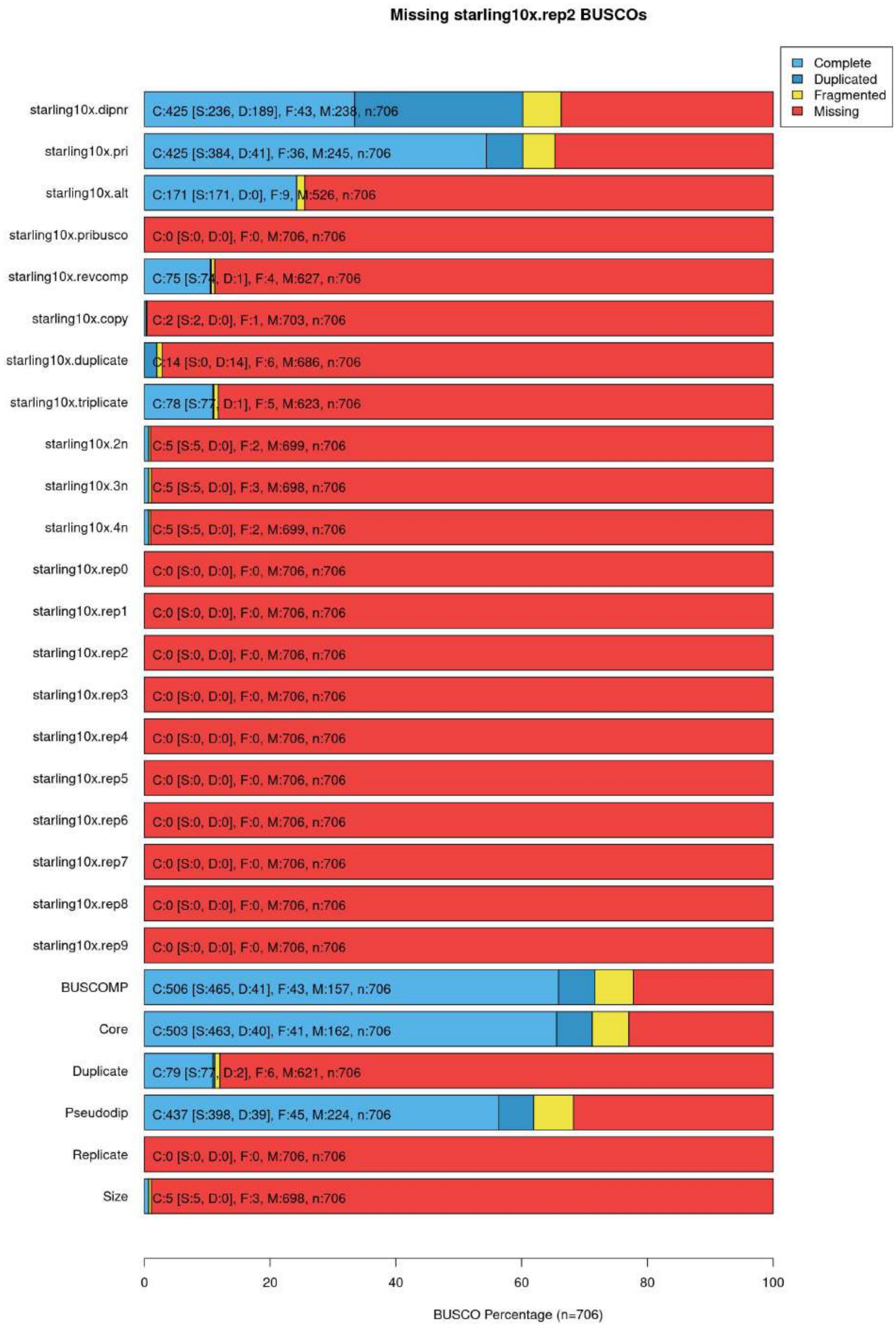

BUSCOMP ratings for `Missing` starling10x.rep2 BUSCO genes:

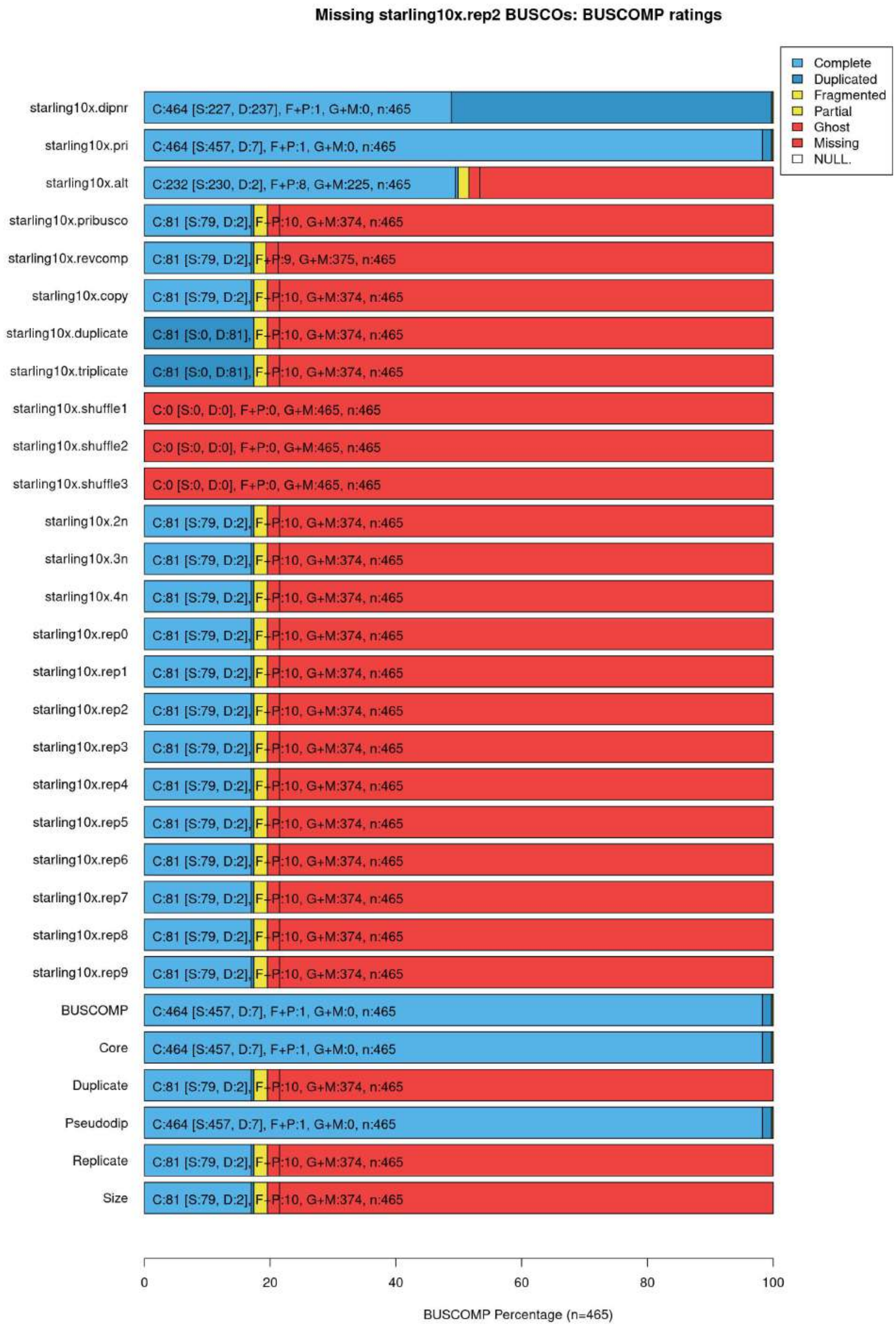

BUSCOMP ratings for `Missing` starling10x.rep2 BUSCOMP genes:

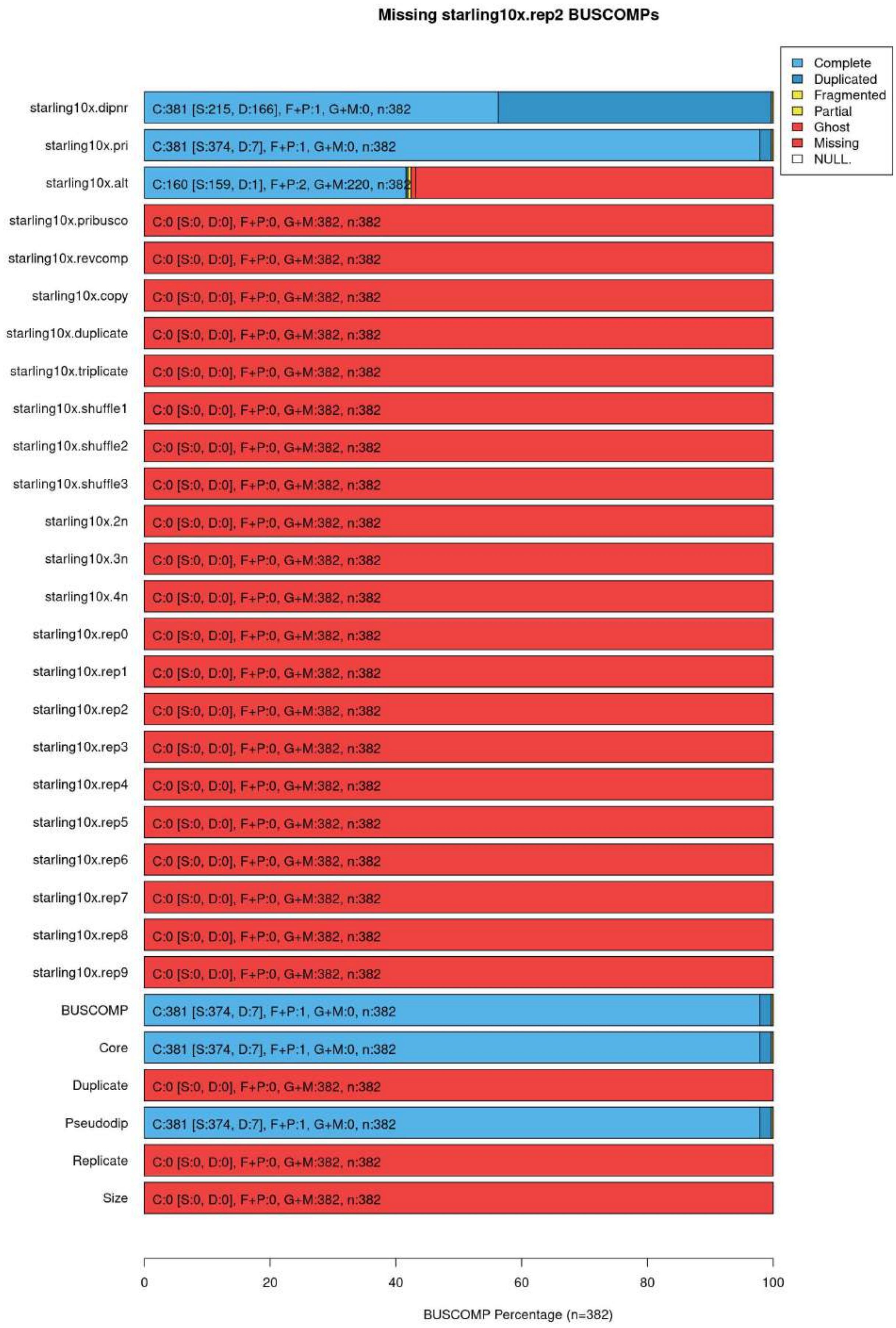

5.25 Missing starling10x.rep3 BUSCO genes

BUSCO ratings for `Missing` starling10x.rep3 BUSCO genes:

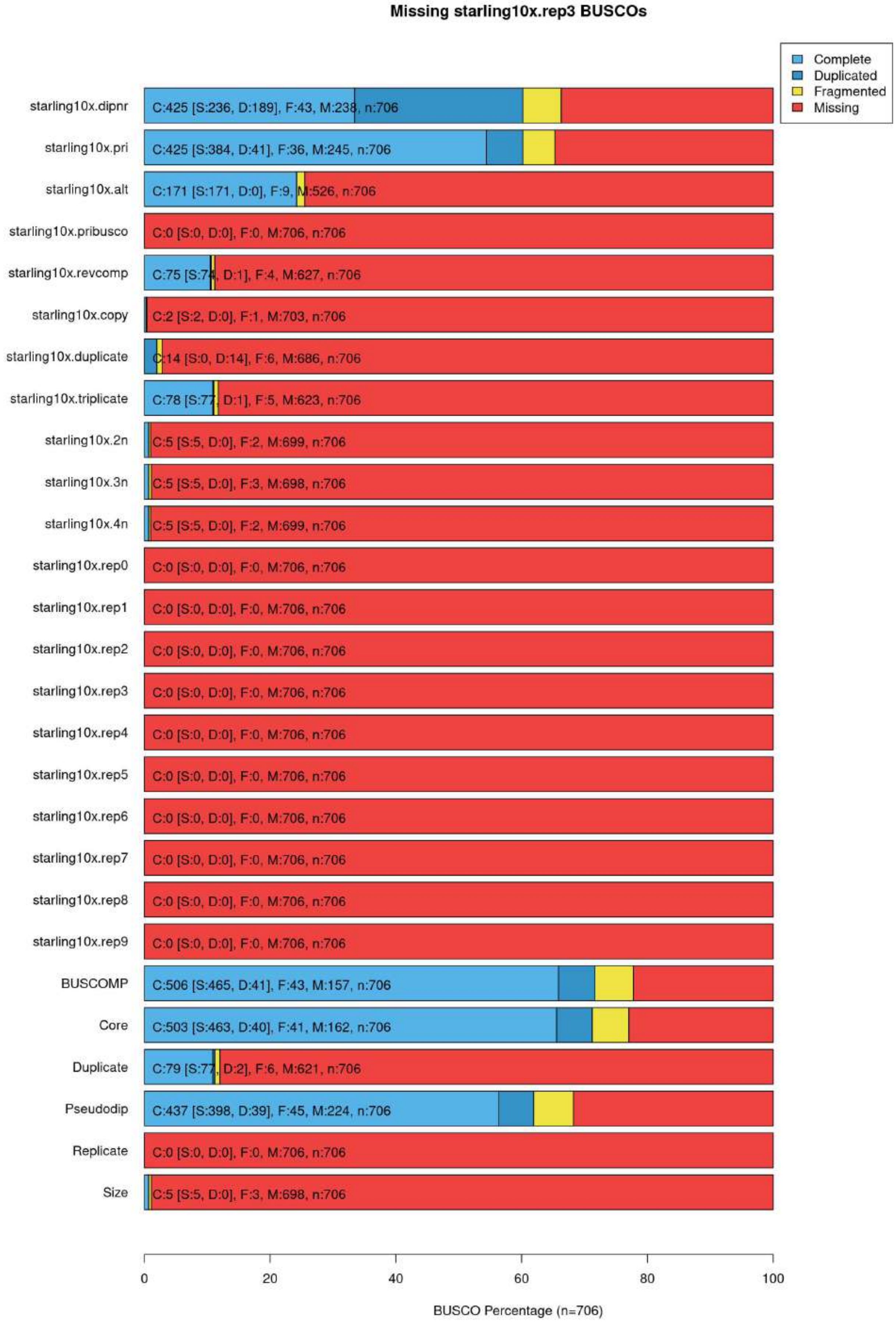

BUSCOMP ratings for `Missing` starling10x.rep3 BUSCO genes:

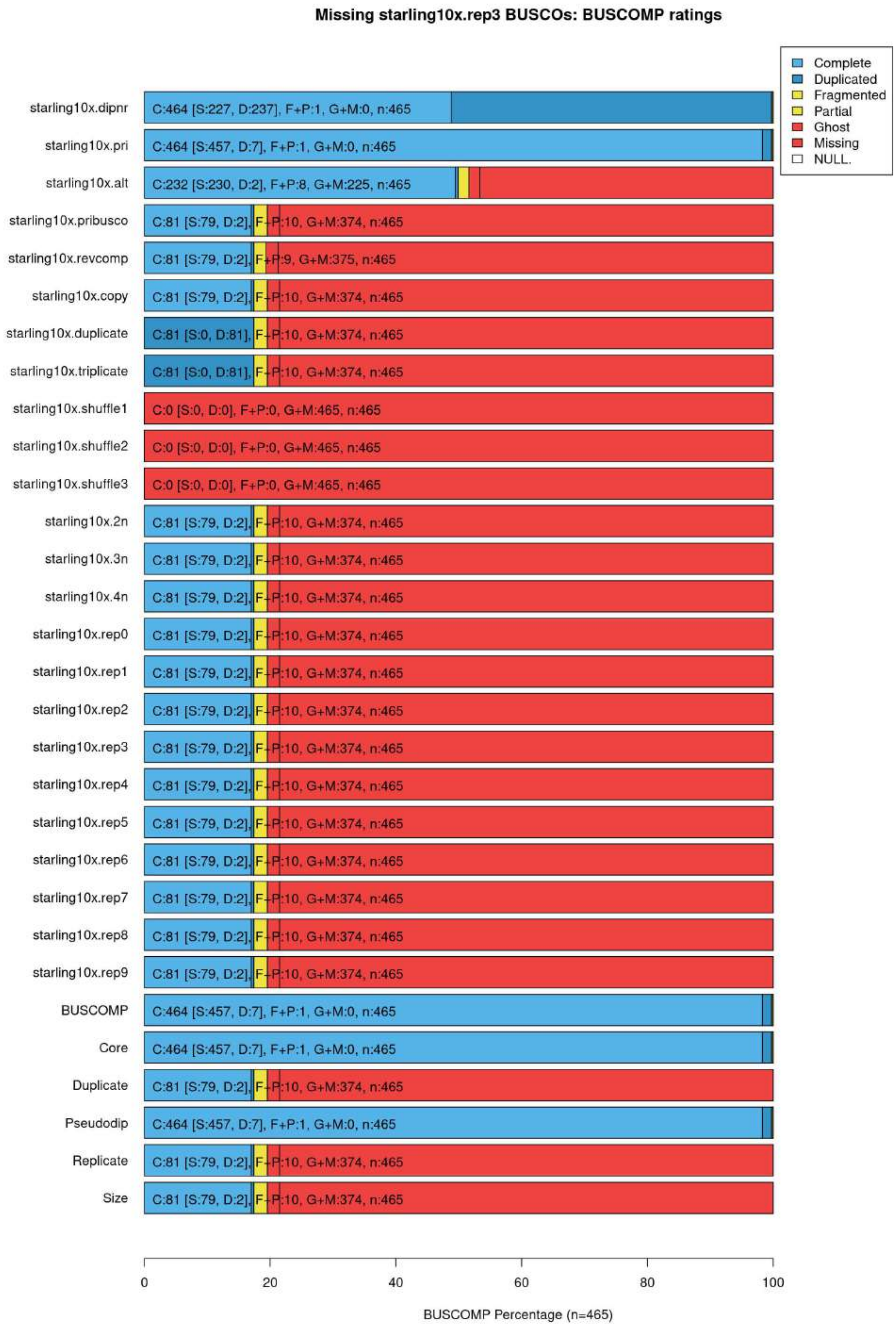

BUSCOMP ratings for `Missing` starling10x.rep3 BUSCOMP genes:

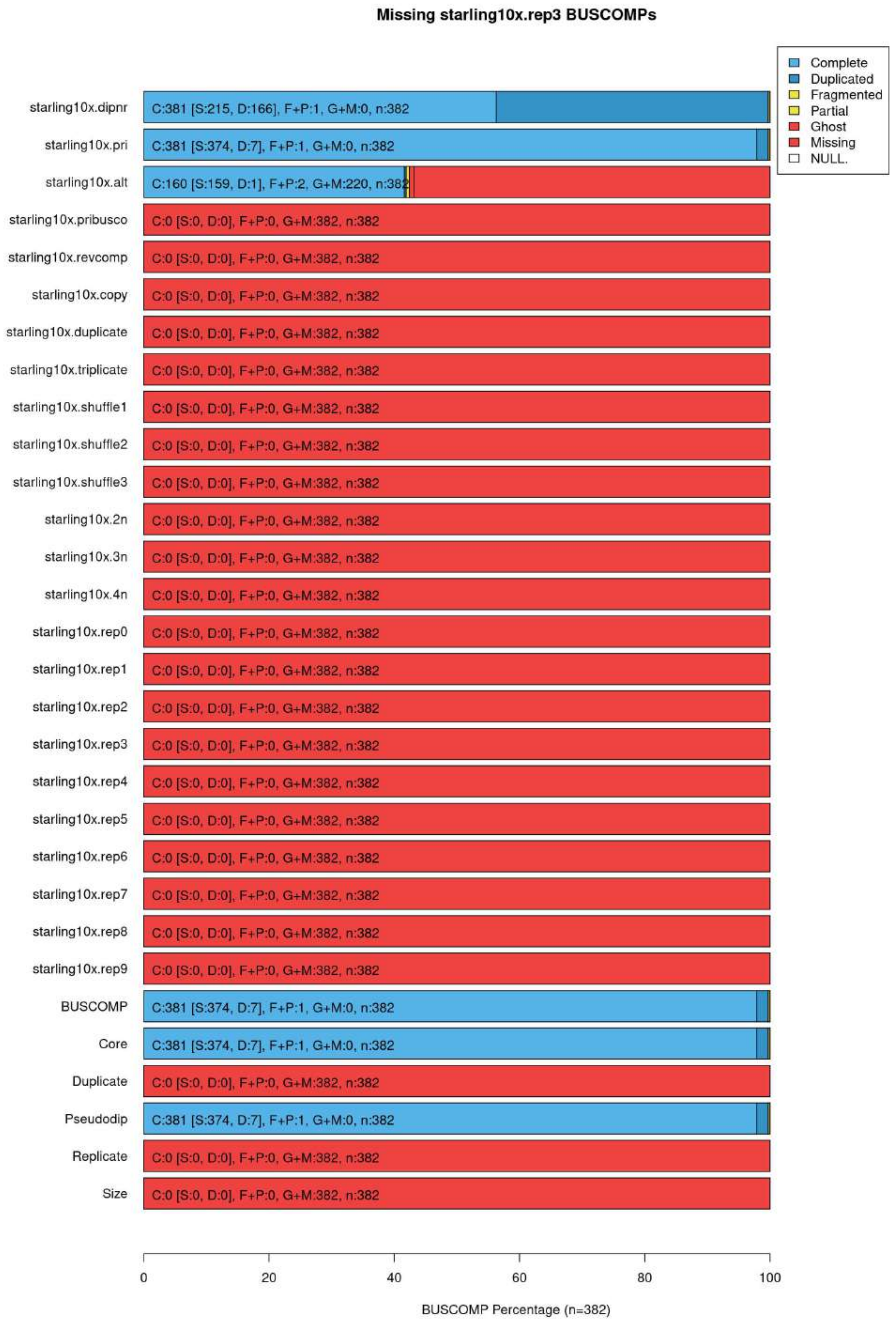

5.26 Missing starling10x.rep4 BUSCO genes

BUSCO ratings for `Missing` starling10x.rep4 BUSCO genes:

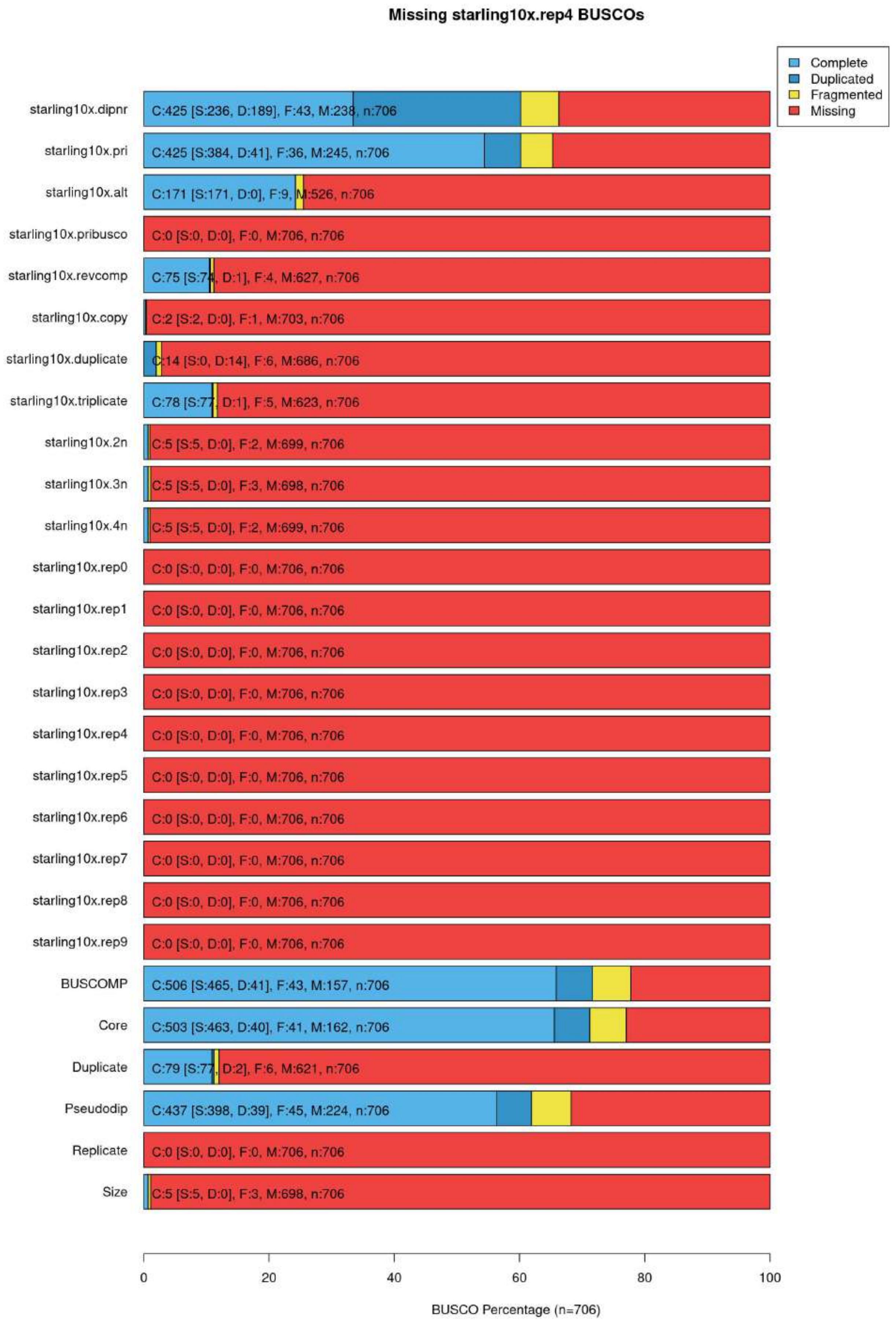

BUSCOMP ratings for `Missing` starling10x.rep4 BUSCO genes:

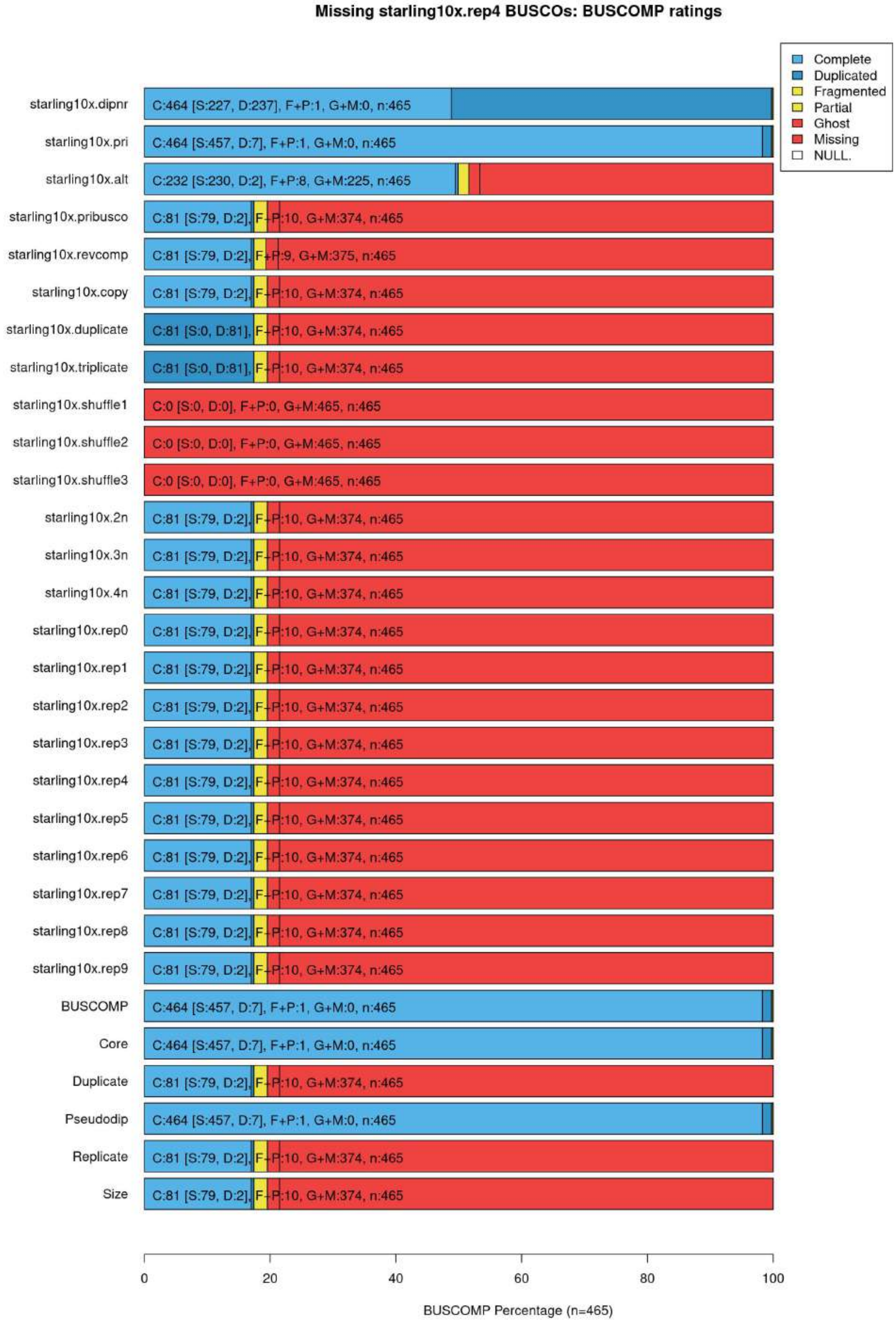

BUSCOMP ratings for `Missing` starling10x.rep4 BUSCOMP genes:

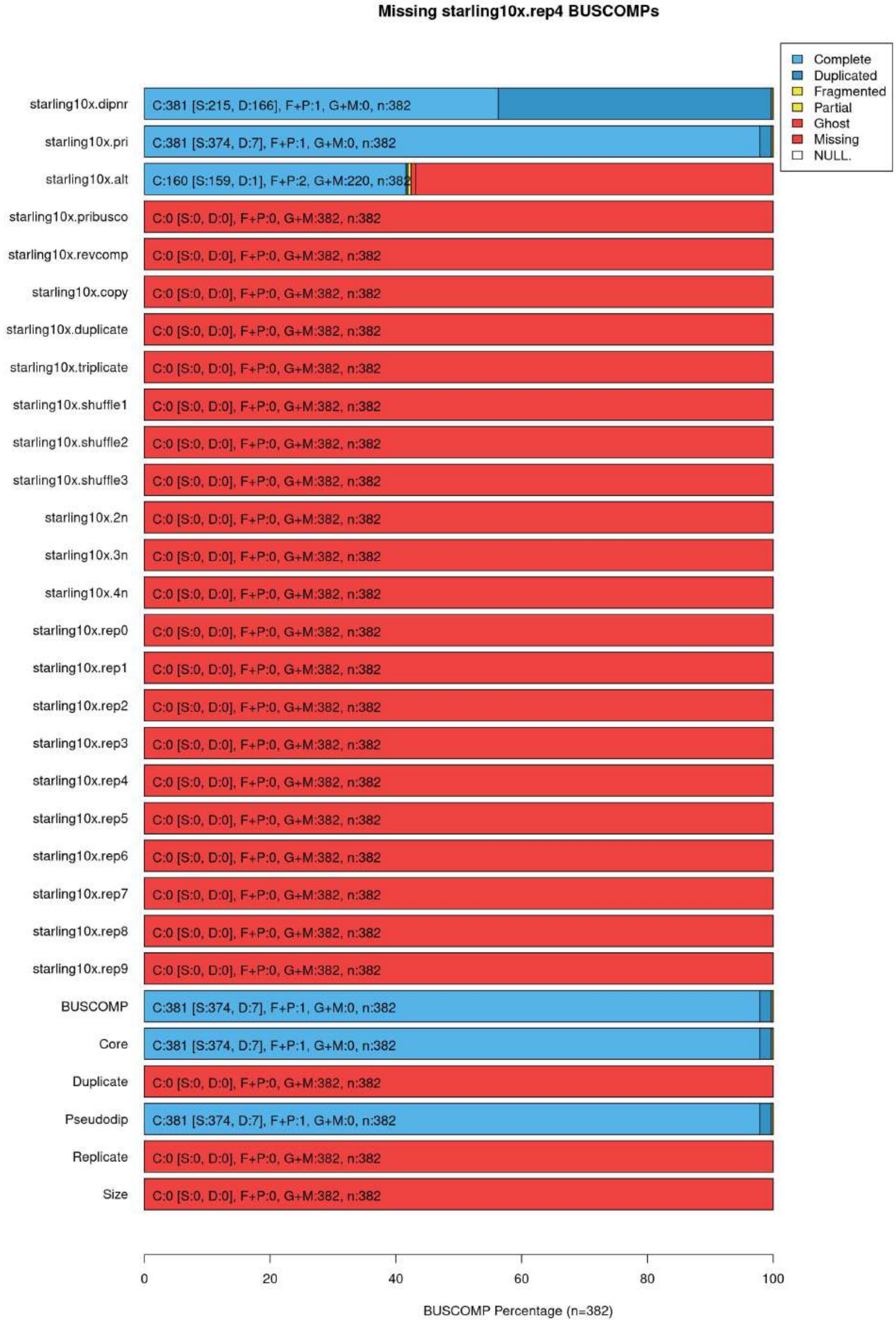

5.27 Missing starling10x.rep5 BUSCO genes

BUSCO ratings for `Missing` starling10x.rep5 BUSCO genes:

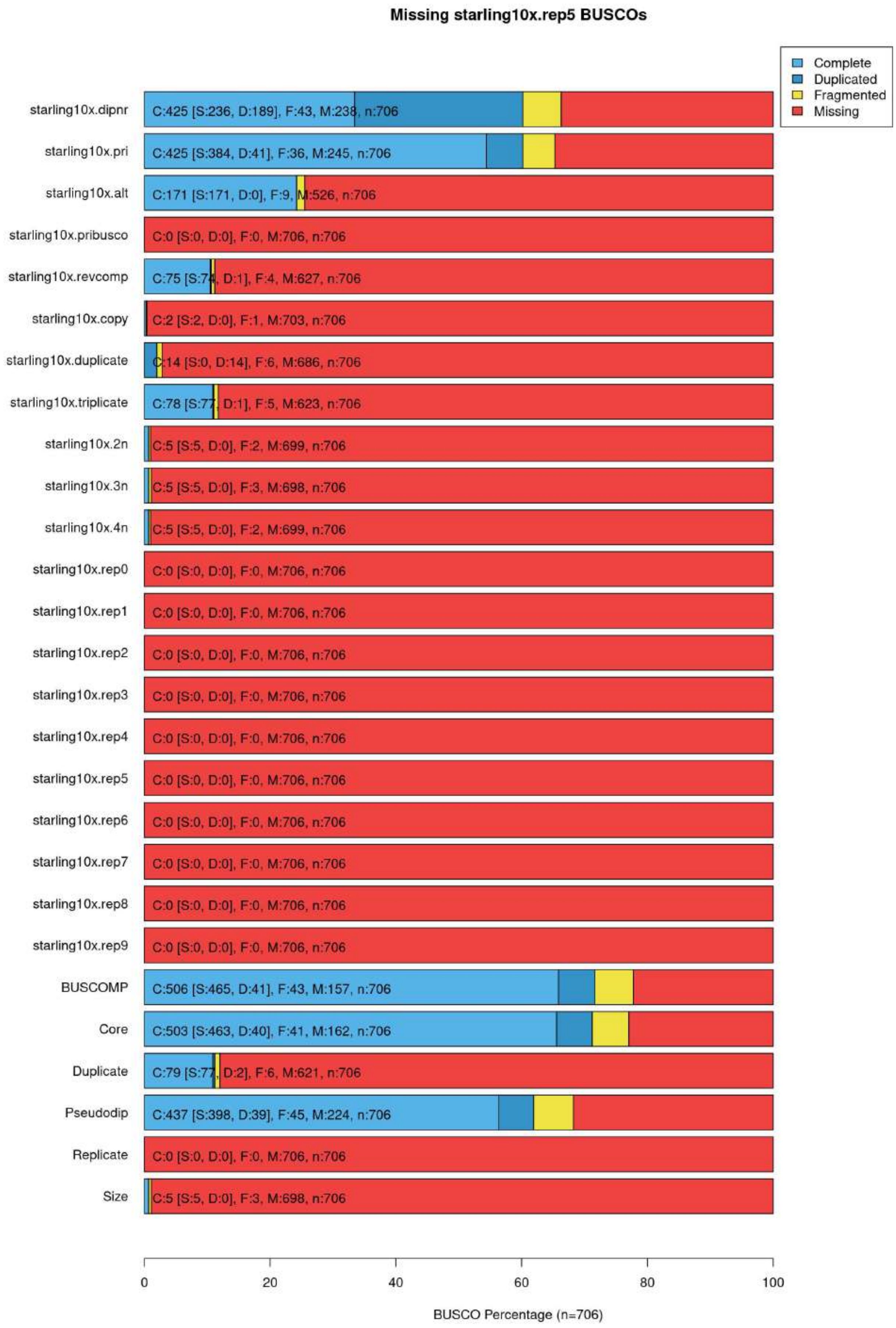

BUSCOMP ratings for `Missing` starling10x.rep5 BUSCO genes:

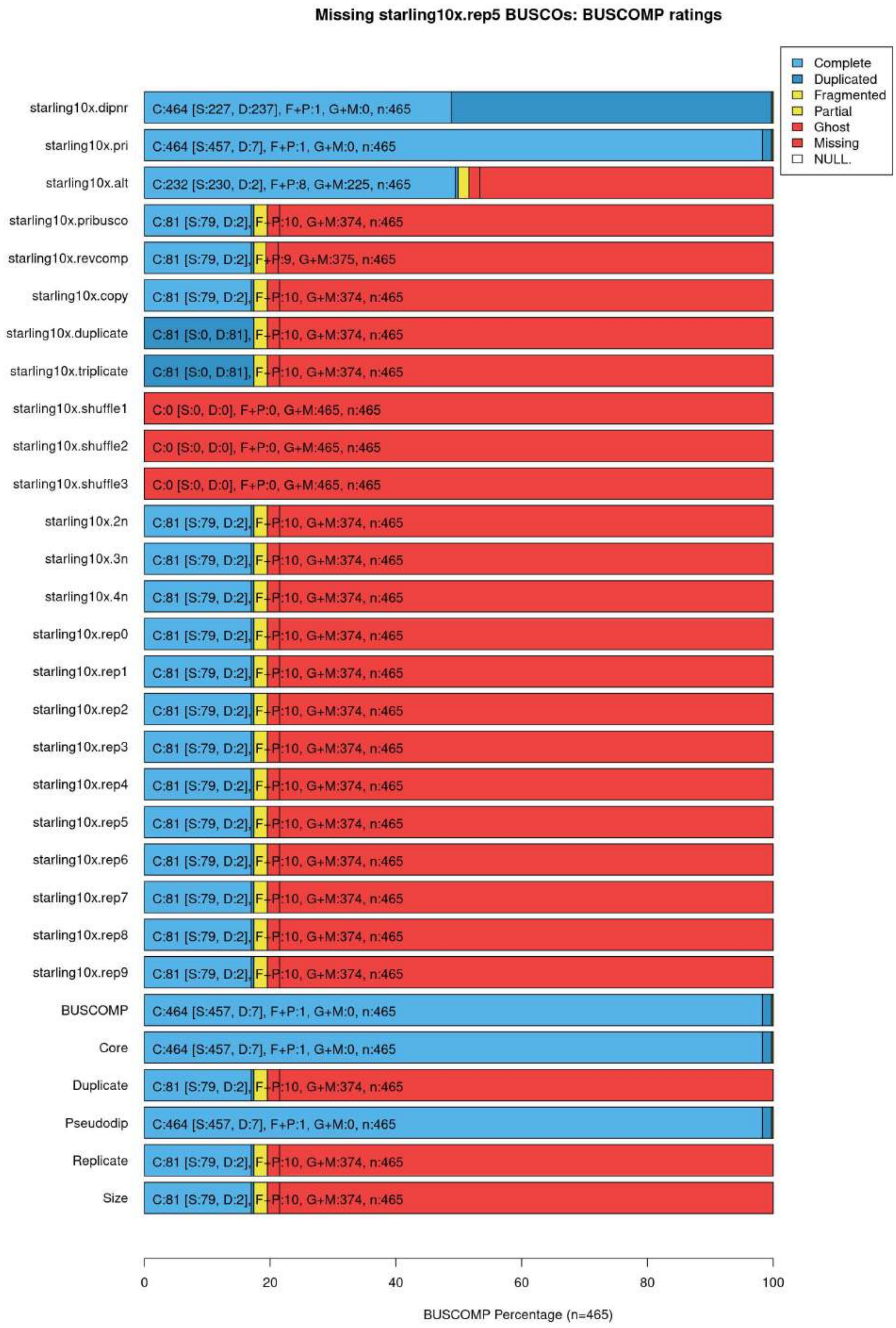

BUSCOMP ratings for `Missing` starling10x.rep5 BUSCOMP genes:

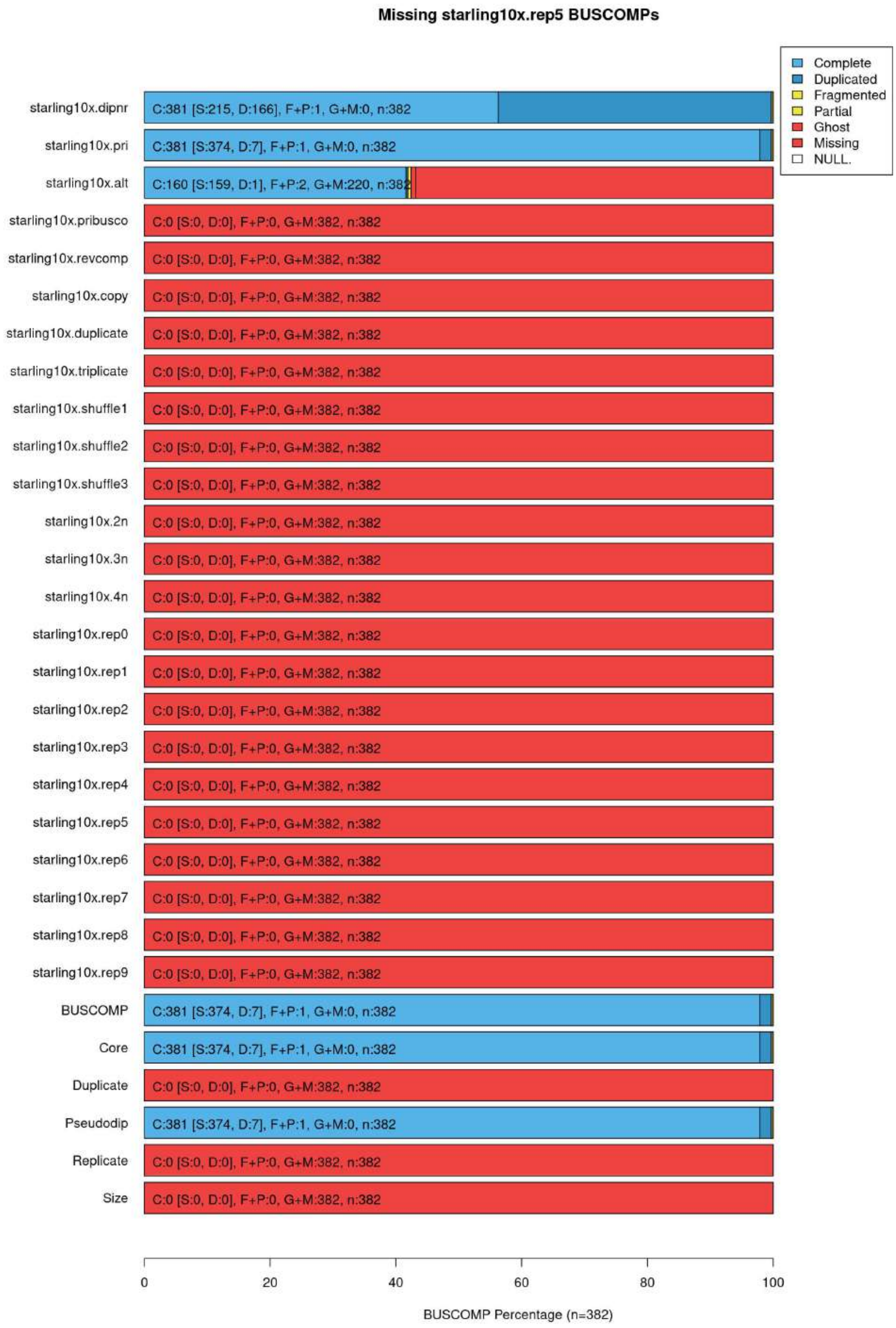

5.28 Missing starling10x.rep6 BUSCO genes

BUSCO ratings for `Missing` starling10x.rep6 BUSCO genes:

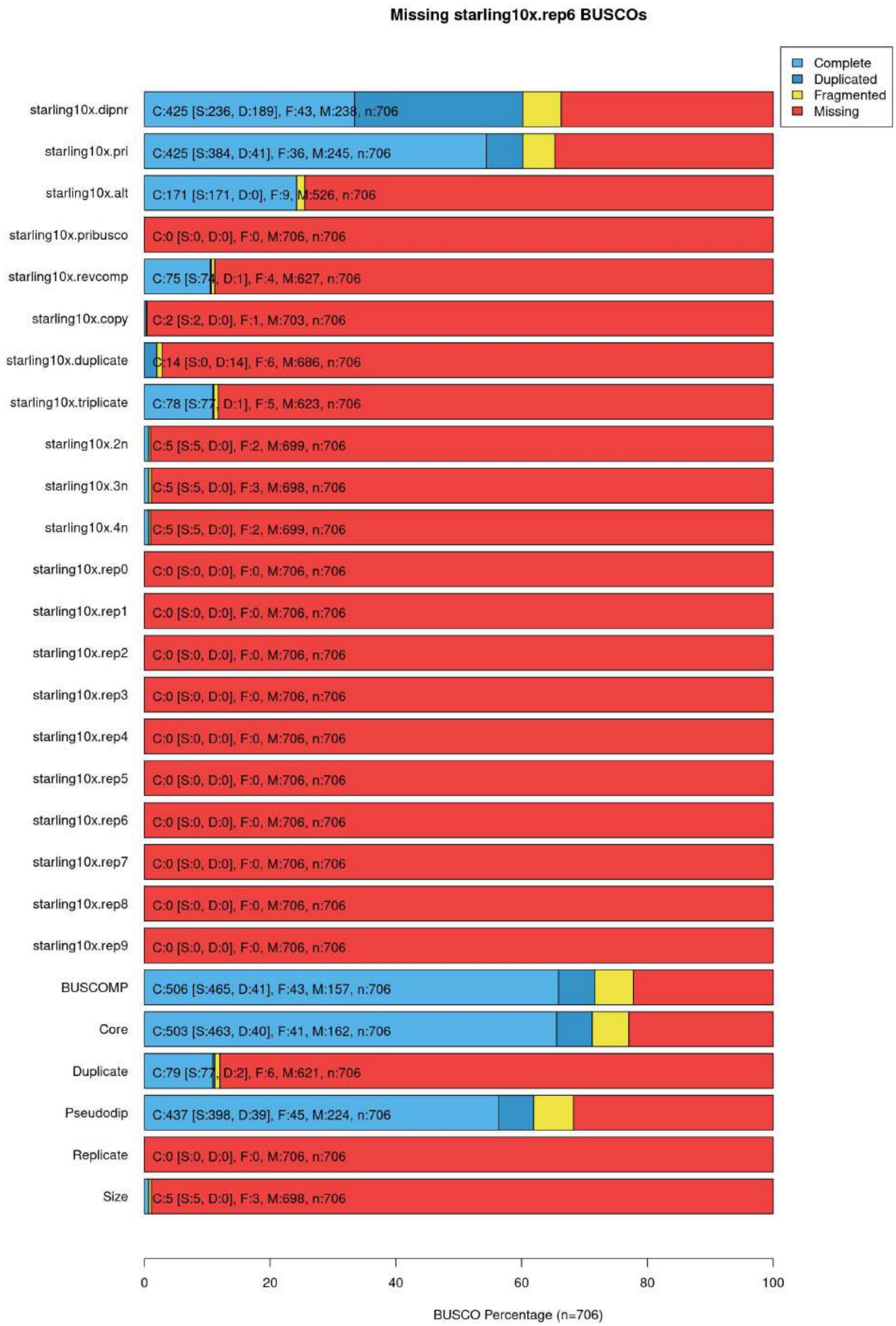

BUSCOMP ratings for `Missing` starling10x.rep6 BUSCO genes:

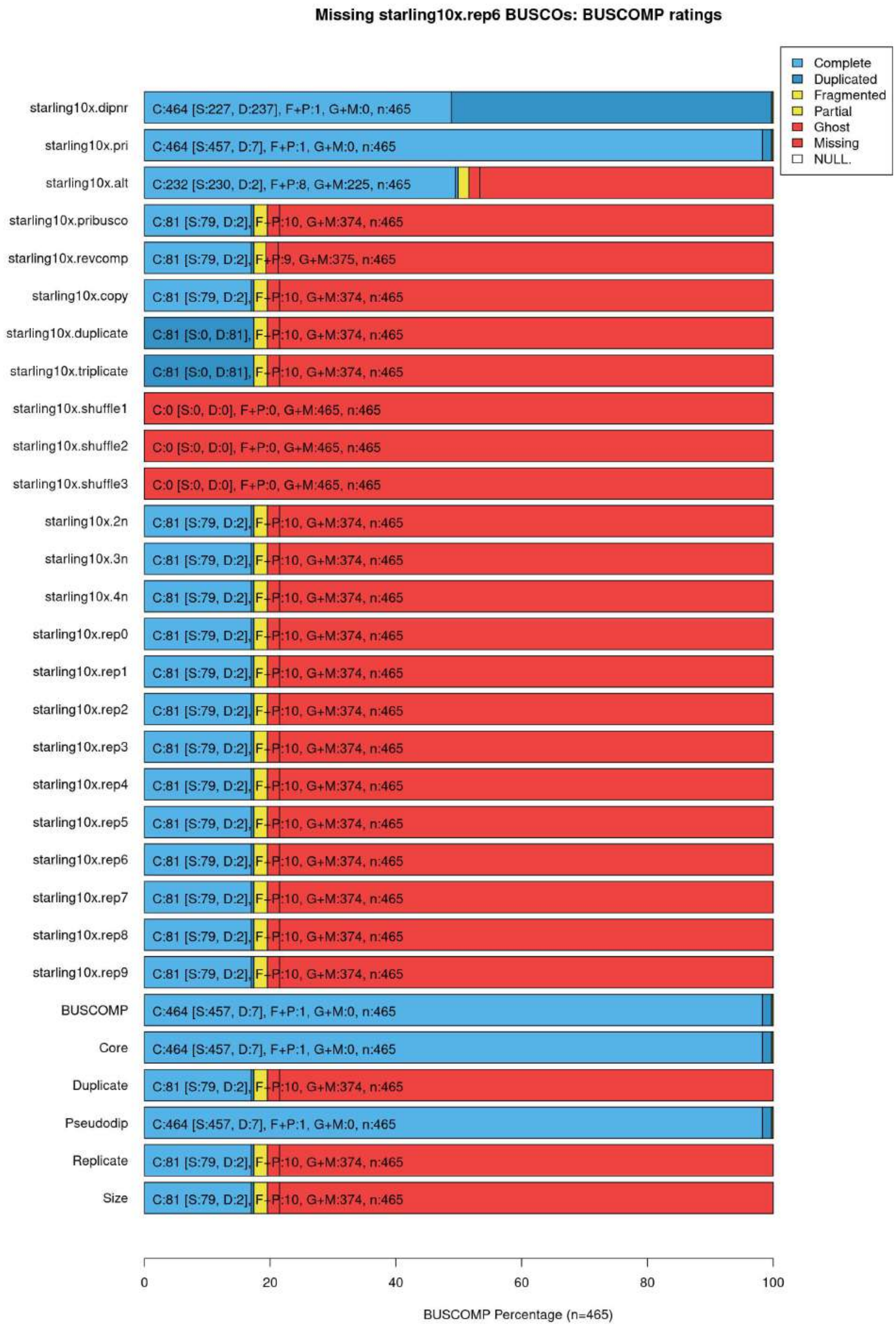

BUSCOMP ratings for `Missing` starling10x.rep6 BUSCOMP genes:

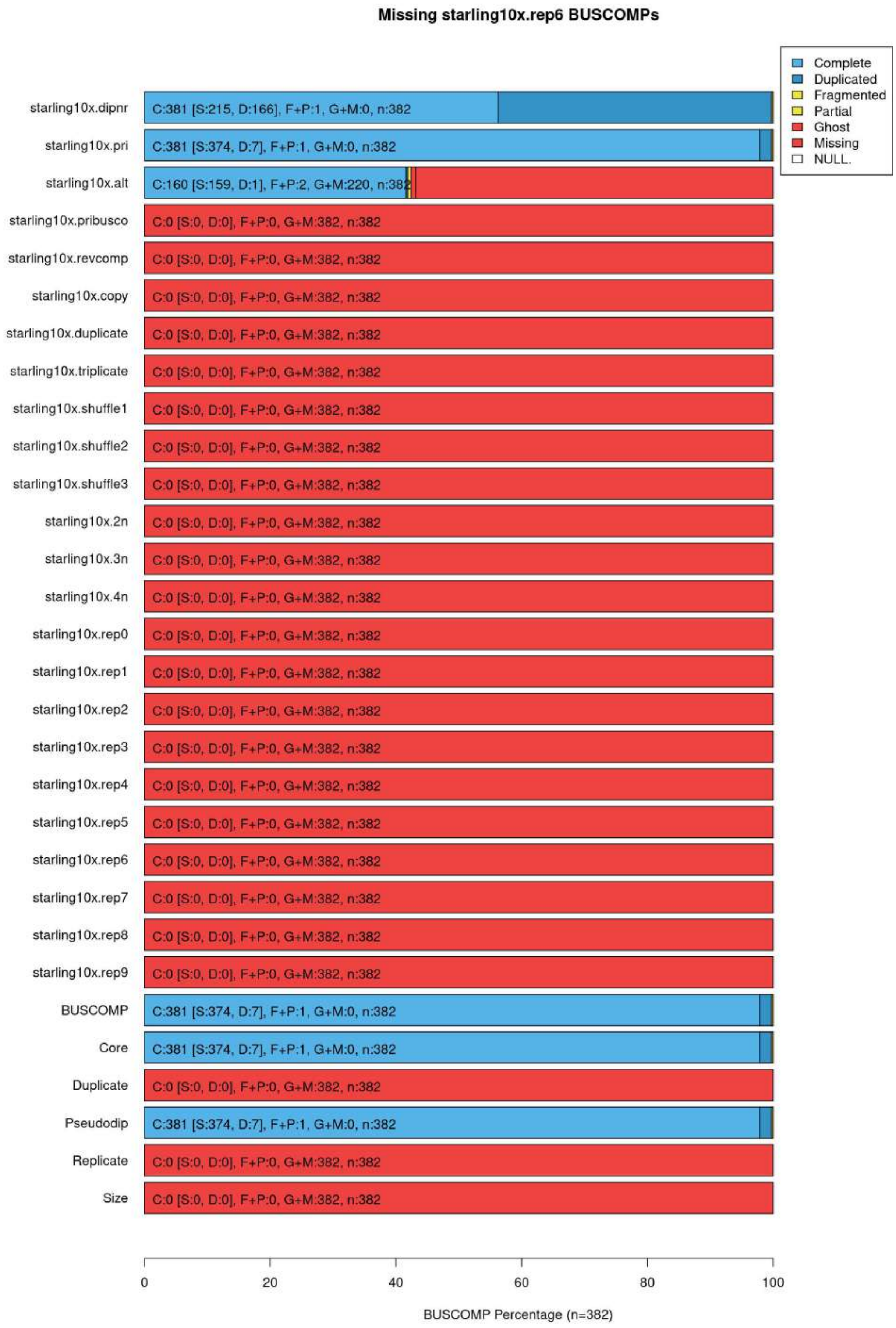

5.29 Missing starling10x.rep7 BUSCO genes

BUSCO ratings for `Missing` starling10x.rep7 BUSCO genes:

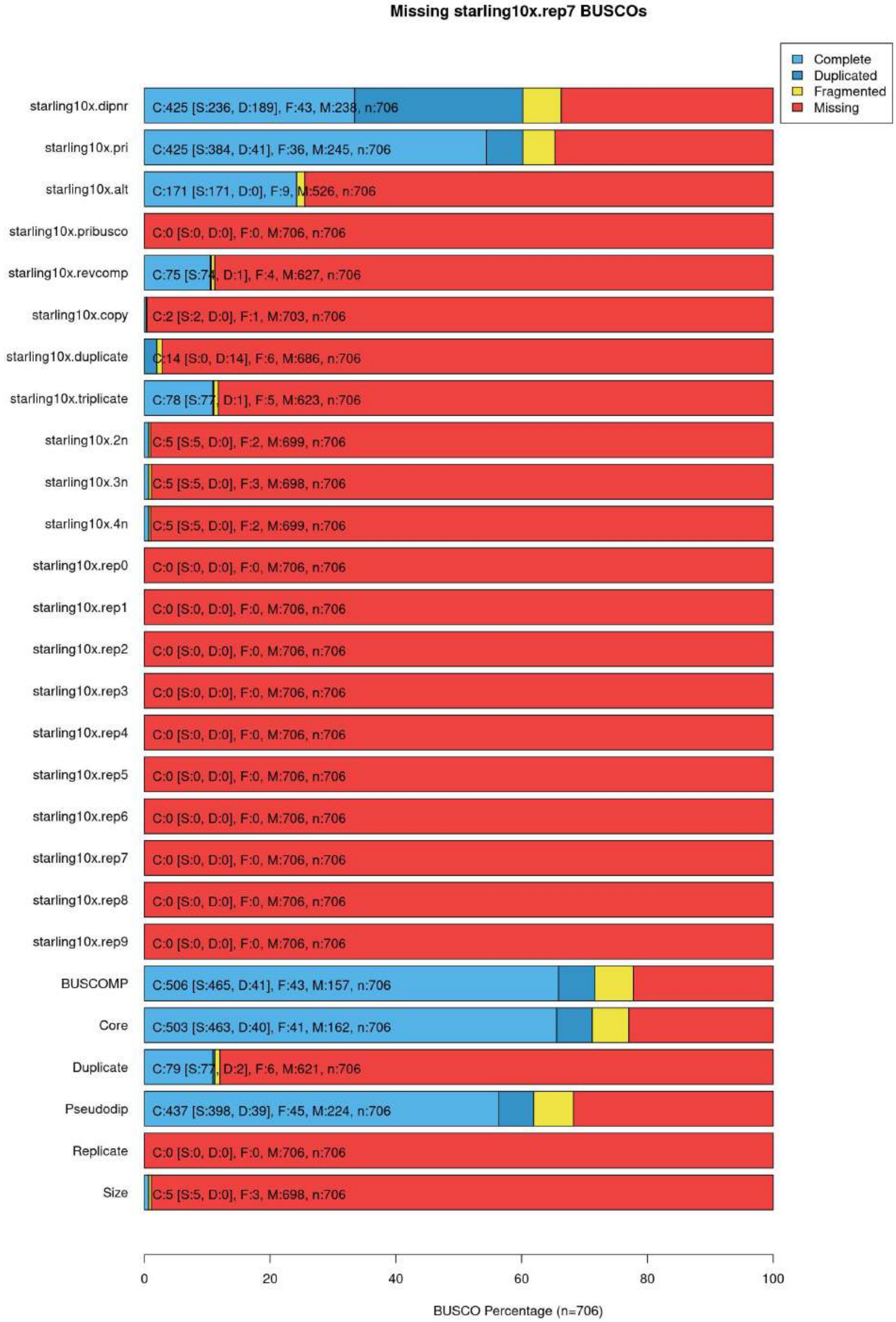

BUSCOMP ratings for `Missing` starling10x.rep7 BUSCO genes:

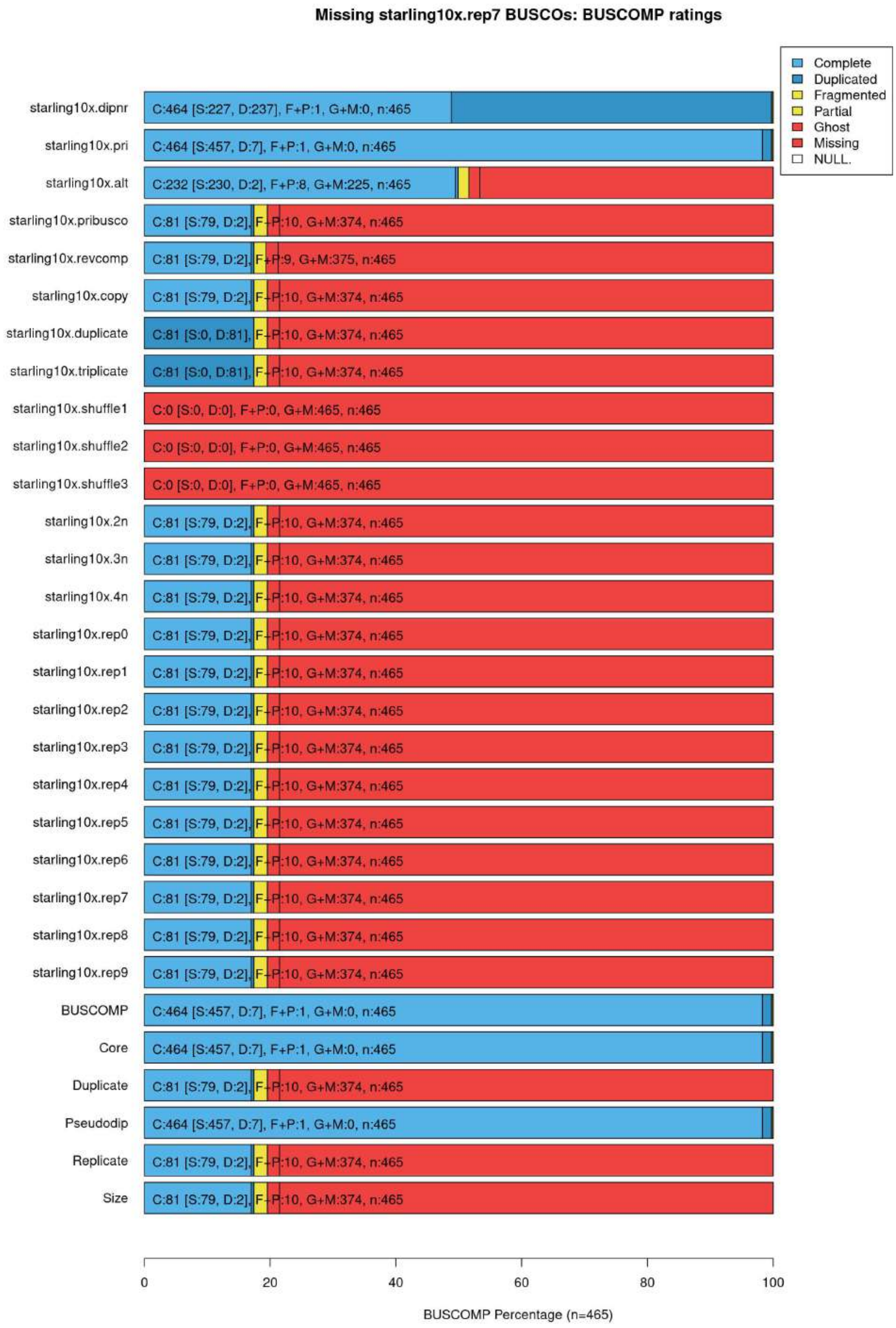

BUSCOMP ratings for `Missing` starling10x.rep7 BUSCOMP genes:

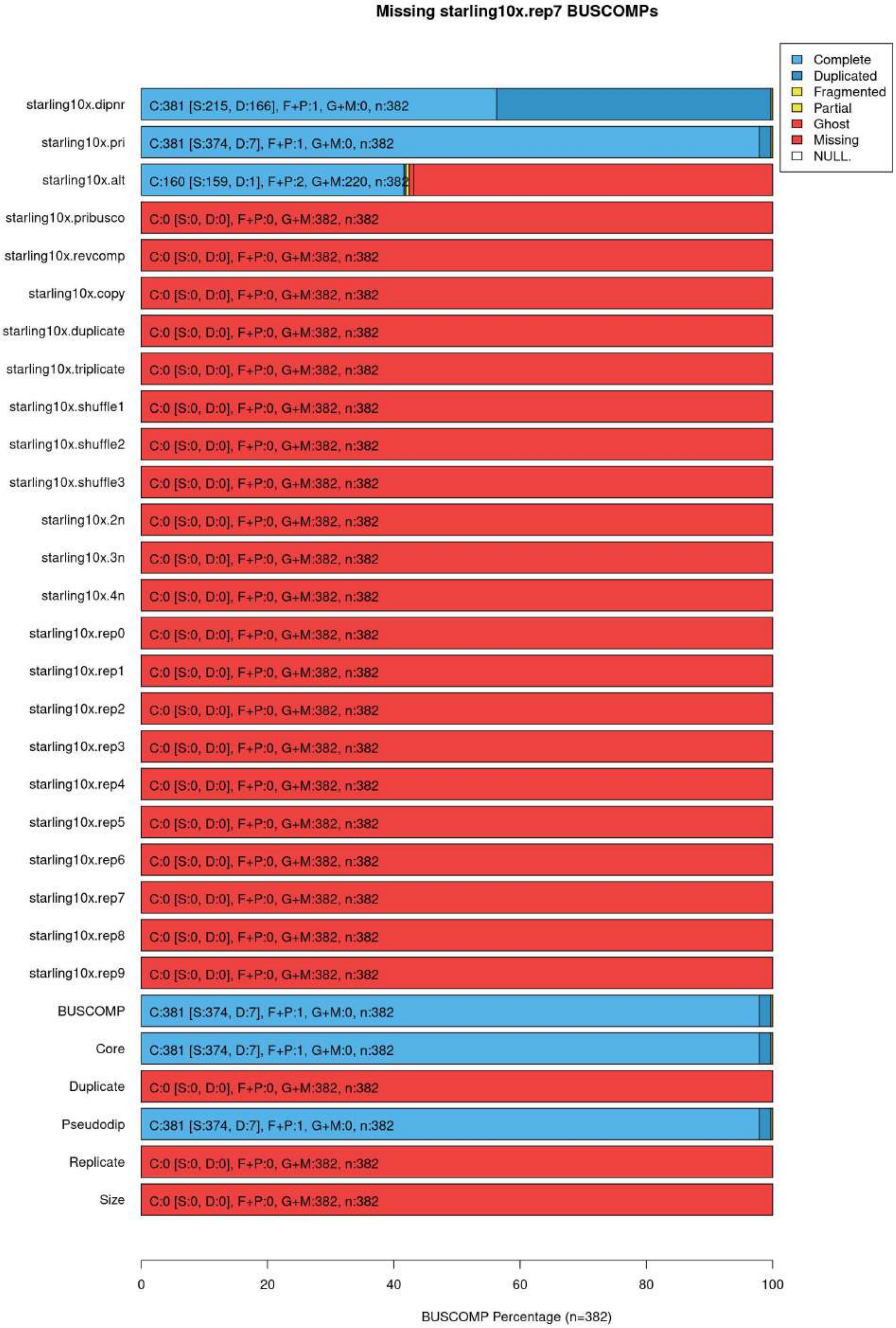

5.30 Missing starling10x.rep8 BUSCO genes

BUSCO ratings for `Missing` starling10x.rep8 BUSCO genes:

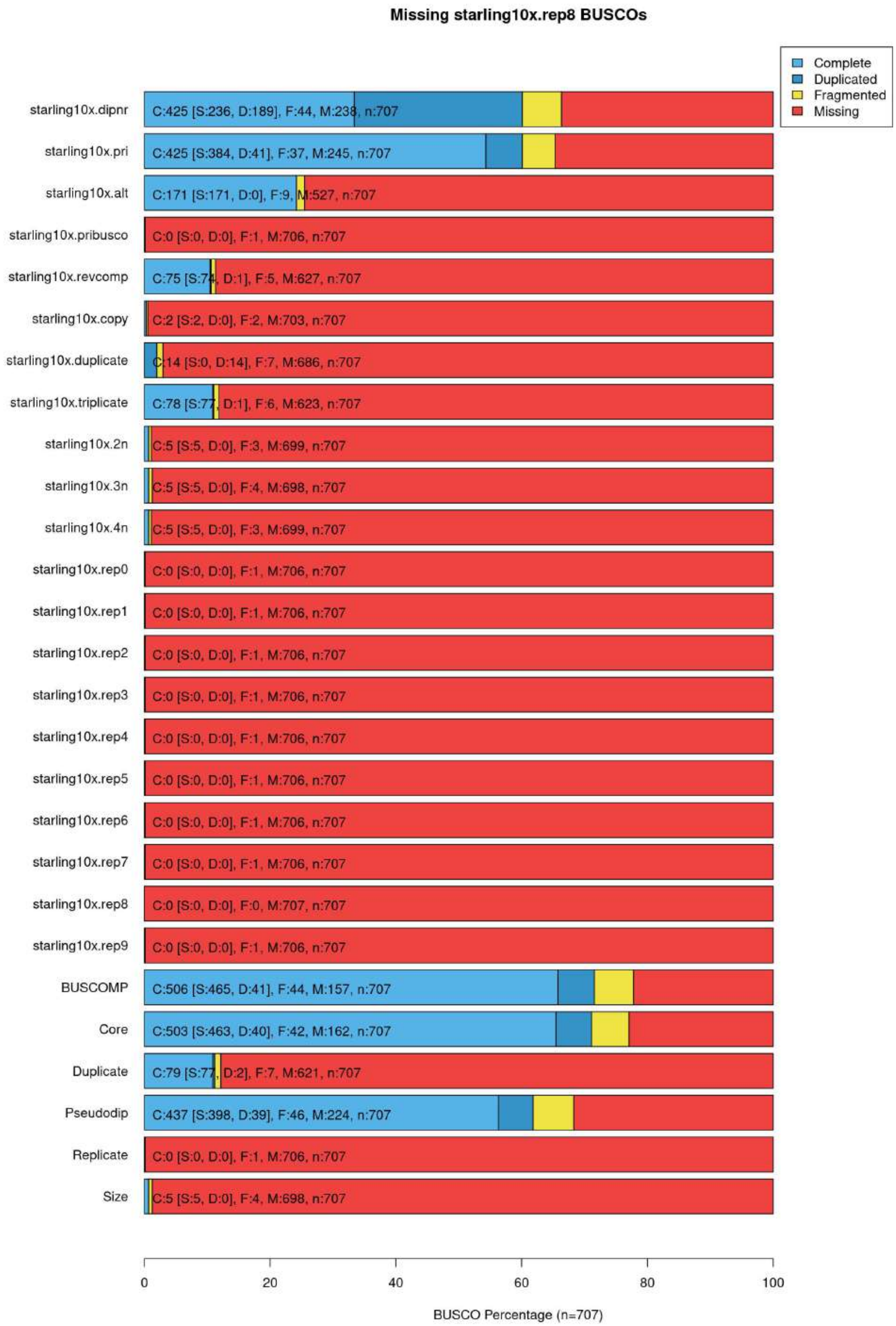

BUSCOMP ratings for `Missing` starling10x.rep8 BUSCO genes:

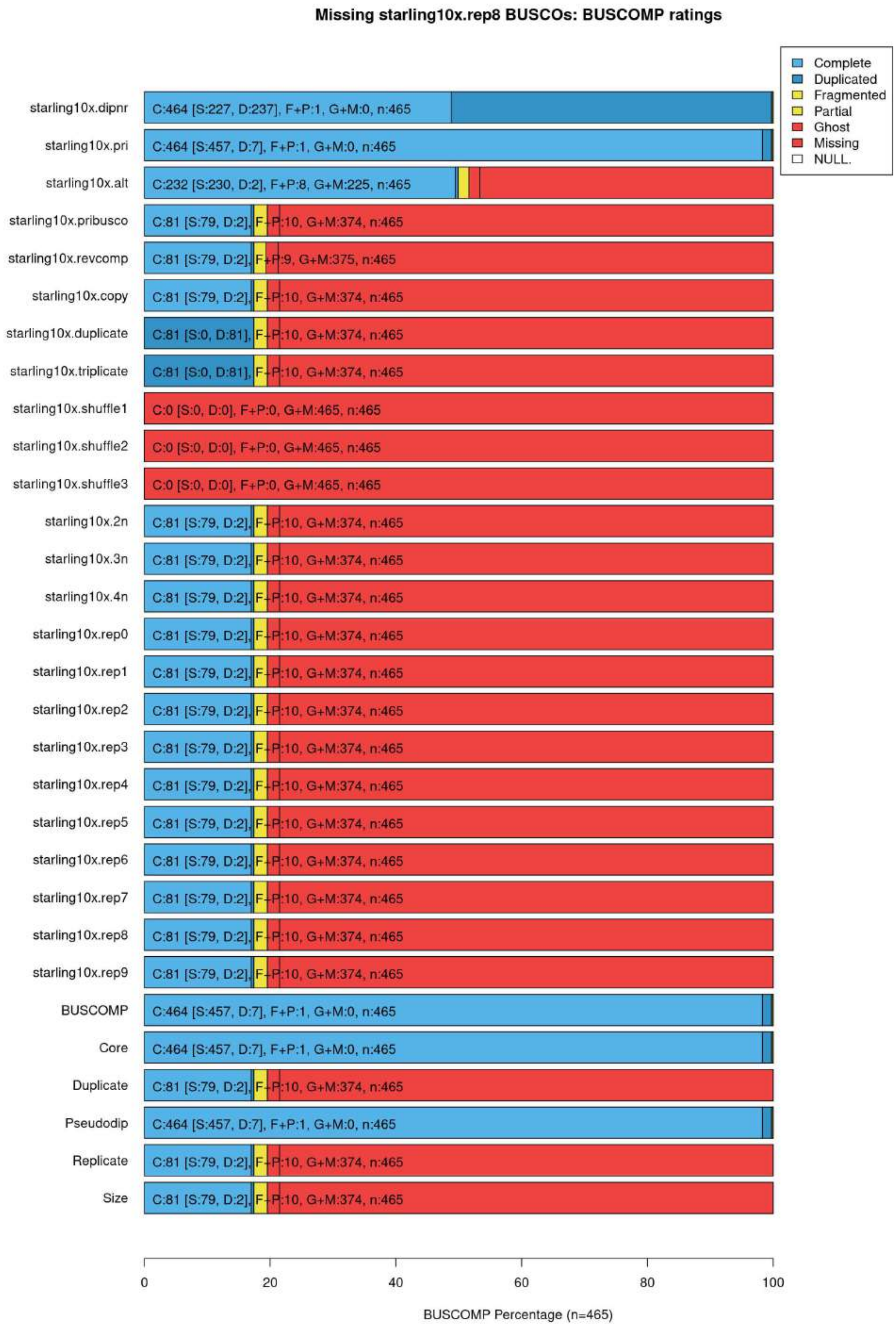

BUSCOMP ratings for `Missing` starling10x.rep8 BUSCOMP genes:

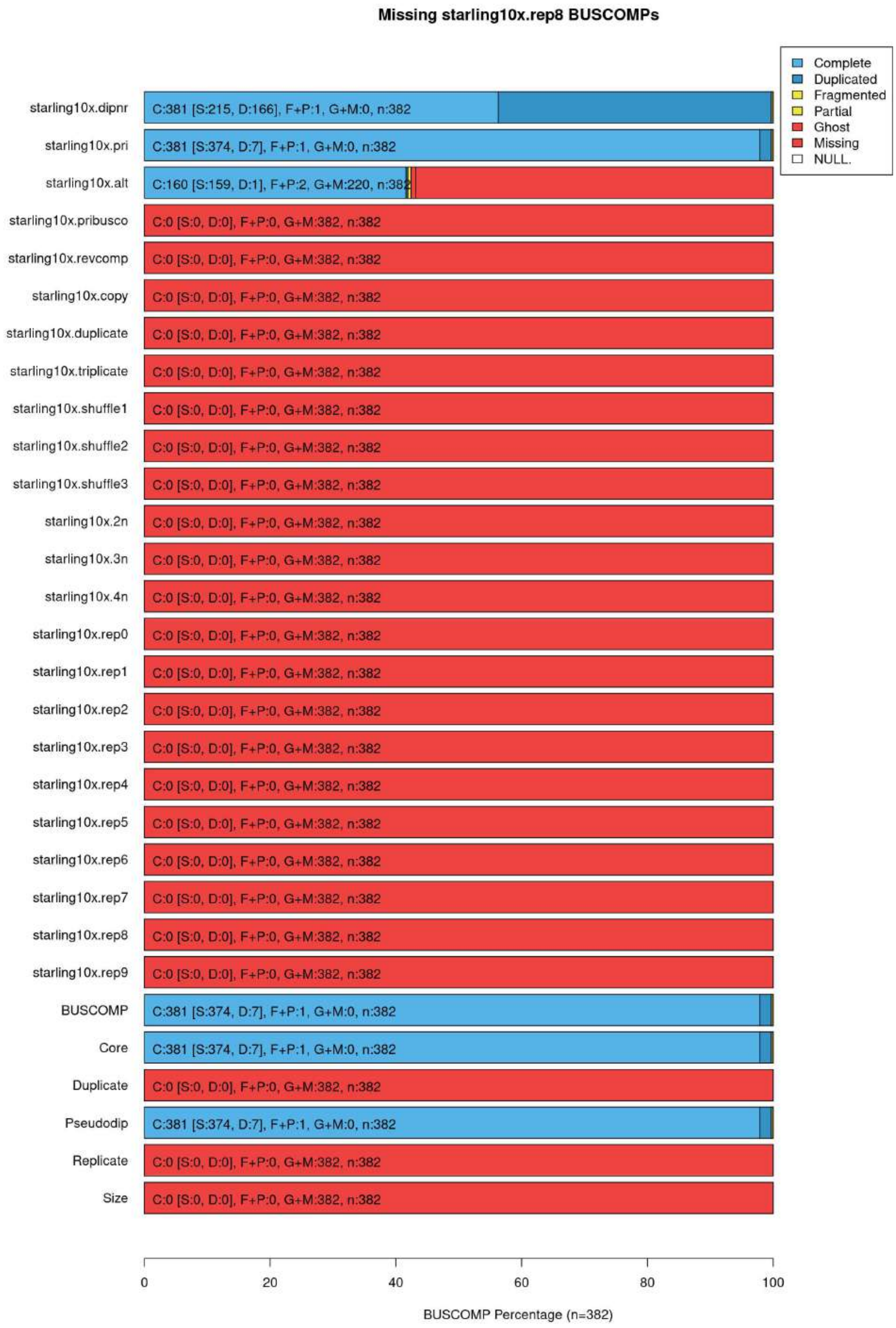

5.31 Missing starling10x.rep9 BUSCO genes

BUSCO ratings for `Missing` starling10x.rep9 BUSCO genes:

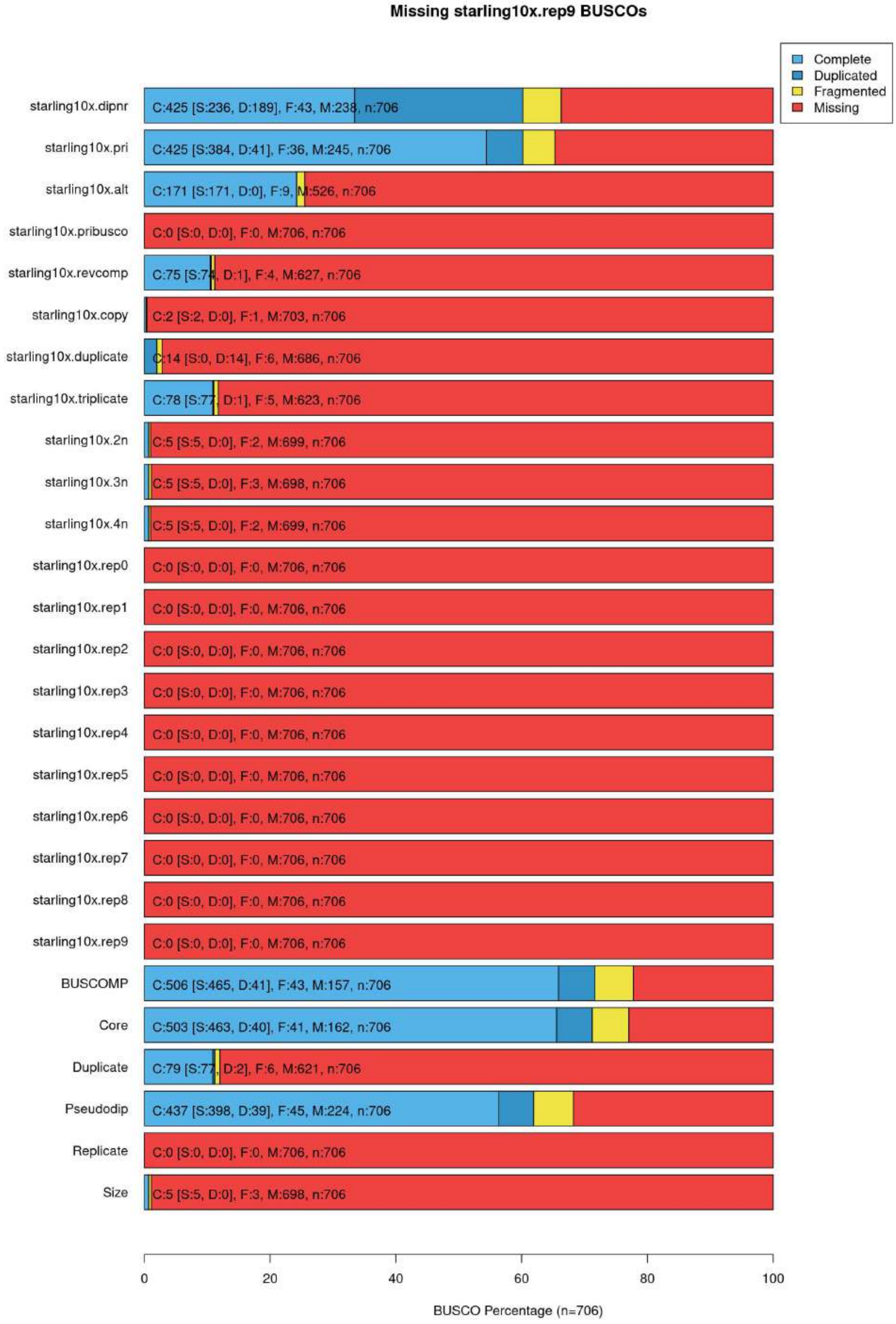

BUSCOMP ratings for `Missing` starling10x.rep9 BUSCO genes:

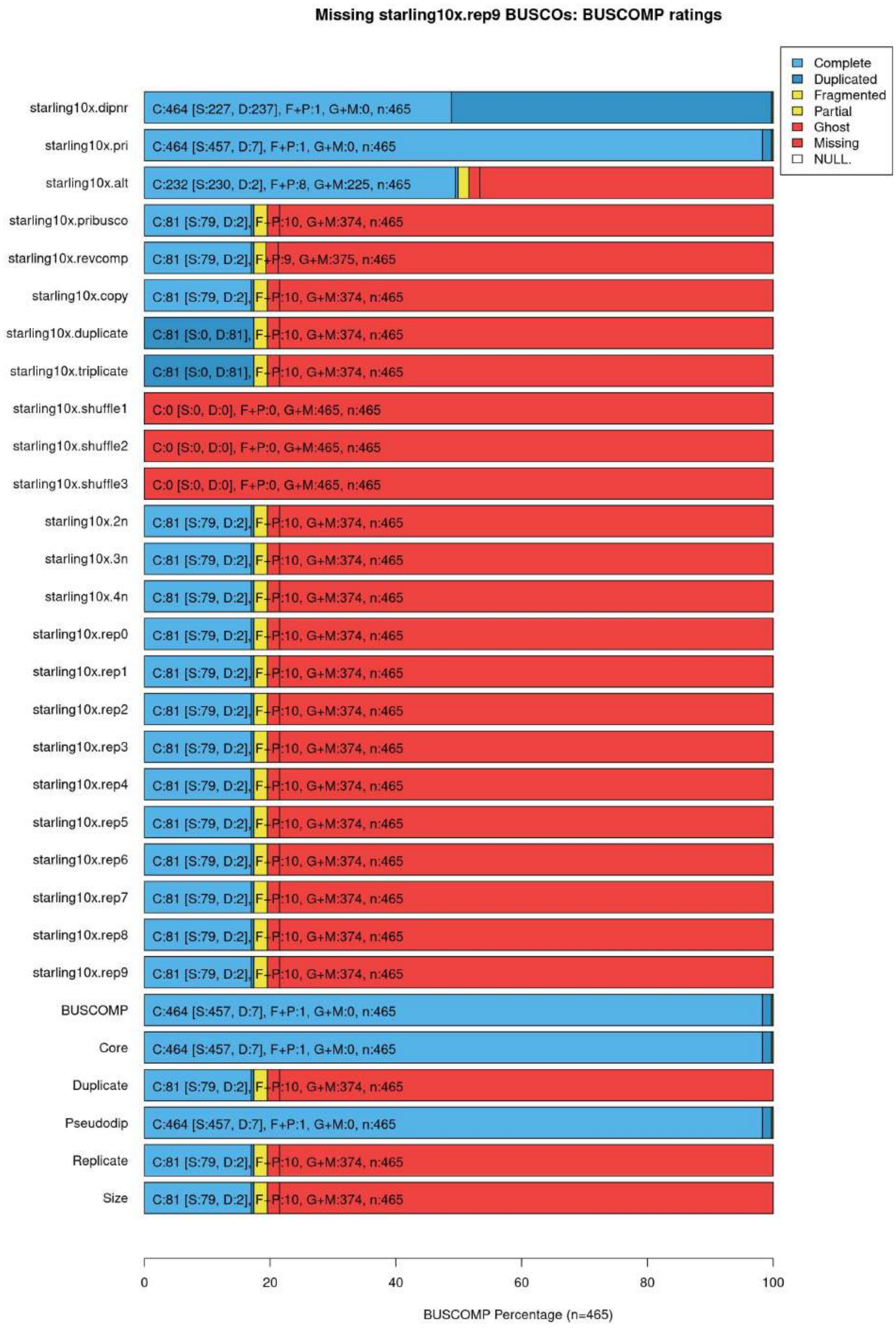

BUSCOMP ratings for `Missing` starling10x.rep9 BUSCOMP genes:

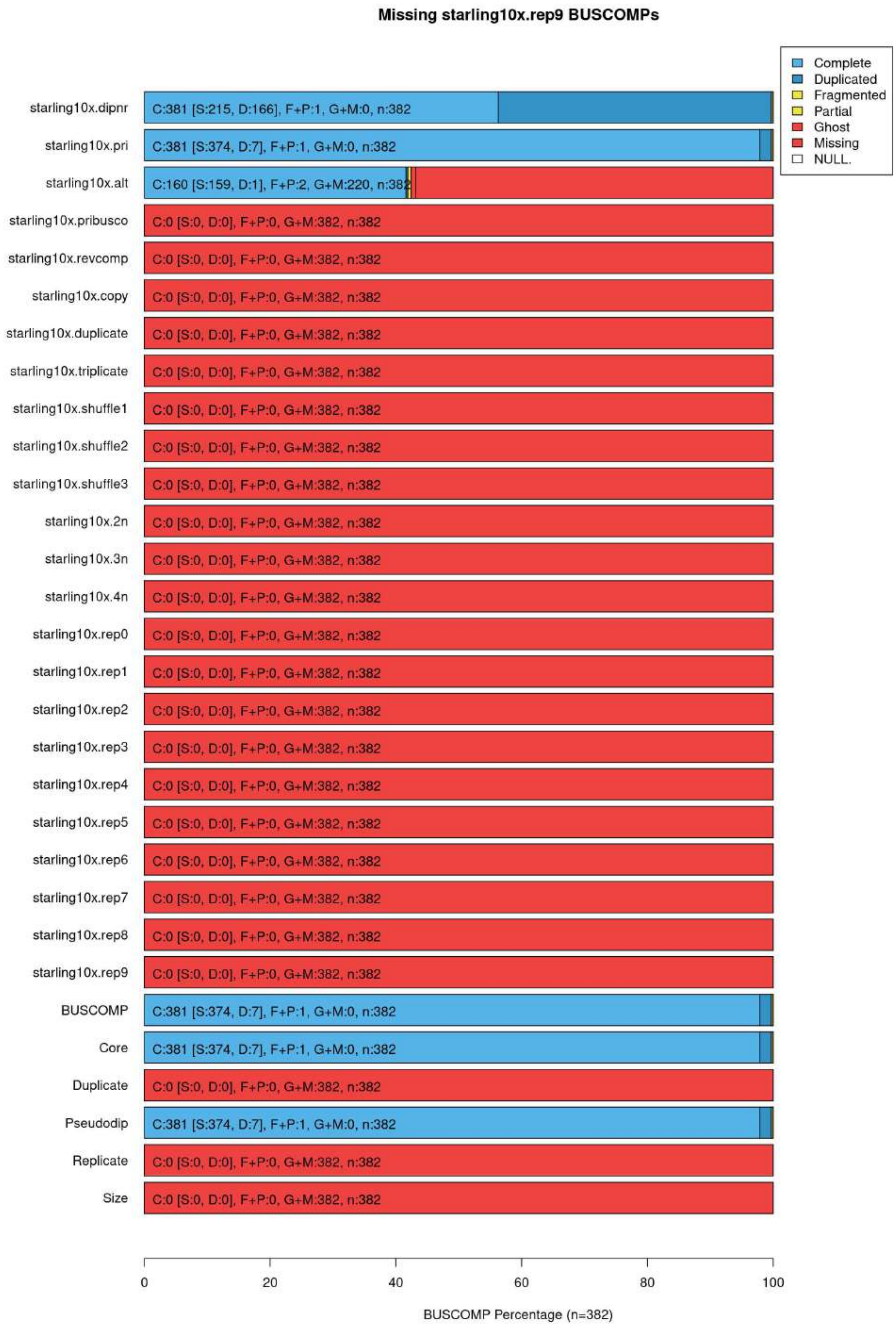

5.32 Missing Replicate BUSCO genes

BUSCO ratings for `Missing` Replicate BUSCO genes:

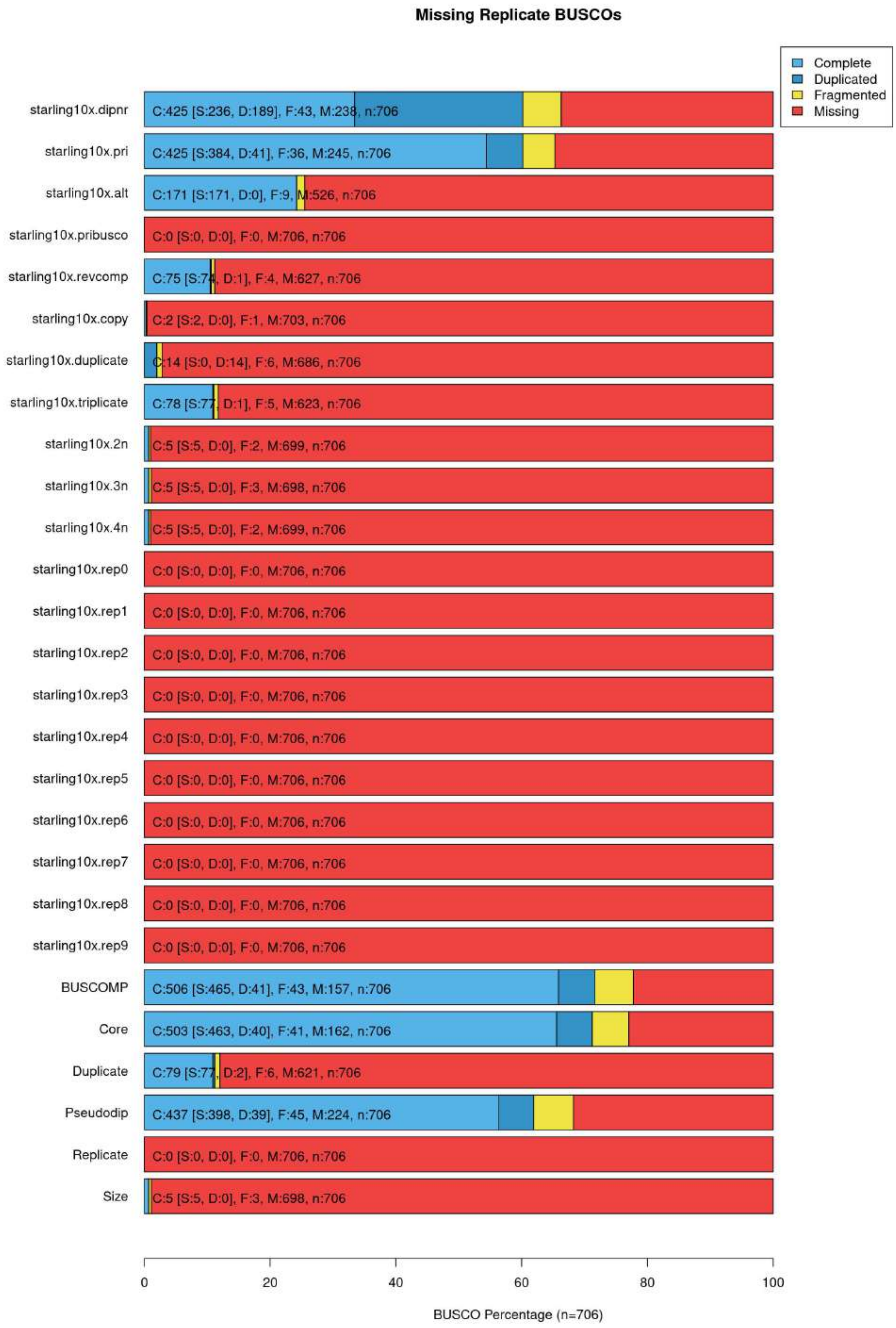

BUSCOMP ratings for `Missing` Replicate BUSCO genes:

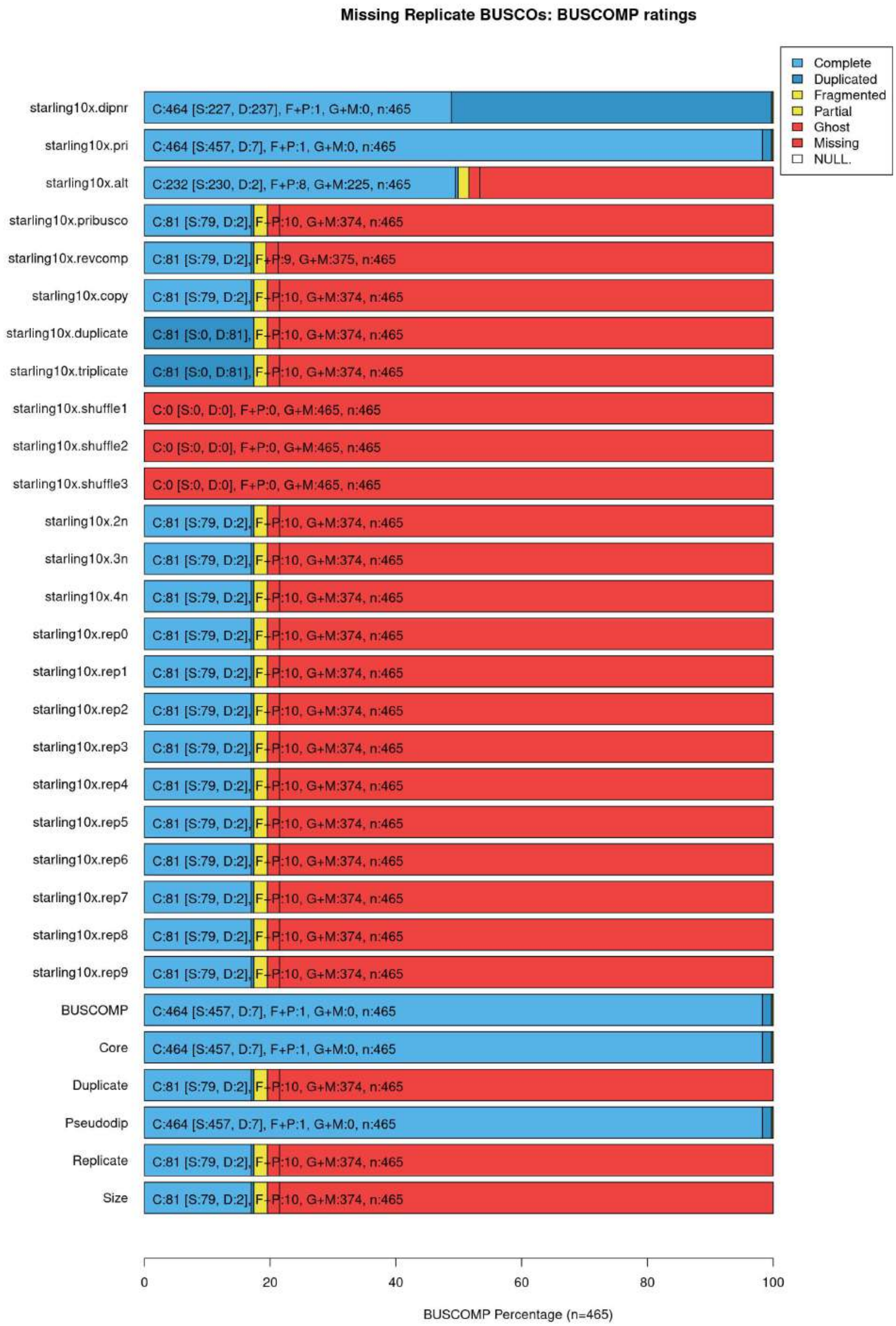

BUSCOMP ratings for `Missing` Replicate BUSCOMP genes:

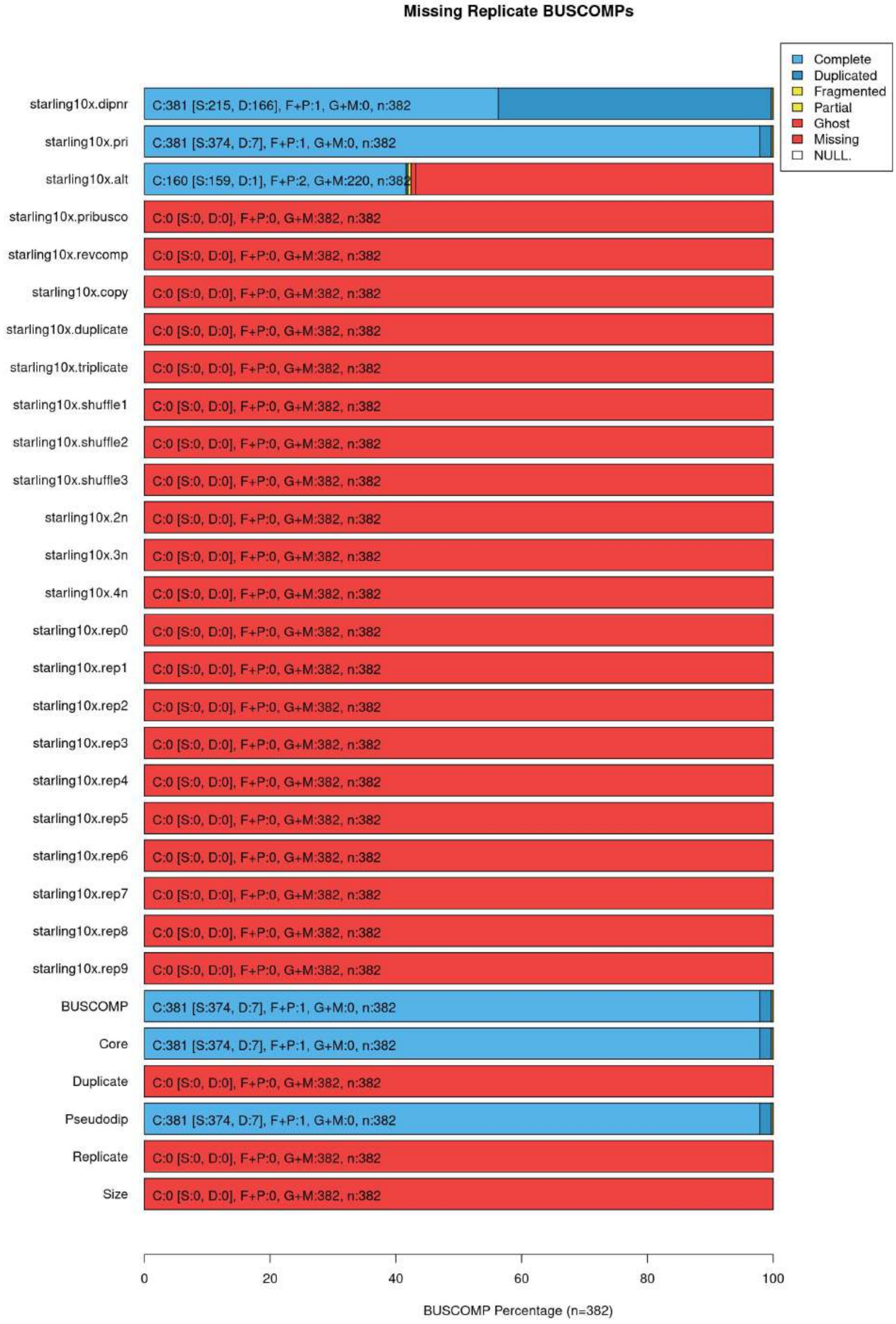

5.33 Missing BUSCOMP BUSCO genes

BUSCO ratings for `Missing` BUSCOMP BUSCO genes:

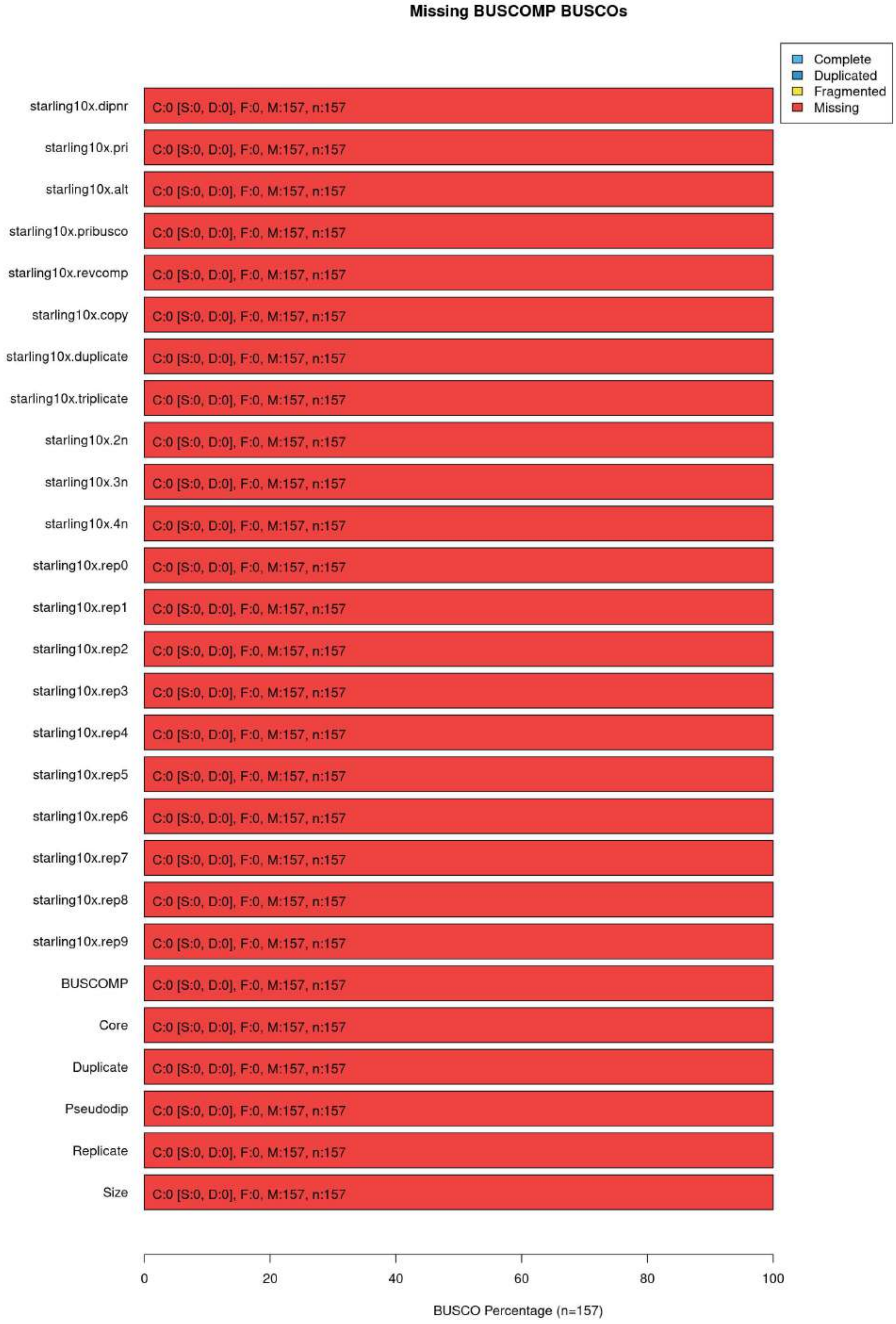

BUSCOMP ratings for `Missing` BUSCOMP BUSCO genes:

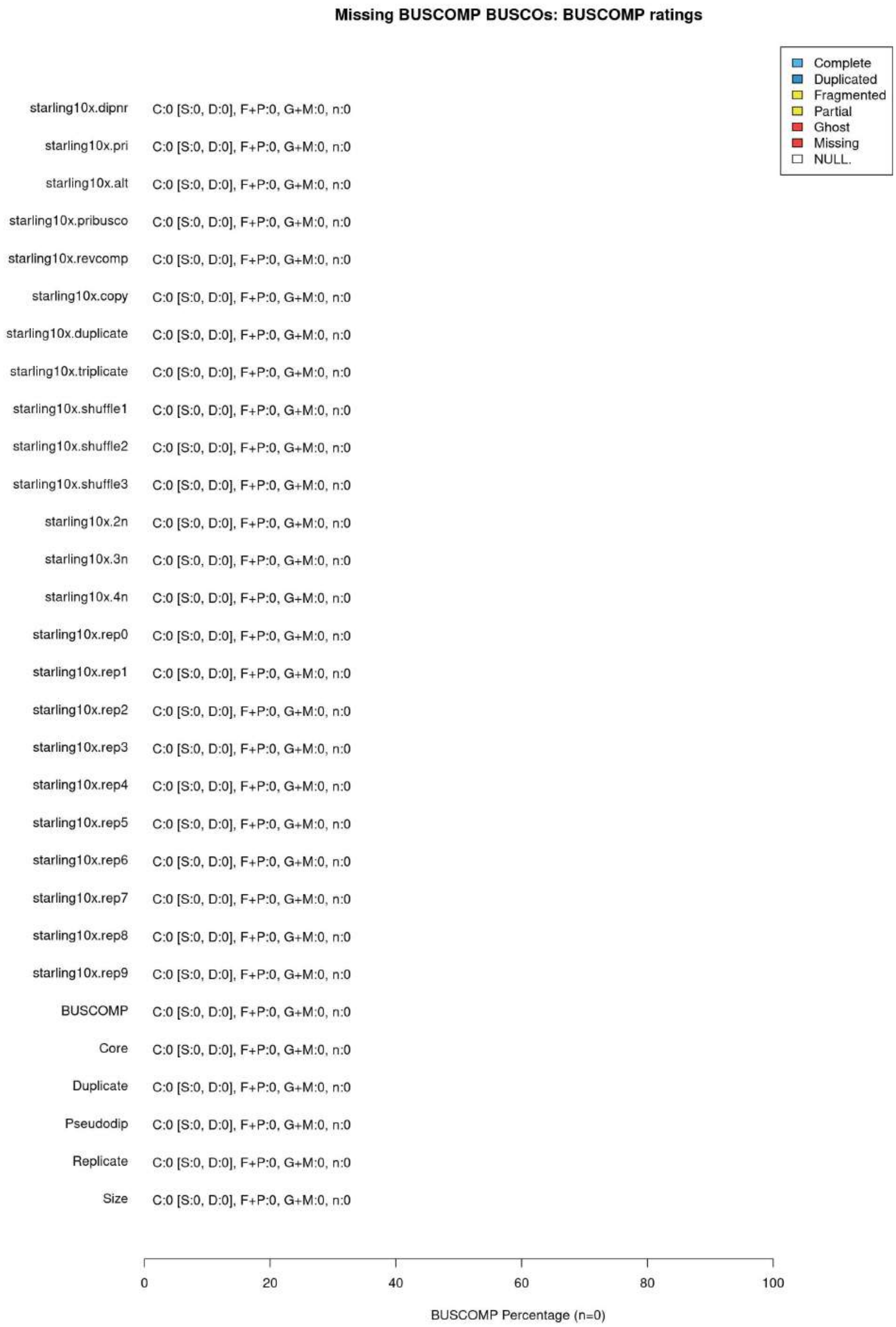

BUSCOMP ratings for `Missing` BUSCOMP BUSCOMP genes:

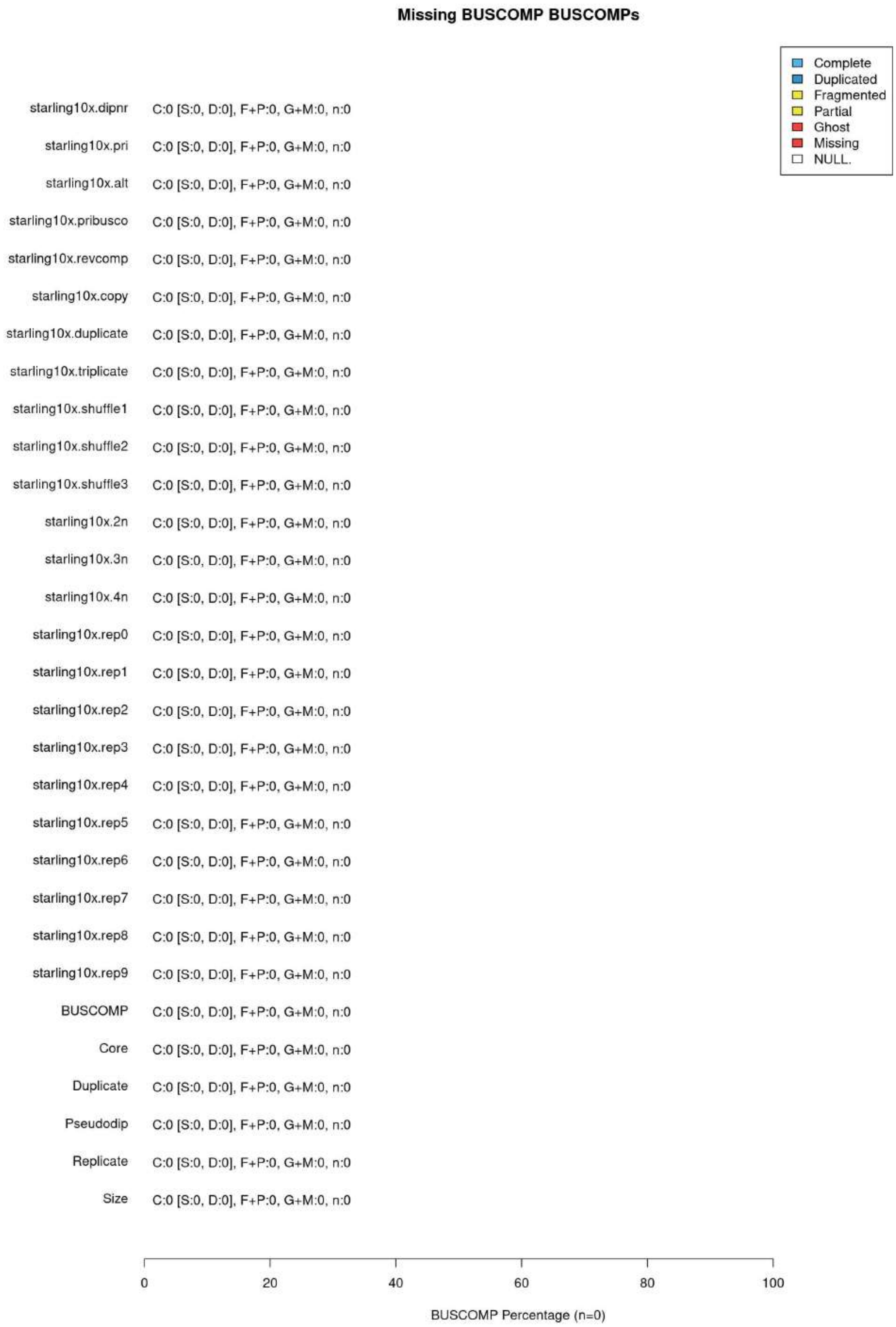

6 Appendix: BUSCOMP run details

```
BUSCOMP V0.11.0: run Thu Mar  4 16:10:40 2021
```

This analysis was run in:

```
/scratch/tmp/z3452659/projects/BUSCOMP-Jan19/analysis/2021-03-03.10xStarling/buscompV5
```

- Log file: `/scratch/tmp/z3452659/projects/BUSCOMP-Jan19/analysis/2021-03-03.10xStarling/buscompV5/starling10xV5.log` **(/scratch/tmp/z3452659/projects/BUSCOMP-Jan19/analysis/2021-03-03.10xStarling/buscompV5/starling10xV5.log)**
- Commandline arguments: `runs=../busco5/run_*` `fastadir=../fasta/` `ratefas=../fasta/*.fasta` `genomesize=1.1e9` `basefile=starling10xV5` `forks=24`
- Full Command List: `mafft=mafft` `clustalo=clustalo` `clustalw=clustalw2` `blast+path=` `iupath=/home/z3452659/bioware/iupred/iupred` `iuchdir=T` `minregion=5` `iucut=0.2` `rpath=R` `modpurge=T` `runs=../busco5/run_*` `fastadir=../fasta/` `ratefas=../fasta/*.fasta` `genomesize=1.1e9` `basefile=starling10xV5` `forks=24`

6.1 BUSCOMP errors

BUSCOMP returned no runtime errors.

6.2 BUSCOMP warnings

See run log for further details:

```
#WARN   00:32:40    116 Length/AlnLen mismatches from PAF file. (May be disparity with N treatment.)
#WARN   00:32:40    71 Identity mismatches from PAF file (PAF identity ignores Ns).
#WARN   00:43:06    74 Length/AlnLen mismatches from PAF file. (May be disparity with N treatment.)
#WARN   00:43:06    51 Identity mismatches from PAF file (PAF identity ignores Ns).
#WARN   00:49:04    37 Length/AlnLen mismatches from PAF file. (May be disparity with N treatment.)
#WARN   00:49:04    26 Identity mismatches from PAF file (PAF identity ignores Ns).
#WARN   00:53:45    49 Length/AlnLen mismatches from PAF file. (May be disparity with N treatment.)
#WARN   00:53:45    38 Identity mismatches from PAF file (PAF identity ignores Ns).
#WARN   01:01:44    58 Length/AlnLen mismatches from PAF file. (May be disparity with N treatment.)
#WARN   01:01:44    32 Identity mismatches from PAF file (PAF identity ignores Ns).
#WARN   01:09:08    49 Length/AlnLen mismatches from PAF file. (May be disparity with N treatment.)
#WARN   01:09:08    38 Identity mismatches from PAF file (PAF identity ignores Ns).
#WARN   01:15:14    96 Length/AlnLen mismatches from PAF file. (May be disparity with N treatment.)
#WARN   01:15:14    75 Identity mismatches from PAF file (PAF identity ignores Ns).
#WARN   01:26:39    155 Length/AlnLen mismatches from PAF file. (May be disparity with N treatment.)
#WARN   01:26:39    110 Identity mismatches from PAF file (PAF identity ignores Ns).
#WARN   01:48:26    47 Length/AlnLen mismatches from PAF file. (May be disparity with N treatment.)
#WARN   01:48:26    33 Identity mismatches from PAF file (PAF identity ignores Ns).
#WARN   01:55:35    48 Length/AlnLen mismatches from PAF file. (May be disparity with N treatment.)
#WARN   01:55:35    33 Identity mismatches from PAF file (PAF identity ignores Ns).
#WARN   02:03:24    48 Length/AlnLen mismatches from PAF file. (May be disparity with N treatment.)
#WARN   02:03:24    37 Identity mismatches from PAF file (PAF identity ignores Ns).
#WARN   02:08:31    49 Length/AlnLen mismatches from PAF file. (May be disparity with N treatment.)
#WARN   02:08:31    38 Identity mismatches from PAF file (PAF identity ignores Ns).
#WARN   02:13:47    49 Length/AlnLen mismatches from PAF file. (May be disparity with N treatment.)
#WARN   02:13:47    38 Identity mismatches from PAF file (PAF identity ignores Ns).
#WARN   02:19:04    49 Length/AlnLen mismatches from PAF file. (May be disparity with N treatment.)
#WARN   02:19:04    38 Identity mismatches from PAF file (PAF identity ignores Ns).
#WARN   02:24:21    49 Length/AlnLen mismatches from PAF file. (May be disparity with N treatment.)
#WARN   02:24:21    38 Identity mismatches from PAF file (PAF identity ignores Ns).
#WARN   02:29:50    49 Length/AlnLen mismatches from PAF file. (May be disparity with N treatment.)
#WARN   02:29:50    38 Identity mismatches from PAF file (PAF identity ignores Ns).
#WARN   02:34:59    49 Length/AlnLen mismatches from PAF file. (May be disparity with N treatment.)
#WARN   02:34:59    38 Identity mismatches from PAF file (PAF identity ignores Ns).
#WARN   02:40:18    49 Length/AlnLen mismatches from PAF file. (May be disparity with N treatment.)
#WARN   02:40:18    38 Identity mismatches from PAF file (PAF identity ignores Ns).
#WARN   02:45:41    49 Length/AlnLen mismatches from PAF file. (May be disparity with N treatment.)
#WARN   02:45:41    38 Identity mismatches from PAF file (PAF identity ignores Ns).
#WARN   02:50:53    49 Length/AlnLen mismatches from PAF file. (May be disparity with N treatment.)
#WARN   02:50:53    38 Identity mismatches from PAF file (PAF identity ignores Ns).
#WARN   02:56:19    49 Length/AlnLen mismatches from PAF file. (May be disparity with N treatment.)
#WARN   02:56:19    38 Identity mismatches from PAF file (PAF identity ignores Ns).
```

Report contents:

- **Run summary**
- **BUSCOMP summary**
- **Genome summary**
- **BUSCO Ratings**
- **BUSCO full results compilation**
- **BUSCOMP Sequence details and rating**
- **BUSCOMP re-rating of genomes**
- **BUSCOMP re-rating full results**
- **Unique BUSCO and BUSCOMP Complete genes**
- **Ratings for Missing BUSCO genes**
- **Appendix: BUSCOMP run details**

Output generated by BUSCOMP v0.11.0 © 2019 Richard Edwards | **richard.edwards@unsw.edu.au (mailto:richard.edwards@unsw.edu.au)**
