# Supplementary material for: Transcript‐ and annotation‐guided genome assembly of the European starling
Source: Mol Ecol Resour. 2022 Jul 18;22(8):3141–60. doi: 10.1111/1755-0998.13679 (PMC9796300; doi:10.1111/1755-0998.13679)
Supplement: Supplementary file 2 — Appendix S2 [file MEN-22-3141-s002.docx]

**Transcript- and annotation-guided genome assembly of the European starling**

Katarina C. Stuart^1†^, Richard J. Edwards^2†^, Yuanyuan Cheng^3^, Wesley C. Warren^4^, David W. Burt^5^, William B. Sherwin^1^, Natalie R. Hofmeister^6,7^, Scott J. Werner^8^, Gregory F. Ball^9^, Melissa Bateson^10^, Matthew C. Brandley^11^, Katherine L. Buchanan^12^, Phillip Cassey^13^, David F. Clayton^14^, Tim De Meyer^15^, Simone L. Meddle^16^, Lee A. Rollins^1^

^1^ Evolution & Ecology Research Centre, School of Biological, Earth and Environmental Sciences, UNSW Sydney, Sydney, New South Wales, Australia

^2^ School of Biotechnology and Biomolecular Sciences, UNSW Sydney, Sydney, New South Wales, Australia

^3^ School of Life and Environmental Sciences, The University of Sydney, Sydney, New South Wales, Australia

^4^ Department of Animal Sciences, Institute for Data Science and Informatics, The University of Missouri, Columbia, Missouri, USA

^5^ Office of the Deputy Vice-Chancellor (Research and Innovation), The University of Queensland, Brisbane, Australia

^6^ Department of Ecology and Evolutionary Biology, Cornell University, Ithaca, NY 14850

^7^ Fuller Evolutionary Biology Program, Cornell Lab of Ornithology, Ithaca, NY 14850

^8^ United States Department of Agriculture, Animal and Plant Health Inspection Service, Wildlife Services, National Wildlife Research Center, Fort Collins, Colorado, USA.

^9^ Department of Psychology, University of Maryland, College Park, MD 20742 USA

^10^ Institute of Neuroscience, Newcastle University, Newcastle upon Tyne, UK

^11^ Carnegie Museum of Natural History, Pittsburgh, Pennsylvania, USA

^12^ School of Life and Environmental Sciences, Deakin University, Waurn Ponds, VIC, 3228, Australia
^13^ Invasion Science & Wildlife Ecology Lab, University of Adelaide, Adelaide SA 5005, Australia

^14^ Department of Genetics & Biochemistry, Clemson University, South Carolina 29634

^15^ Dept. of Data Analysis & Mathematical Modelling, Faculty of Bioscience Engineering, Ghent University, Ghent, Belgium

^16^ The Roslin Institute, The Royal (Dick) School of Veterinary Studies, The University of Edinburgh, Midlothian, EH25 9RG, UK

^†^ Joint first authors

**Supplementary Materials:**

**Appendix 1: Transcriptome sample collection, RNA extraction, and sequencing**

An adult male European starling (non-breeding condition, black beak) was collected (by a private landowner as part of local pest control measures) via trapping from Wee Waa in New South Wales, Australia (30.2266° S, 149.4455° E) during late summer, with access to food and water. Within 24 hours of capture, the bird was euthanized, and we immediately dissected the brain, testes, and heart, and placed these in RNA-later, and kept cold (3-4^o^C) for one week. We extracted RNA from whole brain, testes, and heart tissue using the RNeasy kit as per the manufacturer’s instructions (including DNA removal using RNase-free DNase treatment) (Qiagen, Hilden, Germany). We assessed RNA quality and purity using a Nanodrop spectrophotometer (Thermo Fisher Scientific, Victoria, Australia) and Qubit 4.0 (RNA IQ Assay Kit) (Invitrogen, California, United States). One cDNA synthesis reaction was carried out for each sample, with approximately 2 μg of total RNA used as starting material for the brain and heart and 1 μg for the testis. For the endpoint amplification, two parallel PCRs were carried out for each sample using the TeloPrime PCR Add-on Kit V2 (Lexogen) with 20 cycles and 2 μl of synthesised cDNA per reaction as template. The PCR products from the two reactions were pooled together and then split into two fractions, which were purified using 1x and 0.5x AMPure PB beads (Pacific Biosciences), respectively, and pooled at equal molarity. The heart and testis cDNA libraries were then combined at equal molarity. Three full-length cDNA libraries were constructed, one for each tissue, using the TeloPrime Full-Length cDNA Amplification Kit V2 (Lexogen) following the kit manual. For SMRTbell library prepartion, 740 ng and 677 ng of purified full-length cDNA were used for the brain and heart+testis libraries, respectively, as input for the SMRTbell Template Prep Kit 1.0 (Pacific Biosciences). Libraries were sequenced using full-length isoform transcript sequencing Iso-Seq program (PacBio, California, United States) on the PacBio Sequel 1 platform (software v6.0.0) using the V3 chemistry. The polymerase-bound libraries were sequenced on 1 SMRT Cell each with a 20 h movie time plus a 4 h pre-extension time using the Sequel Sequencing Kit 3.0 (PacBio, 101-597-900) and a Sequel SMRT Cell 1M v3 LR (PacBio, 101-531-001). Sequencing was performed at the Institute for Molecular Bioscience Sequencing Facility (University of Queensland; https://imb.uq.edu.au/sequencing-facility).

**Appendix 2: Genomic DNA sample collection, gDNA extraction, and sequencing**

A juvenile male starling (non-reproductive state) was collected (by a private landowner as part of local pest control measures) from Orange in New South Wales, Australia (33.2833° S, 149.1000° E), euthanized, immediately dissected, and the brain tissue was placed in RNALater, kept cold (3-4^o^C) for two weeks, and then stored for 2 months at -70^o^C. We extracted high molecular weight gDNA from the approximately 1g of brain tissue using the Gentra Puregene Tissue Kit (Qiagen, Hilden, Germany), as per the manufacturer’s instructions. We checked the gDNA purity using a Nanodrop spectrophotometer (Thermo Fisher Scientific). We assessed fragment sizes using a Fragment Analyser (Millennium Science, Victoria, Australia) with an HS Large Fragment 50kb Kit (Agilent, California, USA). High molecular weight gDNA (1 ug) was prepared for 10x Chromium linked-read sequencing according to the manufacturer's recommended protocols. A 10x GEM library was barcoded using the Chromium Genome Reagent Kits (v2 Chemistry). The library was run on a single lane of a S4 flowcell and sequenced using the Illumina Hiseq X Ten sequencing platform (150 bp paired end reads).

High molecular weight gDNA (1 µg) was re-extracted for long read ONT (Oxford Nanopore Technologies, Oxford, United Kingdom) sequencing from the same brain tissue, using the 1D genomic DNA by ligation kit (SQK-LSK109, ONT) according to the standard protocol (with the inclusion of long fragment buffer). 500 ng of DNA was loaded onto two r9.4 minion flow cells and sequenced on an ONT MinION (Oxford Nanopore Technologies, Oxford, United Kingdom). Raw reads were converted to FASTA format, and filtered (guppy_basecaller parameters: min_qscore 7, filtlong parameters: min_mean_q 93, min_length 3000) with Guppy (v.3.2.1) (Oxford Nanopore Technologies) using the high-accuracy flip-flop model (config file: dna_r9.4.1_450bps_hac.cfg).

**Appendix 3: Validation of Supernova genome size prediction using Jellyfish**

To confirm that the genome size estimate of 1.19 Gb produced by Supernova (v2.1.1) (Weisenfeld *et al.* 2017) was correct, we used k-mer frequency analysis through Jellyfish (all k-mers counted) to manually confirm the estimated length of the genome based on the linked-read gDNA data. A k-mer histogram was produced using all the linked read gDNA raw data for an initial value of 20-mer based on approximated genome size of just above 1 Gb (Fig. S1). Counts for k-mer values of 7 or below (i.e., before the trough/red line) where removed as these are often attributed to either extremely rare reads, or random sequencing errors (Fig. S1). The genome size was estimated then by finding the total number of k-mers over all k values and dividing this by the point of mean coverage (Fig. S1, peak denoted by blue line; mean coverage = 32).

Genome Length = 35823112660 / 32

= 1,119,472,271 bp

The final genome assembly size was used to find the using the genome size k-mer estimate.

1,049,838,585/1,119,472,271 = 93.78% complete


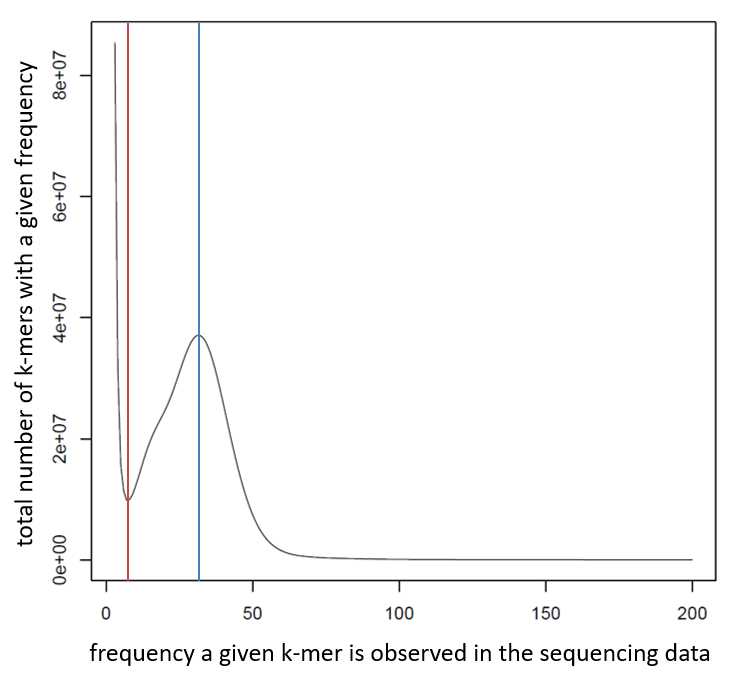


**Figure S1: Histogram of *Sturnus vulgaris* linked read gDNA Km-er counts** as calculated by Jellyfish from k=7 to k=200. Vertical red line denotes histogram peak at k=37.


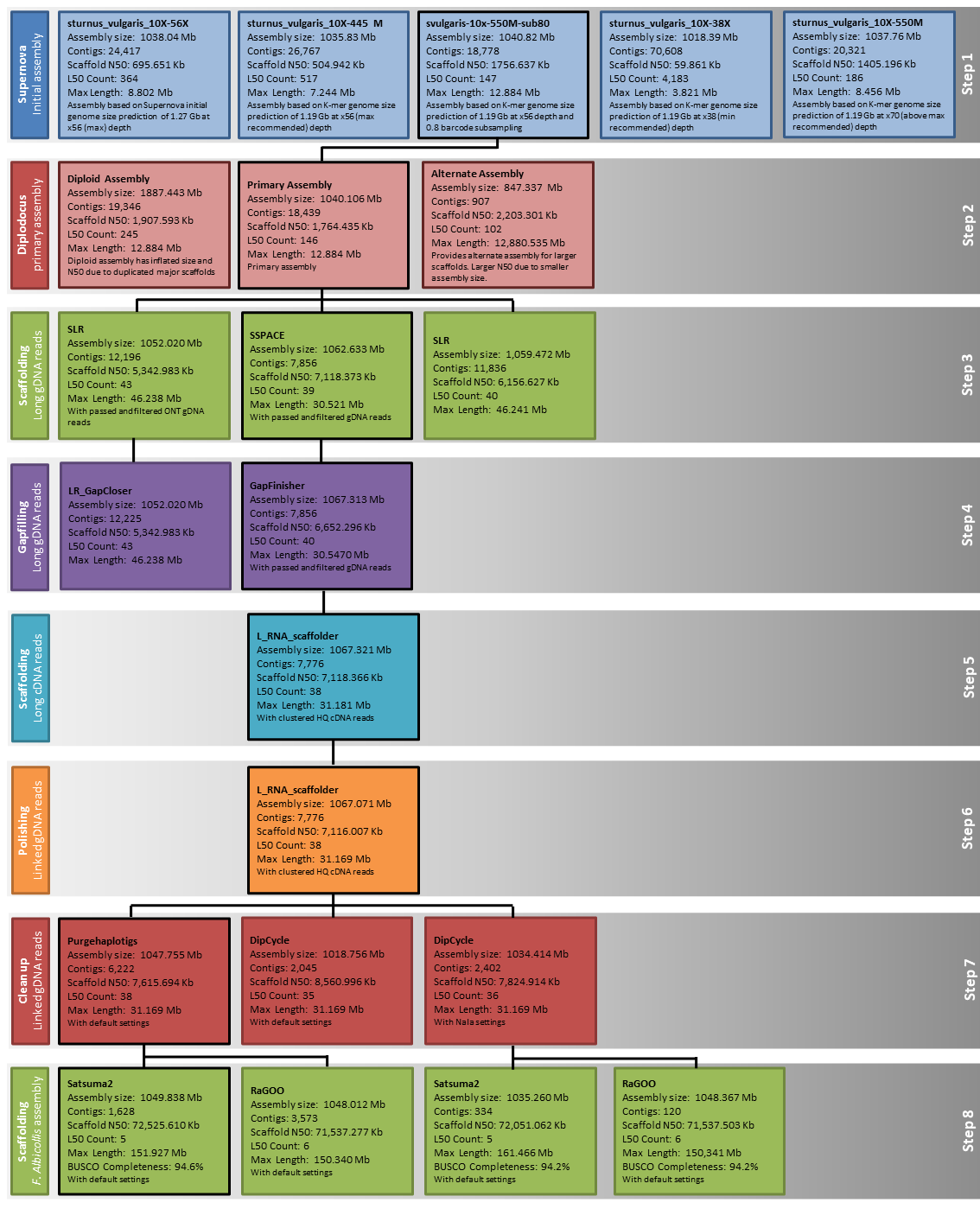


**Figure S2: Flow diagram of the *Sturnus vulgaris* vAU assembly process** covering initial assembly to final chromosome scaffolding, for a total of eight assembly steps; 1) initial assembly, 2) primary assembly, 3) ONT scaffolding, 4) gap-filling, 5) Iso-Seq scaffolding, 6) polishing, 7) cleanup, and 8) chromosome scaffolding.

**Appendix 4: Assembly and annotation of the *Sturnus vulgaris* vNA genome version**

**Materials and Methods:**

***Sample Collection, DNA extraction, and sequencing:*** The S. vulgaris DNA for shotgun sequencing was derived from an adult male (*Sturnus vulgaris*; bird ID 715) collected from North America. Total sequence genome input coverage on the Illumina HiSeq instrument was approx. 74x (39x fragments, and 35x 3kb) using a genome size estimate of 1Gb.

***Genome assembly and scaffolding****:* The combined sequence reads were assembled using ALLPATHS-LG software (Gnerre *et al.* 2011). This 1.0 version has been cleaned of contaminating contigs, and contigs 200 bp and less were removed.

***Genome Annotation and Functional Annotation****:* We annotated the *S. vulgaris* vNA genome using the same pipeline described in the main manuscript, with the only change being that a custom Augustus species profile was not trained for the Maker potion of the annotation.

**Results**

***Assembly:*** The assembly is made up of a total of 2361 scaffolds (including single contig scaffolds) with an N50 scaffold length of 3.6Mb (N50 contig length was 152kb). The assembly spans 1.01 Gb. The *S. vulgaris* reference genome can be downloaded on Genbank (Accession GCF_001447265.1).

***Annotation:*** The initial annotation produced by gemoma reported 20414 genes, with 77.2% BUSCO completeness (Figure S7). The initial Maker2 annotation reported 15150 genes, and a BUSCO completeness of 97.6% (Figure S7). The merged final annotation reported a BUSCO completeness of 98.5% (Figure S7), containing 21,944 genes, and 81,714 mRNAs. There was an average of 11.8 exons and 10.8 introns per gene, with an average intron length of 3343. Of these, 1,122 were single exon genes and 2,018 were single-exon mRNAs. Predicted coding sequences made up 5.5% of the assembly, and 40.91% of the remaining sequences were unannotated.

**Appendix 5: BUSCO versus BUSCOMP performance benchmarking**

BUSCO predictions are susceptible to base calling errors and can also fluctuate due to changes elsewhere in the genome assembly (Edwards 2019). As a consequence, BUSCO can under-report the true number of complete BUSCO genes in an assembly (Edwards et al., 2018; Field et al., 2020; Edwards et al., 2021). We therefore present to tool BUSCOMP

**Benchmarking methods**

BUSCO-containing scaffolds from the Diploidocus primary haploid Supernova assembly of *Sturnus vulgaris* vAU were extracted into a reduced genome ‘pribusco’ assembly for additional BUSCO and BUSCOMP benchmarking (Supplementary Materials: Fig. S3). BUSCO v3.0.2b (Simão et al., 2015) (HMMER v3.2.1 (Wheeler & Eddy 2013), AUGUSTUS v3.3.2 (Stanke & Morgenstern 2005), BLAST+ v2.2.31 blast(Camacho et al., 2009), EMBOSS v6.6.0 (Rice et al., 2000)) was run in genome mode with the aves_odb9 dataset (n=4915) on: the non-redundant pseudodiploid (‘dipnr’), primary (‘pri’) and alternative (‘alt’) assemblies; BUSCO-containing scaffolds from the primary assembly (‘pribusco’); a reverse-complemented copy (‘revcomp’), combined with ‘pribusco’ to make a 100% duplicate assembly (‘duplicate’); a direct copy (‘copy’) combined with ‘duplicate’ to make a triplicated assembly (‘triplicate’); three randomly shuffled versions of ‘pribusco’ (‘shuffle1’, ‘shuffle2’, ‘shuffle3’), added in combination to `pribusco` to generate datasets of increasing assembly size without increasing duplication levels (‘2n’, ‘3n’ and ‘4n’); ten straight repeats of the `pribusco` run (`rep0` to `rep9`). All BUSCO results were processed with BUSCOMP v0.11.0 (Minimap2 v2.17). In addition to the full BUSCOMP analysis of all runs, the following subsets were grouped for analysis (Supplementary File 1, BUSCO v3 BUSCOMP output):

- Pseudodip: ‘dipnr’, ‘pri’ and ‘alt’. (Haploid versus diploid assemblies.)
- Core: ‘dipnr’, ‘pri’, ‘alt’, ‘pribusco’ and ‘revcomp’. (Assembly filtering and manipulation.)
- Duplication: ‘copy’, ‘duplicate’, ‘triplicate’. (Duplicating scaffolds.)
- Size: ‘shuffle1’, ‘shuffle2’, ‘shuffle3’, ‘2n’, ‘3n’, ‘4n’. (Increasing assembly size without duplication.)
- Replicates: ‘rep0’ to ‘rep9’.

The same analysis was repeated with BUSCO v5.0.0 (Simão et al., 2015) (SEPP v4.3.10 (Mirarab et al., 2012), BLAST v2.11.0 (Camacho et al., 2009), HMMer v3.3 (Wheeler & Eddy 2013), AUGUSTUS v3.3.2 (Stanke & Morgenstern 2005), Prodigal v2.6.3 (Hyatt et al., 2010), metaeuk v20200908 (Levy Karin et al., 2020)) and the aves_odb10 dataset (n=8338).

**Figure S3: BUSCO and BUSCOMP benchmarking datasets.** The Supernova pseudohap2 assembly was processed with Diploidocus to generate a non-redundant pseudodiploid assembly (‘dipnr’) consisting of a primary (‘pri’) and alternative (‘alt’) assembly. BUSCO-containing scaffolds from the primary assembly were extracted into a reduced assembly (‘pribusco’) that was used to generate additional test data. **Duplication.** A reverse-complemented copy (‘revcomp’) was generated and combined with ‘pribusco’ to make a 100% duplicate assembly (‘duplicate’). An additional direct copy (‘copy’) was then added to make a triplicated assembly (‘triplicate’). **Assembly size.** Three randomly shuffled versions of ‘pribusco’ were generated (‘shuffle1’, ‘shuffle2’, ‘shuffle3’) and added in combination to `pribusco` to generate datasets of increasing assembly size without increasing duplication levels (‘2n’, ‘3n’ and ‘4n’). **Reproducibility.** Ten straight repeats of the `pribusco` BUSCO run were also performed (`rep0` to `rep9`).

**Benchmarking Results**

For the non-redundant pseudodiploid *S. vulgaris* vAU Supernova assembly, BUSCOMP revealed differences in the BUSCO ratings of scaffolds dependent on the assembly background (Fig. B2). Despite the primary (‘pri’) assembly being a subset of the non-redundant pseudodiploid (‘dinpnr’) assembly, it identified more “Complete” BUSCO genes (4,565 versus 4,532) with fewer “Missing” (131 versus 171) (Fig. B2a). The alternative assembly (‘alt’) subset similarly returned a partially overlapping set of BUSCO genes with ‘dipnr’, including some not found in ‘dipnr’ or ‘pri’: in total, only 101 genes were missing from all three assemblies. Reducing the primary assembly to the 968 (of 18,439) scaffolds containing a complete BUSCO gene (‘pribusco’), increased the number of complete genes from 4,565 to 4,586 and reduced the number missing from 131 to 112. Most unexpectedly, reverse complementing these scaffolds reduced the number of BUSCO genes rated “Complete” by two, and increased the number “Missing” by fifteen (Fig. B2a). All five assemblies returned complete BUSCO genes that were fragmented or missing in all the other four assemblies (Supplementary File 1, BUSCOMP v3 results), for a combined total of 4,760 complete and only 74 missing.

Adding direct or reverse-complemented copies of the ‘pribusco’ scaffolds increased the number of “Duplicated” genes, but still returned single copy complete genes (Fig. B2b). Doubling and then tripling the assembly size also increased the number of “Missing” genes from 112 to 198 (‘duplicate’) and then 207 (‘triplicate’). As before, these summary numbers hide some gene gains as well as gene losses; only 77 genes are missing from all three BUSCO runs, with 4,750 returned as complete by at least one. Adding randomly shuffled versions of the ‘pribusco’ scaffolds only had a marginal effect on BUSCO ratings, with four (‘2n’) to five (‘3n’, ‘4n’) fewer complete genes returned and seven additional genes missing following addition of the random sequences (Fig. B2c). Ten replicate analyses of the `pribusco` scaffolds returned identical results (Supplementary File 1, BUSCOMP v3 results).

In contrast, BUSCOMP completeness is much more consistent across all datasets, with the primary assembly returning the same numbers of complete, partial/fragmented and missing genes as the pseudodiploid assembly (Supplementary File 1, BUSCOMP v3 results). Similarly, reverse complementing scaffolds or increasing genome size gives no difference to the completion statistics. Unlike BUSCO, BUSCOMP rates 100% of complete BUSCO genes for duplicated or triplicated scaffolds as ‘Duplicate’ rather than ‘Single Copy’. Most reassuringly, every complete BUSCO gene returned by a variant or subset of the pseudodiploid assembly is also returned as ‘Complete’ in the pseudodiploid assembly itself. Results using BUSCO v5 and the updated lineage data were qualitatively the same as v3, showing largely identical trends (Supplementary File 2, BUSCOMP v5 results). The exception is that reverse-complementing scaffolds reduced the complete BUSCO genes by one (7,555 to 7,554) and increased the number missing by one (391 to 392). Curiously, this was not reflected by analysis of the duplicated scaffolds, in which all 7,555 ‘pribusco’ complete genes were returned as complete and duplicated. It should be noted that the ‘pribusco’ scaffolds for the v5 analysis are missing a greater proportion of the BUSCOMP-compiled single copy complete BUSCO genes because they were still defined from v3 data.


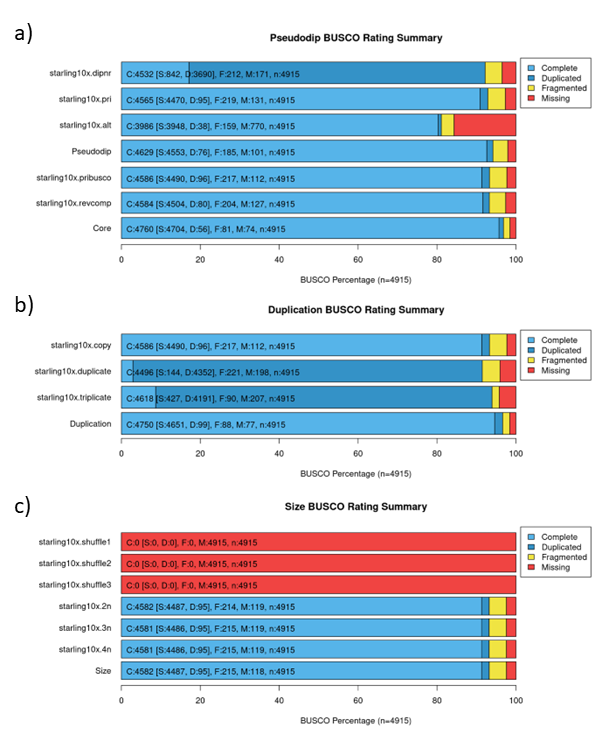


**Figure S4. Compiled BUSCO results for benchmarking data.**

**Table S1: GeMoMa Ensembl reference species** used in GeMoMa annotation of *Sturnus vulgaris* vAU and vNA. Retrieved 18 Nov 2020.

| **Common Name** | **Scientifiec Name** | **Ensembl Assembly** |
| --- | --- | --- |
| Eurasian sparrowhawk | *Accipiter nisus* | Accipiter_nisus_ver1.0 |
| Yellow-billed parrot | *Amazon collaria* | ASM394721v1 |
| Mallard | *Anas platyrhynchos* | ASM874695v1 |
| Pink-footed goose | *Anser brachyrhynchus* | ASM259213v1 |
| Swan goose | *Anser cygnoides* | GooseV1.0 |
| Greater spotted kiwi | *Apteryx haastii* | aptHaa1 |
| Little spotted kiwi | *Apteryx owenii* | aptOwe1 |
| Okarito brown kiwi | *Apteryx rowi* | aptRow1 |
| Golden eagle | *Aquila chrysaetos chrysaetos* | bAquChr1.2 |
| Burrowing owl | *Athene cunicularia* | athCun1 |
| Small tree finch | *Camarhynchus parvulus* | STF_HiC |
| Golden pheasant | *Chrysolophus pictus* | Chrysolophus_pictus_GenomeV1.0 |
| Blue tit | *Cyanistes caeruleus* | cyaCae2 |
| Emu | *Dromaius novaehollandiae* | droNov1 |
| Gouldian finch | *Erythrura gouldiae* | GouldianFinch |
| Flycatcher | *Ficedula albicollis* | FicAlb_1.4 |
| Chicken | *Gallus gallus* | GRCg6a |
| Median ground-finch | *Geospiza fortis* | GeoFor_1.0 |
| Bengalese finch | *Lonchura striata domestica* | LonStrDom1 |
| Turkey | *Meleagris gallopavo* | Turkey_2.01 |
| Great tit | *Parus major* | Parus_major1.1 |
| Ring-necked pheasant | *Phasianus colchicus* | ASM414374v1 |
| Common canary | *Serinus canaria* | SCA1 |
| African ostrich | *Struthio camelus australis* | ASM69896v1 |
| Zebra finch | *Taeniopygia guttata* | bTaeGut1_v1.p |
| White-throated sparrow | *Zonotrichia albicollis* | Zonotrichia_albicollis-1.0.1 |

**Table S2: Read number and length after Iso-Seq analysis** from full-length transcriptome sequencing from *Sturnus vulgaris* brain, and heart and testes pooled.

|  | **Brain** | **Heart + Testis** |
| --- | --- | --- |
| **Raw Data** |  |  |
| Polymerase Read Bases | 39,550,574,401 | 30,329,883,755 |
| Polymerase Reads | 648,290 | 600,764 |
| Polymerase Read Length (Mean) | 61,008 | 50,486 |
| Polymerase Read N50 | 125,257 | 101,226 |
| Subread Length (mean) | 1,880 | 1,556 |
| Subread N50 | 2,069 | 1,719 |
| Insert Length (mean) | 2,985 | 2,625 |
| Insert N50 | 3,225 | 2,866 |
| **Primer removal + demultiplexing** |  |  |
| ZMWs input | 483167 | 446827 |
| ZMWs above all thresholds | 451260 (93%) | 419191 (94%) |
| ZMWs below any threshold | 31907 (7%) | 27636 (6%) |
| **Refine** |  |  |
| Number of reads | 446838 | 414411 |
| Number of reads (polya) | 445670 | 413023 |
| **Clustered** |  |  |
| High quality | 33454 | |
| High quality: mean length | 2005.79 | |
| Low quality | 157 | |
| **Tama Collapse** |  | |
| Non redundant transcripts | 28448 | |
| Non redundant transcripts: mean (bp) | 2014.43 | |


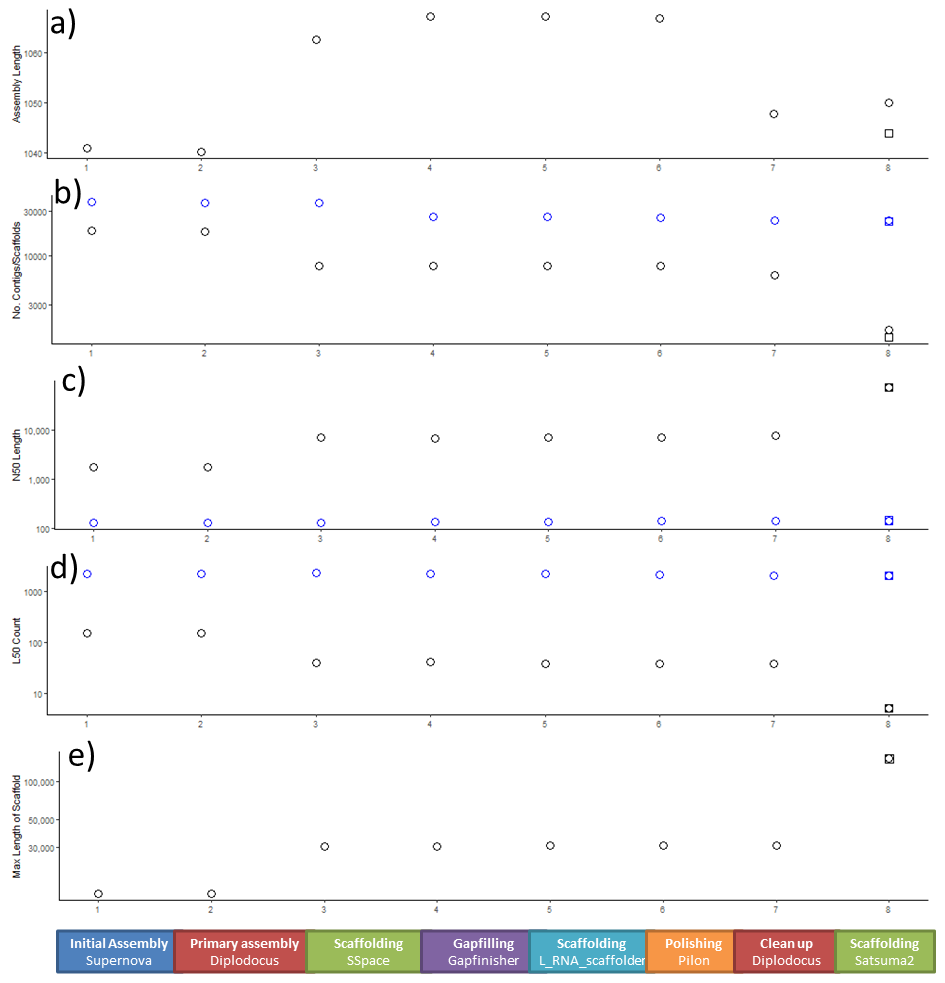


**Figure S5: *Sturnus vulgaris* vAU assessment statistics summary** for each of the eight assembly steps; 1) initial assembly, 2) primary assembly, 3) ONT scaffolding, 4) gap-filling, 5) Iso-Seq scaffolding, 6) polishing, 7) cleanup, and 8) chromosome scaffolding, with black indicating scaffolds and grey contigs, with vAU1.0 represented as circles, and vAU1.1 represented as squares.

**Table S3: NUMTfinder analysis offinal *Sturnus vulgaris* AU assembly,** identifying the location putative NUMT in the assembly.

| **SeqName** | **Start** | **End** | **Strand** | **BitScore** | **Expect** | **Length** | **Identity** | **mtStart** | **mtEnd** |
| --- | --- | --- | --- | --- | --- | --- | --- | --- | --- |
| SV_vAU_seq2 | 1.19E+08 | 1.19E+08 | - | 152 | 4.00E-33 | 302 | 216 | 6450 | 6751 |
| SV_vAU_seq4 | 47522543 | 47522606 | - | 72 | 1.00E-08 | 64 | 55 | 10558 | 10620 |
| SV_vAU_seq5 | 38040731 | 38040937 | - | 62 | 5.00E-06 | 210 | 142 | 15261 | 15470 |
| SV_vAU_seq5 | 1.01E+08 | 1.01E+08 | - | 198 | 1.00E-46 | 803 | 530 | 14706 | 15492 |
| SV_vAU_seq8 | 17525188 | 17525391 | + | 83 | 5.00E-12 | 208 | 145 | 15245 | 15451 |
| SV_vAU_seq8 | 25972974 | 25973368 | + | 189 | 5.00E-44 | 397 | 283 | 8702 | 9095 |
| SV_vAU_seq8 | 41187969 | 41188736 | + | 216 | 4.00E-52 | 814 | 545 | 14615 | 15413 |
| SV_vAU_seq9 | 17334457 | 17334507 | + | 70 | 3.00E-08 | 51 | 46 | 2525 | 2575 |
| SV_vAU_seq10 | 24554055 | 24554239 | - | 124 | 2.00E-24 | 192 | 144 | 12904 | 13094 |
| SV_vAU_seq11 | 355759 | 356192 | + | 291 | 6.00E-75 | 467 | 345 | 3622 | 4088 |
| SV_vAU_seq11 | 8642466 | 8642510 | + | 59 | 6.00E-05 | 45 | 40 | 2088 | 2132 |
| SV_vAU_seq11 | 30396630 | 30396961 | - | 91 | 1.00E-14 | 338 | 227 | 14615 | 14944 |
| SV_vAU_seq12 | 2721351 | 2722141 | - | 537 | 8.00E-149 | 838 | 617 | 2753 | 3578 |
| SV_vAU_seq12 | 4344254 | 4344336 | - | 65 | 1.00E-06 | 83 | 64 | 14345 | 14427 |
| SV_vAU_seq14 | 1657092 | 1657312 | - | 192 | 4.00E-45 | 221 | 175 | 8819 | 9039 |
| SV_vAU_seq14 | 4520796 | 4521008 | - | 133 | 3.00E-27 | 220 | 161 | 12804 | 13023 |
| SV_vAU_seq16 | 788033 | 788121 | + | 62 | 5.00E-06 | 89 | 67 | 10565 | 10653 |
| SV_vAU_seq24 | 2478314 | 2478498 | - | 69 | 3.00E-08 | 186 | 130 | 16408 | 16590 |
| SV_vAU_seq26 | 2392491 | 2392570 | - | 86 | 4.00E-13 | 80 | 67 | 14106 | 14185 |
| SV_vAU_seq26 | 2589746 | 2590569 | + | 159 | 2.00E-35 | 894 | 580 | 13616 | 14501 |
| SV_vAU_seq26 | 2696879 | 2697117 | + | 125 | 5.00E-25 | 240 | 173 | 16409 | 16646 |
| SV_vAU_seq31 | 37052129 | 37052981 | - | 829 | 0 | 863 | 705 | 11644 | 12504 |
| SV_vAU_seq31 | 42850393 | 42851068 | + | 369 | 3.00E-98 | 680 | 498 | 15153 | 15823 |
| SV_vAU_seq31 | 43349606 | 43349796 | - | 68 | 1.00E-07 | 193 | 133 | 4484 | 4675 |
| SV_vAU_seq31 | 62603293 | 62603703 | - | 84 | 2.00E-12 | 451 | 288 | 10162 | 10607 |
| SV_vAU_seq31 | 62603804 | 62604202 | + | 123 | 2.00E-24 | 425 | 282 | 4203 | 4622 |
| SV_vAU_seq31 | 68129474 | 68129742 | - | 96 | 2.00E-16 | 275 | 187 | 8025 | 8289 |

**Table S4: Assembly statistics summary** for sequential assembly steps of *Sturnus vulgaris vAU1.0*

|  | **Step 1:  Assembly** | **Step 2:  Primary Assembly** | **Step 3:  Long gDNA read Scaffolding** | **Step 4: Gapfilling** | **Step 5: Long cDNA read Scaffolding** | **Step 6: Polishing** | **Step 7: Tidy** | **Step 8: Chromosome Scaffolding** |
| --- | --- | --- | --- | --- | --- | --- | --- | --- |
| **Assembly continuity statistics** | | | | | | | | |
| Total number of sequences | 18,778 | 18,439 | 7,856 | 7,856 | 7,776 | 7,776 | 6,222 | 1,628 |
| Total length of sequences | 1,040,824,271 | 1,040,106,492 | 1,062,633,441 | 1,067,313,776 | 1,067,321,776 | 1,067,071,200 | 1,047,755,039 | 1,049,838,585 |
| Min. length of sequences | 1,000 | 1,000 | 1,000 | 977 | 977 | 842 | 917 | 927 |
| Max. length of sequences | 12,884,419 | 12,884,419 | 30,521,271 | 30,547,435 | 31,181,295 | 31,169,695 | 31,169,695 | 151,927,750 |
| Mean length of sequences | 55,427.86 | 56,407.97 | 135,263.93 | 135,859.70 | 137,258.46 | 137,226.23 | 168,395.22 | 644,864.00 |
| Median length of sequences | 2,121 | 2,153 | 2,433 | 2,433 | 2,395 | 2,394 | 2,199 | 1,337 |
| N50 length of sequences | 1,756,637 | 1,764,435 | 7,118,373 | 6,652,296 | 7,118,366 | 7,116,007 | 7,615,694 | 72,525,610 |
| L50 count of sequences | 147 | 146 | 39 | 40 | 38 | 38 | 37 | 5 |
| Total number of contigs | 37,718 | 37,354 | 36,809 | 26,531 | 26,531 | 25,946 | 23,815 | 23,815 |
| Contig N50 length of sequences | 132,941 | 132,973 | 131,172 | 138,163 | 138,163 | 143,478 | 146,413 | 145,864 |
| Contig L50 count of sequences | 2,176 | 2,174 | 2,239 | 2,151 | 2,151 | 2,079 | 2,023 | 2,030 |
| GC content | 41.64% | 41.64% | 41.68% | 41.81% | 41.81% | 41.82% | 41.73% | 41.73% |
| N bases | 6,838,110 (0.66%) | 6,835,790 (0.66%) | 23,897,712 (2.25%) | 11,590,876 (1.09%) | 11,598,876 (1.09%) | 11,396,143 (1.07%) | 11,158,567 (1.06%) | 13,242,113 (1.26%) |
| Gap (10+ N) length | 6,838,110 (0.66%) | 6,835,790 (0.66%) | 23,895,932 (2.25%) | 11,590,274 (1.09%) | 11,598,274 (1.09%) | 11,395,574 (1.07%) | 11,158,028 (1.06%) | 13,241,574 (1.26%) |
| Gap (10+ N) count | 18,940 | 18,915 | 28,953 | 18,675 | 18,755 | 18,170 | 17,593 | 22,187 |
| **BUSCO statistics** | | | | | | | | |
| Complete and single copy | 4,470 | 4,470 | 4,536 | 4,541 | 4,537 | 4,644 | 4,579 | 4,595 |
| Complete and duplicate | 95 | 95 | 101 | 99 | 99 | 98 | 61 | 54 |
| Fragmented Busco | 219 | 219 | 173 | 169 | 170 | 168 | 164 | 154 |
| Missing Busco | 131 | 131 | 104 | 143 | 107 | 103 | 110 | 112 |
| **Iso-Seq mapping statistics** | | | | | | | | |
| ISO-SEQ:Non-mapped transcripts | 264 | 264 | 267 | 244 | 247 | 246 | 246 | 241 |
| ISO-SEQ: Mapped transcripts (Quality = 60) | 32,685 | 32,693 | 32,600 | 32,565 | 32,572 | 32,578 | 32,898 | 32,864 |
| **BUSCOMP Statistics** | | | | | | | | |
| BUSCOMP: Complete and single copy | 4,530 | 4,530 | 4,632 | 4,640 | 4,642 | 4,642 | 4,660 | 4,702 |
| BUSCOMP: Complete and duplicate | 48 | 48 | 55 | 55 | 55 | 55 | 22 | 13 |
| BUSCOMP: Fragmented and Partial | 129 | 129 | 38 | 30 | 28 | 28 | 29 | 12 |
| BUSCOMP: Ghost and Missing | 20 | 20 | 2 | 2 | 2 | 2 | 16 | 0 |
| **SAAGA statistics** | | | | | | | | |
| SAAGA: mean protratio | 0.919338 | 0.919562 | 0.936762 | 0.937015 | 0.938079 | 0.937305 | 0.940878 | 0.946452 |
| SAAGA: protratio_median | 0.998066 | 0.998069 | 0.998674 | 0.998720 | 0.998779 | 0.998773 | 0.998924 | 0.999204 |
| SAAGA: protratio_sd | 0.216565 | 0.216359 | 0.192846 | 0.193475 | 0.192076 | 0.192694 | 0.186809 | 0.178068 |
| SAAGA: duplicity | 0.918160 | 0.917762 | 0.913638 | 0.914098 | 0.913664 | 0.915717 | 0.897704 | 0.897401 |
| SAAGA: mean F1 score | 0.906657 | 0.906810 | 0.922552 | 0.922834 | 0.923881 | 0.923425 | 0.927146 | 0.931119 |
| SAAGA: mean_f1 | 0.598339 | 0.598838 | 0.595627 | 0.586467 | 0.587486 | 0.588122 | 0.593248 | 0.598555 |
| **KAT statistics** | | | | | | | | |
| KAT: R1 reads | 96.54 | 96.54 | 96.54 | 96.61 | 96.61 | 96.73 | 96.24 | 96.69 |
| KAT: R2 reads | 95.01 | 95.77 | 95.77 | 95.84 | 95.84 | 95.96 | 95.46 | 95.92 |


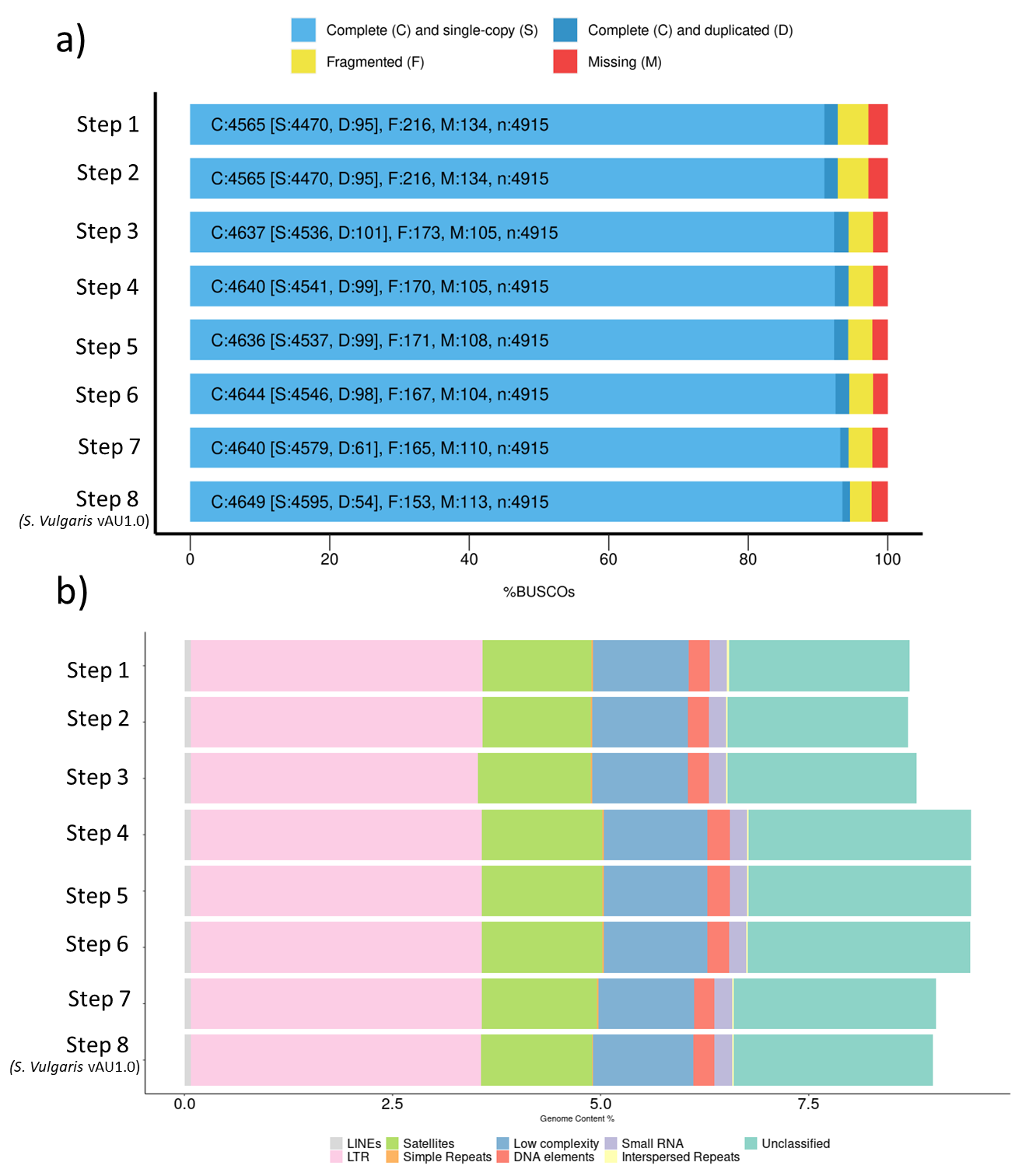


**Figure S6: *Sturnus vulgaris* vAU assembly steps overview.** Quality and completeness assessments for eight sequential assembly steps: step 1 (Supernova assembly), step 2 (Diplodocus primary assembly), step 3 (Sspace-Longreads scaffolding), step 4 (Gapfinisher gapfilling), step 5 (L_RNA_scaffolder), step 6 (Pilon polishing), step 7 (Diplodocus clean up), and step 8 (Satsuma2 Chromosome scaffolding). **a)** BUSCO (Aves, n=4,915) completeness rating summaries for the sequential steps of *S. vulgaris* genome assembly. **b)** Repeat content profile for the sequential steps of *S. vulgaris* genome assembly.


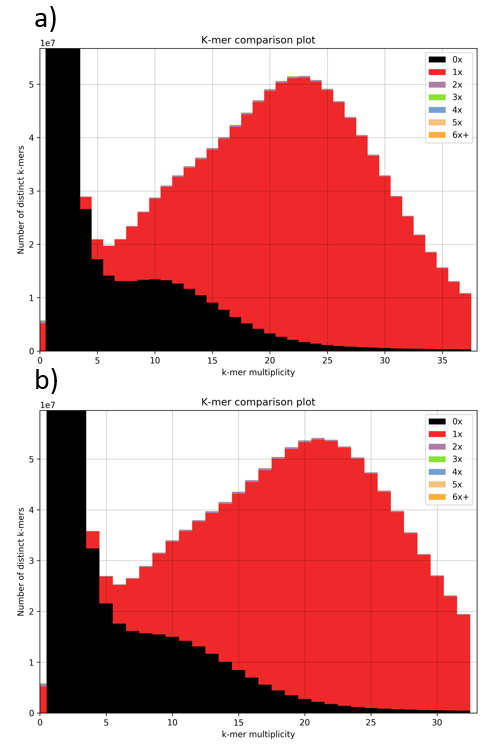


**Figure S7: KAT k-mer analysis of final *Sturnus vulgaris* AU assembly.** Plots depict read k-mer frequency distributions with different assembly copy numbers based on the 10X Chromium linked **a)** Reads 1 and **b)** Reads 2.

**Table S5:** **Summary of genome annotation of *Sturnus vulgaris* vAU and vNA** assemblies. Statistics extracted using AGAT *agat_sp_functional_statistics.pl*.

| **Genome Annotation** | |  | | *S. vulgaris* vAU | *S. vulgaris* vNA |
| --- | --- | --- | --- | --- | --- |
| **Genes** | **Total number** | | 21,863 | | 21,944 |
|  | Average length | | 34,699 bp | | 35,761 bp |
|  | mean mRNAs per gene | | 3.6 | | 3.7 |
| **mRNA** | **Total number** | | 79,359 | | 81,714 |
|  | Average length | | 38,073 bp | | 37,857 bp |
|  | mean exons per mRNA | | 11.8 | | 11.8 |
| **CDS** | **Total number** | | 79,359 | | 81,714 |
|  | Average length | | 1,851 | | 1,836 |
|  | Average intron in CDS length | | 3,364 | | 3,343 |
| **Exons** | **Total number** | | 933,014 | | 962,220 |
|  | Mean length | | 163 | | 158 |
| **Gene Function** | **Ontology Term** | | 60.26% (13174/21863) | | 59.68% (13097/21944) |
|  | **InterPro** | | 78.87% (17244/21863) | | 77.57% (17022/21944) |
|  | **SUPERFAMILY** | | 60.36% (13197/21863) | | 58.26% (12786/21944) |


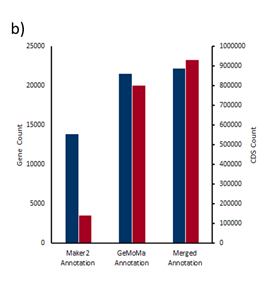


**Figure S8: *Sturnus vulgaris* assessment of annotation. b)** The number of genes (blue) and CDS (red) in the Maker2 annotation, GeMoMa annotation, and merged annotation.


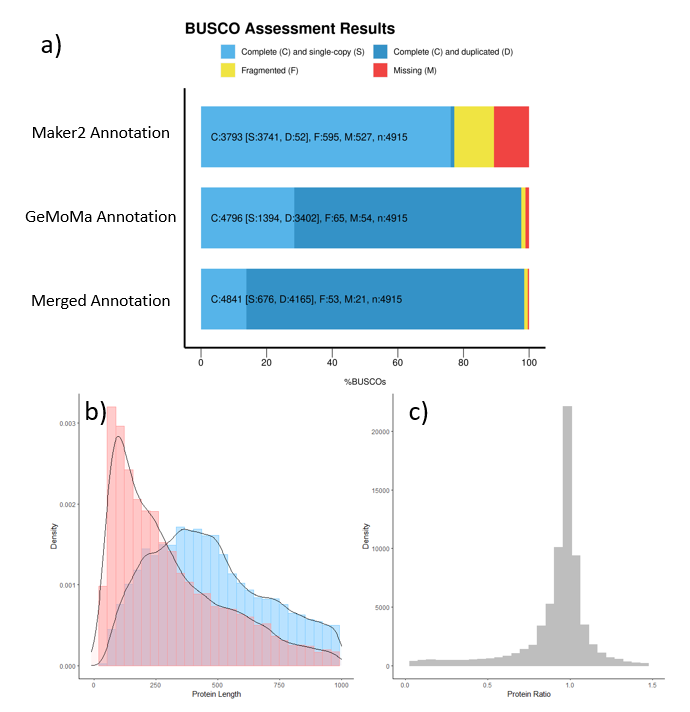


**Figure S9: Summary of *Sturnus vulgaris* vNA genome annotations**, with panel a) BUSCO assessments of initial Maker2 and GeMoMa assemblies, the final *S. vulgaris* vNA annotation (aves lineage). Panel b) depicts protein length of known (blue) and unknown (red) proteins for the merged GeMoMa and Maker2 annotation. Panel d) depicts the protein ratio between output from SAAGA for all known proteins for the merged annotation (where a score close to 1 indicates a high-quality gene annotation, protein ratio calculated as annotated protein length / best Swiss-Prot reference protein length).


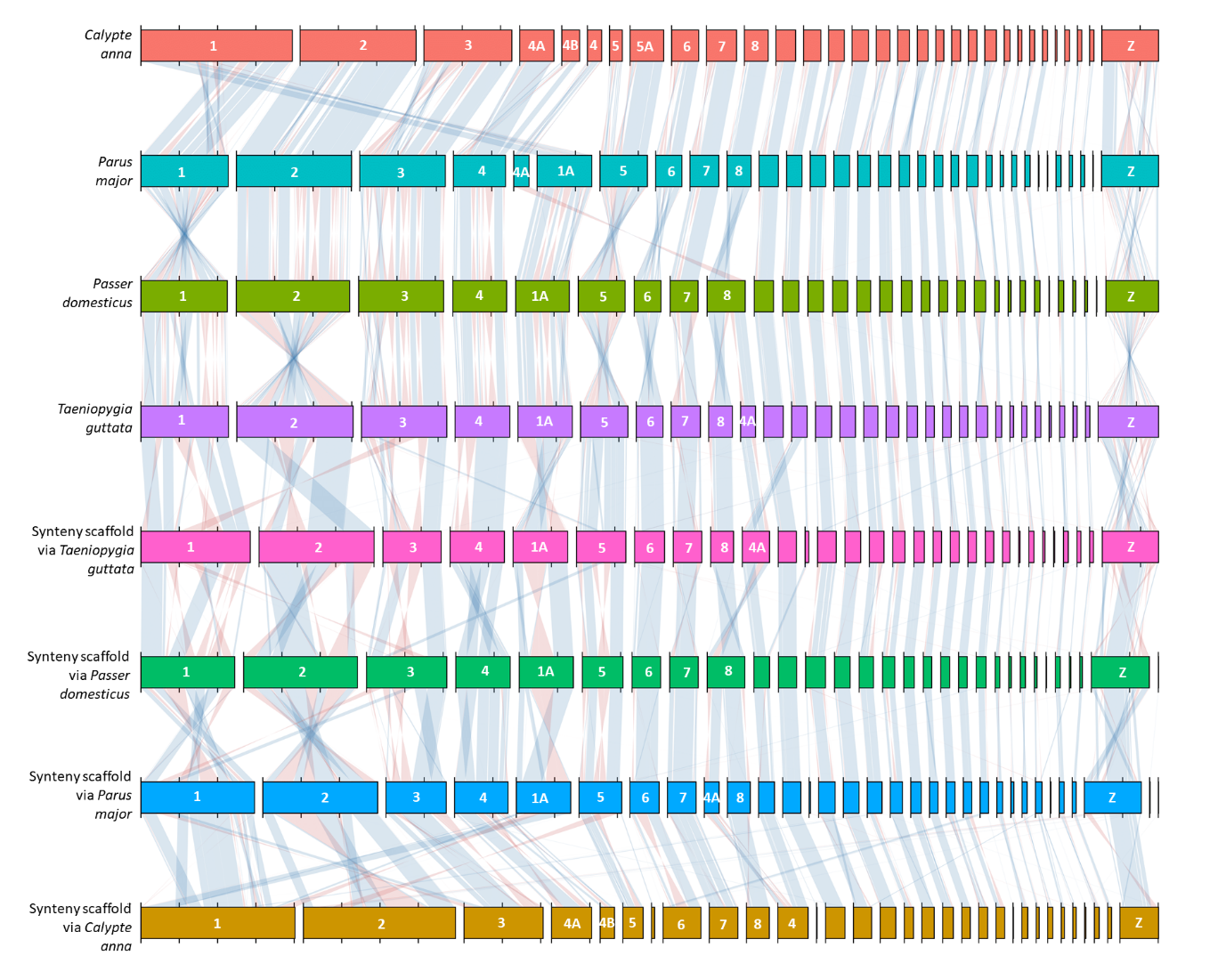


**Figure S10: Synteny analysis of four chromosome level Aves assemblies**. The first four tracks depict the chromosomes of the species’ assemblies, while the bottom four tracks depict the result after step 7 of the *Sturnus vulgaris* is synteny-based scaffolded using the indicated assembly (i.e. synteny scaffold via *T. guttata*  is *S. vulgaris* vAU). Synteny lines between tracks are calculated and plotted using Chromsyn, a BUSCO guided synteny plotting tool. Blue synteny lines indicate the genes occur on the same strand, while red indicates the gene location has switched to the reverse strand.

**References:**

Gnerre S, MacCallum I, Przybylski D, Ribeiro FJ, Burton JN, Walker BJ, Sharpe T, Hall G, Shea TP, Sykes S *et al.* 2011 High-quality draft assemblies of mammalian genomes from massively parallel sequence data. *Proceedings of the National Academy of Sciences* **108** 1513–1518. (doi:10.1073/pnas.1017351108)

Weisenfeld NI, Kumar V, Shah P, Church DM & Jaffe DB 2017 Direct determination of diploid genome sequences. *Genome Research* **27** 757–767. (doi:10.1101/gr.214874.116)
